# Base‐Activated Latent Heteroaromatic Sulfinates as Nucleophilic Coupling Partners in Palladium‐Catalyzed Cross‐Coupling Reactions

**DOI:** 10.1002/anie.202109146

**Published:** 2021-09-08

**Authors:** Xinlan A. F. Cook, Loïc R. E. Pantaine, David C. Blakemore, Ian B. Moses, Neal W. Sach, Andre Shavnya, Michael C. Willis

**Affiliations:** ^1^ Department of Chemistry University of Oxford Chemistry Research Laboratory Mansfield Road Oxford OX1 3TA UK; ^2^ Medicine Design Pfizer Inc. Eastern Point Road Groton CT 06340 USA; ^3^ Pharmaceutical sciences Pfizer Inc. Discovery Park, Ramsgate Road CT13 9ND UK; ^4^ Medicine Design, La Jolla Laboratories Pfizer Inc. 10777 Science Center Drive San Diego CA 92121 USA

**Keywords:** cross-coupling, desulfination, palladium, pyridine, sulfone

## Abstract

Heteroaromatic sulfinates are effective nucleophilic reagents in Pd^0^‐catalyzed cross‐coupling reactions with aryl halides. However, metal sulfinate salts can be challenging to purify, solubilize in reaction media, and are not tolerant to multi‐step transformations. Here we introduce base‐activated, latent sulfinate reagents: β‐nitrile and β‐ester sulfones. We show that under the cross‐coupling conditions, these species generate the sulfinate salt in situ, which then undergo efficient palladium‐catalyzed desulfinative cross‐coupling with (hetero)aryl bromides to deliver a broad range of biaryls. These latent sulfinate reagents have proven to be stable through multi‐step substrate elaboration, and amenable to scale‐up.

## Graphical abstract

Next‐generation, base‐activated latent sulfinate reagents have been successfully developed for use in the construction of heteroaromatic frameworks, such as 2‐arylpyridines. Under Pd‐catalyzed conditions, these species unmask to give the sulfinate in situ, which then undergoes efficient desulfinative cross‐coupling with an array of (hetero)aryl halides.

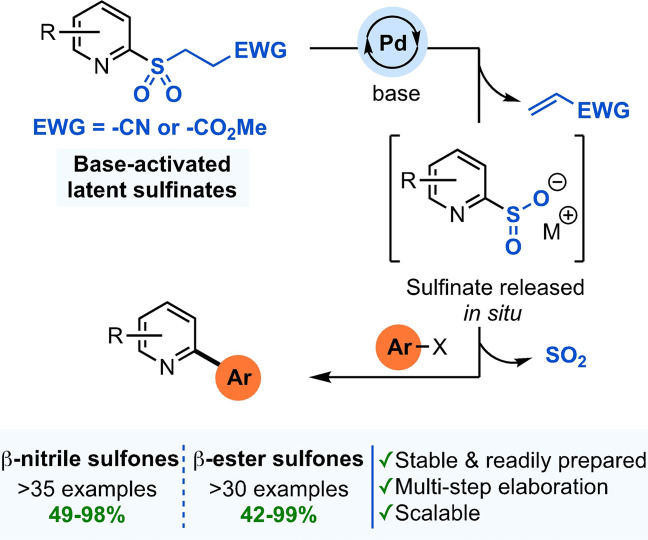

## Introduction

**Figure 1:**
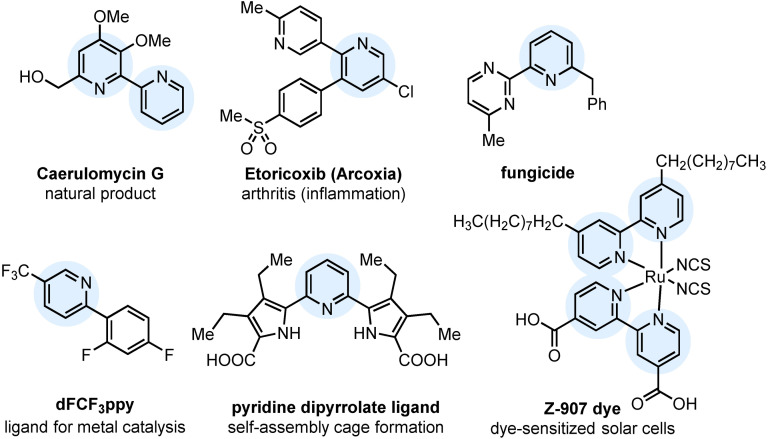
Select examples of 2‐pyridyl derivatives with varied applications.

Cross‐coupling processes are ubiquitous in synthetic chemistry as a dependable method to construct biaryl scaffolds, and are established as a “go to” strategy for carbon–carbon bond formation.^[1]^ The Suzuki–Miyaura cross‐coupling (SMC), which utilizes boron‐derived species as nucleophilic coupling partners, is the most popular variant for C(sp^2^)–C(sp^2^) bond formation.^[2]^ This is due to the commercial accessibility of boronic acids and boronate reagents, the mild reaction conditions, and the plethora of research conducted into these transformations.[^2a^, ^3^] However, traditional cross‐coupling tools, including the Suzuki–Miyaura approach, fall short when it comes to tackling notoriously difficult heteroaryl‐heteroaryl coupling reactions.^[4]^ Heteroaromatic boron reagents, in particular 2‐pyridyl variants, are especially challenging to prepare and couple due to their propensity to undergo protodeboronation.^[5]^ 2‐Arylpyridines, and bi(hetero)aryls in general, are prevalent as motifs in therapeutic and agrochemical molecules,^[6]^ as ligands in metal catalysis,^[7]^ and as key units in functional materials^[8]^ (Figure 1). Owing to the importance of these frameworks there has been significant research towards rectifying this problem,[^4^, ^9^] key examples of which include the development of slow release boronate reagents,^[10]^ and contractive coupling methodologies utilizing phosphorane[^9a^, ^9b^] or sulfurane^[9c]^ intermediates. Correspondingly, the “2‐pyridyl organometallic cross‐coupling problem”^[9d]^ has become a benchmark for challenging biaryl cross‐coupling reactions, and a springboard for innovation. However, a general approach is yet to be established, as many of the solutions reported are plagued with limited heterocycle scopes, difficult reagent preparation, or substrate‐specific re‐optimization.

**Figure 2:**
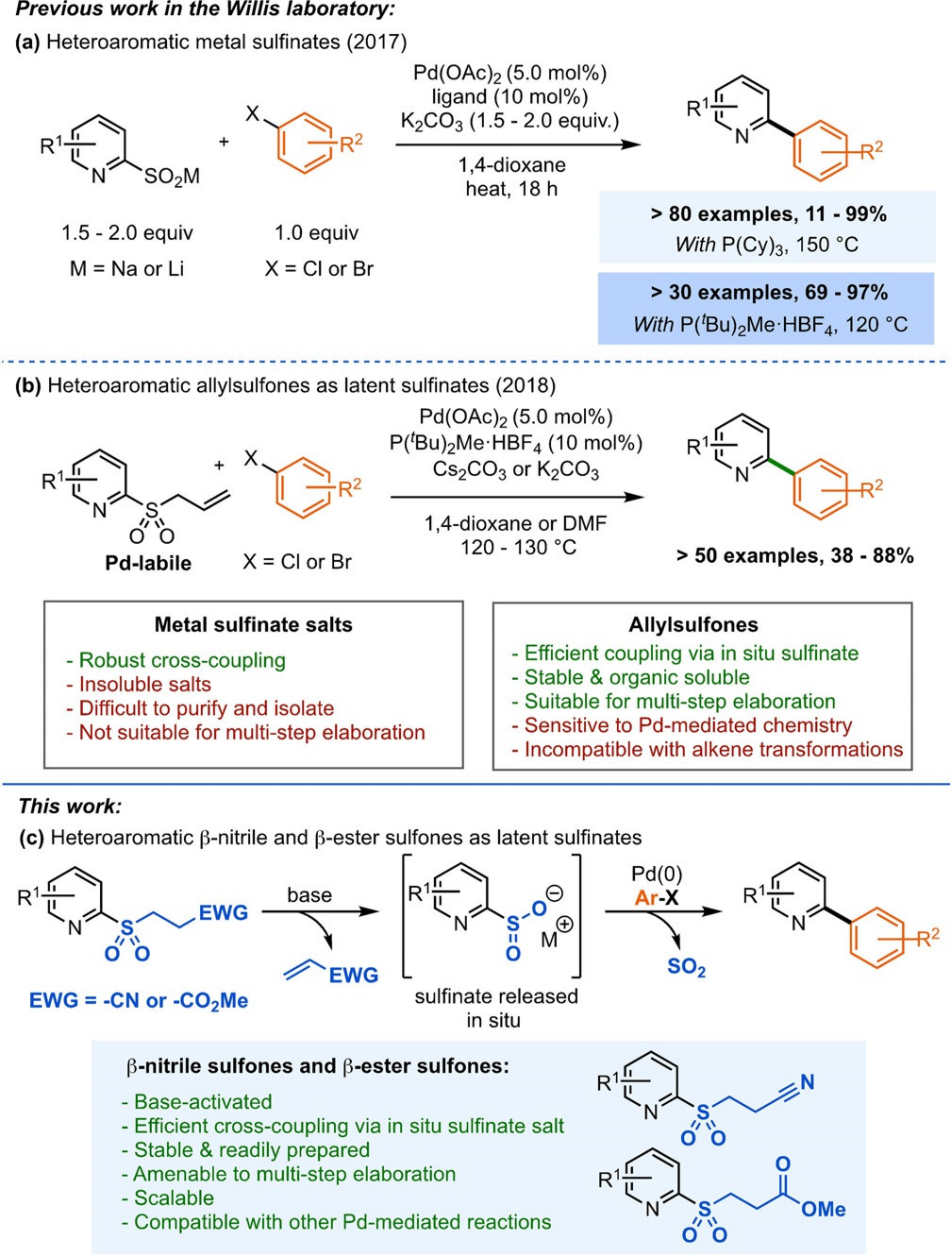
Introduction to previous and current work: a) heteroaromatic sulfinate salts, b) heteroaromatic allylsulfones and c) this work: β‐nitrile sulfones and β‐ester sulfones in cross‐coupling reactions.

Our laboratory has previously contributed to solving these challenges, establishing heteroaromatic sulfinate salts as efficient reagents in Pd‐catalyzed desulfinative cross‐coupling reactions with aryl halides (Figure 2 a).^[11]^ Desulfinative cross‐coupling processes avoid the use of stoichiometric organometallic reagents and only release SO_2_ as a by‐product. Although the use of aryl sulfinate salts in desulfinative cross‐couplings was first reported in the early 1990s,^[12]^ only in recent years has substantial research into their use as nucleophilic coupling partners in Pd‐mediated reactions been implemented.[^13^, ^14^] Importantly, heteroaromatic sulfinate salts are generally more air‐ and moisture‐stable than the corresponding boronic acids, and are now becoming commercially available.^[15]^

Despite being efficient coupling partners, the purification of metal sulfinate salts, as well as their poor solubility in organic media, pose challenges and hinder their ability to be carried through multi‐step synthetic sequences. To address this, our laboratory pioneered the use of latent sulfinates in cross‐coupling reactions. This concept exploits neutral, organosoluble, and easily accessible protected sulfinates that can be selectively unmasked under coupling conditions. The first generation of latent sulfinate reagents developed were allylsulfones (Figure 2 b).^[16]^ Owing to the lability of the allyl group under Pd‐catalysis, no additives were required for the release of the sulfinate coupling partner. However, this also limited the use of such substrates in other palladium‐catalyzed processes, and in alkene functionalization reactions.

This work explores a new generation of latent *N*‐heteroaromatic sulfinates: β‐nitrile and β‐ester sulfones (Figure 2 c).[^9f^, ^17^, ^21^] The active sulfinate species are released via E_1_cB elimination under the basic cross‐coupling conditions.^[18]^ These base‐labile reagents, in contrast to the Pd‐labile allylsulfones, are more amenable to orthogonal Pd‐catalyzed reactions and typical alkene transformations.

## Results and Discussion

**Scheme 1:**
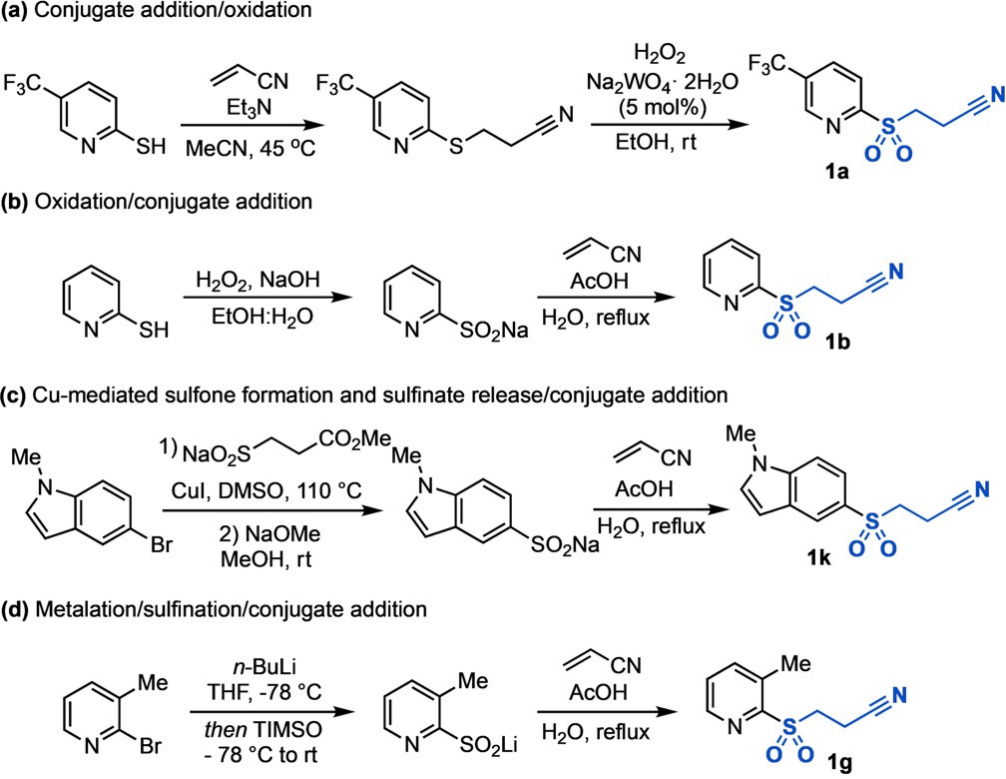
Synthesis of β‐nitrile sulfones. TIMSO=N‐Me‐pyrrolidine SO_2_ adduct.

Heteroaromatic sulfones can be readily accessed from commercially available starting materials, either directly from thiols, halides or via the sulfinate salt.[^19^, ^21^] The β‐nitrile sulfones were primarily accessed from thiols through conjugate addition into acrylonitrile and subsequent oxidation (the order of these steps could also be reversed, Scheme 1 a,b),[^11a^, ^11b^] from aryl halides using the sodium 3‐methoxy‐3‐oxopropane‐1‐sulfinate (SMOPS) reagent (Scheme 1 c),^[21]^ or by utilizing SO_2_ surrogates, such as DABSO, to form the parent sulfinate,[^19b^, ^19e^, ^20^] followed by conjugate addition (Scheme 1 d).

The use of β‐nitrile sulfones in Pd‐mediated cross‐coupling was initially investigated through the reaction of the pyridine sulfone **1 a** with 4‐bromoanisole **2 a** (Table 1). Encouragingly, the desired biaryl product **3 a** was obtained in a 29 % yield under the original conditions reported for the use of 2‐pyridyl metal sulfinate salts (Entry 1).^[11b]^ A strong solvent dependence was quickly observed, as substituting 1,4‐dioxane for toluene more than doubled the yield of biaryl **3 a** (Entry 2). The presence of K_2_CO_3_ proved to be essential to reaction success, in agreement with the reported mechanism of the transformation.[^11c^, ^11d^] A range of ligands were evaluated and electron‐rich, bulky monodentate phosphine ligands performed best (see Supporting Information). Whilst the optimum ligand for the allylsulfone work, P(^*t*^Bu)_2_Me⋅HBF_4_, showed good activity (Entry 3), cataCXium A (PAd_2_Bu) was shown to be superior for this process giving biaryl **3 a** in 84 % yield (Entry 4). Addition of acetic acid to the system was key for achieving high yields of the desired product at 130 °C (Entries 5–6). Other bases and acids were explored, however, none performed as well (see Supporting Information). Considering the poor solubility of sulfinate salts in toluene, the combination of base and acid could potentially create a buffered system, enabling a more gradual release of the sulfinate from the heteroaromatic sulfone. Lastly, by adjusting the stoichiometry of the pyridine sulfone to a slight excess (1.1 equiv), we were able to achieve an 88 % yield of biaryl **3 a** at 120 °C (Entry 8). The acrylonitrile by‐product was not observed and is assumed to be removed by evaporation.

**Table 1:** Selected optimization studies of the desulfinative coupling.^[a]^ 
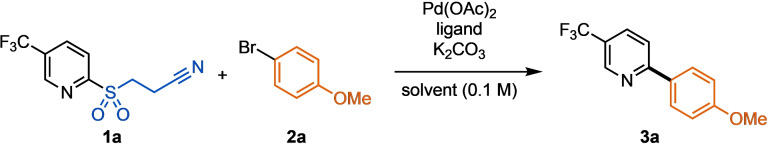

Entry	Solvent	Ligand	Additive (1.0 equiv)	*T* [°C]	Yield [%]^[b]^
1	1,4‐dioxane	PCy_3_	–	150	29^[c,d]^
2	toluene	PCy_3_	–	150	67
3	toluene	P(^*t*^Bu)_2_Me⋅HBF_4_	–	150	76
4	toluene	cataCXium A	–	150	84
5	toluene	cataCXium A	–	130	59
6	toluene	cataCXium A	AcOH	130	84
7^[e]^	toluene	cataCXium A	AcOH	130	91^[d]^
8^[e]^	toluene	cataCXium A	AcOH	120	88^[d]^

[a] Reaction conditions: **1 a** (0.20 mmol, 1.0 equiv), 4‐bromoanisole (0.20 mmol, 1.0 equiv), K_2_CO_3_ (0.30 mmol, 1.5 equiv), Pd(OAc)_2_ (5.0 mol %), ligand (10 mol %), solvent (0.10 M). [b] HPLC yields determined by using *p*‐tolylether as an internal standard. [c] Run on a 0.40 mmol scale. [d] Isolated yield. [e] Using 1.1 equiv of **1 a**. cataCXium A=PAd_2_Bu.

We began the reaction scope exploration by varying the electrophilic partner in combination with sulfone **1 a** (Table 2 a). Aryl bromides were found to couple more efficiently than aryl chlorides, as shown by the 61 % yield of compound **3 a** obtained from 4‐chloroanisole, compared to 88 % using 4‐bromoanisole. This difference in reactivity could be advantageously exploited and chlorobiaryl **3 b** was obtained in a high 80 % yield. The reaction yield remained high when varying the position of the methyl substituent on the aryl bromide (**3 c**–**3 f**), although a slight increase in temperature to 130 °C was needed for some *ortho*‐substituted aryl bromides (**3 e**, **3 f**). Under these reaction conditions, pyridine sulfone **1 a** provides an efficient synthesis of the pyridine **3 h**, used widely as a ligand in photocatalyst complexes and luminescent platinum complexes.[^7a^, ^7b^] The reaction has a broad functional group tolerance, delivering high yields when employing aryl halides containing esters, ketones, nitriles, protected amines and alcohols, thiophenes, substituted pyridines, quinolines, quinoxalines and napthalenes (**3 i**–**3 s**).

**Table 2:**
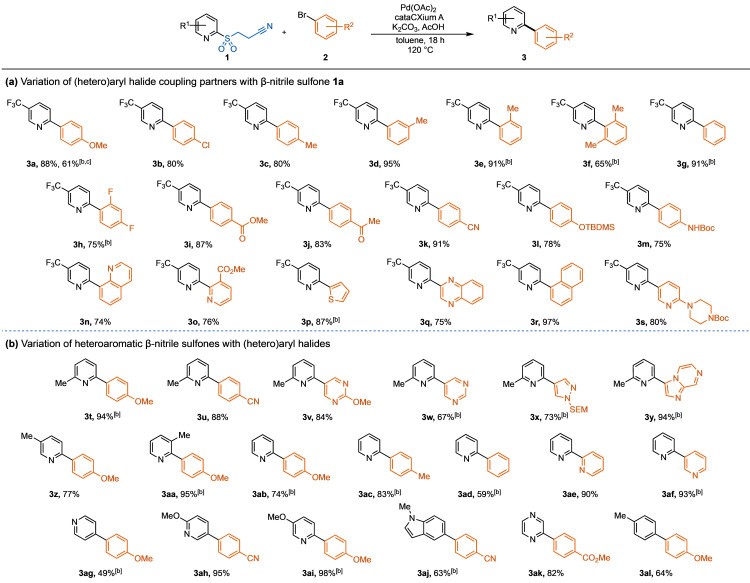
Scope of the desulfinative cross‐coupling of heteroaromatic β‐nitrile sulfones with (hetero)aryl halides.^[a]^

[a] Isolated yields. Reaction conditions: pyridine sulfone (0.22 mmol, 1.1 equiv), aryl halide (0.20 mmol, 1.0 equiv), K_2_CO_3_ (0.30 mmol, 1.5 equiv), AcOH (0.20 mmol, 1.0 equiv), Pd(OAc)_2_ (5.0 mol %), cataCXium A (10 mol %), toluene (0.10 M), 120 °C, 18 h. [b] Reaction run at 130 °C. [c] Using 4‐chloroanisole. Temperatures stated are those of the aluminium heating block.

On variation of the nucleophilic coupling partner (Table 2 b), we found that the 6‐ and 5‐methyl substituted pyridine sulfones behave similarly to the electron‐deficient 5‐trifluoromethyl pyridine sulfone **1 a** (**3 t**, **3 u, 3 z**). Pleasingly, the more challenging couplings to access biaryls, such as pyrimidines (**3 v**–**3 w**), SEM‐protected pyrazole (**3 x**) and imidazo[1,2‐*a*]pyrazine (**3 y**), were achieved in good to high yields. Increasing the steric hindrance at the reaction site via substitution at the 3‐position of the pyridine, was also well tolerated, giving 95 % of 2‐arylpyridine **3 aa**. Increasing the reaction temperature to 130 °C was often necessary to achieve good yields when using unsubstituted pyridine sulfones (**3 ab**–**3 ag**). This is in good agreement with our previous mechanistic studies, which show that extrusion of SO_2_ during the catalytic cycle is easier with electron‐deficient or sterically bulky substrates.^[11c]^ Electron‐rich pyridine sulfones also performed well under these reaction conditions (**3 ah**, **3 ai**). Notably, indole **3 aj** and pyrazine **3 ak** were synthesized in good to high yields. This methodology can also be extended to carbocyclic substrates, as shown with compound **3 al**, obtained in 64 % from *p*‐tolyl β‐nitrile sulfone.

To demonstrate the generality and robustness of base‐labile latent sulfinates, we extended our approach to explore the use of β‐ester sulfones. In comparison to β‐nitrile sulfones, the synthetic routes towards β‐ester substrates are more straightforward, mainly due to β‐methylester sulfones being established intermediates in the synthesis of metal sulfinates.[^9f^, ^19a^, ^21^] The aforementioned SMOPS reagent is commercially available and provides a one‐step route to the β‐methylester sulfone functionality.^[21]^ Additionally, commercially available methyl 3‐mercaptopropionate can also be reacted directly with heteroaromatic halides, or facilitated by metal catalysis if required,^[22]^ which in turn can be followed by S‐oxidation to give β‐methylester sulfones. Thus, synthetic routes towards β‐ester sulfones allow better use of the vast libraries of readily accessible heteroaryl halides.

In contrast to the nitrile derivatives, β‐ester sulfones generally performed better when the AcOH additive was omitted from the system. Under such conditions, the reactivity and functional group tolerance of these alternative latent sulfinates were found to be comparable to the β‐nitrile sulfones, giving the desired biaryls in high yields (Table 3, **3 a**, **3 b**, **3 i**, **3 k**, **3 q**). The free alcohol product **3 l‐OH** was pleasingly obtained in a 65 % yield from the telescoped deprotection of protected alcohol **3 l**. Similar to the β‐nitrile sulfone system, raising the reaction temperature to 130 °C was required for efficient coupling of the unsubstituted pyridyl β‐ester sulfone (**3 ac**, **3 ae**, **3 af**, **3 an**). Interestingly, the coupling of the unsubstituted pyridine sulfone to give the biaryl **3 ac**, was poor yielding under the optimized conditions, despite complete consumption of the sulfone. However, addition of acetic acid (1.5 equiv) into the reaction allowed for the dramatic increase in the yield of product **3 ac** from 29 % to 91 %. The 4‐pyridyl β‐ester sulfone worked comparably to the 2‐pyridyl reagent, giving 4‐arylpyridines **3 ag** and **3 ao**.

**Table 3:**
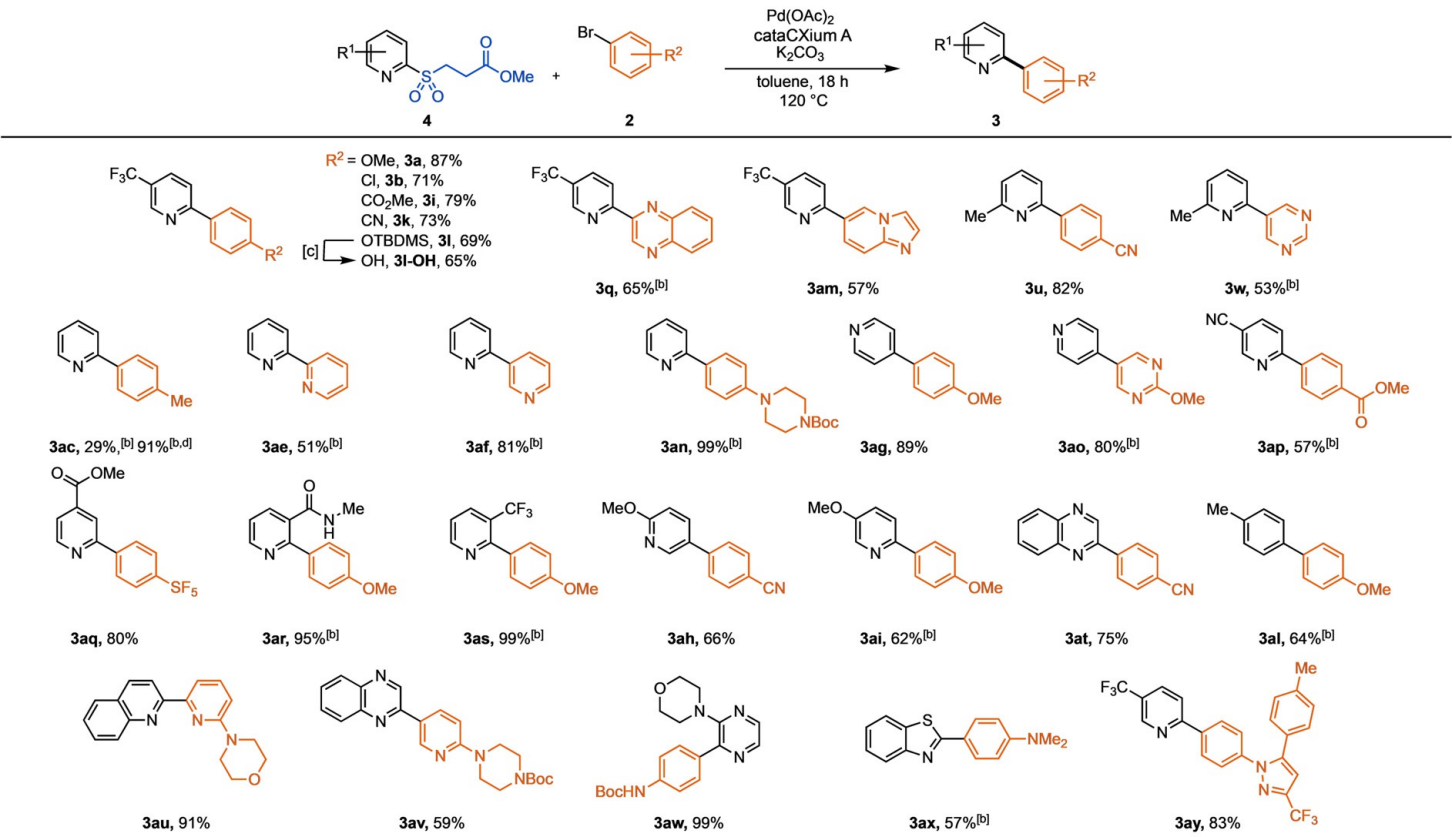
Scope of the desulfinative cross‐coupling of heteroaromatic β‐ester sulfones with (hetero)aryl halides.^[a]^

[a] Isolated yields. Reaction conditions: sulfone (0.3 mmol, 1.5 equiv), aryl bromide (0.2 mmol, 1.0 equiv), Pd(OAc)_2_ (5.0 mol %), cataCXium A (10 mol %), K_2_CO_3_ (0.4 mmol, 2.0 equiv), toluene (0.10 M), 120 °C, 18 h. [b] Run at 130 °C. [c] Telescoped deprotection of protected phenol product directly after cross‐coupling reaction: reaction temperature lowered to 85 °C, ethanol (2 mL), 2 M aq. solution of K_2_CO_3_ (0.2 mL, 0.4 mmol), 18 h. [d] Reaction run with AcOH (0.3 mmol, 1.5 equiv). Temperatures stated are those of the aluminium heating block.

Nucleophilic coupling partners with electron‐withdrawing substituents on the pyridine core, such as a nitrile (**3 ap**) or an ester (**3 aq**), as well as quinoxaline sulfones (**3 at**), performed well, as did electron‐rich β‐ester examples (**3 ah**, **3 ai**). Similar to the β‐nitrile sulfone system, substituents next to the sulfinate coupling site were well tolerated (**3 ar**–**3 as**). More complex, nitrogen‐rich frameworks **3 au** and **3 av**, were constructed from the corresponding 2‐quinoline and 2‐quinoxaline sulfones. The pyrimidine containing molecule **3 aw**, an intermediate of a class of RORγ modulators,^[23]^ was synthesized in a 99 % yield. Pleasingly, the challenging coupling of benzothiazole β‐methyl ester sulfone to give benzothiazole **3 ax**, a product related to PET scanning radiopharmaceutical Flutemetamol (^18^F), was achieved.^[24]^ Lastly, we were able to prepare complex biaryl **3 ay**, containing a fragment of arthritis drug celecoxib, in 83 % yield.^[25]^

The success of the latent sulfinates (Table 2 and Table 3) inspired the design and brief exploration of diethyl succinate sulfones **5**. Upon generation of the sulfinate salt, this alternative masking group releases non‐toxic, high‐boiling diethyl fumarate as the by‐product, making this system more amenable to cross‐coupling on larger scale. Additionally, the majority of these reagents can be readily isolated as solids. Encouragingly, the succinate pyridine sulfone **5** performed well under the same conditions employed for the β‐methyl ester sulfone (Table 4). These reagents react efficiently and comparably to their β‐nitrile and β‐ester counterparts, as exemplified through a representative set of biaryls (Table 4).

**Table 4:**
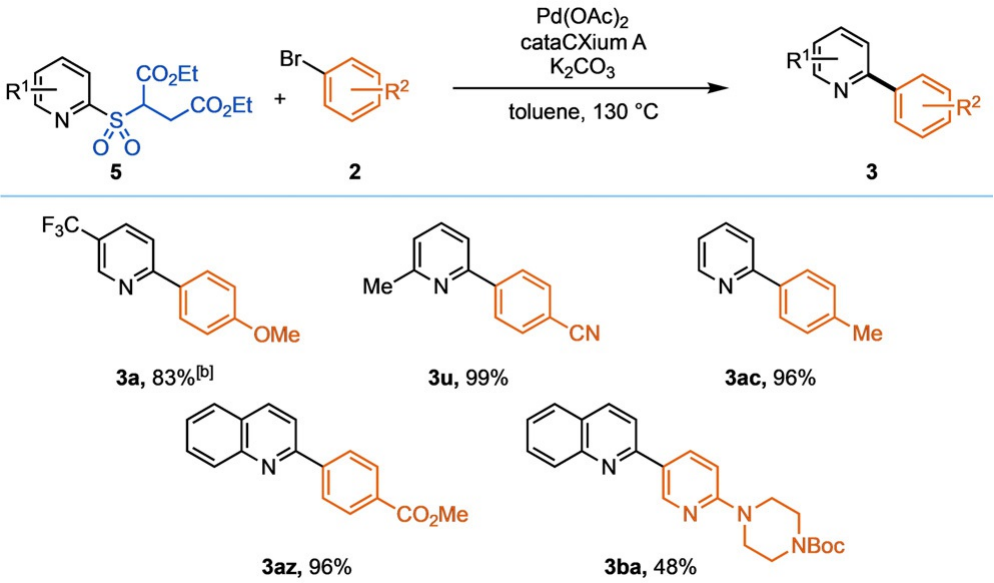
Scope of the desulfinative cross‐coupling of succinate sulfones with aryl bromides.^[a]^

[a] Isolated yields. Reaction conditions: pyridine succinate sulfone (0.3 mmol, 1.5 equiv), aryl bromide (0.2 mmol, 1.0 equiv), Pd(OAc)_2_ (5.0 mol %), cataCXium A (10 mol %), K_2_CO_3_ (0.4 mmol, 2.0 equiv), toluene (0.1 M), 130 °C, 18 h. [b] Reaction run at 120 °C.

**Scheme 2:**
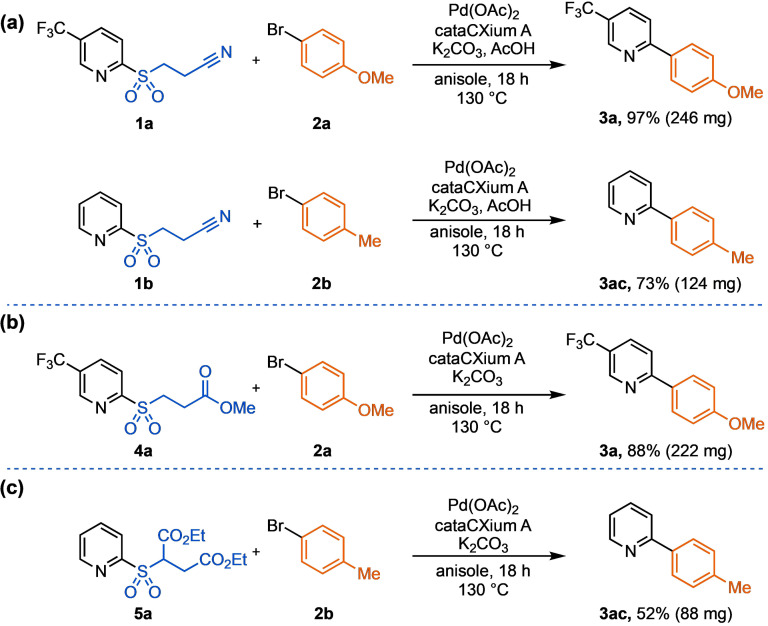
Scale up of the cross‐coupling reactions of base‐labile latent sulfinate reagents. Reaction conditions: a) pyridine sulfone (1.5 mmol, 1.5 equiv), 4‐bromoanisole (1.0 mmol, 1.0 equiv), Pd(OAc)_2_ (5.0 mol %), cataCXium A (10 mol %), K_2_CO_3_ (2.0 mmol, 2.0 equiv), AcOH (1.5 mmol, 1.5 equiv), anisole (0.1 M), 130 °C, 18 h. b and c) pyridine sulfone (1.5 mmol, 1.5 equiv), 4‐bromoanisole (1.0 mmol, 1.0 equiv), Pd(OAc)_2_ (5.0 mol %), cataCXium A (10 mol %), K_2_CO_3_ (2.0 mmol, 2.0 equiv), anisole (0.1 M), 130 °C, 18 h. Isolated yields; internal temperatures measured.

The described reactions of our masked sulfinates were routinely performed on 0.2 mmol scale in sealed reaction vials. However, we were keen to show that the chemistry could be translated to larger, non‐pressurized reaction vessels. To investigate this, the cross‐coupling of sulfones **1 a** or **4 a** with 4‐bromoanisole **2 a** to give biaryl **3 a** was re‐visited. The key substitution of toluene for higher boiling anisole, and a reaction temperature of 130 °C allowed the reactions of both masked sulfinates species to be run effectively on a 1.0 mmol scale (Scheme 2). Pleasingly, an unoptimized 52 % yield of 2‐(*p*‐tolyl)pyridine (**3 ac**) was also obtained using the 2‐pyridyl succinate sulfone **5 a**.

**Scheme 3:**
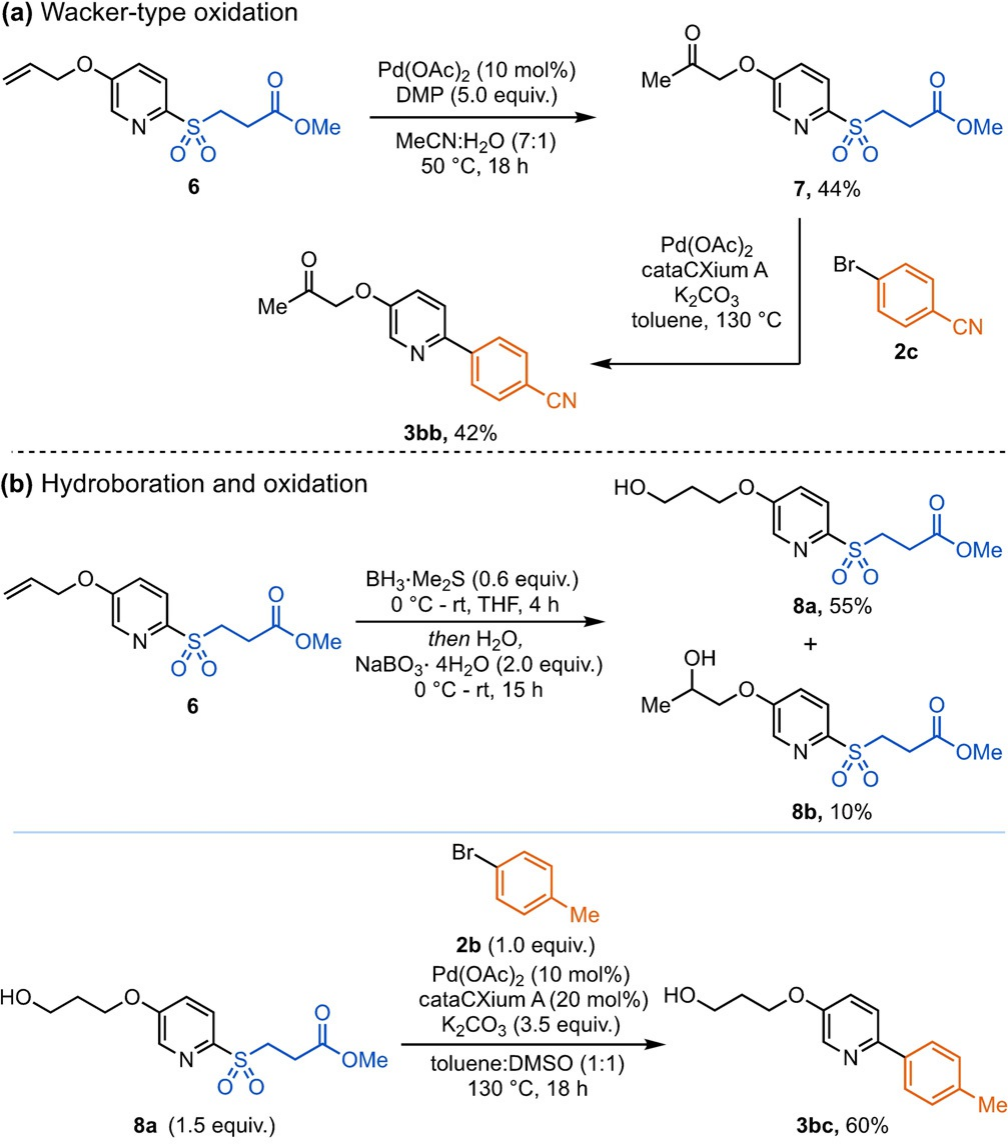
Oxidative transformations of O‐allyl pyridine sulfone **6**. DMP=Dess–Martin periodinane.

To highlight the compatibility of β‐nitrile and β‐methyl ester sulfone with multi‐step substrate functionalization, we investigated the stability and utility of these masking groups through a series of transformations around the pyridine core, primarily focusing on oxidative and palladium‐mediated reactions. Firstly, using O‐allyl pyridine sulfone **6**, we explored oxidative transformations at the alkene functionality (Scheme 3). Pyridine **6** was oxidized under Wacker‐type conditions^[26]^ to give the desired ketone product **7** in a 44 % yield, and the resultant ketone was further reacted to access biaryl **3 bb**. Furthermore, through a hydroboration‐oxidation sequence we could achieve a 65 % yield of the combined alcohol products (**8 a**,**b**); this is a transformation that would not be possible using the corresponding allylsulfone. The primary alcohol **8 a** was then successfully coupled to give 60 % of the desired 2‐arylpyridine **3 bc**.

**Scheme 4:**
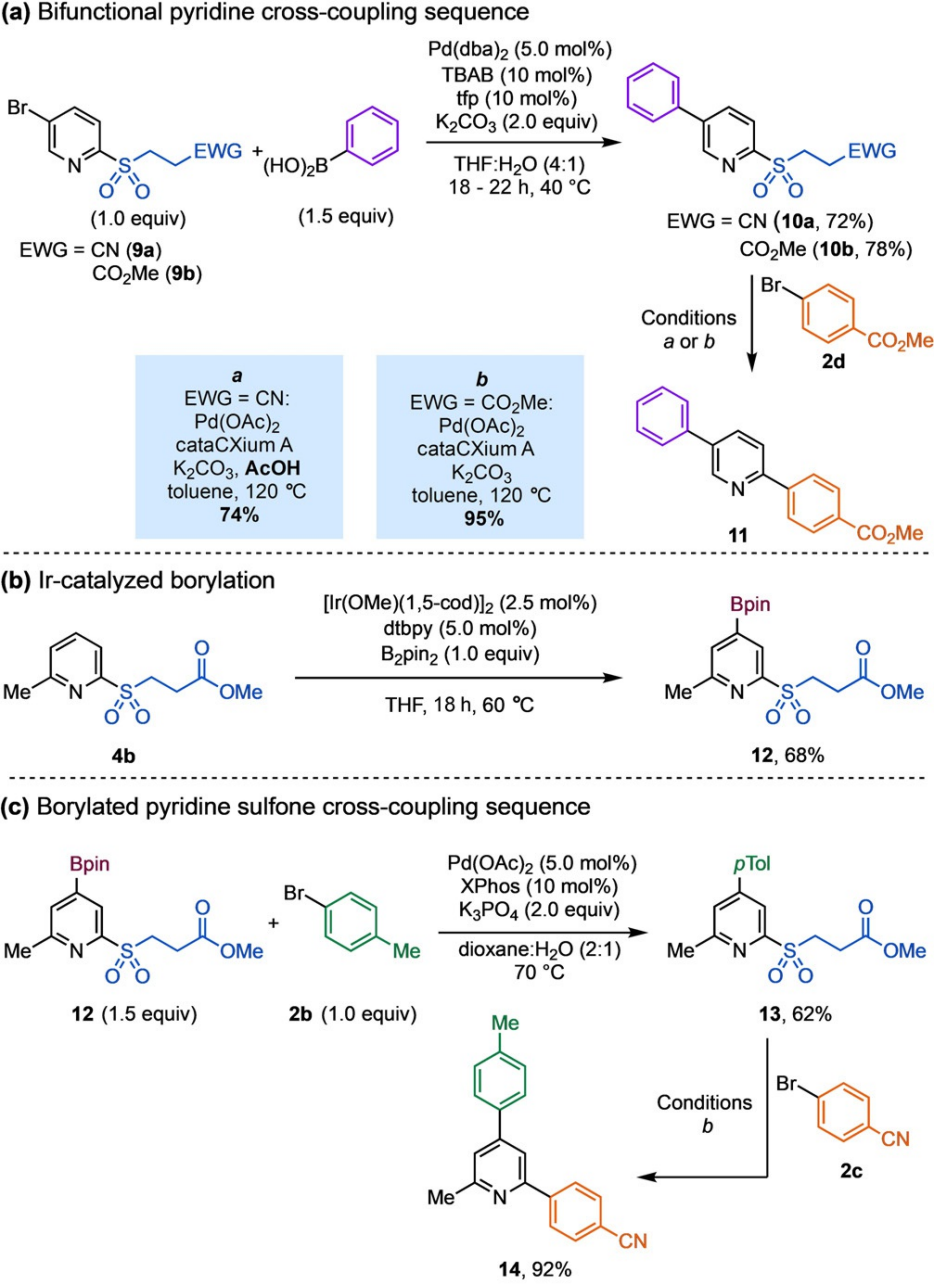
Transition‐metal mediated, multi‐step cross‐coupling sequences of 2‐pyridyl latent sulfinate reagents. tfp=tri(2‐furyl)phosphine. dtbpy=4,4′‐di‐*tert*‐butyl‐2,2′‐dipyridyl. XPhos=2‐dicyclohexylphosphino‐2′,4′,6′‐triisopropylbiphenyl.

Lastly, we explored the bidirectional elaboration of masked pyridine reagents by installing two cross‐coupling reaction sites that could be selectively transformed (Scheme 4). 5‐Bromopyridine sulfones (**9 a**,**b**) successfully underwent Suzuki–Miyaura cross‐coupling (SMC) with phenyl boronic acid to give the biaryl sulfones (**10 a**,**b**) in 72 % and 78 % yields, when using β‐nitrile or β‐ester masking groups, respectively.^[27]^ In both cases the latent sulfinate group was stable under these reaction conditions. Sequentially these products were submitted to desulfinative coupling conditions to give the desired diarylpyridine compound **11** in high yields. To further demonstrate the compatibility of this chemistry with transition metal‐mediated processes, the boronate ester containing substrate **12** was prepared using an Ir‐catalyzed borylation reaction (Scheme 4 b).^[28]^ The bifunctional pyridine **12** underwent SMC with 4‐bromotoluene **2 b** to give sulfone **13** in 62 % yield;^[29]^ notably, the masked sulfinate functionality remained intact. Finally, diarylpyridine **14** was obtained in excellent 92 % yield from biaryl sulfone **13** using our standard cross‐coupling protocol.

## Conclusion

We have designed and developed β‐nitrile and β‐ester sulfones as efficient base‐labile latent sulfinate reagents in palladium‐catalyzed cross‐coupling reactions. This method has been used to construct challenging heteroaryl‐(hetero)aryl linkages, allowing access to a diverse range of 2‐arylpyridines and pharmaceutically relevant fragments. The scope of electrophilic partners is broad, displaying a good tolerance of multiple functional groups, and substitution patterns, delivering the desired cross‐coupled products in good to high yields. Furthermore, both latent sulfinate groups are stable and can be carried through multistep elaboration, notably oxidative and palladium‐mediated transformations. We have further shown the successful use of the succinate sulfone masking group, which releases a benign by‐product on activation. Owing to the prevalence of the pyridine motif in medicinal chemistry and the robustness of this chemistry, we anticipate this method will find wide application.

## Conflict of interest

The authors declare no conflict of interest.

## Supporting information

As a service to our authors and readers, this journal provides supporting information supplied by the authors. Such materials are peer reviewed and may be re‐organized for online delivery, but are not copy‐edited or typeset. Technical support issues arising from supporting information (other than missing files) should be addressed to the authors.

Supporting Information
