# Supplementary material for: Base‐Activated Latent Heteroaromatic Sulfinates as Nucleophilic Coupling Partners in Palladium‐Catalyzed Cross‐Coupling Reactions
Source: Angew Chem Int Ed Engl. 2021 Sep 8;60(41):22461–8. doi: 10.1002/anie.202109146 (PMC8518705; doi:10.1002/anie.202109146)
Supplement: Supplementary file 1 — Supporting Information [file ANIE-60-22461-s001.pdf]

Angewandte Chemie
Eine Zeitschrift der Gesellschaft Deutscher Chemiker

Supporting Information

Base-Activated Latent Heteroaromatic Sulfinates as Nucleophilic Coupling Partners in Palladium-Catalyzed Cross-Coupling Reactions

*Xinlan A. F. Cook, Loïc R. E. Pantaine, David C. Blakemore, Ian B. Moses, Neal W. Sach, Andre Shavnya, and Michael C. Willis**

anie_202109146_sm_miscellaneous_information.pdf

Contents

1. General considerations S2

2. Optimisation Screening Tables S3

2.1 Optimisation of the β-nitrile sulfone system S3

2.2 Elucidation the role of acetic acid S7

2.3 Monitoring the consumption of the sulfone starting material and formation of biaryl product... S8

2.4 Optimisation of the β-methylester sulfone system S10

2.5 Optimisation of both the β-nitrile sulfone and β-methylester sulfone systems outside of a pressurised system S12

3. Synthetic Procedures and characterisation data S13

3.1 HPLC information S13

3.2 Synthesis of starting materials and Intermediates S14

3.2.a Synthesis of heteroaromatic thioethers S14

3.2.b Synthesis of heteroaromatic sulfones (latent sulfinates) S28

3.2.c Synthesis of sulfinates S54

3.3 Heteroaromatic desulfinative cross-coupling S55

3.4 Substrate limitations of the desulfinative cross-coupling reaction S99

3.5 Scaled up desulfinative cross-coupling: Outside of a sealed reaction vessel S100

3.6 Derivatisations of the pyridine core of the masked sulfinate reagents S102

3.7 Synthesis of miscellaneous building blocks S107

4. NMR Spectra S109

4.1 NMR Spectra of heteroaromatic sulfide intermediates S109

4.2 NMR Spectra of heteroaromatic sulfones – *Base-labile sulfinates* S136

4.3 NMR Spectra of metal sulfinates S174

4.4 NMR Spectra of cross-coupled products S177

4.5 NMR Spectra of derivatisation compounds S252

5. References S259

1. General considerations

All reactions were performed under an inert nitrogen atmosphere with anhydrous solvent and constant magnetic stirring, unless otherwise stated. All reaction were run using clean glassware that was oven dried at >100 °C for at least 2 hours, and allowed to cool to room temperature (RT) under a positive pressure of nitrogen. All inert gases were sourced from the University of Oxford's internal supplies and dried through $CaCl_2$ drying columns. Reactions were monitored by thin-layer chromatography (TLC) until deemed to be complete. TLC was performed on Merck Kieselgel 60 PF254 pre-coated aluminium backed TLC sheets and visualized by UV lamp (λ = 254 nm) and/ or TLC staining by dipping in $KMnO_4$, Dragendorff or Vanillin solutions

All reagents used were obtained from commercial sources and used without further purification, unless otherwise stated; commercial sources include Sigma Aldrich Chemical Co. Ltd., Fluorochem Ltd., Alfa Aesar, Insight Biotechnology Ltd., Acros Organics Ltd. and Strem Chemicals Inc. Phosphine ligands and salts (K_2CO_3, Na_2CO_3, Cs_2CO_3) were all stored under inert atmosphere and inside a desiccator. Salts were dried by heating at 180 °C with stirring under high vacuum (< 2 mbar) for 18 h. Dry solvents were obtained from the University of Oxford internal solvent drying system (Innovative Technology Inc. PS-400-7). Solvents used in the desulfinative cross-coupling were degassed thoroughly by vigorously bubbling a stream of N_2 through the solvent for a minimum of 15 mins prior to use. Toluene was stored under nitrogen, over activated 3 Å molecular sieves. Reagent grade solvents were used for purification. Use of 'Petrol' as a solvent, refers to the fraction of petroleum ether which boils within the range 40–60 °C. Flash column chromatography was performed by adsorbing the crude mixture onto celite and dry loading onto a slurried column. Flash columns used fluorochem silica gel (60 A 40-63 u) and the indicated eluent system.

^{1}H, ^{19}F and ^{13}C NMR spectra were recorded on a Bruker AVIII400 Ultrashield spectrometer in deuterated solvent at the stated operating frequency. ^{13}C NMR spectra were recorded with ^{1}H broadband decoupling. Chemical shifts (δ) are reported in parts per million (ppm) and referenced relative to the residual solvent peak. ^{19}F NMR was referenced to $CFCl_3$. Chemical shifts from ^{1}H NMR and ^{19}F NMR spectrometry are recorded to two decimal places, whereas chemical shifts from ^{13}C NMR spectrometry are recorded to 1 decimal place. Multiplicities of the resonances are given as singlet (s), doublet (d), triplet (t), quartet (q), quintet (qn), multiplet (m), broad (br.), apparent (app.), doublet of doublet (dd), etc. Coupling constants (*J*) are given in Hertz (Hz) and rounded to the nearest 0.5 Hz. Spectra assignments were deduced using chemical shifts, coupling constants, 2D-NMR spectra (COSY and HSQC) and/or by comparison to spectra of related compounds.

Low-resolution mass spectra were recorded on an Agilent 6120 Quadruple spectrometer (ESI) using methanol: water: FA (90:10: 0.1). High resolution mass spectra were obtained *via* the in-house service at the Chemistry Research Laboratory, University of Oxford, utilizing a Bruker Daltronics MicroTOF spectrometer. Mass spectra samples were prepared as 1 mg/mL solutions in MeOH/MeCN. The *m/z* values are all recorded in Daltons to one decimal place for the low resolution spectrum and to four decimal places on the high resolution. For high resolution mass spectra, the mass found was compared to the mass calculated from the monoisotopic molecular formula, and all results were found to be within a tolerance of 5 ppm of the calculated values. Infrared spectra were determined neat using a Bruker Tensor 27 FT spectrometer with an internal range of 600-4000 cm^{-1} and all absorptions are given in wavenumbers to the nearest whole number (cm^{-1}). Compound names are generated by PerkinElmer ChemDraw Professional.

2. Optimisation Screening Tables

2.1 Optimisation of the β-nitrile sulfone system

Table 1. Preliminary solvent & base screen.

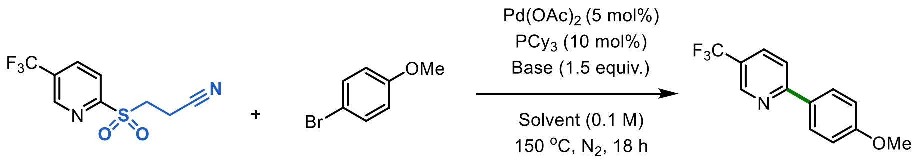

1a **2a** **3a**

Entry	Sulfone (equiv.)	Ar-Br (equiv.)	Solvent	Base	Isolated Yield / %
**1**	2.0	1.0	1,4-dioxane	K_2CO_3	20
**2**	2.0	1.0	1,4-dioxane	Cs_2CO_3	13
**3**	1.0	1.0	1,4-dioxane	K_2CO_3	29[a]
**4**	1.0	1.5	1,4-dioxane	K_2CO_3	28
**5**	2.0	1.0	1,4-dioxane	K_2CO_3 (3.0 equiv.)	17
**6**	1.0	2.0	1,4-dioxane	K_2CO_3	19
**7**	1.0	1.0	1,4-dioxane	^{t}BuOK (1.0 equiv.)	<7
**8**	1.0	1.5	Toluene	K_2CO_3	64
**9**	1.0	1.5	Toluene	Cs_2CO_3	45
**10**	1.0	1.0	Toluene	Na_2CO_3	13

Reaction conditions: Run on a 0.20 mmol scale, pyridine sulfone (specified abov), 4-bromoanisole (specified above), K_2CO_3 (0.30 mmol, 1.5 equiv.), $Pd(OAc)_2$ (0.01 mmol, 5.0 mol%), PCy_3 (0.02 mmol, 10 mol%) and solvent (0.1 M). [a] Run on a 0.40 mmol scale.

Table 2. Screen of Phosphine Ligands and Selected Palladium sources.

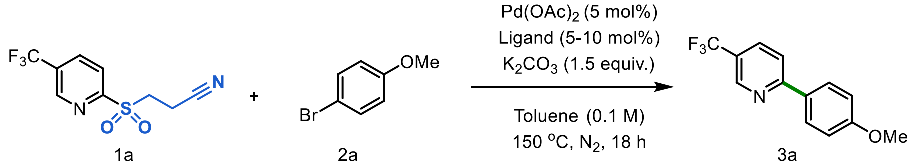

1a 2a 3a

Entry	Ligand	Yield[a] / %
1	CataCXium A	84 / 59[b]
2	$P(^iPr)_3$	80 / 42[b]
3	$P(^tBu)_2MeHBF_4$	76/ 48[b]
4	$P(^tBu)_2MeHBF_4$(6 mol%)	75
5	PCy_3	67
6	PPh_3	56
7	$P(Adm)_3$	12
8	Cy-JohnPhos	13
9	$PCy(^tBu)_2$	3
11	$P(^tBu)_3HBF_4$	3
12	$P(^nBu\ )_3$	0
13	$P(OPh)_3$	0
14	P(2-furyl)$_3$	0
15	$P(Adm)_2Bn$	4[b]
16	$P(Cy)_2Et$	1[b]
17	GorlosPhos HBF_4	34[c]
18	dppe	57
19	dcpe	39
20	dcpb	20
21	dppp	16
22	XantPhos	15
23	dppf	8
24	dcpp	5[b]
25	(PCy_3)Pd-G3	24
26	CataCXium A Pd-G3	34[b]
27	$PdCl_2$ (5.0 mol%)	86[d,b]
28	$Pd(TFA)_2$ (5.0 mol%)	100[d,b]
29	No Palladium	0[e]
30	No Ligand	0

Reaction conditions: pyridine sulfone (0.20 mmol, 1.0 equiv.), 4-bromoanisole (0.20 mmol, 1.0 equiv.), K_2CO_3 (0.30 mmol, 1.5 equiv.), $Pd(OAc)_2$ (0.01 mmol, 5.0 mol%), mono-dentate ligand (0.02 mmol, 10 mol%) or bi-dentate ligand (0.01 mmol, 5.0 mol%) and toluene (0.1 M). [a]HPLC yield determined using *p*-ditolylether as an internal standard. [b] Run at 130 °C, [c]Run at 120 °C, [d] Run with no $Pd(OAc)_2$, CataCXium A (10 mol%) as the ligand and AcOH (0.2 mmol, 1.0 equiv.), [e] Using CataCXium A (10 mol%).

Strutures of Phosphine ligands

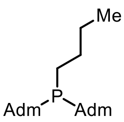

CataCXium A

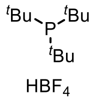

P(^{t}Bu)$_3$•HBF$_4$

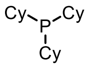

PCy$_3$

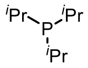

P(^{i}Pr)$_3$

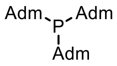

PAdm$_3$

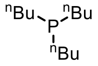

P^{n}Bu$_3$

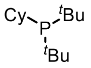

P(^{t}Bu)$_2$Cy

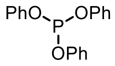

P(OPh)$_3$

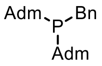

PAdm$_2$Bn

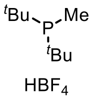

P(^{t}Bu)$_2$Me•HBF$_4$

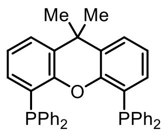

Xantphos

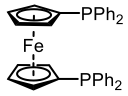

dppf

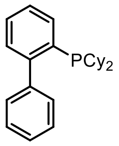

CyJohnPhos

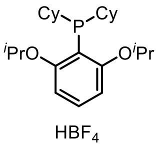

Gorlosphos•HBF$_4$

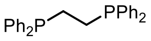

dppe

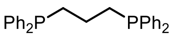

dppp

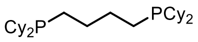

dcpb

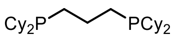

dcpp

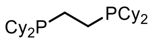

dcpe

Pd-G3 refers to the third generation of the Buchwald palladium precatalysts.with the structure below:

G3:

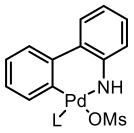

L = CataCXium A or P(Cy)$_3$

Table 3. Screen of additives to the cross-coupling system.

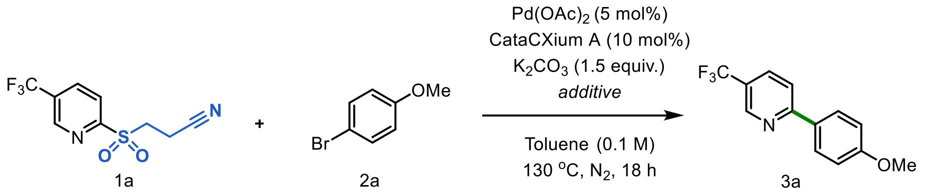

Entry	Additive	Variations to above system	Yield[a] / %
**1**	-	-	59
**2**	5.0 mol% Et_3N	-	69
**3**	10 mol% Et_3N	-	72
**4**	20 mol% Et_3N	-	74
**5**	Et_3N (1.0 equiv.)	-	73
**6**	Et_3N (1.5 equiv.) (*no K_2CO_3)*	-	9
**7**	AcOH (0.1 equiv.)	-	70
**8**	AcOH (0.2 equiv.)	-	79
**9**	AcOH (0.5 equiv.)	-	86
**10**	AcOH (1.0 equiv.)	-	93 (84)
**11**	AcOH (1.5 equiv.)	-	79
**12**	AcOH (1.0 equiv.)	Run in toluene (0.05 M)	78
**13**	AcOH (1.0 equiv.)	Run in toluene (0.20 M)	58
**14**	AcOH (1.0 equiv.) and 10 mol% Et_3N	-	70
**15**	10 mol % acetic anhydride	-	81
**16**	50 mol % acetic anhydride	-	85
**17**	Additional 10 mol% of **1a**	-	81
**18**	Additional 10 mol% of **1a** + AcOH (1.0 equiv.)	-	94
**19**	Additional 10 mol% of **1a** + AcOH (1.0 equiv.)	-	10[b]
**20**	Additional 10 mol% of **1a** + AcOH (1.0 equiv.)	-	96[c] (88)
**21**	Additional 10 mol% of **1a** + AcOH (1.0 equiv.)	Run in anisole (0.10 M)	94[c]
**22**	Additional 10 mol% of **1a** + AcOH (1.0 equiv.)	Run in *p*-xylene (0.10 M)	(77)[c]
**23**	Additional 10 mol% of **1a** + AcOH (1.0 equiv.)	-	86[c, d]

Reaction conditions: pyridine sulfone (0.20 mmol, 1.0 equiv.), 4-bromoanisole (0.20 mmol, 1.0 equiv.), K_2CO_3 (0.30 mmol, 1.5 equiv.), $Pd(OAc)_2$ (0.01 mmol, 5.0 mol%), ligand (0.02 mmol, 10 mol%) and toluene (0.1 M). [a]HPLC yield determined using *p*-ditolylether as an internal standard. [b]Run at 100 °C, [c]Run at 120 °C, [d]Water (100 µL) added to the reaction. Isolated yields in brackets.

After screening additives (Table 3), we found that the addition of AcOH (1.0 equiv.) and a slight excess of pyridine sulfone (1.1 equiv.) enabled us to achieve a high yield of biaryl **2a** at both 130 °C and 120 °C (Entries 18 and 20). However we saw a large reduction in yield when the temperature was lowered to 110 °C (Entry 19).

2.2 Elucidation the role of acetic acid

Table 4. Assessment of acid and various salt additives.

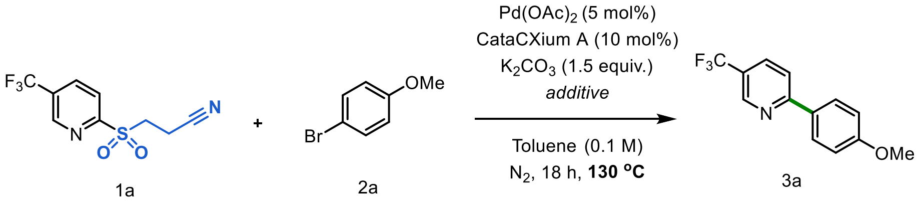

Entry	Additive	Yield[a] / %
1	AcOH (1.0 equiv.)	94
2	Pivalic Acid (1.0 equiv.)	42
3	Benzoic acid (1.0 equiv.)	62
4	$KHCO_3$ (1.5 equiv.) *(no K_2CO_3)*	67
5	$KHCO_3$ (1.5 equiv.) + AcOH (1.0 equiv.)	56
6	KOAc (1.0 equiv.) *(no K_2CO_3)*	34
7	KOAc (1.0 equiv.)	76
8	Only K_2CO_3 (0.7 equiv.)	49
9	Only K_2CO_3 (0.2 equiv.)	33
10	Only K_2CO_3 (0.5 equiv.)	11
11	NaOTf (1.0 equiv.)	35
12	NBu_4OAc (10 mol%)	80
13	NBu_4Br (10 mol%)	23

Reaction conditions: pyridine sulfone **1a** (0.22 mmol, 1.1 equiv.), 4-bromoanisole (0.20 mmol, 1.0 equiv.), K_2CO_3 (0.30 mmol, 1.5 equiv.), $Pd(OAc)_2$ (0.01 mmol, 5.0 mol%), CataCXium A (0.02 mmol, 10 mol%), toluene (2.0 mL, 0.1 M), 130 °C. [a]HPLC yield determined using *p*-ditolylether as an internal standard. Bu = butyl.

In order to help elucidate the mode of action of the acetic acid, we investigated the effects of alternative acid additives on the yield of biaryl **3a** (entries 2-3). The reaction of AcOH and K_2CO_3 produces various salt products, therefore we tried combinations of these salts in the reaction mixture to see the effect (entries 4-10). Ultimately none of these changes gave a yield that compared to the optimised system. It was postulated that the base and acid are buffering the system and potentially enabling a controlled release of the latent species to the sulfinate.

2.3 Monitoring the consumption of the sulfone starting material and formation of biaryl product.

To further investigate the effect of adding acetic acid into the system, the consumption of pyridine sulfone starting material (**1a** or **4a**) and formation of cross-coupled product (**3a**) was monitored under various cross-coupling conditions (Figure 1a -e). In order to achieve this, the reaction was run according to general procedure **F**, with internal standard in the reaction mixture (15 mg, 0.076 mmol, 0.38 equiv., *p*-ditolylether). Aliquots of the reaction mixture were removed and quenched at specific interval and analysed by HPLC. The amount of starting sulfone and cross-coupled biaryl product present in the reaction mixture were monitored.

On comparison of the plots showing the consumption of β- nitrile sulfone **1a** with AcOH additive in the system (Figure 1a) to that without AcOH (Figure 1b), it is evident that the presence of AcOH additive slows the rate of sulfone consumption, and therefore the rate of sulfinate release. With the presence of both K_2CO_3 and AcOH in the reaction medium, a mixture of salts could be generated *in situ,* such as $KHCO_3$ and KOAc. However reaction of β- nitrile sulfone **1a** and arylbromide **2a** using a mixture of these salts, instead of a direct AcOH additive, gave a much slower consumption of sulfone starting material (Figure 1c versus. 1a). Observation of slower rates of sulfone consumption with these salts suggests that their formation is not likely to occur at the start of the reaction, i.e AcOH does not react fully with K_2CO_3 on addition to the reaction. Use of AcOH additive in the reaction of β- ester sulfone **4a** with **2a** (Figure 1d)**,** results in a slower consumption of sulfone **4a,** and therefore slower release of sulfinate salt, compared to when no AcOH additive is used (Figure 1e). By comparing the plots showing the loss of β- nitrile sulfone **1a** under the conditions utilizing an AcOH additive (Figure 1a) to that showing the loss of β- ester sulfone **4a** under the same conditions (Figure 1d), we can see that β- ester sulfone **4a** is consumed at a slower rate than β- nitrile sulfone **1a**. In other words, under the cross-coupling conditions the β- nitrile sulfone **1a** and of β- ester sulfone **4a** are consumed, and release the sulfinate salt, at different rates (Figure 2). Therefore, use of AcOH additive affects the rate of sulfinate release from the corresponding masked sulfone (**1a** or **2a**) to different extents.

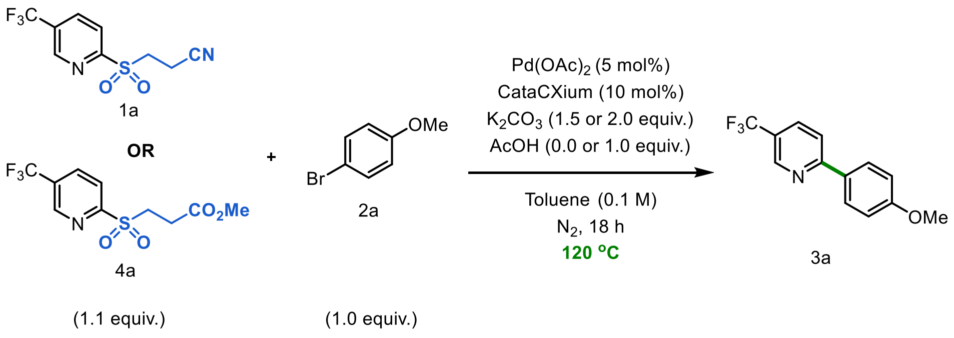

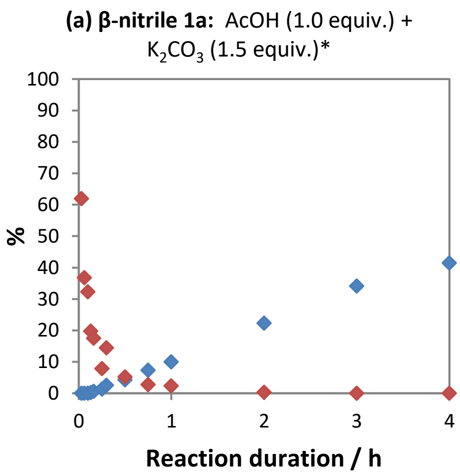

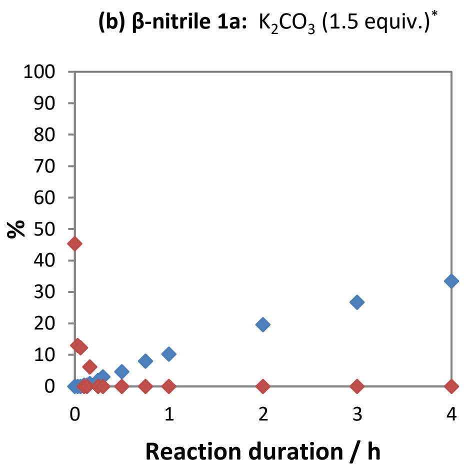

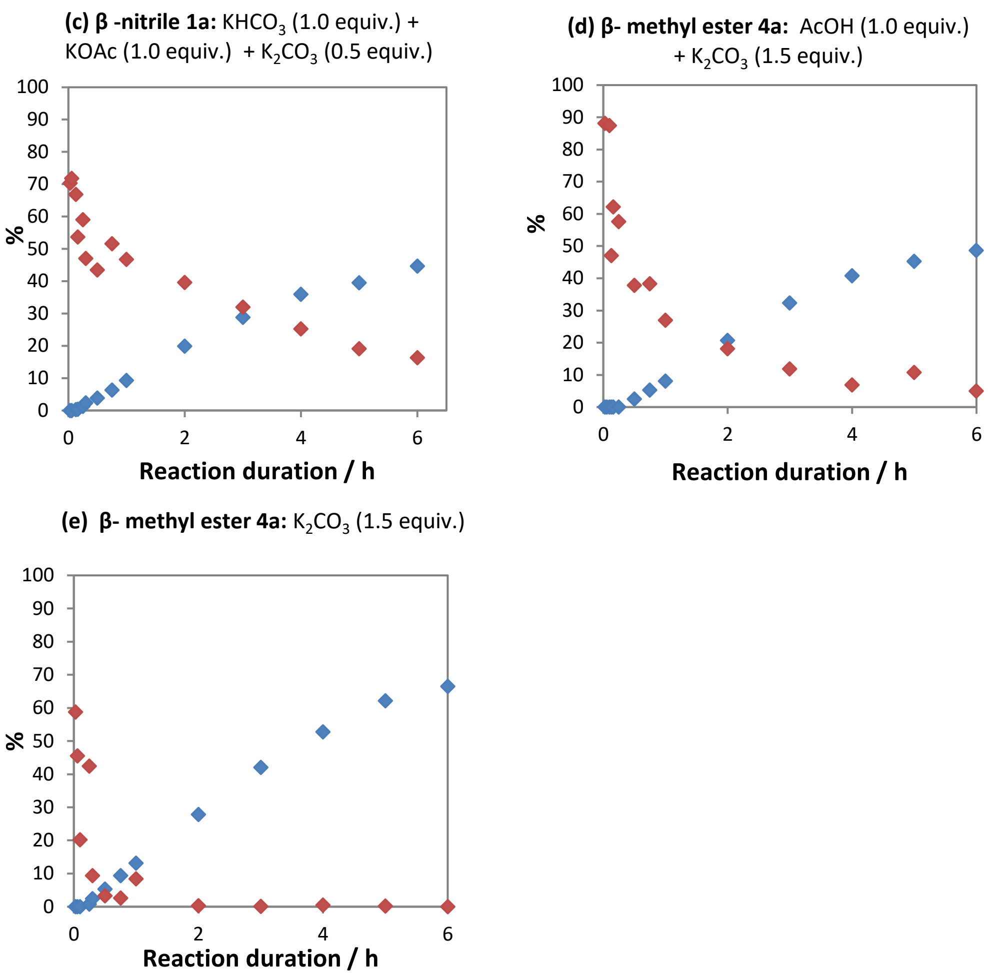

Figure 1: HPLC monitoring of the loss of starting material and the production of product in the reaction of β -nitrile pyridine sulfone (1a) or β –ester pyridine sulfone (4a) with 4- bromoanisole (2a). (a) Reaction of **1a** reaction containing AcOH (1.0 equiv.) and K_2CO_3, (b) Reaction of **1a** with K_2CO_3 (1.5 equiv.) and no AcOH, (c) Reaction of **1a** reaction with a combination of $KHCO_3$ (1.0 equiv.), KOAC (1.0 equiv.) and K_2CO_3 (0.5 equiv.), (d) Reaction of **3a** reaction containing AcOH (1.0 equiv.) and K_2CO_3 (1.5 equiv.), (e) Reaction of **4a** with K_2CO_3 (1.5 equiv.) and no AcOH. The yield/consumption (%) is determined by HPLC using *p*-ditolylether as an internal standard. *These data sets were reproduced multiple times (× 3).

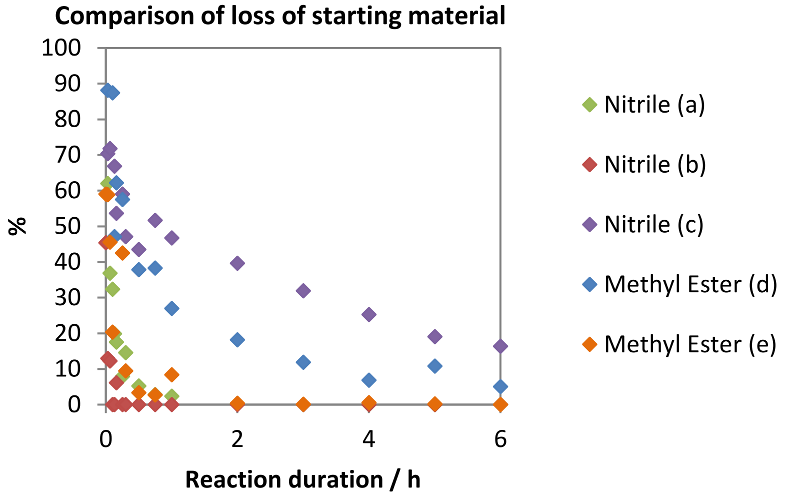

Figure 2: Comparison of the consumption of sulfone starting material under varying cross-coupling conditions (overlay of Figures 1a-e).

2.4 Optimisation of the β-methylester sulfone system

Table 5. Optimisation of the β-methylester sulfone system

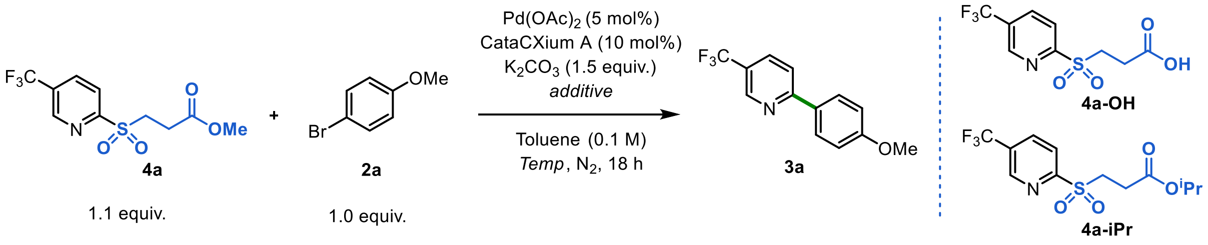

Entry	Variations from system above	Temp / °C	Yield[a] / %
**1**	AcOH (1.0 equiv.)	120	73 (71)
**2**	AcOH (1.0 equiv.)	130	(65)
**3**	AcOH (1.0 equiv.)	110	56
**4**	Pyridine sulfone (1.5 equiv.)	120	78
**5**	Using **4a-OH** instead of **4a**	120	2
**6**	Using **4a-OH** instead of **4a** *(no AcOH)*	120	1
**7**	Using **4a-iPr** instead of **4a**	120	59[b]
**8**	No AcOH	120	81
**9**	AcOH (0.25 equiv.)	120	78
**10**	AcOH (0.50 equiv.)	120	72
**11**	AcOH (0.75 equiv.)	120	77
**12**	No AcOH and K_2CO_3 (2.0 equiv.)	120	81
**13**	No AcOH and Cs_2CO_3 (1.5 equiv.)	120	84

**14**	P(^{t}Bu)$_2$Me·HBF_4 (10 mol%) *(no AcOH)*	120	81
**15**	PCy_3 (10 moL%) *(no AcOH)*	120	64
**16**	TMSOK (1.5 equiv.) instead of K_2CO_3	120	41
**17**	KOPiv (1.5 equiv.) instead of K_2CO_3	120	9
**18**	KO^{t}Am (25% in toluene) (1.5 equiv.) instead of K_2CO_3	120	36
**19**	Sulfone (1.5 equiv.) and Cs_2CO_3 (1.5 equiv.) *(no AcOH)*	120	84
**20**	Sulfone (1.5 equiv.) and Cs_2CO_3 (2.0 equiv.) *(no AcOH)*	120	88
**21**	Sulfone (1.5 equiv.) and K_2CO_3 (1.5 equiv.) *(no AcOH)*	120	91 (85)
**22**	Sulfone (1.5 equiv.) and K_2CO_3 (2.0 equiv.) *(no AcOH)*	120	92 (87)
**23**	Sulfone (1.5 equiv.) and K_2CO_3 (2.0 equiv.) *(no AcOH)*	130	88
**24**	Anisole as the solvent (0.1 M)	120	91 (91)
**25**	*p*-xylene as the solvent (0.1 M)	120	99 (89)
**26**	Toluene: Sulfolane (1:1) as the solvent (0.1 M)	120	93

Reaction conditions: pyridine sulfone **3a** (0.22 mmol, 1.1 equiv.), 4-bromoanisole **2a** (0.20 mmol, 1.0 equiv.), K_2CO_3 (0.30 mmol, 1.5 equiv.), $Pd(OAc)_2$ (0.01 mmol, 5.0 mol%), CataCXium A (0.02 mmol, 10 mol%), toluene (0.1 M). [a] HPLC yield determined using *p*-ditolylether as an internal standard, [b] Reaction run on a 0.10 mmol scale. Isolated yields are in brackets.

2.5 Optimisation of both the β-nitrile sulfone and β-methylester sulfone systems outside of a pressurised system

Table 6. Optimisation of the masked sulfone systems outside of a sealed vial.

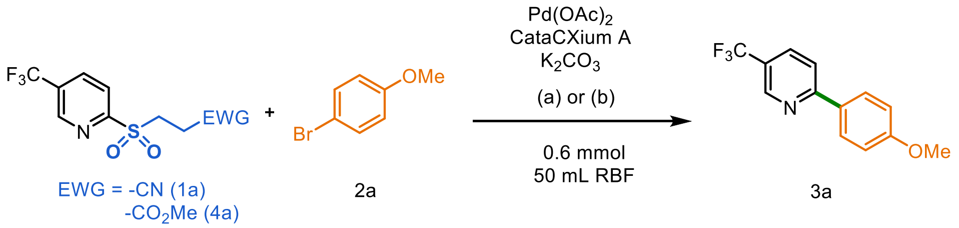

EWG	Entry	Solvent	Variations to system	Internal Temp.	HPLC yield / %
-CN (a)	1	toluene	-	110 °C	25
	2	mesitylene	-	120 °C	46
	3	mesitylene	-	130 °C	62
	4	mesitylene	no AcoH	130 °C	57
	5	mesitylene	propanoic acid instead of AcOH	130 °C	29
	6	*p*-xylene	-	130 °C	78 (68)
	7	toluene: sulfolane (1:1)	-	130 °C	55
	8	anisole	-	130 °C	68
	9	anisole	adjusted stoichiometry[a]	130 °C	88 (82)
$-CO_2Me$ (b)	1	*p*-xylene	-	130 °C	67
	2	toluene: sulfolane (1:1)	-	130 °C	87 (82)
	3	anisole	-	130 °C	91 (85)
	4	anisole	$P(^tBu)_2MeHBF_4$ instead of CataCXium A	130 °C	66
	5	anisole	-	120 °C	47

Reaction conditions: (a) nitrile pyridine sulfone (0.66 mmol, 1.1 equiv.), 4-bromoanisole (0.60 mmol, 1.0 equiv.), K_2CO_3 (0.90 mmol, 1.5 equiv.), AcOH (0.60 mmol, 1.0 equiv.), $Pd(OAc)_2$ (5.0 mol%), CataCXium A (10 mol%), solvent (0.1 M). (b) ester pyridine sulfone (0.90 mmol, 1.5 equiv.), 4-bromoanisole (0.60 mmol, 1.0 equiv.), K_2CO_3 (1.2 mmol, 1.5 equiv.), $Pd(OAc)_2$ (0.03 mmol, 5.0 mol%), CataCXium A (0.06 mmol, 10 mol%), solvent (0.1 M). [a] Adjusted Stoichiometry: pyridine sulfone (0.30 mmol, 1.5 equiv.), AcOH (0.30 mmol, 1.5 equiv.) and K_2CO_3 (0.40 mmol, 2.0 equiv.). HPLC yield determined using *p*-ditolylether as an internal standard. Isolated yields are in brackets.

3. Synthetic Procedures and characterisation data

3.1 HPLC information

The HPLC data was obtained from an Agilent Technologies 1200 series HPLC Hypersol ODS, 5 µm column (100 × 4.0 MM) using an extended 12 min method H_2O (0.1 % v/v H_3PO_4): Acetonitrile (10-95% (9 min) to 100% (10.5 min) to 10% (12 min)). HPLC yields for the ligand screening were obtained *via* standard calibration curve using authentic desired product/starting material and *p*-ditoylether as an internal standard. Yields were calculated from two different wavelengths 254 nm and 210 nm and averaged. The method was verified by product isolation which gave a result ± 2%.

Calibration curve for the production of the model cross-coupled product **3a**:

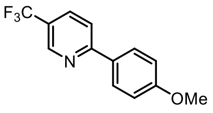

3a

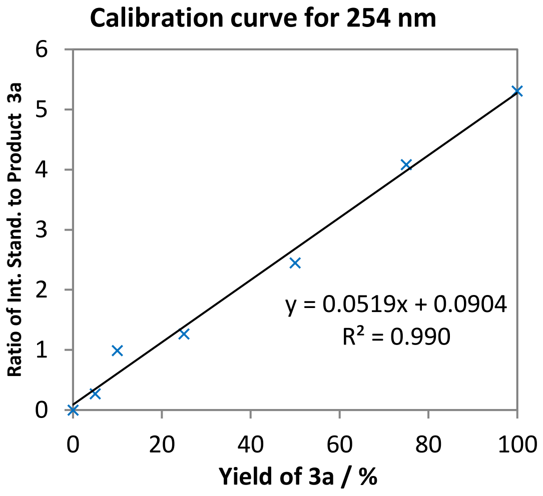

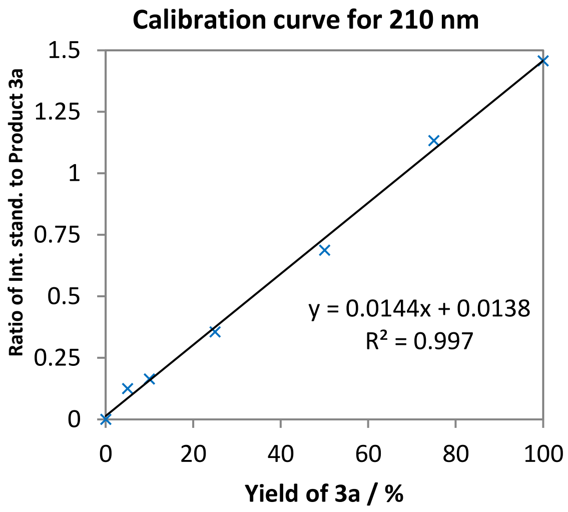

Calibration curve for the loss of the β-nitrile sulfone model starting material **1a**:

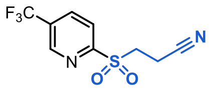

1a

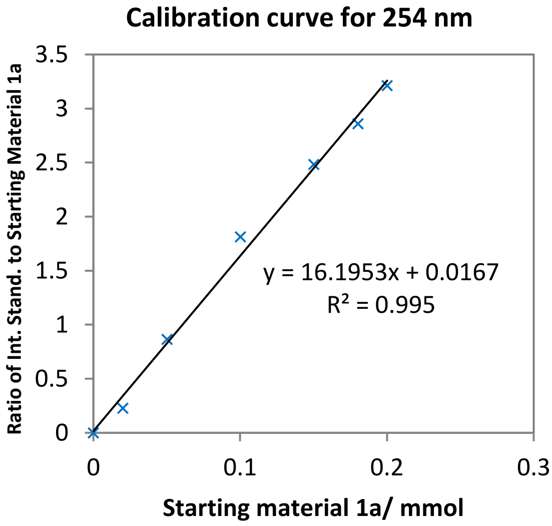

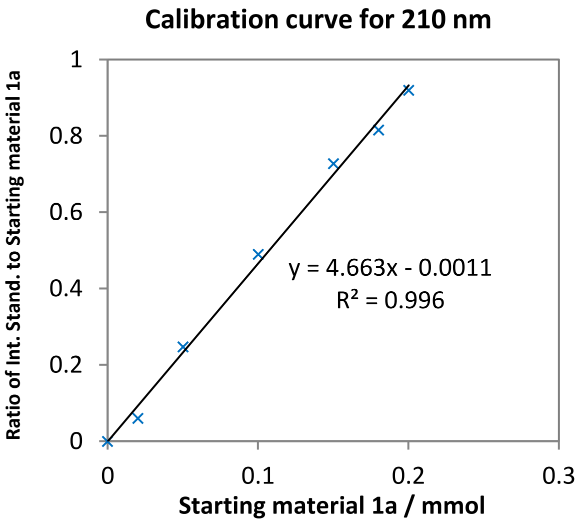

Calibration curve for the loss of the β-methylester sulfone model starting material **4a**:

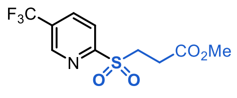

4a

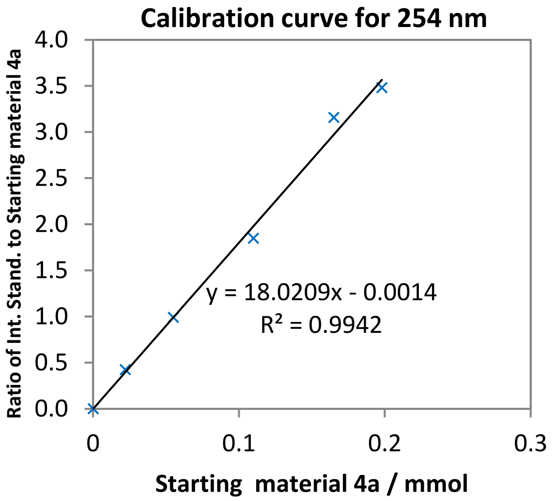

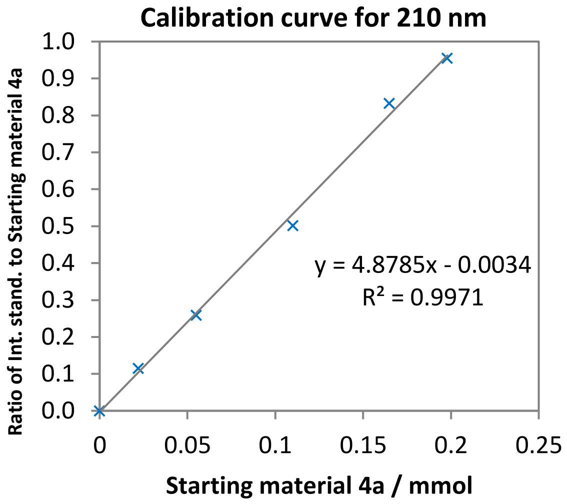

3.2 Synthesis of starting materials and Intermediates

3.2.a Synthesis of heteroaromatic thioethers

General Procedure A:

Under N_2: Acrylonitrile (5.0 equiv.) *or* methyl acrylate (5.0 equiv.) was added dropwise to a stirring solution of pyridine thiol (1.0 equiv.) and Et_3N (1.0 equiv.) in acetonitrile (0.3- 0.7 M). The resultant solution was heated to 40-45 °C for 18- 48 h. After which the reaction was allowed to cool to RT, and concentrated *in vacuo* to remove the excess acrylonitrile/ methyl acrylate and Et_3N. The resultant crude residue was then purified by flash column chromatography on silica gel to give the desired compound.

General Procedure B: Modified procedure from the procedures detailed by Wei *et. al.*[1]

Under N_2: Methyl-3-mercaptopropionate (1.5 equiv.) was added to a stirring suspension of heteroaryl chloride (1.0 equiv.) and K_2CO_3 (1.5 equiv.) in DMF (1.0 M). The reaction was stirred at RT for 24 h and monitored by TLC. The reaction mixture was then diluted with water and extracted with EtOAc thrice. The combined organic layers were then washed twice with 10% aqueous LiCl solution, washed with brine, dried over $MgSO_4$ and concentrated *in vacuo.* The crude residue was then purified by flash column chromatography on silica gel.

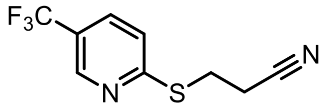

3-((5-(trifluoromethyl)pyridin-2-yl)thio)propanenitrile (*pre*-1a)

Following General procedure **A**, 5-(trifluoromethyl)pyridine-2-thiol (4.97 g, 27.7 mmol, 1.0 equiv.), acrylonitrile (9.20 mL, 139 mmol, 5.0 equiv.) and Et_3N (3.84 mL, 27.7 mmol, 1.0 equiv.) in acetonitrile (40.0 mL, 0.7 M) were heated at 40 °C for 23 h. The yellow crude residue was purified by flash column chromatography (20% EtOAc in Petrol) to give the title compound as a colourless oil (4.06 g, 63%).

^{1}H NMR (400 MHz, $CDCl_3$) δ 8.69 – 8.67 (m, 1H, Ar-*H*), δ 7.71 (app. ddd, *J* = 8.5, 2.5, 0.5 Hz, 1H, Ar-*H*), δ 7.30 (app. dp, *J* = 8.5, 1.0 Hz, 1H, , Ar-*H*), 3.46 (t, *J* = 7.0 Hz, 2H, SC*H*$_2$), 2.87 (t, *J* = 7.0 Hz, 2H, C*H*$_2$CN); **^{13}C NMR** (101 MHz, $CDCl_3$) δ 161.8, 146.5 (q, $^3J_{C-F}$ = 4.5 Hz), 133.1 (q, $^3J_{C-F}$ = 3.5 Hz), 123.7 (q, $^1J_{C-F}$ = 270.5 Hz), 123.1 (q, $^2J_{C-F}$ = 33.5 Hz), 122.2, 118.4, 25.7, 18.6; **^{19}F NMR** (377 MHz, $CDCl_3$) δ -62.22 (s); **LRMS** (ESI$^+$) *m/z* 180.0 [M- C_3H_3N+H]$^+$; **HRMS** (ESI$^+$) found *m/z* 233.0357 [M+H]$^+$, $C_9H_8N_2F_3S$ requires m/z 233.0355; **IR** ν_{max} (neat)/cm^{-1} 3067, 2937, 2252, 1598, 1558, 1323, 1113, 1009, 832; **R$_f$** = 0.45 (20% EtOAc in Petrol).

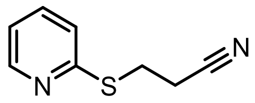

3-(pyridin-2-ylthio)propanenitrile (*pre*-1b)

Following General procedure **A**, 2-mercaptopyridine (1.11 g, 10.0 mmol, 1.0 equiv.), acrylonitrile (3.30 mL, 50.0 mmol, 5.0 equiv.) and Et_3N (1.39 mL, 10.0 mmol, 1.0 equiv.) in acetonitrile (15.0 mL, 0.6 M) was heated at 45 °C for 48 h. The yellow crude mixture was purified by flash column chromatography (20% EtOAc in Petrol) to give the title compound as a slightly yellow oil (696 mg, 42%).

^{1}H NMR (400 MHz, $CDCl_3$) δ 8.42 (ddd, *J* = 5.0, 2.0, 1.0 Hz, 1H, Ar-*H*), 7.50 (ddd, *J* = 8.0, 7.5, 2.0 Hz, 1H, Ar-*H*), 7.19 (dt, *J* = 8.0, 1.0 Hz, 1H, Ar-*H*), 7.02 (ddd, *J* = 7.5, 5.0, 1.0 Hz, 1H, Ar-*H*), 3.42 (t, *J* = 7.0 Hz, 2H, SC*H₂*), 2.86 (t, *J* = 7.0 Hz, 2H, C*H₂*CN); **^{13}C NMR** (101 MHz, $CDCl_3$) δ 156.3, 149.3, 136.0, 122.4, 119.7, 118.4, 25.3, 18.5; **LRMS** (ESI^+) *m/z* 165.0 $[M+H]^+$; **HRMS** (ESI^+) found *m/z* 165.0482 $[M+H]^+$, $C_8H_9N_2S$ requires m/z 165.0481; **IR** ν_{max} (neat)/cm^{-1} 3048, 2918, 2250, 1578, 1557, 1455, 1415, 1291, 1123, 987, 759, 723; **R_f** = 0.30 (20% EtOAc in Petrol). Data consistent with literature: M. Gholinejad, H. Firouzabadi, *New J. Chem.* **2015**, 39, 5953-5959.[2]

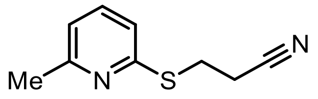

3-((6-methylpyridin-2-yl)thio)propanenitrile (*pre*-1c)

Following General procedure **A**, 6-methylpyridine-2-thiol (218 mg, 1.74 mmol, 1.0 equiv.), acrylonitrile (577 µL, 8.70 mmol, 5.0 equiv.) and Et_3N (243 µL, 1.74 mmol, 1.0 equiv.) in acetonitrile (5.0 mL, 0.3 M) was heated at 30 °C for 24 h. The yellow crude residue was purified by flash column chromatography (15% EtOAc in Petrol) to give the title compound as a slightly yellow oil (294 mg, 95%).

^{1}H NMR (400 MHz, $CDCl_3$) δ 7.39 (t, *J* = 8.0 Hz, 1H, Ar-*H*), 6.99 (d, *J* = 8.0 Hz, 1H, Ar-*H*), 6.87 (d, *J* = 8.0 Hz, 1H, Ar-*H*), 3.41 (t, *J* = 7.0 Hz, 2H, SCH_2), 2.86 (t, *J* = 7.0 Hz, 2H, CH_2CN), 2.49 (s, 3H, CH_3); **^{13}C NMR** (101 MHz, $CDCl_3$) δ 158.8, 155.5, 136.7, 119.6, 119.4, 118.8, 25.7, 24.6, 18.8; **LRMS** (ESI^+) *m/z* 179.0 $[M+H]^+$; **HRMS** (ESI^+) found *m/z* 179.0638 $[M+H]^+$, $C_9H_{11}N_2S$ requires m/z 179.0638; **IR** ν_{max} (neat)/cm^{-1} 2981, 2361, 2248, 1580, 1566, 1438, 1317, 1288, 1258, 1161, 1146, 1087, 958, 906, 869, 778, 729, 677, 615; **R_f** = 0.34 (20% EtOAc in Petrol).

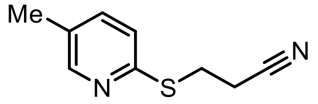

3-((5-methylpyridin-2-yl)thio)propanenitrile (*pre*-1d)

Following General procedure **A**, a solution of 5-methylpyridine-2-thiol (415 mg, 3.29 mmol, 1.0 equiv.), acrylonitrile (1.10 mL, 16.5 mmol, 5.0 equiv.) and Et_3N (459 µL, 5.00 mmol, 1.0 equiv.) in acetonitrile (10.0 mL, 0.3 M) was heated at 45 °C for 24 h. The resultant crude residue was purified by flash column chromatography (8-10% EtOAc in Petrol) to give the title compound as a slightly yellow oil (208 mg, 35%).

^{1}H NMR (400 MHz, $CDCl_3$) δ 8.26 – 8.25 (m, 1H, Ar-*H*), 7.33 (ddd, *J* = 8.0, 2.5, 1.0 Hz, 1H, Ar-*H*), 7.09 (dd, *J* = 8.0, 1.0 Hz, 1H, Ar-*H*), 3.39 (t, *J* = 7.0 Hz, 2H, SC*H*$_2$), 2.83 (t, *J* = 7.0 Hz, 2H, C*H*$_2$CN), 2.27 (s, 3H, C*H*$_3$); **^{13}C NMR** (101 MHz, $CDCl_3$) δ 153.2, 150.0, 137.3, 129.7, 122.4, 118.8, 25.8, 18.9, 18.0; **HRMS** (ESI$^+$) found *m/z* 179.0638 $[M+H]^+$, $C_9H_{11}N_1S$ requires m/z 179.0637; **IR** ν_{max} (neat)/cm^{-1} 2925, 2250, 1593, 1557, 1464, 1421, 1367, 1317, 1292, 1222, 1145, 1112, 1025, 906, 820, 732, 620; **R$_f$** =0.30 (10% EtOAc in Petrol).

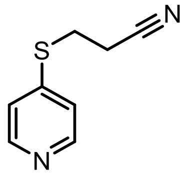

3-(pyridin-4-ylthio)propanenitrile (*pre*-1e)

Following General procedure **A**, 4-mercaptopyridine (500 mg, 4.50 mmol, 1.0 equiv.), acrylonitrile (1.49 mL, 22.0 mmol, 5.0 equiv.) and Et_3N (627 µL, 4.50 mmol, 1.0 equiv.) in acetonitrile (12.0 mL, 0.4 M) were heated at 40 °C for 24 h. The resultant crude residue was purified by flash column chromatography (100% EtOAc). The resultant orange crystalline solid was repurified by flash column chromatography (95% EtOAc in Petrol) to give the title compound as an off-white crystalline solid (211 mg, 29%).

^{1}H NMR (400 MHz, $CDCl_3$) δ 8.48 – 8.47 (m, 2H, Ar-*H*), 7.15 – 7.14 (m, 2H, Ar-*H*), 3.27 (t, *J* = 7.0 Hz, 2H, SC*H*$_2$), 2.74 (t, *J* = 7.0 Hz, 2H, C*H*$_2$CN); **^{13}C NMR** (101 MHz, $CDCl_3$) δ 150.0, 146.0, 121.5, 117.5, 26.7, 18.0; **LRMS** (ESI$^+$) *m/z* 165.0 $[M+H]^+$; **HRMS** (ESI$^+$) found *m/z* 165.0481 $[M+H]^+$, $C_8H_9N_2S$ requires m/z 165.0481; **IR** ν_{max} (neat)/cm^{-1} 2361, 2341, 2251, 1576, 1541, 1484, 1409, 1323, 1288, 1223, 1109, 1066, 986, 904, 805, 711, 669; **mp** 59-61 °C (EtOAc); **R$_f$** = 0.17 (100% EtOAc).

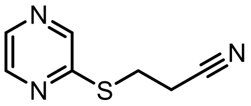

3-(pyrazin-2-ylthio)propanenitrile (*pre*-1f)

Following General procedure **A**, pyrazine-2-thiol (505 mg, 4.50 mmol, 1.0 equiv.), acrylonitrile (1.5 mL, 22.5 mmol, 5.0 equiv.) and Et_3N (630 µL, 4.50 mmol, 1.0 equiv.) in acetonitrile (6.5 mL, 0.7 M) were heated at 40 °C for 24 h. The concentrated crude residue was purified by flash column chromatography (10-20% EtOAc in Petrol) to give the title compound as an off-white solid (383 mg, 52%).

^{1}H NMR (400 MHz, $CDCl_3$) δ 8.47 (s, 1H, Ar-*H*), 8.37 – 8.36 (m, 1H, Ar-*H*), 8.26 (d, *J* = 3.0 Hz, 1H, Ar-*H*), 3.42 (t, *J* = 7.0 Hz, 2H, SC*H₂*), 2.83 (t, *J* = 7.0 Hz, 2H, C*H₂*CN); **^{13}C NMR** (101 MHz, $CDCl_3$) δ 154.9, 144.2, 144.1, 140.2, 118.2, 25.3, 18.6; **LRMS** (ESI^+) *m/z* 166.0 $[M+H]^+$; **HRMS** (ESI^+) found *m/z* 166.0435 $[M+H]^+$, $C_7H_8N_3S$ requires m/z 166.0433; **IR** ν_{max} (neat)/cm^{-1} 2981, 2888, 1564, 1508, 1386, 1129, 1048, 1007, 836; **R_f** = 0.40 (40% EtOAc in Petrol); **mp** 48-50 °C (CH_2Cl_2).

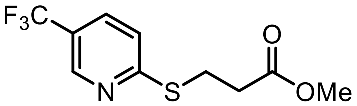

methyl 3-((5-(trifluoromethyl)pyridin-2-yl)thio)propanoate *(pre-*4a)

Following General procedure **A**, a solution of 5-(trifluoromethyl)pyridine-2-thiol (899 mg, 5.00 mmol, 1.0 equiv.), methyl acrylate (2.25 mL, 25.0 mmol, 5.0 equiv.) and Et_3N (697 µL, 5.00 mmol, 1.0 equiv.) in acetonitrile (7.2 mL, 0.7 M) was heated at 45 °C for 24 h. The resultant crude residue was purified by flash column chromatography (2% EtOAc in Petrol) to give the title compound as a colourless liquid (998 mg, 75%). *On a 15 mmol scale the reaction gave a comparable 73% yield.*

Following General Procedure **B,** 2-chloro-5-(trifluoromethyl)pyridine (182 mg, 1.00 mmol, 1.0 equiv.), K_2CO_3 (207 mg, 1.50 mmol, 1.5 equiv.) and methyl 3-mercaptopropionate (166 µL, 1.50 mmol, 1.5 equiv.) were combined in in DMF (1.0 mL, 1.0 M) was added. The reaction mixture was stirred at RT for 18 h and then diluted with water. The reaction mixture was poured into a separating funnel and the aqueous layer was washed with EtOAc (15 mL × 4). The organic layers were combined and washed with 10% aqueous LiCl solution (50 mL × 2). The organic layers were then dried over $MgSO_4$, filtered and concentrated *in vacuo*. The yellow crude oil was then purified by flash column chromatography (5% EtOAc in Petrol) to give the title product as a colourless liquid (205 mg, 77%).

^{1}H NMR (400 MHz, $CDCl_3$) δ 8.67 – 8.65 (m, 1H, Ar-*H*), 7.67 – 7.64 (m, 1H, Ar-*H*), 7.26 – 7.23 (m, 1H, Ar-*H*), 3.71 (s, 3H, CO_2CH_3), 3.47 (t, *J* = 7.0 Hz, 2H, SC*H₂*), 2.80 (t, *J* = 7.0 Hz, 2H, CH_2CO_2Me); **^{13}C NMR** (101 MHz, $CDCl_3$) δ 172.5, 163.5, 146.4 (q, $^3J_{C-F}$ = 4.0 Hz), 132.7 (q, $^3J_{C-F}$

= 3.5 Hz), 123.9 (q, $^{1}J_{C-F}$ = 272.0 Hz), 122.5 (q, $^{2}J_{C-F}$ = 33.0 Hz), 121.9, 52.0, 34.3, 25.1; **^{19}F NMR** (377 MHz, $CDCl_3$) δ -62.20 (s); **LRMS** (ESI^+) *m/z* 266.0 $[M+H]^+$; **HRMS** (ESI^+) found *m/z* 266.0458 $[M+H]^+$, $C_{10}H_{11}O_2NF_3S$ requires m/z 266.0457; **IR** ν_{max} (neat)/cm^{-1} 2928, 1606, 1563, 1481, 1458, 1384, 1327, 1165, 1129, 1083, 1015, 940, 851, 782, 753, 619; **R_f** = 0.37 (5% EtOAc in Petrol).

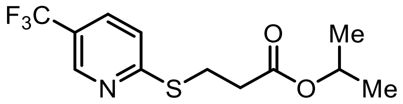

isopropyl 3-((5-(trifluoromethyl)pyridin-2-yl)thio)propanoate (*pre*-4a-iPr)

Note: Iso-propyl acrylate was made freshly via transesterification of methyl acrylate and then used directly as the crude in this reaction. Some methyl 3-((5-(trifluoromethyl)pyridin-2-yl)thio)propanoate side product was observed and isolated from this reaction.

Following General procedure **A**, a solution of 5-(trifluoromethyl)pyridine-2-thiol (179 mg, 1.00 mmol, 1.0 equiv.), crude iso-propyl acrylate in CH_2Cl_2 (12.0 mL, *c.a* 5.00 mmol, 5.0 equiv.) and Et_3N (140 µL, 5.00 mmol, 1.0 equiv.) in acetonitrile (2.0 mL, 0.5 M) was heated at 45 °C for 24 h. The resultant crude residue was purified by flash column chromatography (2% EtOAc in Petrol) to give the title compound as a colourless oil (61.6 mg, 21%).

^{1}H NMR (400 MHz, $CDCl_3$) δ 8.68 – 8.62 (m, 1H, Ar-*H*), 7.66 – 7.62 (dd, *J* = 2.5, 8.5 Hz, 1H, Ar-*H*), 7.24 (dt, *J* = 8.5, 1.0 Hz, 1H, Ar-*H*), 5.04 (hept, *J* = 6.5 Hz, 1H, CO_2CH), 3.45 (t, *J* = 7.0 Hz, 2H, SCH_2), 2.74 (t, *J* = 7.0 Hz, 2H, CH_2CO_2Me), 1.23 (d, *J* = 6.5 Hz, 6H, $C(CH_3)_2$); **^{13}C NMR** (101 MHz, $CDCl_3$) δ 171.5, 163.6, 146.4 (q, $^{1}J_{C-F}$ = 4.5 Hz), 132.7 (q, $^{1}J_{C-F}$ = 3.5 Hz), 123.9 (q, $^{3}J_{C-F}$ = 271.0 Hz), 122.4 (q, $^{2}J_{C-F}$ = 33.0 Hz), 121.9, 68.3, 34.8, 25.2, 22.0; **^{19}F NMR** (377 MHz, $CDCl_3$) δ -62.21 (s); **LRMS** (ESI^+) *m/z* 294.0 $[M+H]^+$; **HRMS** (ESI^+) found *m/z* 294.0772 $[M+H]^+$, $C_{12}H_{15}O_2NF_3S$ requires m/z 294.0770; **IR** ν_{max} (neat)/cm^{-1} 2361, 2341, 1598, 1557, 1473, 1378, 1324, 1251, 1167, 1114, 1075, 1010, 939, 931, 746; **R_f** = 0.30 (5% EtOAc in Petrol).

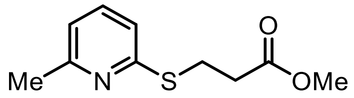

methyl 3-((6-methylpyridin-2-yl)thio)propanoate (*pre*-4b)

Following General procedure **A**, 6-methylpyridine-2-thiol (300 mg, 2.40 mmol, 1.0 equiv.), methyl acrylate (1.09 mL, 12.0 mmol, 5.0 equiv.) and Et_3N (335 µL, 2.40 mmol, 1.0 equiv.) in acetonitrile (3.5 mL, 0.7 M) were heated at 45 °C for 16 h. The crude residue was purified by flash column chromatography (5% EtOAc in petrol) to give the title compound as a colourless oil (410 mg, 81%).

^{1}H NMR (400 MHz, $CDCl_3$) δ 7.35 (t, *J* = 7.5 Hz, 1H, Ar-*H*), 6.96 (d, *J* = 8.0 Hz, 1H, Ar-*H*), 6.82 (d, *J* = 7.5 Hz, 1H, Ar-*H*), 3.70 (s, 3H, CO_2CH_3), 3.41 (t, *J* = 7.0 Hz, 2H, SCH_2), 2.78 (t, *J* = 7.0 Hz, 2H, CH_2CO_2Me), 2.48 (s, 3H, Ar-CH_3). **^{13}C NMR** (101 MHz, $CDCl_3$) δ 172.8, 158.6, 157.2, 136.4, 119.3, 119.0, 51.9, 34.7, 25.1, 24.6; **LRMS** (ESI^+) *m/z* 212.0 $[M+H]^+$; **HRMS** (ESI^+) found *m/z* 212.0741 $[M+H]^+$, $C_{10}H_{14}NO_2S$ requires m/z 212.0740; **IR** v_{max} (neat)/ cm^{-1} 3054, 2844, 1735, 1579, 1566, 1436, 1160, 776; **R_f** = 0.35 (10% EtOAc in Petrol).

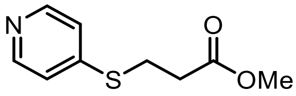

methyl 3-(pyridin-4-ylthio)propanoate (*pre*-4c)

Following General procedure **A**, pyridine-4-thiol (355 mg, 3.20 mmol, 1.0 equiv.), methyl acrylate (1.45 mL, 16.0 mmol, 5.0 equiv.) and Et_3N (445 µL, 3.20 mmol, 1.0 equiv.) in acetonitrile (4.7 mL, 0.7 M) were heated at 45 °C for 16 h. The crude residue was purified by flash column chromatography (30-50% EtOAc in petrol) to give the title compound as a yellow oil (160 mg, 25%).

^{1}H NMR (400 MHz, $CDCl_3$) δ 8.40 (dd, *J* = 4.5 Hz, 1.5 Hz, 2H, Ar-*H*), 7.11 (dd, *J* = 4.5 Hz, 1.5 Hz, 2H, Ar-*H*), 3.70 (s, 3H, CO_2CH_3), 3.25 (t, *J* = 7.5 Hz, 2H, SCH_2), 2.71 (t, *J* = 7.5 Hz, 2H, CH_2CO_2Me); **^{13}C NMR** (101 MHz, $CDCl_3$) δ 171.8, 149.6, 148.1, 120.9, 52.2, 33.5, 25.8; **LRMS** (ESI^+) *m/z* 198.0 $[M+H]^+$; **HRMS** (ESI^+) found *m/z* 198.0585 $[M+H]^+$, $C_9H_{12}NO_2S$ requires m/z 198.0583; **IR** v_{max} (neat)/cm^{-1} 3033, 2844, 1734, 1575, 1437, 1409, 1219, 804, 711; **R_f** = 0.21 (50% EtOAc in Petrol).

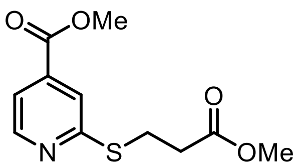

methyl 2-((3-methoxy-3-oxopropyl)thio)isonicotinate (*pre*-4e)

Following general procedure **B,** methyl 2-chloroisonicotinate (514.7 mg, 3.00 mmol, 1.0 equiv.), K_2CO_3 (621.9 mg, 4.50 mmol, 1.5 equiv.) and methyl 3-mercaptopropionate (498.5 µL, 4.50 mmol, 1.5 equiv.) were combined in dry DMF (3.0 mL, 1.0 M) to give a bright yellow suspension. The reaction was stirred at RT for 24 h and monitored by TLC (10% EtOAc in Petrol). The reaction mixture was then worked up and then purified by flash column chromatography (10% EtOAc in Petrol) to give the title product as a colourless oil (748 mg, 98%).

^{1}H NMR (400 MHz, $CDCl_3$) δ 9.00 (dd, *J* = 2.0, 1.0 Hz, 1H, Pyr-*H*), 8.02 (dd, *J* = 8.5, 2.0 Hz, 1H, Pyr-*H*), 7.20 (dd, *J* = 8.5, 1.0 Hz, 1H, Pyr-*H*), 3.92 (s, 3H, CO_2Me), 3.70 (s, 3H, CO_2Me), 3.47 (t, *J* = 7.0 Hz, 2H, SCH_2), 2.80 (t, *J* = 7.0 Hz, 2H, CH_2CO_2Me); **^{13}C NMR** (101 MHz, $CDCl_3$) δ 172.5, 166.0, 164.1, 150.9, 136.5, 122.0, 121.6, 52.3, 51.9, 34.4, 25.1; **LRMS** (ESI$^+$) *m/z* 256.0 [M+H]$^+$; **HRMS** (ESI$^+$) found *m/z* 256.0639 [M+H]$^+$, $C_{11}H_{14}O_4NS$ requires m/z 256.0638; **IR** v_{max} (neat)/cm^{-1} 2954, 2953, 1722, 1588, 1549, 1435, 1361, 1291, 1272, 1251, 1194, 1128, 1018, 980, 831, 767; **R$_f$** = 0.18 (10% EtOAc in Petrol).

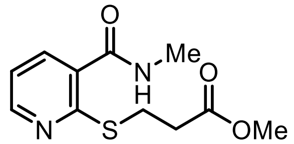

methyl 3-((3-(methylcarbamoyl)pyridin-2-yl)thio)propanoate (*pre*-4f)

Following general procedure **B,** 2-chloro-N-methylnicotinamide (341.2 mg, 2.00 mmol, 1.0 equiv.), K_2CO_3 (414.6 mg, 3.00 mmol, 1.5 equiv.) and methyl 3-mercaptopropionate (332 µL, 3.00 mmol, 1.5 equiv.) were combined in dry DMF (2.0 mL, 1.0 M) to give a slightly yellow suspension. The reaction was stirred at RT for 24 h and monitored by TLC. The reaction mixture was worked up and then purified by flash column chromatography (60% EtOAc in Petrol) to give the title product as a bright white powdery solid (414.2 mg, 81%).

^{1}H NMR (400 MHz, $CDCl_3$) δ 8.47 (dd, *J* = 5.0, 1.5 Hz, 1H, Ar-*H*), 7.81 (ddd, *J* = 7.5, 3.0, 1.5 Hz, 1H, Ar-*H*), 7.06 (ddd, *J* = 7.5, 5.0, 1.0 Hz, 1H, Ar-*H*), 6.29 (br. s, 1H, N*H*), 3.70 (s, 3H, CO_2Me), 3.47 (t, *J* = 7.0, 2H, SCH_2), 3.01 (d, *J* = 5.0, 3H, NH*Me*), 2.79 (t, *J* = 7.0 Hz, 2H, CH_2CO_2Me); **^{13}C NMR** (101 MHz, $CDCl_3$) δ 172.7, 167.1, 156.2, 150.6, 136.5, 130.0, 119.3, 51.9, 34.4, 26.9, 25.5; **LRMS** (ESI$^+$) *m/z* 277.0 [M+Na]$^+$; **HRMS** (ESI$^+$) found *m/z* 255.0799 [M+H]$^+$, $C_{11}H_{15}O_3N_2S$

requires m/z 255.0798; ; **IR** v_{max} (neat)/cm^{-1} 3069, 2951, 1735, 1639, 1578, 1556, 1438, 1410, 1394, 1359, 1318, 1255,1243, 1226, 1171, 1137, 1086, 1063, 1017, 997, 979, 842, 817; **mp** 118-120 °C (EtOAc); **R$_f$** = 0.32 (80% EtOAc in Petrol).

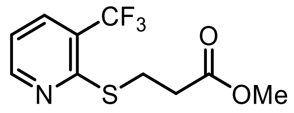

methyl 3-((3-(trifluoromethyl)pyridin-2-yl)thio)propanoate (*pre*-4g)

Following general procedure **B,** 2-chloro-3-trifluoromethylpyridine (908 mg, 5.00 mmol, 1.0 equiv.), K_2CO_3 (1.04 g, 7.50 mmol, 1.5 equiv.) and methyl 3-mercaptopropionate (831 µL, 7.50 mmol, 1.5 equiv.) were combined in dry DMF (5.0 mL, 1.0 M) to give a yellow suspension. The reaction was stirred at RT for 24 h and monitored by TLC. The reaction mixture was then worked up and then purified by flash column chromatography (15% EtOAc in Petrol) to give the title product as a colourless oil (898 mg, 68%).

^{1}H NMR (400 MHz, $CDCl_3$) δ 8.55 (ddd, *J* = 5.0, 2.0, 1.0 Hz, 1H, Pyr-*H*), 7.80 (ddd, *J* = 7.5, 1.5, 1.0 Hz, 1H, Pyr-*H*), 7.09 (ddd, *J* = 8.0, 5.0, 1.0 Hz, 1H, Pyr-*H*), 3.71 (s, 3H, CO_2Me), 3.50 (t, *J* = 7.0 Hz, 2H, CH_2CO_2Me), 2.79 (t, *J* = 7.0 Hz, 2H, SCH_2); **^{13}C NMR** (101 MHz, $CDCl_3$) δ 172.5, 157.7, 151.6, 134.5 (q, $^3J_{C-F}$ = 5.0 Hz), 123.9 (q, $^2J_{C-F}$ = 33.0 Hz), 123.4 (q, $^1J_{C-F}$ = 273.1 Hz), 118.5, 51.9, 34.4, 25.2; **^{19}F NMR** (377 MHz, $CDCl_3$) δ -63.36 (s); **LRMS** (ESI$^+$) *m/z* 266.0 [M+H]$^+$; **HRMS** (ESI$^+$) found *m/z* 266.0457 [M+H]$^+$, $C_{10}H_{11}O_2N_1F_3S$ requires m/z 266.0457; **IR** v_{max} (neat)/ cm^{-1} 3000, 2955, 2360, 1737, 1586, 1561, 1438, 1406, 1359, 1317, 1255, 1238, 1199, 1167, 1129, 1113, 1067, 1028, 981, 895, 807, 751, 717, 642; **R$_f$** = 0.24 (20% EtOAc in Petrol).

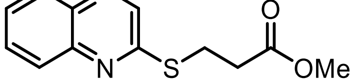

methyl 3-(quinoxalin-2-ylthio)propanoate (*pre*-4h)

Following general procedure **B,** chloroquinoxaline (329.2 mg, 2.00 mmol, 1.0 equiv.), K_2CO_3 (414.6 mg, 3.00 mmol, 1.5 equiv.) and methyl 3-mercaptopropionate (332 µL, 3.00 mmol, 1.5 equiv.) were combined in dry DMF (2.0 mL, 1.0 M) to give a light brown suspension. The reaction was stirred at RT for 24 h and monitored by TLC. The reaction mixture was then worked up and then purified by flash column chromatography (8-12% EtOAc in Petrol) to give the title product as a slightly yellow oil (440.5 mg, 89%).

^{1}H NMR (400 MHz, $CDCl_3$) δ 8.57 (s, 1H, Ar-*H*), 8.01 (dd, *J* = 8.5, 1.5 Hz, 1H, Ar-*H*), 7.92 (dd, *J* = 8.5, 1.5 Hz, 1H, Ar-*H*), 7.69 (ddd, *J* = 8.5, 7.0, 1.5 Hz, 1H, Ar-*H*), 7.62 (ddd, *J* = 8.4, 7.0, 1.5 Hz, 1H, Ar-*H*), 3.73 (s, 3H, CO_2Me), 3.59 (t, *J* = 7.0 Hz, 2H, SCH_2), 2.89 (t, *J* = 7.0 Hz, 2H, CH_2CO_2Me);

^{13}C NMR (101 MHz, $CDCl_3$) δ 172.5, 155.5, 144.9, 142.8, 140.1, 130.3, 129.4, 128.3, 128.0, 52.0, 34.0, 24.6; **LRMS** (ESI$^+$) *m/z* 249.0 (52%, $[M+H]^+$), 163.0 (48%, $[M+H-C_4H_8O_2]^+$; **HRMS** (ESI$^+$) found *m/z* 249.0695 $[M+H]^+$, $C_{12}H_{13}O_2N_2S$ requires m/z 249.0692; **IR** v_{max} (neat)/cm^{-1} 3061, 2999, 2951, 2846, 1737, 1542, 1487, 1437, 1359, 1248, 1223, 1197, 1174, 1152, 1125, 1086, 1016, 980, 962, 762; **R$_f$** = 0.39 (20% EtOAc in Petrol).

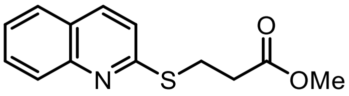

methyl 3-(quinolin-2-ylthio)propanoate (*pre*-4i)

Following general procedure **B**, 2-chloroquinoline (168.6 mg, 1.0 mmol, 1.0 equiv.), K_2CO_3 (207.3 mg, 1.5 mmol, 1.5 equiv.) and methyl-3-mercaptopropionate (166.1 µL, 1.5 mmol, 1.5 equiv.) were combined in dry DMF (1 mL, 0.1 M) to give a cloudy white suspension. The reaction was stirred at RT for 24 h and monitored by TLC (5% EtOAc in Petrol). The reaction mixture was worked up and purified by flash column chromatography (3% EtOAc in Petrol) to give the title product as a colourless oil (99.0 mg, 40%).

^{1}H NMR (400 MHz, $CDCl_3$) δ 7.93 (ddt, *J* = 8.5, 1.5, 1.0 Hz, 1H, Ar-*H*), 7.88 (dd, *J* = 8.5, 1.0 Hz, 1H, Ar-*H*), 7.71 (dd, *J* = 8.0, 1.5 Hz, 1H, Ar-*H*), 7.64 (ddd, *J* = 8.5, 7.0, 1.5 Hz, 1H, Ar-*H*), 7.43 (ddd, *J* = 8.0, 7.0, 1.0 Hz, 1H, Ar-*H*), 7.19 (d, *J* = 8.5 Hz, 1H, Ar-*H*), 3.72 (s, 3H, CO_2Me), 3.59 (t, *J* = 7.0 Hz, 2H, SCH_2), 2.90 (t, *J* = 7.0 Hz, 2H, CH_2CO_2Me); **^{13}C NMR** (101 MHz, $CDCl_3$) δ 172.9, 158.5, 148.5, 135.6, 129.8, 128.3, 127.8, 126.1, 125.5, 121.1, 51.9, 34.5, 24.9; **LRMS** (ESI$^+$) *m/z* 248.0 $[M+H]^+$; **HRMS** (ESI$^+$) found *m/z* 248.07395 $[M+H]^+$, $C_{13}H_{14}O_2NS$ requires m/z 248.07398; **IR** v_{max} (neat)/ cm^{-1} 3057, 3035, 2980, 2953, 2907, 2890, 1735,1614, 1594, 1556, 1498, 1435, 1420, 1357, 1294, 1247, 1197, 1172, 1138, 1089, 1018, 979, 943, 861, 817, 781, 750, 630; **R$_f$** = 0.3 (5% EtOAc in Petrol).

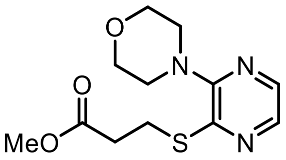

methyl 3-((3-morpholinopyrazin-2-yl)thio)propanoate (*pre*-4j)

Following general procedure **B,** 4-(3-chloropyrazin-2-yl)morpholine (399.3 mg, 2.00 mmol, 1.0 equiv.), K_2CO_3 (414.6 mg, 3.00 mmol, 1.5 equiv.) and methyl-3-mercaptopropionate (332.3 µL, 3.00 mmol, 1.5 equiv.) were combined in dry DMF (2.0 mL, 0.1 M) to give a cloudy yellow suspension. The reaction was stirred at RT for 24 h and monitored by TLC (20% EtOAc in Petrol). The reaction mixture was worked up and purified by flash column chromatography (10% EtOAc in Petrol) to give the title product as a pale yellow oil (472 mg, 83%).

^{1}**H NMR** (400 MHz, $CDCl_3$) δ 8.00 (dd, *J* = 2.5, 1.0 Hz, 1H, Ar-*H*), 7.91 (dd, *J* = 2.5, 1.0 Hz, 1H, Ar-*H*), 3.88 – 3.82 (m, 4H, O(C*H*$_2$)$_2$), 3.71 (s, 3H, CO_2*Me*), 3.40 (t, *J* = 7.0, 2H, SC*H*$_2$), 3.31 – 3.23 (m, 4H, N(C*H*$_2$)$_2$), 2.77 (t, *J* = 7.0 Hz, 2H, C*H*$_2$$CO_2$Me); ^{13}**C NMR** (101 MHz, $CDCl_3$) δ 172.6, 155.4, 148.9, 137.1, 136.6, 67.0, 51.9, 49.3, 34.3, 24.8; **LRMS** (ESI$^+$) *m/z* 284.0 $[M+H]^+$; **HRMS** (ESI$^+$) found *m/z* 284.1063 $[M+H]^+$, $C_{12}H_{18}O_3N_3S$ requires m/z 284.1063; **IR** v_{max} (neat)/ cm^{-1} 2962, 2854, 1741, 1591, 1557, 1509, 1441, 1408, 1366, 1323, 1287, 1267, 1246, 1188, 1141, 1116, 1069, 1043, 940, 845, 800, 761, 691, 621; **R$_f$** = 0.50 (20% EtOAc in Petrol).

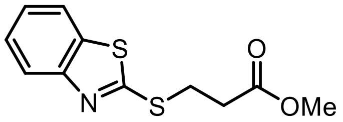

methyl 3-(benzo[d]thiazol-2-ylthio)propanoate (*pre*-4k)

Following General procedure **A**, benzo[d]thiazole-2-thiol (4.00 g, 24.0 mmol, 1.0 equiv.), methyl acrylate (11.0 mL, 120 mmol, 5.0 equiv.) and Et_3N (3.4 mL, 24.0 mmol, 1.0 equiv.) in acetonitrile (34 mL, 0.7 M) were heated at 45 °C for 16 h. The crude residue was purified by flash column chromatography (2- 5% EtOAc in Petrol) to give the title compound as pale pink crystals (1.72 g, 28%).

^{1}**H NMR** (400 MHz, $CDCl_3$) δ 7.88 (d, *J* = 8.0 Hz, 1H, Ar-*H*), 7.75 (d, *J* = 8.0 Hz, 1H, Ar-*H*), 7.42 (t, *J* = 7.5 Hz, 1H, Ar-*H*), 7.30 (t, *J* = 7.5 Hz, 1H, Ar-*H*), 3.73 (s, 3H, OC*H*$_3$), 3.62 (t, *J* = 7.0 Hz, 2H, SC*H*$_2$$CH_2$), 2.95 (t, *J* = 7.0 Hz, 2H, C*H*$_2$$CO_2$Me); ^{13}**C NMR** (101 MHz, $CDCl_3$) δ 172.0, 166.0, 153.1, 135.3, 126.1, 124.3, 121.6, 121.0, 51.9, 34.2, 28.2; **HRMS** (ESI$^+$) found *m/z* 276.0123 $[M+Na]^+$, $C_{11}H_{11}NO_2S_2Na$ requires m/z 276.0123; **IR** v_{max} (neat)/ cm^{-1} 3061, 2844, 1733, 1427, 995, 755; **R$_f$** = 0.37 (10% EtOAc in Petrol); **mp** 48-54 °C (CH_2Cl_2) . Data is consistent with literature: L. Shi, X. Liu, H. Zhang, Y. Jiang and D. Ma, *J. Org. Chem.* 2011, **76**, 10, 4200-4204.[3]

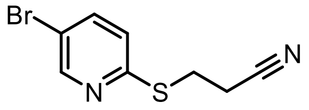

3-((5-bromopyridin-2-yl)thio)propanenitrile (*pre*-9a)

Following General procedure **A**, 5-bromopyridine-2-thiol (380.1 mg, 2.00 mmol, 1.0 equiv.), acrylonitrile (663 µL, 10.0 mmol, 5.0 equiv.) and Et_3N (279 µL, 2.00 mmol, 1.0 equiv.) in acetonitrile (4.0 mL, 0.5 M) was heated at 45 °C for 22.5 h. The resultant crude residue was purified by flash column chromatography (10-20% EtOAc in Petrol) to give the title compound as a white solid (383.2 mg, 79%).

^{1}H NMR (400 MHz, $CDCl_3$) δ 8.51 – 8.43 (m, 1H, Pyr-*H*), 7.61 (dt, *J* = 8.5, 2.0 Hz, 1H, Pyr-*H*), 7.12 – 7.05 (m, 1H, Pyr-*H*), 3.39 (t, *J* = 7.0 Hz, 2H, SCH_2), 2.83 (t, *J* = 7.0 Hz, 2H, CH_2CN); **^{13}C NMR** (101 MHz, $CDCl_3$) δ 155.6, 150.5, 139.0, 123.8, 118.5, 116.8, 25.9, 18.7; **LRMS** (ESI^+) *m/z* 189.9 (50%, $[M-CH_2CHCN+H]^+$), 191.9 (50%, $[M-CH_2CHCN+H]^+$); **HRMS** (ESI^+) found *m/z* 242.9588 $[M+H]^+$, $C_8H_8N_2S^{35}Br$ requires m/z 242.9586; **IR** ν_{max} (neat)/ cm^{-1} 3043, 2928, 2361, 1561, 1446, 1351, 1290, 1223, 1141, 1115, 1089, 1000, 906, 821, 726, 626; **mp** 77-79 °C (EtOAc); **R_F** = 0.42 (20% EtOAc in Petrol).

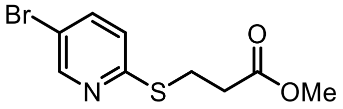

methyl 3-((5-bromopyridin-2-yl)thio)propanoate (*pre*-9b)

Following General procedure **A**, 5-bromopyridine-2-thiol (380.1 mg, 2.00 mmol, 1.0 equiv.), methyl acrylate (900 µL, 10.0 mmol, 5.0 equiv.) and Et_3N (279 µL, 2.00 mmol, 1.0 equiv.) in acetonitrile (4.0 mL, 0.5 M) was heated at 45 °C for 24 h. The resultant crude residue was purified by flash column chromatography (5% EtOAc in Petrol) to give the title compound as a slightly yellow clear oil (481 mg, 87%).

^{1}H NMR (400 MHz, $CDCl_3$) δ 8.46 (dd, *J* = 2.5, 1.0 Hz, 1H, Pyr-*H*), 7.57 (dd, *J* = 8.5, 2.5 Hz, 1H, Pyr-*H*), 7.05 (dd, *J* = 8.5, 1.0 Hz, 1H, Pyr-*H*), 3.70 (s, 3H, CO_2Me), 3.39 (t, *J* = 7.0 Hz, 2H, SCH_2), 2.76 (t, *J* = 7.0 Hz, 2H, CH_2CO_2Me); **^{13}C NMR** (101 MHz, $CDCl_3$) δ 172.5, 157.1, 150.4, 138.6, 123.6, 116.3, 51.9, 34.4, 25.3; **LRMS** (ESI^+) *m/z* 189.9 (30%, $[M-CH_2CHCO_2Me+H]^+$), 191.9 (30%, $[M-CH_2CHCO_2Me+H]^+$), 276.0 (15%, $[M+H]^+$), 278.0 (15%, $[M+H]^+$); **HRMS** (ESI^+) found *m/z* 275.9690 $[M+H]^+$, $C_9H_{11}NO_2S^{35}Br$ requires m/z 275.9688**;** **IR** ν_{max} (neat)/ cm^{-1} 2981, 2952, 2890, 1736, 1561, 1541, 1446, 1417, 1351, 1277, 1247, 1221, 1197, 1173, 1141, 1115, 1089, 1017, 999, 980, 945, 819, 726; **R_f** = 0.6 (20% EtOAc in Petrol).

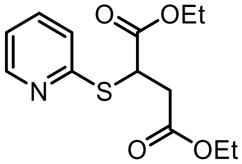

diethyl 2-(pyridin-2-ylthio)succinate (*pre*-5a)

Following General procedure **A**, 2-mercaptopyridine (222.3 mg, 2.00 mmol, 1.0 equiv.), diethyl fumarate (1.64 mL, 10.0 mmol, 5.0 equiv.) and Et_3N (279 µL, 2.00 mmol, 1.0 equiv.) in acetonitrile (3.0 mL, 0.7 M) was heated at 45 °C for 24 h. The resultant crude residue was purified by flash column chromatography (3% EtOAc in Petrol) to give the title compound as slightly yellow oil (479 mg, 84%).

^{1}H NMR (400 MHz, $CDCl_3$) δ 8.42 – 8.39 (m, 1H, Pyr-*H*), 7.50 (app. td, *J* = 7.5, 2.0 Hz, 1H, Pyr-*H*), 7.20 (d, *J* = 8.0 Hz, 1H, Pyr-*H*), 7.03 – 6.99 (m, 1H, Pyr-*H*), 4.95 (dd, *J* = 8.5, 5.5 Hz, 1H, SC*H*), 4.24 – 4.11 (m, 4H, 2 × OCH_2CH_3), 3.14 (dd, *J* = 17.0, 8.5 Hz, 1H, $CH_aH_bCO_2Et$), 2.98 (dd, *J* = 17.0, 5.5 Hz, 1H, CH_aH_bCOOEt), 1.23 (dt, *J* = 12.0, 7.0 Hz, 6H, 2 × CH_2CH_3); ^{13}C NMR (101 MHz, $CDCl_3$) δ 171.3, 171.0, 156.5, 149.6, 136.4, 122.6, 120.3, 61.8, 61.0, 41.9, 37.3, 14.3, 14.1; **HRMS** (ESI$^+$) found *m/z* 284.0950 [M+H]$^+$, $C_{13}H_{18}O_4N^{32}S$ requires m/z 284.0951; **IR** ν_{max} (neat)/cm^{-1} 3048, 2017, 2936, 2936, 2841, 1729, 1578, 1558, 1455, 1416, 1371, 1326, 1300, 1235, 1204, 1175, 1121, 1096, 1028, 988, 858, 761; **R_f** = 0.41 (20% EtOAc in Petrol). Data is consistent with literature: T. Hideo, F. Misa and Y. Masataka, *Bulletin of the Chemical Society of Japan*, 1991, **64**, 57-67.[4]

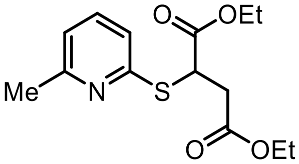

diethyl 2-((6-methylpyridin-2-yl)thio)succinate (*pre*-5b)

Following General procedure **A**, 6-methylpyridine-2-thiol (125.2 mg, 1.00 mmol, 1.0 equiv.), diethylfumarate (818.5 µL, 5.00 mmol, 5.0 equiv.) and Et_3N (139.5 µL, 1.00 mmol, 1.0 equiv.) in acetonitrile (2.0 mL, 0.5 M) was heated at 45 °C for 24 h. The resultant crude residue was purified by flash column chromatography (2-10% EtOAc in Petrol) to give the title compound as a colourless oil (207 mg, 70%).

^{1}H NMR (400 MHz, $CDCl_3$) δ 7.38 (t, *J* = 7.5 Hz, 1H, Pyr-*H*), 7.01 (d, *J* = 7.5 Hz, 1H, Pyr-*H*), 6.86 (d, *J* = 7.5 Hz, 1H, Pyr-*H*), 4.87 (dd, *J* = 8.5, 5.5 Hz, 1H, SC*H*), 4.17 (dq, *J* = 12.0, 7.0 Hz, 4H, 2 × OCH_2CH_3), 3.15 (dd, *J* = 17.0, 8.5 Hz, 1H, $CH_aH_bCO_2Et$), 2.97 (dd, *J* = 17.0, 5.5 Hz, 1H, $CH_aH_bCO_2Et$), 2.47 (s, 3H, Pyr-CH_3), 1.23 (dt, *J* = 12.0, 7.0 Hz, 6H, 2 × CH_2CH_3); **^{13}C NMR** (101 MHz, $CDCl_3$) δ 171.4, 171.1, 158.6, 155.3, 136.7, 119.7, 119.6, 61.7, 61.0, 42.2, 37.3, 24.4,

14.3, 14.2; **LRMS** (ESI$^+$) *m/z* 298.0 (50%,[M+H]$^+$), 320.0 (50%,[M+Na]$^+$); **HRMS** (ESI$^+$) found *m/z* 298.1107 [M+H]$^+$, $C_{14}H_{20}O_4NS$ requires m/z 298.1108; **IR** ν_{max} (neat)/ cm^{-1} 2983, 1732, 1580, 1567, 1439, 1371, 1162, 1030, 779; **R$_f$** = 0.47 (20% EtOAc in Petrol).

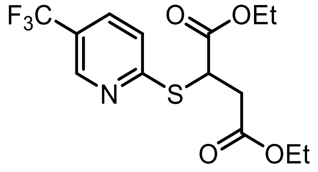

diethyl 2-((5-(trifluoromethyl)pyridin-2-yl)thio)succinate (*pre*-5c)

Following General procedure **A**, 5-(trifluoromethyl)pyridine-2-thiol (1.79 g, 10.0 mmol, 1.0 equiv.), diethyl fumarate (8.20 mL, 50.0 mmol, 5.0 equiv.) and Et_3N (1.40 mL, 10.0 mmol, 1.0 equiv.) in acetonitrile (15.0 mL, 0.7 M) were heated at 40 °C for 24 h. The concentrated crude residue was purified by flash column chromatography (0-10% EtOAc in Petrol) to give the title compound as a yellow oil (3.46 g, 98%).

^{1}H NMR (400 MHz, $CDCl_3$) δ 8.65 (s, 1H, Ar-*H*), 7.70 (d, *J* = 8.5 Hz, 1H, Ar-*H*), 7.29 (d, *J* = 8.5 Hz, 1H, Ar-*H*), 5.06 – 4.99 (m, 1H, SC*H*), 4.30 – 4.09 (m, 4H, OC*H$_2$*), 3.15 (dd, *J* = 17.0, 8.0 Hz, 1H, SCHC*H$_2$*), 2.99 (dd, *J* = 17.0, 5.5 Hz, 1H, SCHC*H$_2$*), 1.35 – 1.17 (m, 6H, CH_2CH_3); **^{13}C NMR** (101 MHz, $CDCl_3$) δ 170.8, 170.6, 161.6, 146.3 (q, $^3J_{C-F}$ = 5.0 Hz), 133.2 (q, $^3J_{C-F}$ = 3.5 Hz), 123.8 (q, $^1J_{C-F}$ = 273.5 Hz), 122.8 (q, $^1J_{C-F}$ = 33.5 Hz), 121.8, 62.1, 61.2, 42.0, 37.0, 14.3, 14.1; **^{19}F NMR** (377 MHz, $CDCl_3$) δ -62.27 (s); **LRMS** (ESI$^+$) *m/z* 374.0 [M+Na]$^+$; **HRMS** (ESI$^+$) found *m/z* 352.0826 [M+H]$^+$, $C_{14}H_{17}NO_4F_3S$ requires m/z 352.0825; **IR** ν_{max} (neat)/cm^{-1} 2940, 1733, 1601, 1327, 1117; **R$_f$** = 0.20 (5% EtOAc in Petrol).

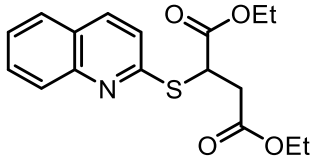

diethyl 2-(quinolin-2-ylthio)succinate (*pre*-5d)

Following General procedure **A**, quinoline-2-thiol (0.483 g, 3.00 mmol, 1.0 equiv.), diethyl fumarate (2.50 mL, 15.0 mmol, 5.0 equiv.) and Et_3N (0.40 mL, 3.00 mmol, 1.0 equiv.) in acetonitrile (4.5 mL, 0.7 M) were heated at 40 °C for 24 h. The concentrated crude residue was purified by flash column chromatography (1-10% EtOAc in Petrol) to give the title compound as a yellow oil (844 mg, 84%).

^{1}**H NMR** (400 MHz, $CDCl_3$) δ 7.93 (d, *J* = 8.5 Hz, 2H, Ar-*H*), 7.73 (d, *J* = 8.5 Hz, 1H, Ar-*H*), 7.65 (t, *J* = 7.5 Hz, 1H, Ar-*H*), 7.45 (t, *J* = 7.5 Hz, 1H, Ar-*H*), 7.21 (d, *J* = 9.0 Hz, 1H, Ar-*H*), 5.18 (dd, *J* = 8.0, 5.5 Hz, 1H, SC*H*), 4.22 (q, *J* = 7.0 Hz, 2H, OC*H₂*), 4.18 (q, *J* = 7.5 Hz, 2H, OC*H₂*), 3.25 (dd, *J* = 17.0, 8.0 Hz, 1H, SCHC*H₂*), 3.11 (dd, *J* = 17.0, 5.5 Hz, 1H, SCHC*H₂*), 1.30 – 1.19 (m, 6H, 2 × CH_2CH_3); ^{13}**C NMR** (101 MHz, $CDCl_3$) δ 171.2, 171.2, 156.6, 148.1, 136.1, 130.0, 128.1, 127.9, 126.3, 125.8, 120.6, 61.9, 61.0, 42.0, 37.3, 14.3, 14.2; **LRMS** (ESI^+) *m/z* 334.0 $[M+H]^+$; **HRMS** (ESI^+) found *m/z* 334.1105 $[M+H]^+$, $C_{17}H_{20}NO_4S$ requires m/z 334.1108; **IR** ν_{max} (neat)/cm^{-1} 2982, 2937, 1731, 1593, 1137, 1089, 1029, 818; $\mathbf{R_f}$ = 0.18 (5% EtOAc in Petrol).

3.2.b Synthesis of heteroaromatic sulfones (latent sulfinates)

General Procedure C:

Open to air: To a stirring solution of pyridine thioether (1.0 equiv.) in ethanol (0.7 M) was added $Na_2WO_4\cdot 2H_2O$ (5.0 mol% or 10 mol%). A solution of hydrogen peroxide (30% w/w in water) (5.0 or 10.0 equiv.) was then added slowly as an exotherm may be observed. The reaction was stirred at RT overnight and the formation of the sulfoxide and sulfone were monitored by TLC analysis. After 16 h, three volumes of water relative to the reaction solvent (ethanol) was added to the reaction mixture and left to stir for 30 min. If a solid precipitated out of the reaction mixture on addition of water, the solid was collected by filtration, washed with copious amounts of water and dried *in vacuo* to give the product. If no precipitate was formed, then the solvent was removed under reduced pressure and the product was extracted from the aqueous layer with EtOAc thrice. The combined organic layers were washed with brine, dried over $MgSO_4$ and concentrated *in vacuo.* The crude residue was then purified by flash column chromatography on silica gel (EtOAc in Petrol).

General Procedure D:

Open to air: To a solution of pyridine sulfinate (1.1 equiv.) in de-ionised water (0.4 M) was added acetic acid (1.1 equiv.) and either acrylonitrile (1.0 equiv.) or methyl acrylate (1.0 equiv.). The reaction mixture was then heated to reflux (115 °C) and monitored by TLC analysis. Upon completion, the reaction mixture was then cooled to RT and solid $NaHCO_3$ was added until the reaction mixture was ≈ pH 8. The aqueous layer was extracted with EtOAc (× 4). The combined organic layers were washed with brine, dried over $MgSO_4$ and concentrated *in vacuo*. The crude residue was then purified by flash column chromatography (EtOAc in Petrol) to give the desired product.

General procedure E: (Adapted from the literature procedure reported by *Wang* et. al[5])

Sodium 3-methoxy-3-oxopropane-1-sulfinate (SMOPS reagent) was prepared according to Baskin and Wang,[5] or obtained from commercial sources.

Under N_2: Iodopyridine *or* bromopyridine (1.0 equiv.) was added to a solution of CuI (3.0 -5.0 equiv.) and sodium 3-methoxy-3-oxopropane-1-sulfinate (SMOPS reagent) (3.0 -5.0 equiv.) in DMSO (0.4 M). The reaction was stirred under nitrogen atmosphere at 110 °C for 16 h. The mixture was then cooled to RT, diluted with EtOAc and filtered through a pad of silica. The filtrate was washed with H_2O (× 2), brine, dried over $MgSO_4$ and concentrated *in vacuo*. The crude product was then purified by flash column chromatography (EtOAc in Petrol) to give the desired sulfone product.

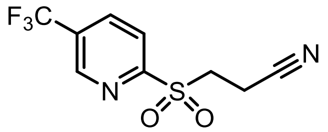

3-((5-(trifluoromethyl)pyridin-2-yl)sulfonyl)propanenitrile (1a)

Following general procedure **C,** 3-((5-(trifluoromethyl)pyridin-2-yl)thio)propanenitrile (4.00 g, 17.2 mmol, 1.0 equiv.), $Na_2WO_4 \cdot 2H_2O$ (284 mg, 0.860 mmol, 5.0 mol%) and hydrogen peroxide solution (30% w/w in water) (8.80 mL, 86.1 mmol, 5.0 equiv.) in ethanol (25.0 mL, 0.7 M) was stirred at RT for 16 h. On diluting, a white solid precipitated out of the reaction mixture. The solid was collected by filtration, washed with copious amounts of water and dried *in vacuo.* The corresponding white solid was purified through hot recrystalisation using hexanes:EtOAc to give the title compound **1a** as iridescent flaky white crystals (3.87 g, 85%).

^{1}H NMR (400 MHz, $CDCl_3$) δ 9.03 – 9.02 (m, 1H, Ar-*H*), 8.30-8.25 (m, 2H, Ar-*H*), 3.79 (t, *J* = 7.5 Hz, 2H, SO_2CH_2), 2.98 (t, *J* = 7.5 Hz, 2H, C*H*$_2$CN); **^{13}C NMR** (101 MHz, $CDCl_3$) δ 159.6, 147.5 (q, $^3J_{C\text{-}F}$ = 4.0 Hz), 136.4 (q, $^3J_{C\text{-}F}$ = 3.5 Hz), 130.9 (q, $^2J_{C\text{-}F}$ = 35.0 Hz), 122.5 (q, $^1J_{C\text{-}F}$ = 274.0 Hz), 122.5, 115.9, 47.1, 12.3; **^{19}F NMR** (377 MHz, $CDCl_3$) δ -62.68 (s); **HRMS** (ESI$^+$) found *m/z* 287.0074 $[M+Na]^+$, $C_9H_7O_2N_2F_3NaS$ requires m/z 287.0073; **IR** ν_{max} (neat)/cm^{-1} 3109, 3057, 2986, 2927, 2257, 1591, 1578, 1408, 1386, 1318 (SO_2), 1148 (SO_2), 1100, 1013, 867, 726, 700; **mp** 118-120 °C (hexanes:EtOAc); **R$_f$** = 0.42 (40% EtOAc in Petrol).

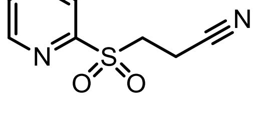

3-(pyridin-2-ylsulfonyl)propanenitrile (1b)

Following general procedure **C,** 3-(pyridin-2-ylthio)propanenitrile (644 mg, 3.92 mmol, 1.0 equiv.), $Na_2WO_4 \cdot 2H_2O$ (64.7 mg, 0.20 mmol, 5.0 mol%) and hydrogen peroxide solution (30% w/w in water) (2.00 mL, 19.6 mmol, 5.0 equiv.) in ethanol (6.0 mL, 0.7 M) was stirred at RT for 16 h. On diluting, no precipitate was formed and the reaction was worked up accordingly.

The crude yellow oil was purified by flash column chromatography (50% EtOAc in Petrol) to give the title compound as an off-white solid (620 mg, 81%).

Following general procedure **D**, sodium pyridine-2-sulfinate (182 mg, 1.10 mmol, 1.1 equiv.), acrylonitrile (66.3 µL, 1.00 mmol, 1.0 equiv.) and AcOH (57.2 µL, 1.00 mmol, 1.0 equiv.) were refluxed in water (3 mL, 0.33 M) for 2.5 h. After workup, the crude residue was purified by flash column chromatography (60% EtOAc in Petrol) to give the title compound as a crystalline white solid (163 mg, 83%).

^{1}H NMR (400 MHz, $CDCl_3$) δ 8.76 (ddd, *J* = 4.5, 1.5, 1.0 Hz, 1H, Ar-*H*), 8.11 (dt, *J* = 8.0, 1.0 Hz, 1H, Ar-*H*), 8.02 (td, *J* = 8.0, 1.5 Hz, 1H, Ar-*H*), 7.62 (ddd, *J* = 7.5, 4.5, 1.0 Hz, 1H, Ar-*H*), 3.73 (t, *J* = 7.5 Hz, 2H, SO_2CH_2), 2.95 (t, *J* = 7.5 Hz, 2H, CH_2CN); **^{13}C NMR** (101 MHz, CDCl3) δ 156.5, 150.5, 138.8, 128.3, 122.4, 116.1, 47.2, 12.3**;** **LRMS** (ESI^+) *m/z* 197.0 $[M+H]^+$; **HRMS** (ESI^+) found *m/z* 197.0381 $[M+H]^+$, $C_8H_9O_2N_2S$ requires m/z 197.0379; **IR** ν_{max} (neat)/cm^{-1} 3061, 3001, 2969, 2953, 2250, 1581, 1453, 1435, 1313 (SO_2), 1292, 1151 (SO_2), 1109, 993, 957, 902, 793, 747, 692; **mp** 50-51 °C (EtOAc:Petrol); **R_f** = 0.27 (60% EtOAc in Petrol).

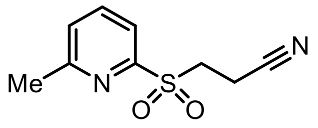

3-((6-methylpyridin-2-yl)sulfonyl)propanenitrile (1c)

Following general procedure **C,** 3-((6-methylpyridin-2-yl)thio)propanenitrile (290 mg, 1.63 mmol, 1.0 equiv.), $Na_2WO_4 \cdot 2H_2O$ (27.0 mg, 0.08 mmol, 5.0 mol%) and hydrogen peroxide solution (30% w/w in water) (890 µL, 8.70 mmol, 5.3 equiv.) in ethanol (2.5 mL, 0.7 M) was stirred at RT for 16 h. On diluting with water, a white solid precipitated out of the reaction mixture. The white solid was collected by filtration, washed with copious amounts of water and dried *in vacuo* to give the title compound as a white solid (121 mg, 35%).

^{1}H NMR (400 MHz, $CDCl_3$) δ 7.93 – 7.85 (m, 2H, Ar-*H*), 7.46 (dd, *J*= 7.5, 1.5 Hz, 1H, Ar-*H*), 3.72 (t, *J* =7.5 Hz, 2H, SO_2CH_2), 2.95 (t, *J* = 7.5 Hz, 2H, CH_2CN), 2.67 (s, 3H, CH_3); **^{13}C NMR** (101 MHz, $CDCl_3$) δ 160.7, 155.6, 138.6, 128.2, 119.5, 116.3, 47.0, 24.5, 12.3; **LRMS** (ESI^+) *m/z* 211.0 (65%, $[M+H]^+$), 232.9 (35%, $[M+Na]^+$); **HRMS** (ESI^+) found *m/z* 211.0538 $[M+H]^+$, $C_9H_{11}O_2N_2S$ requires m/z 211.0536; **IR** ν_{max} (neat)/ cm^{-1} 2962, 2361, 2252, 1593, 1557, 1454, 1420, 1313, 1254, 1177, 1149, 1117, 1087, 1036, 986, 956, 868, 798, 736, 717, 669; **mp** 68-70 °C ($EtOH:H_2O$); **R_f** = 0.18 (50% EtOAc in Petrol).

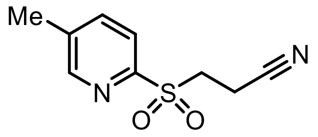

3-((5-methylpyridin-2-yl)sulfonyl)propanenitrile (1d)

Following general procedure **C,** 3-((5-methylpyridin-2-yl)thio)propanenitrile (201 mg, 1.13 mmol, 1.0 equiv.), $Na_2WO_4 \cdot 2H_2O$ (18.6 mg, 0.056 mmol, 5.0 mol%.) and hydrogen peroxide solution (30% w/w in water) (576 µL, 5.64 mmol, 5.0 equiv.) in ethanol (2.0 mL, 0.6 M) was stirred at RT for 20 h, then diluted with water. No precipitate was observed so the reaction mixture was extracted with EtOAc (20.0 mL × 3). The combined organic layers were then washed with brine (10 mL), dried over $MgSO_4$ and concentrated *in vacuo*. The product was then purified by flash column chromatography (70% EtOAc in Petrol) to give the title compound as a crystalline white solid (187 mg, 79%).

^{1}H NMR (400 MHz, $CDCl_3$) δ 8.57 (app. dt, *J* = 2.0, 1.0 Hz, 1H, Ar-*H*), 7.99 (d, *J* = 8.0 Hz, 1H, Ar-*H*), 7.79 (app. ddd, *J* = 8.0, 2.5, 1.0 Hz, 1H, Ar-*H*), 3.69 (t, *J* = 8.0, 2H, SO_2CH_2), 2.92 (t, *J* = 8.0 Hz, 2H, CH_2CN), 2.48 (s, 3H, CH_3); **^{13}C NMR** (101 MHz, $CDCl_3$) δ 153.6, 151.1, 139.3, 138.8, 122.2, 116.2, 47.4, 18.8, 12.3; **LRMS** (ESI$^+$) *m/z* 211.0 (60%, $[M+H]^+$), 233.0 (40%, $[M+Na]^+$); **HRMS** (ESI$^+$) found *m/z* 211.0536 $[M+H]^+$, $C_9H_{11}O_2N_2S$ requires m/z 211.0536; **IR** ν_{max} (neat)/cm^{-1} 2939, 2253, 1574, 1458, 1419, 1374, 1315, 1241, 1213, 1163, 1150, 1131, 1099, 1027, 956, 838, 782, 747, 712, 663, 644, 622; **mp** 66 °C (EtOAc); **R_f** = 0.11 (65% EtOAc in Petrol).

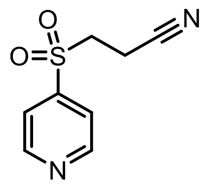

3-(pyridin-4-ylsulfonyl)propanenitrile (1e)

Following general procedure **C,** 3-(pyridin-4-ylthio)propanenitrile (320 mg, 1.95 mmol, 1.0 equiv.), $Na_2WO_4 \cdot 2H_2O$ (64.6 mg, 0.20 mmol, 10 mol%) and hydrogen peroxide solution (30% w/w in water) (1.00 mL, 9.79 mmol, 5.0 equiv.) in ethanol (3.0 mL, 0.7 M) was stirred at RT for 16 h, and then diluted with water. No precipitate was observed so the reaction mixture was extracted with EtOAc (20 mL × 4). The combined organic layers were then washed with brine (50 mL), dried over $MgSO_4$ and concentrated *in vacuo* to give the title compound as a white crystalline solid (306 mg, 80%). The compound was pure by ^{1}H NMR analysis and so was used without further purification.

^{1}H NMR (400 MHz, $CDCl_3$) δ 9.02 – 8.95 (m, 2H, Ar-*H*), 7.84 – 7.79 (m, 2H, Ar-*H*), 3.43 (t, *J* = 7.5 Hz, 2H, SO_2CH_2), 2.88 (t, *J* = 7.5 Hz, 2H, CH_2CN); **^{13}C NMR** (101 MHz, $CDCl_3$) δ 151.9, 145.9, 121.3, 115.6, 50.9, 11.9; **LRMS** (ESI$^+$) *m/z* 197.0 $[M+H]^+$; **HRMS** (ESI$^+$) found *m/z* 197.0380 $[M+H]^+$, $C_8H_9O_2N_2S$ requires m/z 197.0379; **IR** v_{max} (neat)/cm^{-1} 3091, 2974, 2932, 2915, 2361, 2341, 2260, 1576, 1563, 1427, 1406, 1318, 1218, 1173, 1139, 1099, 1069, 966, 823, 751, 726, 684; **mp** 80-82 °C (EtOAc); **R_f** = 0.25 (100% EtOAc).

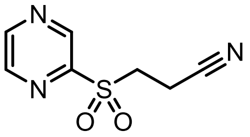

3-(pyrazin-2-ylsulfonyl)propanenitrile (1f)

Following general procedure **C,** 3-(pyrazin-2-ylthio)propanenitrile (300 mg, 1.82 mmol, 1.0 equiv.), $Na_2WO_4 \cdot 2H_2O$ (30 mg, 0.09 mmol, 5.0 mol%) and hydrogen peroxide solution (30% w/w in water) (930 µL, 9.1 mmol, 5.0 equiv.) in ethanol (3.0 mL, 0.7 M) was stirred at RT for 16 h. On diluting with water, a white solid precipitated out of the reaction mixture. The white solid was collected by filtration, washed with copious amounts of water and dried *in vacuo*. The filtrate was further extracted with EtOAc and washed with water. The organic phase was then dried over $MgSO_4$ and concentrated *in vacuo* to give a solid. Both solids from precipitation and extraction were combined to give the title compound as a white solid (252 mg, 70%) without need for further purification.

^{1}H NMR (400 MHz, $CDCl_3$) δ 9.33 (d, *J* = 1.5 Hz, 1H, Ar-*H*), 8.95 (d, *J* = 2.5 Hz, 1H, Ar-*H*), 8.75 (dd, *J* = 2.5, 1.5 Hz, 1H, Ar-*H*), 3.74 (t, *J* = 7.5 Hz, 2H, SO_2CH_2), 2.98 (t, *J* = 7.5 Hz, 3H, CH_2-CN); **^{13}C NMR** (101 MHz, $CDCl_3$) δ 152.3, 149.3, 144.6, 143.5, 115.8, 47.6, 12.2; **LRMS** and **HRMS** (ESI^+) not found; **IR** v_{max} (neat)/cm^{-1} 2981, 2891, 1403, 1310, 1294, 1151, 1114, 958, 777, 712; **mp** 118-122 °C (CH_2Cl_2).

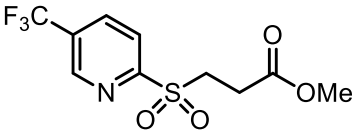

methyl 3-((5-(trifluoromethyl)pyridin-2-yl)sulfonyl)propanoate (4a)

Following general procedure **C,** methyl 3-((5-(trifluoromethyl)pyridin-2-yl)thio)propanoate (974 mg, 3.67 mmol, 1.0 equiv.), $Na_2WO_4 \cdot 2H_2O$ (60.5 mg, 0.18 mmol, 5.0 mol%.) and hydrogen peroxide solution (30% w/w in water) (1.88 mL, 18.4 mmol, 5.0 equiv.) in ethanol (5.2 mL, 0.7 M) was stirred at RT for 16 h. On diluting with water, a white solid precipitated out of the reaction mixture. The white solid was collected by filtration, washed with copious amounts of water and dried *in vacuo* to give the title compound as a crystalline white solid (989 mg, 91%). *When scaled up to a 15 mmol scale, gave a 90% yield.*

Alternative Synthesis: Following general procedure **E**, 2-bromo-5-(trifluoromethyl)pyridine (226 mg, 1.00 mmol, 1.0 equiv.), SMOPS (871 mg, 5.00 mmol, 5.0 equiv.) and CuI (952 mg, 5.00 mmol, 5.0 equiv.) were heated to 110 °C in DMSO (2.5 mL, 0.4 M) for 16h. The crude product was purified by flash column chromatography (20% EtOAc in Petrol) to give the title compound as a white crystalline solid (75.7 mg, 25%).

^{1}H NMR (400 MHz, $CDCl_3$) δ 8.99 – 8.98 (m, 1H, Ar-*H*), 8.26 – 8.21 (m, 2H, Ar-*H*), 3.81 – 3.76 (t, *J* = 7.5 Hz, 2H, SO_2CH_2), 3.69 (s, 3H, $COOCH_3$), 2.87 (t, *J* = 7.5 Hz, 2H, CH_2COOMe); **^{13}C NMR** (101 MHz, $CDCl_3$) δ 170.4, 160.3, 147.4 (q, $^{3}J_{C-F}$ = 4.0 Hz), 136.1 (q, $^{3}J_{C-F}$ = 3.5 Hz), 130.3 (q, $^{2}J_{C-F}$ = 34.5 Hz), 122.6 (q, $^{1}J_{C-F}$ = 273.0 Hz), 122.1, 53.2, 47.6, 27.4; **^{19}F NMR** (377 MHz, $CDCl_3$) δ -62.68 (s); **LRMS** (ESI^+) *m/z* 320.0 $[M+Na]^+$; **HRMS** (ESI^+) found *m/z* 298.0356 $[M+H]^+$, $C_{10}H_{11}O_4NF_3S$ requires m/z 298.0355; **IR** v_{max} (neat)/cm^{-1} 3061, 2956, 2360, 1744, 1732,1594, 1578, 1325, 1149, 1099, 1073, 1014, 830; **mp** 75-76 °C (EtOH:H_2O); **R$_f$** = 0.55 (60% EtOAc in Petrol).

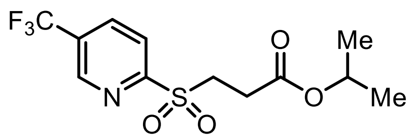

***Iso*-propyl 3-((5-(trifluoromethyl)pyridin-2-yl)sulfonyl)propanoate (4a-iPr)**

Following general procedure **C,** *iso*-propyl 3-((5-(trifluoromethyl)pyridin-2-yl)thio)propanoate (61.6 mg, 0.21 mmol, 1.0 equiv.), $Na_2WO_4 \cdot 2H_2O$ (3.5 mg, 0.01 mmol, 5.0 mol%) and hydrogen peroxide solution (30% w/w in water) (107 µL, 1.05 mmol, 5.0 equiv.) in ethanol (300 µL, 0.7 M) was stirred at RT for 19 h. On diluting with water, a white solid precipitated out of the reaction mixture. The white solid was collected by filtration, washed with copious amounts of water and dried *in vacuo* to give the title compound as a bright white solid (44.4 mg, 65%).

Alternative synthesis:

3-((5-(trifluoromethyl)pyridin-2-yl)sulfonyl)propanoic acid **4a-OH** (30 mg, 0.10 mmol, 1.0 equiv.) was dissolved in degassed *iso*-propanol (5.0 mL, 0.02 M) and conc. H_2SO_4 (1 drop) was added. The resultant cloudy suspension was heated to reflux for 4 h and then cooled to RT. The reaction mixture was then concentrated *in vacuo* to remove the solvent. The residue was dissolved in CH_2Cl_2 and poured into a separating funnel with water. The aqueous layer was basified with solid $NaHCO_3$ until it was *ca.* pH 8. The aqueous layer was washed with CH_2Cl_2 (15 mL × 3). The combined organic layers were dried over $MgSO_4$, concentrated *in vacuo* and purified by flash column chromatography (2-10% EtOAc in Petrol) to give the title compound as a crystalline white solid (26.6 mg, 82%).

^{1}H NMR (400 MHz, $CDCl_3$) δ 9.00 (s, 1H, Ar-*H*), 8.25 – 8.22 (m, 2H, Ar-*H*), 4.99 (hept, *J* = 6.5 Hz, 1H, C*H*(CH_3)$_2$), 3.77 (t, *J* = 8.0 Hz, 2H, SO_2CH_2), 2.82 (t, *J* = 8.0 Hz, 2H, C*H*$_2$CO), 1.23 (d, *J* = 6.5 Hz, 6H, CH(C*H*$_3$)$_2$); **^{13}C NMR** (101 MHz, $CDCl_3$) δ 169.4, 160.4, 147.4 (q, $^3J_{C-F}$ = 3.5 Hz), 136.0 (q, $^3J_{C-F}$ = 3.5 Hz), 130.3 (q, $^2J_{C-F}$ = 34.0 Hz), 122.6 (q, $^1J_{C-F}$ = 273.5 Hz), 122.1, 69.4, 47.7, 27.9, 21.8; **^{19}F NMR** (377 MHz, $CDCl_3$) δ -62.69 (s); **HRMS** (ESI$^+$) found *m/z* 326.0671 $[M+H]^+$, $C_{12}H_{15}O_4NF_3S$ requires m/z 326.0668; **IR** ν_{max} (neat)/cm^{-1} 3104, 3061, 2983, 2933, 2361, 1726, 1323, 1254, 1238, 1162, 1142, 1127, 1101, 1073; **mp** 52-54 °C (EtOH:H_2O); **R$_f$** = 0.44 (30% EtOAc in Petrol).

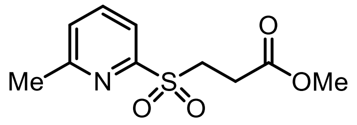

Methyl 3-((6-methylpyridin-2-yl)sulfonyl)propanoate (4b)

Following General procedure **C**, methyl 3-((6-methylpyridin-2-yl)thio)propanoate (317 mg, 1.50 mmol, 1.0 equiv.), $Na_2WO_4 \cdot 2H_2O$ (25 mg, 0.075 mmol, 5.0 mol%) and hydrogen peroxide solution (30% w/w in water) (765 mL, 7.50 mmol, 5.0 equiv.) in ethanol (2.25 mL, 0.7 M) were stirred at RT for 16 h, then diluted with water. No precipitate was observed so the reaction mixture was extracted with EtOAc (20 mL × 4). The combined organic layers were then washed with brine (50 mL), dried over $MgSO_4$ and concentrated *in vacuo.* The crude product was purified by flash column chromatography (30-50% EtOAc in Petrol) to give the title compound as a colourless oil (354 mg, 97%).

Alternative Procedure:

Following General procedure **E**, sodium 3-methoxy-3-oxopropane-1-sulfinate (SMOPS reagent, 522 mg, 3.00 mmol, 3.0 equiv.), CuI (570 mg, 3.00 mmol, 3.0 equiv.) and 2-bromo-6-methylpyridine (114 µL, 1.00 mmol, 1.0 equiv.) in DMSO (2.0 mL, 0.5 M) were heated at 110 °C for 16 h. The crude product was purified by flash column chromatography (2% EtOAc in CH_2Cl_2 to give the title compound as a colourless oil (140 mg, 58%).

^{1}H NMR (400 MHz, $CDCl_3$) δ 7.89 (d, *J* = 7.5 Hz, 1H, Ar-*H*), 7.83 (app. t, *J* = 7.5 Hz, 1H, Ar-*H*), 7.40 (dd, *J* = 7.5, 1.0 Hz, 1H, Ar-*H*), 3.72 (t, *J* = 8.0 Hz, 2H, $SO_2CH_2CH_2$), 3.69 (s, 3H, OCH_3), 2.85 (t, *J* = 8.0 Hz, 2H, CH_2CO_2Me), 2.65 (s, 3H, Ar-CH_3); **^{13}C NMR** (101 MHz, $CDCl_3$) δ 170.8, 160.4, 156.3, 138.3, 127.6, 119.4, 52.4, 47.6, 27.5, 24.5; **LRMS** (ESI$^+$) *m/z* 266.0 $[M+Na]^+$; **HRMS** (ESI$^+$) found *m/z* 266.0457 $[M+Na]^+$, $C_{10}H_{13}NO_4SNa$ requires m/z 266.0457; **IR** ν_{max} (neat)/cm^{-1} 2981, 2956, 1738, 1593, 1453, 1314, 1117 ; **R_f** = 0.33 (5% EtOAc in CH_2Cl_2); **R_f** = 0.29 (50% EtOAc in Petrol).

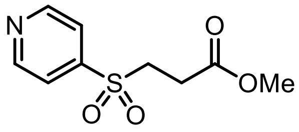

methyl 3-(pyridin-4-ylsulfonyl)propanoate (4c)

Following General procedure **C**, methyl 3-(pyridin-4-ylthio)propanoate (160 mg, 0.810 mmol, 1.0 equiv.), $Na_2WO_4 \cdot 2H_2O$ (13 mg, 0.039 mmol, 5.0 mol%) and hydrogen peroxide solution (30% w/w in water) (0.4 mL, 4.00 mmol, 5.0 equiv.) in ethanol (1.2 mL, 0.7 M) were stirred at RT for 16 h. On diluting with water, a white solid precipitated out of the reaction mixture. The white solid was collected by filtration. The crude product was purified by flash column chromatography (50-70% EtOAc in Petrol) to give the title compound as a white solid (154 mg, 84%).

This 2 step synthetic method to reach **4c** was also tried out as a one-pot sequence, in which the crude mixture of the methyl 3-(pyridin-4-ylthio)propanoate ***pre*-4c** was simply concentrated and used directly for the Noyori oxidation step without further purification. In this case, the final product crashed out when diluting with water, yielding, after filtration and drying, pure white crystals (2.30 g, 42%). This one-pot sequence allows for faster access to the desired product with a better yield and no need for column chromatography at all.

^{1}H NMR (400 MHz, $CDCl_3$) δ 8.89 (dd, *J* = 4.5, 1.5 Hz, 2H, Ar-*H*), 7.74 (dd, *J* = 4.5, 1.5 Hz, 2H, Ar-*H*), 3.61 (s, 3H, OC*H$_3$*), 3.45 (t, *J* = 7.5 Hz, 2H, SC*H$_2$*CH$_2$), 2.75 (t, *J* = 7.5 Hz, 2H, SCH$_2$C*H$_2$*); **^{13}C NMR** (101 MHz, $CDCl_3$) δ 170.1, 151.5, 146.7, 121.2, 52.5, 51.1, 27.3; **LRMS** (ESI$^+$) *m/z* 230.0 [M+H]$^+$; **HRMS** (ESI$^+$) found *m/z* 230.0482 [M+H]$^+$, $C_9H_{12}NO_4S$ requires m/z 230.0482; **IR** ν_{max} (neat)/cm^{-1} 3095, 2936, 1732, 1301, 1266, 1196, 1161, 1096, 984, 801; **mp** 96-98 °C (CH_2Cl_2); **R$_f$** = 0.26 (70% EtOAc in Petrol).

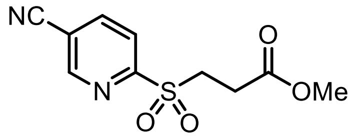

methyl 3-((5-cyanopyridin-2-yl)sulfonyl)propanoate (4d)

Following General procedure **B**, 6-chloronicotinonitrile (693 mg, 5.00 mmol, 1.0 equiv.), K_2CO_3 (1.037 g, 7.50 mmol, 1.5 equiv.) and methyl-3-mercaptopropionate (830 µL, 7.50 mmol, 1.5 equiv.) in DMF (5.0 mL, 1.0 M) were heated at 45 °C for 16 h. After extraction, the crude product was submitted to the oxidation step without further purification.

Following General procedure **C**, the previous crude product methyl 3-((5-cyanopyridin-2-yl)thio)propanoate (5.0 mmol, 1.0 equiv.), $Na_2WO_4 \cdot 2H_2O$ (82.5 mg, 0.250 mmol, 5.0 mol%) and hydrogen peroxide solution (30% w/w in water) (2.5 mL, 25.0 mmol, 5.0 equiv.) in ethanol (7.5 mL, 0.7 M) were stirred at RT for 16 h. On diluting with water, a white solid precipitated out of the reaction mixture. The white solid was collected by filtration and purified by flash column chromatography (10-30% EtOAc in Petrol) to give the title compound as a white solid (330 mg, 26% over two steps).

^{1}H NMR (400 MHz, $CDCl_3$) δ 8.98 (dd, *J* = 2.0, 1.0 Hz, 1H, Ar-*H*), 8.28 (dd, *J* = 8.0, 2.0 Hz, 1H, Ar-*H*), 8.21 (dd, *J* = 8.0, 1.0 Hz, 1H, Ar-*H*), 3.78 (t, *J* = 7.5 Hz, 2H, SC*H$_2$*CH$_2$), 3.69 (s, 3H, OC*H$_3$*), 2.88 (d, *J* = 7.5 Hz, 2H, C*H$_2$*CO$_2$Me); **^{13}C NMR** (101 MHz, $CDCl_3$) δ 170.4, 160.1, 152.6, 142.1, 122.0, 115.1, 113.9, 52.6, 47.6, 27.4; **HRMS** (ESI$^+$) found *m/z* 277.0254 [M+Na]$^+$, $C_{10}H_{10}N_2O_4SNa$ requires m/z 277.0254; **IR** ν_{max} (neat)/cm^{-1} 3093, 2957, 1735, 1320, 1252, 1158, 1099; **mp** 90-92 °C (CH_2Cl_2); **R$_f$** = 0.21 (30% EtOAc in Petrol).

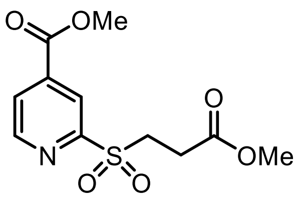

methyl 2-((3-methoxy-3-oxopropyl)sulfonyl)isonicotinate (4e)

Following general procedure **C**, methyl 2-((3-methoxy-3-oxopropyl)thio)isonicotinate (735.8 mg, 2.88 mmol, 1.0 equiv.), $Na_2WO_4{\cdot}2H_2O$ (47.5 mg, 0.140 mmol, 5.0 mol%.) and hydrogen peroxide solution (30% w/w in water) (1.47 mL, 14.4 mmol, 5.0 equiv.) in ethanol (4.1 mL, 0.7 M) was stirred at RT for 24 h, then diluted with water. No precipitate was observed so the reaction mixture was extracted with EtOAc (× 4). The combined organic layers were then washed with brine, dried over $MgSO_4$ and concentrated *in vacuo*. The crude product was then purified by flash column chromatography (35-40% EtOAc in Petrol). However the product co-eluted with the sulfoxide. The mixed fractions were combined, concentrated *in vacuo* and dissolved in ethanol (3.0 mL, 0.7 M). To this stirring solution was added $Na_2WO_4 \cdot 2H_2O$ (33.0 mg, 0.10 mmol, 5.0 mol%.) and hydrogen peroxide (30% w/w water) solution (1.0 mL, 10.0 mmol, 5.0 equiv.) and the reaction mixture was stirred at RT for 16 h. Upon addition of water (12 mL), a white precipitate formed. This precipitate was collected and purified by flash column chromatography (30% EtOAc in Petrol) to give the title compound as a white solid (191 mg, 23%).

^{1}H NMR (400 MHz, $CDCl_3$) δ 9.29 (dd, *J* = 2.0, 1.0 Hz, 1H, Ar-*H*), 8.57 (dd, *J* = 8.0, 2.0 Hz, 1H, Ar-*H*), 8.16 (dd, *J* = 8.0, 1.0 Hz, 1H, Ar-*H*), 4.01 (s, 3H, CO_2*Me*), 3.77 (t, *J* = 8.0 Hz, 2H, SO_2CH_2), 3.69 (s, 3H, CO_2*Me*), 2.86 (t, *J* = 8.0 Hz, 2H, CH_2CO_2Me); **^{13}C NMR** (101 MHz, $CDCl_3$) δ 170.5, 164.2, 160.2, 151.3, 139.7, 129.5, 121.8, 53.2, 52.5, 47.6, 27.5; **LRMS** (ESI$^+$) *m/z* 310.0 $[M+Na]^+$; **HRMS** (ESI$^+$) found *m/z* 288.0537 $[M+H]^+$, $C_{11}H_{14}O_6NS$ requires m/z 288.0536; **IR** ν_{max}(neat)/cm^{-1} 3736, 3622, 2924, 2855, 2361, 1733, 1459, 1371, 1296, 1165, 1122, 1023, 680; **mp** 104-105 °C (EtOAc)**;** **R$_f$** = 0.22 (40% EtOAc in Petrol).

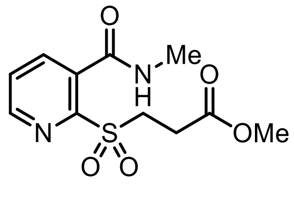

methyl 3-((3-(methylcarbamoyl)pyridin-2-yl)sulfonyl)propanoate (4f)

Following general procedure **C,** methyl 3-((3-(methylcarbamoyl)pyridin-2-yl)thio)propanoate (400 mg, 1.57 mmol, 1.0 equiv.), $Na_2WO_4 \cdot 2H_2O$ (26.4 mg, 0.08 mmol, 5.0 mol%) hydrogen peroxide solution (30% w/w water) (1.60 mL, 15.7 mmol, 10.0 equiv.) in ethanol (2.5 mL, 0.6 M) was stirred at RT for 18 h, then diluted with water. No precipitate was observed so the

reaction mixture was extracted with EtOAc (20 mL × 4). The combined organic layers were then washed with brine (10 mL), dried over $MgSO_4$ and concentrated *in vacuo*. The crude product was then purified by flash column chromatography (100% EtOAc) to give the title compound as a colourless oil that crystallised on standing to give a white solid (207 mg, 46%).

^{1}H NMR (400 MHz, $CDCl_3$) δ 8.66 (dd, *J* = 4.5, 2.0 Hz, 1H, Ar-*H*), 8.07 (dd, *J* = 8.0, 1.5 Hz, 1H, Ar-*H*), 7.59 (dd, *J* = 8.0, 5.0 Hz, 1H, Ar-*H*), 6.69 (br. s, 1H, N*H*), 3.92 (t, *J* = 7.5 Hz, 2H, SO_2CH_3), 3.69 (s, 3H, CO_2CH_3), 3.01 (d, *J* = 5.0 Hz, 3H, $NHCH_3$), 2.90 (t, *J* = 7.5 Hz, 2H, CH_2CO_2Me); **^{13}C NMR** (101 MHz, $CDCl_3$) δ 170.7, 165.5, 154.0, 149.7, 139.5, 132.3, 127.5, 52.5, 48.4, 27.7, 27.3; **LRMS** (ESI$^+$) *m/z* 309.0 $[M+Na]^+$; **HRMS** (ESI$^+$) found *m/z* 287.0698 $[M+H]^+$, $C_{11}H_{15}O_5N_2S$ requires m/z 287.0696; **IR** v_{max} (neat)/cm^{-1} 3369, 3291, 3075, 2954, 2925, 2853, 1737, 1654, 1584, 1554, 1441, 1407, 1365, 1318, 1254, 1179, 1157, 1121, 1085, 1058, 979, 841, 767, 749, 707, 663, 524; **mp** 105-107 °C (EtOAc); **R_f** = 0.12 (100% EtOAc).

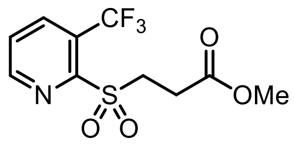

methyl 3-((3-(trifluoromethyl)pyridin-2-yl)sulfonyl)propanoate (4g)

Following general procedure **C,** methyl 3-((3-(trifluoromethyl)pyridin-2-yl)thio)propanoate (898.5 mg, 3.39 mmol, 1.0 equiv.), $Na_2WO_4 \cdot 2H_2O$ (112 mg, 0.34 mmol, 10 mol%), hydrogen peroxide solution (30% w/w water) (3.46 mL, 33.9 mmol, 10.0 equiv.) in ethanol (5.0 mL, 0.6 M) was stirred at RT for 18 h, then diluted with water. No precipitate was observed so the reaction mixture was extracted with EtOAc (× 4). The combined organic layers were then washed with brine, dried over $MgSO_4$ and concentrated *in vacuo*. The crude product was then purified by flash column chromatography (50% EtOAc in Petrol) to give the title compound as a pale yellow oil (310 mg, 31%).

^{1}H NMR (400 MHz, $CDCl_3$) δ 8.82 (dd, *J* = 5.0, 1.5 Hz, 1H, Ar-*H*), 8.26 (dd, *J* = 8.0, 1.5 Hz, 1H, Ar-*H*), 7.71 (dd, *J* = 8.0, 5.0 Hz, 1H, Ar-*H*), 3.98 (t, *J* = 7.5 Hz, 2H, SO_2CH_2), 3.72 (s, 3H, OCH_3), 2.95 (t, *J* = 7.5 Hz, 2H, CH_2CO_2Me); **^{13}C NMR** (101 MHz, $CDCl_3$) δ 170.8, 156.1, 151.2, 137.6 (q, $^3J_{C-F}$ = 5.5 Hz), 127.1, 125.2 (q, $^2J_{C-F}$ = 36.5 Hz), 121.9 (q, $^1J_{C-F}$ = 274.4 Hz), 52.5, 48.6, 27.9; **^{19}F NMR** (377 MHz, $CDCl_3$) δ -58.00 (s); **HRMS** (ESI$^+$) found *m/z* 298.0357 $[M+H]^+$, $C_{10}H_{11}O$-$_4N_1F_3S$ requires m/z 298.0355; **IR** v_{max} (neat)/cm^{-1} 3060, 2956, 2360, 1739, 1586, 149, 1418, 1364, 1314, 1266, 1231, 1160, 1125, 1065, 1032, 981, 898, 817, 732, 702; **R_f** = 0.13 (40% EtOAc in Petrol).

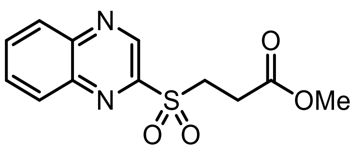

methyl 3-(quinoxalin-2-ylsulfonyl)propanoate (4h)

Following general procedure **C,** methyl 3-(quinoxalin-2-ylthio)propanoate (429 mg, 1.73 mmol, 1.0 equiv.), $Na_2WO_4 \cdot 2H_2O$ (28.5 mg, 0.087 mmol, 5.0 mol%), hydrogen peroxide solution (30% w/w water) (1.77 mL, 17.3 mmol, 10.0 equiv.) in ethanol (2.5 mL, 0.6 M) was stirred at RT for 18 h. On diluting with water, a white solid precipitated out of the reaction mixture. The white solid was collected by filtration, washed with copious amounts of water and dried *in vacuo* to give the title compound as a fine white solid (375 mg, 77%).

^{1}H NMR (400 MHz, $CDCl_3$) δ 9.49 (s, 1H, Ar-*H*), 8.29 – 8.20 (m, 2H, Ar-*H*), 8.03 – 7.92 (m, 2H, Ar-*H*), 3.90 (t, *J* = 7.5 Hz, 2H, SO_2CH_2), 3.67 (s, 3H, CO_2Me), 2.96 (t, *J* = 7.5 Hz, 2H, CH_2CO_2Me); **^{13}C NMR** (101 MHz, $CDCl_3$) δ 170.5, 151.5, 144.1, 141.3, 140.9, 133.4, 132.2, 130.3, 129.9, 52.6, 48.3, 27.5; **LRMS** (ESI$^+$) *m/z* 303.0 [M+Na]$^+$; **HRMS** (ESI$^+$) found *m/z* 281.0592 [M+H]$^+$, $C_{12}H_{13}O_4N_2S$ requires m/z 281.0591; **IR** ν_{max} (neat)/cm^{-1} 3050, 3003, 2953, 2849, 1731, 1611, 1562, 1489, 1436, 1420, 1364, 1318, 1276, 1205, 1169, 1131, 1100, 1057, 965, 930, 870, 801, 782, 769, 708, 639; **mp** 105-107 °C (EtOAc); **R$_f$** = 0.11 (30% EtOAc in Petrol).

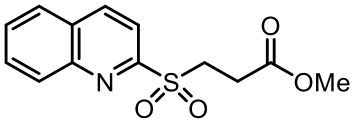

methyl 3-(quinolin-2-ylsulfonyl)propanoate (4i)

Following general procedure **C,** methyl 3-(quinolin-2-ylthio)propanoate (90 mg, 0.360 mmol, 1.0 equiv.), $Na_2WO_4 \cdot 2H_2O$ (5.9 mg, 0.018 mmol, 5.0 mol%.) and hydrogen peroxide solution (30% w/w in water) (185 µL, 1.82 mmol, 5.0 equiv.) in ethanol (1.0 mL, 0.35 M) was stirred at RT for 20 h, then diluted with water. No precipitate was observed so the reaction mixture was extracted with EtOAc (20 mL × 4). The combined organic layers were then washed with brine (10 mL), dried over $MgSO_4$ and concentrated *in vacuo*. The crude product was then purified by flash column chromatography (35% EtOAc in Petrol) to give the title compound as white crystalline solid (80.2 mg, 80%).

^{1}H NMR (400 MHz, $CDCl_3$) δ 8.45 (dd, *J* = 8.5, 1.0 Hz, 1H, Ar-*H*), 8.22 (dd, *J* = 8.5, 1.0 Hz, 1H, Ar-*H*), 8.12 (d, *J* = 8.5 Hz, 1H, Ar-*H*), 7.98 – 7.92 (m, 1H, Ar-*H*), 7.87 (ddd, *J* = 8.5, 7.0, 1.5 Hz, 1H, Ar-*H*), 7.73 (ddd, *J* = 8.0, 7.0, 1.0 Hz, 1H, Ar-*H*), 3.90 (t, *J* = 7.5 Hz, 2H, SO_2CH_2), 3.67 (s, 3H, $COOCH_3$), 2.94 (t, *J* = 7.5 Hz, 2H, CH_2CO_2Me); **^{13}C NMR** (101 MHz, $CDCl_3$) δ 170.8, 156.7, 147.3, 139.1, 131.5, 130.4, 129.6, 129.4, 128.1, 117.2, 52.4, 47.7, 27.7; **LRMS** (ESI^+) *m/z* 302.0 $[M+Na]^+$; **HRMS** (ESI^+) found *m/z* 280.0638 $[M+H]^+$, $C_{13}H_{14}O_4NS$ requires m/z 280.0638; **IR** v_{max} (neat)/cm^{-1} 3005, 2957, 2161, 2033, 1727, 1616, 1580, 1498, 1367, 1313, 1275, 1237, 1200, 1180, 1163, 1124, 1097, 1054, 981, 945, 898, 856, 840, 802, 793, 772, 761, 705, 641, 615; **mp** 53-54 °C (EtOAc); **R_f** = 0.18 (30% EtOAc in Petrol). Data consistent with literature: B. Du, P. Qian, Y. Wang, H. Mei, J. Han and Y. Pan, *Org. Lett.*, 2016, **18**, 4144-4147.[6]

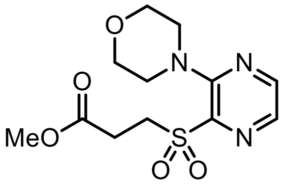

methyl 3-((3-morpholinopyrazin-2-yl)sulfonyl)propanoate (4j)

Following general procedure **C,** methyl 3-((3-morpholinopyrazin-2-yl)thio)propanoate (458 mg, 1.62 mmol, 1.0 equiv.), $Na_2WO_4 \cdot 2H_2O$ (26.6 mg, 0.08 mmol, 5.0 mol%.) and hydrogen peroxide solution (30% w/w in water) (825 µL, 8.08 mmol, 5.0 equiv.) in ethanol (2.5 mL, 0.6 M) was stirred at RT for 20 h, then diluted with water. No precipitate was observed so the reaction mixture was extracted with EtOAc (20 mL × 4). The combined organic layers were then washed with brine (10 mL), dried over $MgSO_4$ and concentrated *in vacuo*. The crude product was then purified by flash column chromatography (55% EtOAc in Petrol) to give the title compound as a viscous yellow oil (418 mg, 82%).

^{1}H NMR (400 MHz, $CDCl_3$) δ 8.26 (d, *J* = 2.0 Hz, 1H, Ar-*H*), 7.95 (d, *J* = 2.0 Hz, 1H, Ar-*H*), 3.88 – 3.81 (m, 6H, SO_2CH_2 and $O(CH_2)_2$), 3.74 – 3.70 (m, 7H, OCH_3 and $(CH_2)_2N$), 2.78 (t, *J* = 8.0 Hz, 2H, CH_2COOMe); **^{13}C NMR** (101 MHz, $CDCl_3$) δ 170.9, 152.7, 144.6, 139.5, 132.5, 66.9, 52.5, 50.2, 48.8, 27.8; **LRMS** (ESI$^+$) *m/z* 338.0 [M+Na]$^+$; **HRMS** (ESI$^+$) found *m/z* 316.0963 [M+H]$^+$, $C_{12}H_{18}O_5N_3S$ requires m/z 316.0962; **IR** ν_{max} (neat)/cm^{-1} 2955, 2859, 1738, 1556, 1492, 1446, 1364, 1308, 1272, 1249, 1217, 1180, 1068, 1045, 980, 946, 855, 806, 718, 620; **R$_f$** = 0.18 (50% EtOAc in Petrol).

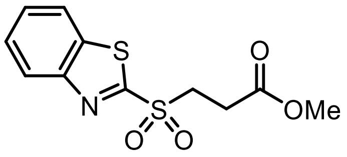

methyl 3-(benzo[*d*]thiazol-2-ylsulfonyl)propanoate (4k)

Following General procedure **C**, methyl 3-(benzo[*d*]thiazol-2-ylthio)propanoate (1.00 g, 4.0 mmol, 1.0 equiv.), $Na_2WO_4 \cdot 2H_2O$ (66 mg, 0.2 mmol, 5.0 mol%) and hydrogen peroxide solution (30% w/w in water) (2.0 mL, 20 mmol, 5.0 equiv.) in ethanol (5.7 mL, 0.7 M) were stirred at RT for 16 h. On diluting with water, a white solid precipitated out of the reaction mixture. The white solid was collected by filtration, washed with copious amounts of water and dried *in vacuo* to give the title compound as a white solid (940 mg, 82%).

^{1}H NMR (400 MHz, $CDCl_3$) δ 8.21 (dd, *J* = 7.5, 1.5 Hz, 1H, Ar-*H*), 8.01 (dd, *J* = 7.5, 1.5 Hz, 1H, Ar-*H*), 7.66 – 7.58 (m, 2H, Ar-*H*), 3.84 (t, *J* = 7.5 Hz, 2H, SO_2CH_2), 3.66 (s, 3H, OCH_3), 2.96 (t, *J* = 7.5 Hz, 2H, CH_2CO_2Me); **^{13}C NMR** (101 MHz, $CDCl_3$) δ 170.2, 165.2, 152.7, 136.9, 128.3, 127.9, 125.6, 122.5, 52.6, 50.4, 27.7; **HRMS** (ESI$^+$) found *m/z* 286.0202 $[M+H]^+$, $C_{11}H_{12}NO_4S_2$ requires m/z 286.0202; **IR** v_{max} (neat)/cm^{-1} 2990, 2954, 1736, 1328, 1253, 1150, 1137, 763, 729; **R_f** = 0.23 (20% EtOAc in Petrol); **mp** 88-89 °C (CH_2Cl_2).

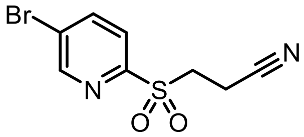

3-((5-bromopyridin-2-yl)sulfonyl)propanenitrile (9a)

Following general procedure **C,** 3-((5-bromopyridin-2-yl)thio)propanenitrile (348.2 mg, 1.43 mmol, 1.0 equiv.), $Na_2WO_4 \cdot 2H_2O$ (46.2 mg, 0.140 mmol, 10 mol%), hydrogen peroxide solution (30% w/w water) (1.46 mL, 14.3 mmol, 10.0 equiv.) in ethanol (2.0 mL, 0.7 M) was stirred at RT for 18 h. On diluting with water, a white solid precipitated out of the reaction mixture. The white solid was collected by filtration, washed with copious amounts of water and dried *in vacuo* to give the title compound as a white solid (305.4 mg, 78%).

^{1}H NMR (400 MHz, $CDCl_3$) δ 8.81 (dd, *J* = 2.5, 1.0 Hz, 1H, Pyr-*H*), 8.15 (dd, *J* = 8.5, 2.5 Hz, 1H, Pyr-*H*), 8.00 (dd, *J* = 8.5, 1.0 Hz, 1H, Pyr-*H*), 3.72 (t, *J* = 7.5 Hz, 2H, SO_2CH_2), 2.95 (t, *J* = 7.5 Hz, 2H, CH_2CN); **^{13}C NMR** (101 MHz, $CDCl_3$) δ 154.8, 151.8, 141.4, 126.4, 123.6, 115.9, 47.3, 12.3; **HRMS** (ESI$^+$) found *m/z* 274.9485 $[M(^{79}Br)+H]^+$, $C_8H_8O_2N_2S^{79}Br$ requires m/z 274.9485; **IR** v_{max} (neat)/cm^{-1} 3082, 3065, 2980, 2970, 2930, 2889, 2248, 1565, 1549, 1445, 1422, 1394, 1382, 1358, 1335, 1310, 1295; **mp** 97-99 °C (EtOAc); **R_f =** 0.37 (50% EtOAc in Petrol).

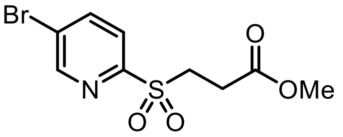

methyl 3-((5-bromopyridin-2-yl)sulfonyl)propanoate (9b)

Following general procedure **C,** methyl 3-((5-bromopyridin-2-yl)thio)propanoate (460.5 mg, 1.67 mmol, 1.0 equiv.), $Na_2WO_4 \cdot 2H_2O$ (55.1 mg, 0.167 mmol, 10 mol%), hydrogen peroxide solution (30% w/w water) (1.70 mL, 16.7 mmol, 10.0 equiv.) in ethanol (2.4 mL, 0.7 M) was stirred at RT for 18 h. On diluting with water, a white solid precipitated out of the reaction mixture. The white solid was collected by filtration, washed with copious amounts of water and dried *in vacuo* to give the title compound as a bright white solid (531 mg, 99%).

^{1}H NMR (400 MHz, $CDCl_3$) δ 8.79 (dd, *J* = 2.0, 1.0 Hz, 1H, Pyr-*H*), 8.11 (dd, *J* = 8.5, 2.0 Hz, 1H, Pyr-*H*), 7.97 (dd, *J* = 8.5, 1.0 Hz, 1H, Pyr-*H*), 3.73 – 3.69 (m, 5H, SO_2CH_2 and CO_2Me), 2.84 (t, *J* = 7.5 Hz, 2H, CH_2CO_2Me); **^{13}C NMR** (101 MHz, $CDCl_3$) δ 170.5, 155.5, 151.6, 141.0, 125.8, 123.6, 52.5, 47.9, 27.5; **LRMS** (ESI$^+$) *m/z* 329.9 (50%, [M (^{79}Br)+Na]$^+$), 331.9 (50%, %, [M (^{81}Br)+Na]$^+$); **HRMS** (ESI$^+$) found *m/z* 307.9587 [M(^{79}Br)+H]$^+$, $C_9H_{11}O_4N^{79}BrS$ requires m/z 307.9587; **IR** ν_{max} (neat)/cm^{-1} 3082, 3056, 2981, 2958, 2890, 1737, 1561, 1550, 1441, 1422, 1361, 1319, 1252, 1201, 1162, 1127, 1099, 1085, 1063, 1008, 979, 834, 806, 770, 711, 623; **mp** 58-60 °C (EtOAc); **R_f =** 0.42 (40% EtOAc in Petrol).

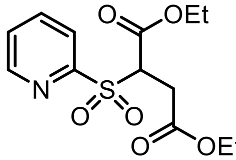

diethyl 2-(pyridin-2-ylsulfonyl)succinate (5a)

Following general procedure **C,** diethyl 2-(pyridin-2-ylthio)succinate (464 mg, 1.64 mmol, 1.0 equiv.), $Na_2WO_4 \cdot 2H_2O$ (54.0 mg, 0.16 mmol, 10 mol%) and hydrogen peroxide solution (30% w/w in water) (837 µL, 8.2 mmol, 5.0 equiv.) in ethanol (2.3 mL, 0.7 M) was stirred at RT for 20 h, then diluted with water. No precipitate was observed so the reaction mixture was extracted with EtOAc (20 mL × 4). The combined organic layers were then washed with brine (10 mL), dried over $MgSO_4$ and concentrated *in vacuo*. The crude product was then purified by flash column chromatography (45% EtOAc in Petrol) to give the title compound as a white solid (305 mg, 59%).

^{1}H NMR (400 MHz, $CDCl_3$) δ 8.81 – 8.79 (m, 1H, Pyr-*H*), 8.07 (d, *J* = 7.5 Hz, 1H, Pyr-*H*), 7.98 (td, *J* = 7.5, 2.0 Hz, 1H, Pyr-*H*), 7.60 (ddd, *J* = 7.5, 5.0, 1.0 Hz, 1H, Pyr-*H*), 5.05 (ddd, *J* = 10.5, 4.0, 1.0 Hz, 1H, SO_2CH), 4.21 – 4.14 (m, 2H, CO_2CH_2), 4.13 – 4.05 (m, 2H, CO_2CH_2), 3.31 (dd, *J* = 17.0, 11.0 Hz, 2H, $SO_2CHCH_aH_b$), 3.23 (dd, *J* = 17.0, 4.5 Hz, 2H, $SO_2CHCH_aH_b$), 1.26 (t, *J* = 7.0 Hz,

3H, CH_2CH_3), 1.06 (t, *J* = 7.0 Hz, 3H, CH_2CH_3); **^{13}C NMR** (101 MHz, $CDCl_3$) δ 169.9, 164.7, 156.4, 150.6, 138.2, 127.9, 123.3, 62.6, 61.7, 30.0, 14.2, 13.8**; HRMS** (ESI$^+$) found *m/z* 316.0852 $[M+H]^+$, $C_{13}H_{18}O_6NS$ requires m/z 316.0849; **IR** ν_{max} (neat)/cm^{-1} 3066, 2990, 2941, 2870, 1736, 1580, 1453, 1428, 1372, 1325, 1215, 1165, 1110, 1083, 1026, 992, 857, 782, 747, 639; **mp** 77-78 °C (EtOAc)**; R$_f$** = 0.13 (40% EtOAc in Petrol).

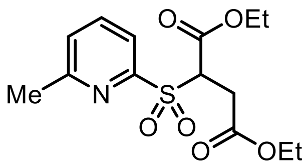

diethyl 2-((6-methylpyridin-2-yl)sulfonyl)succinate (5b)

Following general procedure **C,** diethyl 2-((6-methylpyridin-2-yl)thio)succinate (188.3 mg, 0.63 mmol, 1.0 equiv.), $Na_2WO_4 \cdot 2H_2O$ (21.0 mg, 0.063 mmol, 10 mol%) hydrogen peroxide solution (30% w/w water) (647 µL, 6.33 mmol, 10.0 equiv.) in ethanol (1.0 mL, 0.6 M) was stirred at RT for 18 h. On diluting with water, a white solid precipitated out of the reaction mixture. The white solid was collected by filtration, washed with copious amounts of water and dried *in vacuo* to give the title compound as a white solid (115.1 mg, 55%).

^{1}H NMR (400 MHz, $CDCl_3$) δ 7.87 (dd, *J* = 8.0, 1.5 Hz, 1H, Pyr-*H*), 7.83 (app. t, *J* = 7.5 Hz, 1H, Pyr-*H*), 7.42 (dd, *J* = 7.5, 1.5 Hz, 1H, Pyr-*H*), 5.04 (dd, *J* = 11.0, 4.0 Hz, 1H, SO_2CH), 4.22 – 4.03 (m, 4H, 2 × OCH_2), 3.30 (dd, *J* = 17.5, 11.0 Hz, 1H, $SO_2CHCH_aH_b$), 3.20 (dd, *J* = 17.5, 4.0 Hz, 1H, $SO_2CHCH_aH_b$), 2.67 (s, 3H, Pyr-*Me*), 1.27 (t, *J* = 7.0 Hz, 3H, CH_2CH_3), 1.06 (t, *J* = 7.0 Hz, 3H, , CH_2CH_3); **^{13}C NMR** (101 MHz, $CDCl_3$) δ 170.0, 164.8, 160.6, 155.5, 138.0, 127.8, 120.3, 62.5, 62.4, 61.7, 30.0, 24.5, 14.2, 13.8; **LRMS** (ESI$^+$) *m/z* 352.0 $[M+Na]^+$; **HRMS** (ESI$^+$) found *m/z* 330.1007 $[M+H]^+$, $C_{14}H_{20}O_6NS$ requires m/z 330.1006; **IR** ν_{max} (neat)/cm^{-1} 2982, 2940, 2907, 1737, 1594, 1556, 1454, 1413, 1395, 1372, 1324, 1256, 1216, 1175, 1153, 1119, 1097, 1026, 991, 864, 836, 797, 735, 640; **mp** 70-71 °C (EtOAc); **R$_f$ =** 0.26 (20% EtOAc in Petrol).

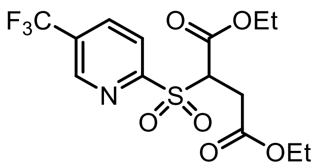

diethyl 2-((5-(trifluoromethyl)pyridin-2-yl)sulfonyl)succinate (5c)

Following general procedure **C,** diethyl 2-((5-(trifluoromethyl)pyridin-2-yl)thio)succinate (2.00 g, 5.69 mmol, 1.0 equiv.), $Na_2WO_4 \cdot 2H_2O$ (87 mg, 0.26 mmol, 5.0 mol%) and hydrogen peroxide solution (30% w/w in water) (5.35 mL, 52.2 mmol, 9.2 equiv.) in ethanol (7.5 mL, 0.8 M) was stirred at RT for 16 h, then diluted with water. No precipitate was observed so the reaction mixture was extracted with EtOAc (20 mL × 4). The combined organic layers were then washed with brine (10 mL), dried over $MgSO_4$ and concentrated *in vacuo*. The crude product was purified by flash column chromatography (10-20% EtOAc in Petrol) to give the title compound as a colorless oil (1.75 g, 80%).

^{1}H NMR (400 MHz, $CDCl_3$) δ 9.04 – 9.03 (m, 1H, Ar-*H*), 8.24 (ddd, *J* = 8.0, 2.0, 0.5 Hz, 1H, Ar-*H*), 8.20 (d, *J* = 8.0 Hz, 1H, Ar-*H*), 5.11 (dd, *J* = 10.0, 5.0 Hz, 1H, SO_2CH), 4.18 (q, *J* = 7.0 Hz, 2H, OCH_2), 4.15 – 3.94 (m, 2H, OCH_2), 3.36 – 3.18 (m, 2H, $SCHCH_2$), 1.26 (t, *J* = 7.0 Hz, 3H, CH_2CH_3), 1.05 (t, *J* = 7.0 Hz, 3H, CH_2CH_3); **^{13}C NMR** (101 MHz, $CDCl_3$) δ 169.6, 164.3, 159.5, 147.5 (q, $^3J_{C-F}$ = 4.0 Hz), 135.8 (q, $^3J_{C-F}$ = 3.5 Hz), 130.4 (q, $^3J_{C-F}$ = 31.5 Hz), 123.0, 122.6 (q, $^1J_{C-F}$ = 272.0 Hz), 62.8, 62.4, 61.8, 29.6, 14.2, 13.7; **^{19}F NMR** (377 MHz, $CDCl_3$) δ -62.63 (s); **LRMS** (ESI$^+$) *m/z* 406.0 $[M+Na]^+$; **HRMS** (ESI$^+$) found *m/z* 384.0731 $[M+H]^+$, $C_{14}H_{17}O_6NF_3S$ requires m/z 384,0723; **IR** ν_{max} (neat)/cm^{-1} 2987, 1737, 1326, 1162, 1141, 1099, 1072, 1015, 723; **R_f** = 0.27 (10% EtOAc in Petrol).

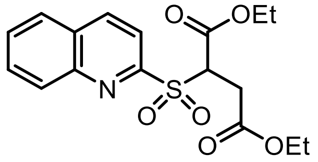

diethyl 2-(quinolin-2-ylsulfonyl)succinate (5d)

Following general procedure **C,** diethyl 2-(quinolin-2-ylthio)succinate (547mg, 1.64 mmol, 1.0 equiv.), $Na_2WO_4 \cdot 2H_2O$ (27 mg, 0.082 mmol, 5.0 mol%) and hydrogen peroxide solution (30% w/w in water) (1.7 mL, 16.4 mmol, 10.0 equiv.) in ethanol (2.5 mL, 0.7 M) was stirred at RT for 16 h, then diluted with water. No precipitate was observed so the reaction mixture was extracted with EtOAc (20 mL × 4). The combined organic layers were then washed with brine (10 mL), dried over $MgSO_4$ and concentrated *in vacuo*. The crude product was purified by flash column chromatography (10-30% EtOAc in Petrol) to give the title compound as a colorless oil that solidified upon standing (296 mg, 45%).

^{1}**H NMR** (400 MHz, $CDCl_3$) δ 8.41 (d, *J* = 8.5 Hz, 1H, Ar-*H*), 8.19 (d, *J* = 8.5 Hz, 1H, Ar-*H*), 8.05 (d, *J* = 8.5 Hz, 1H, Ar-*H*), 7.91 (d, *J* = 8.5 Hz, 1H, Ar-*H*), 7.82 (ddd, *J* = 8.5, 7.0, 1.5 Hz, 1H, Ar-*H*), 7.69 (ddd, *J* = 8.0, 7.0, 1.0 Hz, 1H, Ar-*H*), 5.19 (dd, *J* = 10.5, 4.5 Hz, 1H, SO_2CH), 4.13 (q, *J* = 7.0 Hz, 2H, OCH_2), 4.11 – 3.92 (m, 2H, OCH_2), 3.32 (dd, *J* = 17.5, 10.5 Hz, 1H, $SO_2CHCH_aH_b$), 3.24 (dd, *J* = 17.0, 4.5 Hz, 1H, $SO_2CHCH_aH_b$), 1.21 (t, *J* = 7.0 Hz, 3H, CH_2CH_3), 0.91 (t, *J* = 7.0 Hz, 3H, CH_2CH_3); ^{13}**C NMR** (101 MHz, $CDCl_3$) δ 169.7, 164.6, 155.5, 147.2, 138.8, 131.4, 130.1, 129.7, 129.2, 127.9, 118.0, 62.6, 62.4, 61.5, 30.0, 14.0, 13.5; **LRMS** (ESI^+) *m/z* 388.0 $[M+Na]^+$; **HRMS** (ESI^+) found *m/z* 366.1015 $[M+H]^+$, $C_{17}H_{20}O_6NS$ requires m/z 366.1006; **IR** v_{max} (neat)/cm^{-1} 2984, 2940, 1735, 1323, 1213, 1164, 1128, 1094, 1025, 831, 757; **mp** 58-60 °C (CH_2Cl_2); $\mathbf{R_f}$ = 0.34 (30% EtOAc in Petrol).

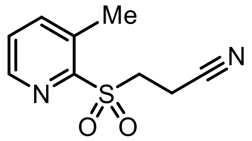

3-((3-methylpyridin-2-yl)sulfonyl)propanenitrile (1g)

Following general procedure **D**, lithium 3-methylpyridine-2-sulfinate (300 mg, 1.84 mmol, 1.0 equiv.), acrylonitrile (603 µL, 9.2mmol, 5.0 equiv.) and AcOH (105 µL, 1.84 mmol, 1.0 equiv.) were refluxed in water (3.5 mL, 0.5 M) for 16 h. After workup, the crude product was purified by flash column chromatography (20-30% EtOAc in Petrol) and repurified by flash column chromatography (5-10% EtOAc in toluene) to give the title compound as a clear, colourless oil (213 mg, 55%).

^{1}**H NMR** (400 MHz, $CDCl_3$) δ 8.43 – 8.42 (m, 1H, Ar-*H*), 7.72 – 7.71 (m, 1H, Ar-*H*), 7.45 (dd, *J* = 7.5, 4.5 Hz, 1H, Ar-*H*), 3.95 – 3.87 (m, 2H, SO_2CH_2), 3.07 – 2.99 (m, 2H, CH_2CN), 2.69 (s, 3H, Pyr-CH_3); ^{13}**C NMR** (101 MHz, $CDCl_3$) δ 155.4, 146.0, 141.9, 133.2, 127.4, 116.7, 47.3, 17.7, 12.9; **LRMS** (ESI^+) m/z 211.0 (50%, $[M+H]^+$), 233.0 (50%, $[M+Na]^+$)**;** **HRMS** (ESI^+) found *m/z* 211.0539 $[M+H]^+$, $C_9H_{11}O_2N_2S$ requires m/z 211.0536; **IR** v_{max} (neat)/cm^{-1} 30003, 2974, 2941, 2361, 2341, 2252, 1563, 1452, 1423, 1399, 1303, 1242, 1205, 1151, 1123, 1094, 1040, 954, 824, 803, 751, 714, 668; $\mathbf{R_f}$ = 0.34 (60% EtOAc in Petrol) or $\mathbf{R_f}$ = 0.36 (20% EtOAc in toluene).

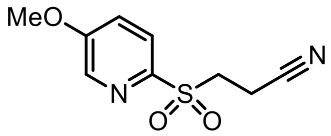

3-((5-methoxypyridin-2-yl)sulfonyl)propanenitrile (1h)

Following a modified version of general procedure **D**, crude sodium 5-methoxypyridine-2-sulfinate (390 mg, 2.0 mmol, 1.0 equiv.), acrylonitrile (197 µL, 3.0 mmol, 1.5 equiv.) and AcOH (170 µL, 3.0 mmol, 1.5 equiv.) were refluxed in water (4.5 mL, 0.4 M) for 16 h. After workup, the resultant crude product required no further purification to give the title compound as a white solid (285 mg, 63%).

^{1}**H NMR** (400 MHz, $CDCl_3$) δ 8.37 (d, *J* = 3.0 Hz, 1H, Ar-*H*), 8.03 (d, *J* = 9.0 Hz, 1H, Ar-*H*), 7.36 (dd, *J* = 9.0, 3.0 Hz, 1H, Ar-*H*), 3.94 (s, 3H, O*Me*), 3.63 (t, *J* = 7.5 Hz, 2H, SO_2CH_2), 2.88 (t, *J* = 7.5 Hz, 2H, CH_2CN); ^{13}**C NMR** (101 MHz, $CDCl_3$) δ 159.0, 147.2, 139.4, 124.5, 120.5, 116.3, 56.3, 47.7, 12.2; **LRMS** (ESI^+) *m/z* 249.0 $[M+Na]^+$; **HRMS** (ESI^+) found *m/z* 227.0487 $[M+H]^+$, $C_9H_{11}O_3N_2S$ requires m/z 227.0485; **IR** v_{max} (neat)/cm^{-1} 3088, 3019, 2846, 1573, 1466, 1313, 1165, 1133, 1098, 1018, 842, 713; **mp** 85-90 °C (CH_2Cl_2); **R$_f$** = 0.24 (50% EtOAc in Petrol).

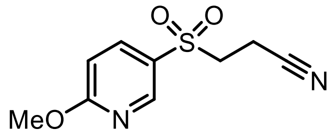

3-((6-methoxypyridin-3-yl)sulfonyl)propanenitrile (1i)

Following general procedure **D**, lithium 6-methoxypyridine-3-sulfinate (268.7 mg, 1.5 mmol, 1.1 equiv.), acrylonitrile (90.4 µL, 1.37 mmol, 1.0 equiv.) and AcOH (86.0 µL, 1.5 mmol, 1.1 equiv.) were refluxed in water (3.4 mL, 0.4 M) for 6 h. On addition of cold water (4 mL) and cooling, a white precipitate crashed out of the reaction mixture. The precipitate was collected by vacuum filtration, wash with water and dried to give the title compound as a white solid (258 mg, 84%).

^{1}H NMR (400 MHz, $CDCl_3$) δ 8.71 (dd, *J* = 2.5, 1.0 Hz, 1H, Ar-*H*), 8.00 (dd, *J* = 9.0, 2.5 Hz, 1H, Ar-*H*), 6.91 (dd, *J* = 9.0, 1.0 Hz, 1H, Ar-*H*), 4.04 (s, 3H, OC*H*$_3$), 3.39 (t, *J* = 8.0 Hz, 2H, SO_2C*H*$_2$), 2.86 (t, *J* = 8.0 Hz, 2H, C*H*$_2$CN); **^{13}C NMR** (101 MHz, $CDCl_3$) δ 168.0, 149.5, 138.1, 127.0, 116.0, 112.3, 54.8, 51.9, 12.2; **HRMS** (ESI$^+$) found *m/z* 227.0487 [M+H]$^+$, $C_9H_{11}O_3N_2S$ requires m/z 227.0485; **IR** ν_{max} (neat)/cm^{-1} 2990, 2950, 2246, 1734, 1593, 1560, 1486, 1461, 1434, 1377, 1322, 1289, 1244, 1172, 1150, 1128, 1095, 1012, 943, 847, 786, 768, 727; **mp** 122-124 °C (AcOH: H_2O); **R$_f$** = 0.21 (20% EtOAc in Petrol).

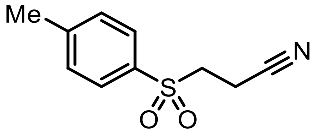

3-tosylpropanenitrile (1j)

Following general procedure **D**, sodium *p*-toluenesulfinate (196.0 mg, 1.1 mmol, 1.1 equiv.), acrylonitrile (65.5 µL, 1.0 mmol, 1.0 equiv.) and AcOH (63.0 µL, 1.1 mmol, 1.1 equiv.) were refluxed in water (4.0 mL, 0.25 M) for 2.5 h. The reaction was cooled to RT and a white precipitate formed. The solid was collected by vacuum filtration, washed with water and dried over vacuum to give the title compound as a white solid (153.2 mg, 73%).

^{1}H NMR (400 MHz, $CDCl_3$) δ 7.81 (d, 2H, *J* = 8.5 Hz, Ar-*H*), 7.42 (d, 2H, *J* = 8.5 Hz, Ar-*H*), 3.37 (t, *J*= 8.0 Hz, 2H, SO_2C*H*$_2$), 2.81 (t, *J* = 8.0 Hz, 2H, C*H*$_2$CN), 2.48 (s, 3H, Ar-C*H*$_3$); **^{13}C NMR** (101 MHz, $CDCl_3$) 146.2, 134.7, 130.6, 128.5, 116.1, 51.4, 21.9, 12.2; **HRMS** (ESI$^+$) found *m/z* 232.0404 [M+Na]$^+$, $C_{10}H_{11}O_2NSNa$ requires m/z 232.0403; **IR** ν_{max} (neat)/cm^{-1} 2991, 2946, 2247, 1596, 1496, 1422, 1303, 1292, 1245, 1168, 1139, 1085, 1019, 985, 828, 808, 780, 757, 637; **mp** 86-88 °C (H_2O); **R$_f$** = 0.24 (50% EtOAc in Petrol). Data is consistent with literature: W. Li, L. Gao, W. Zhuge, X. Sun and G. Zheng, *Org. Biomol. Chem.*, 2017, **15**, 7819-7823.[7]

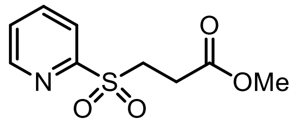

methyl 3-(pyridin-2-ylsulfonyl)propanoate (4l)

Following general procedure **D**, sodium pyridine-2-sulfinate (182 mg, 1.10 mmol, 1.1 equiv.), methyl acrylate (90 µL, 1.00 mmol, 1.0 equiv.) and AcOH (57 µL, 1.00 mmol, 1.0 equiv.) were refluxed in water (3.0 mL, 0.3 M) for 2.5 h. After workup, the crude product was purified by flash column chromatography (50% EtOAc in Petrol) to give the title compound as a clear, colourless oil (211 mg, 92%).

^{1}H NMR (400 MHz, $CDCl_3$) δ 8.75 (ddd, *J* = 5.0, 2.0, 1.0 Hz, 1H, Ar-*H*), 8.08 (app. dt, *J* = 8.0, 1.0 Hz, 1H, Ar-*H*), 7.98 (app. td, *J* = 8.0, 2.0 Hz, 1H, Ar-*H*), 7.57 (ddd, *J* = 8.0, 5.0, 1.0 Hz, 1H, Ar-*H*), 3.73 (t, *J* = 8.0 Hz, 2H, SO_2CH_2), 3.68 (s, 3H, $COOCH_3$), 2.84 (t, *J* = 8.0 Hz, 2H, CH_2CO_2Me); **^{13}C NMR** (101 MHz, $CDCl_3$) δ 170.6, 157.1, 150.4, 138.4, 127.7, 122.3, 52.5, 47.7, 27.5; **LRMS** (ESI^+) *m/z* 252.0 $[M+Na]^+$; **HRMS** (ESI^+) found *m/z* 252.0301 $[M+Na]^+$, $C_9H_{11}O_4NNaS$ requires m/z 252.0301; **IR** v_{max} (neat)/cm^{-1} 2955, 2349, 1736 (CO), 1579, 1428, 1365, 1315, 1252, 1201, 1164, 1110, 1083, 1044, 1017, 992, 900, 834, 809, 782, 752, 700, 617; **R_f** = 0.32 (60% EtOAc in Petrol).

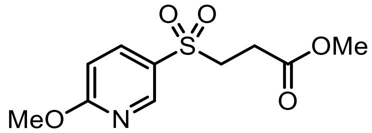

methyl 3-((6-methoxypyridin-3-yl)sulfonyl)propanoate (4m)

Following general procedure **D**, lithium 6-methoxypyridine-3-sulfinate (150.0 mg, 0.84 mmol, 1.1 equiv.), methyl acrylate (68.6 µL, 0.76 mmol, 1.0 equiv.) and AcOH (48.0 µL, 0.84 mmol, 1.1 equiv.) were refluxed in water (2.0 mL, 0.4 M) for 6 h. After workup, the product was concentrated *in vacuo* to give the title compound as a crystalline white solid (174 mg, 88%).

^{1}H NMR (400 MHz, $CDCl_3$) δ 8.63 (dd, *J* = 2.5, 1.0 Hz, 1H, Ar-*H*), 7.94 (dd, *J* = 9.0, 2.5 Hz, 1H, Ar-*H*), 6.83 (dd, *J* = 9.0, 1.0 Hz, 1H, Ar-*H*), 3.98 (s, 3H, CH_3OAr), 3.61 (s, 3H, $COOCH_3$), 3.40 (t, *J* = 7.5 Hz, 2H, SO_2CH_2), 2.73 (t, *J* = 7.5 Hz, 2H, CH_2COOMe); **^{13}C NMR** (101 MHz, $CDCl_3$) δ 170.4, 167.4, 149.0, 138.1, 127.8, 111.8, 54.5, 52.4, 52.0, 27.7; **LRMS** (ESI^+) m/z 260.0 (50%, $[M+H]^+$), 282.0 (50%, $[M+Na]^+$)**;** **HRMS** (ESI^+) found *m/z* 260.0589 $[M+H]^+$, $C_{10}H_{14}O_5NS$ requires m/z 260.0587; **IR** v_{max} (neat)/cm^{-1} 2991, 2953, 2161, 1732, 1590, 1563, 1485, 1437, 1417, 1371, 1316, 1292, 1259, 1181, 1160, 1131, 1099, 1071, 1018, 1007, 989, 953, 902, 853, 825, 795, 777, 698, 669, 630; **mp** 80-84 °C (EtOAc); **R_f** = 0.31 (40% EtOAc in Petrol).

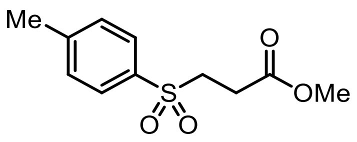

methyl 3-tosylpropanoate (4n)

Following general procedure **D**, sodium *p*-toluenesulfinate (196.0 mg, 1.10 mmol, 1.1 equiv.), methyl acrylate (90.1 µL, 1.00 mmol, 1.0 equiv.) and AcOH (63.0 µL, 1.10 mmol, 1.1 equiv.) were refluxed in water (4.0 mL, 0.25 M) for 2.5 h. The reaction was cooled to RT and a white precipitate was formed. The solid was collected by vacuum filtration and dried over vacuum to give the title compound as a white solid (166.8 mg, 69%).

^{1}H NMR (400 MHz, $CDCl_3$) δ 7.79 (d, 2H, *J* = 8.5 Hz, Ar-*H*), 7.36 (d, 2H, *J* = 8.5 Hz, Ar-*H*), 3.64 (s, 3H, COOC), 3.41 (t, *J* = 7.5 Hz, 2H, SO_2CH_2), 2.75 (t, *J* = 7.5 Hz, 2H, CH_2COOMe), 2.46 (s, 3H, Ar-CH_3); **^{13}C NMR** (101 MHz, $CDCl_3$) δ 170.7, 145.2, 135.7, 130.2, 128.4, 52.4, 51.7, 27.9, 21.8; **HRMS** (ESI$^+$) found *m/z* 265.0507 [M+Na]$^+$, $C_{11}H_{14}O_4SNa$ requires m/z 265.0505; **IR** ν_{max} (neat)/cm^{-1} 3072, 3037, 2936, 2926, 2847, 2824, 1731, 1596, 1439, 1422, 1366, 1315, 1290, 1260, 1196, 1179, 1153, 1132, 1087, 1061, 985, 815, 768; **mp** 74-75 °C (H_2O)**;** **R_f** = 0.31 (50% EtOAc in Petrol). Data is consistent with literature: H. E. Bartrum, D. C. Blakemore, C. J. Moody and C. J. Hayes, *Tetrahedron*, 2013, **69**, 2276-2282.[8]

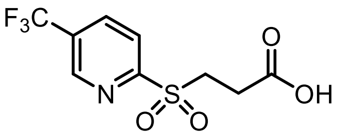

3-((5-(trifluoromethyl)pyridin-2-yl)sulfonyl)propanoic acid (4a-OH)

Following a modified version of general procedure **D**, sodium 5-(trifluoromethyl)pyridine-2-sulfinate (233 mg, 1.00 mmol, 1.0 equiv.), acrylic acid (137 µL, 2.00 mmol, 2.00 equiv.) and AcOH (57.2 µL, 1.00 mmol, 1.0 equiv.) were refluxed in water (4.0 mL, 0.25 M) for 5 h. The reaction was then cooled to RT on which a white precipitate formed (some precipitate may have been observed forming during the reaction). The precipitate was collected by vacuum filtration and washed with copious H_2O to give the title compound as a white solid (138 mg, 49%).

^{1}H NMR (400 MHz, MeOD) δ 9.10 (d, *J* = 2.0 Hz, 1H, Ar-*H*), 8.48 (dd, *J* = 8.5, 2.0 Hz, 1H, Ar-*H*), 8.28 (d, *J* = 8.0 Hz, 1H, Ar-*H*), 3.78 (t, *J* = 7.5 Hz, 2H, SO_2CH_2), 2.79 (t, *J* = 7.5 Hz, 2H, CH_2COOH); **^{13}C NMR** (126 MHz, MeOD) δ 173.5, 161.6, 148.5 (q, $^3J_{C-F}$ = 4.0 Hz), 137.9 (q, $^3J_{C-F}$ = 4.0 Hz), 131.2 (q, $^2J_{C-F}$ = 34.0 Hz), 124.3 (q, $^1J_{C-F}$ = 272.0 Hz), 123.6, 49.0 (overlaps with solvent peak), 28.5; **^{19}F NMR** (377 MHz, MeOD) δ -64.23 (s); **LRMS** (ESI$^-$) m/z 281.9 [M-H]$^-$; **HRMS** (ESI$^+$) found *m/z* 306.0019 [M+Na]$^+$, $C_9H_8O_4NF_3NaS$ requires m/z 306.0018; **IR** ν_{max} (neat)/cm^{-1} 3104, 3066,

2994, 2361, 2341, 1710, 1596, 1581, 1298, 1167, 1153, 1128, 1102, 1074, 1014, 867; **mp** 141-143 °C (AcOH: H_2O); **R$_f$** = 0.38 (80% EtOAc in Petrol).

Telescope synthesis:

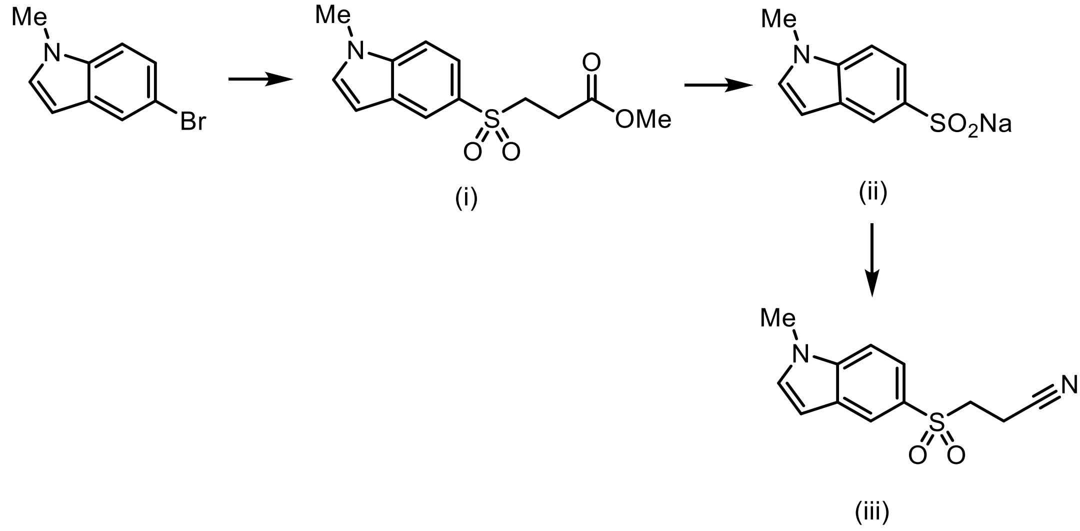

Route to synthesise 3-((1-methyl-1H-indol-5-yl)sulfonyl)propanenitrile (iii) (1k)

i)

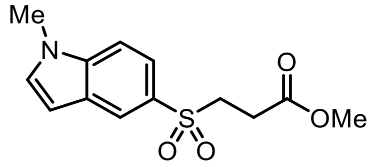

methyl 3-((1-methyl-1H-indol-5-yl)sulfonyl)propanoate

Following general procedure **E**, 5-bromo-1-methylindole (210.1 mg, 1.00 mmol, 1.0 equiv.), SMOPS reagent (522 mg, 3.00 mmol, 3.0 equiv.) and CuI (574 mg, 3.00 mmol, 3.0 equiv.) were heated at 110 °C in DMSO (2.0 mL, 0.5 M) for 16 h. The crude product was purified by flash column chromatography (20-50% EtOAc in Petrol) to the title compound as an off-white solid (262 mg, purity ≈ 80%, 75%).

The product was used in the next step without any further purification.

^{1}H NMR (400 MHz, $CDCl_3$) δ 8.22 (dd, *J* = 2.0, 0.5 Hz, 1H, Ar-*H*), 7.71 (dd, *J* = 8.5, 2.0 Hz, 1H, Ar-*H*), 7.45 (d, *J* = 9.0 Hz, 1H, Ar-*H*), 7.22 (d, *J* = 3.0 Hz, 1H, Ar-*H*), 6.65 (dd, *J* = 3.0, 1.0 Hz, 1H, Ar-*H*), 3.86 (s, 3H, N*Me*), 3.60 (s, 3H, CO_2*Me*), 3.45 (t, *J* = 8.0 Hz, 2H, SO_2CH_2), 2.75 (t, *J* = 8.0 Hz, 2H, CH_2CO_2Me); **^{13}C NMR** (101 MHz, $CDCl_3$) δ 170.7, 138.9, 131.5, 128.8, 128.0, 122.7, 120.6, 109.9, 102.9, 52.1, 52.0, 33.2, 28.0; **LRMS** (ESI$^+$) *m/z* 304.0 [M+Na]$^+$; **HRMS** (ESI$^+$) found *m/z* 282.0794 [M+H]$^+$, $C_{13}H_{16}O_4N_1S$ requires m/z 282.0795; **R$_f$** = 0.23 (60% EtOAc in Petrol).

ii)

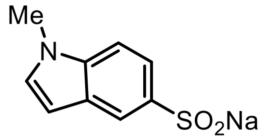

sodium 1-methyl-1H-indole-5-sulfinate

Following a modified literature procedure by Willis *et. al.*:[9] To a stirring solution of crude methyl 3-((1-methyl-1H-indol-5-yl)sulfonyl)propanoate (250 mg, 0.89 mmol, 1.0 equiv.) in MeOH (1.0 mL, 1.0 M) was added NaOMe solution (25% in MeOH) (204 µL, 0.89 mmol, 1.0 equiv.). The reaction was monitored by TLC. After 30 min, the reaction mixture was concentrated *in vacuo* to give the crude title compound as a white solid. The crude product was used directly in the next step.

iii)

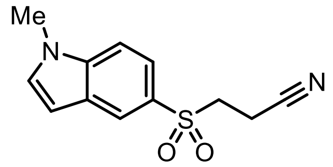

3-((1-methyl-1H-indol-5-yl)sulfonyl)propanenitrile (1k)

Following a modified version of general procedure **D**, crude sodium 1-methyl-1H-indole-5-sulfinate (193 mg, 0.890 mmol, 1.0 equiv.), acrylonitrile (88 µL, 1.34 mmol, 1.5 equiv.) and AcOH (76 µL, 1.34 mmol, 1.5 equiv.) were refluxed in water (2.0 mL, 0.4 M) for 7 h. After workup, the resultant crude product was purified by flash chromatography (40% EtOAc in Petrol) to give the title compound as a white solid (152 mg, 69%).

^{1}H NMR (400 MHz, $CDCl_3$) δ 8.22 (d, *J* = 2.0 Hz, 1H, Ar-*H*), 7.69 (dd, *J* = 9.0, 2.0 Hz, 1H, Ar-*H*), 7.47 (d, *J* = 9.0 Hz, 1H, Ar-*H*), 7.24 (d, *J* = 3.0 Hz, 1H, Ar-*H*), 6.66 (dd, *J* = 3.0, 1.0 Hz, 1H, Ar-*H*), 3.87 (s, 3H, N-*Me*), 3.39 (t, *J* = 8.0 Hz, 2H, SO_2CH_2), 2.78 (t, *J* = 8.0 Hz, 2H, CH_2CN); **^{13}C NMR** (101 MHz, $CDCl_3$) δ 139.4, 132.1, 128.4, 127.7, 123.0, 120.6, 116.4, 110.4, 103.2, 51.8, 33.4, 12.4; **HRMS** (ESI^+) found *m/z* 249.0693 $[M+H]^+$, $C_{12}H_{13}O_2N_2S$ requires m/z 249.0692; **IR** ν_{max} (neat)/cm^{-1} 2990, 2943, 2518, 2247, 1735, 1606, 1517, 1482, 1423, 1344, 1297, 1260, 1240, 1210, 1165, 1131, 1105, 1062, 983, 911, 799, 771, 575, 737, 628, 616; **mp** 104-105 °C (EtOAc); **R_f** = 0.19 (50% EtOAc in Petrol).

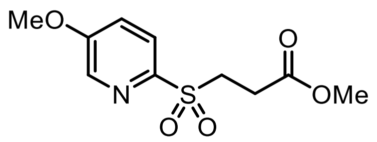

methyl 3-((5-methoxypyridin-2-yl)sulfonyl)propanoate (4o)

Following General procedure **E**, and 2-bromo-5-methoxypyridine (188 mg, 1.00 mmol, 1.0 equiv.), SMOPS reagent (522 mg, 3.00 mmol, 3.0 equiv.) and CuI (570 mg, 3.00 mmol, 3.0 equiv.) in DMSO (2.0 mL, 0.5 M) were heated at 110 °C for 16 h. The crude product was purified by flash column chromatography (EtOAc: CH_2Cl_2: toluene = 5:45:50) to give the title compound as a colourless oil (124 mg, 48%).

^{1}H NMR (400 MHz, $CDCl_3$) δ 8.37 (dd, *J* = 3.0 Hz, 0.5 Hz, 1H, Ar-*H*), 8.02 (d, *J* = 8.5 Hz, 1H, Ar-*H*), 7.34 (dd, *J* = 8.5 Hz, 3.0 Hz, 1H, Ar-*H*), 3.94 (s, 3H, OCH_3), 3.67-3.62 (m, 5H, CO_2CH_3 and $SO_2CH_2CH_2$), 2.82-2.78 (m, 2H, CH_2CO_2); **^{13}C NMR** (101 MHz, $CDCl_3$) δ 170.7, 158.7, 148.2, 139.2, 124.2, 120.4, 56.2, 52.4, 48.3, 27.6; **LRMS** (ESI$^+$) *m/z* 282.0 $[M+Na]^+$; **HRMS** (ESI$^+$) found *m/z* 260.0589 $[M+H]^+$, $C_{10}H_{14}NO_5S$ requires m/z 260.0587; **IR** ν_{max} (neat)/cm^{-1} 2981, 2954, 1736, 1574, 1313, 1160, 980, 836; **R$_f$** = 0.25 (EtOAc: CH_2Cl_2: toluene = 15:45:50). Data is consistent with literature: A. de Gombert, A. I. Mckay, C. J. Davis, K. M. Wheelhouse and M. C. Willis *J. Am. Chem. Soc,.* 2020, **142**, 7, 3564-3576.[10]

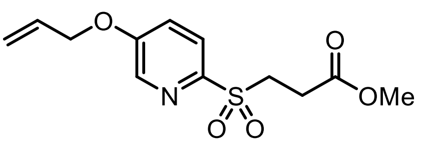

methyl 3-((5-(allyloxy)pyridin-2-yl)sulfonyl)propanoate (6)

Following General procedure **E**, and 5-(allyloxy)-2-bromopyridine (359.0 mg, 1.67 mmol, 1.0 equiv.), SMOPS reagent (435.4 mg, 2.50 mmol, 1.5 equiv.) and CuI (476.1 mg, 2.50 mmol, 1.5 equiv.) in DMSO (3.3 mL, 0.5 M) were heated at 110 °C for 24 h. The crude product was purified by flash column chromatography (30% EtOAc in Petrol) to give the title compound as a slightly yellow oil that crystallised on standing to an off-white crystalline solid (181.4 mg, 38%).

^{1}H NMR (400 MHz, $CDCl_3$) δ 8.40 (d, *J* = 3.0 Hz, 1H, Pyr-*H*), 8.02 (d, *J* = 8.5 Hz, 1H, Pyr-*H*), 7.34 (dd, *J* = 8.5, 3.0 Hz, 1H, Pyr-*H*), 6.03 (ddd, *J* = 17.0, 10.5, 5.5 Hz, 1H, CH_aH_b=C*H*), 5.45 (d, *J* = 17.0 Hz, 1H, C$H_a$$H_b$=CH), 5.39 (d, *J* = 10.5 Hz, 1H, $CH_a$$H_b$=CH), 4.68 (app. dt, *J* = 5.5, 2.0 Hz, 2H, CH_2O), 3.70 – 3.63 (m, 5H, CH_3 and SO_2CH_2), 2.82 (t, 2H, *J* = 8.0 Hz, CH_2CO_2Me); **^{13}C NMR** (101 MHz, $CDCl_3$) δ 170.7, 157.7, 148.4, 139.6, 131.3, 124.2, 121.4, 119.5, 69.8, 52.4, 48.3, 27.6; **LRMS** (ESI$^+$) *m/z* 308.0 $[M+Na]^+$; **HRMS** (ESI$^+$) found *m/z* 286.0740 $[M+H]^+$, $C_{12}H_{16}O_5NS$ requires m/z 288.0744; **IR** ν_{max} (neat)/cm^{-1} 3086, 3023, 2995, 2953, 2854, 1737, 1571, 1452, 1426, 1365, 1313, 1277, 1247, 1200, 1160, 1131, 1099, 1059, 1059, 1013, 932, 900, 835, 797, 773, 753; **mp** 26-28 °C (EtOAc); **R$_f$** = 0.2 (30% EtOAc in Petrol).

3.2.c Synthesis of sulfinates

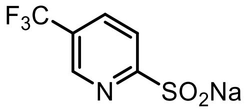

sodium 5-(trifluoromethyl)pyridine-2-sulfinate (15a)

Following a procedure adapted from Sato *et. al.*: [11] (All other sulfinates used were commercial or were previously prepared using the same method)

Open to atmosphere: To a solution of NaOH (98.0 mg, 2.45 mmol, 0.98 equiv.) in 1:1 Ethanol: H_2O (22.0 mL, 0.1 M) was added 5-(trifluoromethyl)pyridine-2-thiol (899 mg, 5.00 mmol, 1.0 equiv.). Hydrogen peroxide solution (30% w/w H_2O) (382 µL, 3.75 mmol, 1.5 equiv.) was added dropwise to the stirring yellow solution at RT and the reaction was stirred at RT for 18 h. The reaction mixture was then concentrated *in vacuo.* The resultant solid washed with EtOAc (15 mL × 5) and dried to give the title product as a white solid (441 mg, 77%).

^{1}H NMR (400 MHz, MeOD) δ 8.85 – 8.81 (s, 1H, Ar-*H*), 8.26 – 8.21 (dd, *J* = 8.0, 2.0 Hz, 1H, Ar-*H*), 8.05 (d, *J* = 8.0 Hz, 1H, Ar-*H*); **^{13}C NMR** (126 MHz, MeOD) δ 178.7, 146.7 (q, $^3J_{C\text{-}F}$ = 4.0 Hz), 136.4 (q, $^3J_{C\text{-}F}$ = 3.5 Hz), 128.1 (q, $^2J_{C\text{-}F}$ = 33.0 Hz), 124.9 (q, $^2J_{C\text{-}F}$ = 271.0 Hz), 119.1; **^{19}F NMR** (377 MHz, MeOD) δ -63.85 (s); **HRMS** (ESI$^-$) found *m/z* 209.9837 [M-Na]$^-$, $C_6H_3O_2NF_3S$ requires m/z 209.9842; **IR** v_{max} (neat)/cm^{-1} 3380, 1624, 1595, 1376, 1333, 1159, 1130, 1105, 1066, 1020, 1001, 984, 852, 767, 708, 623; **mp** >250 °C (EtOH: H_2O); **R$_f$** = 0.00 (60% EtOAc in Petrol). Data consistent with literature: T. Markovic, B. N. Rocke, D. C. Blakemore, V. Mascitti and M. C. Willis, *Chem. Sci.*, 2017, **8**, 4437-4442.[9]

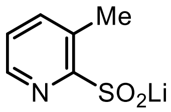

lithium 3-methylpyridine-2-sulfinate (15b)

Following a procedure adapted from Markovic *et. al.:*[9]

*n*BuLi solution (1.62 mL, 2.2 M in hexane, 3.60 mmol, 0.9 equiv.) was slowly added to a stirring solution of 2-bromo-3-methylpyridine (446 µL, 4.00 mmol, 1.0 equiv.) in THF (20.0 mL, 0.2 M) at -78 °C. The reaction mixture was stirred for 1 hour before TIMSO (514.5 µL, 4.0 mmol, 1.0 equiv., den: 1.16 g/cm^3) was added dropwise. The reaction was then allowed to warm to room temperature. The crude product was collected by vacuum filtration. The product was then purified by washing with acetone and diethyl ether and drying under vacuum to yield the title compound as a pale yellow solid (552.1 mg, 94%).

^{1}H NMR (400 MHz, D_2O) δ 8.41 (d, *J* = 4.5 Hz, 1H, Pyr-*H*), 7.78 – 7.72 (dq, *J* = 7.5, 1.0 Hz, 1H, Pyr-*H*), 7.43 (dd, *J* = 7.5, 4.5 Hz, 1H, Pyr-*H*), 2.61 (s, 3H, Pyr-*Me*); **^{13}C NMR** (101 MHz, D_2O) δ 165.7, 146.2, 141.0, 131.5, 125.4, 15.7; **LRMS** (ESI$^-$) *m/z* 155.9 [M-Li]$^-$; **HRMS** (ESI$^-$) found *m/z*

156.0117[M-Na]$^-$, C_6H6O_2NS requires m/z 156.0125; **IR** ν_{max} (neat)/cm^{-1} 3388, 2957, 2359, 1652, 1573, 1449, 1405, 1326, 1202, 1111, 976, 799, 754, 682, 642; **mp** decompose >250 °C.

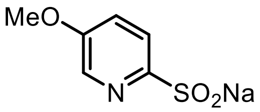

sodium 5-methoxypyridine-2-sulfinate (15c)

Following a modified literature procedure by Markovic *et. al.*:[11]

To a stirring solution of crude methyl 3-((5-methoxypyridin-2-yl)sulfonyl)propanoate, (520 mg, 2.00 mmol, 1.0 equiv.) in MeOH (2.25 mL, 1.0 M) was added NaOMe solution (25% in MeOH) (457 µL, 2.00 mmol, 1.0 equiv.) . The reaction was monitored by TLC (note: the by-product thiol has a strong odour). After 30 mins the reaction mixture was concentrated *in vacuo* to give the crude title compound as a white solid. The crude sulfinate product was used directly in the next step.

$\mathbf{R_f}$ = 0.0 (30% EtOAc in Petrol).

3.3 Heteroaromatic desulfinative cross-coupling

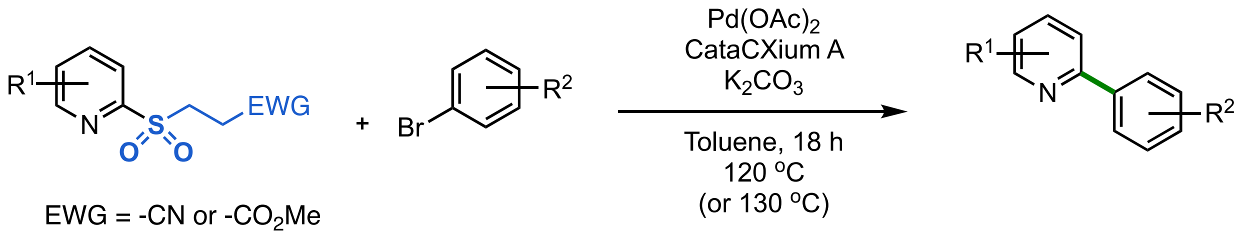

CAUTION: SO_2 is formed during the reaction.

General Coupling procedure F or the pyridine β-nitrile sulfone reagent with aryl halides: To an oven-dried microwave vial (10 mL) was added pyridine β-nitrile sulfone (0.22 mmol, 1.1 equiv.), aryl halide (if it is a solid) (0.20 mmol, 1.0 equiv.), $Pd(OAc)_2$ (2.2 mg, 0.010 mmol, 5.0 mol%), CataCXium A® (7.2 mg, 0.020 mmol, 10 mol%) and K_2CO_3 (41.4 mg, 0.30 mmol, 1.5 equiv.). The vial was capped and purged with N_2 for 10 mins. Dry toluene (2.0 mL, 0.1 M) was then added, followed by the addition of aryl halide (if it is a liquid) (0.20 mmol, 1.0 equiv.) and AcOH (11.5 µL, 0.20 mmol, 1.0 equiv.) with stirring. The reaction mixture was heated to 120 °C or 130 °C for 18 h. The reaction mixture was then allowed to cool to RT, filtered through a short pad of silica and washed with EtOAc. The resultant crude solution was concentrated *in vacuo*, adsorbed onto celite and purified by flash column chromatography (EtOAc in Petrol) to give the desired product.

General Coupling procedure G for the pyridine β-methylester sulfone reagent with aryl halides:

To an oven dried microwave vial (10 mL) was added pyridine β-methylester sulfone (0.30 mmol, 1.5 equiv.), aryl halide (if it is a solid) (0.20 mmol, 1.0 equiv.), $Pd(OAc)_2$ (2.2 mg, 0.010 mmol, 5.0 mol%), CataCXium A (7.2 mg, 0.020 mmol, 10 mol%) and K_2CO_3 (55.3 mg, 0.40 mmol, 2.0 equiv.). The vial was capped and purged with N_2 for 10 mins. Dry toluene (2.0 mL, 0.1 M) was then added, followed by the addition of aryl halide (if it is a liquid) (0.20 mmol, 1.0 equiv.) with stirring. The reaction mixture was heated to 120 °C or 130 °C for 18 h. The reaction mixture was then allowed to cool to RT, filtered through a pad of silica and washed with EtOAc. The resultant crude solution was concentrated *in vacuo*, adsorbed onto celite and purified by flash column chromatography (EtOAc in Petrol) to give the desired product.

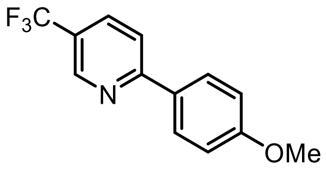

2-(4-methoxyphenyl)-5-(trifluoromethyl)pyridine (3a)

From the β-nitrile sulfone: Following general procedure **F**, 3-((5-(trifluoromethyl)pyridin-2-yl)sulfonyl)propanenitrile (58.1 mg, 0.22 mmol, 1.1 equiv.), 4-bromoanisole (25 µL, 0.20 mmol, 1.0 equiv.), $Pd(OAc)_2$ (2.2 mg, 0.010 mmol, 5.0 mol%), K_2CO_3 (41.4 mg, 0.30 mmol, 1.5 equiv.), CataCXium A (7.2 mg, 0.020 mmol, 10 mol%) and AcOH (11.5 µL, 0.20 mmol, 1.0 equiv.) in toluene (2.0 mL) were heated at 120 °C for 18 h. The crude reaction mixture was purified by flash column chromatography on silica gel (3% EtOAc in Petrol) to give the title product as a white solid (44.8 mg, 88%).

From the β-methylester sulfone (for comparison to the β-nitrile sulfone): Following general procedure **F**, methyl 3-((5-(trifluoromethyl)pyridin-2-yl)sulfonyl)propanoate (65.4 mg, 0.22 mmol, 1.1 equiv.), 4-bromoanisole (25 µL, 0.20 mmol, 1.0 equiv.), $Pd(OAc)_2$ (2.2 mg, 0.010 mmol, 5.0 mol%), K_2CO_3 (41.4 mg, 0.30 mmol, 1.5 equiv.), CataCXium A (7.2 mg, 0.020 mmol, 10 mol%) and AcOH (11.5 µL, 0.20 mmol, 1.0 equiv.) in toluene (2.0 mL) were heated at 120 °C for 18 h. The crude reaction mixture was purified by flash column chromatography on silica gel (3% EtOAc in Petrol) to give the title product as a white solid (36.0 mg, 71%).

From the β-methylester sulfone: Following general procedure **G**, methyl 3-((5-(trifluoromethyl)pyridin-2-yl)sulfonyl)propanoate (89.2 mg, 0.30 mmol, 1.5 equiv.), 4-bromoanisole (25 µL, 0.20 mmol, 1.0 equiv.), $Pd(OAc)_2$ (2.2 mg, 0.010 mmol, 5.0 mol%), K_2CO_3 (55.3 mg, 0.40 mmol, 2.0 equiv.) and CataCXium A (7.2 mg, 0.020 mmol, 10 mol%) in toluene (2.0 mL) were heated at 120 °C for 18 h. The crude reaction mixture was purified by flash purified by column chromatography on silica gel (3% EtOAc in Petrol) to give the title product as a white solid (44.4 mg, 87%).

From the diethyl succinate sulfone:

Following general procedure **G**, diethyl 2-((5-(trifluoromethyl)pyridin-2-yl)sulfonyl)succinate (115.0 mg, 0.30 mmol, 1.5 equiv.), 4-bromoanisole (25 µL, 0.20 mmol, 1.0 equiv.), $Pd(OAc)_2$ (2.2 mg, 0.010 mmol, 5.0 mol%), K_2CO_3 (55.3 mg, 0.40 mmol, 2.0 equiv.) and CataCXium A (7.2 mg, 0.020 mmol, 10 mol%) in toluene (2.0 mL) were heated at 120 °C for 18 h. The crude reaction mixture was purified by flash column chromatography on silica gel (3% EtOAc in Petrol) to give the title product as a white solid (42.1 mg, 83%). *When run at 130 °C, the reaction gave the title product in a 72% yield (36.5 mg).*

^{1}H NMR (400 MHz, $CDCl_3$) δ 8.89 (app. s, 1H, Pyr-*H*), 8.01 (d, *J* = 9.0 Hz, 2H, Ar-*H*), 7.93 (dd, *J* = 8.5, 2.5 Hz, 1H, Pyr- *H*), 7.78 (d, *J* = 8.5 Hz, 1H, Pyr- *H*), 7.03 (app. dt, *J* = 9.0 Hz, 2H, Ar-*H*), 3.88 (s, 3H, OCH_3); **^{13}C NMR** (101 MHz, $CDCl_3$) 161.5, 160.4, 146.6 (q, $^3J_{C-F}$ = 4.0 Hz), 133.9 (q, $^3J_{C-F}$ = 4.0 Hz), 130.6, 128.8, 124.2 (q, $^2J_{C-F}$ = 34.0 Hz), 123.9 (q, $^1J_{C-F}$ = 272.0 Hz), 119.2, 114.5, 55.6; **^{19}F NMR** (377 MHz, $CDCl_3$) δ -62.15 (s); **LRMS** (ESI^+) *m/z* 254.0 $[M+H]^+$; **HRMS** (ESI^+) found *m/z* 254.0781 $[M+H]^+$, $C_{13}H_{11}ONF_3$ requires m/z 254.0787; **IR** ν_{max} (neat)/cm^{-1} 3031, 2970, 2043, 1598, 1319, 1299, 1283, 1249, 1114, 1084, 824, 774, 711; **mp** 111-113 °C (EtOAc); **R$_f$** = 0.44 (20% EtOAc in Petrol). Retention time on HPLC: 6.63 min. Data Consistent with literature: J. Wei, H. Liang, C. Ni, R. Sheng, J. Hu, *Org. Lett.,* 2019, **21**, 4, 937-940.[1]

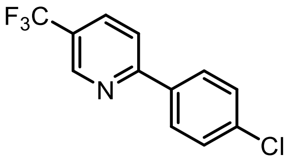

2-(4-chlorophenyl)-5-(trifluoromethyl)pyridine (3b)

From the β-nitrile sulfone: Following general procedure **F**, 3-((5-(trifluoromethyl)pyridin-2-yl)sulfonyl)propanenitrile (58.1 mg, 0.22 mmol, 1.1 equiv.), 1-bromo-4-chlorobenzene (38.3 mg, 0.20 mmol, 1.0 equiv.), $Pd(OAc)_2$ (2.2 mg, 0.010 mmol, 5.0 mol%), K_2CO_3 (41.4 mg, 0.30 mmol, 1.5 equiv.), CataCXium A (7.2 mg, 0.020 mmol, 10 mol%) and AcOH (11.5 µL, 0.20 mmol, 1.0 equiv.) in toluene (2.0 mL) were heated at 120 °C for 18 h. The crude reaction mixture was purified by flash column chromatography on silica gel (1% EtOAc in Petrol) to give the title product as an off-white crystalline solid (41.0 mg, 80%).

From the β-methylester sulfone: Following general procedure **G**, methyl 3-((5-(trifluoromethyl)pyridin-2-yl)sulfonyl)propanoate (89.2 mg, 0.30 mmol, 1.5 equiv.), 1-bromo-4-chlorobenzene (38.3 mg, 0.20 mmol, 1.0 equiv.), $Pd(OAc)_2$ (2.2 mg, 0.010 mmol, 5.0 mol%), K_2CO_3 (55.3 mg, 0.40 mmol, 2.0 equiv.) and CataCXium A (7.2 mg, 0.020 mmol, 10 mol%) in toluene (2.0 mL) were heated at 120 °C for 18 h. The crude reaction mixture was purified by flash column chromatography on silica gel (1% EtOAc in Petrol) to give the title product as an off-white solid (36.5 mg, 71%).

^{1}H NMR (400 MHz, $CDCl_3$) δ 8.95 – 8.92 (m, 1H, Pyr-*H*), 8.01 – 7.97 (m, 3H, Ar-*H* and Pyr-*H* overlap), 7.82 (d, *J* = 8.5 Hz, 1H, Pyr-*H*), 7.50 – 7.46 (m, 2H, Ar-*H*); **^{13}C NMR** (101 MHz, $CDCl_3$) δ 159.5, 146.8 (q, $^3J_{C-F}$ = 4.0 Hz), 136.5, 136.4, 134.2 (q, $^3J_{C-F}$ = 4.0 Hz), 129.3, 128.7, 125.2 (q, $^2J_{C-F}$ = 33.0), 123.8 (q, $^1J_{C-F}$ = 272.0), 119.9; **^{19}F NMR** (377 MHz, $CDCl_3$) δ -62.28 (s); **LRMS** (ESI^+) *m/z* 258.0 $[M+H]^+$; **HRMS** (ESI^+) found *m/z* 258.0292 $[M+H]^+$, $C_{12}H_8N^{35}ClF_3$ requires m/z 258.0292; **IR** ν_{max} (neat)/cm^{-1} 1595, 1477, 1379, 1337, 1134, 1092, 1014, 829, 768; **mp** 72-

75 °C (EtOAc: Petrol); **R$_f$** = 0.53 (10% EtOAc in Petrol). Data is consistent with literature: Y. Xie, H. Chi, A. Guan, C. Liu, H. Ma, D. Cui. *J. Agric. Food Chem.* 2014, **62**, 52, 12491-12496.[12]

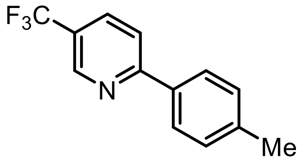

2-(p-tolyl)-5-(trifluoromethyl)pyridine (3c)

From the β-nitrile sulfone: Following general procedure **F**, 3-((5-(trifluoromethyl)pyridin-2-yl)sulfonyl)propanenitrile (58.1 mg, 0.22 mmol, 1.1 equiv.), 4-bromotoluene (34.2 mg, 0.20 mmol, 1.0 equiv.), Pd(OAc)$_2$ (2.2 mg, 0.010 mmol, 5.0 mol%), K_2CO_3 (41.4 mg, 0.30 mmol, 1.5 equiv.), CataCXium A (7.2 mg, 0.020 mmol, 10 mol%) and AcOH (11.5 µL, 0.20 mmol, 1.0 equiv.) in toluene (2.0 mL) were heated at 120 °C for 18 h. The crude reaction mixture was purified by flash column chromatography on silica gel (1% EtOAc in Petrol) to give the title product as a white crystalline solid (38.0 mg, 80%). *When run at 130 °C, the reaction gave the title product in 89% yield (42.3 mg)*

^{1}H NMR (400 MHz, $CDCl_3$) δ 8.93 – 8.91 (m, 1H, Pyr-*H*), 7.98 – 7.91 (m, 3H, Ar-*H* and Pyr-*H* overlap), 7.82 (d, *J* = 8.5 Hz, 1H, Pyr-*H*), 7.32 (d, *J* = 8.0 Hz, 2H, Ar-*H*), 2.43 (s, 3H, C*H*$_3$); **^{13}C NMR** (101 MHz, $CDCl_3$) δ 160.8, 146.7 (q, $^3J_{C-F}$ = 4.0 Hz), 140.5, 135.3, 134.0 (q, $^3J_{C-F}$ = 3.5 Hz), 129.9, 127.3, 124.5 (q, $^2J_{C-F}$ = 32.0 Hz), 123.8 (q, $^1J_{C-F}$ = 272.0 Hz), 119.8, 21.5; **^{19}F NMR** (377 MHz, $CDCl_3$) δ -62.18 (s); **LRMS** (ESI$^+$) *m/z* 238.0 $[M+H]^+$; **HRMS** (ESI$^+$) found *m/z* 238.0839 $[M+H]^+$, $C_{13}H_{11}NF_3$ requires m/z 238.0838; **IR** ν_{max} (neat)/cm^{-1} 1713, 1599, 1332, 1224, 1124, 1014, 914, 823, 732; **mp** 101-103 °C (EtOAc); **R$_f$** = 0.53 (10% EtOAc in Petrol). Data is consistent with literature: T. Markovic, B. N. Rocke, D. C. Blakemore, V. Mascitti and M. C. Willis, *Chem. Sci.*, 2017, **8**, 4437-4442.[9]

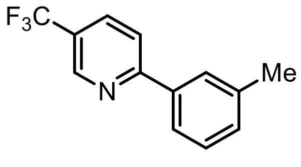

2-(m-tolyl)-5-(trifluoromethyl)pyridine (3d)

From the β-nitrile sulfone: Following general procedure **F**, 3-((5-(trifluoromethyl)pyridin-2-yl)sulfonyl)propanenitrile (58.1 mg, 0.22 mmol, 1.1 equiv.), Pd(OAc)$_2$ (2.2 mg, 0.010 mmol, 5.0 mol%), CataCXium A (7.2 mg, 0.020 mmol, 10 mol%), K_2CO_3 (41.4 mg, 0.30 mmol, 1.5 equiv.), 3-bromotoluene (24 µL, 0.20 mmol, 1.0 equiv.) and AcOH (11.4 µL, 0.20 mmol, 1.0 equiv.) in toluene (2.0 mL) were heated at 120 °C for 18 h. The crude reaction mixture was purified by flash column chromatography (0-0.5% EtOAc in Petrol) to give the title compound as a yellow solid (45.0 mg, 95%).

^{1}H NMR (400 MHz, $CDCl_3$) δ 8.95 – 8.94 (m, 1H, Ar-*H*), 7.97 (dd, *J* = 8.5 Hz, 2.5 Hz, 1H, Ar-*H*), 7.88 – 7.80 (m, 3H, Ar-*H*), 7.40 (t, *J* = 7.5 Hz, 1H, Ar-*H*), 7.30 (br d, *J* = 7.5 Hz, 1H, Ar-*H*), 2.46 (s, 3H, Ar-C*H$_3$*); **^{13}C NMR** (101 MHz, $CDCl_3$) δ 161.0, 146.7 (q, $^{3}J_{C-F}$ = 4.0 Hz), 138.9, 138.1, 134.0 (q, $^{3}J_{C-F}$ = 3.5 Hz), 130.1, 129.0, 128.1, 124.9 (q, $^{2}J_{C-F}$ = 32.0 Hz), 124.5, 123.9 (q, $^{1}J_{C-F}$ = 272.0 Hz), 120.2, 21.6; **^{19}F NMR** (377 MHz, $CDCl_3$) δ -62.23 (s); **LRMS** (ESI$^+$) *m/z* 238.0 [M+H]$^+$; **HRMS** (ESI$^+$) found *m/z* 238.0839 [M+H]$^+$, $C_{13}H_{11}F_3N$ requires m/z 238.0838; **IR** ν_{max} (neat)/cm^{-1} 3031, 2926, 1605, 1326, 1124, 1083, 1014, 760; **mp** 60-65 °C (CH_2Cl_2); **R$_f$** = 0.22 (1% EtOAc in Petrol). Data is consistent with literature: J. Wei, H. Liang, C. Ni, R. Sheng and J. Hu, *Org. Lett.* 2019, **21**, 4, 937-940.[1]

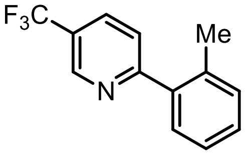

2-(o-tolyl)-5-(trifluoromethyl)pyridine (3e)

From the β-nitrile sulfone: Following general procedure **F**, 3-((5-(trifluoromethyl)pyridin-2-yl)sulfonyl)propanenitrile (58.1 mg, 0.22 mmol, 1.1 equiv.), 2-bromotoluene (24.1 µL, 0.20 mmol, 1.0 equiv.), $Pd(OAc)_2$ (2.2 mg, 0.010 mmol, 5.0 mol%), K_2CO_3 (41.4 mg, 0.30 mmol, 1.5 equiv.), CataCXium A (7.2 mg, 0.020 mmol, 10 mol%) and AcOH (11.5 µL, 0.20 mmol, 1.0 equiv.) in toluene (2.0 mL) were heated at 130 °C for 18 h. The crude reaction mixture was purified by flash column chromatography on silica gel (3% EtOAc in Petrol) to give the title product as a pale yellow oil (43.1 mg, 91%). *When run at 120 °C, the reaction gave the title product in a 56% yield (26.5 mg)*

^{1}H NMR (400 MHz, $CDCl_3$) δ 8.97 (dq, *J* = 2.5, 1.0 Hz, 1H, Pyr-*H*), 7.99 (dd, *J* = 8.0, 2.5 Hz, 1H, Pyr-*H*), 7.55 (d, *J* = 8.0 Hz, 1H, Pyr-*H*), 7.43 – 7.28 (m, 4H, Ar-*H*), 2.39 (s, 3H, C*H$_3$*); **^{13}C NMR** (101 MHz, $CDCl_3$) δ 163.7, 146.2 (q, $^{3}J_{C-F}$ = 4.0 Hz), 139.2, 136.0, 133.5 (q, $^{3}J_{C-F}$ = 4.0 Hz), 131.2, 129.8, 129.2, 126.2, 124.7 (q, $^{2}J_{C-F}$ = 33.0 Hz), 123.9 (q, $^{1}J_{C-F}$ = 273.0 Hz), 123.9, 20.4; **^{19}F NMR** (377 MHz, $CDCl_3$) δ -62.21 (s); **LRMS** (ESI$^+$) *m/z* 238.0 [M+H]$^+$; **HRMS** (ESI$^+$) found *m/z* 238.0838 [M+H]$^+$, $C_{13}H_{11}NF_3$ requires m/z 238.0838; **IR** ν_{max} (neat)/ cm^{-1} 3065, 3025, 2928, 2927, 1605, 1564, 1480, 1457, 1384, 1324, 1271, 1238, 1163, 1123, 1081, 1014, 940, 850, 816, 785, 751, 721, 682, 637, 619; **R$_f$** = 0.46 (5% EtOAc in Petrol). Data is consistent with literature: J. Wei, H. Liang, C. Ni, R. Sheng, J. Hu, *Org. Lett.,* 2019, **21**, 14, 937-940.[1]

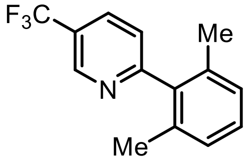

2-(2,6-dimethylphenyl)-5-(trifluoromethyl)pyridine (3f)

From the β-nitrile sulfone: Following general procedure **F**, 3-((5-(trifluoromethyl)pyridin-2-yl)sulfonyl)propanenitrile (58.1 mg, 0.22 mmol, 1.1 equiv.), 2-bromo-1,3-dimethylbenzene (26.6 µL, 0.20 mmol, 1.0 equiv.), $Pd(OAc)_2$ (2.2 mg, 0.010 mmol, 5.0 mol%), K_2CO_3 (41.4 mg, 0.30 mmol, 1.5 equiv.), CataCXium A (7.2 mg, 0.020 mmol, 10 mol%) and AcOH (11.5 µL, 0.20 mmol, 1.0 equiv.) in toluene (2.0 mL) were heated at 130 °C for 18 h. The crude reaction mixture was purified by flash column chromatography on silica gel (2% EtOAc in Petrol) to give the title product as yellow oil (32.8 mg, 65%).

^{1}H NMR (400 MHz, $CDCl_3$) δ 9.01 (m, 1H, Pyr-*H*), 8.01 (app. ddd, *J* = 8.0, 2.5, 1.0 Hz, 1H, Pyr-*H*), 7.39 (app. dt, *J* = 8.0, 1.0 Hz, 1H, Pyr-*H*), 7.23 (dd, *J* = 8.0, 7.0 Hz, 1H, Ar-*H*), 7.13 (app. d, *J* = 7.5 Hz, 2H, Ar-*H*), 2.04 (s, 6H, $(CH_3)CCC(CH_3)$); **^{13}C NMR** (101 MHz, $CDCl_3$) δ 163.9, 146.8 (q, $^3J_{C-F}$ = 4.0 Hz), 139.4, 135.7, 133.6 (q, $^3J_{C-F}$ = 3.5 Hz), 128.6, 127.9, 124.9 (q, $^2J_{C-F}$ = 33.0 Hz), 124.5, 123.8 (q, J^1_{C-F} = 272.5 Hz), 20.3; **^{19}F NMR** (377 MHz, $CDCl_3$) δ -62.25 (s); **LRMS** (ESI^+) m/z 252.0 $[M+H]^+$**;** **HRMS** (ESI^+) found *m/z* 250.0995 $[M+H]^+$, $C_{14}H_{13}NF_3$ requires m/z 252.0995; **IR** v_{max} (neat)/cm^{-1} 3025, 2924, 2361, 1607,1567, 1466, 1382, 1326, 1282, 1233, 1162, 1127, 1080, 1015, 940, 850, 771, 746, 698, 637; **R_f** = 0.33 (5% EtOAc in Petrol). Data is consistent with literature: T. Markovic, B. N. Rocke, D. C. Blakemore, V. Mascitt, M. C. Willis, *Org. Lett.* 2017, **19**, 22, 6033-6035.[13]

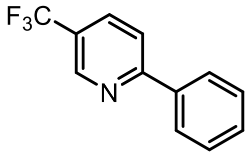

2-phenyl-5-(trifluoromethyl)pyridine (3g)

From the β-nitrile sulfone: Following general procedure **F**, 3-((5-(trifluoromethyl)pyridin-2-yl)sulfonyl)propanenitrile (58.1 mg, 0.22 mmol, 1.1 equiv.), bromobenzene (21.3 µL, 0.20 mmol, 1.0 equiv.), $Pd(OAc)_2$ (2.2 mg, 0.010 mmol, 5.0 mol%), K_2CO_3 (41.4 mg, 0.30 mmol, 1.5 equiv.), CataCXium A (7.2 mg, 0.020 mmol, 10 mol%) and AcOH (11.5 µL, 0.20 mmol, 1.0 equiv.) in toluene (2.0 mL) were heated at 130 °C for 18 h. The crude product was purified by flash column chromatography on silica gel (1% EtOAc in Petrol) to give the title product as a white crystalline solid (41.0 mg, 91%). *When run at 120 °C, the reaction gave the title product in a 54% yield (24.1 mg)*

^{1}H NMR (400 MHz, $CDCl_3$) δ 8.96 – 8.95 (m, 1H, Pyr-*H*), 8.06 – 8.02 (m, 2H, Ar-*H*), 7.99 (dd, *J* = 8.5, 2.5 Hz, 1H, Pyr-*H*), 7.85 (d, *J* = 8.5 Hz, 1H, Pyr-*H*), 7.56 – 7.45 (m, 3H, Ar-*H*); **^{13}C NMR** (101 MHz, $CDCl_3$) δ 160.8, 146.8 (q, $^3J_{C\text{-}F}$ = 4.0 Hz), 138.1, 134.1 (q, $^3J_{C\text{-}F}$ = 3.5 Hz), 130.2, 129.1, 127.4, 125.0 (q, $^2J_{C\text{-}F}$ = 33.0 Hz), 123.9 (q, $^1J_{C\text{-}F}$ = 272.0 Hz), 120.1; **^{19}F NMR** (377 MHz, $CDCl_3$) δ -62.21 (s); **LRMS** (ESI$^+$) *m/z* 223.9 [M+H]$^+$; **HRMS** (ESI$^+$) found *m/z* 224.0681 [M+H]$^+$, $C_{12}H_9NF_3$ requires m/z 224.0682; **IR** v_{max} (neat)/cm^{-1} 2925, 2923, 2360, 1711, 1601, 1481, 1449, 1387, 1337, 1238, 1165, 1123, 1087, 1013, 939, 839, 790, 739, 648; **mp** 64-66 °C (EtOAc); **R$_f$** = 0.50 (5% EtOAc in Petrol). Data is consistent with literature: J. Wei, H. Liang, C. Ni, R. Sheng, J. Hu, *Org. Lett.,* 2019, **21**, 4, 937-940.[1]

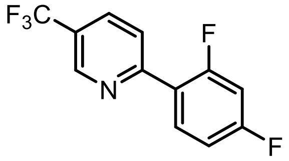

2-(2,4-difluorophenyl)-5-(trifluoromethyl)pyridine (3h)

From the β-nitrile sulfone: Following general procedure **F**, 3-((5-(trifluoromethyl)pyridin-2-yl)sulfonyl)propanenitrile (58.1 mg, 0.22 mmol, 1.1 equiv.), 1-bromo-2,4-difluorobenzene (22.6 µL, 0.20 mmol, 1.0 equiv.), $Pd(OAc)_2$ (2.2 mg, 0.010 mmol, 5.0 mol%), K_2CO_3 (41.4 mg, 0.30 mmol, 1.5 equiv.), CataCXium A (7.2 mg, 0.020 mmol, 10 mol%) and AcOH (11.5 µL, 0.20 mmol, 1.0 equiv.) in toluene (2.0 mL) were heated at 130 °C for 18 h. The crude reaction mixture was purified by flash column chromatography on silica gel (1% EtOAc in Petrol) to give the title product as an off-white, crystalline solid (38.7 mg, 75%). *When run at 120 °C, the reaction gave the title product in a 68% yield (35.2 mg)*

^{1}H NMR (400 MHz, $CDCl_3$) δ 8.98 – 8.94 (m, 1H, Pyr-*H*), 8.10 (td, *J* = 9.0, 6.5 Hz, 1H, Ar-*H*), 7.99 (dd, *J* = 8.0, 2.5 Hz, 1H, Pyr-*H*), 7.90 (d, *J* = 8.0 Hz, 1H, Pyr-*H*), 7.07 – 7.01 (m, 1H, Ar-*H*), 6.95 (ddd, *J* = 11.0, 9.0, 2.5 Hz, 1H, Ar-*H*); **^{13}C NMR** (101 MHz, $CDCl_3$) δ 164.0 (dd, *J* = 253.0, 12.5 Hz), 161.0 (dd, *J* = 252.5, 12.0 Hz), 155.9, 146.7 (q, $^3J_{C-F}$ = 4.0 Hz), 133.9 (q, $^3J_{C-F}$ = 3.5 Hz), 132.6 (dd, *J* = 10.0, 4.0 Hz), 125.3 (q, $^2J_{C-F}$ = 33.0 Hz), 123.7 (q, $^1J_{C-F}$ = 273.0 Hz), 123.8 (d, *J* = 11.0 Hz), 122.6 (dd, *J* = 11.0, 4.0 Hz), 112.4 (dd, *J* = 21.0, 3.5 Hz), 104.8 (dd, *J* = 27.0, 25.5 Hz); **^{19}F NMR** (377 MHz, $CDCl_3$) δ -62.40 (s, C*F$_3$*), -107.22 – -107.25 (m, C*F*), -112.06 – -112.09 (m, C*F*); **HRMS** (ESI$^+$) found *m/z* 260.0493 [M+H]$^+$, $C_{12}H_7NF_5$ requires m/z 260.0493; **IR** ν_{max} (neat)/ cm^{-1} 3095, 3035, 2920, 2852, 2183, 2152, 2027, 1987, 1908, 1702, 1602, 1571, 1511, 1488, 1430, 1389, 1333, 1295, 1266, 1243, 1217, 1177, 1133, 1108, 1084, 1018, 966, 913, 851, 820, 774, 739, 719, 641; **mp** 56-58 °C (EtOAc); **R$_f$** = 0.54 (5% EtOAc in Petrol). Data consistent with literature: T. Rossolini, J. A. Leitch, R. Grainger, D. Dixon, *Org. Lett*. 2018, **20,** 21, 6794-6798.[13]

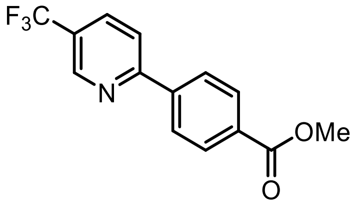

methyl 4-(5-(trifluoromethyl)pyridin-2-yl)benzoate (3i)

From the β-nitrile sulfone: Following general procedure **F**, 3-((5-(trifluoromethyl)pyridin-2-yl)sulfonyl)propanenitrile (58.0 mg, 0.22 mmol, 1.1 equiv.), methyl 4-bromobenzoate (43.0 mg, 0.20 mmol, 1.0 equiv.), $Pd(OAc)_2$ (2.2 mg, 0.010 mmol, 5.0 mol%), CataCXium A (7.2 mg, 0.020 mmol, 10 mol%), K_2CO_3 (41.4 mg, 0.30 mmol, 1.5 equiv.) and AcOH (11.4 µL, 0.20 mmol, 1.0 equiv.) in toluene (2.0 mL) were heated at 120 °C for 18 h. The crude reaction mixture was purified by flash column chromatography (2-3% EtOAc in Petrol) to give the title compound as a white solid (49.0 mg, 87%).

From the β-methylester sulfone: Following general procedure **G**, methyl 3-((5-(trifluoromethyl)pyridin-2-yl)sulfonyl)propanoate (89.0 mg, 0.30 mmol, 1.5 equiv.), methyl 4-bromobenzoate (43.0 mg, 0.20 mmol, 1.0 equiv.), $Pd(OAc)_2$ (2.2 mg, 0.010 mmol, 5.0 mol%), K_2CO_3 (55.3 mg, 0.40 mmol, 2.0 equiv.) and CataCXium A (7.2 mg, 0.020 mmol, 10 mol%) in toluene (2.0 mL) were heated at 120 °C for 18 h. The crude reaction mixture was purified by flash column chromatography on silica gel (2-3% EtOAc in Petrol) to give the title product as a white solid (44.4 mg, 79%).

^{1}H NMR (400 MHz, $CDCl_3$) δ 8.97 – 8.96 (m, 1H, Ar-*H*), 8.16 (d, *J* = 8.5 Hz, 2H, Ar-*H*), 8.10 (d, *J* = 8.5 Hz, 2H, Ar-*H*), 8.01 (dd, *J* = 8.5 Hz, 2.0 Hz, 1H, Ar-*H*), 7.89 (d, *J* = 8.5 Hz, 1H, Ar-*H*), 3.95 (s, 3H, CO_2CH_3); **^{13}C NMR** (101 MHz, $CDCl_3$) δ 166.8, 159.5, 146.9 (q, $^{3}J_{C\text{-}F}$ = 4.0 Hz), 142.0, 134.2 (q, $^{3}J_{C\text{-}F}$ = 3.5 Hz), 131.5, 130.3, 127.4, 125.7 (q, $^{2}J_{C\text{-}F}$ = 33.0 Hz), 123.7 (q, $^{1}J_{C\text{-}F}$ = 272.5 Hz), 120.6, 52.4; **^{19}F NMR** (377 MHz, $CDCl_3$) δ -62.34 (s); **LRMS** (ESI$^+$) *m/z* 282.0 $[M+H]^+$; **HRMS** (ESI$^+$) found *m/z* 282.0735 $[M+H]^+$, $C_{14}H_{11}F_3NO_2$ requires m/z 282.0736; **IR** ν_{max} (neat)/cm^{-1} 2928, 1722, 1606, 1343, 1281, 1129, 1014, 839, 752; **mp** 103-105 °C; **R$_f$** = 0.17 (5% EtOAc in Petrol). Data is consistent with literature: T. Markovic, B. N. Rocke, D. C. Blakemore, V. Mascitti and M. C. Willis, *Org. Lett.,* 2017, **19**, 22, 6033-6035.[13]

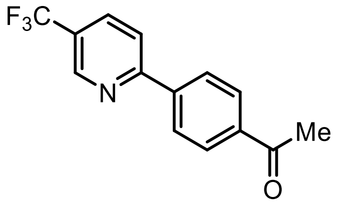

1-(4-(5-(trifluoromethyl)pyridin-2-yl)phenyl)ethan-1-one (3j)

From the β-nitrile sulfone: Following general procedure **F**, 3-((5-(trifluoromethyl)pyridin-2-yl)sulfonyl)propanenitrile (58 mg, 0.22 mmol, 1.1 equiv.), 4-bromoacetophenone (40.0 mg, 0.20 mmol, 1.0 equiv.), $Pd(OAc)_2$ (2.2 mg, 0.010 mmol, 5.0 mol%), CataCXium A (7.2 mg,

0.020 mmol, 10 mol%), K_2CO_3 (41.4 mg, 0.30 mmol, 1.5 equiv.) and AcOH (11.4 µL, 0.20 mmol, 1.0 equiv.) in toluene (2.0 mL) were heated at 120 °C for 18 h. The crude reaction mixture was purified by flash column chromatography (5% EtOAc in Petrol) to give the title compound as a yellow solid (44.0 mg, 83%).

^{1}H NMR (400 MHz, $CDCl_3$) δ 8.99 – 8.98 (m, 1H, Ar-*H*), 8.15 (d, *J* = 8.5 Hz, 2H, Ar-*H*), 8.09 (d, *J* = 8.5 Hz, 2H, Ar-*H*), 8.03 (dd, *J* = 8.5 Hz, 2.0 Hz, 1H, Ar-*H*), 7.91 (d, *J* = 8.5 Hz, 1H, Ar-*H*), 2.67 (s, 3H, C(O)CH_3); **^{13}C NMR** (101 MHz, $CDCl_3$) δ 197.7, 159.4, 146.9 (q, $^3J_{C-F}$ = 4.0 Hz), 142.1, 138.1, 134.3 (q, $^3J_{C-F}$ = 3.5 Hz), 129.0, 127.6, 125.7 (q, $^2J_{C-F}$ = 33.0 Hz), 123.7 (q, $^1J_{C-F}$ = 271.5 Hz), 120.6, 26.9; **^{19}F NMR** (377 MHz, $CDCl_3$) δ -62.34 (s); **LRMS** (ESI$^+$) *m/z* 266.0 $[M+H]^+$; **HRMS** (ESI$^+$) found *m/z* 266.0787 $[M+H]^+$, $C_{14}H_{11}F_3NO$ requires m/z 266.0787; **IR** ν_{max} (neat)/cm^{-1} 2981, 2889, 1683, 1329, 1137, 905, 727; **mp** 110-115 °C (CH_2Cl_2); **R$_f$** = 0.28 (10% EtOAc in Petrol).

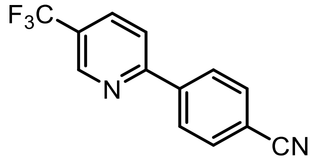

4-(5-(trifluoromethyl)pyridin-2-yl)benzonitrile (3k)

From the β-nitrile sulfone: Following general procedure **F**, 3-((5-(trifluoromethyl)pyridin-2-yl)sulfonyl)propanenitrile (58.1 mg, 0.22 mmol, 1.1 equiv.), 4-bromobenzonitrile (36.4 mg, 0.20 mmol, 1.0 equiv.), $Pd(OAc)_2$ (2.2 mg, 0.010 mmol, 5.0 mol%), K_2CO_3 (41.4 mg, 0.30 mmol, 1.5 equiv.), CataCXium A (7.2 mg, 0.020 mmol, 10 mol%) and AcOH (11.5 µL, 0.20 mmol, 1.0 equiv.) in toluene (2.0 mL) were heated at 120 °C for 18 h. The crude reaction mixture was purified by flash column chromatography on silica gel (5% EtOAc in Petrol) to give the title product as an off-white solid (45.3 mg, 91%).

From the β-methylester sulfone: Following general procedure **G**, methyl 3-((5-(trifluoromethyl)pyridin-2-yl)sulfonyl)propanoate (89.2 mg, 0.30 mmol, 1.5 equiv.), 4-bromobenzonitrile (36.4 mg, 0.20 mmol, 1.0 equiv.), $Pd(OAc)_2$ (2.2 mg, 0.010 mmol, 5.0 mol%), K_2CO_3 (55.3 mg, 0.40 mmol, 2.0 equiv.) and CataCXium A (7.2 mg, 0.020 mmol, 10 mol%) in toluene (2.0 mL) were heated at 120 °C for 18 h. The crude reaction mixture was purified by flash column chromatography on silica gel (5% EtOAc in Petrol) to give the title product as an off-white solid (36.2 mg, 73%).

^{1}H NMR (400 MHz, $CDCl_3$) δ 8.98 (dq, *J* = 2.5, 1.0 Hz, 1H, Pyr-*H*), 8.17 (d, *J* = 9.0 Hz, 2H, Ar-*H*), 8.05 (dd, *J* = 8.5, 2.5 Hz, 1H, Pyr-*H*), 7.89 (d, *J* = 8.5 Hz, 1H, Pyr-*H*), 7.79 (d, *J* = 9.0 Hz, 2H, Ar-*H*); **^{13}C NMR** (101 MHz, $CDCl_3$) δ 158.5, 147.1 (q, $^3J_{C-F}$ = 4.0 Hz), 142.0, 134.5 (q, $^3J_{C-F}$ = 3.5 Hz), 132.9, 128.0, 126.0 (q, $^2J_{C-F}$ = 33.0 Hz), 123.3 (q, $^1J_{C-F}$ = 127.0 Hz), 120.6, 118.6, 113.7; **^{19}F NMR** (377 MHz, $CDCl_3$) δ -62.40 (s); **LRMS** (ESI$^+$) *m/z* 249.3 $[M+H]^+$; **HRMS** (ESI$^+$) found *m/z* 249.0634 $[M+H]^+$, $C_{13}H_8N_2F_3$ requires m/z 249.0640; **IR** ν_{max} (neat)/cm^{-1} 304, 2924, 2360, 2226m 1718, 1601, 1581, 1560, 1485, 1413, 1386, 1325, 1297, 1244, 1171, 1142, 11201, 1083, 1032, 1013, 982, 951, 863, 834, 770, 740, 721; **mp** 95-102 °C (EtOAc); **R$_f$** = 0.23 (5% EtOAc in Petrol). Data is consistent with literature: T. Markovic, B. N. Rocke, D. C. Blakemore, V. Mascitti, M. C. Willis, *Org. Lett.* 2017, **19**, 22, 6033-6035.[13]

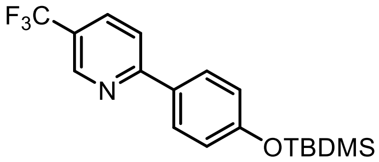

2-(4-((tert-butyldimethylsilyl)oxy)phenyl)-5-(trifluoromethyl)pyridine (3l)

From the β-nitrile sulfone: Following general procedure **F**, 3-((5-(trifluoromethyl)pyridin-2-yl)sulfonyl)propanoate (58.2 mg, 0.22 mmol, 1.1 equiv.), (4-bromophenoxy)(tert-butyl)dimethylsilane (57.5 mg, 0.20 mmol, 1.0 equiv.), $Pd(OAc)_2$ (2.2 mg, 0.010 mmol, 5.0 mol%), K_2CO_3 (41.4 mg, 0.30 mmol, 1.5 equiv.), CataCXium A (7.2 mg, 0.020 mmol, 10 mol%) and AcOH (11.5 µL, 0.20 mmol, 1.0 equiv.) in toluene (2.0 mL) were heated at 120 °C for 18 h. The crude reaction mixture was purified by flash column chromatography on silica gel (0.5% EtOAc in Petrol) to give the title product as a yellow oil (55.0 mg, 78%).

From the β-methyl ester sulfone: Following general procedure **G**, methyl 3-((5-(trifluoromethyl)pyridin-2-yl)sulfonyl)propanoate (89.2 mg, 0.30 mmol, 1.5 equiv.), (4-bromophenoxy)(tert-butyl)dimethylsilane (57.5 mg, 0.20 mmol, 1.0 equiv.), $Pd(OAc)_2$ (2.2 mg, 0.010 mmol, 5.0 mol%), K_2CO_3 (55.3 mg, 0.40 mmol, 2.0 equiv.) and CataCXium A (7.2 mg, 0.020 mmol, 10 mol%) in toluene (2.0 mL) were heated at 120 °C for 18 h. The crude reaction mixture was purified by flash column chromatography on silica gel (0.5% EtOAc in Petrol) to give the title product as a yellow oil (49.0 mg, 69%).

^{1}H NMR (400 MHz, $CDCl_3$) δ 8.92 – 8.87 (m, 1H, Ar-*H*), 7.99 – 7.89 (m, 3H, Ar-*H*), 7.77 (d, *J* = 8.5 Hz, 1H, Ar-*H*), 6.96 (d, *J* = 9.0 Hz, 2H, Ar-*H*), 1.01 (s, 9H, C-C*H3*), 0.24 (s, 6H, Si-C*H3*); **^{13}C NMR** (101 MHz, $CDCl_3$) δ 160.5, 158.0, 146.5 (q, $^3J_{C-F}$ = 4.0 Hz), 134.0 (q, $^3J_{C-F}$ = 3.0 Hz), 131.1, 128.8, 124.1 (q, $^2J_{C-F}$ = 33.5 Hz), 123.9 (q, $^1J_{C-F}$ = 272.0 Hz), 120.8, 119.4, 25.8, 18.4, 4.2; **^{19}F NMR** (377 MHz, $CDCl_3$) δ -62.17 (s); **LRMS** (ESI$^+$) m/z 354.0 [M+H]$^+$; **HRMS** (ESI$^+$) found *m/z* 354.1488 [M+H]$^+$, $C_{18}H_{23}F_3NOSi$ requires m/z 354.1496; **IR** ν_{max} (neat)/cm^{-1} 2957, 2932, 2860, 1598, 1327, 1261, 1130, 910, 833; **R$_f$** = 0.20 (1% EtOAc in Petrol).

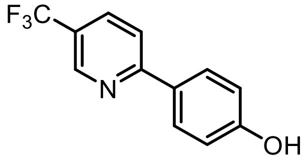

4-(5-(trifluoromethyl)pyridin-2-yl)phenol (3l-OH)

From the β-methyl ester sulfone: Following general procedure **G**, methyl 3-((5-(trifluoromethyl)pyridin-2-yl)sulfonyl)propanoate (89.2 mg, 0.30 mmol, 1.5 equiv.), (4-bromophenoxy)(tert-butyl)dimethylsilane (57.5 mg, 0.20 mmol, 1.0 equiv.), $Pd(OAc)_2$ (2.2 mg, 0.010 mmol, 5.0 mol%), K_2CO_3 (55.3 mg, 0.40 mmol, 2.0 equiv.) and CataCXium A (7.2 mg, 0.020 mmol, 10 mol%) in toluene (2.0 mL) were heated at 120 °C for 18 h. The reaction temperature was then lowered to 85 °C. Ethanol (2.0 mL) and an aqueous solution of K_2CO_3 (200 µL, 2.0 M) were added to the sealed vial. The reaction was left to heat at 85 °C for another 18 h. Once cooled, the reaction mixture was concentrated *in vacuo*. The crude reaction

mixture was purified by flash column chromatography on silica gel (5% to 10% EtOAc in Petrol) to give the title product as a pale yellow solid (31.0 mg, 65% over two steps).

^{1}H NMR (400 MHz, $CDCl_3$) δ 8.91 – 8.85 (m, 1H, Ar-*H*), 7.97 (ddd, *J* = 8.5, 2.5, 0.5 Hz, 1H, Ar-*H*), 7.84 (d, *J* = 9.0 Hz, 2H, Ar-*H*), 7.77 (d, *J* = 8.5 Hz, 1H, Ar-*H*), 6.85 (d, *J* = 9.0 Hz, 2H, Ar-*H*); **^{13}C NMR** (101 MHz, $CDCl_3$) δ 160.7, 158.6, 146.2 (q, $^3J_{C\text{-}F}$ = 4.5 Hz), 134.6 (q, $^3J_{C\text{-}F}$ = 4.0 Hz), 129.8, 129.2, 124.6 (q, $^2J_{C\text{-}F}$ = 32.5 Hz), 123.8 (q, $^1J_{C\text{-}F}$ = 273.5 Hz), 120.2, 116.3; **^{19}F NMR** (377 MHz, $CDCl_3$) δ -62.21 (s); **LRMS** (ESI^+) m/z 240.0 $[M+H]^+$; **HRMS** (ESI^+) found *m/z* 240.0631 $[M+H]^+$, $C_{12}H_9F_3NO$ requires m/z 240.0631; **IR** v_{max} (neat)/cm^{-1} 3073, 1604, 1328, 1129, 1086, 831; **mp** 99-101 °C (CH_2Cl_2); **R_f** = 0.22 (20% EtOAc in Petrol).

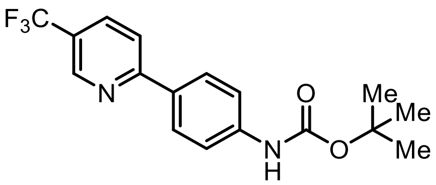

tert-butyl (4-(5-(trifluoromethyl)pyridin-2-yl)phenyl)carbamate (3m)

From the β-nitrile sulfone: Following general procedure **F**, 3-((5-(trifluoromethyl)pyridin-2-yl)sulfonyl)propanenitrile (58.1 mg, 0.22 mmol, 1.1 equiv.), *tert*-butyl (4-bromophenyl) carbamate, (54.4 mg, 0.20 mmol, 1.0 equiv.), $Pd(OAc)_2$ (2.2 mg, 0.010 mmol, 5.0 mol%), K_2CO_3 (41.4 mg, 0.30 mmol, 1.5 equiv.), CataCXium A (7.2 mg, 0.020 mmol, 10 mol%) and AcOH (11.5 µL, 0.20 mmol, 1.0 equiv.) in toluene (2.0 mL) were heated at 120 °C for 18 h. The crude reaction mixture was purified by flash column chromatography on silica gel (2-4% EtOAc in Petrol) to give the title product as an off-white solid (50.4 mg, 75%).

^{1}H NMR (400 MHz, $CDCl_3$) δ 8.92 – 8.87 (m, 1H, Pyr-*H*), 8.00 (d, *J* = 9.0 Hz, 2H, Ar-*H*), 7.93 (app. ddd, *J* = 8.5, 2.5, 1.0 Hz, 1H, Pyr-*H*), 7.80 (d, *J* = 8.5 Hz, 1H, Pyr-*H*), 7.51 (d, *J* = 9.0 Hz, 2H, Ar-*H*), 6.62 (br.s, 1H, N*H*), 1.54 (s, 9H, $C(CH_3)_3$); **^{13}C NMR** (101 MHz, $CDCl_3$) δ 160.1, 152.6, 146.6 (q, $^3J_{C\text{-}F}$ = 4.0 Hz), 140.5, 134.0 (q, $^3J_{C\text{-}F}$ = 4.0 Hz), 132.5, 128.2, 124.4 (q, $^2J_{C\text{-}F}$ = 33.0 Hz), 124.0 (q, $^1J_{C\text{-}F}$ = 272.3 Hz), 119.4, 118.6, 81.1, 28.5; **^{19}F NMR** (377 MHz, $CDCl_3$) δ -62.20 (s); **LRMS** (ESI^+) m/z 339.2 $[M+H]^+$**;** **HRMS** (ESI^+) found *m/z* 339.1316 $[M+H]^+$, $C_{17}H_{18}N_2O_2F_3$ requires m/z 339.1315; **IR** v_{max} (neat)/cm^{-1} 3362, 2987, 2924, 2284, 1699, 1602, 1590, 1568, 1522, 1506, 1416, 1374, 1337, 1268, 1156, 1138, 1084, 832, 772, 716, 618; **mp** 159-162 °C (EtOAc); **R_f** = 0.35 (4% EtOAc in Petrol). Data is consistent with literature: T. Markovic, B. N. Rocke, D. C. Blakemore, V. Mascitt, M. C. Willis, *Org. Lett.* 2017, **19**, 22, 6033-6035.[13]

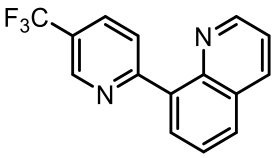

8-(5-(trifluoromethyl)pyridin-2-yl)quinolone (3n)

From the β-nitrile sulfone: Following general procedure **F**, 3-((5-(trifluoromethyl)pyridin-2-yl)sulfonyl)propanenitrile (58.1 mg, 0.22 mmol, 1.1 equiv.), 8-bromoquinoline (41.6 mg, 0.20 mmol, 1.0 equiv.), $Pd(OAc)_2$ (2.2 mg, 0.010 mmol, 5.0 mol%), K_2CO_3 (41.4 mg, 0.30 mmol, 1.5 equiv.), CataCXium A (7.2 mg, 0.020 mmol, 10 mol%) and AcOH (11.5 µL, 0.20 mmol, 1.0 equiv.) in toluene (2.0 mL) were heated at 120 °C for 18 h. The crude reaction mixture was purified by flash column chromatography on silica gel (8% EtOAc in Petrol). The product was then re-purified by flash column chromatography on silica gel (5% EtOAc in Petrol) to give the title product as an off-white, crystalline solid (40.5 mg, 74%).

^{1}H NMR (400 MHz, $CDCl_3$) δ 9.05 (dq, *J* = 2.5, 1.0 Hz, 1H, Pyr-*H*), 8.99 (dd, *J* = 4.0, 2.0 Hz, 1H, Quin-*H*), 8.31 (d, *J* = 8.5 Hz, 1H, Pyr-*H*), 8.27 (dd, *J* = 8.5, 2.0 Hz, 1H, Quin-*H*), 8.21 (dd, *J* = 7.0, 1.5 Hz, 1H, Quin-*H*), 8.04 (dd, *J* = 8.5, 2.5 Hz, 1H, Pyr-*H*), 7.95 (dd, *J* = 8.0, 1.5 Hz, 1H, Quin-*H*), 7.71 (dd, *J* = 8.0, 7.0 Hz, 1H, Quin-*H*), 7.48 (dd, *J* = 8.5, 4.0 Hz, 1H, Quin-*H*); **^{13}C NMR** (101 MHz, $CDCl_3$) δ 160.5, 150.7, 146.5 (q, $^3J_{C-F}$ = 4.0 Hz), 145.6, 137.4, 137.0, 132.8 (q, $^3J_{C-F}$ = 3.5 Hz), 131.8, 129.9, 128.9, 126.8, 126.7, 125.0 (q, J^2_{C-F} = 33.5 Hz), 124.0 (q, $^1J_{C-F}$ = 272.0 Hz), 121.5; **^{19}F NMR** (377 MHz, $CDCl_3$) δ -62.24 (s); **LRMS** (ESI^+) *m/z* 275.0 $[M+H]^+$; **HRMS** (ESI^+) found *m/z* 275.0791 $[M+H]^+$, $C_{15}H_{10}ON_2F_3$ requires m/z 275.0791; **IR** v_{max} (neat)/ cm^{-1} 3057, 2924, 2854, 2108, 1926, 1823, 1739; 1600, 1560, 1327, 1190, 1156, 1130, 1103, 1079, 1026, 844, 825, 812, 794, 772; **mp** 67-70 °C (EtOAc); **R$_f$** = 0.18 (5% EtOAc in Petrol). Data consistent with literature: T. Markovic, B. N. Rocke, D. C. Blakemore, V. Mascitti and M. C. Willis, *Chem. Sci.*, 2017, **8**, 4437-4442.[9]

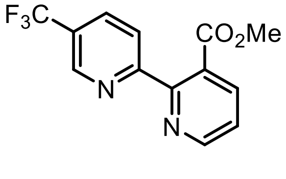

methyl 5'-(trifluoromethyl)-[2,2'-bipyridine]-3-carboxylate (3o)

From the β-nitrile sulfone: Following general procedure **F**, 3-((5-(trifluoromethyl)pyridin-2-yl)sulfonyl)propanenitrile (58.1 mg, 0.22 mmol, 1.1 equiv.), methyl 2-bromonicotinate (43.0 mg, 0.20 mmol, 1.0 equiv.), $Pd(OAc)_2$ (2.2 mg, 0.010 mmol, 5.0 mol%), CataCXium A (7.2 mg, 0.020 mmol, 10 mol%), K_2CO_3 (41.4 mg, 0.30 mmol, 1.5 equiv.) and AcOH (11.4 µL, 0.20 mmol, 1.0 equiv.) in toluene (2.0 mL) were heated at 120 °C for 18 h. The crude reaction mixture was purified by flash column chromatography (5-20% EtOAc in Petrol) to give the title compound as a yellow oil (43.0 mg, 76%).

^{1}H NMR (400 MHz, $CDCl_3$) δ 8.87 – 8.86 (m, 1H, Ar-*H*), 8.77 (dd, *J* = 5.0 Hz, 1.5 Hz, 1H, Ar-*H*), 8.29 (d, *J* = 8.0 Hz, 1H, Ar-*H*), 8.07 (dd, *J* = 8.5 Hz, 2.5 Hz, 1H, Ar-*H*), 8.00 (dd, *J* = 8.0 Hz, 1.5 Hz, 1H, Ar-*H*), 7.44 (dd, *J* = 8.0 Hz, 5.0 Hz, 1H, Ar-*H*), 3.81 (s, 3H, CO_2CH_3); **^{13}C NMR** (101 MHz, $CDCl_3$) δ 169.0, 159.3, 154.1, 150.7, 145.4 (q, $^{3}J_{C\text{-}F}$ = 4.0 Hz), 137.3, 134.2 (q, $^{3}J_{C\text{-}F}$ = 3.5 Hz), 129.0, 126.4 (q, $^{2}J_{C\text{-}F}$ = 33.0 Hz), 123.7, 123.6 (q, $^{1}J_{C\text{-}F}$ = 272.5 Hz), 122.6, 52.7; **^{19}F NMR** (377 MHz, $CDCl_3$) δ -62.38 (s); **LRMS** (ESI^+) *m/z* 283.0 ($[M+H]^+$); **HRMS** (ESI^+) found *m/z* 283.0688 $[M+H]^+$, $C_{13}H_{10}F_3N_2O_2$ requires m/z 283.0689; **IR** ν_{max} (neat)/cm^{-1} 3052, 2847, 1731, 1605, 1325, 1124, 1017, 759; **R_f** = 0.12 (20% EtOAc in Petrol).

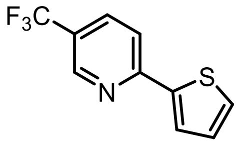

2-(thiophen-2-yl)-5-(trifluoromethyl)pyridine (3p)

From the β-nitrile sulfone: Following general procedure **F**, 3-((5-(trifluoromethyl)pyridin-2-yl)sulfonyl)propanenitrile (58.1 mg, 0.22 mmol, 1.1 equiv.), $Pd(OAc)_2$ (2.2 mg, 0.010 mmol, 5.0 mol%), CataCXium A (7.2 mg, 0.020 mmol, 10 mol%), K_2CO_3 (41.4 mg, 0.30 mmol, 1.5 equiv.), 2-bromothiophene (32.0 mg, 0.20 mmol, 1.0 equiv.) and AcOH (11.4 µL, 0.20 mmol, 1.0 equiv.) in toluene (2.0 mL) were heated at 130 °C for 18 h. The crude reaction mixture was purified by flash column chromatography (0-0.5% EtOAc in Petrol) to give the title compound as a white solid (40.0 mg, 87%). *When run at 120 °C, the reaction gave the title product in a 52% yield (24.0 mg)*

^{1}H NMR (400 MHz, $CDCl_3$) δ 8.81 (s, 1H, Ar-*H*), 7.90 (dd, *J* = 8.5 Hz, 2.5 Hz, 1H, Ar-*H*), 7.74 (d, *J* = 8.5 Hz, 1H, Ar-*H*), 7.68 (dd, *J* = 4.0 Hz, 1.0 Hz, 1H, Ar-*H*), 7.49 (dd, *J* = 5.0 Hz, 1.0 Hz, 1H, Ar-*H*), 7.15 (dd, *J* = 5.0 Hz, 3.5 Hz, 1H, Ar-*H*); **^{13}C NMR** (101 MHz, $CDCl_3$) δ 155.8, 146.8 (q, $^{3}J_{C\text{-}F}$ = 4.0 Hz), 143.4, 134.0 (q, $^{3}J_{C\text{-}F}$ = 3.5 Hz), 129.6, 128.5, 126.5, 124.5 (q, $^{2}J_{C\text{-}F}$ = 32.5 Hz), 123.8 (q, $^{1}J_{C\text{-}F}$ = 272.0 Hz), 118.3; **^{19}F NMR** (377 MHz, $CDCl_3$) δ -62.32 (s); **LRMS** (ESI^+) *m/z* 230.0 $[M+H]^+$; **HRMS** (ESI^+) found *m/z* 230.02480 $[M+H]^+$, $C_{10}H_7F_3NS$ requires m/z 230.02458; **IR** ν_{max} (neat)/cm^{-1} 2981, 2889, 1603, 1393, 1337, 1124, 1108, 949, 847, 715; **mp** 80-82 °C (CH_2Cl_2); **R_f** = 0.15 (1% EtOAc in Petrol).

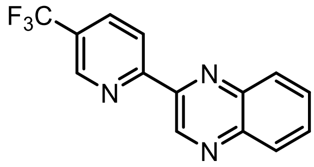

2-(5-(trifluoromethyl)pyridin-2-yl)quinoxaline (3q)

From the β-nitrile sulfone: Following general procedure **F**, 3-((5-(trifluoromethyl)pyridin-2-yl)sulfonyl)propanenitrile (58.1 mg, 0.22 mmol, 1.1 equiv.), 2-bromoquinoxaline (42.0 mg, 0.20 mmol, 1.0 equiv.), $Pd(OAc)_2$ (2.2 mg, 0.010 mmol, 5.0 mol%), CataCXium A (7.2 mg, 0.020 mmol, 10 mol%), K_2CO_3 (41.4 mg, 0.30 mmol, 1.5 equiv.) and AcOH (11.4 µL, 0.20 mmol, 1.0 equiv.) in toluene (2.0 mL) were heated at 120 °C for 18 h. The crude reaction mixture was purified by flash column chromatography (5-10% EtOAc in Petrol) to give the title compound as a white solid (41.1 mg, 75%).

From the β-methylester sulfone: Following general procedure **G**, methyl 3-((5-(trifluoromethyl)pyridin-2-yl)sulfonyl)propanoate (89.0 mg, 0.30 mmol, 1.5 equiv.), 2-bromoquinoxaline (42.0 mg, 0.20 mmol, 1.0 equiv.), $Pd(OAc)_2$ (2.2 mg, 0.010 mmol, 5.0 mol%), CataCXium A (7.2 mg, 0.020 mmol, 10 mol%) and K_2CO_3 (55.2 mg, 0.40 mmol, 2.0 equiv.) in toluene (2 mL, 0.1 M) were heated at 130 °C for 18 h. The crude reaction mixture was purified by flash column chromatography (5-10% EtOAc in Petrol) to give the title compound as a white solid (36.0 mg, 65%). *When run at 120 °C, the reaction gave the title product in a 55% yield (30.0 mg).*

^{1}H NMR (400 MHz, $CDCl_3$) δ 9.98 (s, 1H, Ar-*H*), 9.02 (dd, *J* = 1.5 Hz, 1.0 Hz, 1H, Ar-*H*), 8.73 (d, *J* = 8.5 Hz, 1H, Ar-*H*), 8.20 – 8.14 (m, 2H, Ar-*H*), 8.13 – 8.09 (m, 1H, Ar-*H*), 7.85 – 7.78 (m, 2H, Ar-*H*); **^{13}C NMR** (101 MHz, $CDCl_3$) δ 157.8, 148.8, 146.5 (q, $^3J_{C\text{-}F}$ = 4.0 Hz), 144.2, 143.1, 141.9, 134.4 (q, $^3J_{C\text{-}F}$ = 3.5 Hz), 130.9, 130.6, 130.0, 129.5, 127.2 (q, $^2J_{C\text{-}F}$ = 33.0 Hz), 123.7 (q, $^1J_{C\text{-}F}$ = 272.5 Hz), 121.7; **^{19}F NMR** (377 MHz, $CDCl_3$) δ -62.43 (s); **HRMS** (ESI^+) found *m/z* 276.0743 $[M+H]^+$, $C_{14}H_9N_3F_3$ requires m/z 276.0743; **IR** ν_{max} (neat)/cm^{-1} 2980, 2888, 1332, 1312, 1115, 1078, 1012, 856, 757; **mp** 138-142 °C (CH_2Cl_2); **R_f** = 0.38 (10% EtOAc in Petrol).

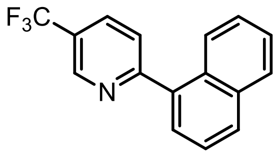

2-(naphthalen-1-yl)-5-(trifluoromethyl)pyridine (3r)

From the β-nitrile sulfone: Following general procedure **F**, 3-((5-(trifluoromethyl)pyridin-2-yl)sulfonyl)propanenitrile (58.1 mg, 0.22 mmol, 1.1 equiv.), 1-bromonaphthalene (28.0 µL, 0.20 mmol, 1.0 equiv.), $Pd(OAc)_2$ (2.2 mg, 0.010 mmol, 5.0 mol%), CataCXium A (7.2 mg, 0.020 mmol, 10 mol%), K_2CO_3 (41.4 mg, 0.30 mmol, 1.5 equiv.) and AcOH (11.4 µL, 0.20 mmol, 1.0 equiv.) in toluene (2.0 mL) were heated at 120 °C for 18 h. The crude reaction mixture was purified by flash column chromatography (0-0.4% EtOAc in Petrol) to give the title compound as a white solid (53.0 mg, 97%).

^{1}H NMR (400 MHz, $CDCl_3$) δ 9.08 (s, 1H, Ar-*H*), 8.08 – 8.06 (m, 2H, Ar-*H*), 7.98 – 7.93 (m, 2H, Ar-*H*), 7.73 (d, *J* = 8.0 Hz, 1H, Ar-*H*), 7.64 – 7.49 (m, 4H, Ar-*H*); **^{13}C NMR** (101 MHz, $CDCl_3$) δ 162.9, 146.6 (q, $^3J_{C-F}$ = 4.0 Hz), 137.2, 134.1, 133.7 (q, $^3J_{C-F}$ = 3.0 Hz), 130.9, 129.9, 128.7, 128.0, 127.1, 126.3, 125.4, 125.2, 125.1 (q, $^2J_{C-F}$ = 33.0 Hz), 124.8, 123.9 (q, $^2J_{C-F}$ = 272.0 Hz); **^{19}F NMR** (377 MHz, $CDCl_3$) δ -62.17 (s); **LRMS** (ESI^+) *m/z* 274.0 ($[M+H]^+$); **HRMS** (ESI^+) found *m/z* 274.0838 $[M+H]^+$, $C_{16}H_{11}F_3N$ requires m/z 274.0838; **IR** ν_{max} (neat)/cm^{-1} 3053, 2925, 1605, 1330, 1126, 782; **mp** 58-62 °C (CH_2Cl_2); **R_f** = 0.26 (5% EtOAc in Petrol). Data is consistent with literature: A. Gavryushin, C. Kofink, G. Manolikakes and P. Knochel, *Tetrahedron*, 2006, **62**, 32, 7521-7533.[14]

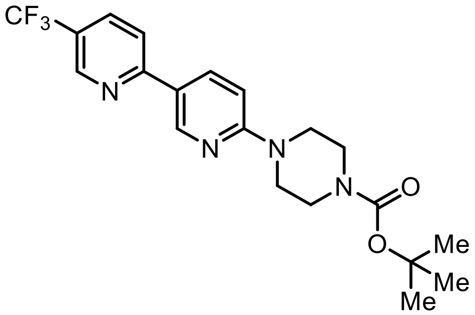

tert-butyl 4-(5-(trifluoromethyl)-[2,3'-bipyridin]-6'-yl)piperazine-1-carboxylate (3s)

From the β-nitrile sulfone: Following general procedure **F**, 3-((5-(trifluoromethyl)pyridin-2-yl)sulfonyl)propanenitrile (58.1 mg, 0.22 mmol, 1.1 equiv.), *tert*-butyl 4-(5-bromopyridin-2-yl)piperazine-1-carboxylate (68.5 mg, 0.20 mmol, 1.0 equiv.), $Pd(OAc)_2$ (2.2 mg, 0.010 mmol, 5.0 mol%), K_2CO_3 (41.4 mg, 0.30 mmol, 1.5 equiv.), CataCXium A (7.2 mg, 0.020 mmol, 10 mol%) and AcOH (11.5 µL, 0.20 mmol, 1.0 equiv.) in toluene (2.0 mL) were heated at 120 °C for 18 h. The crude reaction mixture was purified by flash column chromatography on silica gel (15% EtOAc in Petrol) to give the title product as an off-white solid (65.3 mg, 80%).

^{1}H NMR (400 MHz, $CDCl_3$) δ 8.87 – 8.86 (m, 1H, Pyr-*H*), 8.84 (d, *J* = 2.5 Hz, 1H, Pyr-*H*), 8.23 (dd, *J* = 9.0, 2.5 Hz, 1H, Pyr-*H*), 7.91 (dd, *J* = 8.5, 2.5 Hz, 1H, Pyr-*H*), 7.74 (d, *J* = 8.5 Hz, 1H, Pyr-*H*), 6.73 (d, *J* = 9.0 Hz, 1H, Pyr-*H*), 3.69 – 3.64 (m, 4H, N(C*H*$_2$)$_2$), 3.57 (m, 4H, N(C*H*$_2$)$_2$), 1.49 (s, 9H, C(C*H*$_3$)$_3$); **^{13}C NMR** (101 MHz, $CDCl_3$) δ 159.7, 158.8, 154.9, 147.5, 146.8 (d, $^3J_{C-F}$ = 4.0 Hz), 136.5, 133.9 (d, $^3J_{C-F}$ = 4.0 Hz), 124.1 (q, $^2J_{C-F}$ = 33.0 Hz), 123.9 (q, $^1J_{C-F}$ = 273.0 Hz), 123.2, 118.3, 106.7, 80.2, 44.9, 28.6; **^{19}F NMR** (377 MHz, $CDCl_3$) δ -62.19 (s); **LRMS** (ESI$^+$) m/z 309.2 (25%, [M+H - C_4H_8]$^+$), 353.0 (75%,[M+H- $C_5O_2H_9$]$^+$)**;** **HRMS** (ESI$^+$) found *m/z* 409.1836 [M+H]$^+$, $C_{20}H_{24}O_2N_4F_3$ requires m/z 409.1846; **IR** v_{max} (neat)/cm^{-1} 2986, 2926, 2854, 2359, 1687, 1598, 1421, 1336, 1166, 1128, 1088; **mp** 152-156 °C (EtOAc); **R$_f$** = 0.25 (20% EtOAc in Petrol).

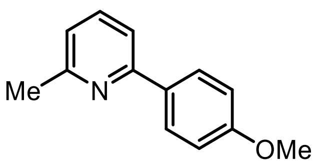

2-(4-methoxyphenyl)-6-methylpyridine (3t)

From the β-nitrile sulfone: Following general procedure **F**, 3-((6-methylpyridin-2-yl)sulfonyl)propanenitrile (46.3 mg, 0.22 mmol, 1.1 equiv.), 4-bromoanisole (25 µL, 0.20 mmol, 1.0 equiv.), $Pd(OAc)_2$ (2.2 mg, 0.010 mmol, 5.0 mol%), K_2CO_3 (41.4 mg, 0.30 mmol, 1.5 equiv.), CataCXium A (7.2 mg, 0.020 mmol, 10 mol%) and AcOH (11.5 µL, 0.20 mmol, 1.0 equiv.) in toluene (2.0 mL) were heated at 130 °C for 18 h. The crude reaction mixture was purified by flash column chromatography on silica gel (5% EtOAc in Petrol) to give the title product as a colourless, viscous oil that crystallised on standing (37.5 mg, 94%).

^{1}H NMR (400 MHz, $CDCl_3$) δ 7.97 – 7.92 (m, 2H, Ar-*H*), 7.59 (t, *J* = 8.0 Hz, 1H, Pyr-*H*), 7.45 (d, *J* = 8.0 Hz, 1H, Pyr-*H*), 7.04 (d, *J* = 8.0 Hz, 1H, Pyr-*H*), 7.01 – 6.96 (m, 2H, Ar-*H*), 3.86 (s, 3H, OCH_3), 2.61 (s, 3H, Pyr-CH_3); **^{13}C NMR** (101 MHz, $CDCl_3$) δ 160.4, 158.3, 156.7, 136.9, 132.6, 128.4, 121.0, 117.0, 114.2, 55.5, 24.9; **LRMS** (ESI$^+$) *m/z* 200.1 $[M+H]^+$; **HRMS** (ESI$^+$) found *m/z* 200.1069 $[M+H]^+$, $C_{13}H_{14}ON$ requires m/z 200.1070; **IR** ν_{max} (neat)/cm^{-1} 2924, 1580, 1514, 1453, 1301, 1249, 1179, 1033, 839, 791, 645; **mp** 52-54 °C (EtOAc); **R_f** = 0.27 (10% EtOAc in Petrol). Data is consistent with literature: M. Zheng, P. Chen, W. Wu, H. Jiang, *Chem. Comm.,* 2016, **52**, 1, 84-87.[15]

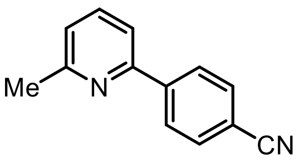

4-(6-methylpyridin-2-yl)benzonitrile (3u)

From the β-nitrile sulfone: Following general procedure **F**, 3-((6-methylpyridin-2-yl)sulfonyl)propanenitrile (46.3 mg, 0.22 mmol, 1.1 equiv.), 4-bromobenzonitrile (36.4 mg, 0.20 mmol, 1.0 equiv.), $Pd(OAc)_2$ (2.2 mg, 0.010 mmol, 5.0 mol%), CataCXium A (7.2 mg, 0.020 mmol, 10 mol%), K_2CO_3 (41.4 mg, 0.30 mmol, 1.5 equiv.) and AcOH (11.4 µL, 0.20 mmol, 1.0 equiv.) in toluene (2.0 mL) were heated at 120 °C for 18 h. The crude reaction mixture was purified by flash column chromatography (5-10% EtOAc in Petrol) to give the title compound as a white solid (34.0 mg, 88%).

From the β-methylester sulfone: Following general procedure **G**, methyl 3-((6-methylpyridin-2-yl)sulfonyl)propanoate (73.0 mg, 0.30 mmol, 1.5 equiv.), 4-bromobenzonitrile (36.4 mg, 0.20 mmol, 1.0 equiv.), $Pd(OAc)_2$ (2.2 mg, 0.010 mmol, 5.0 mol%), CataCXium A (7.2 mg, 0.020 mmol, 10 mol%) and K_2CO_3 (55.2 mg, 0.40 mmol, 2.0 equiv.) in toluene (2.0 mL) were heated at 120 °C for 18 h. The crude reaction mixture was purified by flash column chromatography (5%-10% EtOAc in Petrol) to give the title compound as a white solid (32.0 mg, 82%).

From the diethyl succinate sulfone: Following general procedure **G**, diethyl 2-((6-methylpyridin-2-yl)sulfonyl)succinate (98.8 mg, 0.30 mmol, 1.5 equiv.), 4-bromobenzonitrile (36.4 mg, 0.20 mmol, 1.0 equiv.), $Pd(OAc)_2$ (2.2 mg, 0.010 mmol, 5.0 mol%), CataCXium A (7.2 mg, 0.020 mmol, 10 mol%) and K_2CO_3 (55.2 mg, 0.40 mmol, 2.0 equiv.) in toluene (2.0 mL) were heated at 130 °C for 18 h. The crude reaction mixture was purified by flash column chromatography (5%-10% EtOAc in Petrol) to give the title compound as a white solid (39.0 mg, 99%).

^{1}H NMR (400 MHz, $CDCl_3$) δ 8.11 (d, *J* = 8.5 Hz, 2H, Ar-*H*), 7.75 (d, *J* = 8.5 Hz, 2H, Ar-*H*), 7.69 (t, *J* = 8.0 Hz, 1H, Ar-*H*), 7.56 (d, *J* = 8.0 Hz, 1H, Ar-*H*), 7.18 (d, *J* = 7.5 Hz, 1H, Ar-*H*), 2.64 (s, 3H, Ar-C*H*$_3$); **^{13}C NMR** (101 MHz, $CDCl_3$) δ 159.1, 154.8, 144.0, 137.3, 132.7, 127.7, 123.0, 119.1, 118.2, 112.3, 24.9; **LRMS** (ESI$^+$) *m/z* 195.0 ([M+H]$^+$); **HRMS** (ESI$^+$) found *m/z* 195.0918 [M+H]$^+$, $C_{13}H_{11}N_2$ requires m/z 195.0917; **IR** v_{max} (neat)/cm^{-1} 3057, 2927, 2854, 2229, 1591, 1455, 1160, 851, 792; **mp** 76-80 °C (CH_2Cl_2); **R$_f$** = 0.20 (10% EtOAc in Petrol). Data is consistent with literature: K. Cooper, M. J. Fray, M. J. Parry, K. Richardson and J. Steele, *J. Med. Chem.* 1992, **35**, 17, 3115-3129.[16]

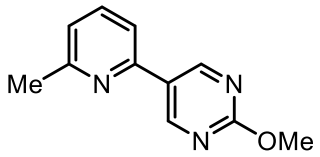

2-methoxy-5-(6-methylpyridin-2-yl)pyrimidine (3v)

From the β-nitrile sulfone: Following general procedure **F**, 3-((6-methylpyridin-2-yl)sulfonyl)propanenitrile (46.3 mg, 0.22 mmol, 1.1 equiv.), 5-bromo-2-methoxypyrimidine (37.8 mg, 0.20 mmol, 1.0 equiv.), $Pd(OAc)_2$ (2.2 mg, 0.010 mmol, 5.0 mol%), K_2CO_3 (41.4 mg, 0.30 mmol, 1.5 equiv.), CataCXium A (7.2 mg, 0.020 mmol, 10 mol%) and AcOH (11.5 µL, 0.20 mmol, 1.0 equiv.) in toluene (2.0 mL) were heated at 120 °C for 18 h. The crude reaction mixture was purified by flash column chromatography on silica gel (30% EtOAc in Petrol) to give the title product as a white, crystalline solid (34.0 mg, 84%).

^{1}H NMR (400 MHz, $CDCl_3$) δ 9.10 (s, 2H, Pyrim-*H*), 7.66 (t, *J* = 8.0 Hz, 1H, Pyr-*H*), 7.45 (d, *J* = 8.0 Hz, 1H, Pyr-*H*), 7.14 (d, *J* = 8.0 Hz, 1H, Pyr-*H*), 4.08 (s, 3H, OC*H*$_3$), 2.61 (s, 3H, C*H*$_3$); **^{13}C NMR** (101 MHz, $CDCl_3$) δ 165.9, 159.3, 158.0, 151.9, 137.3, 127.1, 122.5, 116.8, 55.3, 24.8; **LRMS** (ESI$^+$) *m/z* 202.0 [M+H]$^+$; **HRMS** (ESI$^+$) found *m/z* 202.0975 [M+H]$^+$, $C_{11}H_{12}ON_3$ requires m/z 202.0975; **IR** v_{max} (neat)/cm^{-1} 3017, 2956, 2924, 2854, 2472, 2349, 2163, 1992, 1728, 1653, 1575, 1479. 1455, 1359, 1336, 1315, 1280, 1241, 1440, 1192, 1170, 1108, 1037, 1025, 996, 960, 899, 875, 830, 804, 744; **mp** 79-81 °C (EtOAc); **R$_f$** = 0.14 (30% EtOAc in Petrol).

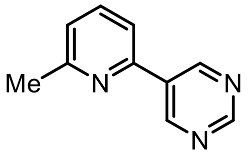

5-(6-methylpyridin-2-yl)pyrimidine (3w)

From the β-nitrile sulfone: Following general procedure **F,** 3-((6-methylpyridin-2-yl)sulfonyl)propanenitrile (46.3 mg, 0.22 mmol, 1.1 equiv.), $Pd(OAc)_2$ (2.2 mg, 0.010 mmol, 5.0 mol%), CataCXium A (7.2 mg, 0.020 mmol, 10 mol%), K_2CO_3 (41.4 mg, 0.30 mmol, 1.5 equiv.), 5-bromopyrimidine (32.0 mg, 0.20 mmol, 1.0 equiv.) and AcOH (11.4 µL, 0.20 mmol, 1.0 equiv.) in toluene (2.0 mL) were heated at 130 °C for 18 h. The crude reaction mixture was purified by flash column chromatography (10-40% EtOAc in Petrol) to give the title compound as a white solid (23.0 mg, 67%). *When run at 120 °C, the reaction gave the title product in a 58% yield (20.0 mg).*

From the β-methylester sulfone: Following general procedure **G**, methyl 3-((6-methylpyridin-2-yl)sulfonyl)propanoate (73.0 mg, 0.30 mmol, 1.5 equiv.), $Pd(OAc)_2$ (2.2 mg, 0.010 mmol, 5.0 mol%), CataCXium A (7.2 mg, 0.020 mmol, 10 mol%), K_2CO_3 (55.2 mg, 0.40 mmol, 2.0 equiv.), and 5-bromopyrimidine (32.0 mg, 0.20 mmol, 1.0 equiv.) in toluene (2.0 mL) were heated at 130 °C for 18 h. The crude reaction mixture was purified by flash column chromatography (5-10% EtOAc in Petrol) to give the title compound as a white solid (18.0 mg, 53%). *When run at 120 °C, the reaction gave the title product in a 41% yield (14.0 mg).*

^{1}H NMR (400 MHz, $CDCl_3$) δ 9.31 (s, 2H, Ar-*H*), 9.23 (s, 1H, Ar-*H*), 7.70 (t, *J* = 8.0 Hz, 1H, Ar-*H*), 7.54 (d, *J* = 8.0 Hz, 1H, Ar-*H*), 7.20 (d, *J* = 7.5 Hz, 1H, Ar-*H*), 2.63 (s, 3H, Ar-C*H*$_3$); **^{13}C NMR** (101 MHz, $CDCl_3$) δ 159.6, 158.6, 155.3, 151.5, 137.5, 132.8, 123.3, 117.7, 24.8; **LRMS** (ESI$^+$) *m/z* 172.0 ([M+H]$^+$); **HRMS** (ESI$^+$) found *m/z* 172.0870 [M+H]$^+$, $C_{10}H_{10}N_3$ requires m/z 172.0869; **IR** v_{max} (neat)/cm^{-1} 3659, 3415, 2981, 2918, 1596, 1577, 1409, 1189, 794; **mp** 100-102 °C (CH_2Cl_2); **R_f** = 0.11 (20% EtOAc in Petrol).

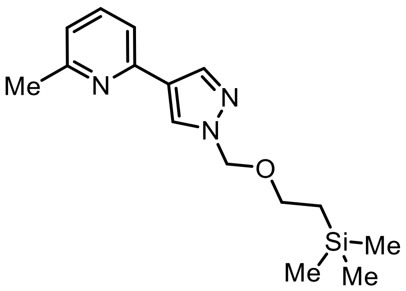

2-methyl-6-(1-((2-(trimethylsilyl)ethoxy)methyl)-1H-pyrazol-4-yl)pyridine (3x)

From the β-nitrile sulfone: Following general procedure **F**, 3-((6-methylpyridin-2-yl)sulfonyl)propanenitrile (46.3 mg, 0.22 mmol, 1.1 equiv.), 4-bromo-1-((2-(trimethylsilyl)ethoxy)methyl)-1H-pyrazole (55.5 mg, 0.20 mmol, 1.0 equiv.), $Pd(OAc)_2$ (2.2 mg, 0.010 mmol, 5.0 mol%), K_2CO_3 (41.4 mg, 0.30 mmol, 1.5 equiv.), CataCXium A (7.2 mg, 0.020 mmol, 10 mol%) and AcOH (11.5 µL, 0.20 mmol, 1.0 equiv.) in toluene (2.0 mL) were heated at 130 °C for 18 h. The crude reaction mixture was purified by flash column chromatography on silica gel (25% EtOAc in Petrol) to give the title product as a colourless oil (42.0 mg, 73%). *When run at 120 °C, the reaction gave the title product in 10% yield (5.8 mg).*

^{1}H NMR (400 MHz, $CDCl_3$) δ 8.10 (d, *J* = 1.0 Hz, 1H, Pyraz-*H*), 8.00 (d, *J* = 1.0 Hz, 1H, Pyraz-*H*), 7.54 (t, *J* = 8.0 Hz, 1H, Pyr-*H*), 7.28 (d, *J* = 7.5 Hz, 1H, Pyr-*H*), 6.97 (d, *J* = 7.5 1H, Pyr-*H*), 5.45 (s, 2H, NC*H*$_2$O), 3.62 – 3.55 (m, 2H, OC*H*$_2$), 2.55 (s, 3H, C*H*$_3$-Pyr), 0.95 – 0.89 (m, 2H, C*H*$_2$TMS), -0.03 (s, 9H, TMS); **^{13}C NMR** (101 MHz, $CDCl_3$) δ 158.6, 151.2, 138.3, 136.9, 128.2, 125.1, 121.0, 116.8, 80.6, 67.0, 24.8, 18.0, -1.3; **LRMS** (ESI$^+$) *m/z* 290.2 $[M+H]^+$; **HRMS** (ESI$^+$) found *m/z* 290.1687 $[M+H]^+$, $C_{15}H_{24}ON_3{}^{28}Si$ requires m/z 290.1683; **IR** ν_{max} (neat)/cm^{-1} 2953, 2360, 1599, 1578, 1562, 1458, 1430, 1249, 1214, 1178, 1096, 978, 916, 859, 837, 791, 754, 694, 668; **R$_f$** = 0.25 (20% EtOAc in Petrol).

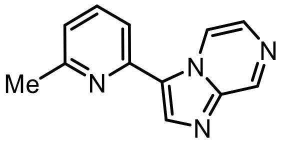

3-(6-methylpyridin-2-yl)imidazo[1,2-a]pyrazine (3y)

From the β-nitrile sulfone: Following general procedure **F**, 3-((6-methylpyridin-2-yl)sulfonyl)propanenitrile (46.3 mg, 0.22 mmol, 1.1 equiv.), 2-bromoimidazo[1,2-a]pyrazine (39.6 mg, 0.20 mmol, 1.0 equiv.), $Pd(OAc)_2$ (2.2 mg, 0.010 mmol, 5.0 mol%), K_2CO_3 (41.4 mg, 0.30 mmol, 1.5 equiv.), CataCXium A (7.2 mg, 0.020 mmol, 10 mol%) and AcOH (11.5 µL, 0.20 mmol, 1.0 equiv.) in toluene (2.0 mL) were heated at 130 °C for 18 h. The crude reaction mixture was purified by flash column chromatography on silica gel (40-60% EtOAc in CH_2Cl_2) to give the title product as a white solid (39.8 mg, 94%).

^{1}H NMR (400 MHz, $CDCl_3$) δ 9.85 (dd, *J* = 4.5, 1.5 Hz, 1H, Ar-*H*), 9.18 (d, *J* = 1.5 Hz, 1H), 8.26 (s, 1H, , Ar-*H*), 8.02 (d, *J* = 4.5 Hz, 1H, , Ar-*H*), 7.69 (t, *J* = 8.0 Hz, 1H, Pyr-*H*, , Ar-*H*), 7.60 (d, *J* = 8.0 Hz, 1H, Pyr-*H*), 7.11 (d, *J* = 8.0 Hz, 1H, Pyr-*H*), 2.67 (s, 3H, C*H*$_3$); **^{13}C NMR** (101 MHz, $CDCl_3$) δ 158.2, 148.9, 144.0, 142.3, 137.2, 135.7, 130.4, 124.9, 121.5, 120.7, 118.1, 24.7; **HRMS** (ESI$^+$) found *m/z* 211.0979 $[M+H]^+$, $C_{12}H_{11}N_4$ requires m/z 211.0978**;** **IR** ν_{max} (neat)/cm^{-1} 3095, 2915,

2361, 1726, 1583, 1530, 1481, 1310, 1448, 1171, 1044, 847, 798; **mp** 130-132 °C (EtOAc); $\mathbf{R_f}$ = 0.09 (80% EtOAc in Petrol).

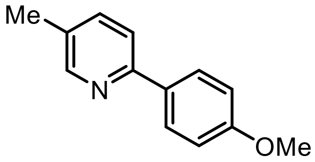

2-(4-methoxyphenyl)-5-methylpyridine (3z)

From the β-nitrile sulfone: Following general procedure **F**, 3-((5-methylpyridin-2-yl)sulfonyl)propanenitrile (46.3 mg, 0.22 mmol, 1.1 equiv.), 4-bromoanisole (25.0 µL, 0.20 mmol, 1.0 equiv.), $Pd(OAc)_2$ (2.2 mg, 0.010 mmol, 5.0 mol%), K_2CO_3 (41.4 mg, 0.30 mmol, 1.5 equiv.), CataCXium A (7.2 mg, 0.020 mmol, 10 mol%) and AcOH (11.5 µL, 0.20 mmol, 1.0 equiv.) in toluene (2.0 mL) were heated at 120 °C for 18 h. The crude reaction mixture was purified by flash column chromatography on silica gel (8% EtOAc in Petrol) to give the title product as an off-white solid (30.8 mg, 77%). *When run at 130 °C, the reaction gave the title product in 86% yield (34.3 mg)*

^{1}H NMR (400 MHz, $CDCl_3$) δ 8.48 – 8.46 (m, 1H, Pyr-*H*), 7.92 (d, *J* = 9.0 Hz, 2H, Ar-*H*), 7.57 (dd, *J* = 8.0, 0.5 Hz, 1H, Pyr-*H*), 7.52 (ddd, *J* = 8.0, 2.5, 0.5 Hz, 1H, Pyr-*H*), 6.99 (d, *J* = 9.0 Hz, 2H, Ar-*H*), 3.86 (s, 3H, OC*H₃*), 2.35 (s, 3H, C*H*₃); **^{13}C NMR** (101 MHz, $CDCl_3$) δ 160.3, 154.7, 150.1, 137.4, 132.3, 130.9, 128.0, 119.4, 114.2, 55.5, 18.3; **LRMS** (ESI$^+$) *m/z* 200.0 $[M+H]^+$; **HRMS** (ESI$^+$) found *m/z* 200.1071 $[M+H]^+$, $C_{13}H_{14}ON$ requires m/z 200.1071; **IR** ν_{max} (neat)/cm^{-1} 3000, 2924, 2836, 2361, 1609, 1584, 1562, 1515, 1477, 1441, 1418, 1377, 1307, 1273, 1246, 1225, 1174, 1143, 1109, 1047, 1026, 848, 820, 791, 757, 750, 719, 668, 643; **mp** 53-55 °C (EtOAc); $\mathbf{R_f}$ = 0.10 (5% EtOAc in Petrol). Data is consistent with literature: C. Liu, W. Yang, *Chem. Comm.* 2009, **41**, 6267-6269.[17]

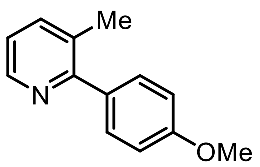

2-(4-methoxyphenyl)-3-methylpyridine (3aa)

From the β-nitrile sulfone: Following general procedure **F**, 3-((3-methylpyridin-2-yl)sulfonyl)propanenitrile (46.3 mg, 0.22 mmol, 1.1 equiv.), 4-bromoanisole (25 µL, 0.20 mmol, 1.0 equiv.), $Pd(OAc)_2$ (2.2 mg, 0.010 mmol, 5.0 mol%), CataCXium A (7.2 mg, 0.020 mmol, 10 mol%), K_2CO_3 (41.4 mg, 0.30 mmol, 1.5 equiv.) and AcOH (11.4 µL, 0.20 mmol, 1.0 equiv.) in toluene (2.0 mL) were heated at 130 °C for 18 h. The crude reaction mixture was purified by flash column chromatography (15% EtOAc in Petrol) to give the title compound as a colourless oil (37.7 mg, 95%). *When run at 120 °C, the reaction gave the title product in a 37% yield (15.0 mg).*

^{1}H NMR (400 MHz, $CDCl_3$) δ 8.51 (dd, *J* = 5.0, 1.5 Hz, 1H, Pyr-*H*), 7.55 (ddd, *J* = 8.0, 1.5, 1.0 Hz, 1H, Pyr-*H*), 7.49 (d, *J* = 8.5 Hz, 2H, Ar-*H*), 7.14 (dd, *J* = 8.0, 5.0 Hz, 1H, Pyr-*H*), 6.98 (d, *J* = 8.5 Hz, 2H, Ar-*H*), 3.86 (s, 3H, CO_2*Me*), 2.37 (s, 3H, Pyr-*Me*); **^{13}C NMR** (101 MHz, $CDCl_3$) δ 159.5, 158.5, 147.1, 138.7, 133.3, 130.8, 130.4, 121.8, 113.7, 55.5, 20.4; **LRMS** (ESI$^+$) *m/z* 200.0 $[M+H]^+$; **HRMS** (ESI$^+$) found *m/z* 200.1072 $[M+H]^+$, $C_{13}H_{14}ON$ requires m/z 200.1070; **IR** ν_{max} (neat)/cm^{-1} 3047, 2957, 2933, 2915, 2836, 2361, 2341, 1610, 1580, 1514, 1460, 1442, 1422, 1302, 1246, 1176, 1109, 1043, 1025, 838, 791, 773; **R_f** = 0.14 (10% EtOAc in Petrol). Data is consistent with literature: W. Lin, J. Tang, S. Li, X. Zheng, M. Yuan, B. Xu, W. Jiang, H. Fu, R. Li, H. Chen, *Org. Lett.* 2020, 22, 20, 7814–7819.[18]

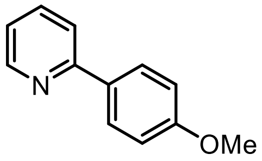

2-(4-methoxyphenyl)pyridine (3ab)

From the β-nitrile sulfone: Following general procedure **F**, 3-(pyridin-2-ylsulfonyl)propanenitrile (43.2 mg, 0.22 mmol, 1.1 equiv.), 4-bromoanisole (25 µL, 0.20 mmol, 1.0 equiv.), $Pd(OAc)_2$ (2.2 mg, 0.010 mmol, 5.0 mol%), K_2CO_3 (41.4 mg, 0.30 mmol, 1.5 equiv.), CataCXium A (7.2 mg, 0.020 mmol, 10 mol%) and AcOH (11.5 µL, 0.20 mmol, 1.0 equiv.) in toluene (2.0 mL) were heated at 130 °C for 18 h. The crude reaction mixture was purified by flash column chromatography on silica gel (12% EtOAc in Petrol) to give the title product as an off-white crystalline solid (27.3 mg, 74%).

^{1}H NMR (400 MHz, $CDCl_3$) δ 8.65 (ddd, *J* = 5.0, 2.0, 1.0 Hz, 1H, Pyr-*H*), 7.96 (d, *J* = 9.0 Hz, 2H, Ar-*H*), 7.71 (ddd, *J* = 8.0, 7.0, 2.0 Hz, 1H, Pyr-*H*), 7.66 (dt, *J*= 8.0, 1.0 Hz 1H, Pyr-*H*), 7.17 (ddd, *J* = 7.0, 5.0, 1.0 Hz, 1H, Pyr-*H*), 7.00 (d, *J* = 9.0 Hz, 2H, Ar-*H*), 3.86 (s, 3H, OC*H*$_3$); **^{13}C NMR** (101 MHz, $CDCl_3$) δ 160.6, 157.3, 149.7, 136.8, 132.2, 128.3, 121.5, 119.9, 114.2, 55.5; **LRMS** (ESI$^+$) *m/z* 186.1 [M+H]$^+$; **HRMS** (ESI$^+$) found *m/z* 186.0913 [M+H]$^+$, $C_{12}H_{12}ON$ requires m/z 186.0913; **IR** ν_{max} (neat)/ cm^{-1} 2359, 2255, 2160, 2035, 1971, 1598, 1514, 1465, 1383, 1310, 1252, 1178; **mp** 49-51 °C (EtOAc); **R$_f$** = 0.27 (20% EtOAc in Petrol). Data is consistent with literature: T. Markovic, B. N. Rocke, D. C. Blakemore, V. Mascitti and M. C. Willis, *Chem. Sci.*, 2017, **8**, 4437-4442.[9]

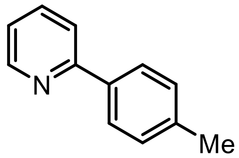

2-(p-tolyl)pyridine (3ac)

From the β-nitrile sulfone: Following general procedure **F**, 3-(pyridin-2-ylsulfonyl)propanenitrile (43.2 mg, 0.22 mmol, 1.1 equiv.), 4-bromotoluene (34.2 mg, 0.20 mmol, 1.0 equiv.), $Pd(OAc)_2$ (2.2 mg, 0.010 mmol, 5.0 mol%), K_2CO_3 (41.4 mg, 0.30 mmol, 1.5 equiv.), CataCXium A (7.2 mg, 0.020 mmol, 10 mol%) and AcOH (11.5 µL, 0.20 mmol, 1.0 equiv.) in toluene (2.0 mL) were heated at 130 °C for 18 h. The crude reaction mixture was purified by flash column chromatography on silica gel (6% EtOAc in Petrol) to give the title product as a colourless oil (28.0 mg, 83%). *When run at 120 °C, the reaction gave the title product in a 52% yield (17.6 mg)*

From the β-methylester sulfone: Following a stoichiometrically *modified* version of general procedure **F**, methyl 3-(pyridin-2-ylsulfonyl)propanoate (68.8 mg, 0.30 mmol, 1.5 equiv.), 4-bromotoluene (34.2 mg, 0.20 mmol, 1.0 equiv.), $Pd(OAc)_2$ (2.2 mg, 0.010 mmol, 5.0 mol%), K_2CO_3 (55.3 mg, 0.40 mmol, 2.0 equiv.), AcOH (17.2 µL, 0.30 mmol, 1.5 equiv.) and CataCXium A (7.2 mg, 0.020 mmol, 10 mol%) in toluene (2.0 mL) were heated at 130 °C for 18 h. The crude reaction mixture was purified by flash column chromatography on silica gel (6% EtOAc in Petrol) to give the title product as a colourless oil (30.7 mg, 91%).

Following general procedure **G**, methyl 3-(pyridin-2-ylsulfonyl)propanoate (68.8 mg, 0.30 mmol, 1.5 equiv.), 4-bromotoluene (34.2 mg, 0.20 mmol, 1.0 equiv.), $Pd(OAc)_2$ (2.2 mg, 0.010 mmol, 5.0 mol%), K_2CO_3 (55.3 mg, 0.40 mmol, 2.0 equiv.) and CataCXium A (7.2 mg, 0.020 mmol, 10 mol%) in toluene (2.0 mL) were heated at 130 °C for 18 h. The crude reaction mixture was purified by flash column chromatography on silica gel (6% EtOAc in Petrol) to give the title product as a colourless oil (9.7 mg, 29%).

Following general procedure **F**, methyl 3-(pyridin-2-ylsulfonyl)propanoate (50.4 mg, 0.22 mmol, 1.1 equiv.), 4-bromotoluene (34.2 mg, 0.20 mmol, 1.0 equiv.), $Pd(OAc)_2$ (2.2 mg, 0.010 mmol, 5.0 mol%), K_2CO_3 (41.4 mg, 0.30 mmol, 1.5 equiv.), CataCXium A (7.2 mg, 0.02 mmol, 10 mol%) and AcOH (11.5 µL, 0.20 mmol, 1.0 equiv.) in toluene (2.0 mL) were heated at 120 °C for 18 h. The crude reaction mixture was purified by flash column chromatography on silica gel (6% EtOAc in Petrol) to give the title product as a colourless oil (22.6 mg, 67%). *When run at 130 °C, the reaction gave the title product in a 40% yield (13.5 mg).*

From the diethyl 2-(pyridin-2-ylsulfonyl)succinate: Following general procedure **G**, diethyl 2-(pyridin-2-ylsulfonyl)succinate (94.6 mg, 0.30 mmol, 1.5 equiv.), 4-bromotoluene (34.2 mg, 0.20 mmol, 1.0 equiv.), $Pd(OAc)_2$ (2.2 mg, 0.010 mmol, 5.0 mol%), K_2CO_3 (55.3 mg, 0.40 mmol, 2.0 equiv.) and CataCXium A (7.2 mg, 0.020 mmol, 10 mol%) in toluene (2.0 mL) were heated at 130 °C for 18 h. The crude reaction mixture was purified by flash column chromatography on silica gel (6% EtOAc in Petrol) to give the title product as a colourless oil (32.4 mg, 96%). *When run at 120 °C, the reaction gave the title product in a 29% yield (9.8 mg)*

^{1}H NMR (400 MHz, $CDCl_3$) δ 8.69 – 8.67 (m, 1H, Pyr- *H*), 7.90 (d, *J*= 8.0 Hz, 2H, Tol-*H*), 7.75 – 7.67 (m, 2H, Pyr-*H*), 7.28 (d, *J*= 8.0 Hz, 2H, Tol-*H*), 7.20 (ddd, *J* = 6.5, 5.0, 2.0 Hz, 1H, Pyr-*H*), 2.41 (s, 3H, C*H*$_3$); **^{13}C NMR** (101 MHz, $CDCl_3$) δ 157.6, 149.7, 139.1, 136.8 (2C), 129.6, 126.9, 121.9, 120.4, 21.4; **LRMS** (ESI$^+$) *m/z* 170.0 [M+H]$^+$; **HRMS** (ESI$^+$) found *m/z* 170.0964 [M+H]$^+$, $C_{12}H_{12}N$ requires m/z 170.0964; **IR** ν_{max} (neat)/cm^{-1} 3009, 2921, 2862, 2361, 2340, 1735, 1614, 1588, 1563, 1515, 1466, 1433, 1299, 1266, 1185, 1154, 1109, 1059, 1038, 1017, 989, 830, 773, 743, 722, 707, 678, 643, 621; **R$_f$** = 0.43 (20% EtOAc in Petrol). Data is consistent with literature: T. Markovic, B. N. Rocke, D. C. Blakemore, V. Mascitti and M. C. Willis, *Chem. Sci.*, 2017, **8**, 4437-4442.[9]

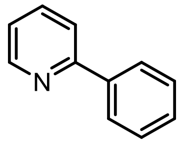

2-phenylpyridine (3ad)

From the β-nitrile sulfone: Following general procedure **F**, 3-(pyridin-2-ylsulfonyl)propanenitrile (43.2 mg, 0.22 mmol, 1.1 equiv.), bromobenzene (21 µL, 0.20 mmol, 1.0 equiv.), $Pd(OAc)_2$ (2.2 mg, 0.010 mmol, 5.0 mol%), K_2CO_3 (41.4 mg, 0.30 mmol, 1.5 equiv.), CataCXium A (7.2 mg, 0.020 mmol, 10 mol%) and AcOH (11.5 µL, 0.20 mmol, 1.0 equiv.) in toluene (2.0 mL) were heated at 130 °C for 18 h. The crude reaction mixture was purified by flash column chromatography on silica gel (8% EtOAc in Petrol) to give the title product as a colourless oil (18.4 mg, 59%).

^{1}**H NMR** (400 MHz, $CDCl_3$) δ 8.72 – 8.68 (m, 1H, Pyr-*H*), 8.01 – 7.97 (m, 2H, Pyr-), 7.78 – 7.71 (m, 2H, Ar-*H*), 7.52 – 7.38 (m, 3H, Ar-*H*), 7.23 (ddd, *J* = 6.5, 5.0, 2.0 Hz, 1H, Pyr-*H*); ^{13}**C NMR** (101 MHz, $CDCl_3$) δ 157.6, 149.8, 139.5, 136.9, 129.1, 128.9, 127.0, 122.2, 120.7; **LRMS** (ESI^+) *m/z* 156.0 $[M+H]^+$; **HRMS** (ESI^+) found *m/z* 156.0807 $[M+H]^+$, $C_{11}H_{10}N$ requires m/z 156.0808; **IR** ν_{max} (neat)/ cm^{-1} 3058, 3008, 2923, 2363, 2335, 1970, 1584, 1499, 1469, 1448, 1424, 1325, 1295, 1266, 1151, 1123, 1073, 990, 922, 799, 745, 694; $\mathbf{R_f}$ = 0.31 (20% EtOAc in Petrol). Data is consistent with literature: J. Wei, H. Liang, C. Ni, R. Sheng, J. Hu, *Org. Lett.,* 2019, **21**, 4, 937-940.[1]

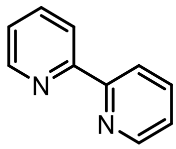

2,2'-bipyridine (3ae)

From the β-nitrile sulfone: Following general procedure **F**, 3-(pyridin-2-ylsulfonyl)propanenitrile (43.2 mg, 0.22 mmol, 1.1 equiv.), $Pd(OAc)_2$ (2.2 mg, 0.010 mmol, 5.0 mol%), CataCXium A (7.2 mg, 0.020 mmol, 10 mol%), K_2CO_3 (41.4 mg, 0.30 mmol, 1.5 equiv.), 2-bromopyridine (19 µL, 0.20 mmol, 1.0 equiv.) and AcOH (11.4 µL, 0.20 mmol, 1.0 equiv.) in toluene (2.0 mL) were heated at 120 °C for 18 h. The crude reaction mixture was purified by flash column chromatography (5-20% EtOAc in Petrol) to give the title compound as a white solid (28.0 mg, 90%).

From the β-methylester sulfone: Following general procedure **G**, methyl 3-(pyridin-2-ylsulfonyl)propanoate (68.8 mg, 0.30 mmol, 1.5 equiv.), $Pd(OAc)_2$ (2.2 mg, 0.010 mmol, 5.0 mol%), CataCXium A (7.2 mg, 0.020 mmol, 10 mol%), K_2CO_3 (55.2 mg, 0.40 mmol, 2.0 equiv.), and 2-bromopyridine (19 µL, 0.20 mmol, 1.0 equiv.) in toluene (2.0 mL) were heated at 130 °C for 18 h. The crude reaction mixture was purified by flash column chromatography (5-10% EtOAc in Petrol) to give the title compound as a white solid (16.0 mg, 51%). *When run at 120 °C, the reaction gave the title product in a 35% yield (11.0 mg).*

^{1}**H NMR** (400 MHz, $CDCl_3$) δ 8.68 (d, *J* = 4.5 Hz, 2H, Ar-*H*), 8.39 (d, *J* = 8.0 Hz, 2H, Ar-*H*), 7.81 (td, *J* = 7.5 Hz, 2.0 Hz, 2H, Ar-*H*), 7.30 (ddd, *J* = 7.5 Hz, 4.5 Hz, 1.0 Hz, 2H, Ar-*H*); ^{13}**C NMR** (101 MHz, $CDCl_3$) δ 156.3, 149.3, 137.0, 123.8, 121.2; **LRMS** (ESI^+) *m/z* 157.0 $[M+H]^+$; **HRMS** (ESI^+) found *m/z* 157.0759 $[M+H]^+$, $C_{10}H_9N_2$ requires m/z 157.0760; **IR** ν_{max} (neat)/cm^{-1} 3052, 3007, 1581, 1560, 1455, 1419, 1255, 1090, 755; **mp** 65-68 °C (CH_2Cl_2); $\mathbf{R_f}$ = 0.24 (20% EtOAc in Petrol). Data is consistent with literature: T. Markovic, B. N. Rocke, D. C. Blakemore, V. Mascitti and M. C. Willis, *Chem. Sci.*, 2017, **8**, 4437-4442.[9]

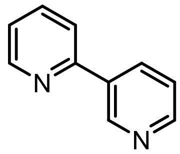

2,3'-bipyridine (3af)

From the β-nitrile sulfone: Following general procedure **F**, 3-(pyridin-2-ylsulfonyl)propanenitrile (43.2 mg, 0.22 mmol, 1.1 equiv.), 3-bromopyridine (19.3 µL, 0.20 mmol, 1.0 equiv.), $Pd(OAc)_2$ (2.2 mg, 0.010 mmol, 5.0 mol%), K_2CO_3 (41.4 mg, 0.30 mmol, 1.5 equiv.), CataCXium A (7.2 mg, 0.020 mmol, 10 mol%) and AcOH (11.5 µL, 0.20 mmol, 1.0 equiv.) in toluene (2.0 mL) were heated at 130 °C for 18 h. The reaction mixture was filtered through a silica plug, washing with copious EtOAc and MeOH, before being concentrated *in vacuo*. The crude residue was purified by flash column chromatography on silica gel (75% EtOAc in Petrol) to give the title product as a pale yellow oil (28.9 mg, 93%). *When run at 120 °C, the reaction gave the title product in a 44% yield (13.7 mg).*

From the β-methylester sulfone: Following general procedure **G**, methyl 3-(pyridin-2-ylsulfonyl)propanoate (68.8 mg, 0.30 mmol, 1.5 equiv.), 3-bromopyridine (19.3 µL, 0.20 mmol, 1.0 equiv.), $Pd(OAc)_2$ (2.2 mg, 0.010 mmol, 5.0 mol%), K_2CO_3 (55.3 mg, 0.40 mmol, 2.0 equiv.) and CataCXium A (7.2 mg, 0.020 mmol, 10 mol%) in toluene (2.0 mL) were heated at 130 °C for 18 h. The crude reaction mixture was purified by flash column chromatography on silica gel (75% EtOAc in Petrol) to give the title product as a pale yellow oil (25.4 mg, 81%). *When run at 120 °C, the reaction gave the title product in a 36% yield (11.2 mg).*

^{1}H NMR (400 MHz, $CDCl_3$) δ 9.19 (d, *J* = 2.5 Hz, 1H, Pyr-*H*), 8.72 (ddd, *J* = 5.0, 2.0, 1.0 Hz, 1H, Pyr-*H*), 8.65 (dd, *J* = 5.0, 2.0 Hz, 1H, Pyr-*H*), 8.32 (ddd, *J* = 8.0, 1.5, 2.0 Hz, 1H, Pyr-*H*), 7.82 – 7.72 (m, 2H, Pyr-*H*), 7.40 (dd, *J* = 8.0, 5.0 Hz, 1H, Pyr-*H*), 7.29 (ddd, *J* = 7.0, 5.0, 1.5 Hz, 1H, Pyr-*H*); **^{13}C NMR** (101 MHz, $CDCl_3$) δ 154.9, 150.1, 150.0, 148.3, 137.1, 135.0, 134.4, 123.7, 122.9, 120.7; **LRMS** (ESI^+) m/z 157.0 $[M+H]^+$**;** **HRMS** (ESI^+) found *m/z* 157.0761 $[M+H]^+$, $C_{10}H_9N_2$ requires m/z 157.0760; **IR** v_{max} (neat)/cm^{-1} 3053, 2928, 1588, 1461, 1433, 1407, 1334, 1285, 1192, 1156, 1097, 1068, 1050, 1017, 990, 823, 772, 744, 708, 654, 615; **R_f** = 0.15 (80% EtOAc in Petrol). Data consistent with literature: T. Markovic, B. N. Rocke, D. C. Blakemore, V. Mascitti and M. C. Willis, *Chem. Sci.*, 2017, **8**, 4437-4442.[9]

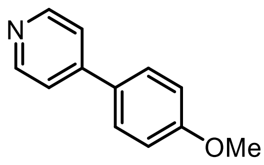

4-(4-methoxyphenyl)pyridine (3ag)

From the β-nitrile sulfone: Following general procedure **F**, 3-(pyridin-4-ylsulfonyl)propanenitrile (43.2 mg, 0.22 mmol, 1.1 equiv.), 4-bromoanisole (25 µL, 0.20 mmol, 1.0 equiv.), $Pd(OAc)_2$ (2.2 mg, 0.010 mmol, 5.0 mol%), K_2CO_3 (41.4 mg, 0.30 mmol, 1.5 equiv.), CataCXium A (7.2 mg, 0.020 mmol, 10 mol%) and AcOH (11.5 µL, 0.20 mmol, 1.0 equiv.) in toluene (2.0 mL) were heated at 130 °C for 18 h. The crude reaction mixture was purified by flash column chromatography on silica gel (35% EtOAc in Petrol) to give the title product as a white crystalline solid (18.3 mg, 49%).

From the β-methylester sulfone: Following general procedure **G**, methyl 3-(pyridin-4-ylsulfonyl)propanoate (68.8 mg, 0.30 mmol, 1.5 equiv.), 4-bromoanisole (25 µL, 0.20 mmol, 1.0 equiv.), $Pd(OAc)_2$ (2.2 mg, 0.010 mmol, 5.0 mol%), K_2CO_3 (55.3 mg, 0.40 mmol, 2.0 equiv.) and CataCXium A (7.2 mg, 0.020 mmol, 10 mol%) in toluene (2.0 mL) were heated at 120 °C for 18 h. The crude reaction mixture was purified by flash column chromatography on silica gel (35% EtOAc in Petrol) to give the title product as a white solid (33.0 mg, 89%).

^{1}H NMR (400 MHz, $CDCl_3$) δ 8.62 (dd, *J* = 4.0, 2.0 Hz, 2H, Pyr-*H*), 7.60 (d, *J* = 9.0 Hz, 2H, Ar-*H*), 7.47 (dd, *J* = 4.5, 1.5 Hz, 2H, Pyr-*H*), 7.01 (d, *J* = 9.0 Hz, 2H, Ar-*H*), 3.87 (s, 3H, OCH_3); **^{13}C NMR** (101 MHz, $CDCl_3$) δ 160.7, 150.3, 147.9, 130.5, 128.3, 121.2, 114.7, 55.5; **LRMS** (ESI$^+$) *m/z* 186.0 $[M+H]^+$; **HRMS** (ESI$^+$) found *m/z* 186.0915 $[M+H]^+$, $C_{12}H_{12}ON$ requires m/z 186.0913; **IR** ν_{max} (neat)/cm^{-1} 3080, 3023, 2965, 2927, 2846, 2360, 1738, 1605, 1579, 1542, 1523, 1489, 1287, 1257, 1228, 1094, 1035, 809; **mp** 74-76 °C (EtOAc); **R_f** = 0.15 (40% EtOAc in Petrol). Data is consistent with literature: T. Markovic, B. N. Rocke, D. C. Blakemore, V. Mascitti and M. C. Willis, *Chem. Sci.*, 2017, **8**, 4437-4442.[9]

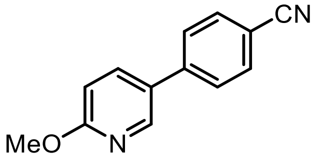

4-(6-methoxypyridin-3-yl)benzonitrile (3ah)

From the β-nitrile sulfone: Following general procedure **F**, 3-((6-methoxypyridin-3-yl)sulfonyl)propanenitrile (49.8 mg, 0.22 mmol, 1.1 equiv.), 4-bromobenzonitrile (36.4 mg, 0.20 mmol, 1.0 equiv.), $Pd(OAc)_2$ (2.2 mg, 0.010 mmol, 5.0 mol%), K_2CO_3 (41.4 mg, 0.30 mmol, 1.5 equiv.), CataCXium A (7.2 mg, 0.020 mmol, 10 mol%) and AcOH (11.5 µL, 0.20 mmol, 1.0 equiv.) in toluene (2.0 mL) were heated at 120 °C for 18 h. The crude reaction mixture was purified by flash column chromatography on silica gel (4-5% EtOAc in Petrol) to give the title product as a white solid (40.1 mg, 95%).

From the β-methylester sulfone: Following general procedure **G**, methyl 3-((6-methoxypyridin-3-yl)sulfonyl)propanoate (77.8 mg, 0.30 mmol, 1.5 equiv.), 4-bromobenzonitrile (36.4 mg, 0.20 mmol, 1.0 equiv.), $Pd(OAc)_2$ (2.2 mg, 0.010 mmol, 5.0 mol%), K_2CO_3 (55.3 mg, 0.40 mmol, 2.0 equiv.) and CataCXium A (7.2 mg, 0.020 mmol, 10 mol%) in toluene (2.0 mL) were heated at 120 °C for 18 h. The crude reaction mixture was purified by flash column chromatography on silica gel (7% EtOAc in Petrol) to give the title product as a white solid (27.8 mg, 66%).

^{1}H NMR (400 MHz, $CDCl_3$) δ 8.40 (dd, *J* = 2.5, 1.0 Hz, 1H, Pyr-*H*), 7.79 (dd, *J* = 8.5, 2.65 Hz, 1H, Pyr-*H*), 7.75 – 7.71 (m, 2H, Ar-*H*), 7.65 – 7.60 (m, 2H, Ar-*H*), 6.85 (dd, *J* = 8.5, 1.0 Hz, 1H, Pyr-*H*), 3.99 (s, 3H, OC*H*$_3$); **^{13}C NMR** (101 MHz, $CDCl_3$) δ 164.6, 145.6, 142.6, 137.4, 132.9, 128.2, 127.2, 118.9, 111.5, 111.1, 53.9; **LRMS** (ESI$^+$) m/z 211.3 $[M+H]^+$**;** **HRMS** (ESI$^+$) found *m/z* 211.0867 $[M+H]^+$, C_{13} $H_{11}ON_2$ requires m/z 211.0866**;** **IR** v_{max} (neat)/cm^{-1} 2943, 2359, 2224, 1604, 1573, 1483, 1430, 1416, 1372, 1288, 1251, 1175, 1142, 1042, 1042, 1017, 999, 934, 821, 664; **mp** 113-115 °C (EtOAc); **R$_f$** = 0.16 (5% EtOAc in Petrol). Data is consistent with literature: N. A. Isley, F. Gallou and B. H. Lipshutz, 2013, **135**, 17707-17710.[19]

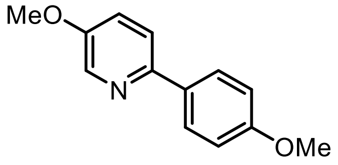

5-methoxy-2-(4-methoxyphenyl)pyridine (3ai)

From the β-nitrile sulfone: Following general procedure **F**, 3-((5-methoxypyridin-2-yl)sulfonyl)propanenitrile (49.8 mg, 0.22 mmol, 1.1 equiv.), 4-bromoanisole (25.0 µL, 0.20 mmol, 1.0 equiv.), Pd(OAc)2 (2.2 mg, 0.010 mmol, 5.0 mol%), K2CO3 (41.4 mg, 0.30 mmol, 1.5 equiv.), CataCXium A (7.2 mg, 0.020 mmol, 10 mol%) and AcOH (11.5 µL, 0.20 mmol, 1.0 equiv.) in toluene (2.0 mL) were heated at 130 °C for 18 h. The reaction mixture was filtered through a silica plug, washing with EtOAc (volume) and MeOH (volume) before being concentrated *in vacuo*. The crude residue was purified by flash column chromatography on silica gel (0%-10% EtOAc in Petrol) to give the title product as a white solid (42.0 mg, 98%). *When run at 120 °C, the reaction gave the title product in a 60% yield (25.9 mg).*

From the β-methylester sulfone: Following general procedure **G**, methyl 3-((5-methoxypyridin-2-yl)sulfonyl)propanoate (77.8 mg, 0.30 mmol, 1.5 equiv.), 4-bromoanisole (25 µL, 0.20 mmol, 1.0 equiv.), $Pd(OAc)_2$ (2.2 mg, 0.010 mmol, 5.0 mol%), CataCXium A (7.2 mg, 0.020 mmol, 10 mol%) and K_2CO_3 (55.3 mg, 0.40 mmol, 2.0 equiv.) in toluene (2.0 mL) were heated at 130 °C for 18 h. The crude reaction mixture was purified by flash column chromatography (15% EtOAc in Petrol) to give the title compound as a crystalline white solid (26.5 mg, 62%). *When run at 120 °C, the reaction gave the title product in a 37% yield (16.0 mg).*

^{1}H NMR (400 MHz, $CDCl_3$) δ 8.35 (dd, *J* = 3.0, 1.0 Hz, 1H, Pyr-*H*), 7.86 (d, *J* = 9.0 Hz, 2H, Ar-*H*), 7.59 (dd, *J* = 8.5, 1.0 Hz, 1H, Pyr-*H*), 7.24 (dd, *J* = 8.5, 3.0 Hz, 1H, Pyr-*H*), 6.98 (d, *J* = 9.0 Hz, 2H, Ar-*H*), 3.88 (s, 3H, OC*H*$_3$), 3.85 (s, 3H, OC*H*$_3$); **^{13}C NMR** (101 MHz, $CDCl_3$) δ 160.0, 154.5, 150.1, 136.97, 132.0, 127.7, 121.6, 120.2, 114.2, 55.8, 55.5; **LRMS** (ESI$^+$) m/z 216.0 [M+H]$^+$; **HRMS** (ESI$^+$) found m/z 216.1019 [M+H]$^+$, $C_{13}H_{14}NO_2$ requires m/z 216.1019; **IR** ν_{max} (neat)/cm^{-1} 2964, 2942, 2841, 1607, 1480, 1262, 1247, 1181, 1050, 1016, 757; **mp** 111-115 °C (CH_2Cl_2); **R$_f$** = 0.27 (20% EtOAc in Petrol).

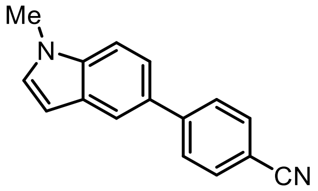

4-(1-methyl-1H-indol-5-yl)benzonitrile (3aj)

From the β-nitrile sulfone: Following general procedure **F**, 3-((1-methyl-1H-indol-5-yl)sulfonyl)propanenitrile (54.6 mg, 0.22 mmol, 1.1 equiv.), 4-benzonitrile (36.4 mg, 0.20 mmol, 1.0 equiv.), $Pd(OAc)_2$ (2.2 mg, 0.010 mmol, 5.0 mol%), CataCXium A (7.2 mg, 0.020 mmol, 10 mol%), K_2CO_3 (41.4 mg, 0.30 mmol, 1.5 equiv.) and AcOH (11.4 µL, 0.20 mmol, 1.0 equiv.) in toluene (2.0 mL) were heated at 130 °C for 18 h. The crude reaction mixture was purified by flash column chromatography (5% EtOAc in Petrol) to give the title compound as an off-white solid (29.4 mg, 63%).

^{1}H NMR (400 MHz, $CDCl_3$) δ 7.86 (dd, *J* = 2.0, 1.0 Hz, 1H, Indole-*H*), 7.77 – 7.69 (m, 4H, Ar-*H*), 7.47 (dd, *J* = 8.5, 2.0 Hz, 1H, Indole-*H*), 7.41 (dt, *J* = 8.5, 1.0 Hz, 1H, Indole-*H*), 7.12 (d, *J* = 3.0 Hz, 1H, Indole-*H*), 6.56 (dd, *J* = 3.0, 1.0 Hz, 1H, Indole-*H*), 3.84 (s, 3H, N-*Me*); **^{13}C NMR** (101 MHz, $CDCl_3$) δ 147.3, 137.0, 132.6, 130.8, 130.2, 129.2, 127.9, 121.2, 120.0, 119.5, 110.0, 109.8, 101.8, 33.1; **LRMS** (ESI^+) *m/z* 233.0 $[M+H]^+$; **HRMS** (ESI^+) found *m/z* 233.1073 $[M+H]^+$, $C_{16}H_{13}N_2$ requires m/z 233.1073**;** **IR** ν_{max} (neat)/cm^{-1} 2960, 2927, 2359, 2343, 2225, 1712, 1600, 1513, 1335, 1172, 1160, 807, 799, 763, 734, 723; **mp** 185-187 °C (EtOAc); **R_f** = 0.25 (8% EtOAc in Petrol).

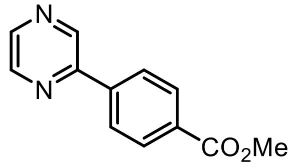

methyl 4-(pyrazin-2-yl)benzoate (3ak)

From the β-nitrile sulfone: Following general procedure **F**, 3-(pyrazin-2-ylsulfonyl)propanenitrile (43.5 mg, 0.22 mmol, 1.1 equiv.), methyl 4-bromobenzoate (43 mg, 0.20 mmol, 1.0 equiv.), $Pd(OAc)_2$ (2.2 mg, 0.010 mmol, 5.0 mol%), K_2CO_3 (41.5 mg, 0.30 mmol, 1.5 equiv.), CataCXium A (7.2 mg, 0.020 mmol, 10 mol%) and AcOH (11.5 µL, 0.20 mmol, 1.0 equiv.) in toluene (2.0 mL) were heated at 120 °C for 18 h. The crude reaction mixture was purified by flash column chromatography on silica gel (10-20% EtOAc in Petrol) to give the title product as a yellow solid (35.0 mg, 82%).

^{1}H NMR (400 MHz, $CDCl_3$) δ 9.08 (s, 1H, Ar-*H*), 8.68 – 8.66 (m, 1H, Ar-*H*), 8.57 (d, *J* = 2.5 Hz, 1H, Ar-*H*), 8.18 (d, *J* = 8.5 Hz, 2H, Ar-*H*), 8.10 (d, *J* = 8.5 Hz, 2H, Ar-*H*), 3.96 (s, 3H, CO_2CH_3); **^{13}C NMR** (101 MHz, $CDCl_3$) δ 166.8, 151.9, 144.5, 143.7, 142.5, 140.5, 131.5, 130.4, 127.0, 52.5; **LRMS** (ESI^+) m/z 215.0 $[M+H]^+$**;** **HRMS** (ESI^+) found *m/z* 215.0815 $[M+H]^+$, $C_{12}H_{11}N_2O_2$ requires m/z 215.0815; **IR** ν_{max} (neat)/cm^{-1} 2981, 2889, 1725, 1392, 1289, 1153, 1115, 957, 830, 774; **R_f** = 0.35 (30% EtOAc in Petrol); **mp** 148-156 °C (CH_2Cl_2).

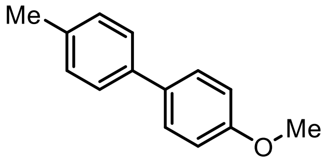

4-methoxy-4'-methyl-1,1'-biphenyl (3al)

From the β-nitrile sulfone: Following general procedure **F**, 3-tosylpropanenitrile (46.0 mg, 0.22 mmol, 1.1 equiv.), 4-bromoanisole (25 µL, 0.20 mmol, 1.0 equiv.), $Pd(OAc)_2$ (2.2 mg, 0.010 mmol, 5.0 mol%), K_2CO_3 (41.4 mg, 0.30 mmol, 1.5 equiv.), CataCXium A (7.2 mg, 0.020 mmol, 10 mol%) and AcOH (11.5 µL, 0.20 mmol, 1.0 equiv.) in toluene (2.0 mL) were heated at 120 °C for 18 h. The crude reaction mixture was purified by flash column chromatography on silica gel (0-1% EtOAc in Petrol) to give the title product as a white solid (25.3 mg, 64%).

From the β-methylester sulfone: Following general procedure **G**, methyl 3-tosylpropanoate (72.7 mg, 0.30 mmol, 1.5 equiv.), 4-bromoanisole (25 µL, 0.20 mmol, 1.0 equiv.), $Pd(OAc)_2$ (2.2 mg, 0.010 mmol, 5.0 mol%), K_2CO_3 (55.3 mg, 0.40 mmol, 2.0 equiv.) and CataCXium A (7.2 mg, 0.020 mmol, 10 mol%) in toluene (2.0 mL) were heated at 130 °C for 18 h. The crude reaction mixture was purified by flash column chromatography on silica gel (0-1% EtOAc in Petrol) to give the title product as a white solid (25.4 mg, 64%). *When run at 120 °C, the reaction gave the title product in a 43% yield (17.0 mg).*

^{1}H NMR (400 MHz, $CDCl_3$) δ 7.52 (d, *J* = 9.0 Hz, 2H, Ar-*H*), 7.46 (d, *J*= 8.0 Hz, 2H, Ar-*H*), 7.23 (d, *J*= 8.0 Hz, 2H, Ar-*H*), 6.98 (d, *J* = 9.0 Hz, 2H, Ar-*H*), 3.85 (s, 3H, Ar-OCH_3), 2.39 (s, 3H, Ar-CH_3); **^{13}C NMR** (101 MHz, $CDCl_3$) δ 159.1, 138.1, 136.5, 133.9, 129.6, 128.1, 126.7, 114.3, 55.5, 21.1; **HRMS** (APCI) found *m/z* 199.1121 $[M+H]^+$, $C_{14}H_{15}O$ requires m/z 199.1117**;** **IR** ν_{max} (neat)/ cm^{-1} 2926, 2839, 1609, 1401, 1290, 1247, 1177, 1040, 906, 842, 808, 129, 650; **mp** 96-98 °C (EtOAc); **R_f** = 0.37 (1% EtOAc in Petrol). Data is consistent with literature: C. Fricke, G. J. Sherborne, I. Funes-Ardoiz, E. Senol, S. Guven and F. Schoenebeck, *Angewandte Chemie International Edition*, 2019, **58**, 17788-17795. [20]

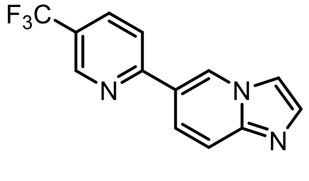

6-(5-(trifluoromethyl)pyridin-2-yl)imidazo[1,2-a]pyridine (3am)

From the β-methylester sulfone: Following general procedure **G**, methyl 3-((5-(trifluoromethyl)pyridin-2-yl)sulfonyl)propanoate (89.0 mg, 0.30 mmol, 1.5 equiv.), 6-bromoimidazo[1,2-a]pyridine (40.0 mg, 0.20 mmol, 1.0 equiv.), $Pd(OAc)_2$ (2.2 mg, 0.010 mmol, 5.0 mol%), K_2CO_3 (55.3 mg, 0.40 mmol, 2.0 equiv.) and CataCXium A (7.2 mg, 0.020 mmol, 10 mol%) in toluene (2.0 mL) were heated at 120 °C for 18 h. The crude reaction mixture was purified by flash column chromatography (50-80% EtOAc in Petrol) to give the

title compound as a yellow solid (30.0 mg, 57%). *When run at 130 °C, the reaction gave the title product in a 57% yield (30.0 mg).*

^{1}H NMR (400 MHz, $CDCl_3$) δ 9.04 (t, *J* = 1.0 Hz, 1H, Ar-*H*), 8.93 (dd, *J* = 1.5 Hz, 1.0 Hz, 1H, Ar-*H*), 8.02 (ddd, *J* = 6.0 Hz, 2.5 Hz, 0.5 Hz, 1H, Ar-*H*), 7.82 (d, *J* = 8.5 Hz, 1H, Ar-*H*), 7.78 (dd, *J* = 9.5 Hz, 1.5 Hz, 1H, Ar-*H*), 7.74 (d, *J* = 9.5 Hz, 1H, Ar-*H*), 7.71 (app. s, 2H, Ar-*H*); **^{13}C NMR** (101 MHz, $CDCl_3$) δ 157.4, 146.9 (q, $^3J_{C-F}$ = 4.0 Hz), 145.4, 134.6, 134.4 (q, $^3J_{C-F}$ = 3.5 Hz), 126.0, 125.4 (q, $^2J_{C-F}$ = 33.5 Hz), 123.8, 123.6 (q, $^1J_{C-F}$ = 272.0 Hz), 123.3, 119.3, 117.8, 113.7; **^{19}F NMR** (377 MHz, $CDCl_3$) δ -62.32 (s); **LRMS** (ESI$^+$) *m/z* 264.0 [M+H]$^+$; **HRMS** (ESI$^+$) found *m/z* 264.0742 [M+H]$^+$, $C_{13}H_9N_3F_3$ requires m/z 264.0743; **IR** ν_{max} (neat)/cm^{-1} 3075, 2918, 1607, 1319, 1107, 1086, 812; **mp** 155-159 °C (CH_2Cl_2); **R$_f$** = 0.17 (80% EtOAc in Petrol).

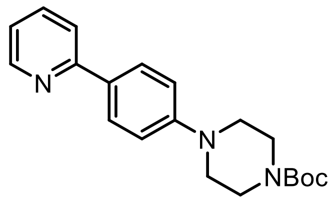

tert-butyl 4-(4-(pyridin-2-yl)phenyl)piperazine-1-carboxylate (3an)

From the β-methylester sulfone: Following general procedure **G**, methyl 3-(pyridin-2-ylsulfonyl)propanoate (68.8 mg, 0.30 mmol, 1.5 equiv.), tert-butyl 4-(4-bromophenyl)piperazine-1-carboxylate (68.3 mg, 0.20 mmol, 1.0 equiv.), $Pd(OAc)_2$ (2.2 mg, 0.010 mmol, 5.0 mol%), CataCXium A (7.2 mg, 0.020 mmol, 10 mol%) and K_2CO_3 (55.2 mg, 0.40 mmol, 2.0 equiv.) in toluene (2.0 mL) were heated at 130 °C for 18 h. The crude reaction mixture was purified by flash column chromatography (10-20% EtOAc in Petrol) to give the title compound as an off-white solid (67.0 mg, 99%). *When run at 120 °C, the reaction gave the title product in a 56% yield (38.0 mg).*

^{1}H NMR (400 MHz, $CDCl_3$) δ 8.63 (d, *J* = 5.0 Hz, 1H, Ar-*H*), 7.93 (d, *J* = 9.0 Hz, 2H, Ar-*H*), 7.73 – 7.62 (m, 2H, Ar-*H*), 7.14 (ddd, *J* = 6.5 Hz, 5.0 Hz, 1.5 Hz, 1H, Ar-*H*), 6.99 (d, *J* = 9.0 Hz, 2H, Ar-*H*), 3.62 – 3.57 (m, 4H, N$CH_2$$CH_2$), 3.26 – 3.20 (m, 4H, N$CH_2$$CH_2$), 1.49 (s, 9H, OC($CH_3$)$_3$); **^{13}C NMR** (101 MHz, $CDCl_3$) δ 157.2, 154.8, 151.8, 149.7, 136.7, 130.9, 127.9, 121.3, 119.7, 116.2, 80.1, 48.9, 28.6; **LRMS** (ESI$^+$) *m/z* 340.2 [M+H]$^+$; HRMS (ESI$^+$) found *m/z* 340.2017 [M+H]$^+$, $C_{20}H_{26}N_3O_2$ requires m/z 340.2020; **IR** ν_{max} (neat)/cm^{-1} 2980, 2890, 1686, 1467, 1422, 1364, 1238, 1164, 1120, 779; **mp** 110-115 °C (CH_2Cl_2); **R$_f$** = 0.18 (20% EtOAc in Petrol); **R$_f$** = 0.37 (40% EtOAc in Petrol).

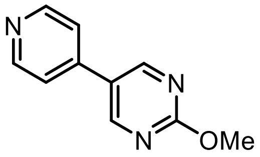

2-methoxy-5-(pyridin-4-yl)pyrimidine (3ao)

From the β-methylester sulfone: Following General procedure **G**, methyl 3-(pyridin-4-ylsulfonyl)propanoate (68.8 mg, 0.30 mmol, 1.5 equiv.), 5-bromo-2-methoxypyrimidine (38.0 mg, 0.20 mmol, 1.0 equiv.), $Pd(OAc)_2$ (2.2 mg, 0.010 mmol, 5.0 mol%), CataCXium A (7.2 mg, 0.020 mmol, 10 mol%) and K_2CO_3 (55.2 mg, 0.40 mmol, 2.0 equiv.) in toluene (2.0 mL) were heated at 130 °C for 18 h. The crude reaction mixture was purified by flash column chromatography (30-50% EtOAc in Petrol) to give the title compound as a white solid (30.0 mg, 80%). *When run at 120 °C, the reaction gave the title product in a 59% yield (22.0 mg).*

^{1}H NMR (400 MHz, $CDCl_3$) δ 8.79 (s, 2H, Ar-*H*), 8.71 (d, *J* = 2.5 Hz, 2H, Ar-*H*), 7.47 (d, *J* = 5.5 Hz, 2H, Ar-*H*), 4.08 (s, 3H, O*CH₃*); **^{13}C NMR** (101 MHz, $CDCl_3$) δ 166.2, 157.6, 150.7, 142.3, 125.6, 120.9, 55.5; **LRMS** (ESI$^+$) *m/z* 188.0 [M+H]$^+$; **HRMS** (ESI$^+$) found *m/z* 188.0819 [M+H]$^+$, $C_{10}H_{10}N_3O$ requires m/z 188.0818; **IR** ν_{max} (neat)/cm^{-1} 3053, 2928, 1610, 1564, 1484, 1416, 1342, 1330, 1035, 824; **mp** 137-141 °C (CH_2Cl_2); **R$_f$** = 0.13 (50% EtOAc in Petrol).

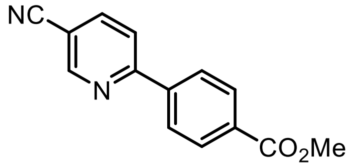

methyl 4-(5-cyanopyridin-2-yl)benzoate (3ap)

From the β-methylester sulfone: Following general procedure **G**, methyl 3-((5-cyanopyridin-2-yl)sulfonyl)propanoate (76.3 mg, 0.30 mmol, 1.5 equiv.), methyl 4-bromobenzoate (43.0 mg, 0.20 mmol, 1.0 equiv.), $Pd(OAc)_2$ (2.2 mg, 0.010 mmol, 5.0 mol%), CataCXium A (7.2 mg, 0.020 mmol, 10 mol%) and K_2CO_3 (55.2 mg, 0.40 mmol, 2.0 equiv.) in toluene (2.0 mL) were heated at 130 °C for 18 h. The crude reaction mixture was purified by flash column chromatography (5-10% EtOAc in Petrol) to give the title compound as a white solid (27.0 mg, 57%). *When run at 120 °C, the reaction gave the title product in a 34% yield (16.0 mg).*

^{1}H NMR (400 MHz, $CDCl_3$) δ 8.98 (dd, *J* = 2.0 Hz, 0.5 Hz, 1H), 8.18 (d, *J* = 8.5 Hz, 2H), 8.12 (d, *J* = 8.5 Hz, 2H), 8.06 (dd, *J* = 8.5, 2.0 Hz, 1H), 7.91 (dd, *J* = 8.5, 0.5 Hz, 1H), 3.96 (s, 3H); **^{13}C NMR** (101 MHz, $CDCl_3$) δ 166.6, 159.4, 152.7, 141.4, 140.2, 132.0, 130.4, 127.5, 120.6, 116.9, 108.9, 52.5; **HRMS** (ESI$^+$) found *m/z* 239.0817 [M+H]$^+$, $C_{14}H_{11}N_2O_2$ requires m/z 239.0815; **IR** ν_{max} (neat)/cm^{-1} 2980, 2889, 1719, 1380, 1271, 1102, 954, 869, 748; **mp** 155-160 °C (CH_2Cl_2); **R$_f$** = 0.10 (10% EtOAc in Petrol). Data is consistent with literature: V. Bonnet, F. Mongin, F. Trecourt, G. Gueguiner and P. Knochel, *Tetrahedron*, 2002, **58**, 4429-4438.[21]

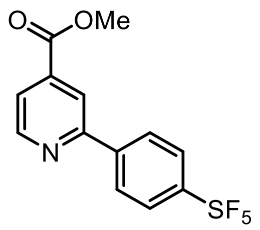

methyl 2-(4-(pentafluoro-l6-sulfaneyl)phenyl)isonicotinate (3aq)

From the β-methylester sulfone: Following general procedure **G**, methyl 2-((3-methoxy-3-oxopropyl)sulfonyl)isonicotinate (86.2 mg, 0.30 mmol, 1.5 equiv.), 4-bromophenylsulfurpentoxide (56.6 mg, 0.20 mmol, 1.0 equiv.), $Pd(OAc)_2$ (2.2 mg, 0.010 mmol, 5.0 mol%), K_2CO_3 (55.3 mg, 0.40 mmol, 2.0 equiv.) and CataCXium A (7.2 mg, 0.020 mmol, 10 mol%) in toluene (2.0 mL) were heated at 120 °C for 18 h. The crude reaction mixture was purified by flash column chromatography on silica gel (1-5% EtOAc in Petrol) to give the title product as a white crystalline solid (54.6 mg, 80%).

^{1}H NMR (500 MHz, CD_3CN) δ 9.23 (dd, *J* = 2.0, 1.0 Hz, 1H, Pyr-*H*), 8.40 (dd, *J* = 8.5, 2.0 Hz, 1H, Pyr-*H*), 8.27 (d, *J* = 8.5 Hz, 2H, Ar-*H*), 8.04 (dd, *J* = 8.5, 1.0 Hz, 1H, Pyr-*H*), 7.97 (d, *J* = 9.0 Hz, 2H, Ar-*H*), 3.93 (s, 3H, CO_2*Me*); **^{13}C NMR** (126 MHz, CD_3CN) δ 166.3, 158.9, 155.1 (p, *J* = 17.0 Hz), 151.6, 142.5, 139.1, 128.8, 127.4 (p, *J* = 4.5 Hz), 126.5, 121.7, 53.0; **^{19}F NMR** (470 MHz, CD_3CN) δ 83.93 (p, *J* = 147.5 Hz), 62.18 (d, *J* = 147.5 Hz); **LRMS** (ESI^+) *m/z* 340.0 $[M+H]^+$; **HRMS** (ESI^+) found *m/z* 340.0424 $[M+H]^+$, $C_{13}H_{11}NO_2SF_5$ requires m/z 340.0425**;** **IR** ν_{max} (neat)/cm^{-1} 2361, 1716, 1594, 1439, 1302, 1121, 1101, 1022, 976, 830, 783, 764, 739, 665; **mp** 138-140 °C (EtOAc); **R_f** = 0.18 (2% EtOAc in Petrol).

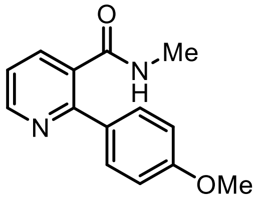

2-(4-methoxyphenyl)-N-methylnicotinamide (3ar)

From the β-methylester sulfone: Following general procedure **G**, methyl 3-((3-(methylcarbamoyl)pyridin-2-yl)sulfonyl)propanoate (85.9 mg, 0.30 mmol, 1.5 equiv.), 4-bromoanisole (25.0 µL, 0.20 mmol, 1.0 equiv.), $Pd(OAc)_2$ (2.2 mg, 0.010 mmol, 5.0 mol%), CataCXium A (7.2 mg, 0.020 mmol, 10 mol%) and K_2CO_3 (55.3 mg, 0.40 mmol, 2.0 equiv.) in toluene (2.0 mL) were heated at 130 °C for 18 h. The crude reaction mixture was purified by flash column chromatography (90-100% EtOAc in Petrol) to give the title compound as a colourless oil that crystallised to a white solid on standing (46.0 mg, 95%).

^{1}H NMR (400 MHz, $CDCl_3$) δ 8.59 (dd, *J* = 5.0, 2.0 Hz, 1H, Pyr-*H*), 7.87 (dd, *J* = 8.0, 2.0 Hz, 1H, Pyr-*H*), 7.59 – 7.50 (m, 2H, Ar-*H*), 7.22 (dd, *J* = 8.0, 5.0 Hz, 1H, Pyr-*H*), 6.98 – 6.88 (m, 2H, Ar-*H*), 5.70 (br. q, *J* = 5.0 Hz, 1H, CON*H*), 3.82 (s, 3H, O*Me*), 2.70 (d, *J* = 5.0 Hz, 3H, NH*Me*); **^{13}C NMR**

(101 MHz, $CDCl_3$) δ 169.5, 160.6, 155.5, 150.5, 137.3, 131.4, 131.0, 130.4, 121.6, 114.1, 55.5, 26.9; **LRMS** (ESI$^+$) *m/z* 243.0 $[M+H]^+$; **HRMS** (ESI$^+$) found *m/z* 243.1129 $[M+H]^+$, $C_{14}H_{15}O_2N_2$ requires m/z 243.1128; **IR** ν_{max} (neat)/cm^{-1} 3061, 3047, 2936, 2454, 2360, 1634, 1609, 1466, 1253, 1178, 1148, 1107, 1041, 841, 782,; **mp** 136-138 °C (EtOAc); $\mathbf{R_f}$ = 0.14 (100% EtOAc in Petrol).

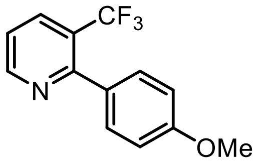

2-(4-methoxyphenyl)-3-(trifluoromethyl)pyridine (3as)

From the β-methylester sulfone: Following general procedure **G**, methyl 3-((3-(trifluoromethyl)pyridin-2-yl)sulfonyl)propanoate (89.2 mg, 0.30 mmol, 1.5 equiv.), 4-bromoanisole (25 µL, 0.20 mmol, 1.0 equiv.), $Pd(OAc)_2$ (2.2 mg, 0.010 mmol, 5.0 mol%), CataCXium A (7.2 mg, 0.020 mmol, 10 mol%) and K_2CO_3 (55.2 mg, 0.40 mmol, 2.0 equiv.) in toluene (2.0 mL) were heated at 130 °C for 18 h. The crude reaction mixture was purified by flash column chromatography (10% EtOAc in Petrol) to give the title compound as a pale yellow oil (50.1 mg, 99%).

^{1}H NMR (400 MHz, $CDCl_3$) δ 8.81 (d, *J* = 5.0 Hz, 1H, Pyr-*H*), 8.05 (d, *J* = 8.0 Hz, 1H, Pyr-*H*), 7.48 (d, *J* = 8.5 Hz, 2H, Ar-*H*), 7.36 (dd, *J* = 8.0, 5.0 Hz, 1H, Pyr-*H*), 6.98 (d, *J* = 8.5 Hz, 2H, Ar-*H*), 3.86 (s, 3H, O*Me*); **^{13}C NMR** (101 MHz, $CDCl_3$) δ 160.3, 158.3 (d, $^4J_{C-F}$ = 2.0 Hz), 151.9, 135.0 (q, $^3J_{C-F}$ = 5.0 Hz), 131.9, 130.3 (q, $^4J_{C-F}$ = 2.0 Hz), 124.8 (q, $^2J_{C-F}$ = 31.5 Hz), 123.9 (q, $^1J_{C-F}$ = 273.0 Hz), 121.4, 113.6, 55.4; **^{19}F NMR** (377 MHz, $CDCl_3$) δ -57.41 (s); **LRMS** (ESI$^+$) m/z 254.0 $[M+H]^+$**;** **HRMS** (ESI$^+$) found m/z 254.0787 $[M+H]^+$, $C_{13}H_{11}NOF_3$ requires m/z 254.0787; **IR** ν_{max} (neat)/cm^{-1} 3056, 3002, 2961, 2934, 2917, 2840, 2359, 2340, 1609, 1588, 1580, 1566, 1515, 1437, 1320, 1301, 1250, 1226, 1161, 1111, 1089, 1043, 1026, 836, 814, 780, 716; $\mathbf{R_f}$ = 0.20 (10% EtOAc in Petrol). Data is consistent with literature: X. Lin, C. Hou, H. Li, Z. Weng, *Chem. Eur. J.* 2016, **22,** 2075-2084.[22]

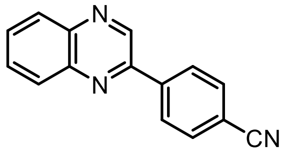

4-(quinoxalin-2-yl)benzonitrile (3at)

From the β-methylester sulfone: Following general procedure **G**, methyl 3-(quinoxalin-2-ylsulfonyl)propanoate (84.1 mg, 0.30 mmol, 1.5 equiv.), 4-bromobenzonitrile (36.4 mg, 0.20 mmol, 1.0 equiv.), $Pd(OAc)_2$ (2.2 mg, 0.010 mmol, 5.0 mol%), CataCXium A (7.2 mg, 0.020 mmol, 10 mol%) and K_2CO_3 (55.3 mg, 0.40 mmol, 2.0 equiv.) in toluene (2.0 mL) were heated at 120 °C for 18 h. The crude reaction mixture was purified by flash column chromatography (15% EtOAc in Petrol) to give the title compound as a white solid (34.9 mg, 75%).

^{1}H NMR (400 MHz, $CDCl_3$) δ 9.36 (s, 1H, Quinox-*H*), 8.86 – 8.83 (m, 2H, Ar-*H*), 8.20 – 8.14 (m, 2H, Quinox-*H*), 7.90 – 7.85 (m, 2H, Ar-*H*), 7.85 – 7.81 (m, 2H, Quinox-*H*); **^{13}C NMR** (101 MHz, $CDCl_3$) δ 149.7, 142.9, 142.4, 142.3, 141.0, 133.0, 131.0, 130.7, 130.0, 129.4, 128.2, 118.6, 113.9; **LRMS** (ESI$^+$) *m/z* 232.0 $[M+H]^+$; **HRMS** (ESI$^+$) found *m/z* 232.0871 $[M+H]^+$, $C_{15}H_{10}N_3$ requires m/z 232.0869; **IR** ν_{max} (neat)/ cm^{-1} 2925, 2210, 1600, 1558, 1503, 1482, 1458, 1015, 1360, 1315, 1267, 1213, 1167, 1143, 1090, 1048, 1020, 959, 844, 765, 733, 669, 657; **mp** 178-180 °C (EtOAc); **R$_f$** = 0.27 (30% EtOAc in Petrol). Data is consistent with literature: A. Y. Dubovtsev, D. V. Dar'in, V. Y. Kukushkin, *J. Org. Chem.* 2020, **85,** 751.[23]

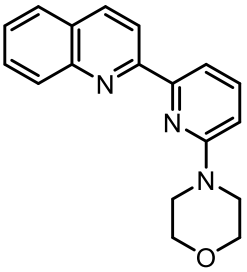

4-(6-(quinolin-2-yl)pyridin-2-yl)morpholine (3au)

From the β-methylester sulfone: Following general procedure **G**, methyl 3-(quinolin-2-ylsulfonyl)propanoate (83.8 mg, 0.30 mmol, 1.5 equiv.), 2-bromo-6-morpholinopyridine (48.6 mg, 0.20 mmol, 1.0 equiv.), $Pd(OAc)_2$ (2.2 mg, 0.010 mmol, 5.0 mol%), K_2CO_3 (55.3 mg, 0.40 mmol, 2.0 equiv.) and CataCXium A (7.2 mg, 0.020 mmol, 10 mol%) in toluene (2.0 mL) were heated at 120 °C for 18 h. The crude reaction mixture was purified by flash column chromatography on silica gel (10% EtOAc in Petrol) to give the title product as a white solid (53.0 mg, 91%).

^{1}H NMR (400 MHz, $CDCl_3$) δ 8.53 (d, *J* = 8.5 Hz, 1H, Ar-*H*), 8.23 (d, *J* = 8.5 Hz, 1H, Ar-*H*), 8.16 (d, *J* = 8.5 Hz, 1H, Ar-*H*), 8.07 (d, *J* = 7.5 Hz, 1H, Ar-*H*), 7.84 (dd, *J* = 8.0, 1.5 Hz, 1H, Ar-*H*), 7.75 – 7.68 (m, 2H, Ar-*H*), 7.53 (ddd, *J* = 8.0, 7.0, 1.0 Hz, 1H, Ar-*H*), 6.74 (d, *J* = 8.5 Hz, 1H, Ar-*H*),

3.93 – 3.87 (m, 4H, (C*H*$_2$)$_2$), 3.68 – 3.62 (m, 4H, (C*H*$_2$)$_2$); **^{13}C NMR** (101 MHz, $CDCl_3$) δ 159.2, 156.9, 154.4, 148.0, 138.7, 136.5, 129.9, 129.5, 128.3, 127.7, 126.6, 119.2, 111.9, 107.5, 67.0, 45.8; **HRMS** (ESI$^+$) found *m/z* 292.1444 [M+H]$^+$, $C_{18}H_{18}ON_3$ requires m/z 292.1444; **IR** v_{max} (neat)/cm^{-1} 3059, 2852, 2360, 2341, 1735, 1591, 1567, 1466, 1447, 1123, 984, 801, 786; **mp** 112-114 °C (EtOAc); **R$_f$** = 0.23 (20% EtOAc in Petrol).

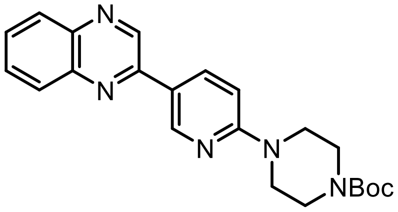

tert-butyl 4-(5-(quinoxalin-2-yl)pyridin-2-yl)piperazine-1-carboxylate (3av)

From the β-methylester sulfone: Following general procedure **G**, methyl 3-(quinoxalin-2-ylsulfonyl)propanoate (84.1 mg, 0.30 mmol, 1.5 equiv.), tert-butyl 4-(5-bromopyridin-2-yl)piperazine-1-carboxylate (68.4 mg, 0.20 mmol, 1.0 equiv.), $Pd(OAc)_2$ (2.2 mg, 0.010 mmol, 5.0 mol%), CataCXium A (7.2 mg, 0.020 mmol, 10 mol%) and K_2CO_3 (55.3 mg, 0.40 mmol, 2.0 equiv.) in toluene (2.0 mL) were heated at 120 °C for 18 h. The crude reaction mixture was purified by flash column chromatography (25% EtOAc in Petrol) to give the title compound as a yellow solid (45.9 mg, 59%).

^{1}H NMR (400 MHz, $CDCl_3$) δ 9.25 (s, 1H, Ar-*H*), 9.00 (dd, *J* = 2.5, 1.0 Hz, 1H, Pyr-*H*), 8.38 (dd, *J* = 9.0, 2.5 Hz, 1H, Pyr-*H*), 8.07 (dt, *J* = 8.0, 2.0 Hz, 2H, Ar-*H*), 7.74 (ddd, *J* = 8.5, 7.0, 1.5 Hz, 1H, Ar-*H*), 7.68 (ddd, *J* = 8.5, 7.0, 1.5 Hz, 1H, Ar-*H*), 6.78 (dd, *J* = 9.0, 1.0 Hz, 1H, Pyr-*H*), 3.74 – 3.56 (m, 8H, N(C*H*$_2$)$_2$(C*H*$_2$)$_2$), 1.49 (s, 9H, C(C*H*$_3$)$_3$); **^{13}C NMR** (101 MHz, $CDCl_3$) δ 159.6, 154.9, 150.0, 147.6, 142.6, 142.5, 141.3, 136.6, 130.4, 129.3, 129.2, 129.0, 122.2, 107.0, 80.2, 44.8, 28.6; **LRMS** (ESI$^+$) *m/z* 336.2 [M- $CH_2C(CH_3)_2$ +H]$^+$; **HRMS** (ESI$^+$) found *m/z* 392.2081 [M+H]$^+$, $C_{22}H_{26}O_2N_5$ requires m/z 392.2081; **IR** v_{max} (neat)/cm^{-1} 2975, 2928, 2856, 1686, 1604, 1420, 1241, 1172, 765; **mp** 105-107°C (EtOAc); **R$_f$** = 0.35 (50% EtOAc in Petrol).

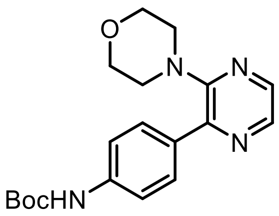

tert-butyl (4-(3-morpholinopyrazin-2-yl)phenyl)carbamate (3aw)

From the β-methylester sulfone: Following general procedure **G**, methyl 3-((3-morpholinopyrazin-2-yl)sulfonyl)propanoate (94.6 mg, 0.30 mmol, 1.5 equiv.), *tert*-butyl (4-bromophenyl)carbamate (54.4 mg, 0.20 mmol, 1.0 equiv.), $Pd(OAc)_2$ (2.2 mg, 0.010 mmol, 5.0 mol%), K_2CO_3 (55.3 mg, 0.40 mmol, 2.0 equiv.) and CataCXium A (7.2 mg, 0.020 mmol, 10 mol%) in toluene (2.0 mL) were heated at 120 °C for 18 h. The crude reaction mixture was purified by flash column chromatography on silica gel (30% EtOAc in Petrol with 1% Et_3N) to give the title product as an off-white crystalline solid (71.2 mg, 99%).

^{1}H NMR (400 MHz, $CDCl_3$) δ 8.16 (d, *J* = 2.5 Hz, 1H, Pyrazine-*H*), 8.07 (d, *J* = 2.5 Hz, 1H, Pyrazine-*H*), 7.90 (d, *J* = 9.0 Hz, 2H, Ar-*H*), 7.46 (d, *J* = 9.0 Hz, 2H, Ar-*H*), 6.58 (br. s, 1H, N*H*), 3.72 – 3.68 (m, 4H), 3.19 – 3.15 (m, 4H), 1.54 (s, 9H, COO(C*H*$_3$)$_3$); **^{13}C NMR** (101 MHz, $CDCl_3$) δ 155.6, 152.7, 144.5, 139.4, 139.2, 136.4, 133.7, 128.6, 118.4, 80.8, 66.7, 49.0, 28.5; **LRMS** (ESI$^+$) *m/z* 357.2 (20%, $[M+H]^+$), 301.1 (80%, $[(M- H_2C=C(CH_3)_2)+H]^+$; **HRMS** (ESI$^+$) found *m/z* 357.19220 $[M+H]^+$, $C_{19}H_{25}N_4O_3$ requires m/z 357.19212**;** **IR** ν_{max} (neat)/ cm^{-1} 3305, 2975, 2854, 2360, 2341, 1727, 1594, 1520, 1419, 1367, 1314, 1265, 1241, 1159, 1117, 1053, 941, 839, 732, 641; **mp** 80-82 °C (EtOAc); **R_f** = 0.24 (40% EtOAc).

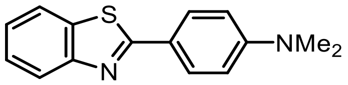

4-(1,3-Benzothiazol-2-yl)-N,N-dimethylaniline (3ax)

From the β-methylester sulfone: Following general procedure **G**, methyl 3-(benzo[d]thiazol-2-ylsulfonyl)propanoate (85.6 mg, 0.30 mmol, 1.5 equiv.), 4-bromo-N,N-dimethylaniline (40.0 mg, 0.20 mmol, 1.0 equiv.), $Pd(OAc)_2$ (2.2 mg, 0.010 mmol, 5.0 mol%), CataCXium A (7.2 mg, 0.020 mmol, 10 mol%) and K_2CO_3 (55.2 mg, 0.40 mmol, 2.0 equiv.) in toluene (2.0 mL) were heated at 130 °C for 18 h. The crude reaction mixture was purified by flash column chromatography (5-10% EtOAc in Petrol) to give the title compound as a white solid (29.0 mg, 57%). *When run at 120 °C, the reaction gave the title product in a 49% yield (25.0 mg).*

^{1}H NMR (400 MHz, $CDCl_3$) δ 7.99 – 7.94 (m, 3H, Ar-*H*), 7.86 – 7.82 (m, 1H, Ar-*H*), 7.43 (ddd, *J* = 8.5 Hz, 7.5 Hz, 1.0 Hz, 1H, Ar-*H*), 7.30 (ddd, *J* = 8.0, 7.5, 1.0 Hz, 1H, Ar-*H*), 6.75 (d, *J* = 9.0 Hz, 2H, Ar-*H*), 3.06 (s, 6H, NC*H*$_3$); **^{13}C NMR** (101 MHz, $CDCl_3$) δ 168.9, 154.6, 152.3, 134.7, 129.0, 126.1, 124.3, 122.4, 121.6, 121.5, 111.8, 40.3; **HRMS** (ESI$^+$) found *m/z* 255.0952 $[M+H]^+$, $C_{15}H_{15}N_2S$ requires m/z 255.0950; **IR** ν_{max} (neat)/cm^{-1} 3058, 2814, 1610, 1486, 1432, 1188, 815, 753, 723; **mp** 143-150 °C (CH_2Cl_2); **R_f** = 0.28 (10% EtOAc in Petrol). Data is consistent with literature: X. Yu, Z. Zhang, R. Song, L. Gou and G. Wang, *Heterocyclic Commun.*, 2020, **26**, 1-5.[24]

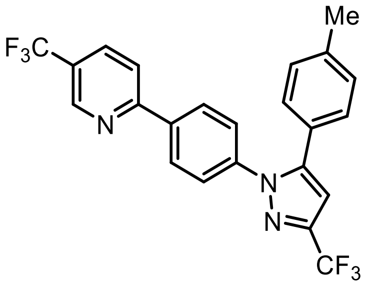

2-(4-(5-(p-tolyl)-3-(trifluoromethyl)-1H-pyrazol-1-yl)phenyl)-5-(trifluoromethyl)pyridine (3ay)

From the β-methylester sulfone: Following general procedure **G**, methyl 3-((5-(trifluoromethyl)pyridin-2-yl)sulfonyl)propanoate (89.2 mg, 0.30 mmol, 1.5 equiv.), 1-(4-bromophenyl)-5-(p-tolyl)-3-(trifluoromethyl)-1H-pyrazole (76.2 mg, 0.20 mmol, 1.0 equiv.), $Pd(OAc)_2$ (2.2 mg, 0.010 mmol, 5.0 mol%), K_2CO_3 (55.3 mg, 0.40 mmol, 2.0 equiv.) and CataCXium A (7.2 mg, 0.020 mmol, 10 mol%) in toluene (2.0 mL) were heated at 120 °C for 18 h. The crude reaction mixture was purified by flash column chromatography on silica gel (5% EtOAc in Petrol) to give the title product as a white solid (73.4 mg, 83%).

^{1}H NMR (400 MHz, $CDCl_3$) δ 8.94 (app. dt, *J* = 2.5, 1.0 Hz, 1H, Pyr-*H*), 8.05 (d, *J* = 8.5 Hz, 2H, Ar-*H*), 8.00 (dd, *J* = 2.5, 8.0 Hz, 1H, Pyr-*H*), 7.85 (d, *J* = 8.5 Hz, 1H, Pyr-*H*), 7.47 (d, *J* = 8.5 Hz, 2H), 7.15 (br. s, 4H, Ar-*H*), 6.75 (s, 1H, Pyrazole-*H*), 2.36 (s, 3H, Ar-C*H*3); **^{1}H NMR** (400 MHz, CD_3CN) δ 8.94 (dq, *J* = 2.5, 1.0 Hz, 1H, Pyr-*H*), 8.14 – 8.08 (m, 3H, Ar-*H* and Pyr-*H*), 7.99 (dt, *J* = 8.5, 1.0 Hz, 1H, Pyr-*H*), 7.43 (d, *J*= 9.0 Hz, 2H, Ar-*H*), 7.23 – 7.13 (m, 4H, Ar-*H*), 6.90 (s, 1H, Pyrazole-*H*), 2.31 (s, 3H, Ar-C*H*3); **^{13}C NMR** (101 MHz, $CDCl_3$) δ 159.4 (q, J^5_{C-F} = 2.0 Hz), 146.8 (q, $^3J_{C-F}$ = 4.0 Hz), 145.1, 143.7 (q, $^2J_{C-F}$ = 33.0 Hz), 140.8, 139.5, 137.7, 134.2 (q, $^3J_{C-F}$ = 3.5 Hz), 129.7, 128.9, 128.1, 126.3, 125.8, 125.4 (q, $^2J_{C-F}$ = 38.5 Hz), 123.9 (q, $^1J_{C-F}$ = 271.5 Hz), 121.4 (q, $^1J_{C-F}$ = 268.5 Hz), 120.2, 105.9 (q, $^3J_{C-F}$ = 2.0 Hz), 21.4; **^{19}F NMR** (377 MHz, CD_3CN) δ -62.72 (s), -62.86 (s); **HRMS** (ESI$^+$) found *m/z* 448.1241 [M+H]$^+$, $C_{23}H_{16}N_3F_6$ requires m/z 448.1243; **IR** $ν_{max}$ (neat)/ cm^{-1} 2926, 2361, 1603, 1472, 1329, 1236, 1163, 1130, 1014, 976, 834, 806; **mp** 98-100 °C (EtOAc); **R_f =** 0.22 (5% EtOAc in Petrol).

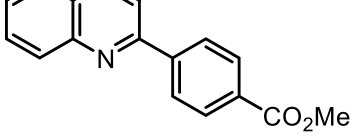

methyl 4-(quinolin-2-yl)benzoate (3az)

From the diethyl succinate sulfone: Following general procedure **G**, diethyl 2-(quinolin-2-ylsulfonyl)succinate (109.6 mg, 0.30 mmol, 1.5 equiv.), methyl 4-bromobenzoate (43.0 mg, 0.20 mmol, 1.0 equiv.), $Pd(OAc)_2$ (2.2 mg, 0.010 mmol, 5.0 mol%), CataCXium A (7.2 mg, 0.020 mmol, 10 mol%) and K_2CO_3 (55.3 mg, 0.40 mmol, 2.0 equiv.) in toluene (2.0 mL) were heated at 130 °C for 18 h. The crude reaction mixture was purified by flash column

chromatography (3-5% EtOAc in Petrol) to give the title compound as a crystalline white solid (50.4 mg, 96%).

^{1}H NMR (400 MHz, $CDCl_3$) δ 8.29 – 8.23 (m, 3H, Ar-*H*), 8.23 – 8.17 (m, 3H, Ar-*H*), 7.91 (d, *J* = 8.5 Hz, 1H, Ar-*H*), 7.85 (dd, *J* = 8.0, 1.5 Hz, 1H, Ar-*H*), 7.76 (ddd, *J* = 8.5, 7.0, 1.5 Hz, 1H, Ar-*H*), 7.56 (ddd, *J* = 8.0, 7.0, 1.0 Hz, 1H, Ar-*H*), 3.96 (s, 3H, CO_2Me); **^{13}C NMR** (101 MHz, $CDCl_3$) δ 167.1, 156.2, 148.3, 143.8, 137.2, 130.8, 130.3, 130.1, 129.9, 127.7 (2C), 127.6, 127.0, 119.1, 52.4; **LRMS** *m/z* 264.0 $[M+H]^+$; **HRMS** (ESI^+) found *m/z* 264.1018 $[M+H]^+$, $C_{17}H_{14}O_2N$ requires m/z 264.1019; **IR** v_{max} (neat)/cm^{-1} 2947, 1717, 1606, 1595, 1575, 1514, 1495, 1439, 1312, 1275, 1244, 1211, 1194, 1125, 1107, 1051, 1016, 940, 855, 825, 775; **mp** 148-150 °C (EtOAc); **R_f =** 0.23 (5% EtOAc in Petrol). Data is consistent with literature: S. Y. Lee and C-H. Cheon, *J. Org. Chem.*, 2018, **83**, 13036-13044.[25]

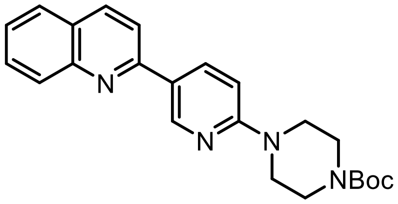

tert-butyl 4-(5-(quinolin-2-yl)pyridin-2-yl)piperazine-1-carboxylate (3ba)

From the diethyl succinate sulfone: Following general procedure **G**, diethyl 2-(quinolin-2-ylsulfonyl)succinate (109.6 mg, 0.30 mmol, 1.5 equiv.), 4-Boc-1-(5-bromo-2-pyridyl)piperazine (68.5 mg, 0.20 mmol, 1.0 equiv.), $Pd(OAc)_2$ (2.2 mg, 0.010 mmol, 5.0 mol%), CataCXium A (7.2 mg, 0.020 mmol, 10 mol%) and K_2CO_3 (55.3 mg, 0.40 mmol, 2.0 equiv.) in toluene (2.0) were heated at 130 °C for 18 h. The crude reaction mixture was purified by flash column chromatography (20% EtOAc in Petrol) to give the title compound as a pale yellow solid (37.8 mg, 48%).

^{1}H NMR (400 MHz, $CDCl_3$) δ 8.94 (d, *J* = 2.5 Hz, 1H, Pyr-*H*), 8.42 (dd, *J* = 9.0, 2.5 Hz, 1H, Pyr-*H*), 8.15 (d, *J* = 8.5 Hz, 1H, Quin-*H*), 8.10 (d, *J* = 8.5 Hz, 1H, Quin-*H*), 7.82 – 7.75 (m, 2H, 2 × Quin-*H*), 7.69 (ddd, *J* = 8.5, 7.0, 1.5 Hz, 1H, Quin-*H*), 7.48 (ddd, *J* = 8.0, 7.0, 1.0 Hz, 1H, Quin-*H*), 6.77 (d, *J* = 9.0 Hz, 1H, Pyr-*H*), 3.68 – 3.55 (m 8H, 2 × $N(CH_2)_2$), 1.49 (s, 9H, $C(CH_3)_3$); **^{13}C NMR** (101 MHz, $CDCl_3$) δ 159.4, 155.2, 154.9, 148.4, 147.5, 136.9, 136.8, 129.8, 129.4, 127.6, 127.0, 126.0, 125.1, 117.9, 106.9, 80.2, 45.1, 28.6 (piperazine carbon environments appear at the same chemical shift); **LRMS** *m/z* 391.2 (60%, $[M+H]^+$), 355.2 (40%, $[M-C_4H_8+H]^+$); **HRMS** (ESI^+) found *m/z* 391.2126 $[M+H]^+$, $C_{23}H_{27}O_2N_4$ requires m/z 391.2129; **IR** v_{max} (neat)/cm^{-1} 2977, 2858, 1677, 1596, 1496, 1430, 1239, 1172, 933, 813, 731; **mp** 128-130 °C (EtOAc); **R_f =** 0.27 (30% EtOAc in Petrol)

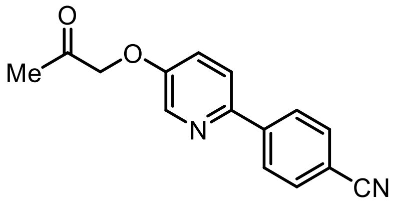

4-(5-(2-oxopropoxy)pyridin-2-yl)benzonitrile (3bb)

From the β-methylester sulfone: Following general procedure **G**, methyl 3-((5-(2-oxopropoxy)pyridin-2-yl)sulfonyl)propanoate (45.2 mg, 0.15 mmol, 1.5 equiv.), 4-bromobenzonitrile (18.2 mg, 0.10 mmol, 1.0 equiv.), $Pd(OAc)_2$ (1.1 mg, 5.0 µmol, 5.0 mol%), CataCXium A (3.6 mg, 0.010 mmol, 10 mol%) and K_2CO_3 (27.6 mg, 0.20 mmol, 2.0 equiv.) in toluene (1.0 mL) were heated at 130 °C for 18 h. The crude reaction mixture was purified by flash column chromatography (35% EtOAc in Petrol) to give the title compound as a white solid (10.5 mg, 42%).

^{1}H NMR (400 MHz, $CDCl_3$) δ 8.42 (d, *J* = 3.0 Hz, 1H, Pyr-*H*), 8.07 – 8.03 (m, 2H, Ar-*H*), 7.75 – 7.70 (m, 3H, Ar-*H* and Pyr-*H*), 7.29 – 7.25 (dd, *J* = 8.5, 3.0 Hz, 1H, Pyr-*H*), 4.68 (s, 2H, C*H$_2$*O), 2.32 (s, 3H, CO*Me*); **^{13}C NMR** (101 MHz, $CDCl_3$) δ 203.7, 154.1, 148.8, 143.0, 138.3, 132.7, 127.0, 122.1, 121.5, 119.0, 112.0, 73.2, 26.7; **LRMS** *m/z* 253.0 $[M+H]^+$; **HRMS** (ESI^+) found *m/z* 253.0972 $[M+H]^+$, $C_{15}H_{13}O_2N_2$ requires m/z 253.0972; **IR** v_{max} (neat)/cm^{-1} 2226, 1734, 1577, 1485, 1470, 1279, 1245, 1222, 1175, 1075, 823; **mp** 92-94 °C (EtOAc); **R_f =** 0.27 (50% EtOAc in Petrol).

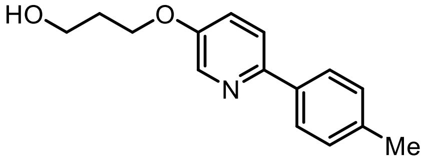

3-((6-(p-tolyl)pyridin-3-yl)oxy)propan-1-ol (3bc)

From the β-methylester sulfone: Following general procedure **G**, methyl 3-((5-(3-hydroxypropoxy)pyridin-2-yl)sulfonyl)propanoate (91.0 mg, 0.30 mmol, 1.5 equiv.), 4-bromotoluene (34.0 mg, 0.20 mmol, 1.0 equiv.), $Pd(OAc)_2$ (2.2 mg, 0.010 mmol, 5.0 mol%), CataCXium A (7.2 mg, 0.020 mmol, 10 mol%) and K_2CO_3 (96.7 mg, 0.70 mmol, 3.5 equiv.) in toluene: DMSO (1:1) (2.0 mL) were heated at 130 °C for 18 h. The crude reaction mixture was purified by flash column chromatography (40% EtOAc in n-pentane) to give the title compound as a white solid (29.4 mg, 60%).

^{1}H NMR (400 MHz, $CDCl_3$) δ 8.39 (d, *J* = 3.0 Hz, 1H, Pyr-*H*), 7.83 (d, *J* = 8.5 Hz, 2H, Ar-*H*), 7.66 (d, *J* = 9.0 Hz, 1H, Ar-*H*), 7.33 (dd, *J* = 9.0, 3.0 Hz, 1H, Pyr-*H*), 7.29 – 7.26 (m, 2H, Ar-*H*), 4.22 (t, *J* = 6.0 Hz, 2H, C*H$_2$*O), 3.88 (t, *J* = 6.0 Hz, 2H, HOC*H$_2$*), 2.44 (br. s, 1H, O*H*), 2.39 (s, 3H, Ar-*Me*), 2.08 (p, *J* = 6.0 Hz, 2H, HOCH$_2$C*H$_2$*); **^{13}C NMR** (101 MHz, $CDCl_3$) δ 154.1, 150.3, 138.3, 137.3, 136.3, 129.6, 126.4, 122.2, 120.8, 65.9, 59.7, 32.1, 21.3; **LRMS** (ESI^+) *m/z* 244.2 $[M+H]^+$; **HRMS** (ESI^+) found *m/z* 244.1337 $[M+H]^+$, $C_{15}H_{18}O_2N$ requires m/z 244.1332; **IR** v_{max} (neat)/cm^{-1} 3356,

3026, 2926, 2878, 2360, 2340, 1469, 1276, 1220, 1059, 816; **mp** 72-74 °C (EtOAc); **R$_f$** = 0.24 (60% EtOAc in Petrol).

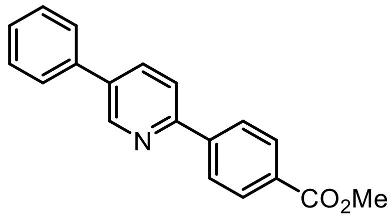

methyl 4-(5-phenylpyridin-2-yl)benzoate (11)

From the β-nitrile sulfone: Following general procedure **F**, 3-((5-phenylpyridin-2-yl)sulfonyl)propanenitrile (59.9 mg, 0.22 mmol, 1.1 equiv.), methyl 4-bromobenzoate (43.0 mg, 0.20 mmol, 1.0 equiv.), $Pd(OAc)_2$ (2.2 mg, 0.010 mmol, 5.0 mol%), CataCXium A (7.2 mg, 0.020 mmol, 10 mol%), K_2CO_3 (41.5 mg, 0.30 mmol, 1.5 equiv.) and AcOH (11.4 µL, 0.20 mmol, 1.0 equiv.) in toluene (2.0 mL) were heated at 120 °C for 18 h. The crude reaction mixture was purified by flash column chromatography (0-5% EtOAc in CH_2Cl_2) to give the title compound as a white crystalline solid (42.7 mg, 74%).

From the β-methylester sulfone: Following general procedure **G**, methyl 3-((5-phenylpyridin-2-yl)sulfonyl)propanoate (84.1 mg, 0.30 mmol, 1.5 equiv.), methyl 4-bromobenzoate (43.0 mg, 0.20 mmol, 1.0 equiv.), $Pd(OAc)_2$ (2.2 mg, 0.010 mmol, 5.0 mol%), CataCXium A (7.2 mg, 0.020 mmol, 10 mol%) and K_2CO_3 (55.3 mg, 0.40 mmol, 2.0 equiv.) in toluene (2 mL) were heated at 120 °C for 18 h. The crude reaction mixture was purified by flash column chromatography (0-5% EtOAc in CH_2Cl_2) to give the title compound as a white crystalline solid (55.2 mg, 95%).

^{1}H NMR (400 MHz, $CDCl_3$) δ 8.97 (d, *J* = 2.5, 1.0 Hz, 1H, Pyr*H*), 8.19 – 8.11 (m, 4H, Ar*H*), 8.00 (dd, *J* = 8.0, 2.5 Hz, 1H, Pyr*H*), 7.88 (dd, *J* = 8.0, 1.0 Hz, 1H, Pyr*H*), 7.67 – 7.63 (m, 2H, Ar*H*), 7.54 – 7.48 (m, 2H, Ar*H*), 7.46 – 7.41 (m, 1H, Ar*H*), 3.96 (s, 3H, CO_2*Me*); **^{13}C NMR** (101 MHz, $CDCl_3$) δ 167.1, 155.0, 148.5, 143.3, 137.5, 135.9, 135.4, 130.5, 130.3, 129.3, 128.4, 127.2, 126.9, 121.0, 52.4; **LRMS** (ESI$^+$) *m/z* 290.0 $[M+H]^+$; **HRMS** (ESI$^+$) found *m/z* 290.1175 $[M+H]^+$, $C_{19}H_{16}O_2N$ requires m/z 290.1176; **IR** ν_{max} (neat)/cm^{-1} 2949, 1719, 1607, 1556, 1471, 1438, 1407, 1371, 1312, 1280, 1194, 1181, 1111, 1014, 837, 763, 746, 698; **mp** 189-191 °C (EtOAc); **R$_f$ =** 0.28 (100% CH_2Cl_2).

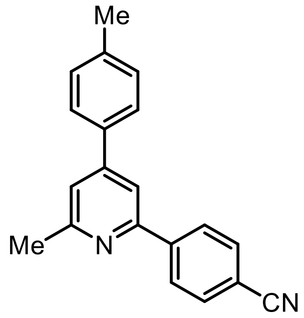

4-(6-methyl-4-(p-tolyl)pyridin-2-yl)benzonitrile (14)

From the β-methylester sulfone: Following general procedure **G**, methyl 3-((6-methyl-4-(p-tolyl)pyridin-2-yl)sulfonyl)propanoate (50.0 mg, 0.15 mmol, 1.5 equiv.), 4-bromobenzonitrile (18.2 mg, 0.10 mmol, 1.0 equiv.), $Pd(OAc)_2$ (1.1 mg, 5.0 µmol, 5.0 mol%), CataCXium A (3.6 mg, 0.010 mmol, 10 mol%) and K_2CO_3 (27.6 mg, 0.20 mmol, 2.0 equiv.) in toluene (1.0 mL) were heated at 120 °C for 18 h. The crude reaction mixture was purified by flash column chromatography (3-5% EtOAc in Petrol) to give the title compound as a white solid (26.1 mg, 92%).

^{1}H NMR (400 MHz, $CDCl_3$) δ 8.16 (d, *J* = 8.5 Hz, 2H, Ar-*H*), 7.77 (d, *J* = 8.5 Hz, 2H, Ar-*H*), 7.74 (d, *J* = 1.5 Hz, 1H, Pyr-*H*), 7.58 (d, *J* = 8.0 Hz, 2H, Ar-*H*), 7.38 (d, *J* = 1.5 Hz, 1H, Pyr-*H*), 7.30 (d, *J* = 8.5 Hz, 2H, Ar-*H*), 2.69 (s, 3H, Pyr-*Me*), 2.43 (s, 3H, Ar-*Me*); **^{13}C NMR** (101 MHz, $CDCl_3$) δ 159.4, 155.3, 149.9, 144.1, 139.5, 135.4, 132.6, 130.0, 127.8, 127.0, 120.9, 119.1, 116.4, 112.3, 24.9, 21.4; **LRMS** (ESI$^+$) *m/z* 285.0 $[M+H]^+$; **HRMS** (ESI$^+$) found *m/z* 285.1383 $[M+H]^+$, $C_{20}H_{17}N_2$ requires m/z 285.1386; **IR** v_{max} (neat)/ cm^{-1} 2923, 2362, 2226, 1734, 1601, 1548, 1507, 1457, 1389, 1114, 1019, 847, 815, 748, 626; **mp** 137-139 °C (EtOAc); **R_f** = 0.24 (5% EtOAc in Petrol).

3.4 Substrate limitations of the desulfinative cross-coupling reaction

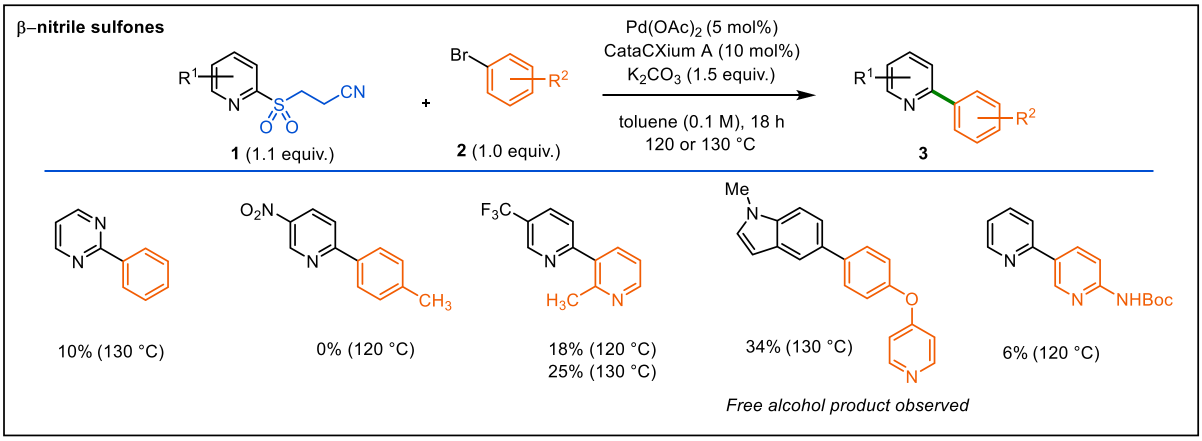

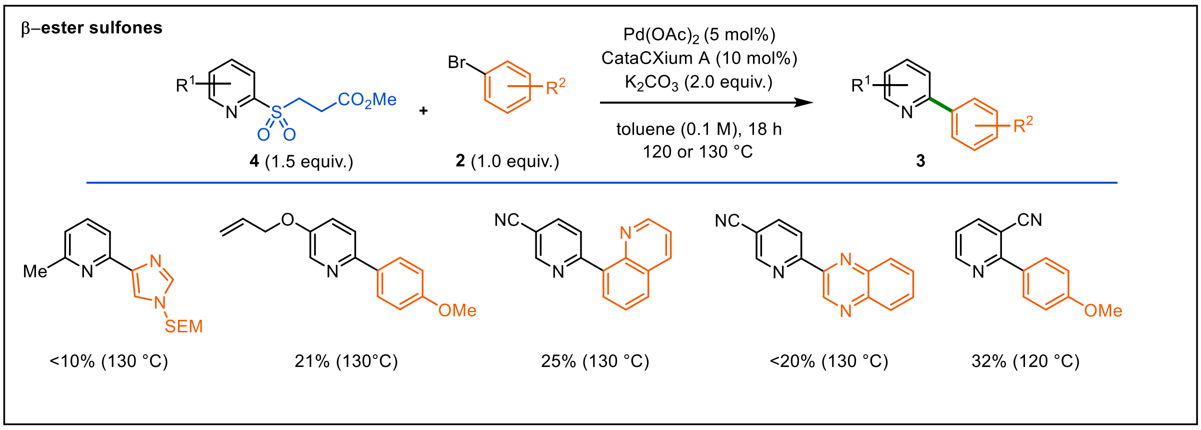

Table 7. Substrates that gave low yields of desired biaryl or observed degradation.

3.5 Scaled up desulfinative cross-coupling: Outside of a sealed reaction vessel

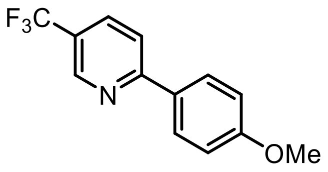

2-(4-methoxyphenyl)-5-(trifluoromethyl)pyridine (3a)

See above for characterisation data

From the β-nitrile sulfone:

On a 0.6 mmol scale:

To an oven-dried two-neck round-bottom flask (25 mL) was added 3-((5-(trifluoromethyl)pyridin-2-yl)sulfonyl)propanenitrile (237.8 mg, 0.90 mmol, 1.5 equiv.), $Pd(OAc)_2$ (6.7 mg, 0.030 mmol, 5.0 mol%), CataCXium A (21.5 mg, 0.060 mmol, 10 mol%) and K_2CO_3 (165.8 mg, 1.20 mmol, 2.0 equiv.). The flask was fitted with a condenser, sealed with a Suba-seal® and purged with N_2 for 30 mins. Degassed, dry anisole (6.0 mL, 0.1 M) was then added, followed by the addition of 4-bromoanisole (75.3 µL, 0.60 mmol, 1.0 equiv.) and AcOH (51.5 µL, 0.84 mmol, 1.5 equiv.) with stirring. The reaction mixture was then heated to 130 °C for 18 h. The reaction mixture was then allowed to cool to RT and concentrated *in vacuo*. The resultant crude residue was adsorbed onto celite and purified by flash column chromatography (3% EtOAc in Petrol) to give the title compound as a white solid (134.0 mg, 88%).

On a 1.0 mmol scale:

To an oven-dried two-neck round-bottom flask (50 mL) was added 3-((5-(trifluoromethyl)pyridin-2-yl)sulfonyl)propanenitrile (396.3 mg, 1.5 mmol, 1.5 equiv.), $Pd(OAc)_2$ (11.2 mg, 0.050 mmol, 5.0 mol%), CataCXium A (35.8 mg, 0.10 mmol, 10 mol%) and K_2CO_3 (276.4 mg, 2.0 mmol, 2.0 equiv.). The flask was fitted with a condenser, sealed with a Suba-seal® and purged with N_2 for 30 mins. Degassed, dry anisole (10 mL, 0.1 M) was then added, followed by the addition of 4-bromoanisole (125.5 µL, 0.60 mmol, 1.0 equiv.) and AcOH (85.9 µL, 1.5 mmol, 1.5 equiv.) with stirring. The reaction mixture was then heated to 130 °C for 18 h. The reaction mixture was then allowed to cool to RT and concentrated *in vacuo*. The resultant crude residue was adsorbed onto celite and purified by flash column chromatography (3% EtOAc in Petrol) to give the title compound as a white solid (245.8 mg, 97%).

From the β-methylester sulfone:

On a 1.0 mmol scale:

To an oven-dried two-neck round-bottom flask (50 mL) was added methyl 3-((5-(trifluoromethyl)pyridin-2-yl)sulfonyl)propanoate (445.9 mg, 0.90 mmol, 1.5 equiv.), $Pd(OAc)_2$ (11.2 mg, 0.050 mmol, 5.0 mol%), CataCXium A (35.8 mg, 0.10 mmol, 10 mol%) and K_2CO_3 (276.4 mg, 2.0 mmol, 2.0 equiv.). The flask was fitted with a condenser, sealed with a

Suba-seal® and purged with N_2 for 30 mins. Degassed, dry anisole (10 mL, 0.1 M) and 4-bromoanisole (125.5 µL, 0.60 mmol, 1.0 equiv.) were added with stirring. The reaction mixture was then heated to 130 °C for 18 h. The reaction mixture was then allowed to cool to RT and concentrated *in vacuo*. The resultant crude residue was adsorbed onto celite and purified by flash column chromatography (3% EtOAc in Petrol) to give the title compound as a white solid (221.8 mg, 88%).

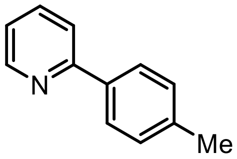

2-(p-tolyl)pyridine (3ac)

See above for characterisation data

From the β-nitrile sulfone:

To an oven-dried two-neck round-bottom flask (50 mL) was added 3-(pyridin-2-ylsulfonyl)propanenitrile (294.3 mg, 1.5 mmol, 1.5 equiv.), 4-bromotoluene (171.0 mg, 1.0 mmol, 1.0 equiv.), $Pd(OAc)_2$ (11.2 mg, 0.050 mmol, 5.0 mol%), CataCXium A (35.8 mg, 0.10 mmol, 10 mol%) and K_2CO_3 (276.4 mg, 2.0 mmol, 2.0 equiv.). The flask was fitted with a condenser, sealed with a Suba-seal® and purged with N_2 for 30 mins. Degassed, dry anisole (10 mL, 0.1 M) was then added, followed by the addition of AcOH (85.9 µL, 1.5 mmol, 1.5 equiv.) with stirring. The reaction mixture was then heated to 130 °C for 18 h. The reaction mixture was then allowed to cool to RT and concentrated *in vacuo*. The resultant crude residue was adsorbed onto celite and purified by flash column chromatography (5% EtOAc in Petrol) to give the title compound as a colourless oil (123.7 mg, 73%).

From the diethyl 2-(pyridin-2-ylsulfonyl)succinate:

To an oven-dried two-neck round-bottom flask (50 mL) was added diethyl 2-(pyridin-2-ylsulfonyl)succinate (473.0 mg, 1.5 mmol, 1.5 equiv.), 4-bromotoluene (171.0 mg, 1.0 mmol, 1.0 equiv.), $Pd(OAc)_2$ (11.2 mg, 0.050 mmol, 5.0 mol%), CataCXium A (35.8 mg, 0.10 mmol, 10 mol%) and K_2CO_3 (276.4 mg, 2.0 mmol, 2.0 equiv.). The flask was fitted with a condenser, sealed with a Suba-seal® and purged with N_2 for 30 mins. Degassed, dry anisole (10 mL, 0.1 M) was then added with stirring. The reaction mixture was then heated to 130 °C for 18 h. The reaction mixture was then allowed to cool to RT and concentrated *in vacuo*. The resultant crude residue was adsorbed onto celite and purified by flash column chromatography (6% EtOAc in Petrol) to give the title compound as a white solid (88.0 mg, 52%).

3.6 Derivatisations of the pyridine core of the masked sulfinate reagents

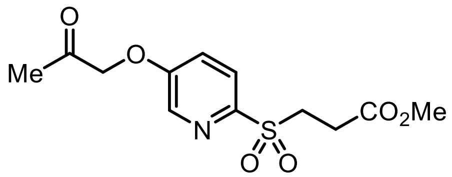

methyl 3-((5-(2-oxopropoxy)pyridin-2-yl)sulfonyl)propanoate (7)

Following a modified Wacker-like oxidation procedure reported by D. A. Chaudhari and R. A. Fernandes:[26]

An oven-dried round bottom flask containing $Pd(OAc)_2$ (4.4 mg, 0.020 mmol, 10 mol%) and Dess-Martin periodinane (424.1 mg, 1.0 mmol, 5.0 equiv.) was purged with N_2 for 10 mins. Methyl 3-((5-(allyloxy)pyridin-2-yl)sulfonyl)propanoate (57.1 mg, 0.2 mmol, 1.0 equiv.) in MeCN (4.5 mL, 0.04 M) was added to the reaction vessel, followed by the addition of water (0.750 mL). The reaction was then heated to 50 °C for 18 h. On cooling to room temperature the reaction mixture was filtered through a pad of silica and washed with EtOAc. The filtrate was concentrated *in vacuo*, adsorbed onto celite and purified by flash column chromatography (60% EtOAc in Petrol) to give the title product as a clear oil that crystallised to a white solid on standing (26.4 mg, 44%).

^{1}H NMR (400 MHz, $CDCl_3$) δ 8.40 (dd, *J* = 3.0, 0.5 Hz, 1H, Pyr-*H*), 8.03 (dd, *J* = 8.5, 0.5 Hz, 1H, Pyr-*H*), 7.29 (dd, *J* = 8.5, 3.0 Hz, 1H, Pyr-*H*), 4.73 (s, 2H, CH_2OPyr), 3.70 – 3.64 (m, 5H, CO_2Me and SO_2CH_2), 2.85 – 2.80 (t, *J* = 8.0 Hz, 2H, CH_2CO_2Me), 2.32 (s, 3H, *Me*CO); **^{13}C NMR** (101 MHz, $CDCl_3$) δ 202.1, 170.7, 156.9, 149.2, 139.3, 124.2, 121.5, 72.9, 52.5, 48.2, 27.6, 26.6; **LRMS** (ESI^+) *m/z* 324.0 $[M+Na]^+$; **HRMS** (ESI^+) found *m/z* 302.0691 $[M+H]^+$, $C_{12}H_{16}O_6NS$ requires m/z 302.0693; **IR** ν_{max} (neat)/cm^{-1} 2955, 2361, 1733, 1573, 1456, 1438, 1396, 1365, 1312, 1246, 1161, 1134, 1100, 1059, 1013, 977, 836, 754, 722; **mp** 62-64 °C (EtOAc); **R_f =** 0.23 (80% EtOAc in Petrol).

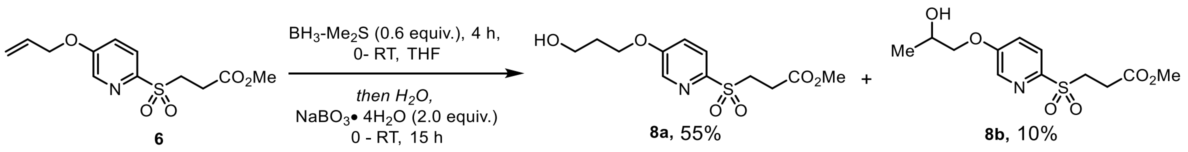

methyl 3-((5-(3-hydroxypropoxy)pyridin-2-yl)sulfonyl)propanoate (8a) and methyl 3-((5-(2-hydroxypropoxy)pyridin-2-yl)sulfonyl)propanoate (8b)

Methyl 3-((5-(allyloxy)pyridin-2-yl)sulfonyl)propanoate (142.6 mg, 0.50 mmol, 1.00 equiv.) was dissolved in dry, degassed THF (2.5 mL, 0.2 M) under an argon atmosphere and the solution was cooled to 0 °C. Borane dimethyl sulfide complex solution (30.0 µL, 0.32 mmol, 0.630 equiv.) was added dropwise to the stirring solution, after which the reaction mixture was allowed to warm to RT and stirred for 3.5 h. The reaction mixture was then cooled to 0 °C again and H_2O (2.0 mL) was added dropwise to the solution (gas release observed). Sodium perborate tetrahydrate (307.7 mg, 2.0 mmol, 4.0 equiv.) was then added in one portion, the reaction was allowed to warm to RT and stirred for 15 h. The reaction mixture was then diluted with EtOAc and the aqueous layer extracted with EtOAc (3 × 20 mL). The organic layers were combined, washed with brine, dried over $MgSO_4$, filtered and concentrated *in vacuo*. The crude product was then purified by flash column chromatography on silica gel (80% EtOAc in Petrol to 100% EtOAc) to give the anti-markovnikov product **8a** as a colourless oil (83.7 mg, 55%) and the markovnikov side product **8b** (14.9 mg, 10%).

Anti-markovnikov product (8a): ^{1}H NMR (400 MHz, $CDCl_3$) δ 8.34 (dd, *J* = 3.0, 0.5 Hz, 1H, Pyr-*H*), 7.96 (dd, *J* = 9.0, 0.5 Hz, 1H, Pyr-*H*), 7.34 (dd, *J* = 9.0, 3.0 Hz, 1H, Pyr-*H*), 4.22 (t, *J* = 6.0 Hz, 2H, CH_2O), 3.82 (t, *J* = 6.0 Hz, 2H, $HOCH_2$), 3.65 (s, 3H, CO_2Me), 3.61 (t, *J* = 8.5 Hz, 2H, SO_2CH_2), 2.76 (t, *J* = 8.5 Hz, 2H, CH_2CO_2Me), 2.06 (p, *J* = 6.0 Hz, 2H, $CH_2CH_2CH_2$); **^{13}C NMR** (101 MHz, $CDCl_3$) δ 170.7, 158.2, 147.8, 139.5, 124.3, 120.9, 66.1, 58.9, 52.4, 48.2, 31.7, 27.6; **LRMS** (ESI^+) m/z 326.0 $[M+Na]^+$; **HRMS** (ESI^+) found *m/z* 304.0849 $[M+H]^+$, $C_{12}H_{18}O_6NS$ requires m/z 304.0849; **IR** ν_{max} (neat)/cm^{-1} 3519, 2953, 2361, 1736, 1572, 1453, 1366, 1309, 1278, 1250, 1201, 1160, 1131, 1098, 1054, 1013, 981, 949, 838, 753, 722, 670, 625; **R_f** = 0.23 (80% EtOAc in Petrol).

Markovnikov side- product (8b): ^{1}H NMR (400 MHz, $CDCl_3$) δ 8.41 (d, *J* = 3.0 Hz, 1H, Pyr-*H*), 8.03 (d, *J* = 8.5 Hz, 1H, Pyr-*H*), 7.37 (dd, *J* = 8.5, 3.0 Hz, 1H, Pyr-*H*), 4.26 (ddp, *J* = 13.0, 6.5, 3.5 Hz, 1H, MeC*H*OH), 4.06 (dd, *J* = 9.5, 3.5 Hz, 1H, $CHCH_aH_b$), 3.96 (dd, *J* = 9.5, 7.5 Hz, 1H, $CHCH_aH_b$), 3.70 – 3.63 (m, 5H, CO_2*Me* and SO_2CH_2), 2.81 (t, *J* = 8.0 Hz, 2H, CH_2CO_2Me), 1.34 (d, *J* = 6.5 Hz, 3H, *Me*COH); **^{13}C NMR** (101 MHz, $CDCl_3$) δ 170.7, 157.8, 148.6, 139.4, 124.2, 121.2, 74.2, 66.1, 52.5, 48.3, 27.6, 19.1; **LRMS** (ESI^+) m/z 326.0 $[M+Na]^+$; **HRMS** (ESI^+) found *m/z* 304.0849 $[M+H]^+$, $C_{12}H_{18}O_6NS$ requires m/z 304.0849; **IR** ν_{max} (neat)/cm^{-1} 3501, 2953, 2360, 2341, 1738, 1573, 1450, 1368, 1315, 1280, 1252, 1201, 1162, 1133, 1099, 1062, 1021, 985, 934, 840, 800, 755, 724, 668, 625; **R_f** = 0.25 (80% EtOAc in Petrol).

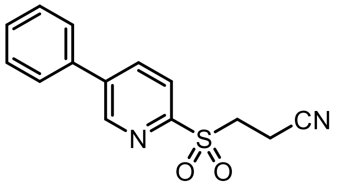

3-((5-phenylpyridin-2-yl)sulfonyl)propanenitrile (10a)

Following a modified Suzuki-Miyaura procedure reported by D. Hédou and A. S. Voisin-Chiret:[27]

3-((5-Bromopyridin-2-yl)sulfonyl)propanenitrile (137.6 mg, 0.5 mmol, 1.0 equiv.), phenylboronic acid (91.5 mg, 0.75 mmol, 1.5 equiv.), tris(dibenzylideneacetone)dipalladium(0) (22.9 mg, 0.025 mmol, 5.0 mol%), tetrabutylammonium bromide (TBAB, 16.1 mg, 0.05 mmol, 10 mol%) and tri(2-furyl)phosphine (tfp, 11.6 mg, 0.05 mmol, 10 mol%) were combined in a sealed tube and the reaction vessel was evacuated and flushed with N_2 three times. To the reaction mixture was then added THF (2.0 mL, 0.25 M) and potassium carbonate (138.2 mg, 1.0 mmol, 2.0 equiv.) as a solution in water (0.5 mL). The reaction was heated to 40 °C for 18 h, before being cooled to RT and quenched with saturated aqueous NH_4Cl solution (5 mL). The product was then extracted with CH_2Cl_2 (2 × 10 mL), the organic layers combined and washed with brine, dried over $MgSO_4$, filtered and concentrated *in vacuo*. The crude product was then purified through flash column chromatography on silica gel (12% EtOAc in toluene) to give the title compound as a white solid (98.3 mg, 72%).

^{1}H NMR (400 MHz, $CDCl_3$) δ 8.96 (t, *J* = 1.5 Hz, 1H, Pyr-*H*), 8.18 – 8.15 (m, 2H, overlapping Pyr-*H*), 7.65 – 7.60 (m, 2H, Ar-*H*), 7.58 – 7.49 (m, 3H, Ar-*H*), 3.76 (t, *J* = 7.5 Hz, 2H, C*H*$_2$CN), 2.99 (t, *J* = 7.5 Hz, 2H, SO_2C*H*$_2$); **^{13}C NMR** (101 MHz, $CDCl_3$) δ 154.7, 149.1, 141.5, 136.6, 135.8, 129.8, 129.7, 127.7, 122.5, 116.2, 47.5, 12.3; **LRMS** (ESI^+) *m/z* 273.0 (30%, $[M+H]^+$), 295.0 (70%, $[M+Na]^+$); **HRMS** (ESI^+) found *m/z* 273.0692 $[M+H]^+$, $C_{14}H_{13}O_2N_2S$ requires m/z 273.0692; **IR** ν_{max} (neat)/ cm^{-1} 3061, 2940, 2252, 1581, 1455, 1419, 1367, 1316, 1166, 1134, 1100, 1028, 1005, 953, 854, 811, 768, 748, 700, 672, 634; **mp** 108-110 °C (EtOAc); **R_f** = 0.23 (10% EtOAc in toluene).

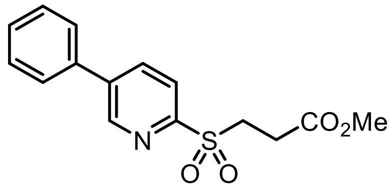

methyl 3-((5-phenylpyridin-2-yl)sulfonyl)propanoate (10b)

Following a modified Suzuki-Miyaura procedure reported by D. Hédou, A. S. Voisin-Chiret:[27]

Methyl 3-((5-bromopyridin-2-yl)sulfonyl)propanoate (154.1 mg, 0.50 mmol, 1.0 equiv.), phenylboronic acid (91.5 mg, 0.75 mmol, 1.5 equiv.), tris(dibenzylideneacetone)dipalladium(0) (22.9 mg, 0.025 mmol, 5.0 mol%), tetrabutylammonium bromide (TBAB, 16.1 mg, 0.05 mmol, 10 mol%) and tri(2-furyl)phosphine (tfp, 11.6 mg, 0.05, 10 mol%) were combined in a sealed tube and the reaction vessel was evacuated and flushed with N_2 three times. To the reaction mixture was then added THF (2.0 mL, 0.25 M) and potassium carbonate (138.2 mg, 1.00 mmol, 2.0 equiv.) as a solution in water (0.5 mL). The reaction was heated to 40 °C for 22 h, before being cooled to RT and diluted with saturated aqueous NH_4Cl solution (5 mL). The product was then extracted with CH_2Cl_2 (2 × 10 mL), the organic layers combined and washed with brine, dried over $MgSO_4$, filtered and concentrated *in vacuo*. The crude product was then purified through flash column chromatography on silica gel (12% EtOAc in toluene) to give the title compound as a white solid (118.8 mg, 78%).

^{1}H NMR (400 MHz, $CDCl_3$) δ 8.95 (dd, *J* = 2.0, 1.0 Hz, 1H, Pyr-*H*), 8.15 (dd, *J* = 8.0, 1.0 Hz, 1H, Pyr-*H*), 8.13 (d, *J* = 2.0 Hz, 1H, Pyr-*H*), 7.63 – 7.60 (m, 2H, Ar-*H*), 7.57 – 7.46 (m, 3H, Ar-*H*), 3.77 (t, *J* = 7.5 Hz, 2H, SO_2CH_2), 3.70 (s, 3H, CO_2Me), 2.88 (t, *J* = 7.5 Hz, 2H, CH_2CO_2Me); **^{13}C NMR** (101 MHz, $CDCl_3$) δ 170.7, 155.3, 148.9, 140.9, 136.3, 136.0, 129.62, 129.59, 127.6, 122.5, 52.5, 48.0, 27.6; **LRMS** (ESI^+) *m/z* 328.0 $[M+Na]^+$; **HRMS** (ESI^+) found *m/z* 306.0794 $[M+H]^+$, $C_{15}H_{16}O_4NS$ requires m/z 306.0795; **IR** v_{max} (neat)/ cm^{-1} 3061, 2952, 1738, 1581, 1455, 1438, 1366, 1316, 1251, 1200, 1162, 1131, 1100, 1005, 853, 834, 768, 749, 699, 664; **mp** 102-104 °C (EtOAc); **R_f** = 0.18 (10% EtOAc in toluene).

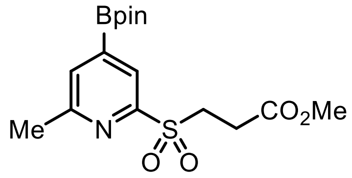

methyl 3-((6-methyl-4-(4,4,5,5-tetramethyl-1,3,2-dioxaborolan-2-yl)pyridin-2-yl)sulfonyl)propanoate (12)

Following a modified Ir-catalysed borylation procedure reported by L. T. Pilarski and coworkers:[28]

Note: The B_2pin_2 was dried overnight under vacuum at 60 °C before use.

To an oven-dried sealed tube equipped with a magnetic stirrer bar was added (1,5-cyclooctadiene)(methoxy)iridium(I) dimer (8.3 mg, 0.0125 mmol, 2.5.0 mol%), B_2pin_2, (127 mg, 0.500 mmol, 1.0 equiv.) and 4,4′-di-tert-butyl-2,2′-dipyridyl (dtbpy, 6.7 mg, 0.0250 mmol, 5.0 mol%). The reaction vessel was evacuated under vacuum and flushed with N_2 (× 5) before the addition of THF (2 mL). The reaction was stirred at RT for 30 mins (solution should go from a dark brown to a deep red colour). Methyl 3-((6-methylpyridin-2-yl)sulfonyl)propanoate (121.6 mg, 0.500 mmol, 1.0 equiv.) in THF (1.0 mL, 0.5 M) was added to the reaction mixture and reaction was heated to 60 °C for 18 h. The reaction was cooled to RT, diluted with Et_2O and concentrated *in vacuo*. The crude product was then purified by triturating with minimal Et_2O and removing the solid impurities by filtering through filter paper (× 4). The residue was then dried overnight under vacuum at 60 °C to give the desired product as a pale orange oil (125.3 mg, 68%).

^{1}H NMR (400 MHz, $CDCl_3$) δ 8.22 (s, 1H, Pyr-*H*), 7.74 (s, 1H, Pyr-*H*), 3.72 – 3.66 (m, 5H, SO_2CH_2 and CO_2Me), 2.83 (t, *J* = 7.5 Hz, 2H, CH_2CO_2Me), 2.64 (s, 3H, Pyr-*Me*), 1.35 (s, 12H, $C(CH_3)_2C(CH_3)_2$); **^{13}C NMR** (101 MHz, $CDCl_3$) δ 170.8, 159.7, 155.7, 133.0, 124.2, 85.2, 52.4, 47.6, 27.5, 25.0, 24.3; **HRMS** (ESI$^+$) found *m/z* 369.1527 $[M+H]^+$, $C_{15}H_{25}O_6NBS$ requires m/z 369.1526; **IR** v_{max} (neat)/ cm^{-1} 2979, 2954, 2361, 2342, 1741, 1593, 1533, 1436, 1393, 1373, 1128, 1060, 1019, 983, 965, 911, 898, 845, 827, 795, 742, 682; **R_f** = 0.16 (Spot drags off baseline - 80% EtOAc in Petrol).

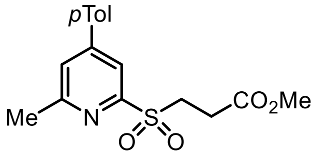

methyl 3-((6-methyl-4-(p-tolyl)pyridin-2-yl)sulfonyl)propanoate (13)

Following a modified Suzuki-Miyaura cross-coupling procedure reported by T. S.-B. Lou and M. C. Willis:[29]

An oven-dried sealed tube containing 4-bromotoluene (34.2 mg, 0.20 mmol, 1.0 equiv.), methyl-3-((6-methyl-4-(4,4,5,5-tetramethyl-1,3,2-dioxaborolan-2-yl)pyridin-2-yl)sulfonyl) propanoate (110.8 mg, 0.30 mmol, 1.5 equiv.), $Pd(OAc)_2$ (2.2 mg, 0.010 mmol, 5.0 mol%) and XPhos (9.5 mg, 0.020 mmol, 10 mol%) was evacuated and backfilled with N_2 (× 5). To the reaction vessel was added K_3PO_4 (84.9 mg, 0.40 mmol, 2.0 equiv.) as solution in water (400 µL) and then 1,4-dioxane (800 µL). The resulting bright orange suspension was heated at 70 °C for 18 h. The reaction mixture was then cooled to RT, diluted with EtOAc and anhydrous $MgSO_4$ was added directly. The reaction was filtered through a pad of celite (washing with EtOAc) and concentrated *in vacuo*. The crude residue was then purified by flash column chromatography (10% EtOAc in toluene) to give the title product as a cloudy colourless oil (41.3 mg, 62%).

^{1}H NMR (400 MHz, $CDCl_3$) δ 8.09 (d, *J* = 1.0 Hz, 1H, Ar-*H*), 7.61 – 7.53 (m, 3H, Ar-*H*), 7.31 (d, *J* = 8.0 Hz, 2H, Ar-*H*), 3.75 (t, *J* = 8.0 Hz, 2H, SO_2CH_2), 3.68 (s, 3H, CO_2Me), 2.87 (t, *J* = 8.0 Hz, 2H, CH_2CO_2Me), 2.68 (s, 3H, Pyr-*Me*), 2.42 (s, 3H, Ar-*Me*); **^{13}C NMR** (101 MHz, $CDCl_3$) δ 170.8, 160.7, 156.8, 151.0, 140.5, 133.7, 130.2, 127.1, 124.7, 117.1, 52.4, 47.6, 27.5, 24.5, 21.4; **LRMS** (ESI$^+$) m/z 356.0 $[M+Na]^+$**;** **HRMS** (ESI$^+$) found *m/z* 334.1102 $[M+H]^+$, $C_{17}H_{20}NO_4S$ requires m/z 334.1108; **IR** ν_{max} (neat)/cm^{-1} 2953, 1740, 1599, 1537, 1518, 1437, 1388, 1313, 1250, 1163, 1120, 1020, 989, 908, 841, 817, 736, 719, 618; **R_f** = 0.25 (40% EtOAc in Petrol).

3.7 Synthesis of miscellaneous building blocks

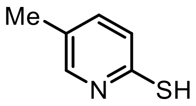

5-methylpyridine-2-thiol

Following a modified procedure by Kanishchev *et.al.*:[30]

Note: Due to the stench of Lawesson's reagent, it should be handled only inside a fumehood.

Under a N_2 atmosphere: 2-hydroxy-5-methylpyridine (1.09 g, 10.0 mmol, 1.7 equiv.) and Lawesson's reagent (6.50 g, 6.06 mmol, 1.0 equiv.) were combined in dry, degassed toluene (55 mL, 0.11 M). The resultant yellow suspension was stirred at reflux for 14 h. The reaction mixture was then transferred to a separating funnel containing aqueous NaOH solution (5.60 g in 60 mL H_2O). The aqueous layer was washed with Et_2O (30 mL). The organic layer was then discarded. The aqueous layer was then acidified with AcOH (45 mL). The aqueous layer was then washed with CH_2Cl_2 (3 × 25 mL). The organic layers were then combined and washed with brine (50 mL), dried over $MgSO_4$ and concentrated *in vacuo* with additional toluene to remove all AcOH to give the crude title product as a bright yellow solid (442 mg, 35%). *The crude reaction product was used directly in the next step.*

^{1}H NMR (400 MHz, $CDCl_3$) δ 13.78 (s, 1H, S*H*), 7.48 (d, *J* = 9.0 Hz, 1H, Pyr-*H*), 7.42 (s, 1H, Pyr-*H*), 7.25 – 7.21 (m, 1H, Pyr-*H*), 2.18 (s, 3H, C*H*$_3$); **LRMS** (ESI$^+$) *m/z* 126.1 $[M+H]^+$. Data is consistent with the literature: O. S. Kanishchev and W. R. Dolbier, *Ang. Chem. Int. Ed.*, 2015, **54**, 280-284.[30]

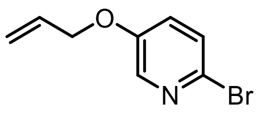

5-(allyloxy)-2-bromopyridine

Following a modified allylation procedure reported J-X. Chen and coworkers:[31]

Under a N_2 atmosphere: To a stirring solution of 6-bromopyridin-3-ol (348.0 mg, 2.0 mmol, 1.0 equiv.) in anhydrous DMF (4 mL, 2.0 M) was added NaH (60% in mineral oil, 88.0 mg, 2.2. mmol, 1.1 equiv.). at 0 °C. The resultant cloudy white suspension was allowed to warm to room temperature and stirred for 1 h. A solution of 3-bromoprop-1-ene (186 µL, 2.2 mmol, 1.1 equiv.) in DMF (1 mL) was added dropwise and the reaction heated to 70 °C for 1 h. The reaction was then cooled to RT and quenched with H_2O (2 mL). The aqueous layer was extracted with EtOAc, and the organic layer washed with brine (x3), dried over Na_2SO_4, filtered and concentrated *in vacuo*. The crude residue was then purified by flash column chromatography (5% EtOAc in Petrol) to give the title product as a colourless oil (359.1 mg, 84%).

^{1}H NMR (400 MHz, $CDCl_3$) δ 8.07 (dd, *J* = 3.0, 0.5 Hz, 1H, Pyr-*H*), 7.36 (dd, *J* = 8.5, 0.5 Hz, 1H, Pyr-*H*), 7.10 (dd, *J* = 8.5, 3.0 Hz, 1H, Pyr-*H*), 6.01 (ddt, *J* = 17.5, 10.5, 5.5 Hz, 1H, CH^aH^b=C*H*), 5.41 (dq, *J* = 17.0, 1.5 Hz, 1H, CH^aH^b=CH), 5.33 (dq, *J* = 10.5, 1.5 Hz, 1H, CH^aH^b=CH), 4.56 (dt, *J* = 5.5, 1.5 Hz, 2H, OC*H*$_2$); **^{13}C NMR** (101 MHz, $CDCl_3$) δ 154.6, 137.9, 132.4, 132.2, 128.3, 125.3, 118.8, 69.6; **LRMS** (ESI$^+$) m/z 214.0 (50%, $[M+H]^+$), 216.0 (50%, $[M+H]^+$); **HRMS** (ESI$^+$) found *m/z* 213.9864 $[M(^{79}Br)+H]^+$, $C_8H_9ON^{79}Br$ requires m/z 213.9862; **IR** ν_{max} (neat)/ cm^{-1} 3084, 2867, 1650, 1577, 1564, 1449, 1424, 1367, 1269, 1223, 1120, 1089, 1011, 991, 928, 822, 721, 676, 622; **R$_f$** = 0.40 (10% EtOAc in Petrol).

4. NMR Spectra

4.1 NMR Spectra of heteroaromatic sulfide intermediates

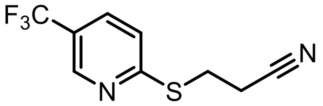

3-((5-(trifluoromethyl)pyridin-2-yl)thio)propanenitrile (*pre*-1a)

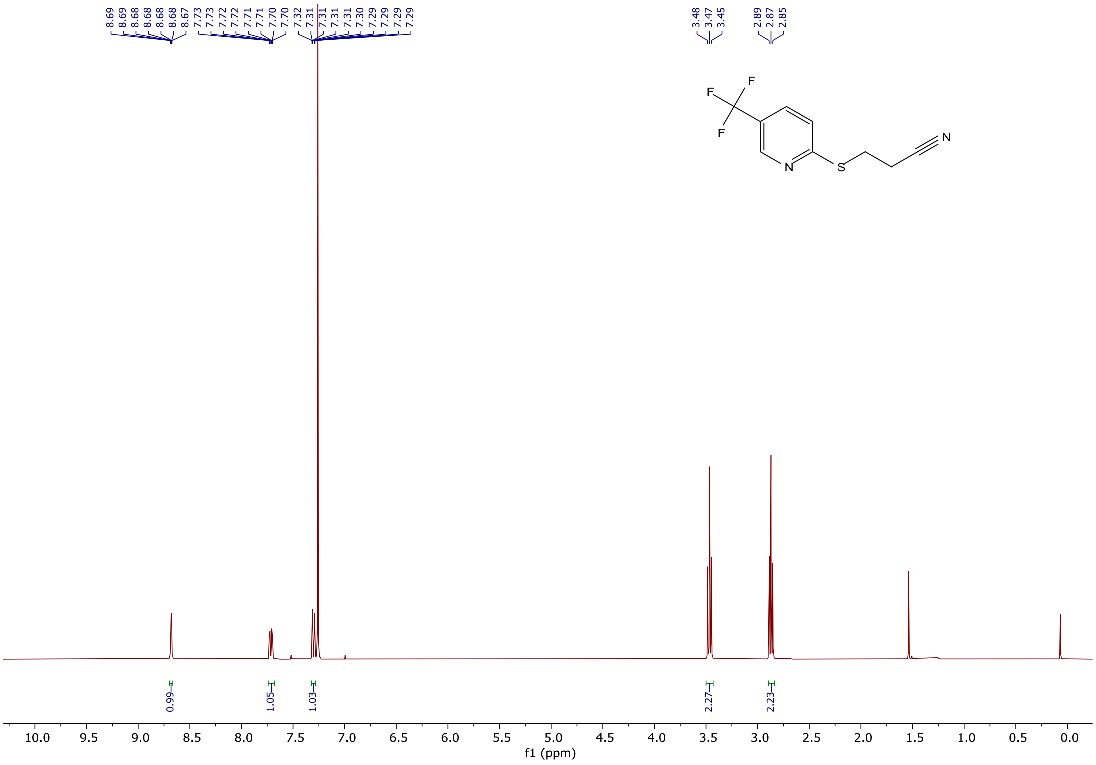

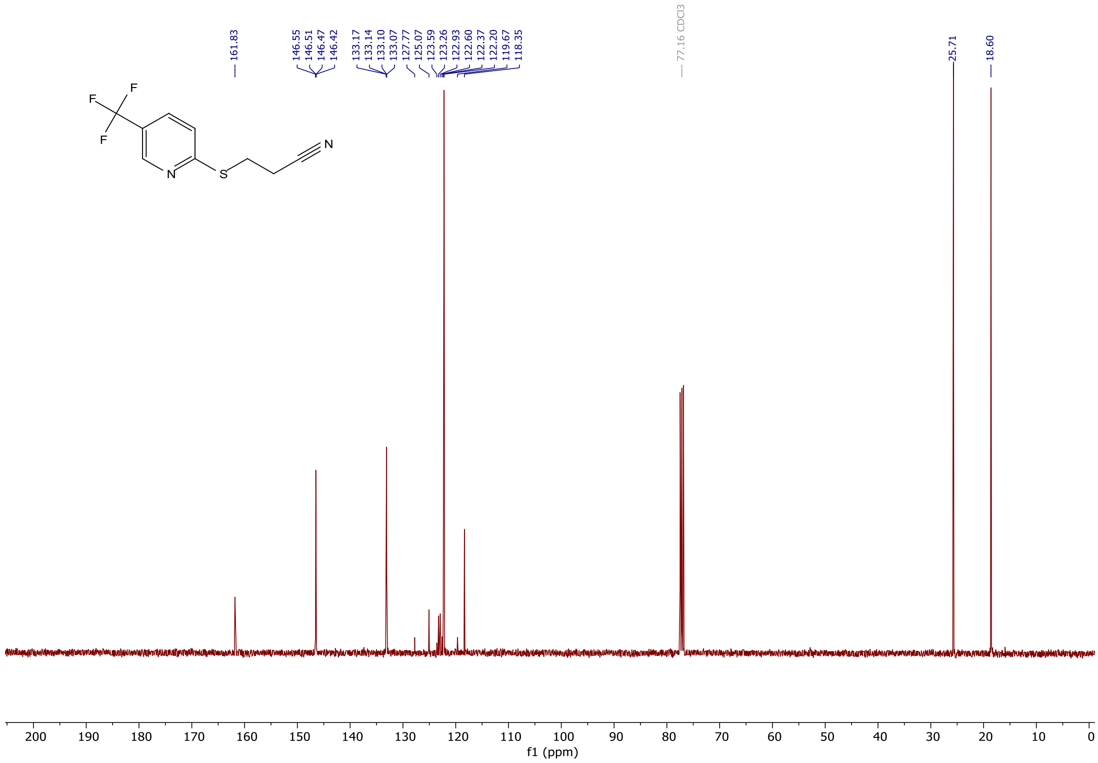
161.83
146.55
146.51
146.47
146.42
133.17
133.14
133.10
133.07
127.77
125.07
123.59
123.26
122.93
122.60
122.37
122.20
119.67
118.35
77.16 CDCl3
25.71
18.60
f1 (ppm)

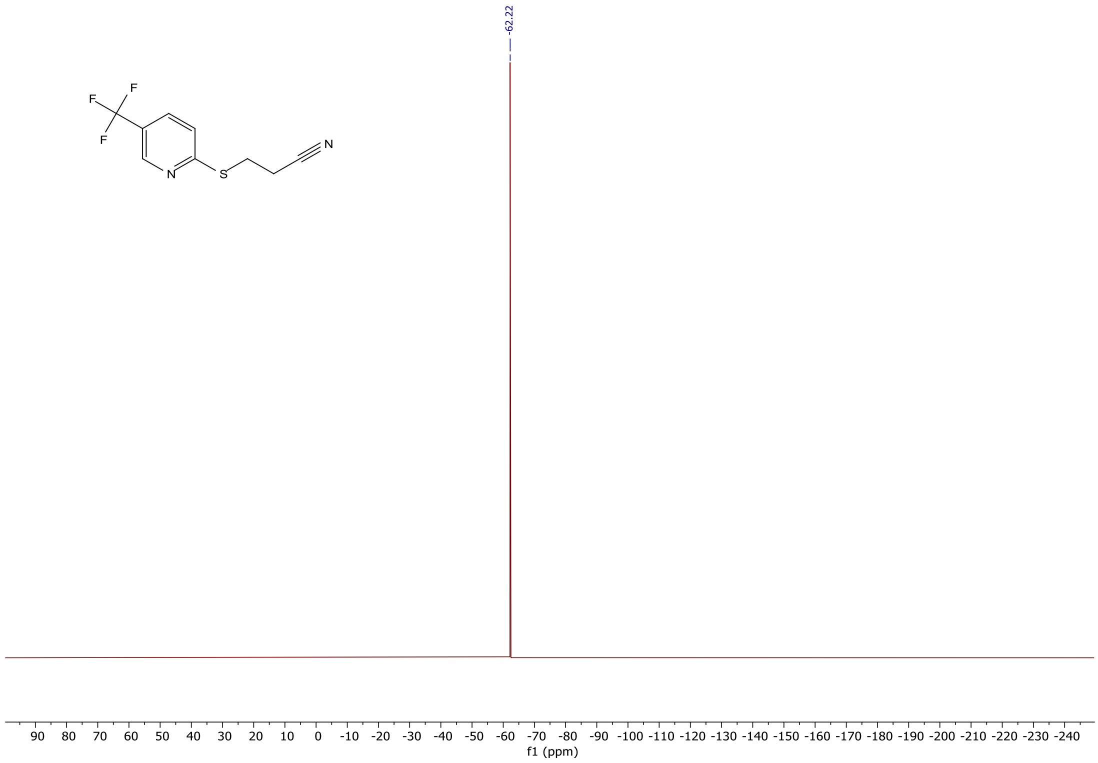
-62.22
f1 (ppm)

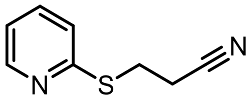

3-(pyridin-2-ylthio)propanenitrile (*pre*-1b)

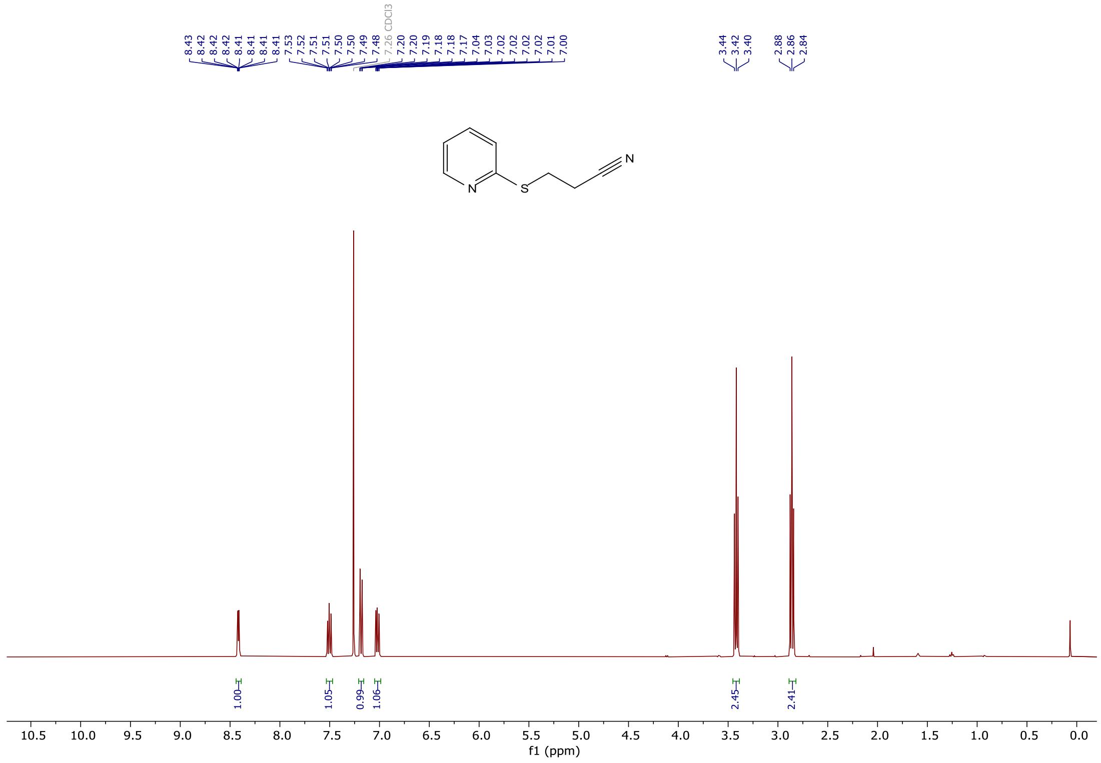

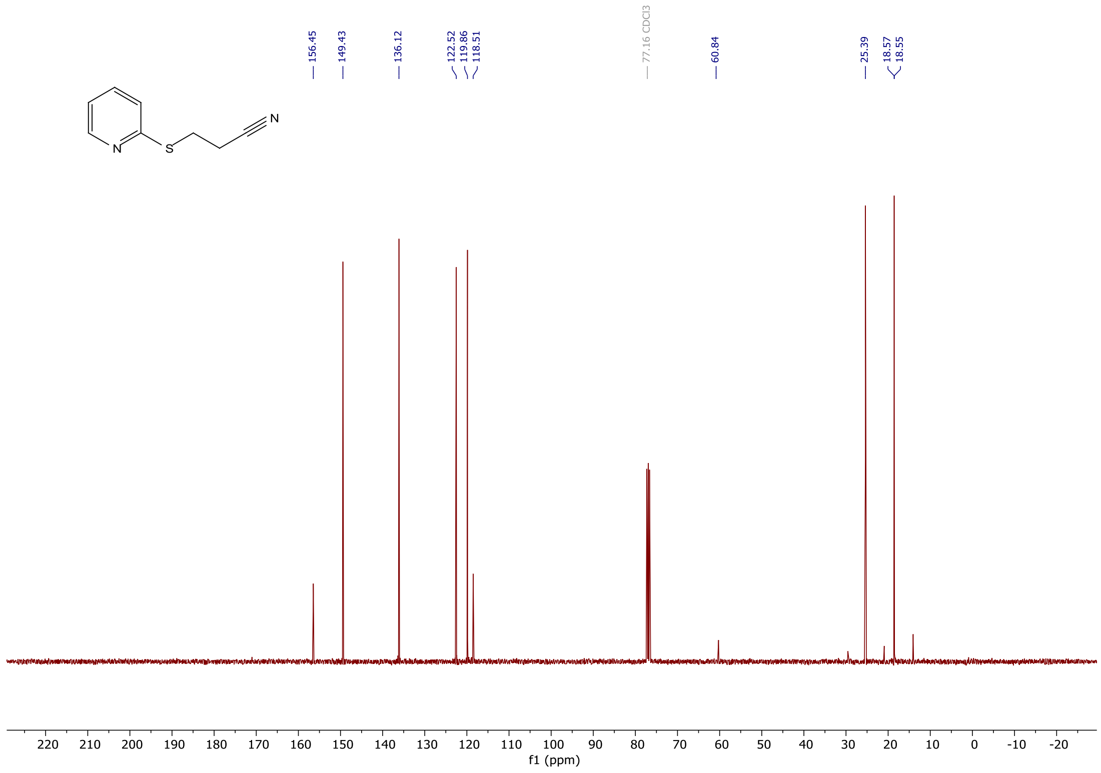

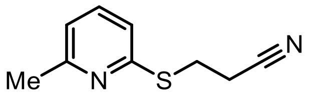

3-((6-methylpyridin-2-yl)thio)propanenitrile (*pre*-1c)

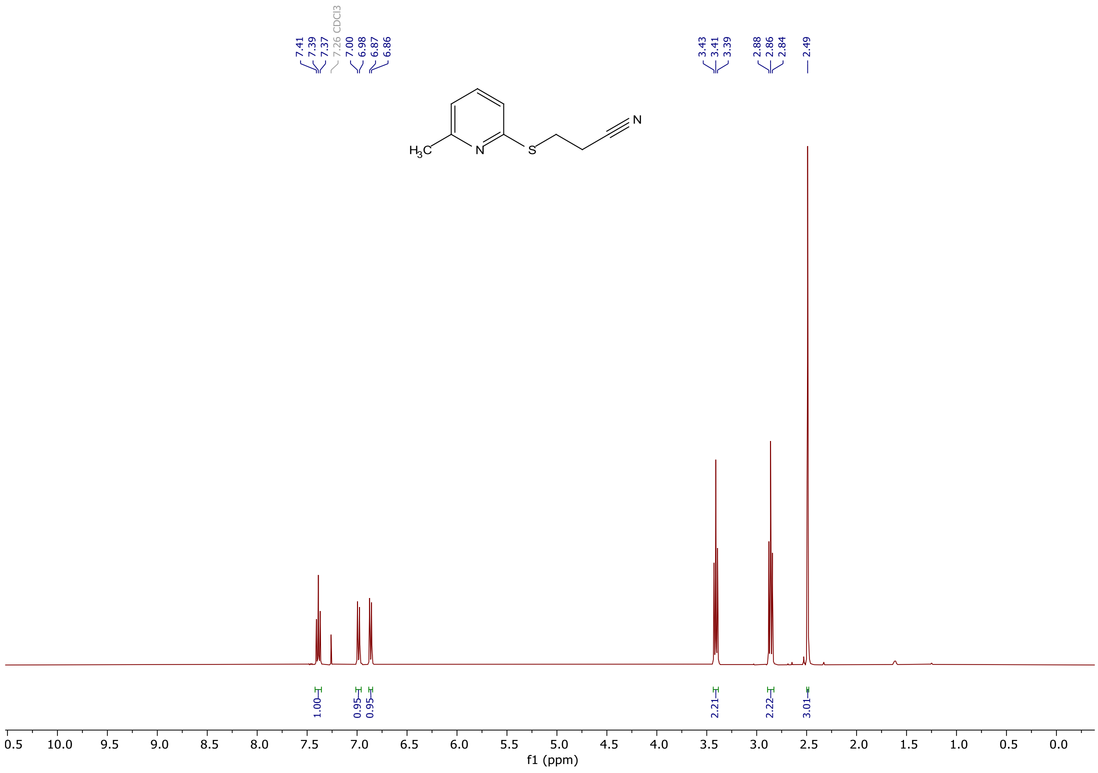

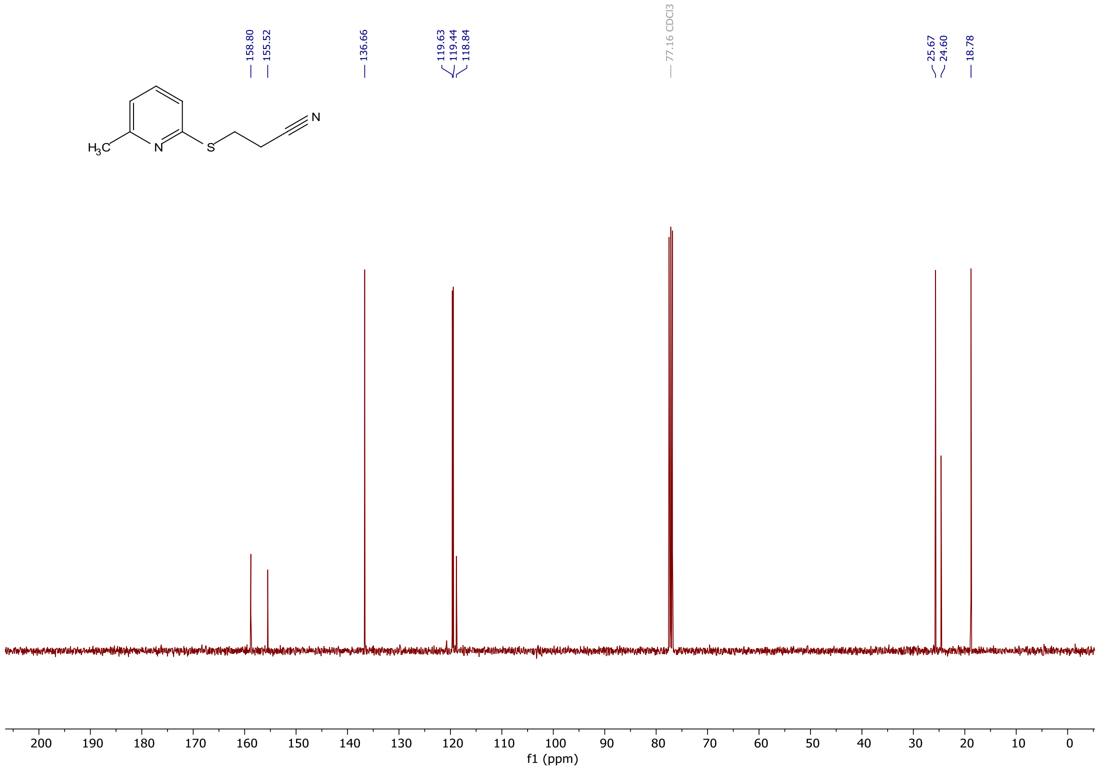

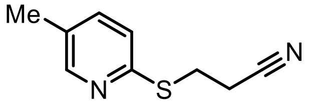

3-((5-methylpyridin-2-yl)thio)propanenitrile (*pre*-1d)

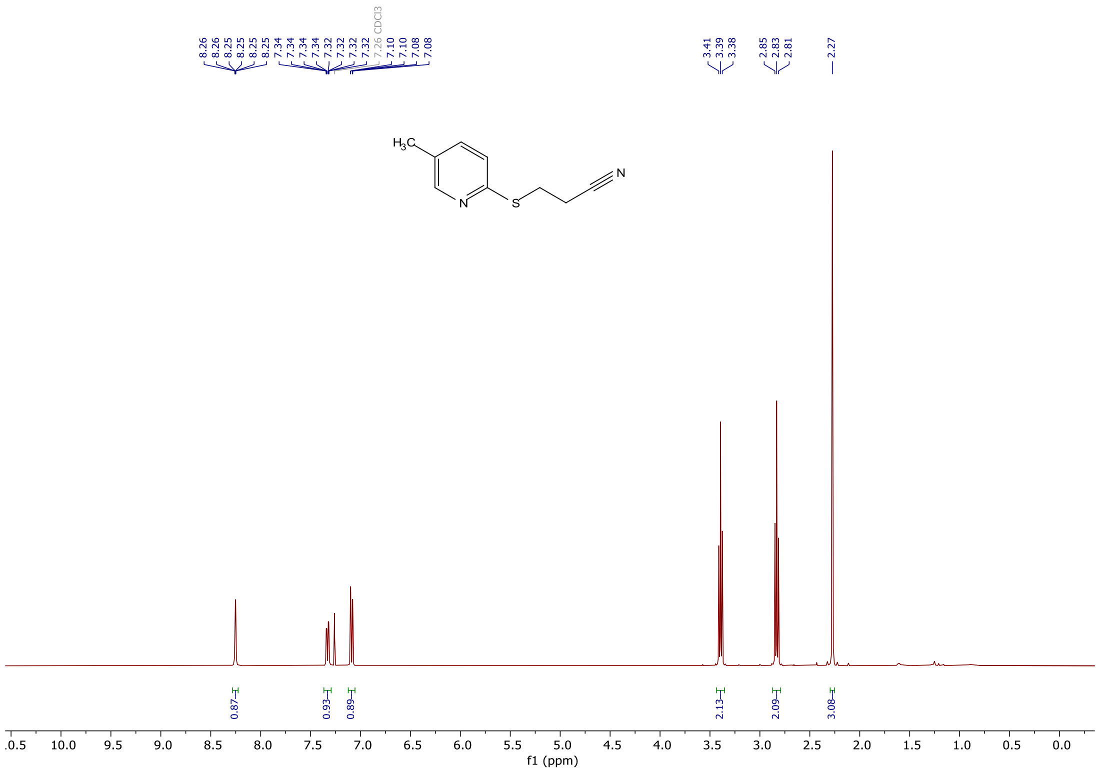

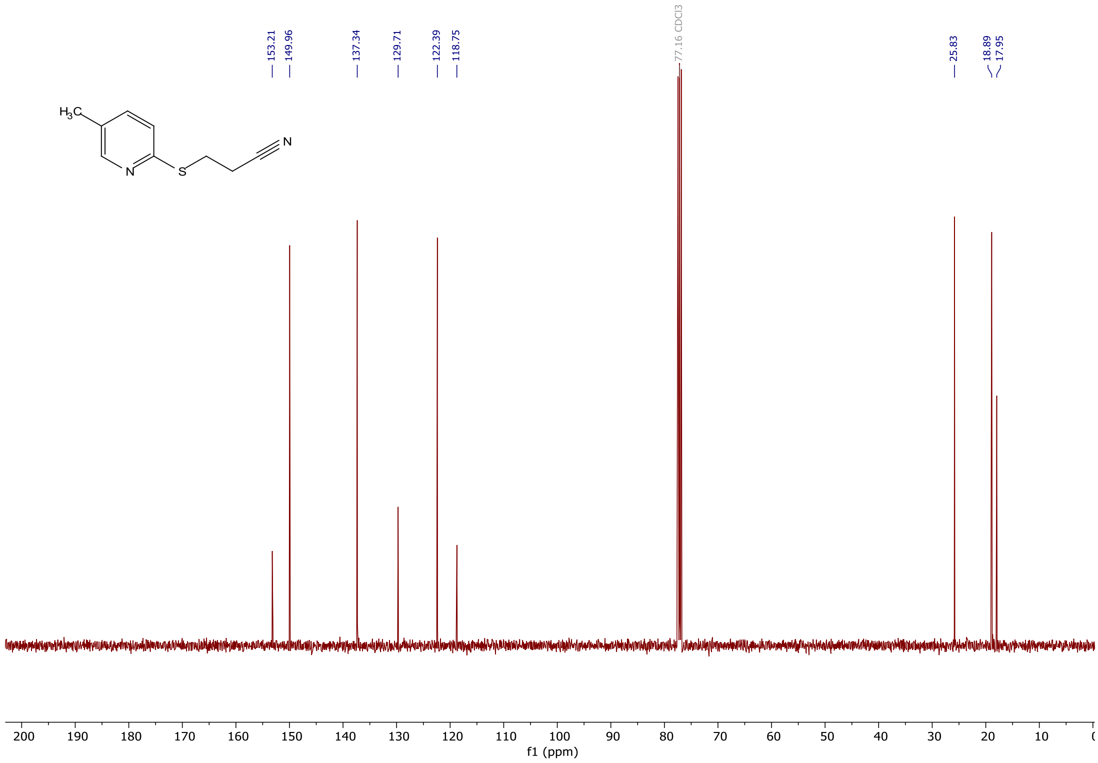

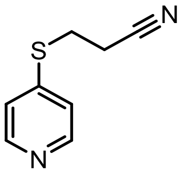

3-(pyridin-4-ylthio)propanenitrile (*pre*-1e)

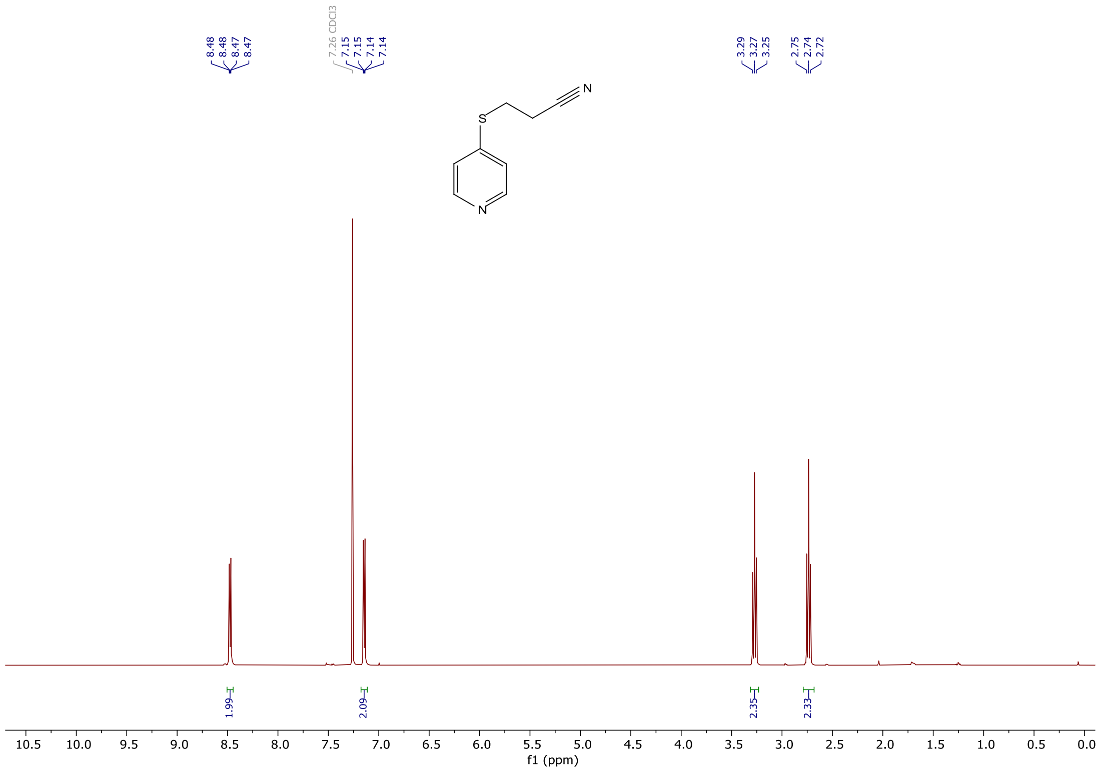

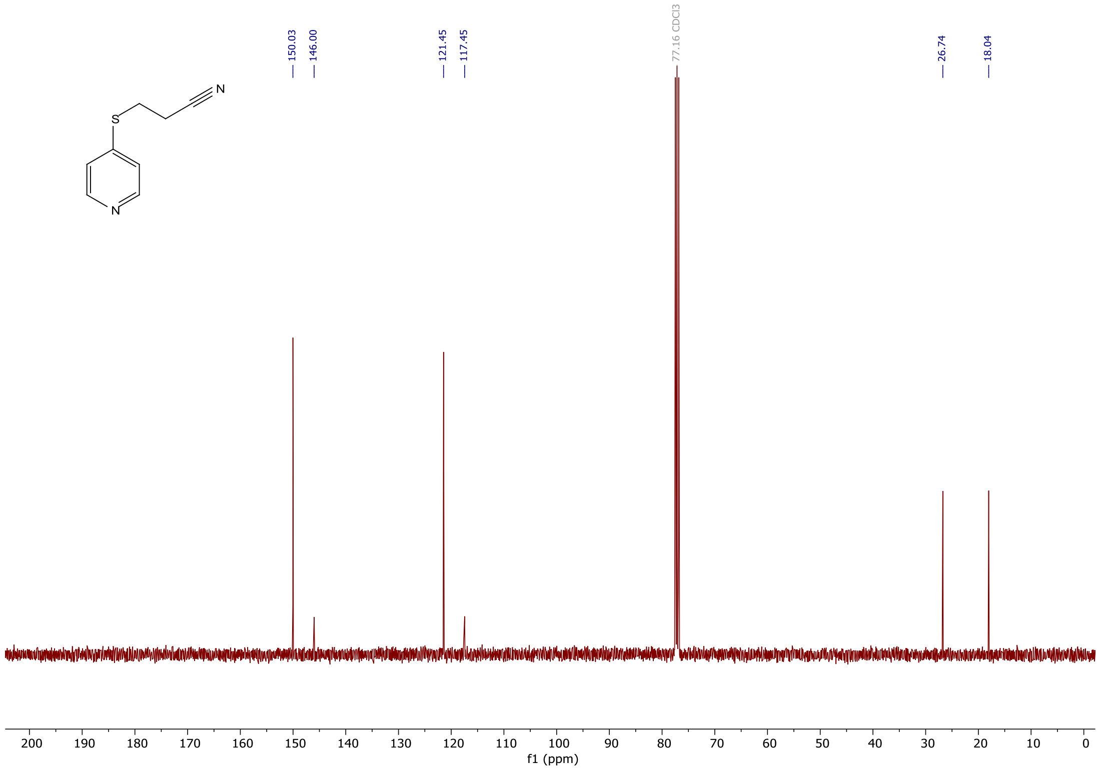

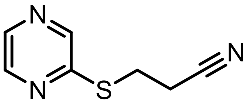

3-(pyrazin-2-ylthio)propanenitrile (*pre*-1f)

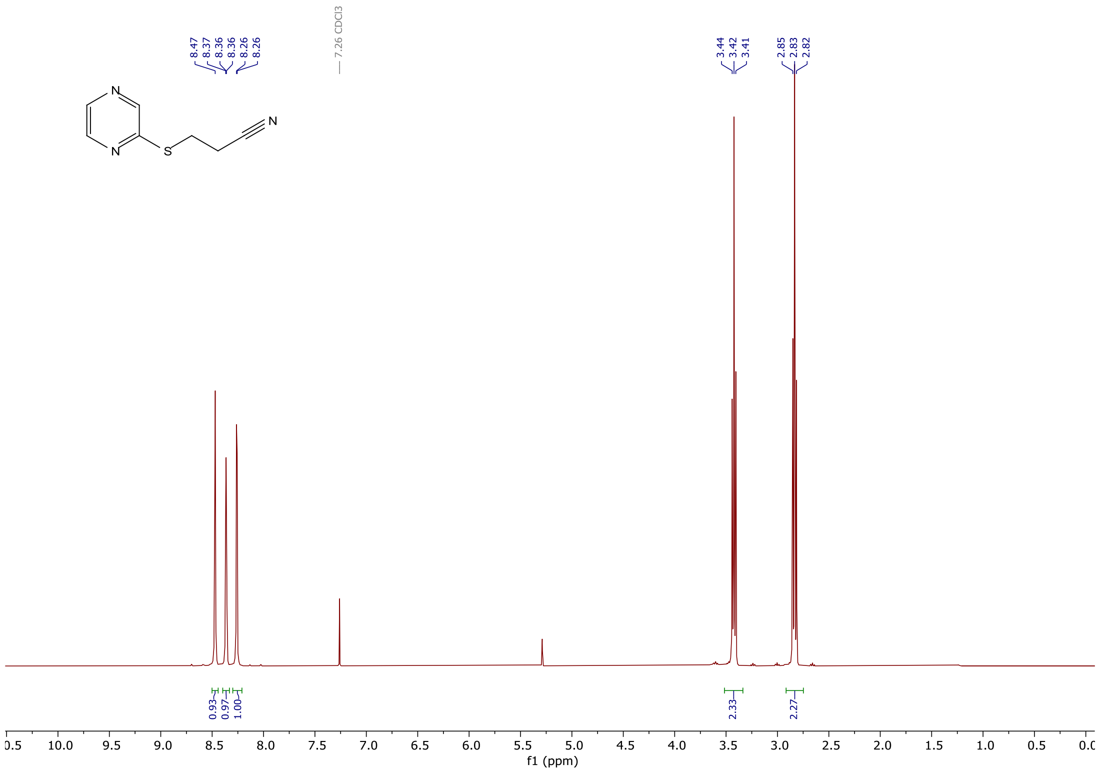

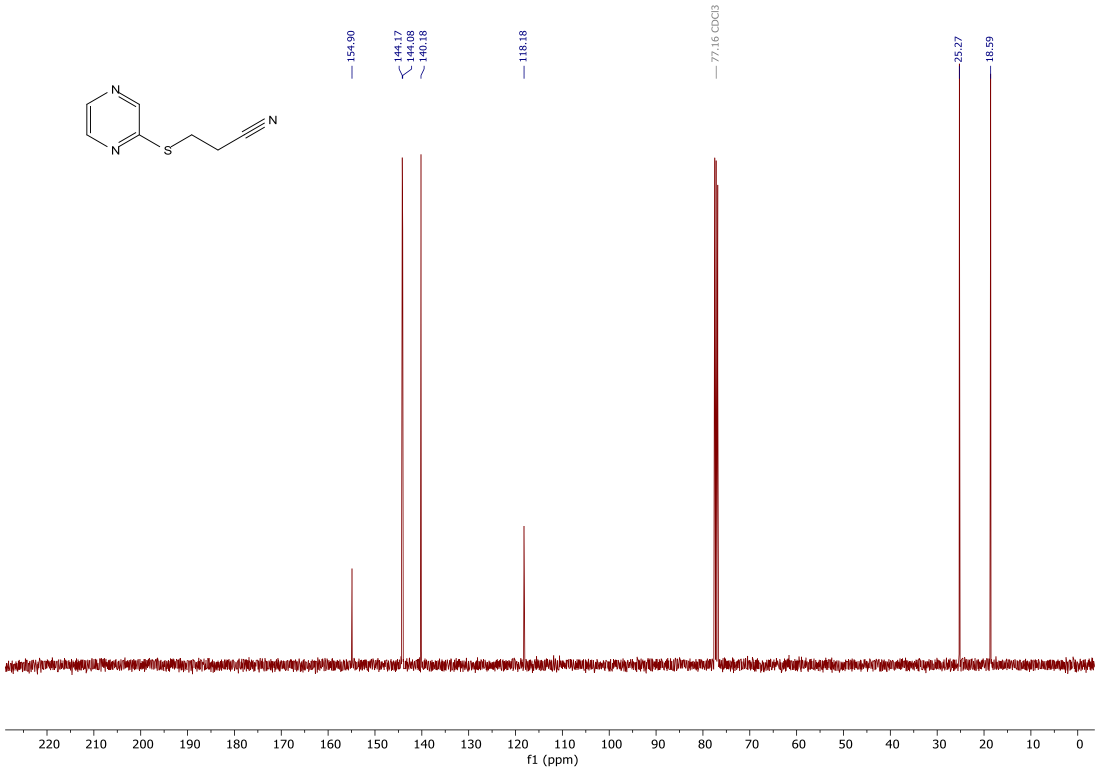

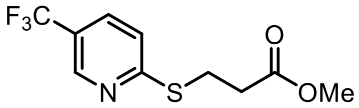

methyl 3-((5-(trifluoromethyl)pyridin-2-yl)thio)propanoate *(pre-*4a)

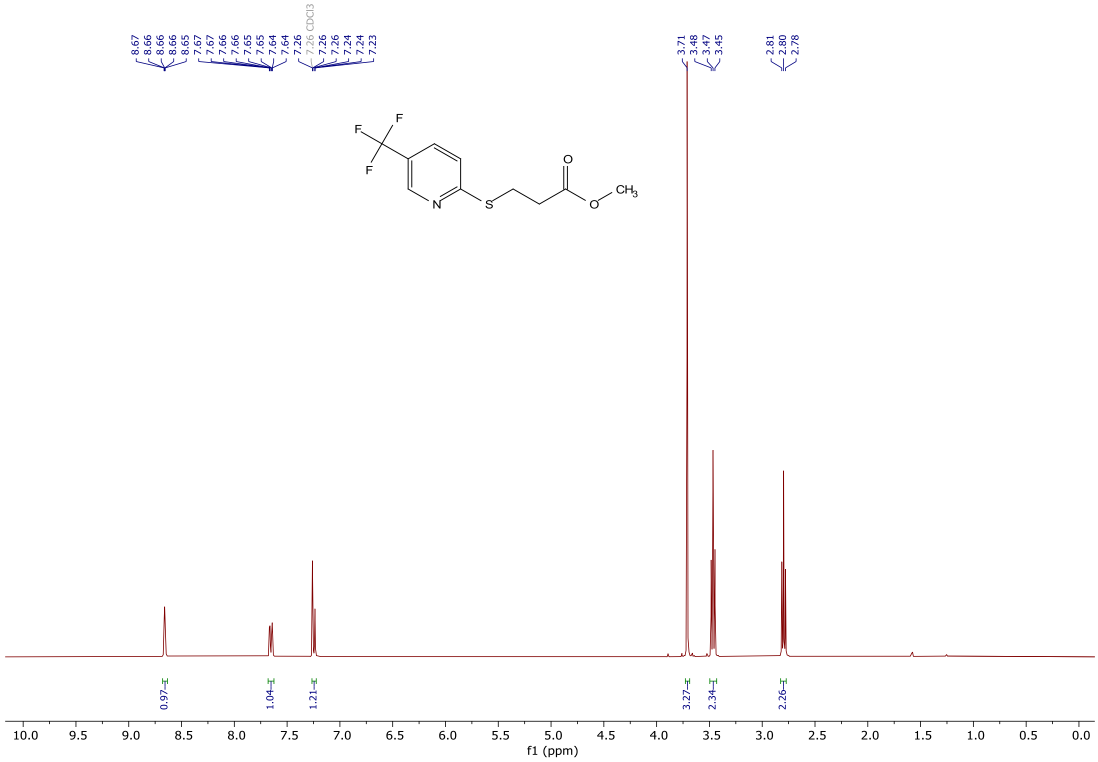

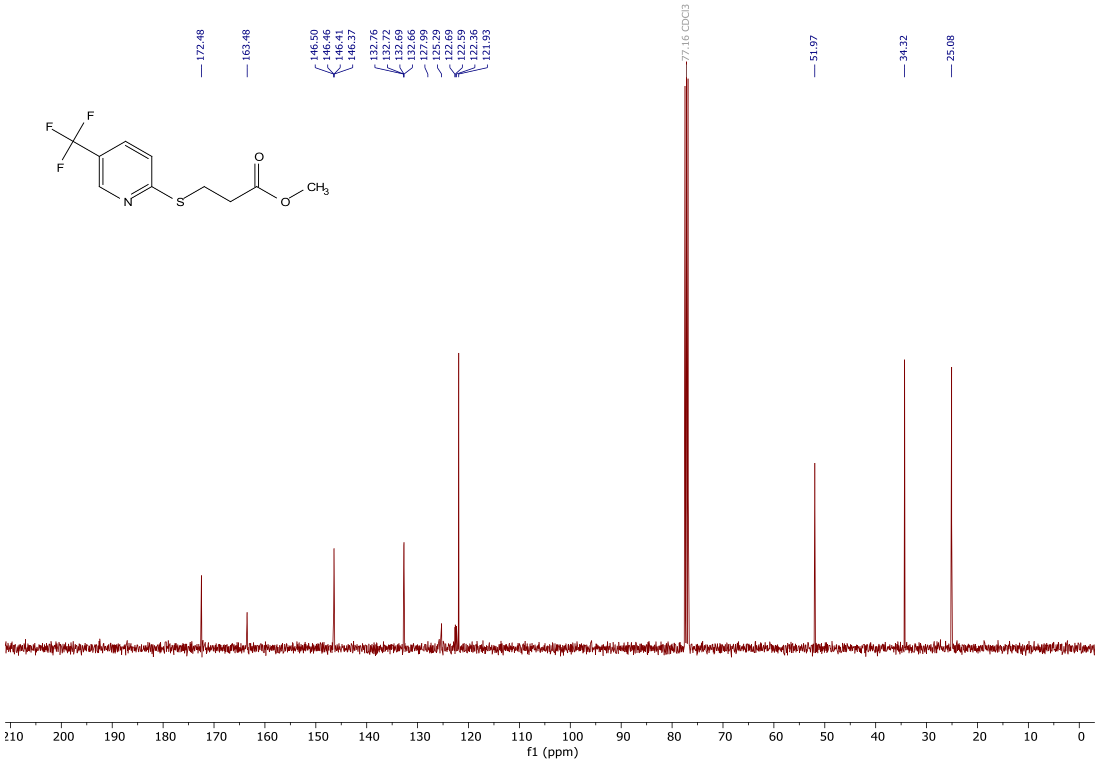

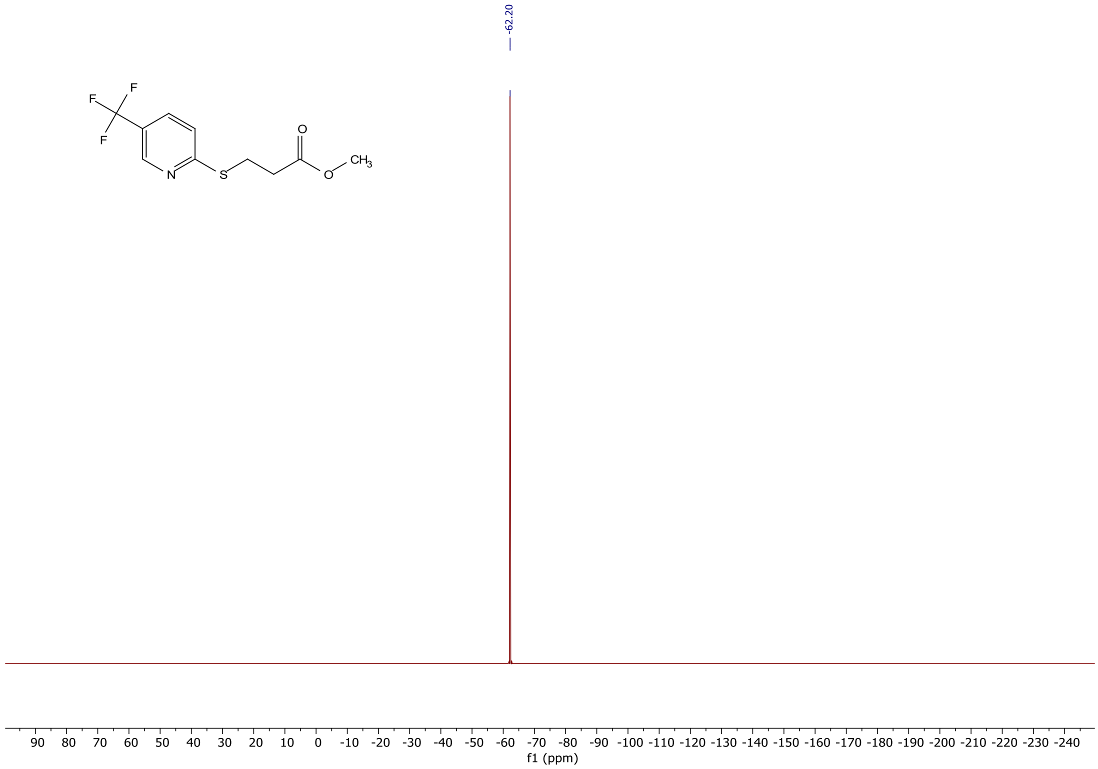

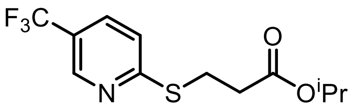

isopropyl 3-((5-(trifluoromethyl)pyridin-2-yl)thio)propanoate (*pre*-4a-iPr)

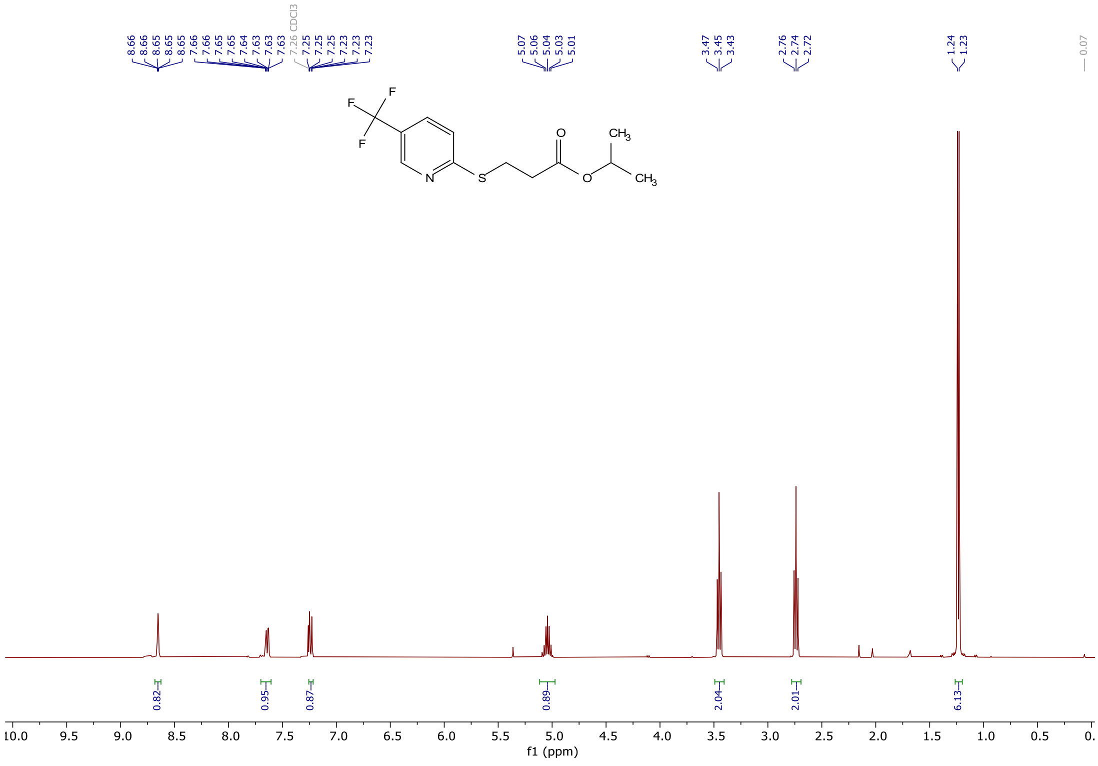

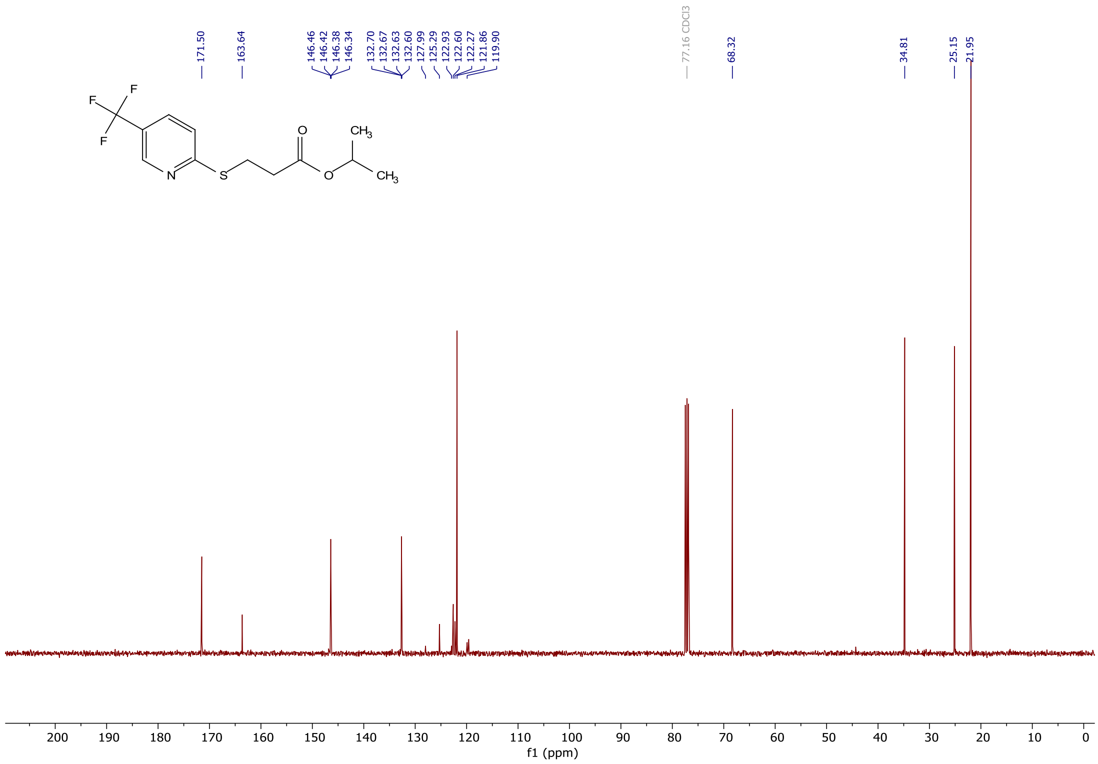
171.50
163.64
146.46
146.42
146.38
146.34
132.70
132.67
132.63
132.60
127.99
125.29
122.93
122.60
122.27
121.86
119.90
77.16 CDCl3
68.32
34.81
25.15
21.95
F
F
F
N
S
O
O
CH3
CH3
f1 (ppm)

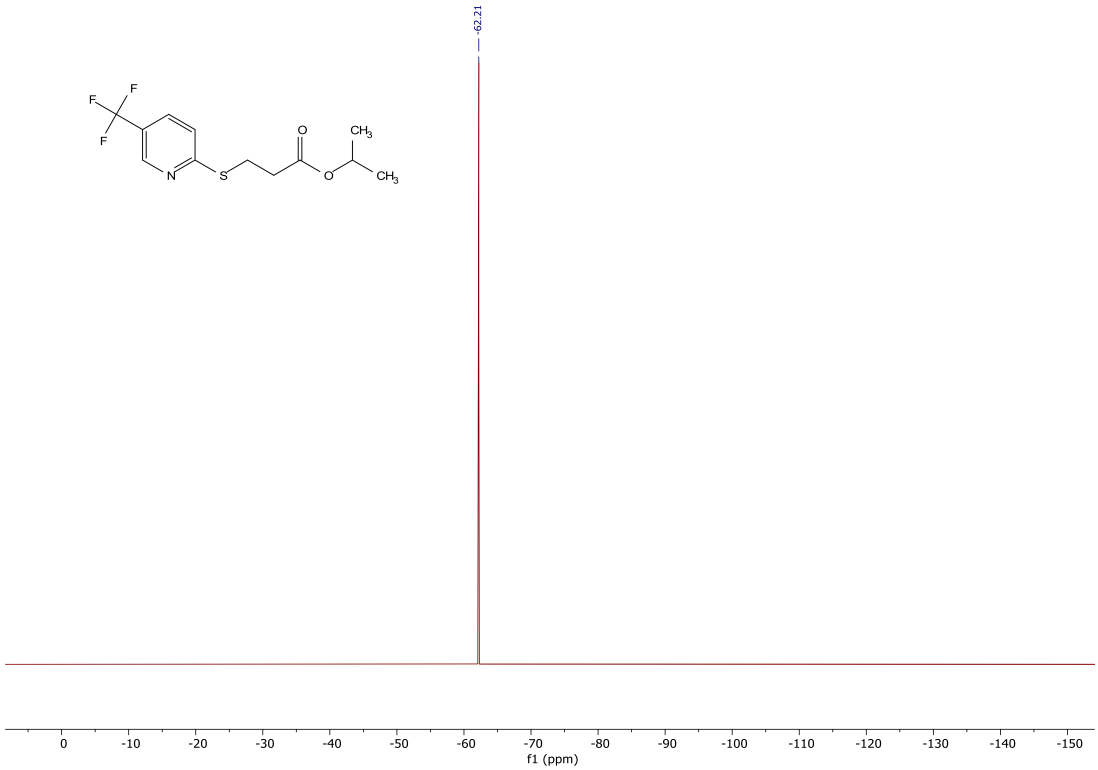
-62.21
F
F
F
N
S
O
O
CH3
CH3
f1 (ppm)

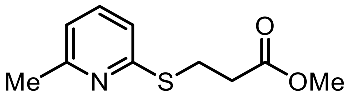

methyl 3-((6-methylpyridin-2-yl)thio)propanoate (*pre*-4b)

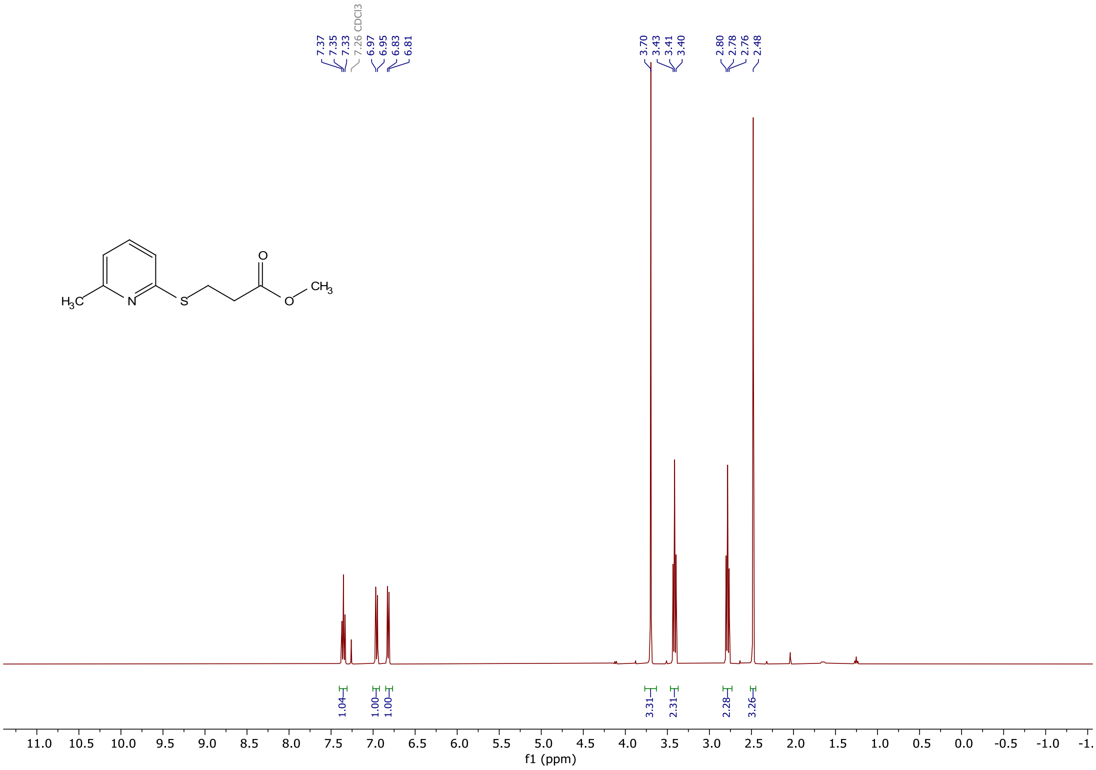

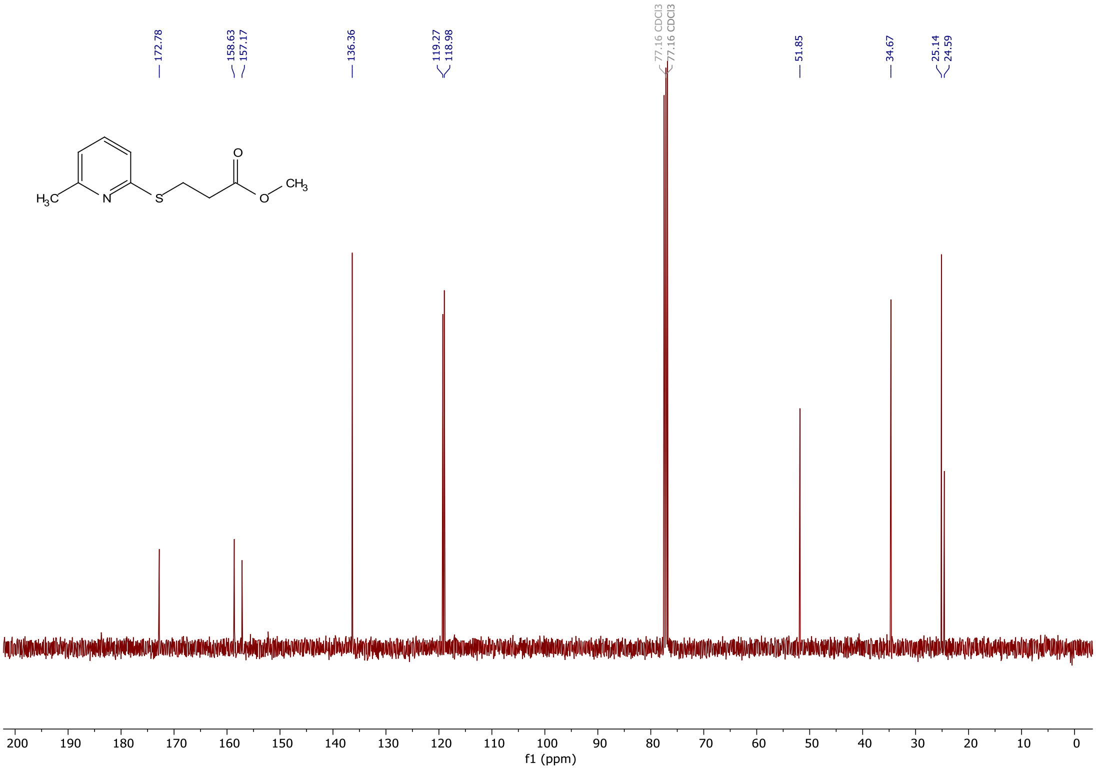

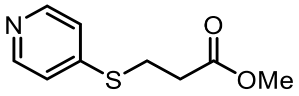

methyl 3-(pyridin-4-ylthio)propanoate (*pre*-4c)

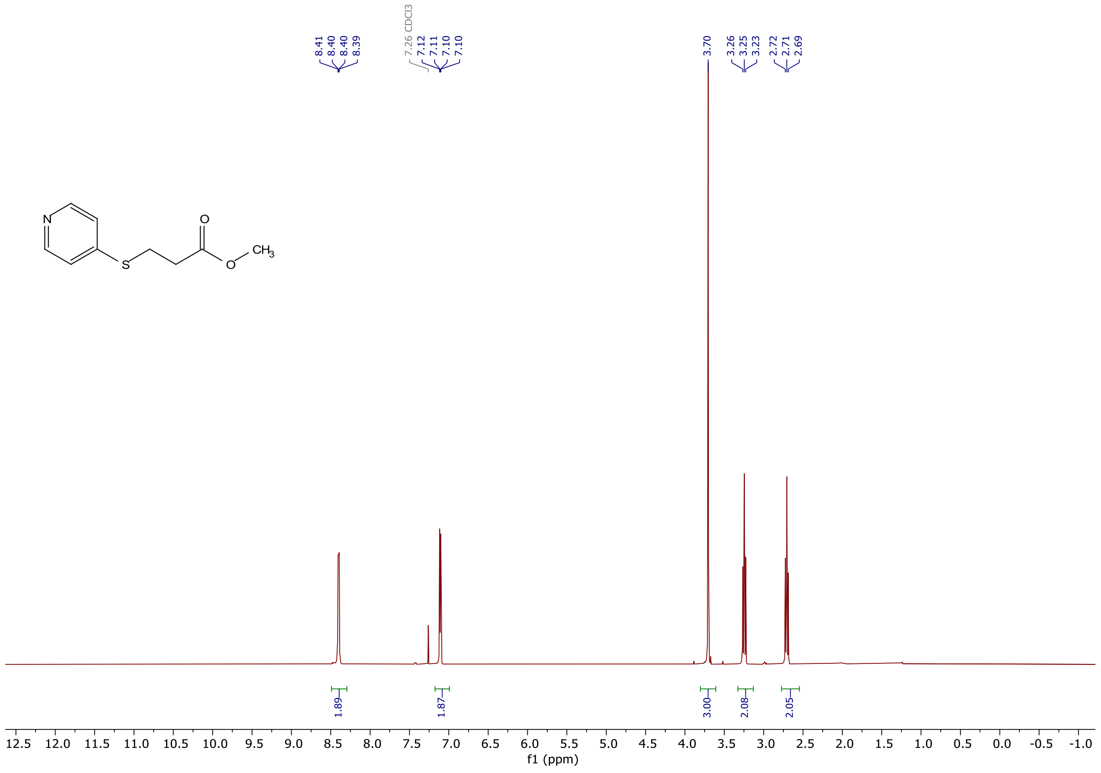

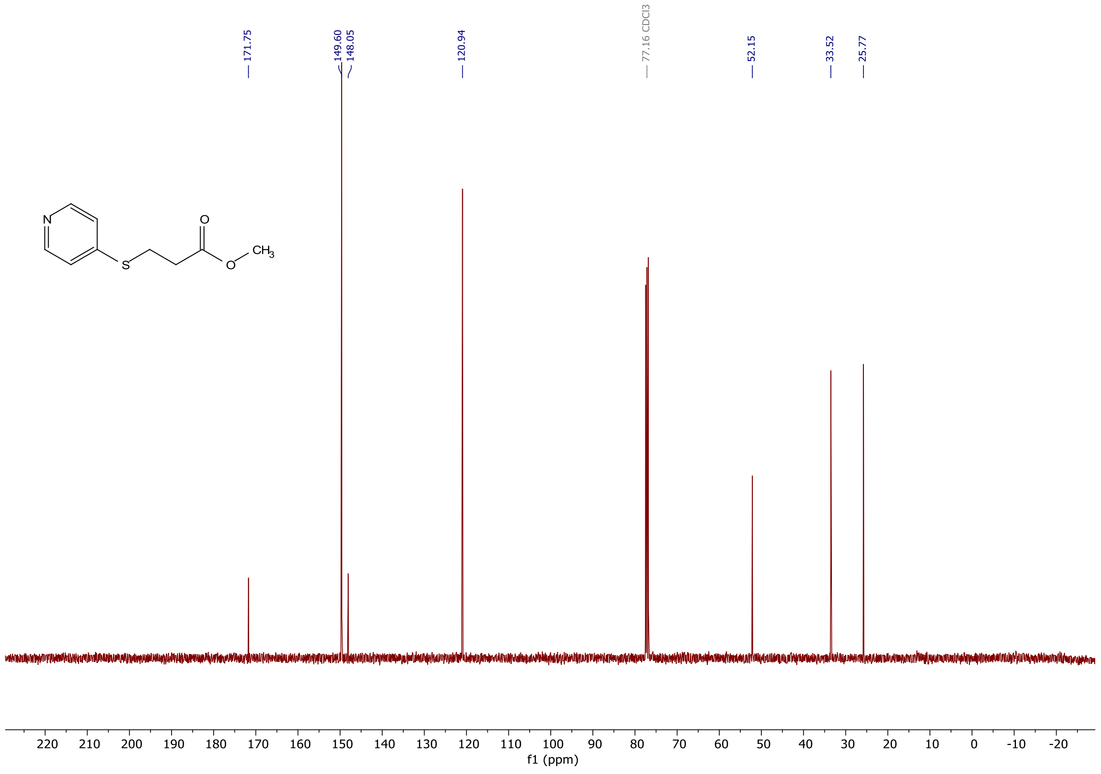

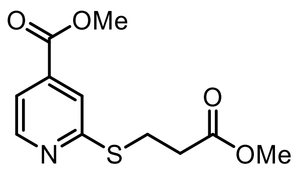

methyl 2-((3-methoxy-3-oxopropyl)thio)isonicotinate (*pre*-4e)

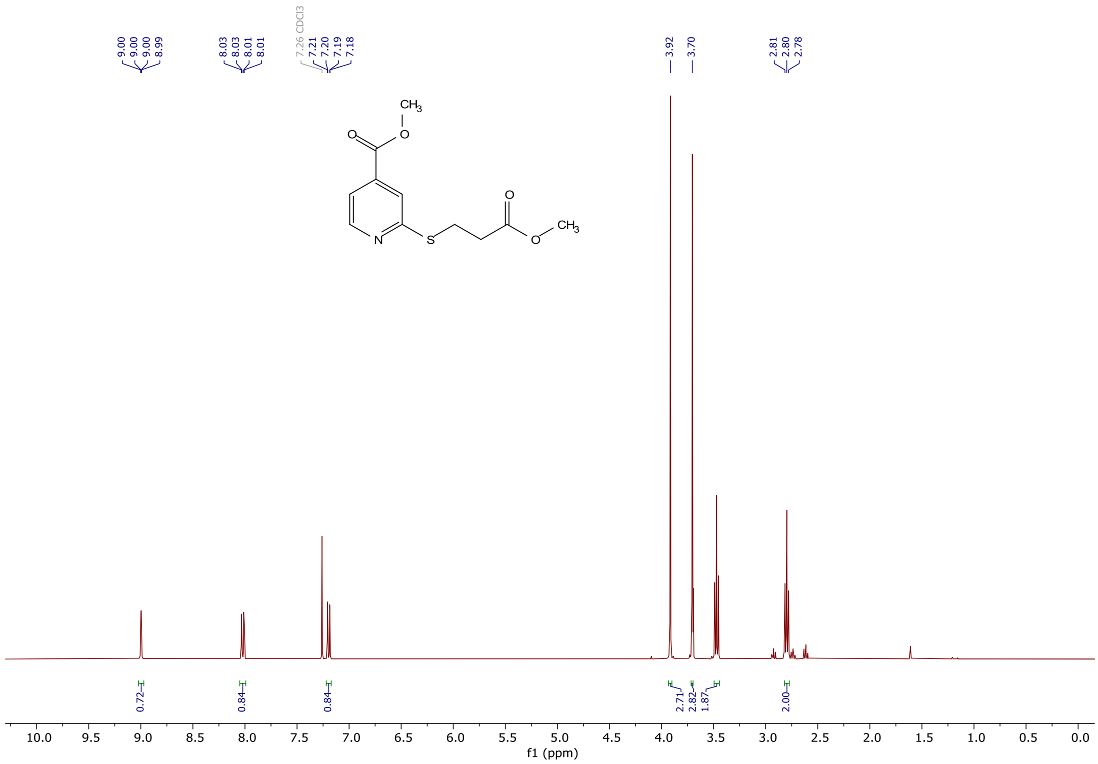

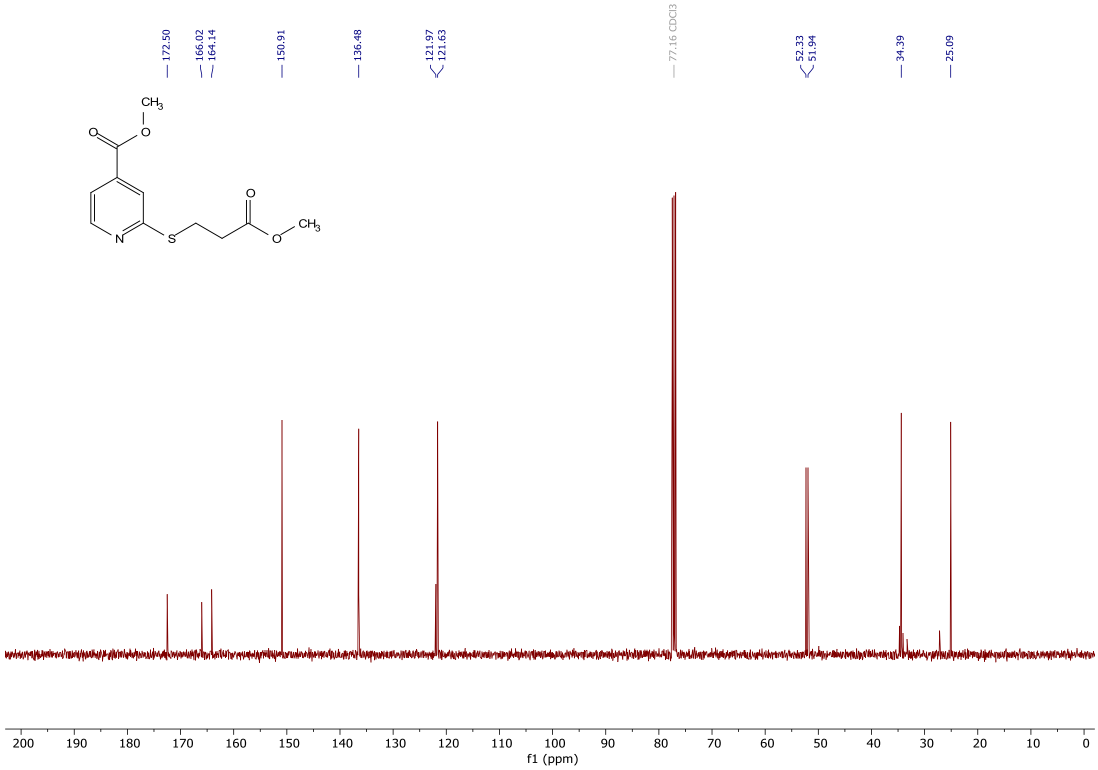

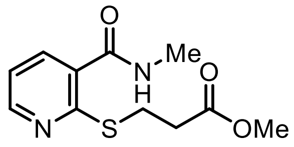

methyl 3-((3-(methylcarbamoyl)pyridin-2-yl)thio)propanoate (*pre*-4f)

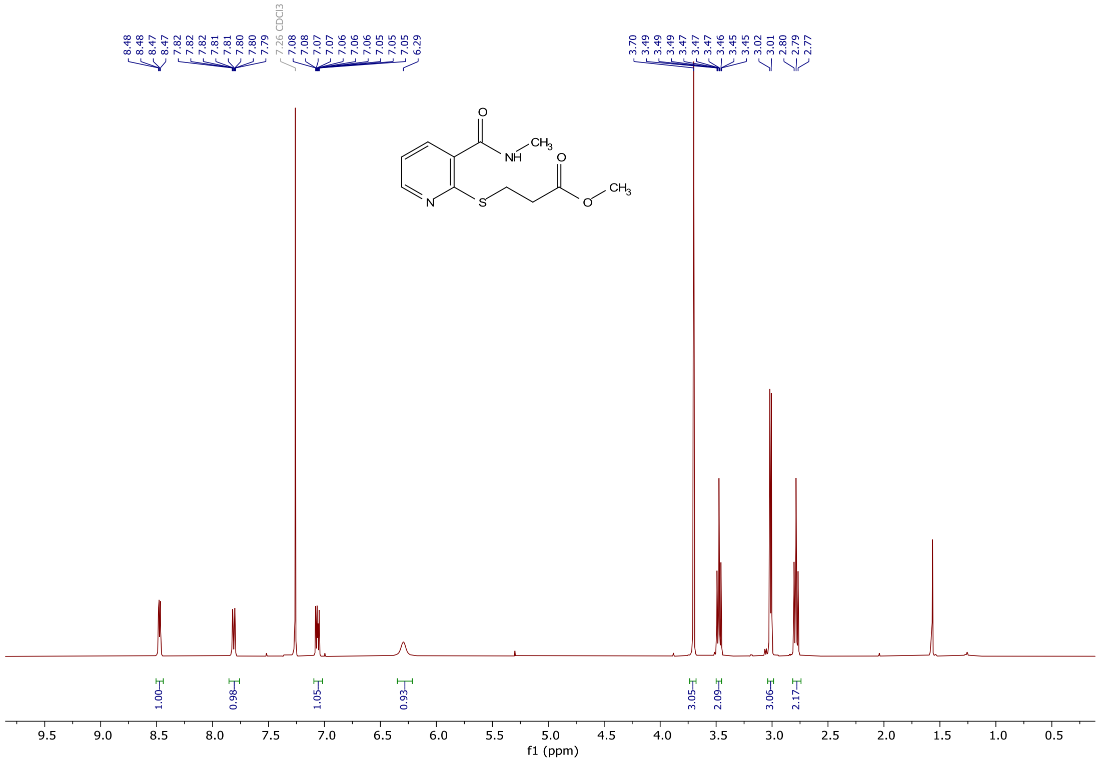

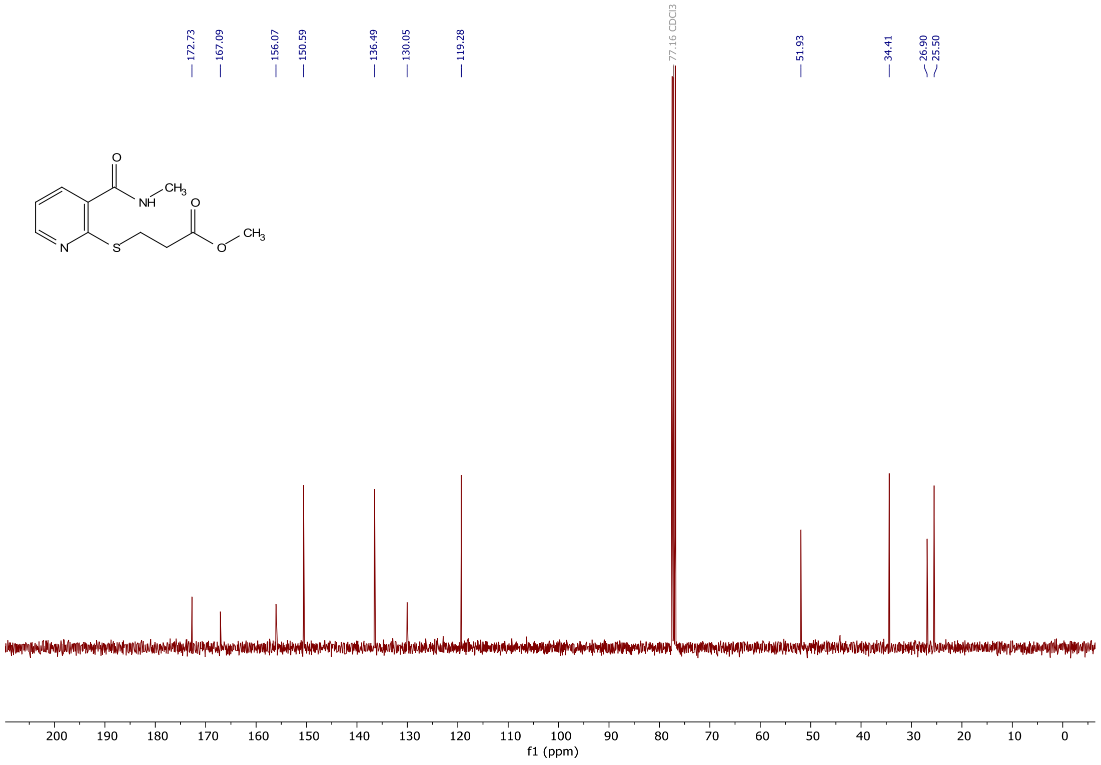

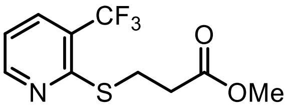

methyl 3-((3-(trifluoromethyl)pyridin-2-yl)thio)propanoate (*pre*-4g)

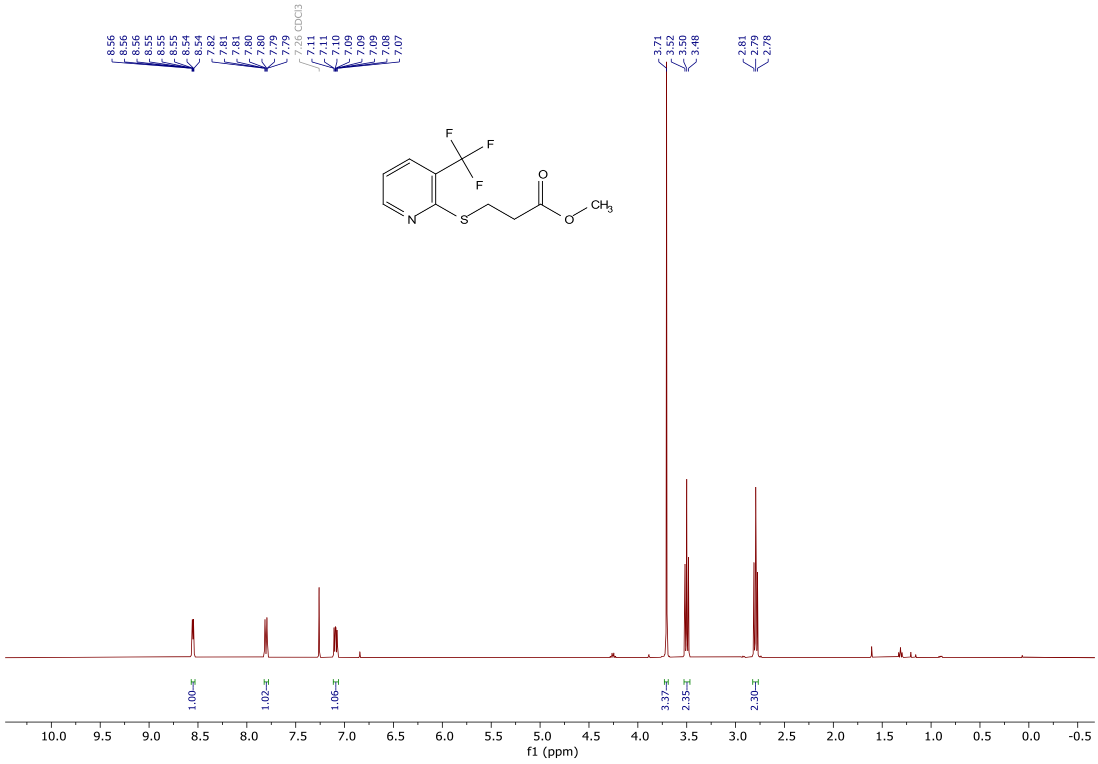

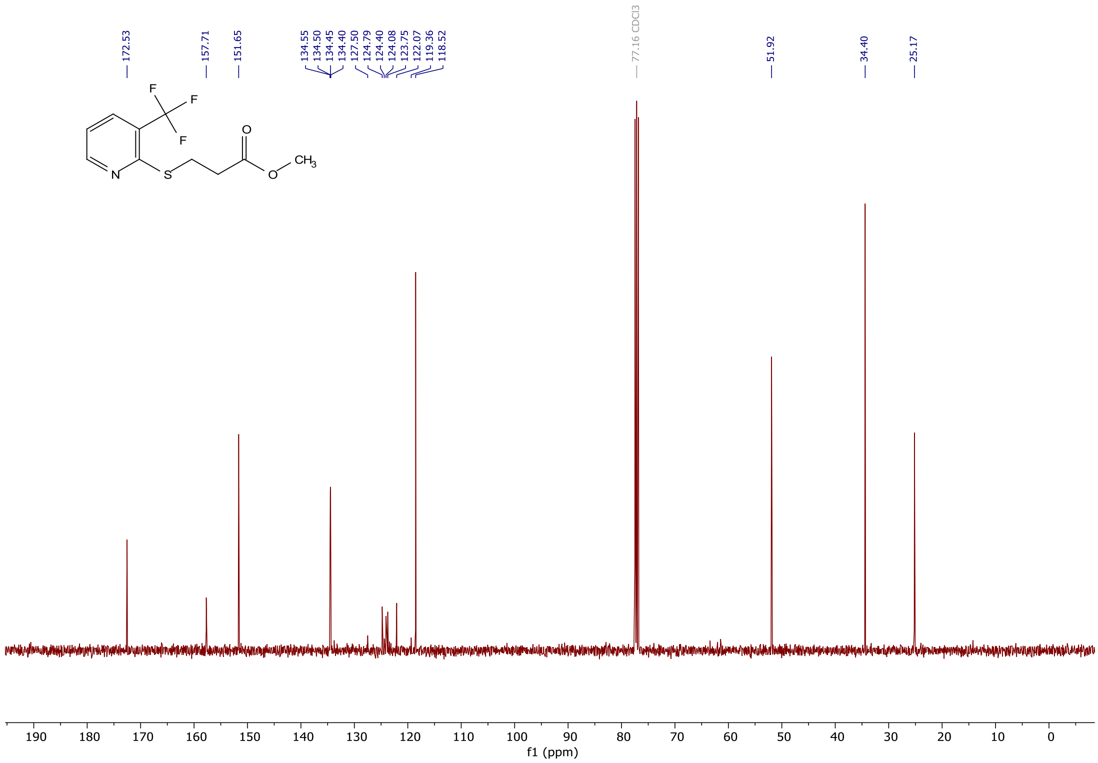

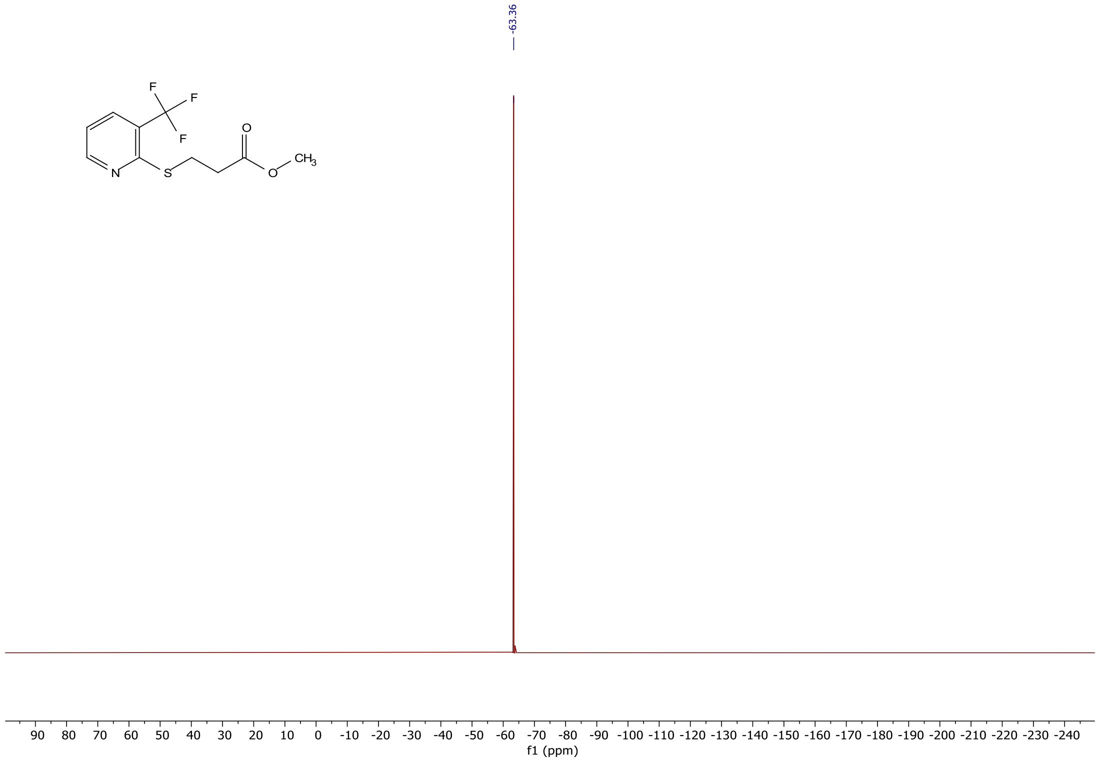

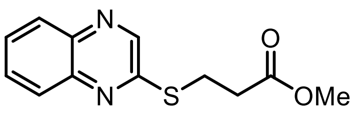

methyl 3-(quinoxalin-2-ylthio)propanoate (*pre*-4h)

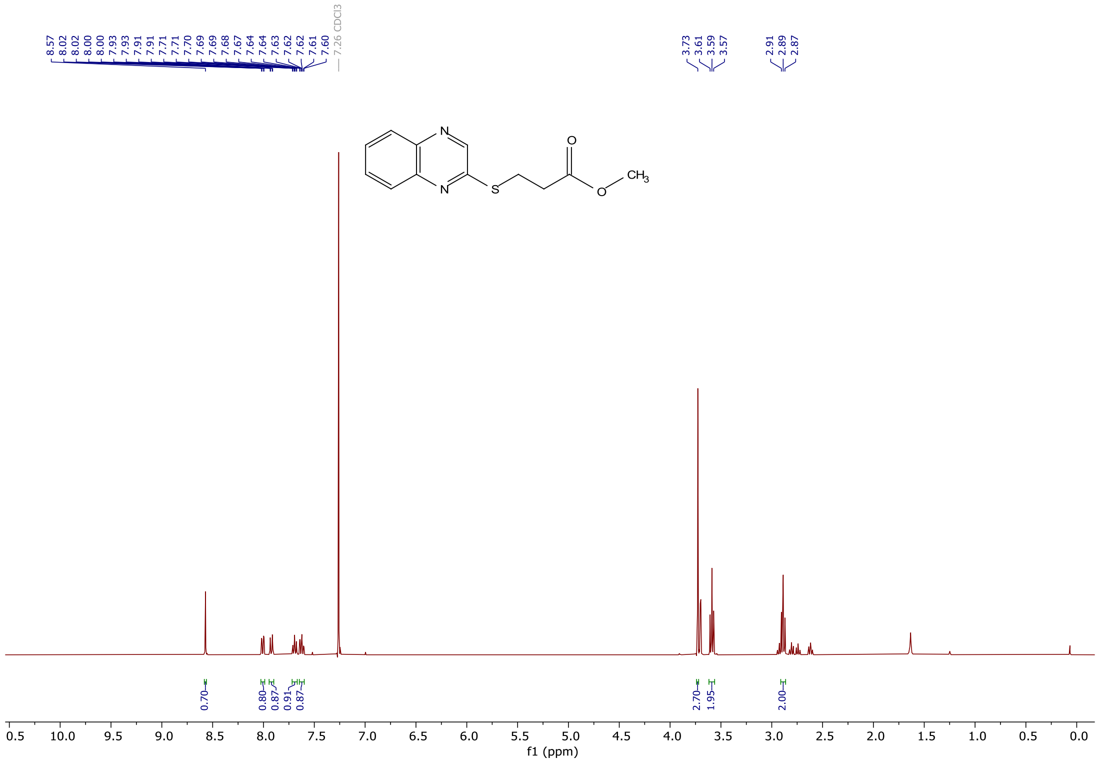

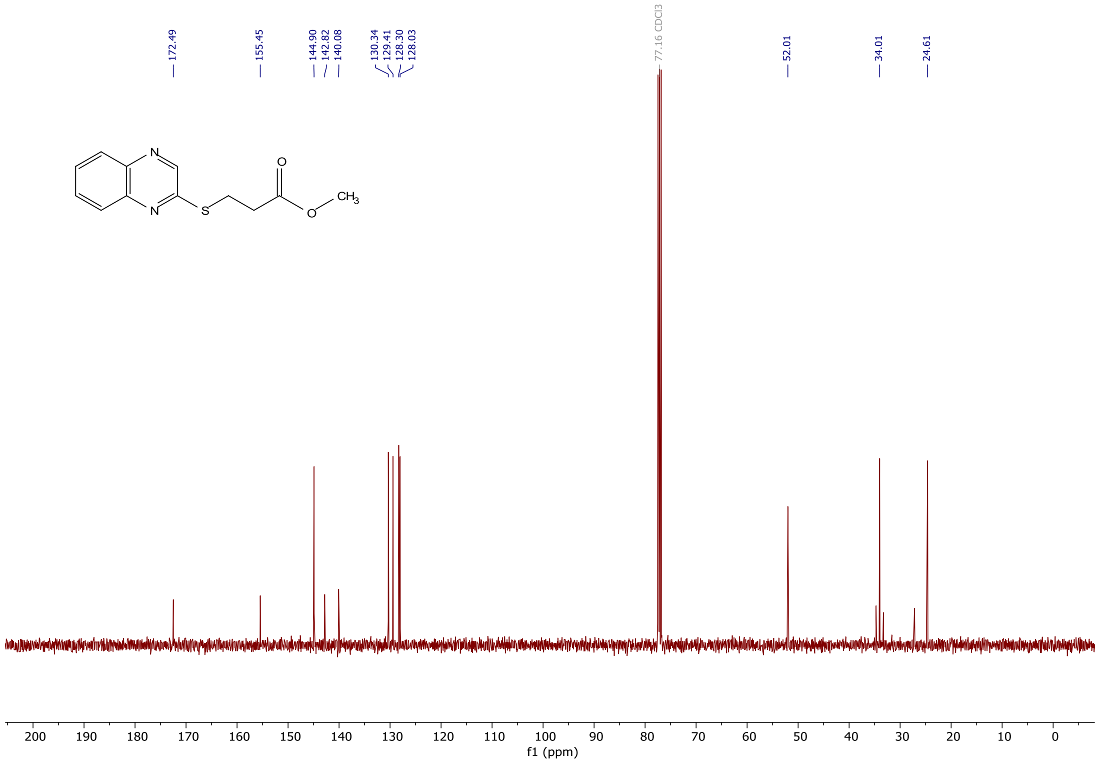

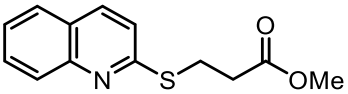

methyl 3-(quinolin-2-ylthio)propanoate (*pre*-4i)

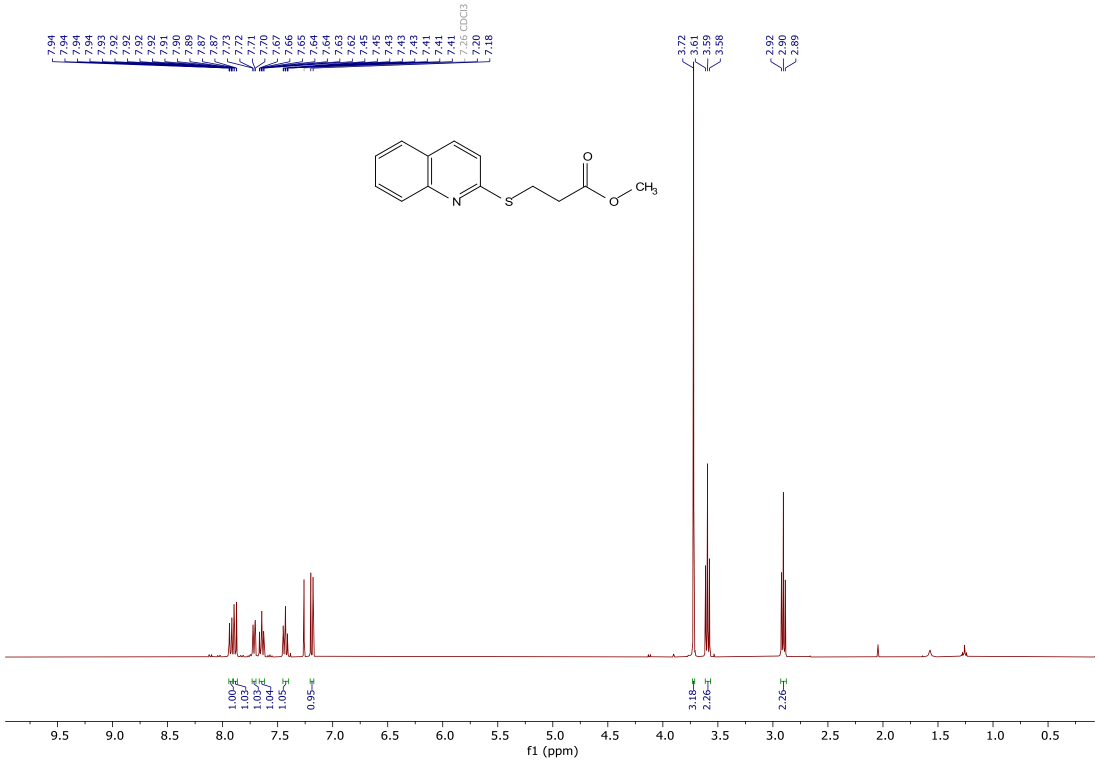

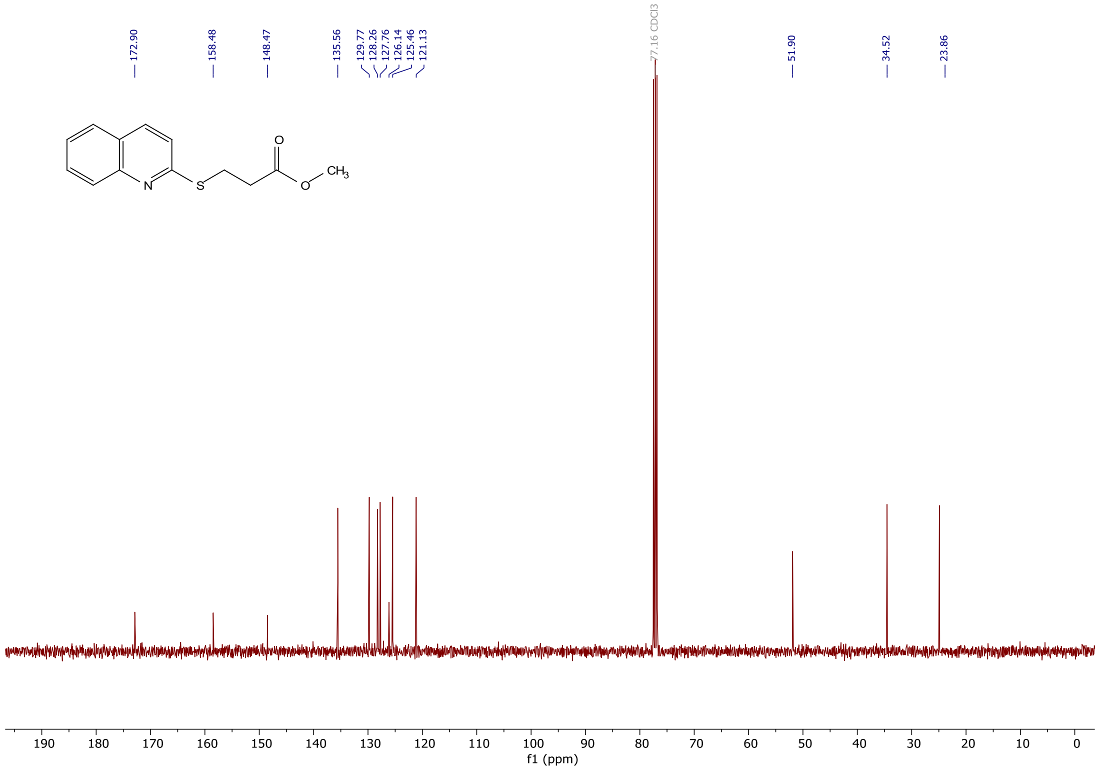

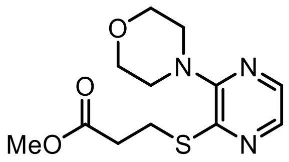

methyl 3-((3-morpholinopyrazin-2-yl)thio)propanoate (*pre*-4j)

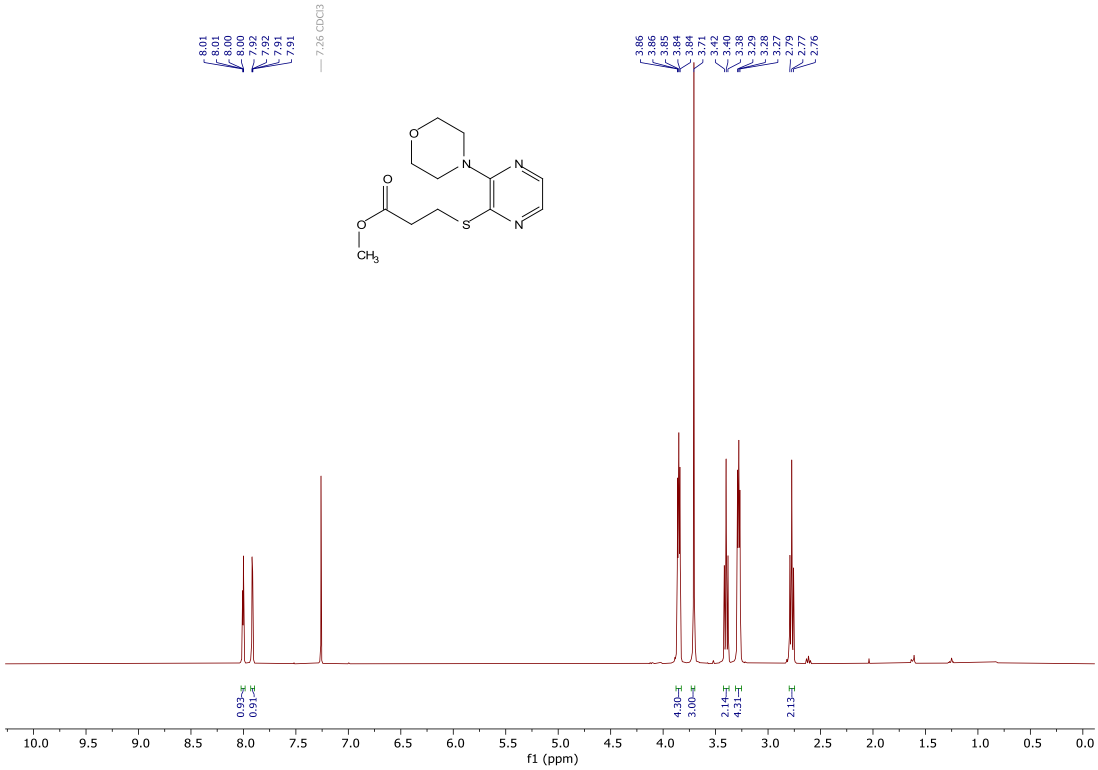

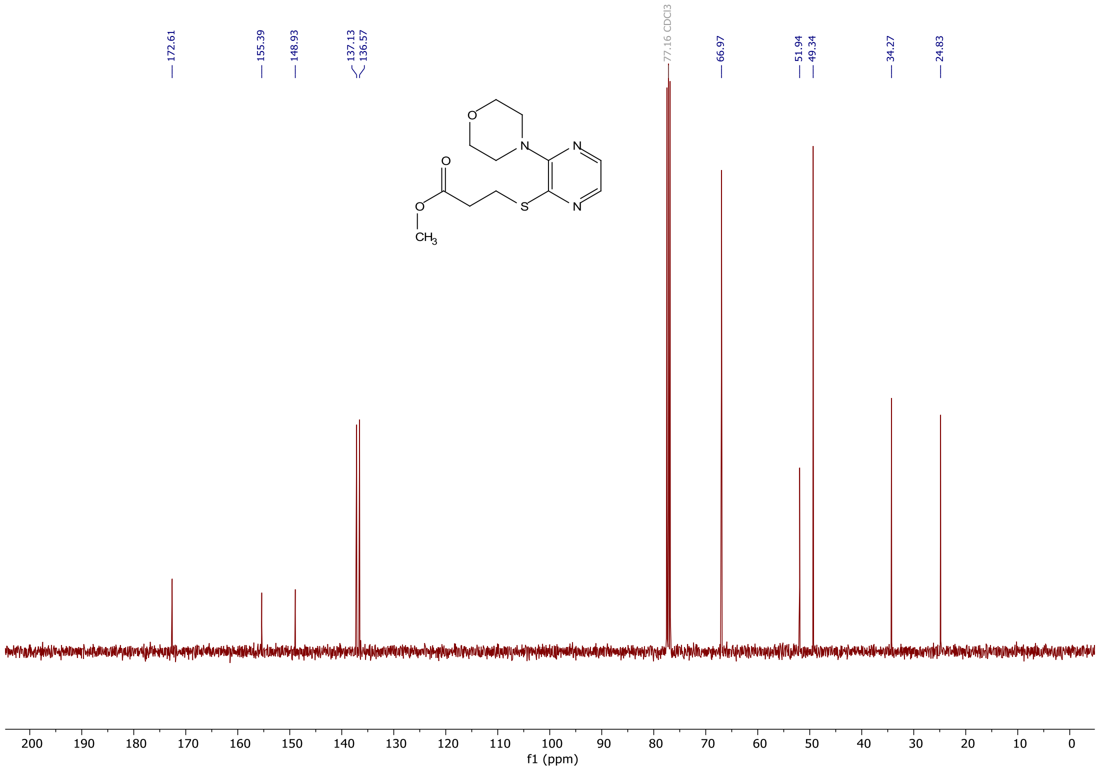

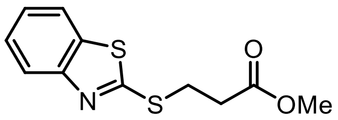

methyl 3-(benzo[d]thiazol-2-ylthio)propanoate (*pre*-4k)

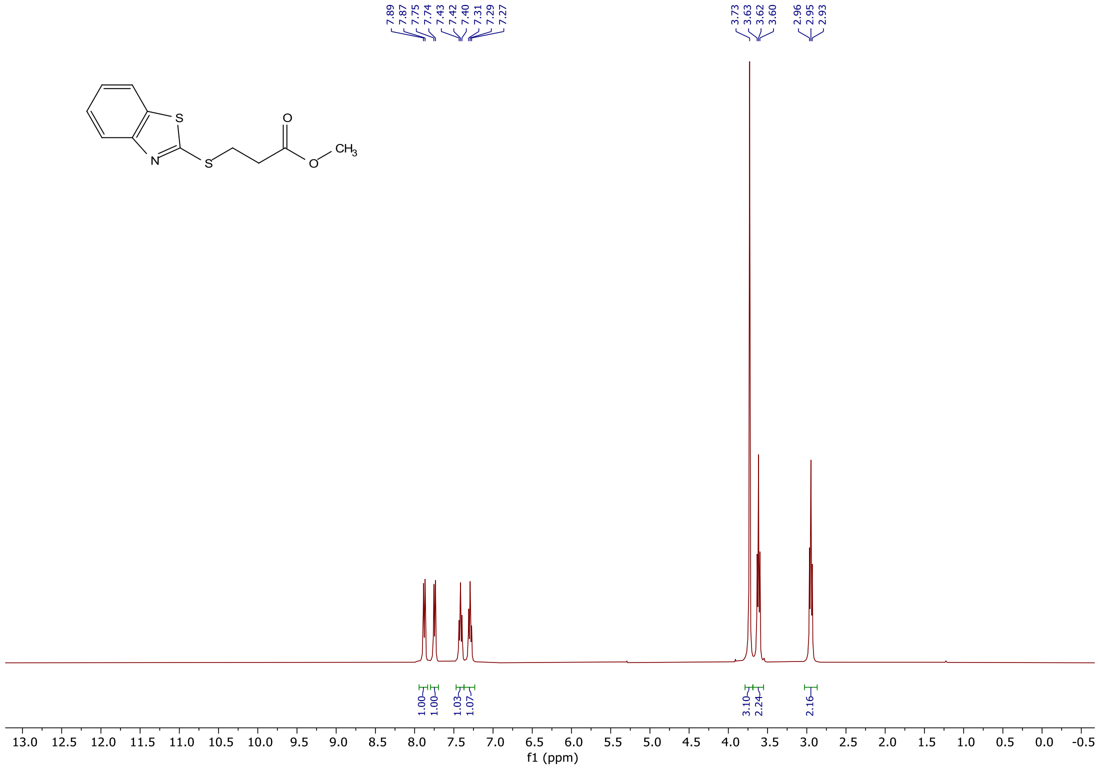

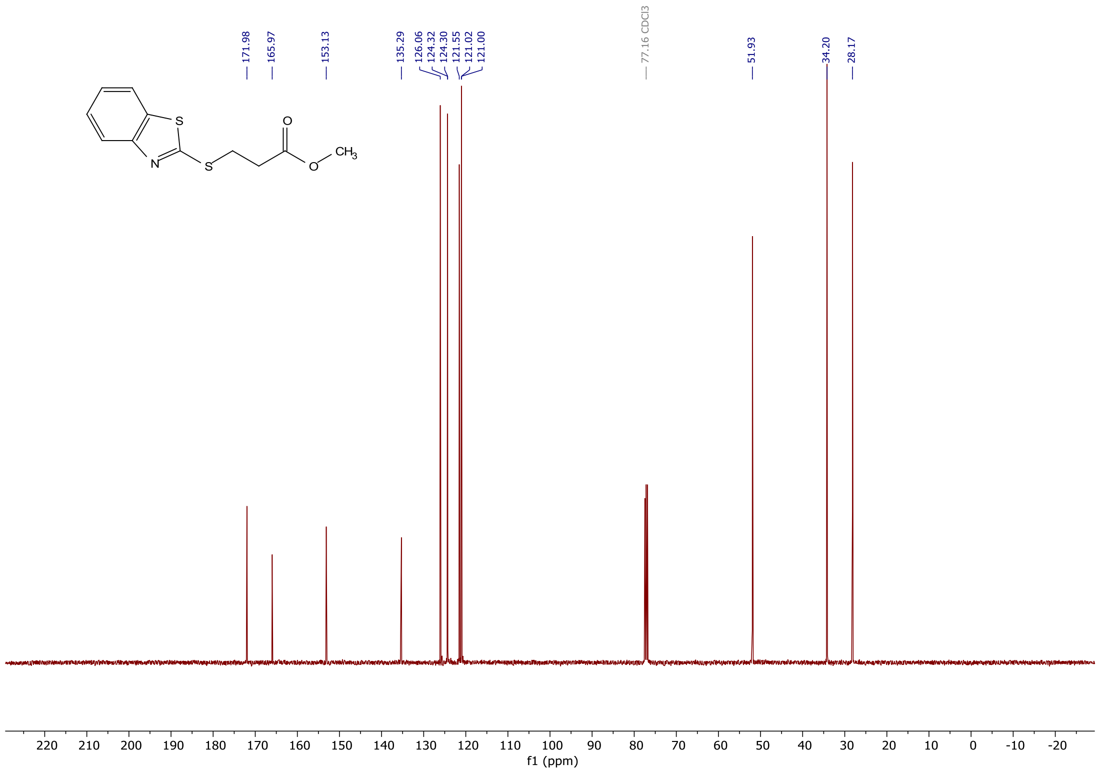

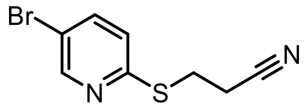

3-((5-bromopyridin-2-yl)thio)propanenitrile (*pre*-9a)

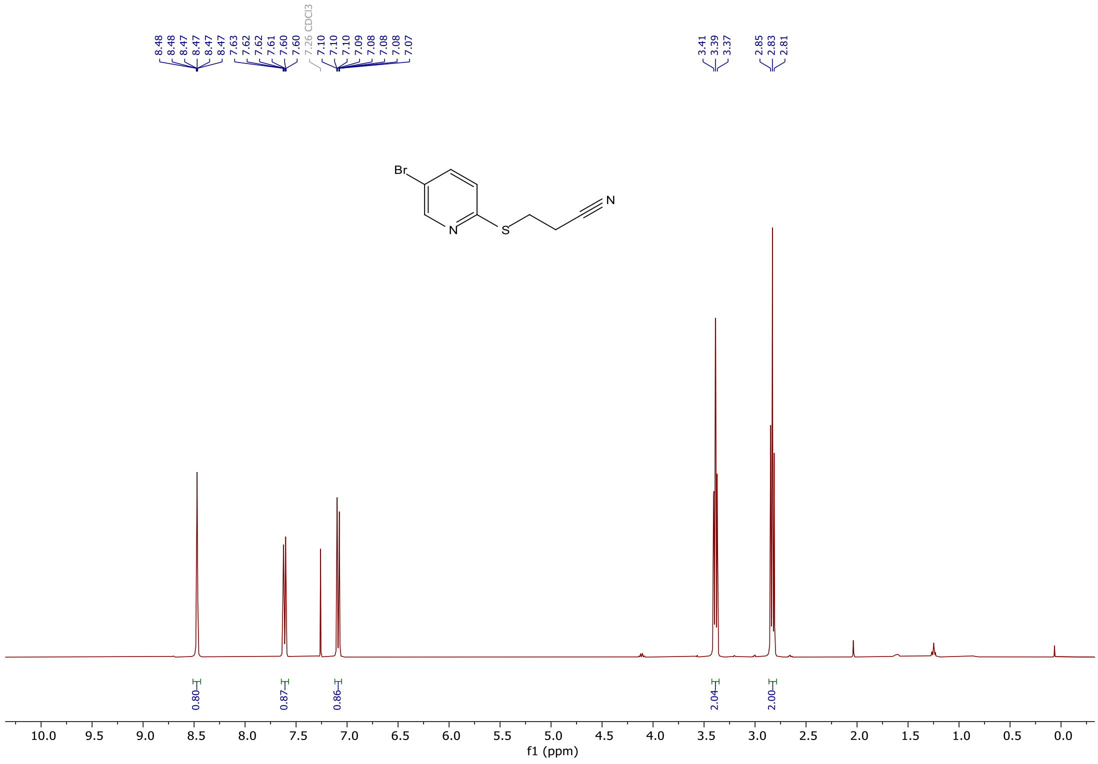

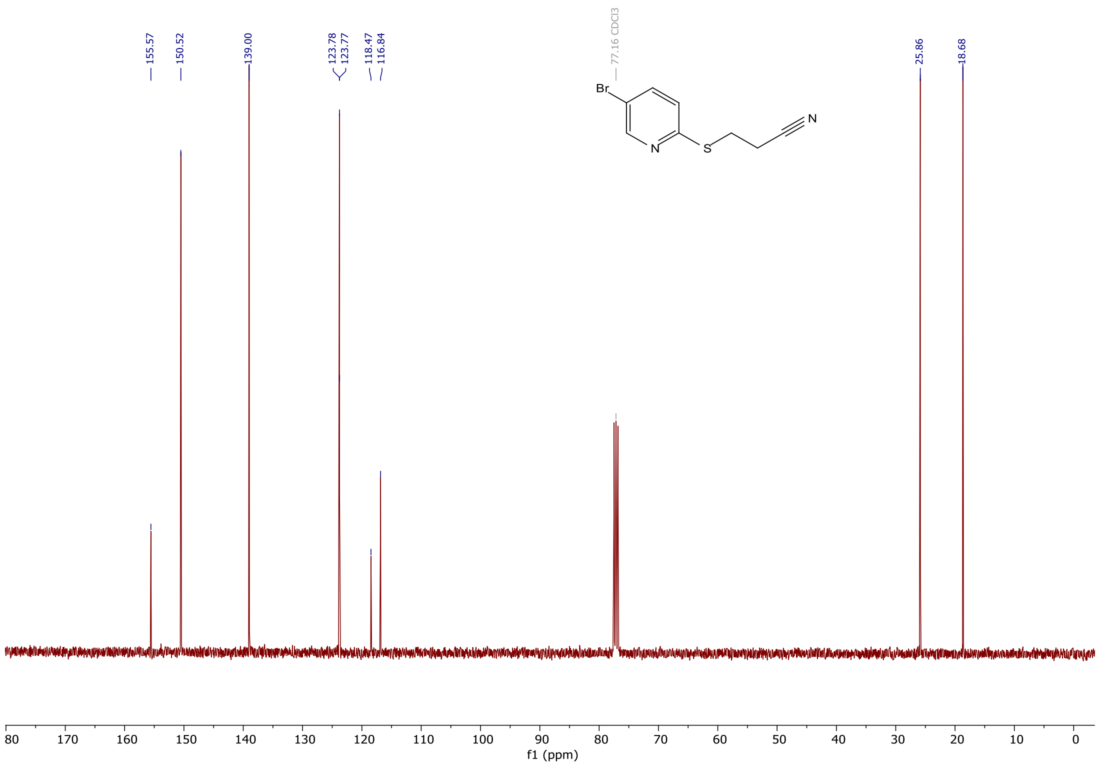

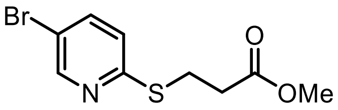

methyl 3-((5-bromopyridin-2-yl)thio)propanoate (*pre*-9b)

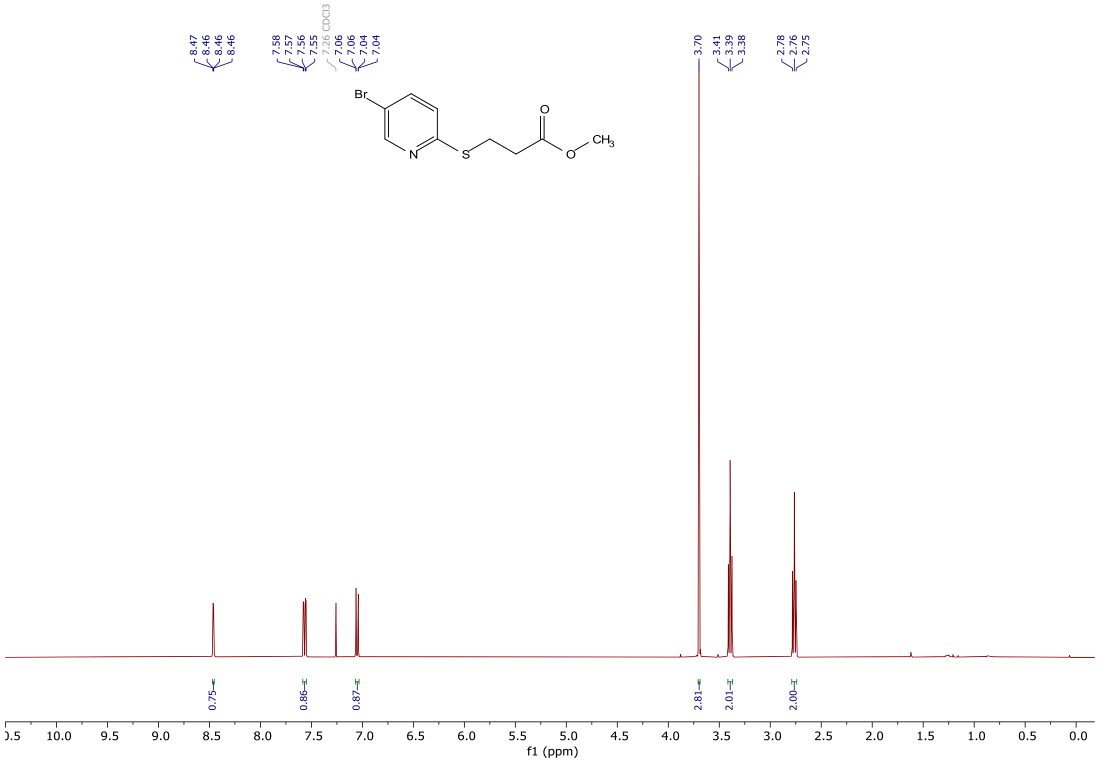

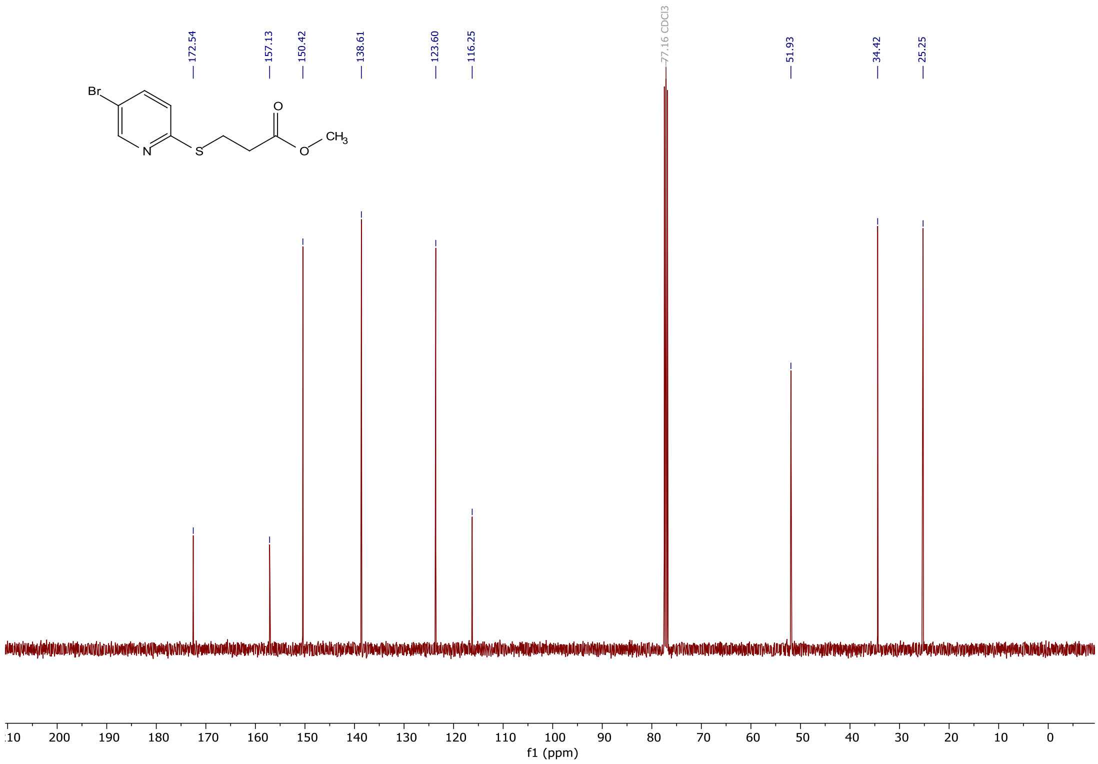

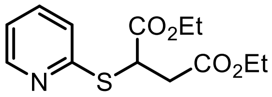

diethyl 2-(pyridin-2-ylthio)succinate (*pre*-5a)

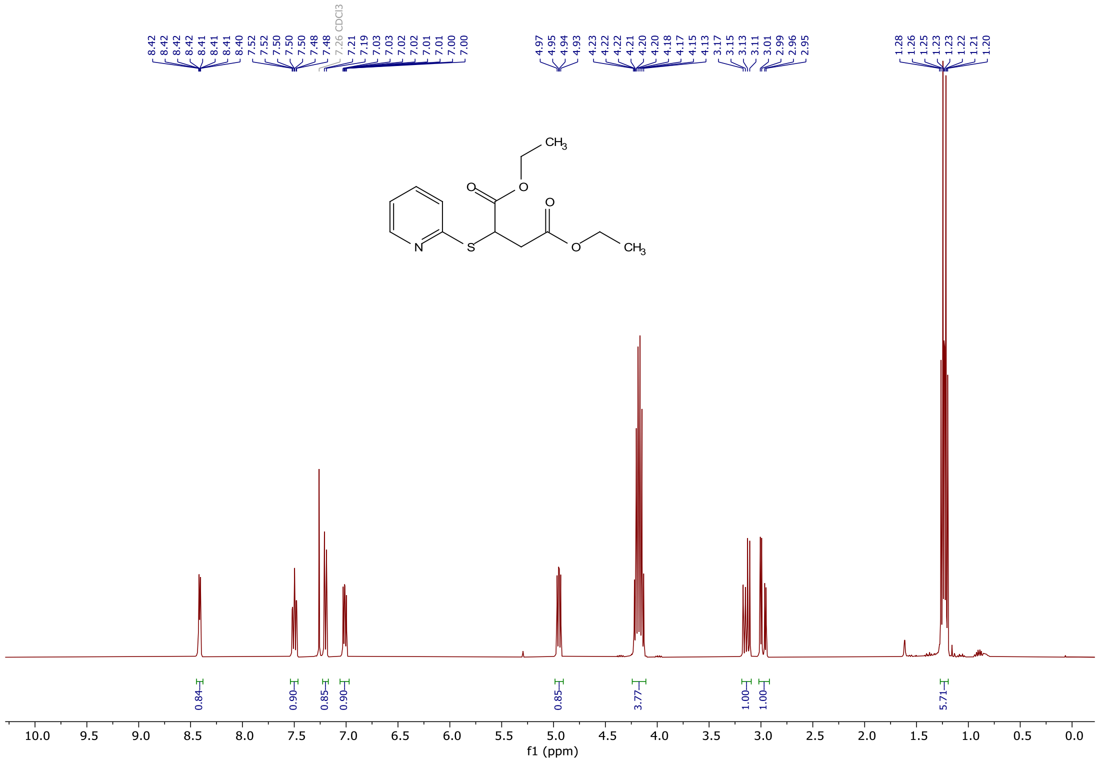

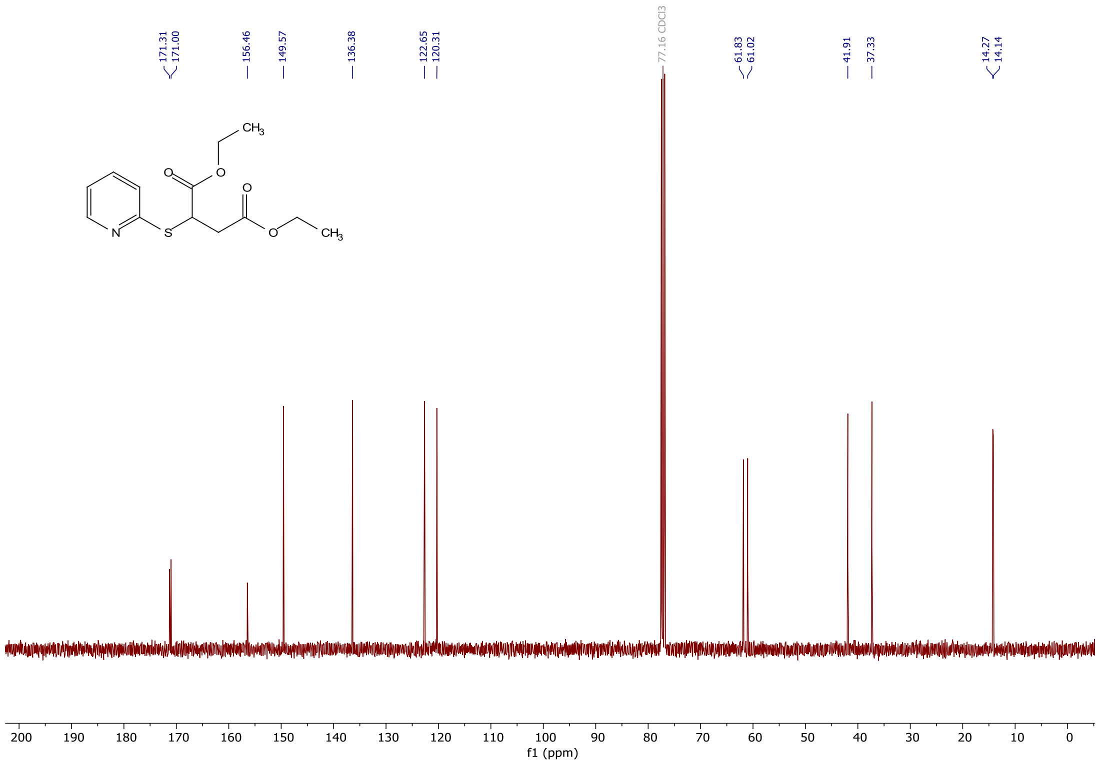

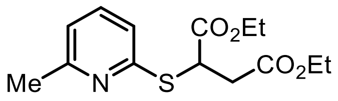

diethyl 2-((6-methylpyridin-2-yl)thio)succinate (*pre*-5b)

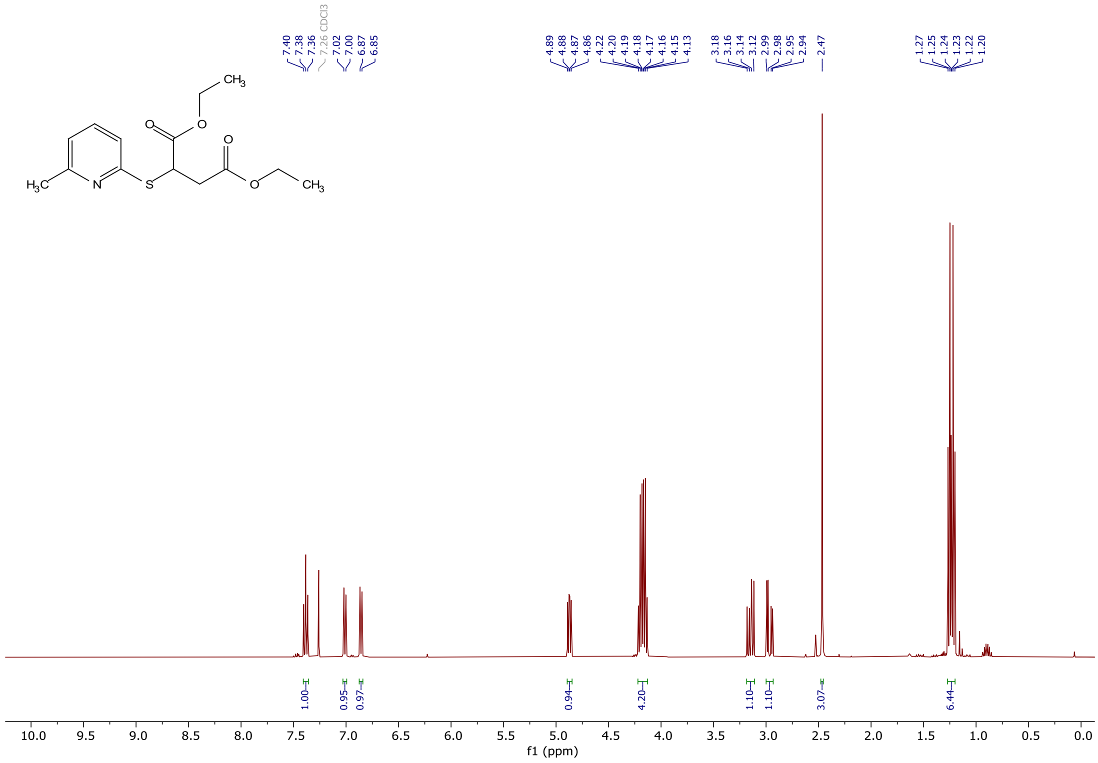

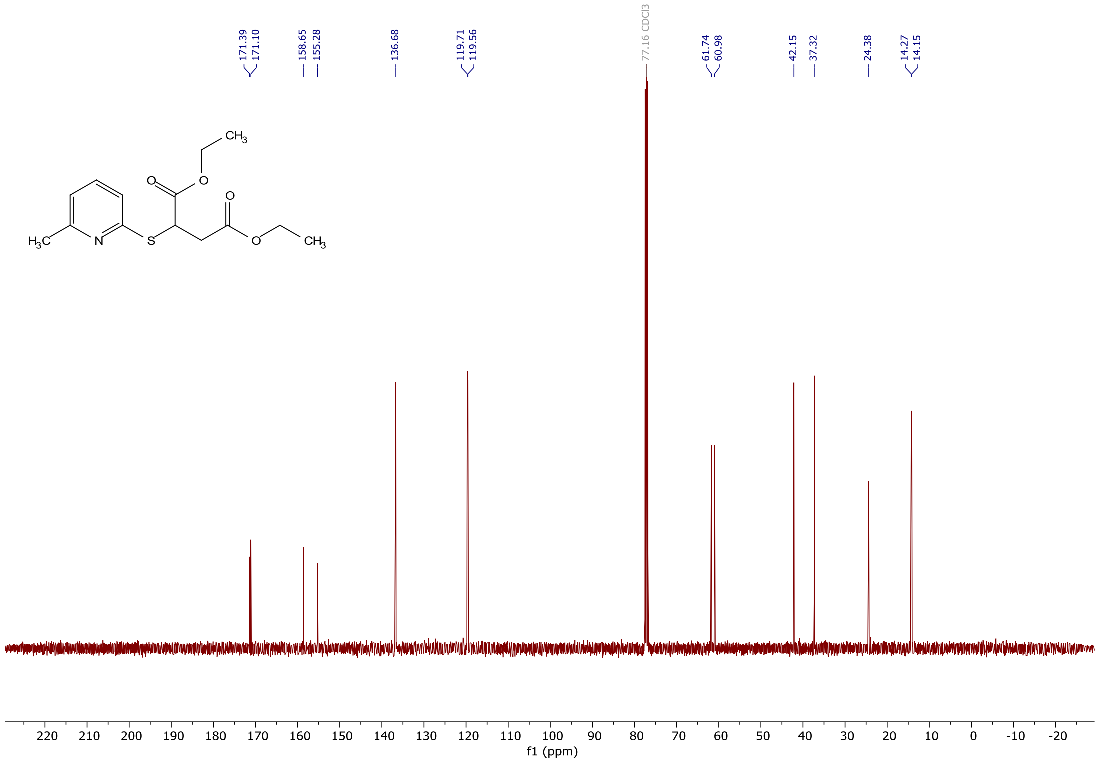
171.39
171.10
158.65
155.28
136.68
119.71
119.56
77.16 CDCl3
61.74
60.98
42.15
37.32
24.38
14.27
14.15
220
210
200
190
180
170
160
150
140
130
120
110
100
90
80
70
60
50
40
30
20
10
0
-10
-20
f1 (ppm)

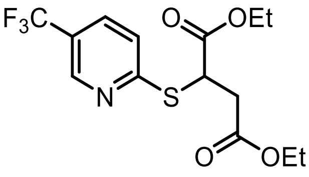

diethyl 2-((5-(trifluoromethyl)pyridin-2-yl)thio)succinate (*pre*-5c)

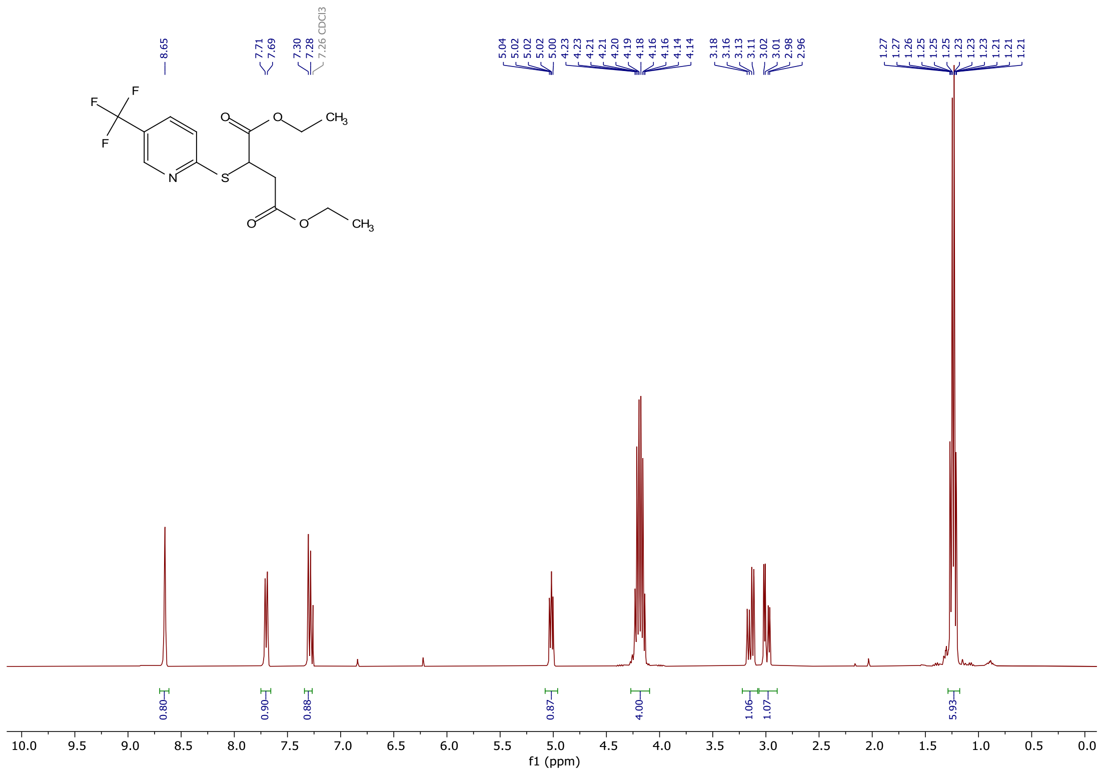

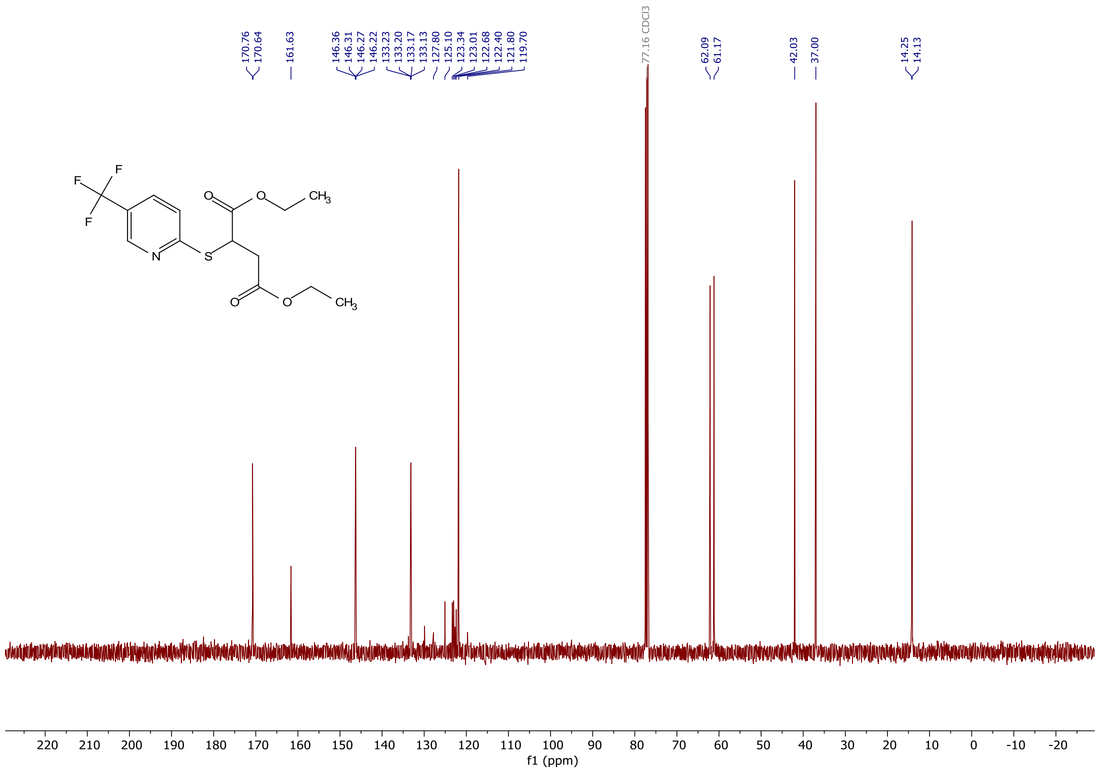

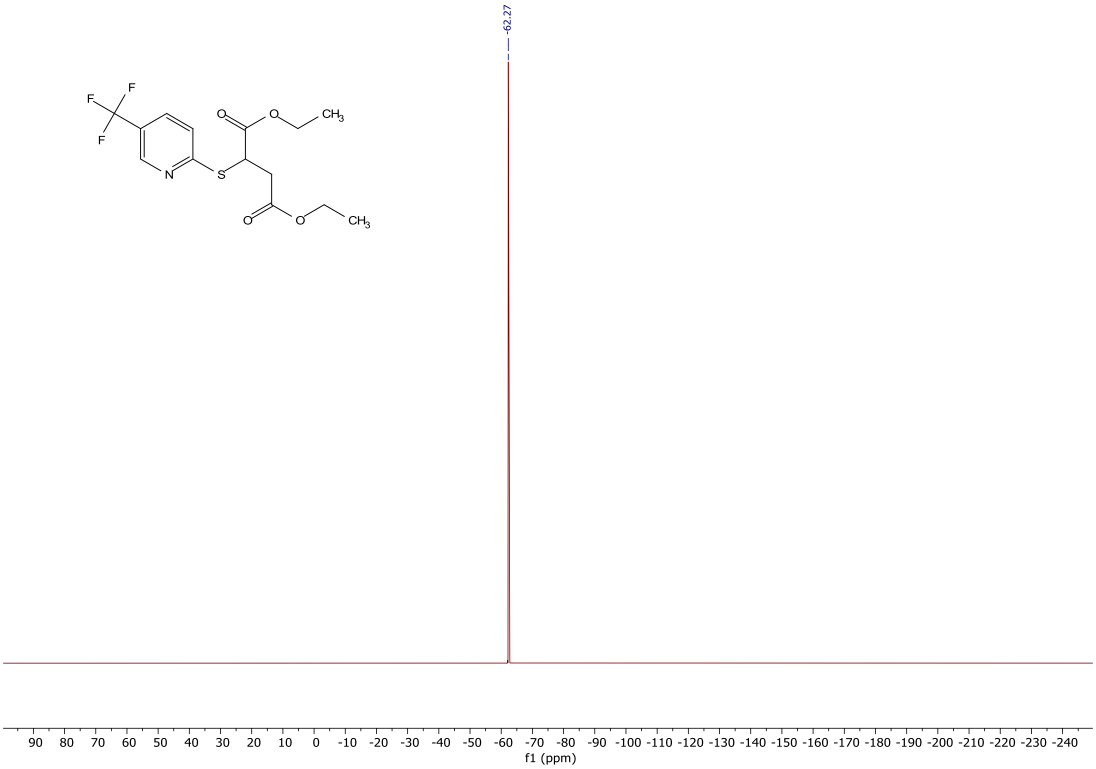

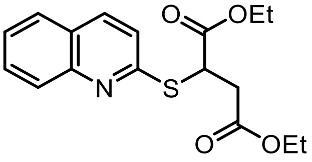

diethyl 2-(quinolin-2-ylthio)succinate (*pre*-5d)

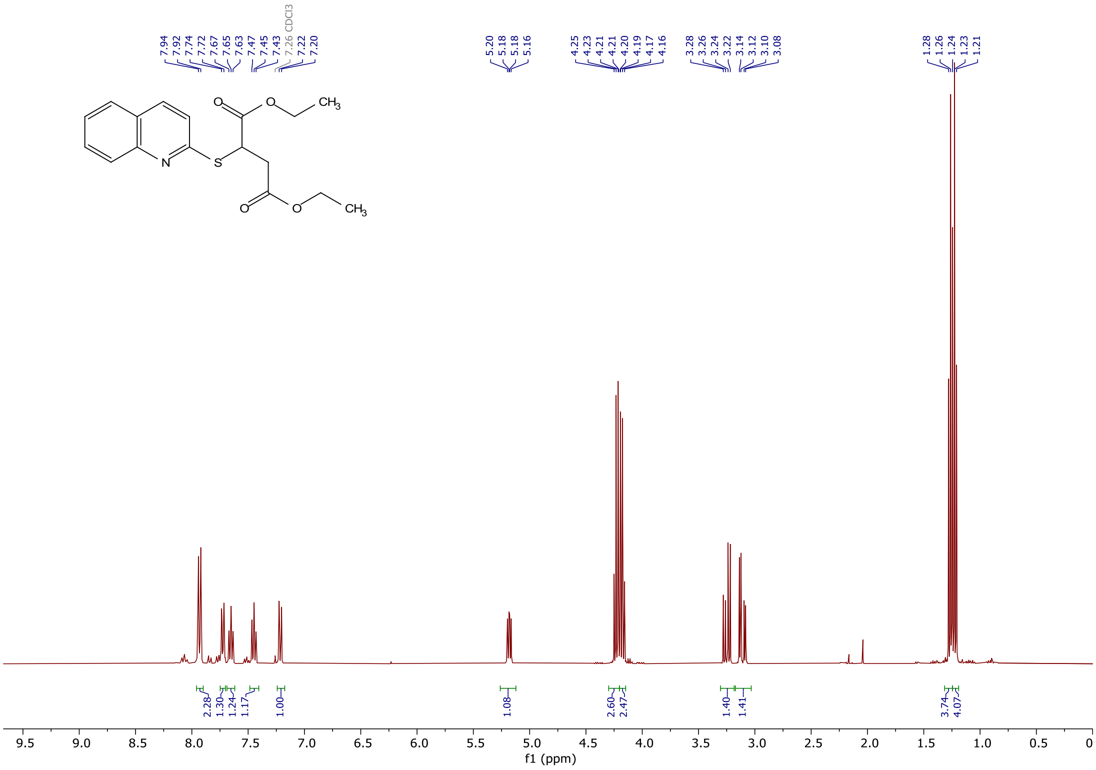

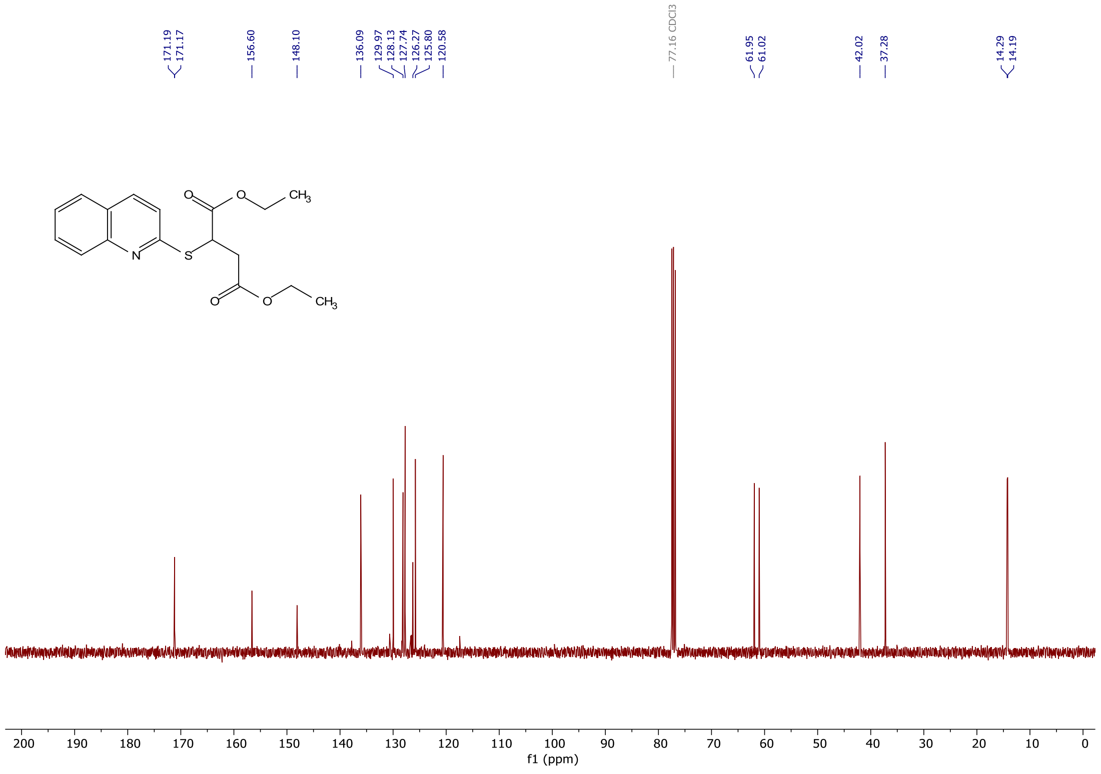

171.19
171.17
156.60
148.10
136.09
129.97
128.13
127.74
126.27
125.80
120.58
77.16 CDCl3
61.95
61.02
42.02
37.28
14.29
14.19
N
S
O
O
O
O
CH3
CH3
200
190
180
170
160
150
140
130
120
110
100
90
80
70
60
50
40
30
20
10
0
f1 (ppm)

4.2 NMR Spectra of heteroaromatic sulfones – *Base-labile sulfinates*

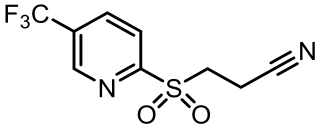

3-((5-(trifluoromethyl)pyridin-2-yl)sulfonyl)propanenitrile (1a)

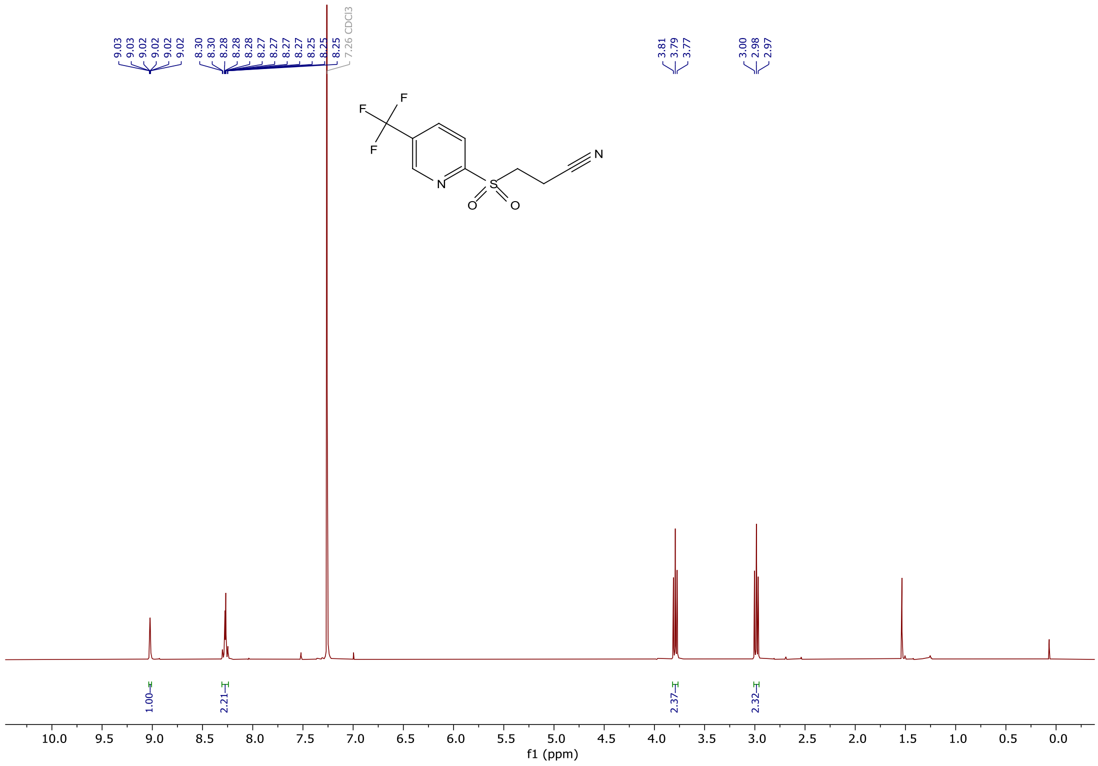

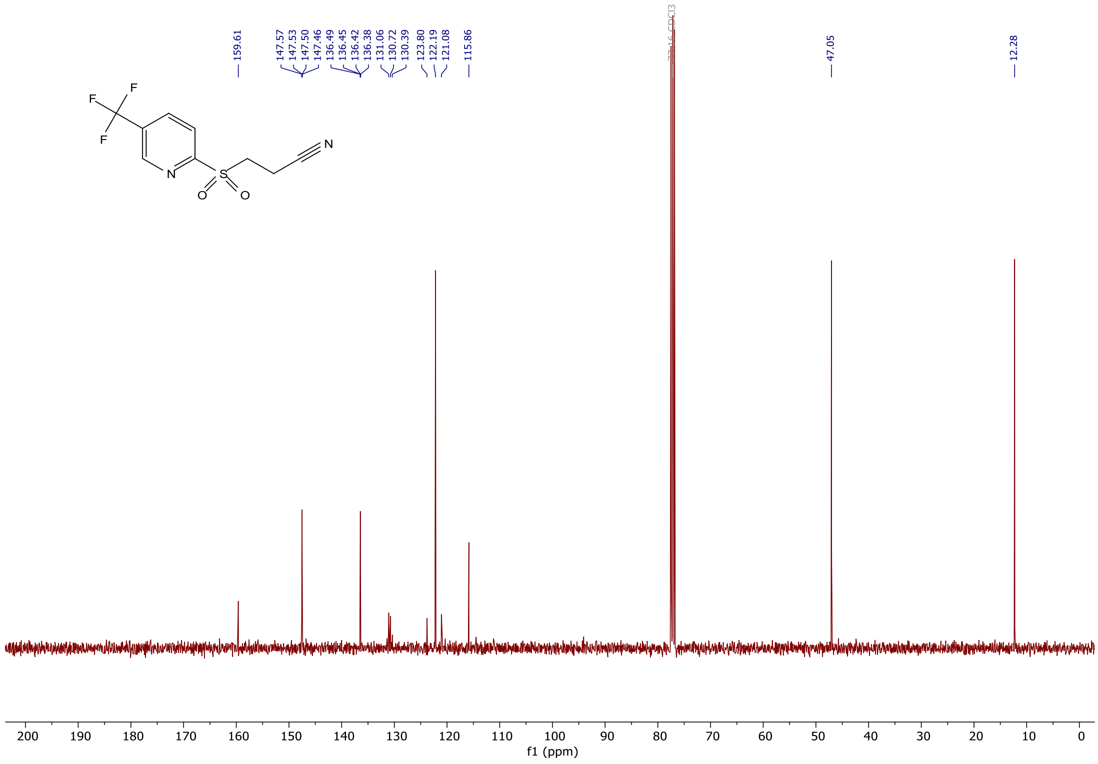

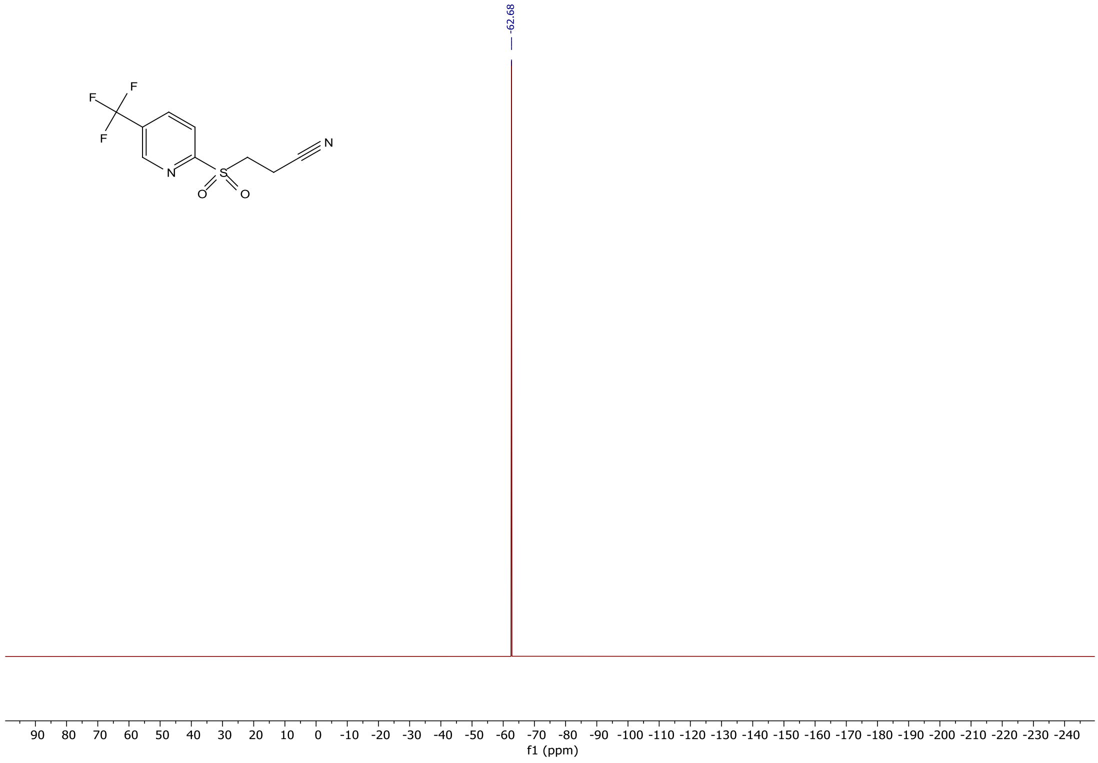

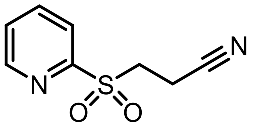

3-(pyridin-2-ylsulfonyl)propanenitrile (1b)

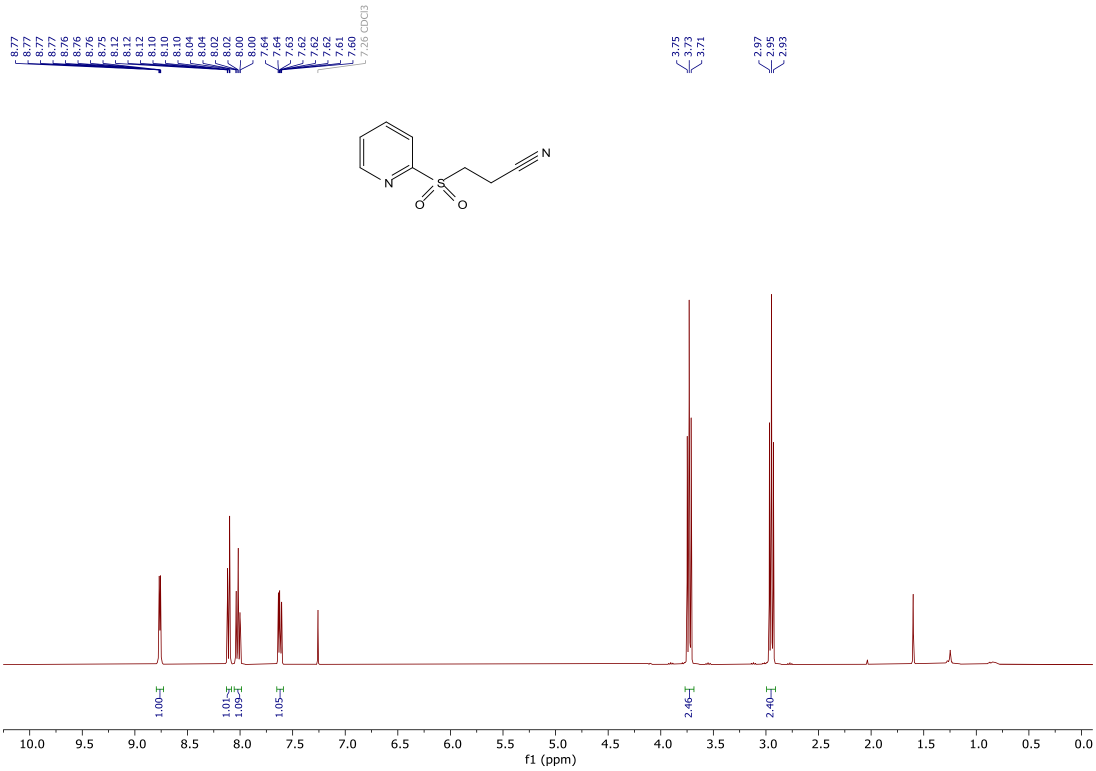

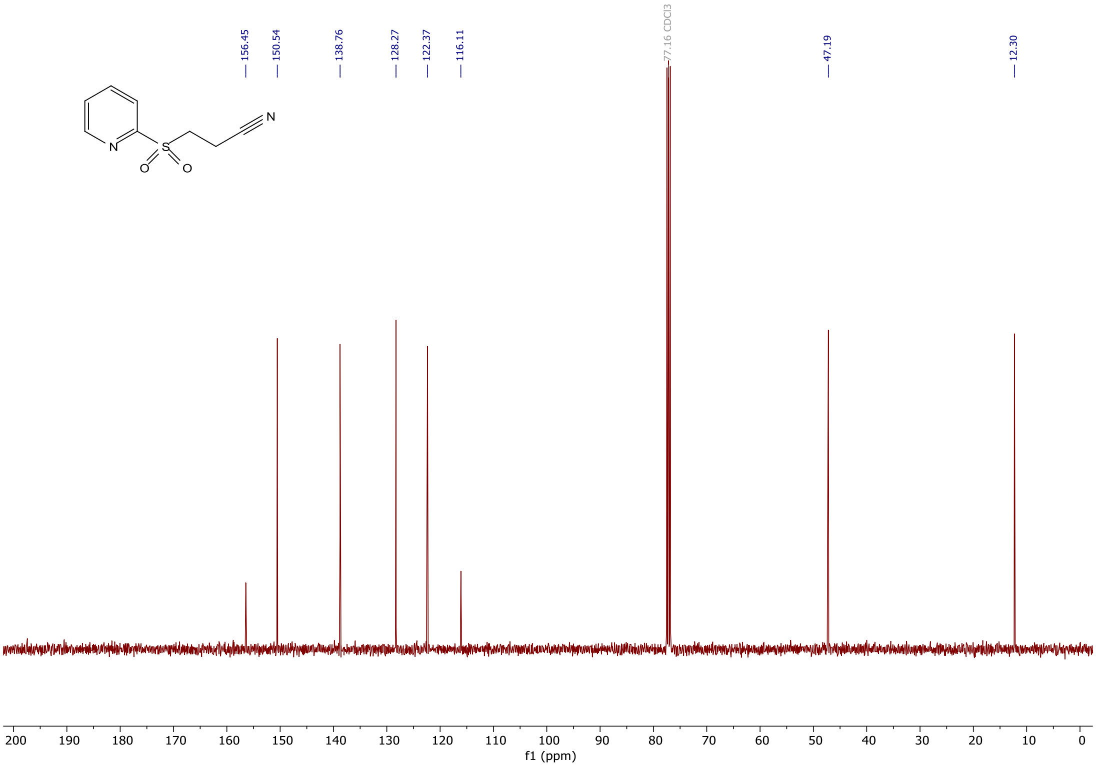

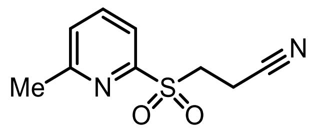

3-((6-methylpyridin-2-yl)sulfonyl)propanenitrile (1c)

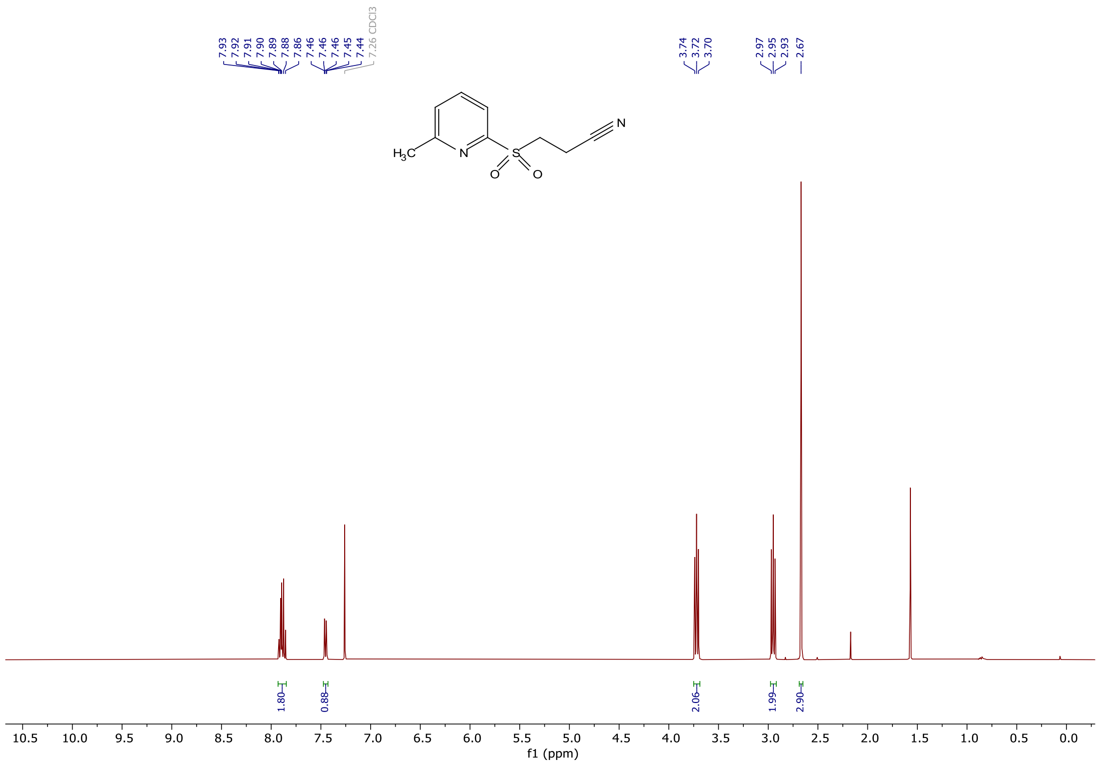

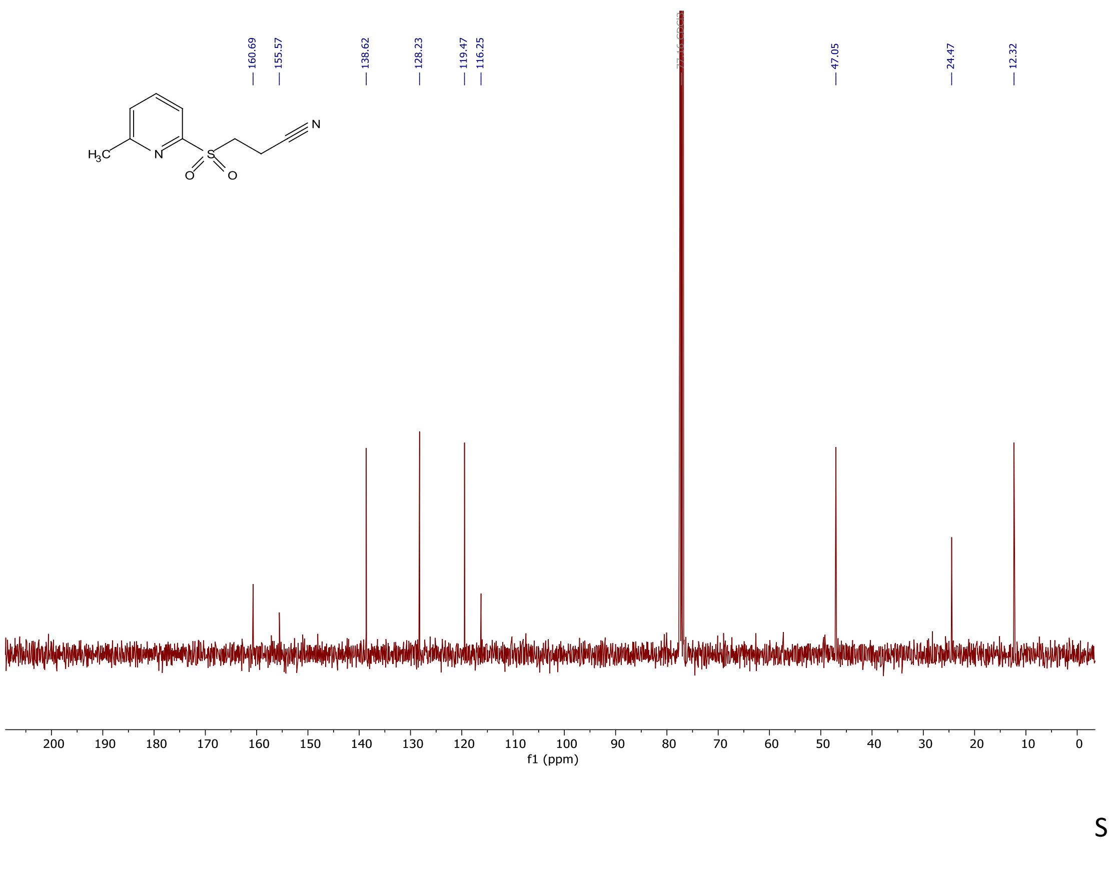

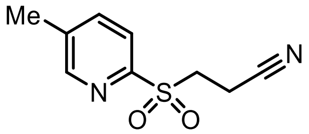

3-((5-methylpyridin-2-yl)sulfonyl)propanenitrile (1d)

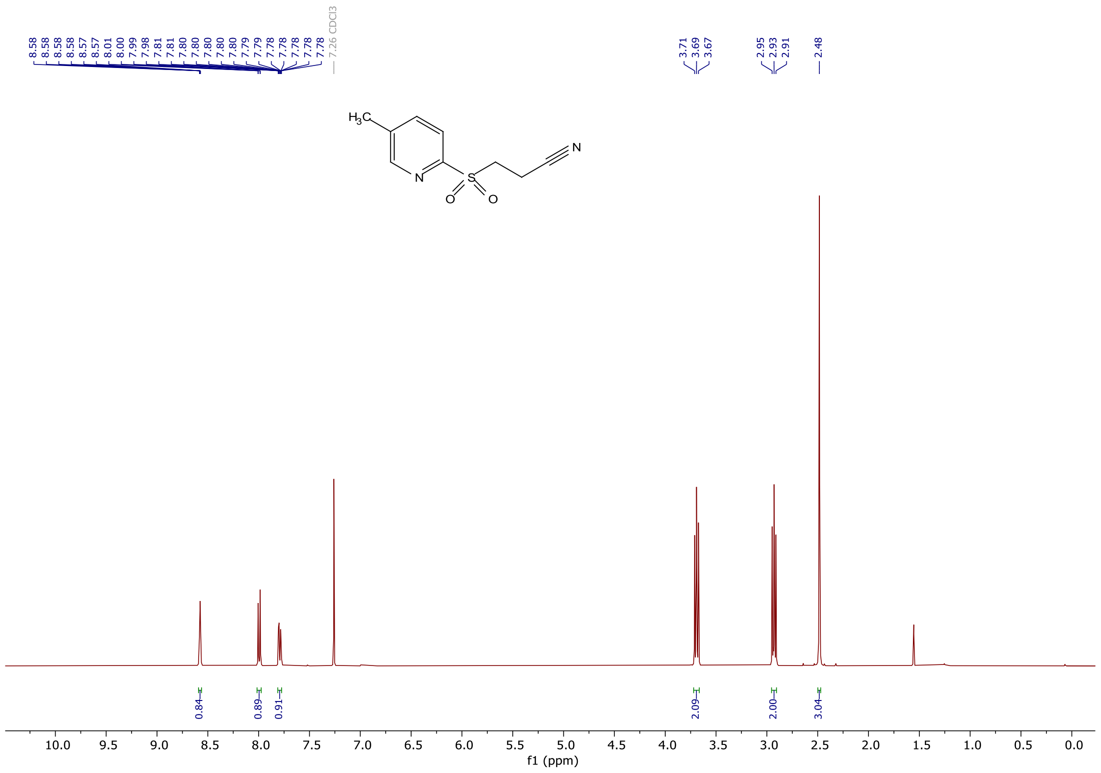

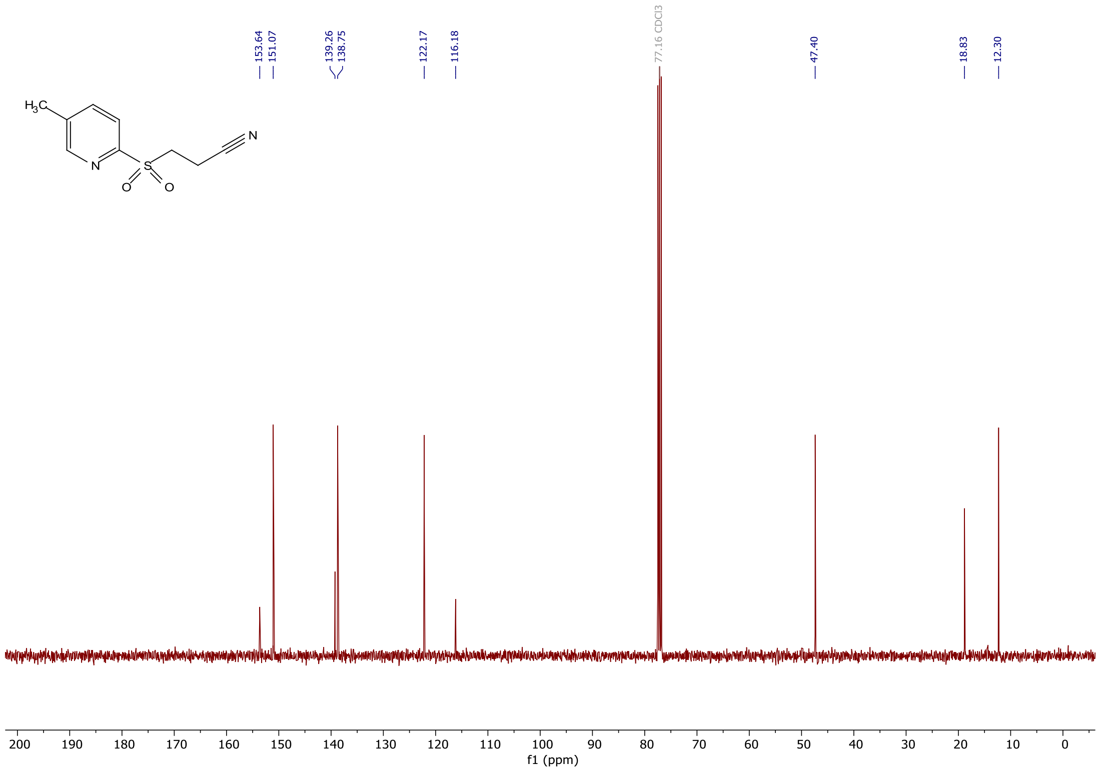

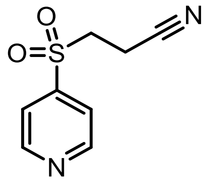

3-(pyridin-4-ylsulfonyl)propanenitrile (1e)

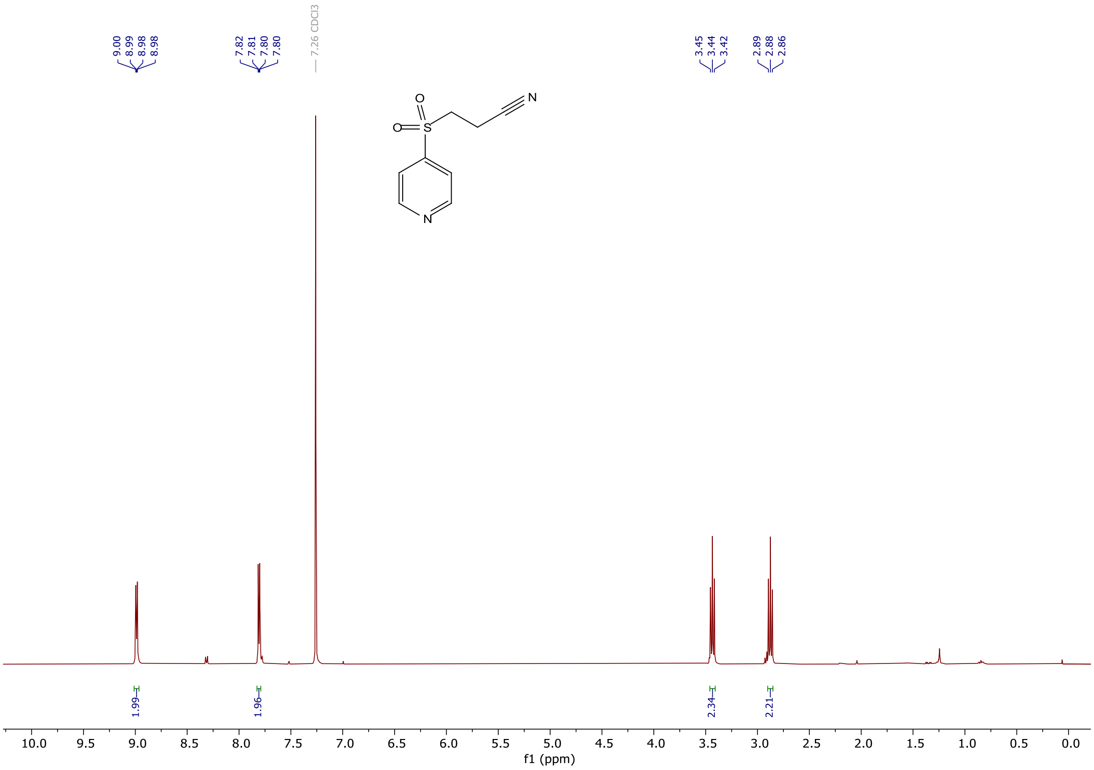

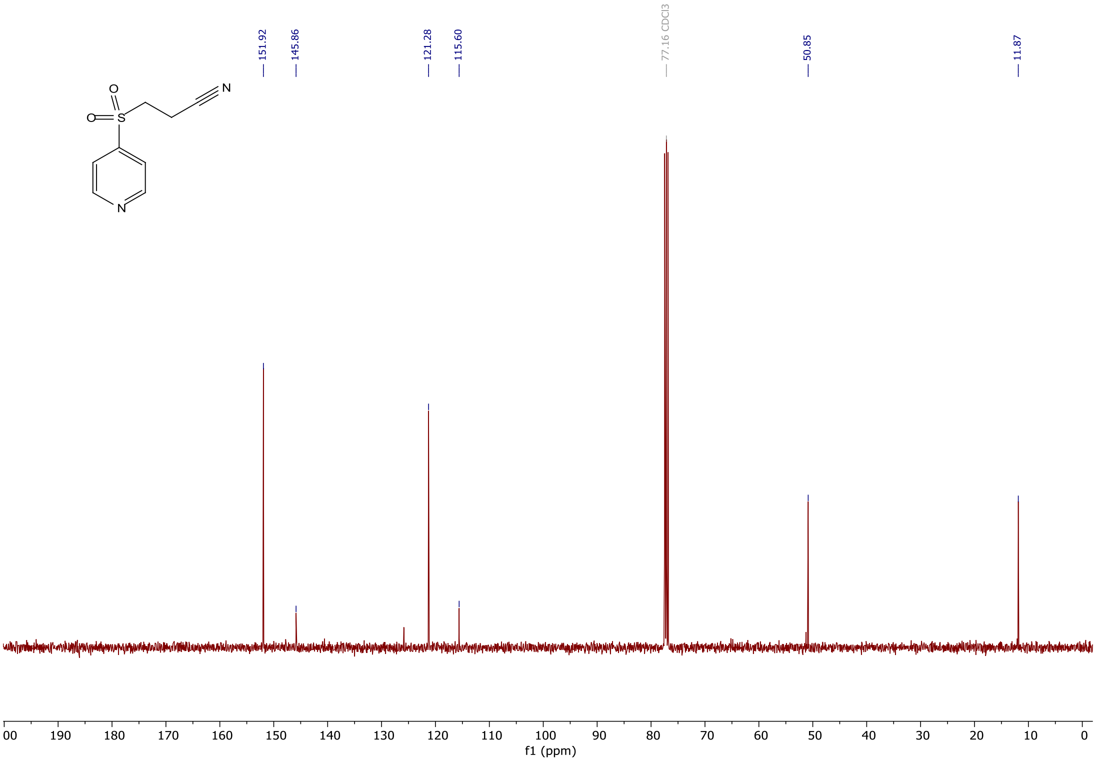

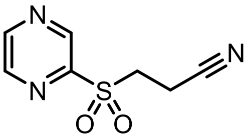

3-(pyrazin-2-ylsulfonyl)propanenitrile (1f)

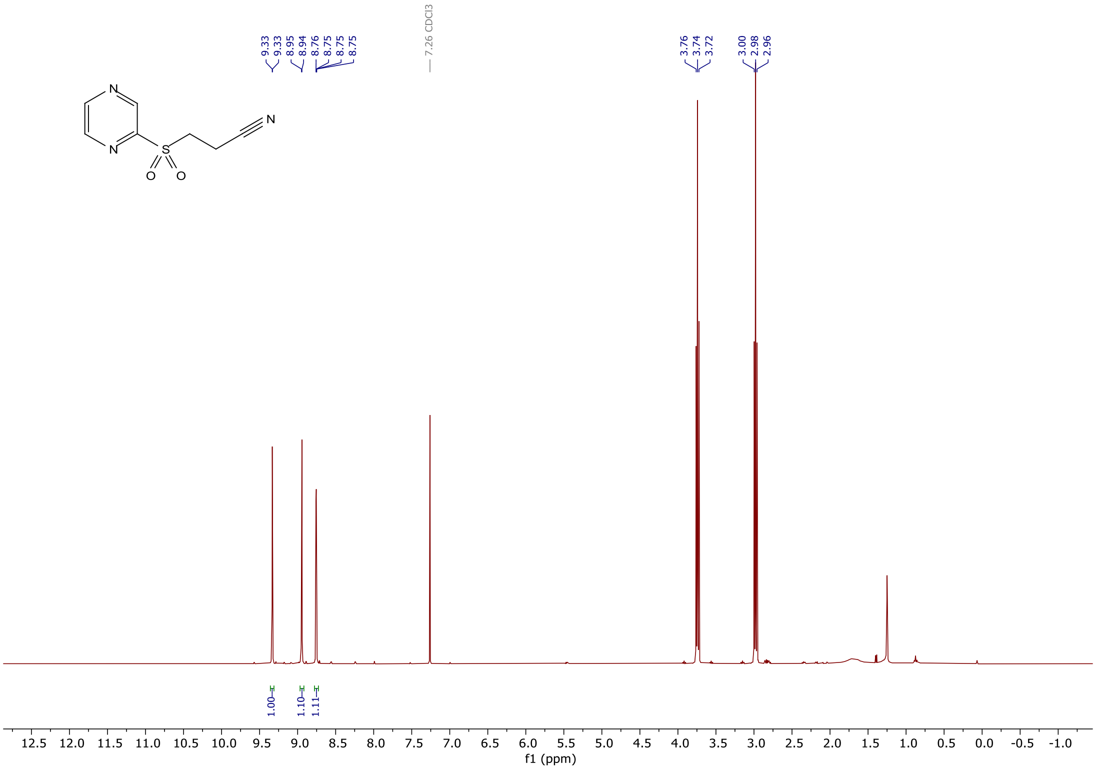

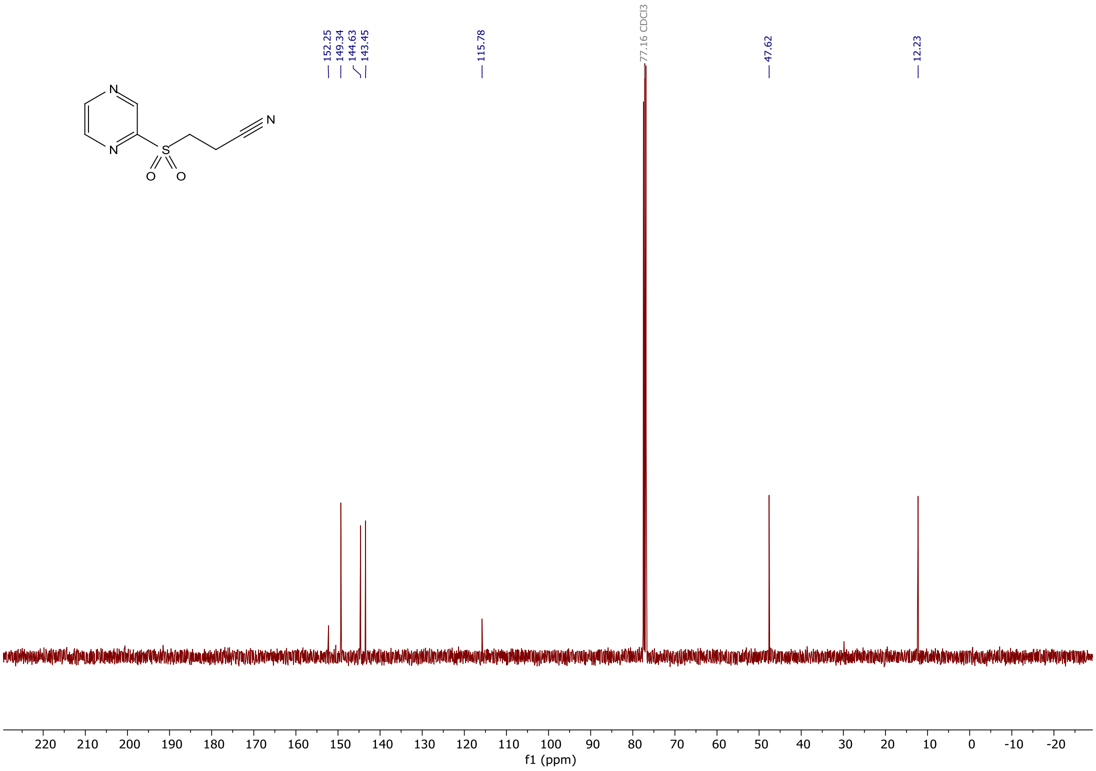

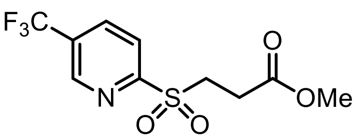

methyl 3-((5-(trifluoromethyl)pyridin-2-yl)sulfonyl)propanoate (4a)

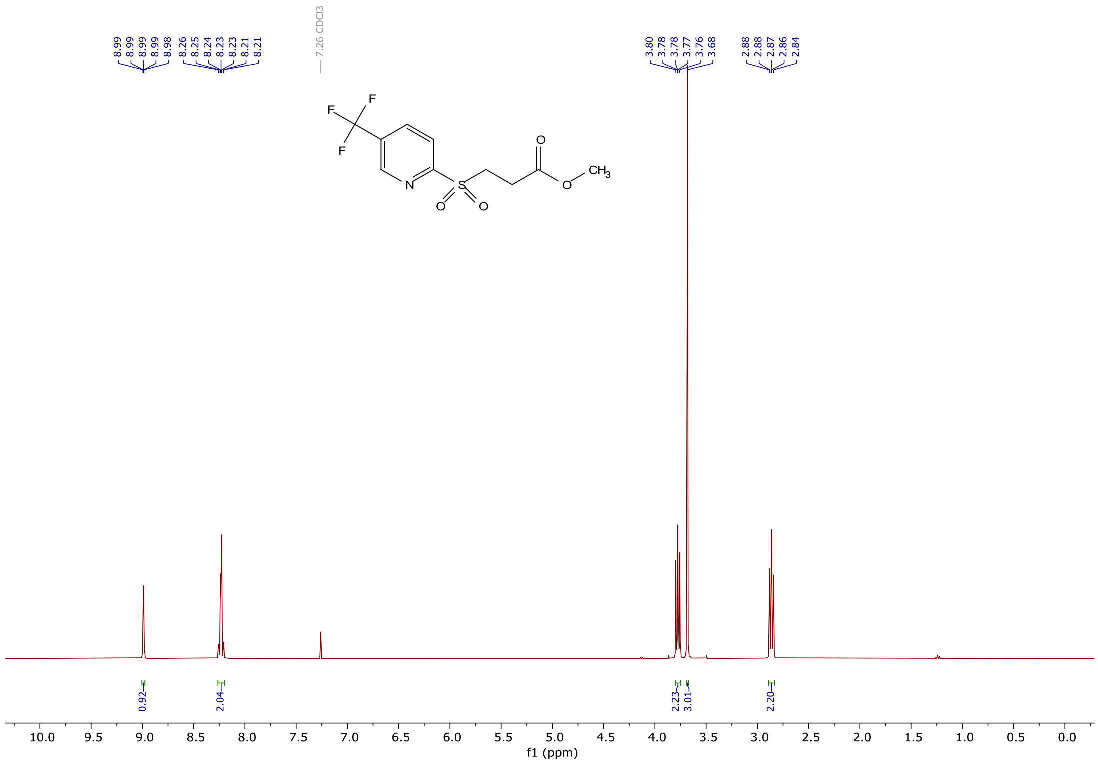

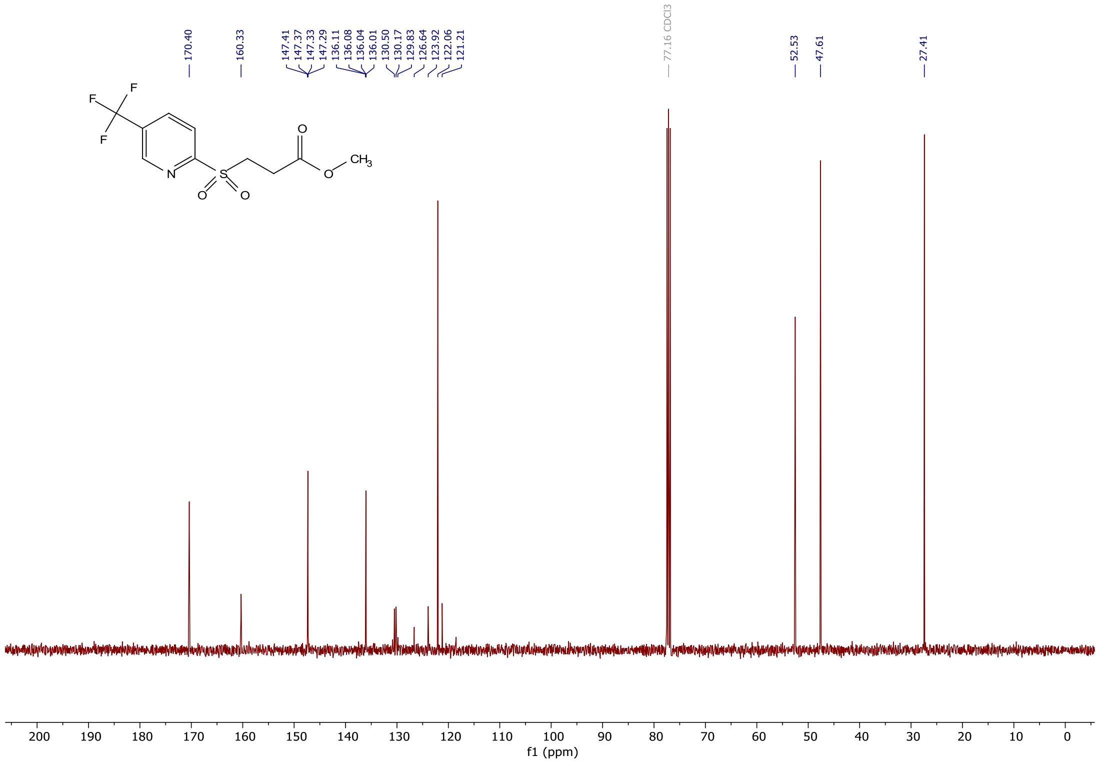

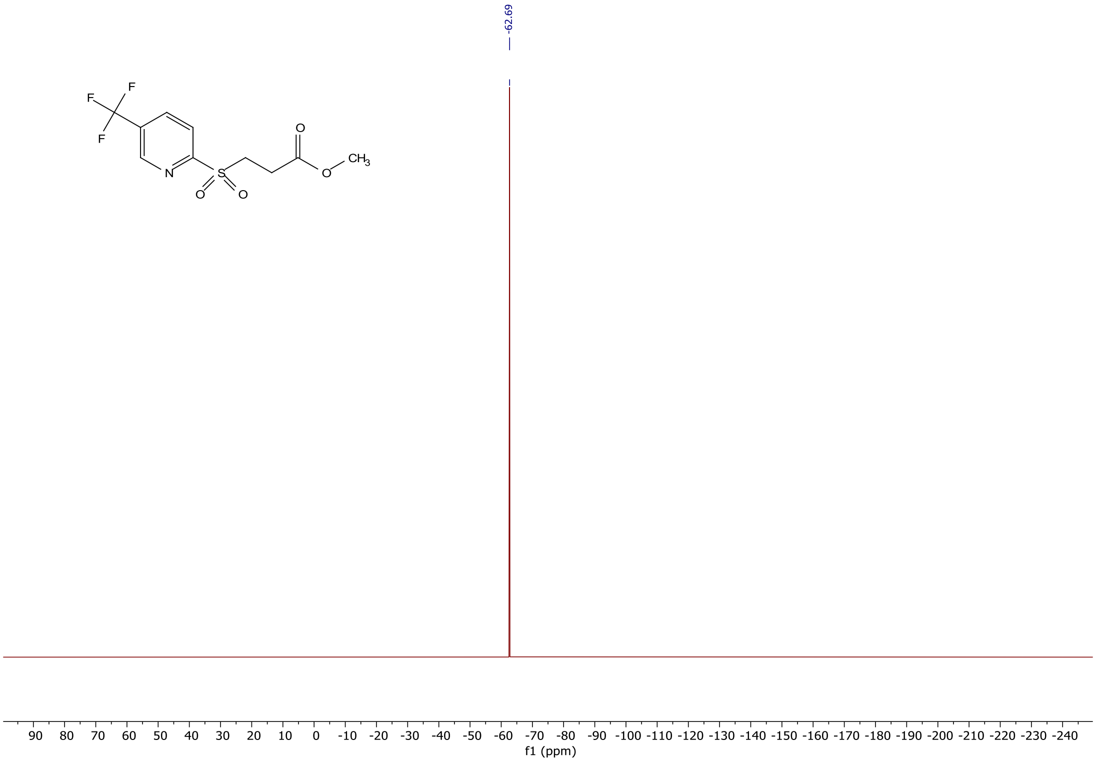

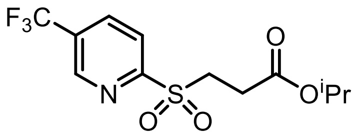

Iso-propyl 3-((5-(trifluoromethyl)pyridin-2-yl)sulfonyl)propanoate (4a-iPr)

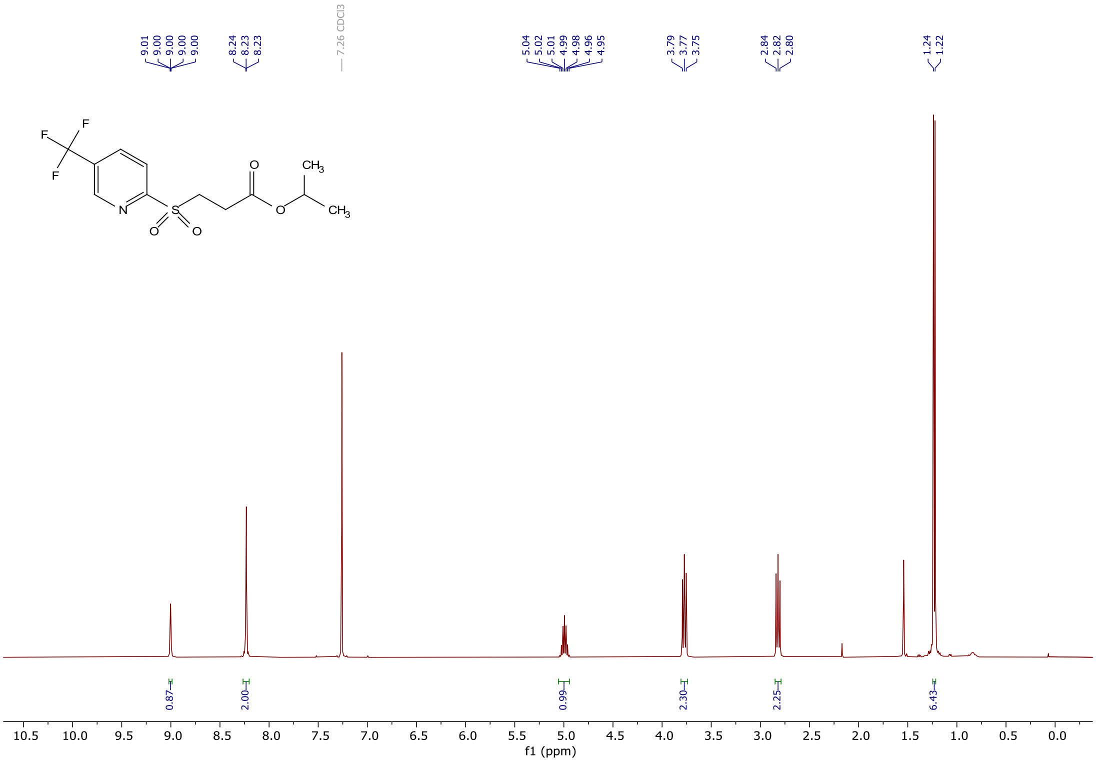

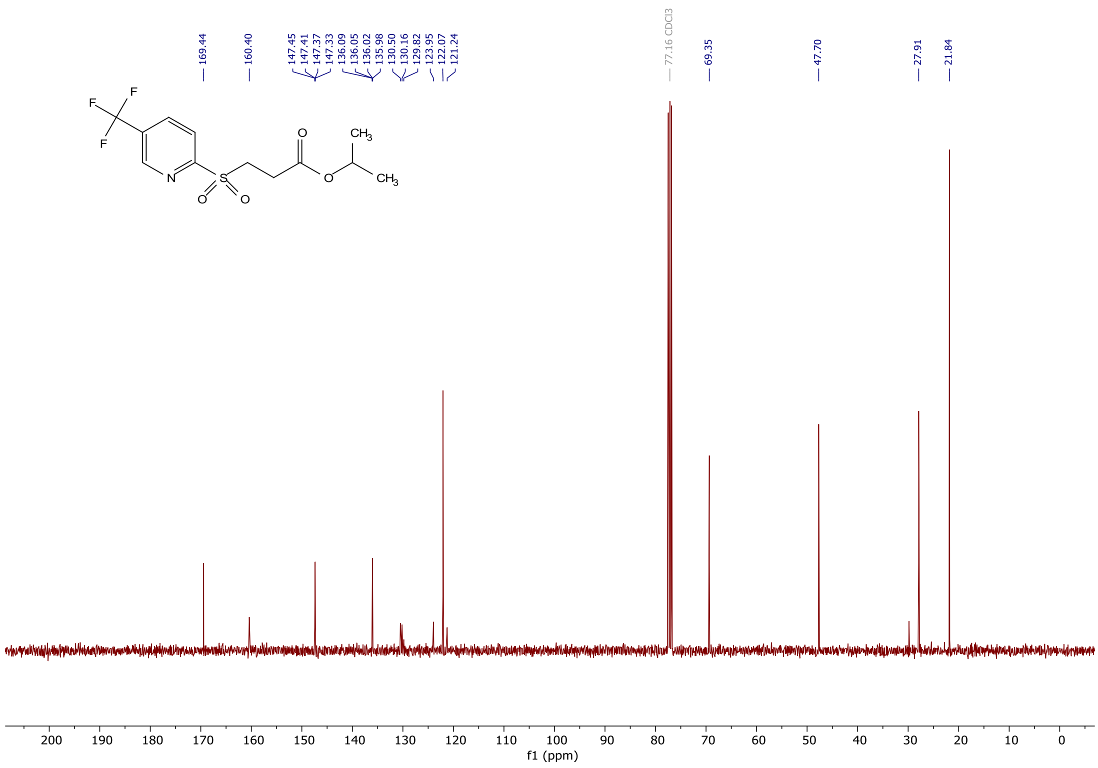
169.44
160.40
147.45
147.41
147.37
147.33
136.09
136.05
136.02
135.98
130.50
130.16
129.82
123.95
122.07
121.24
77.16 CDCl3
69.35
47.70
27.91
21.84
f1 (ppm)

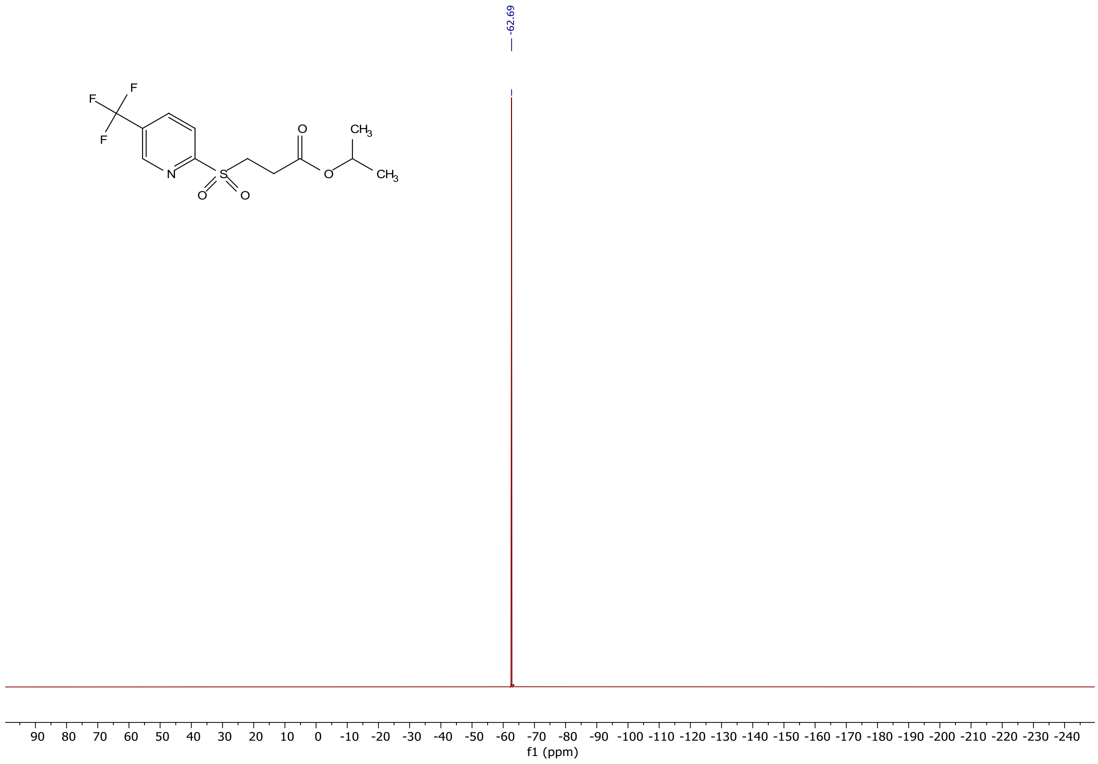
-62.69
f1 (ppm)

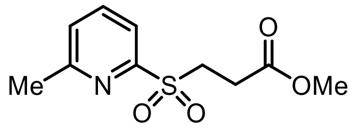

Methyl 3-((6-methylpyridin-2-yl)sulfonyl)propanoate (4b)

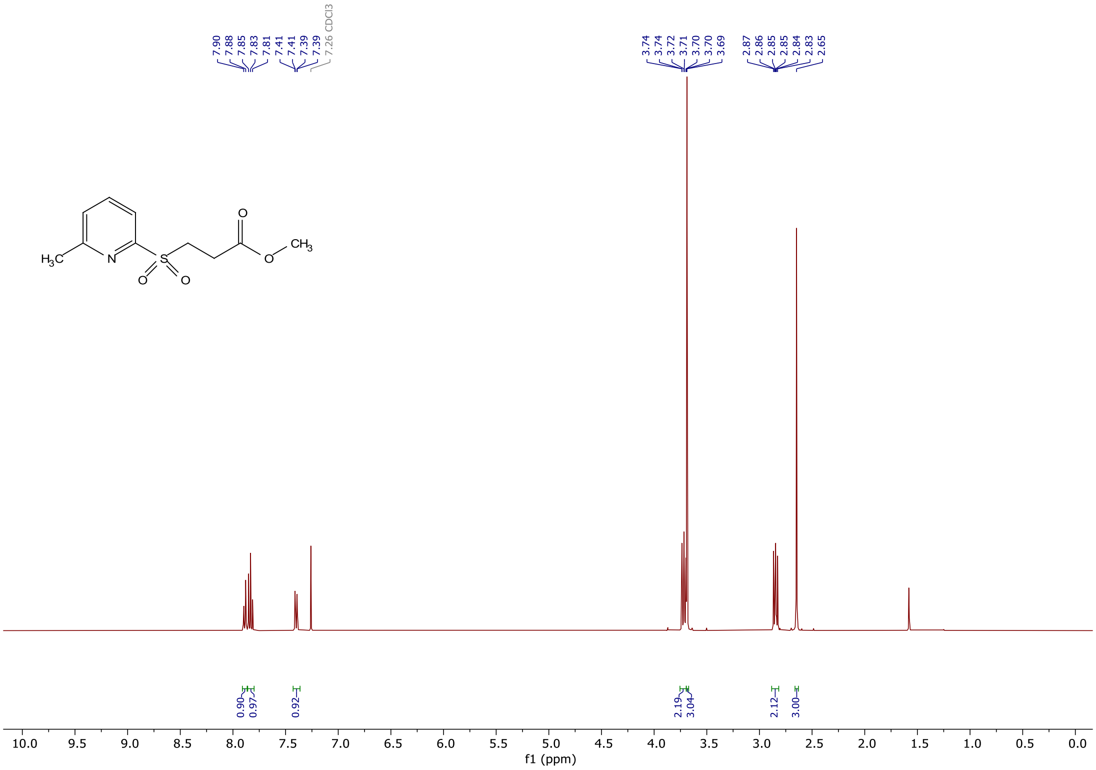

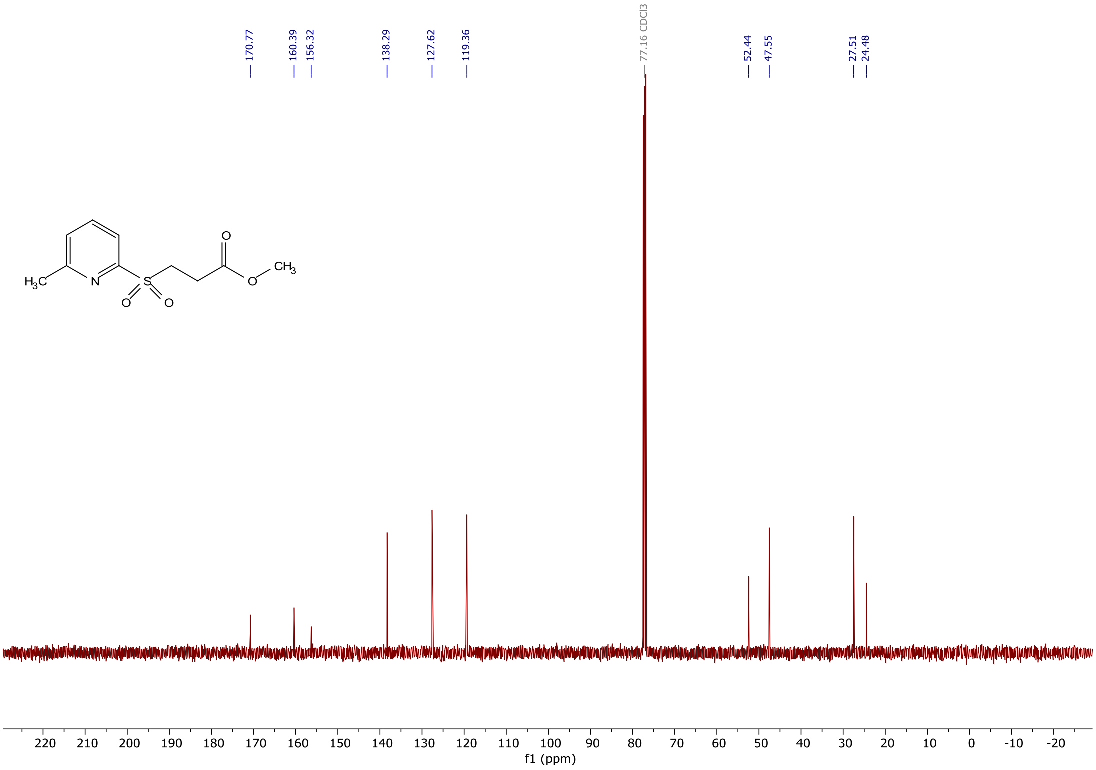

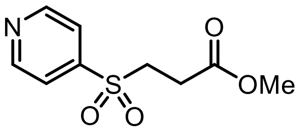

methyl 3-(pyridin-4-ylsulfonyl)propanoate (4c)

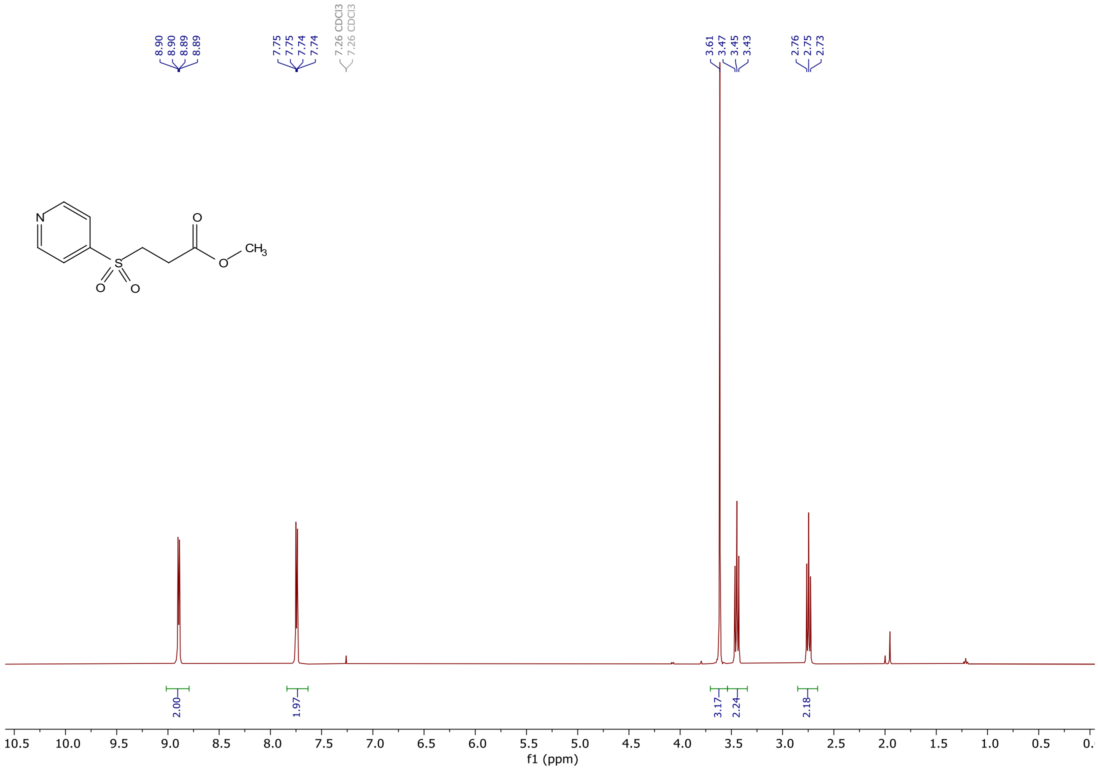

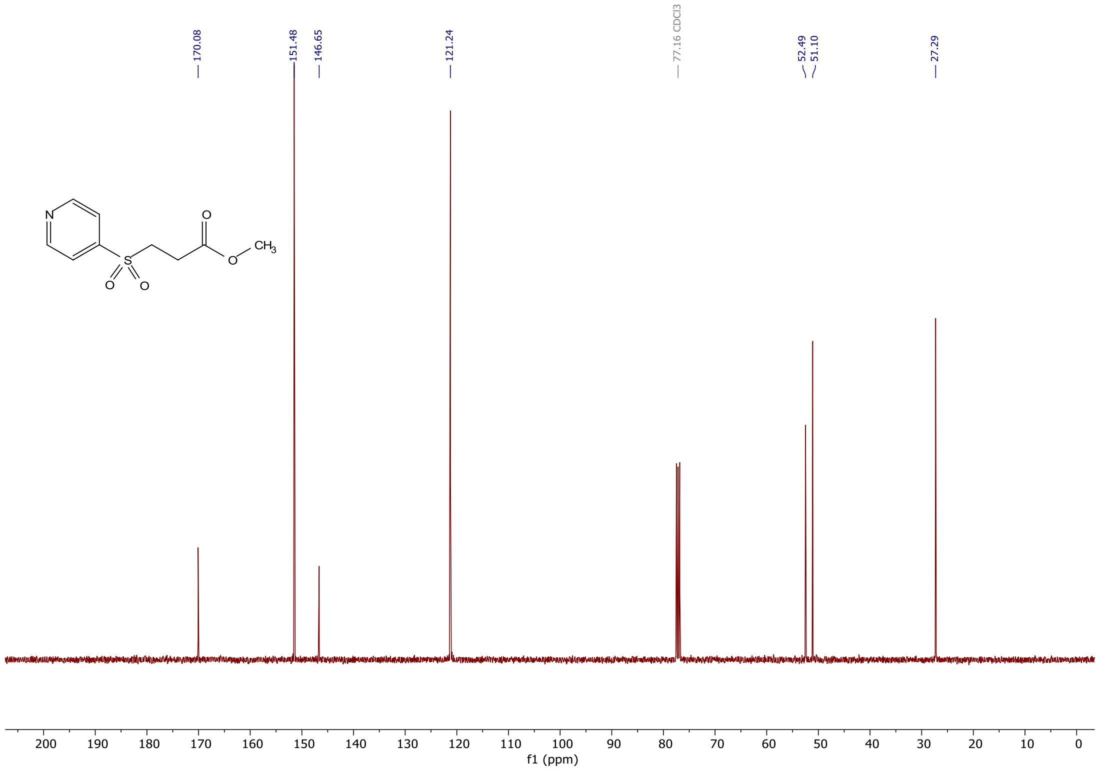

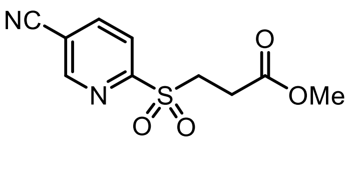

methyl 3-((5-cyanopyridin-2-yl)sulfonyl)propanoate (4d)

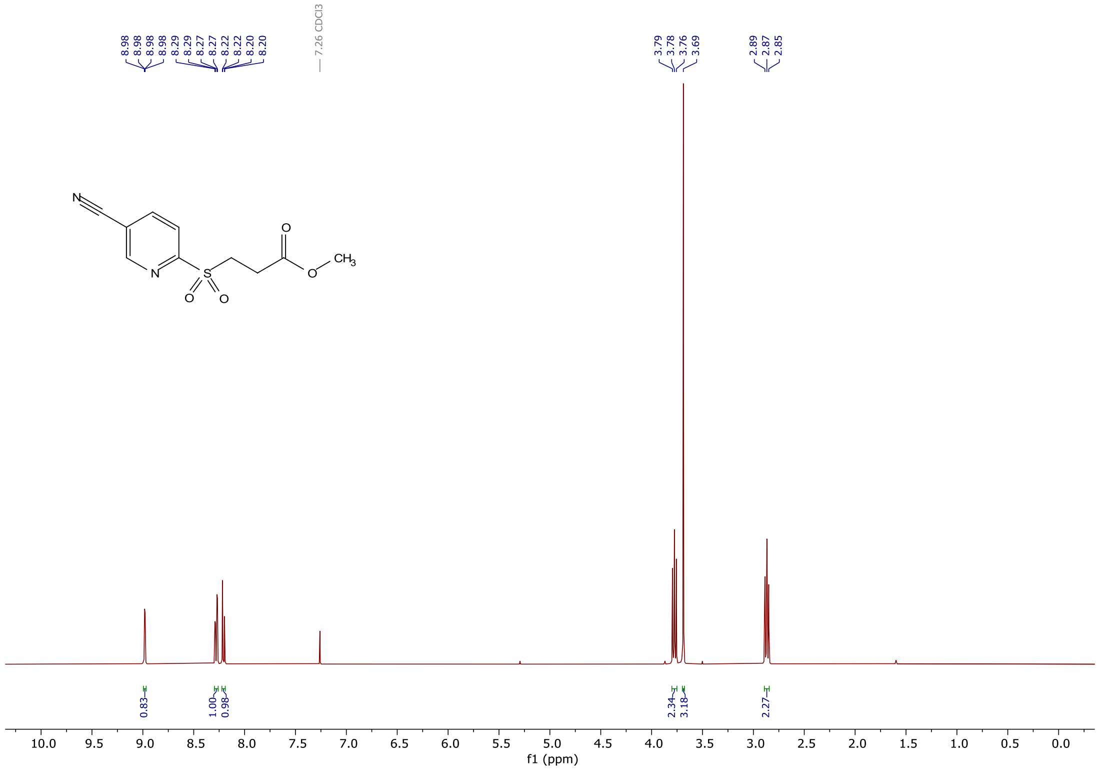

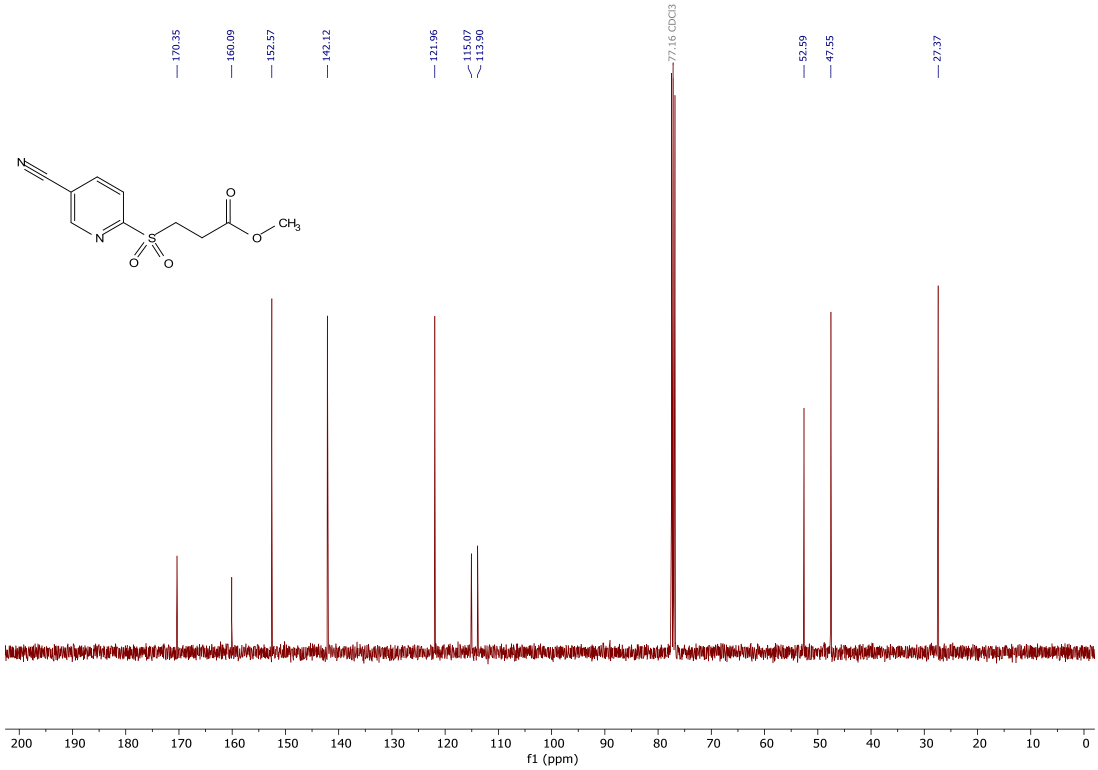

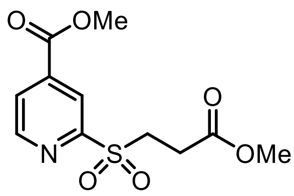

methyl 2-((3-methoxy-3-oxopropyl)sulfonyl)isonicotinate (4e)

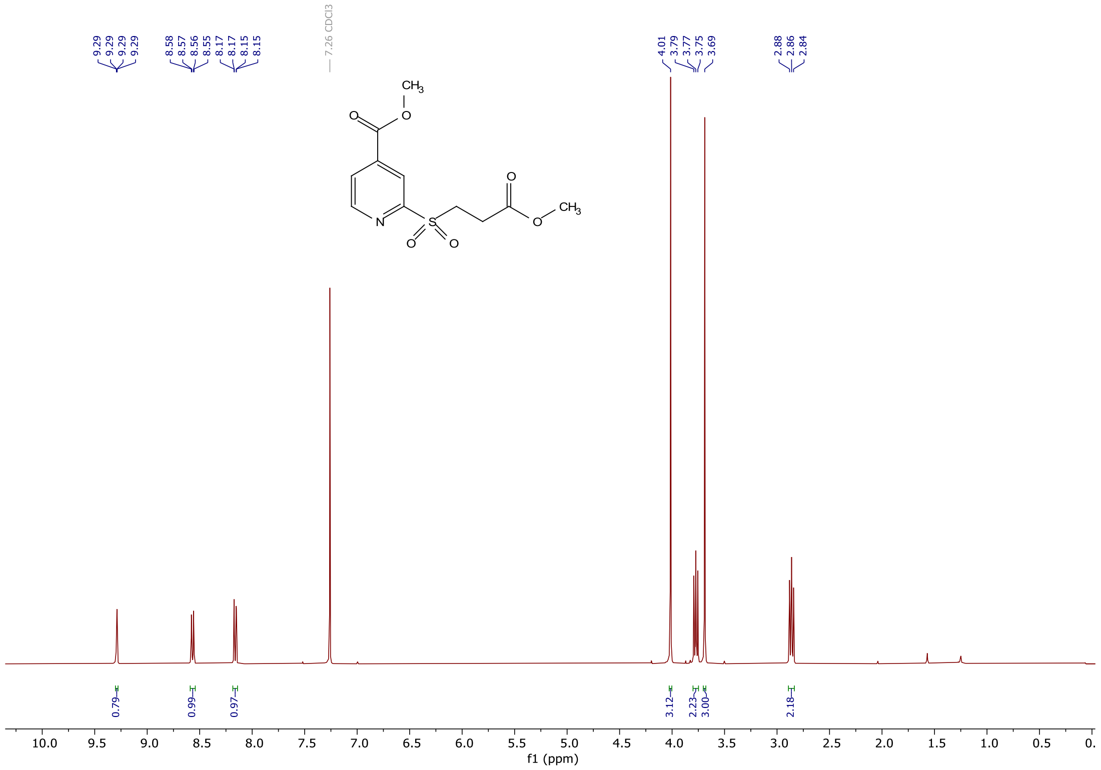

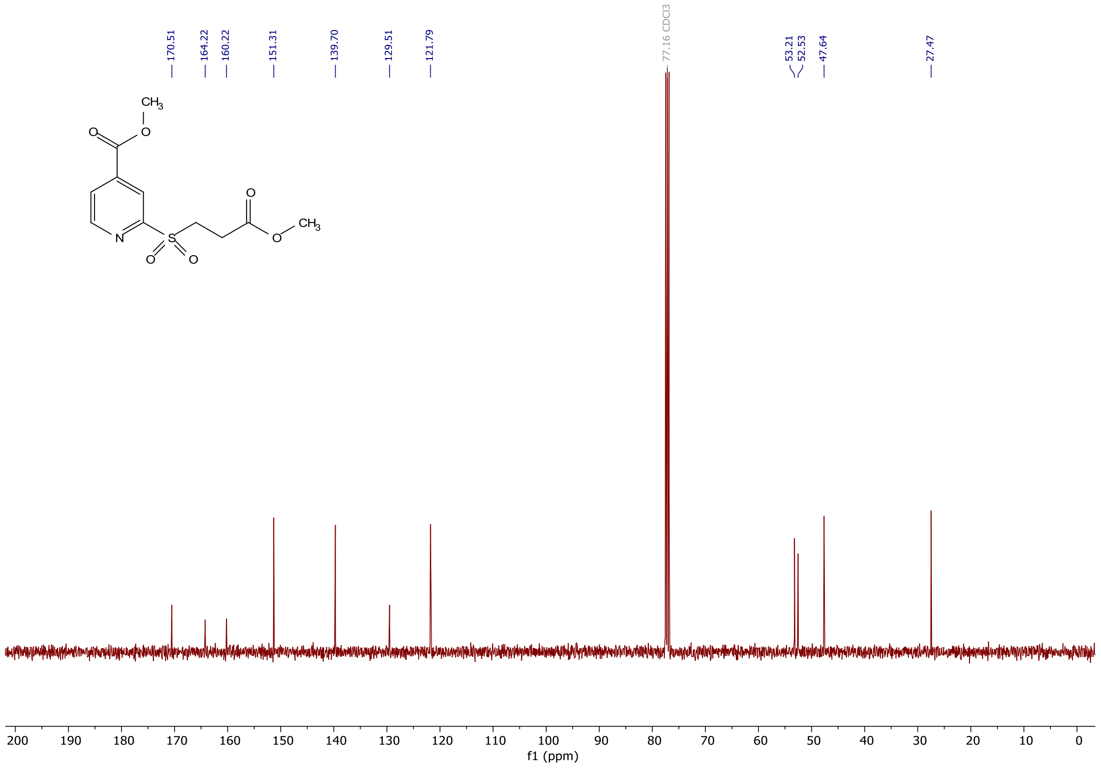

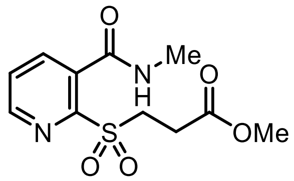

methyl 3-((3-(methylcarbamoyl)pyridin-2-yl)sulfonyl)propanoate (4f)

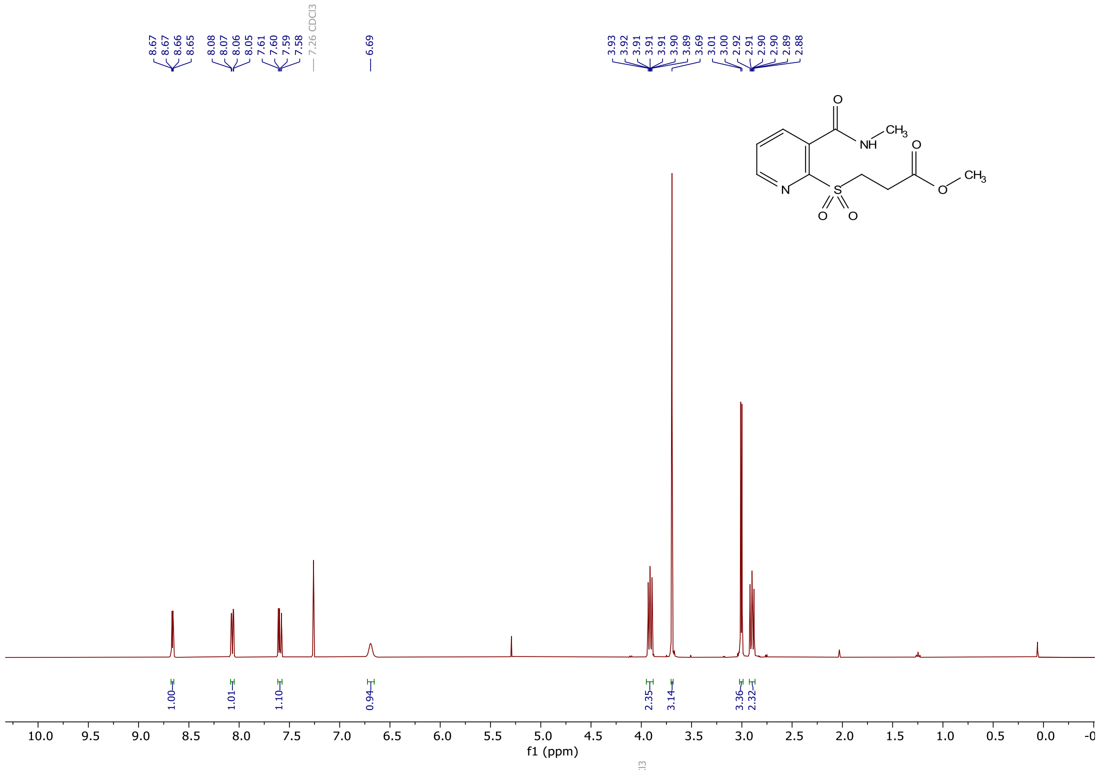

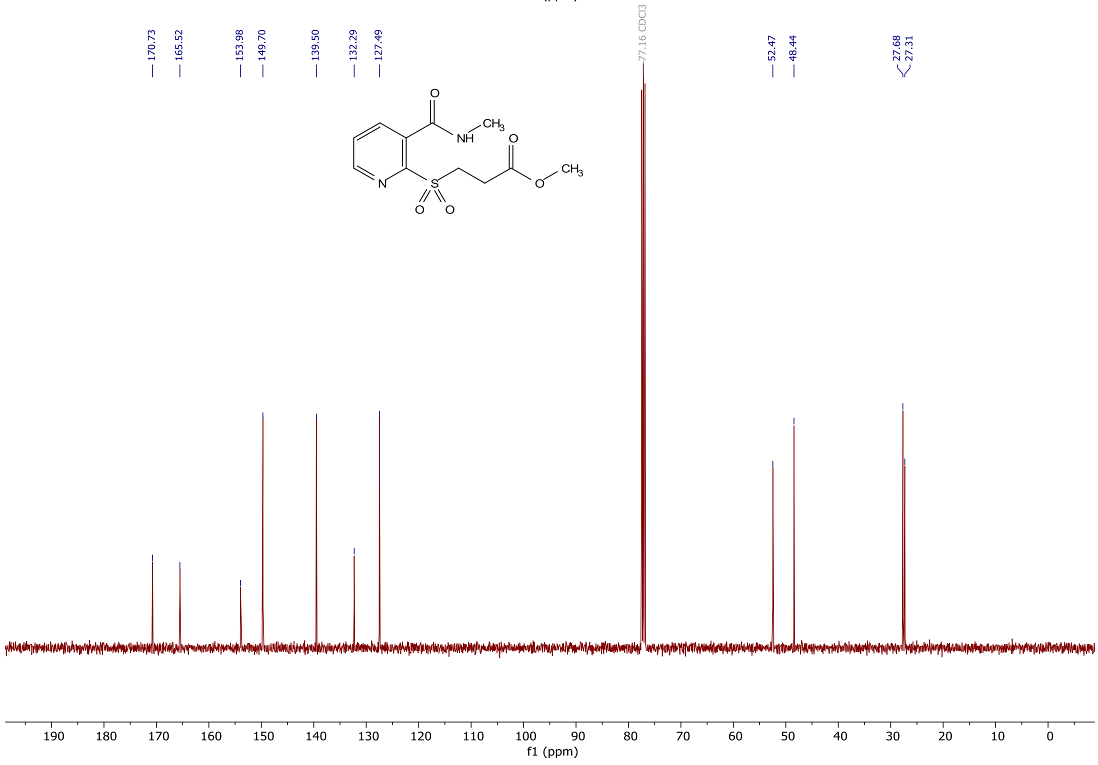

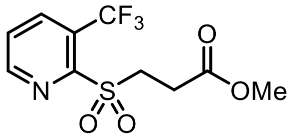

methyl 3-((3-(trifluoromethyl)pyridin-2-yl)sulfonyl)propanoate (4g)

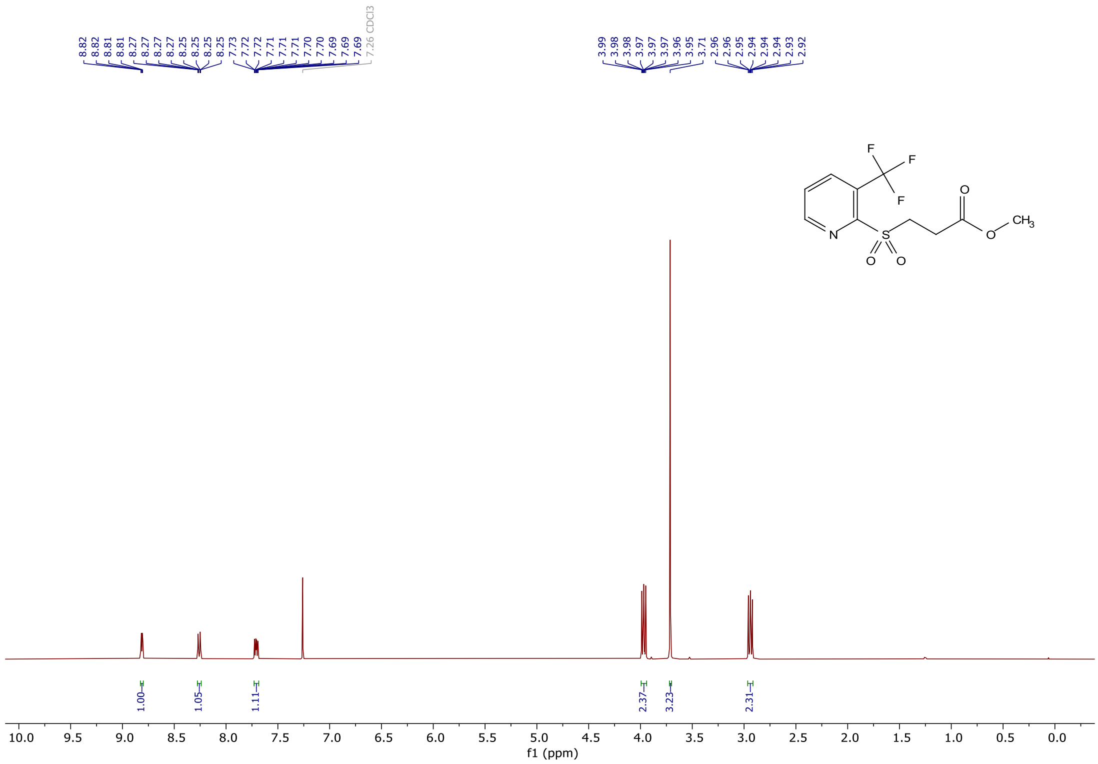

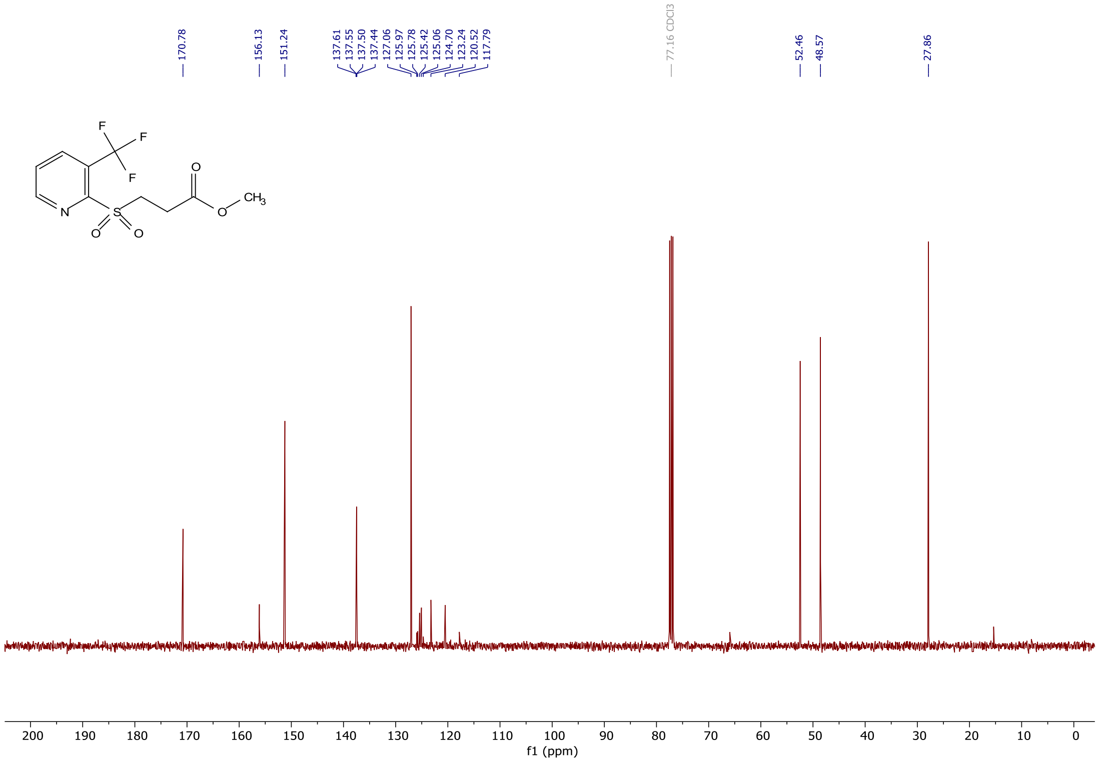

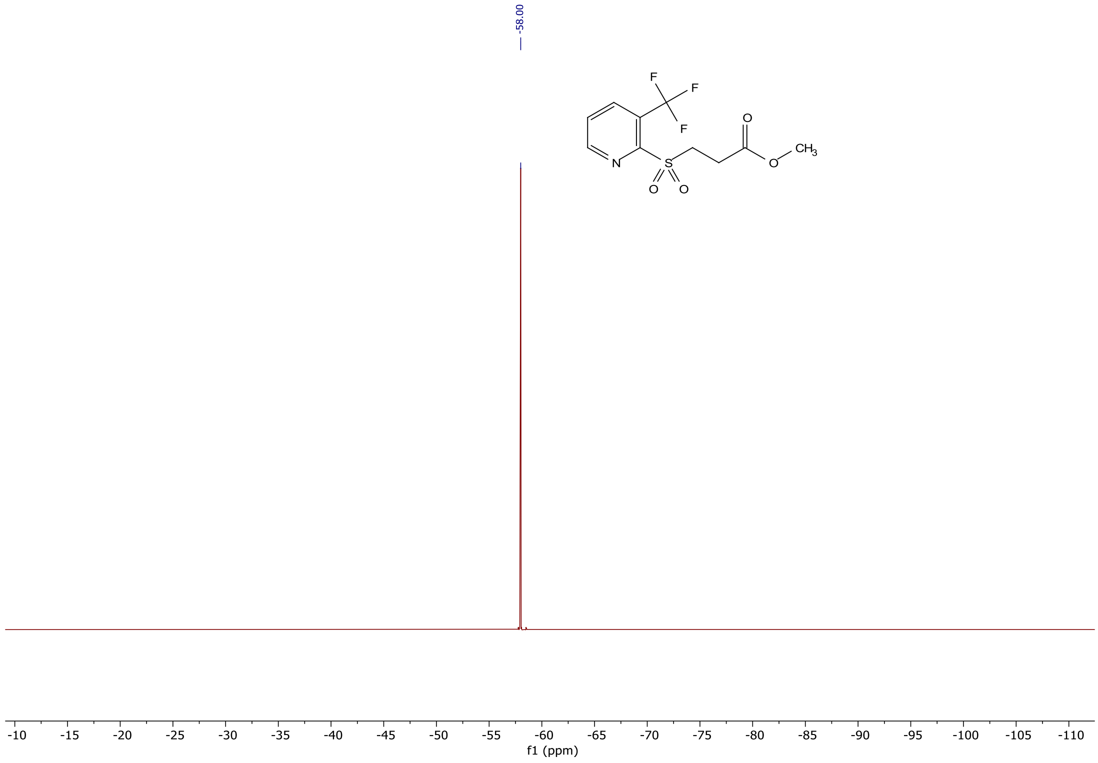

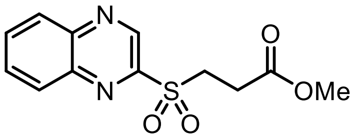

methyl 3-(quinoxalin-2-ylsulfonyl)propanoate (4h)

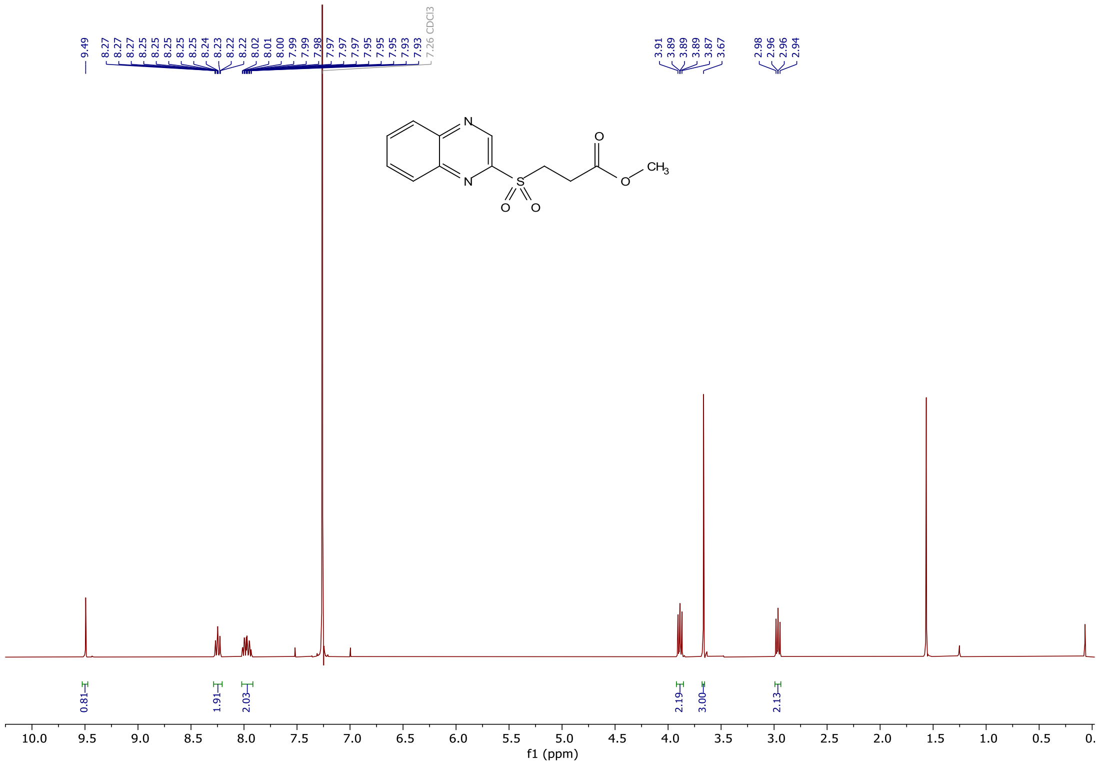

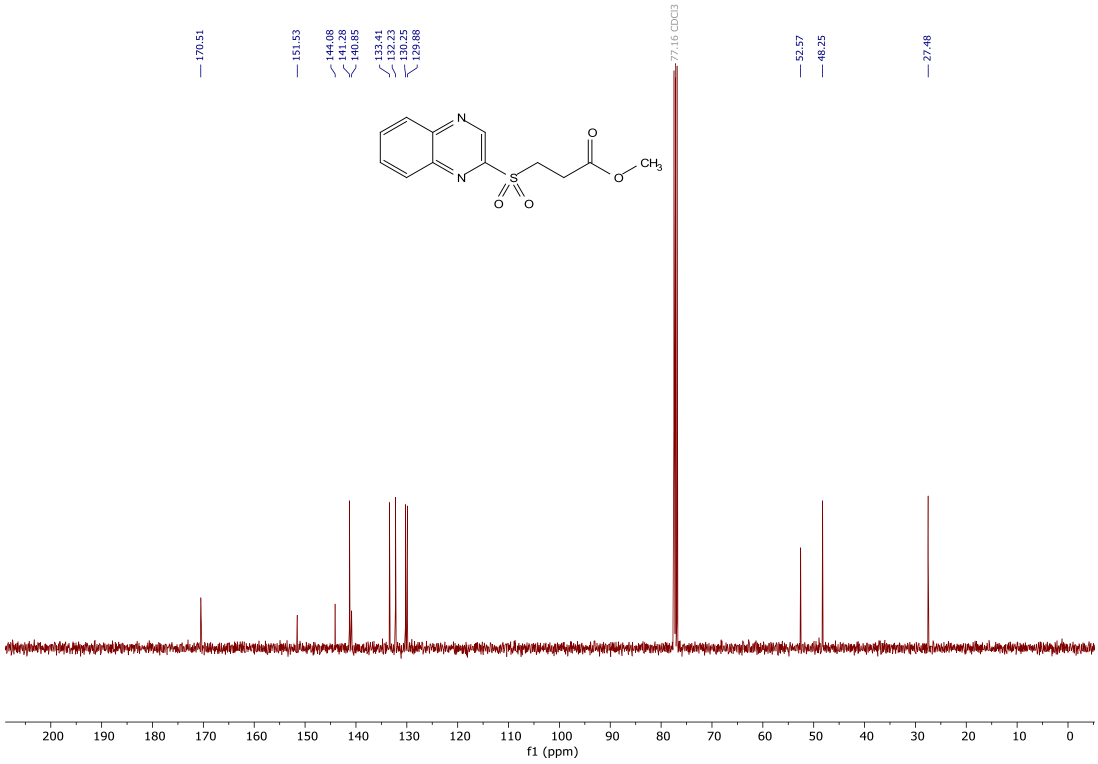

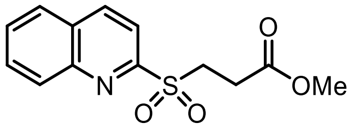

methyl 3-(quinolin-2-ylsulfonyl)propanoate (4i)

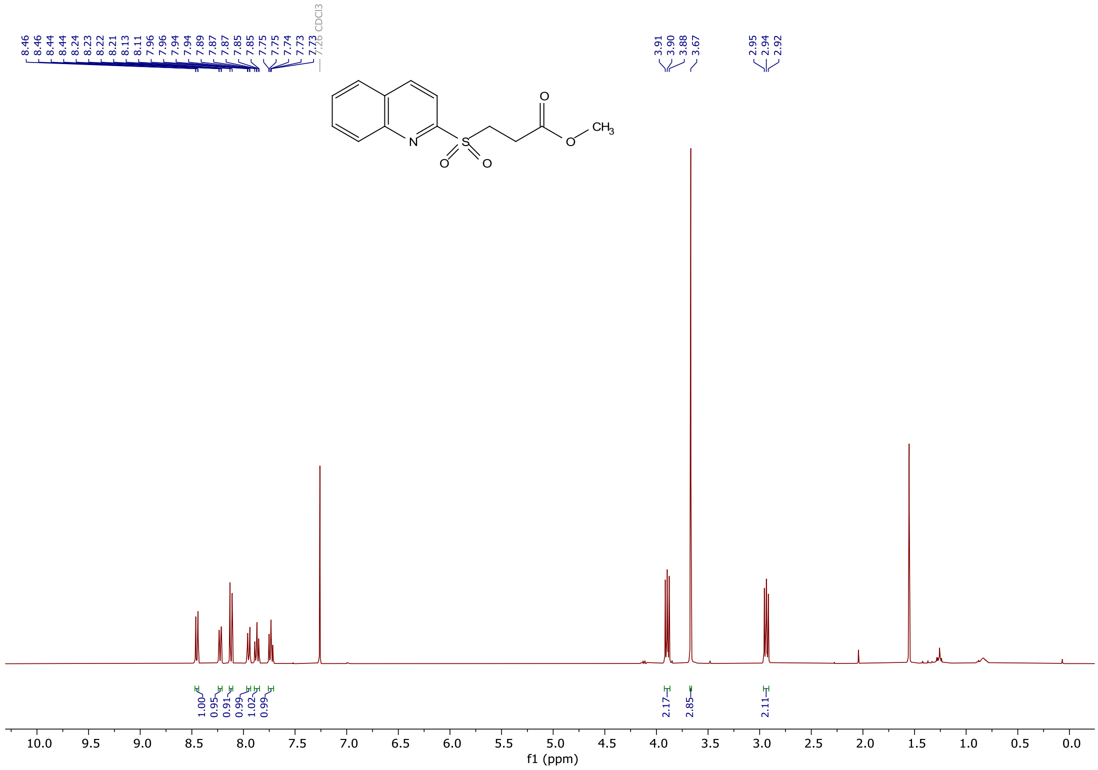

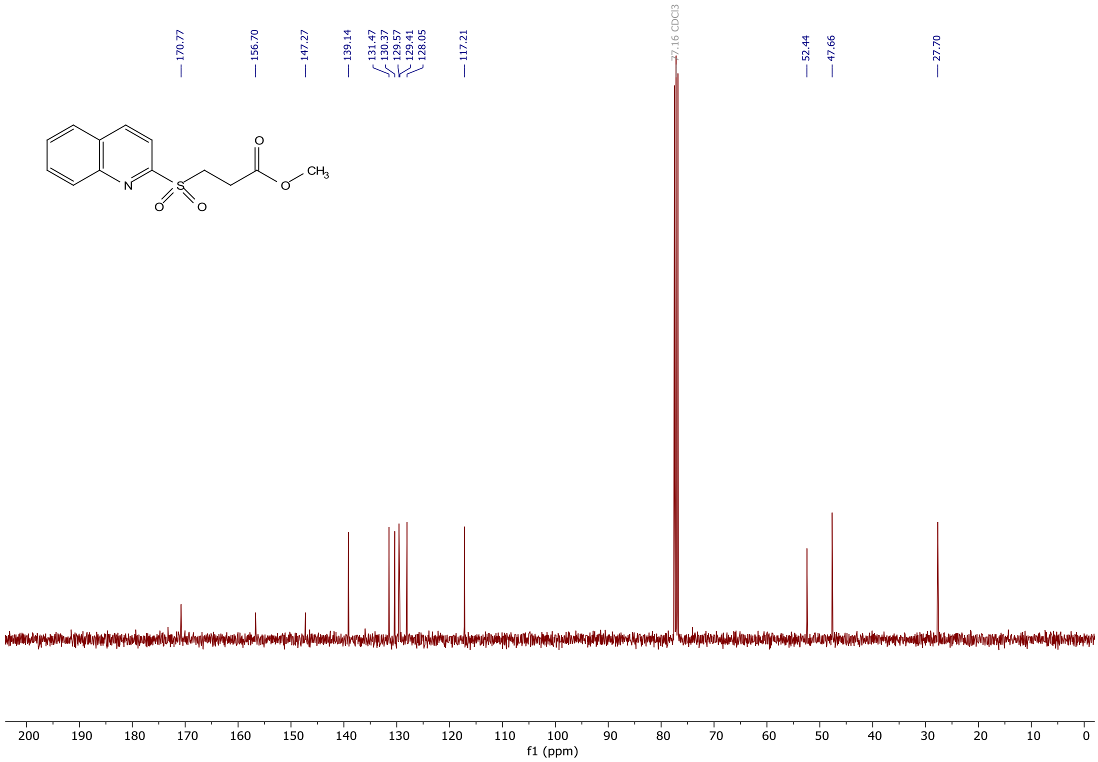

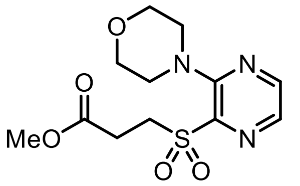

methyl 3-((3-morpholinopyrazin-2-yl)sulfonyl)propanoate (4j)

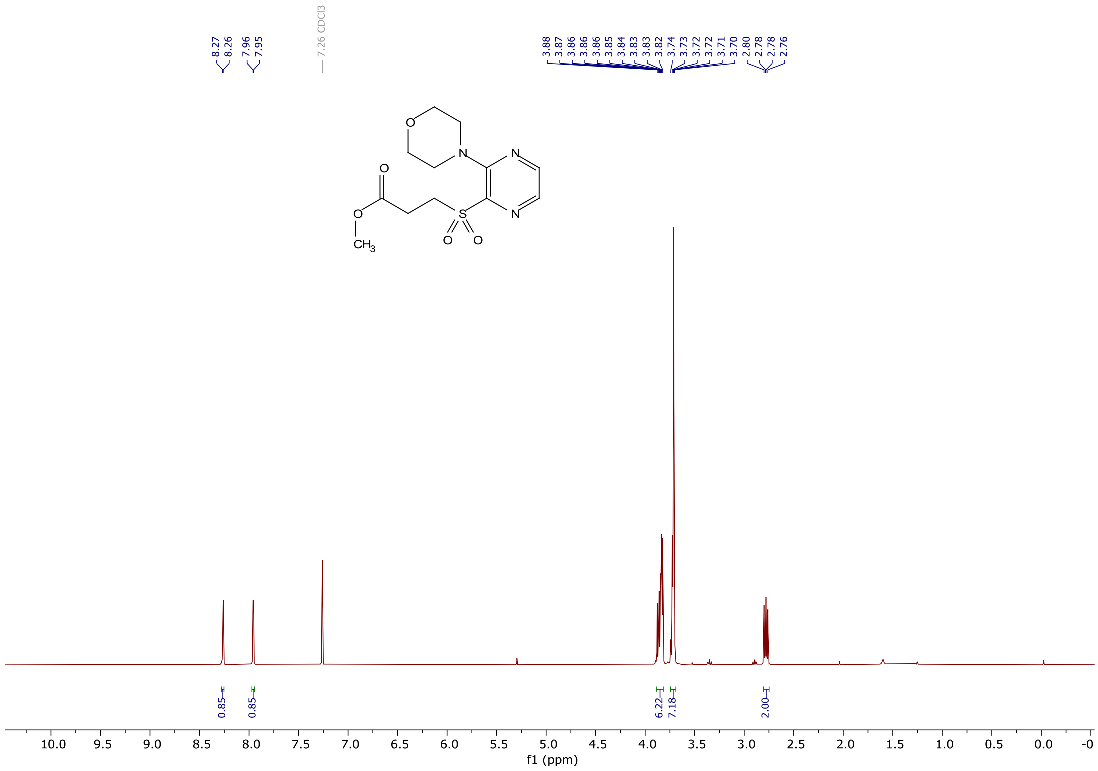

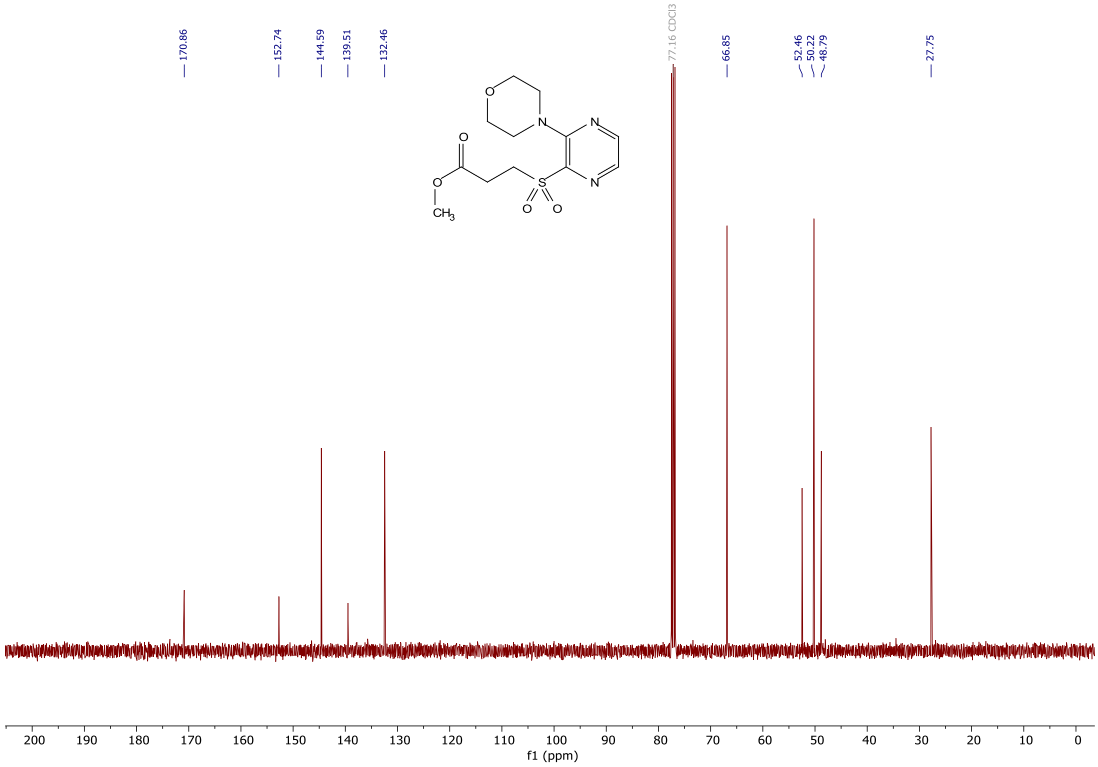

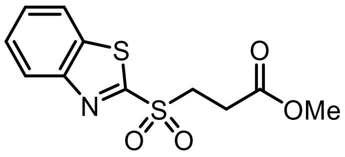

methyl 3-(benzo[d]thiazol-2-ylsulfonyl)propanoate (4k)

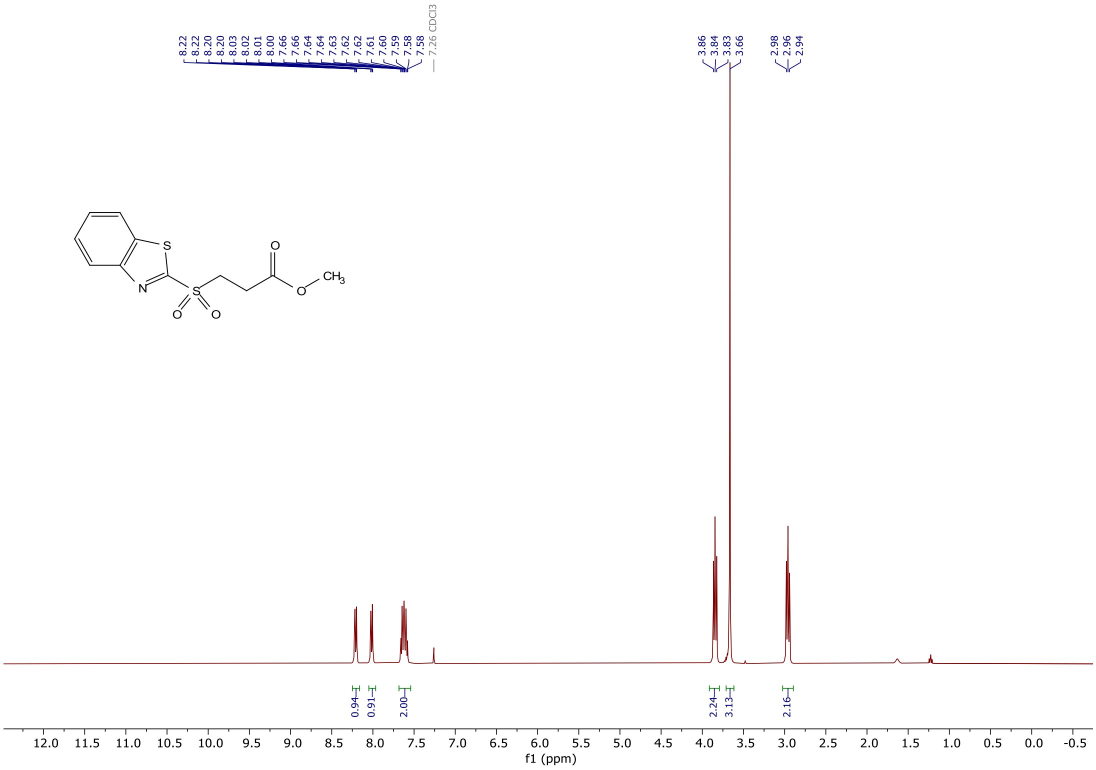

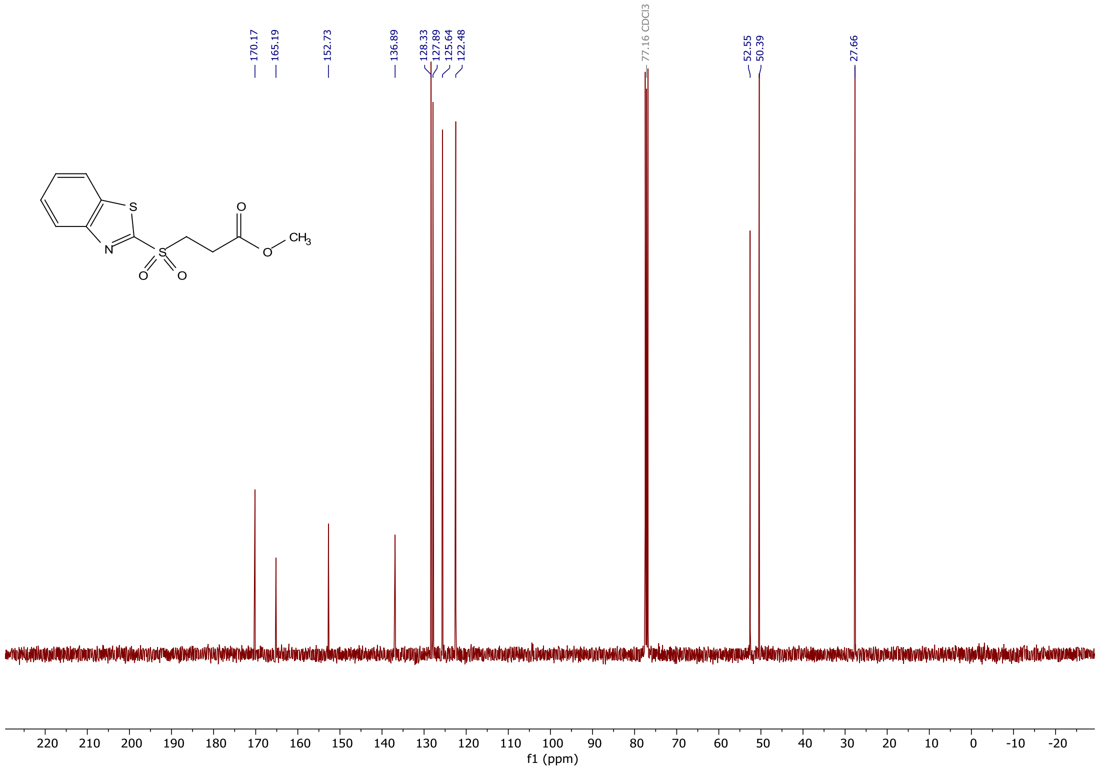

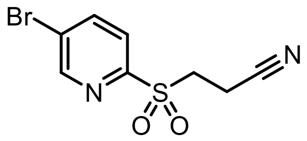

3-((5-bromopyridin-2-yl)sulfonyl)propanenitrile (9a)

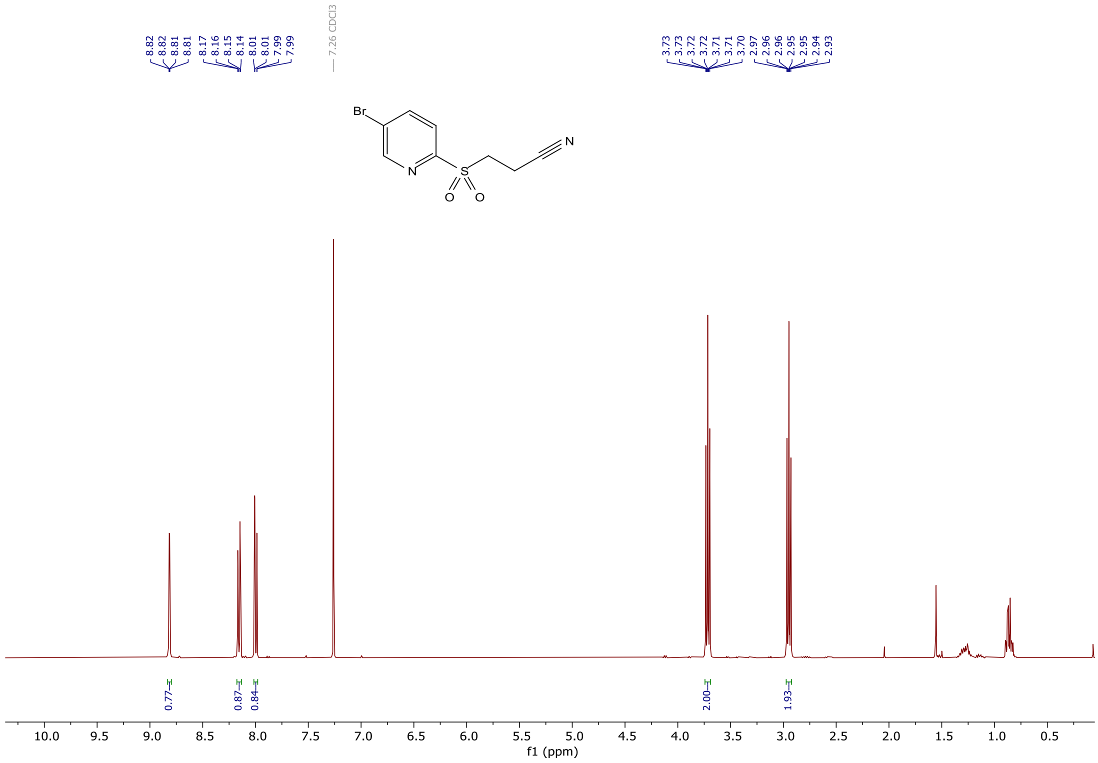

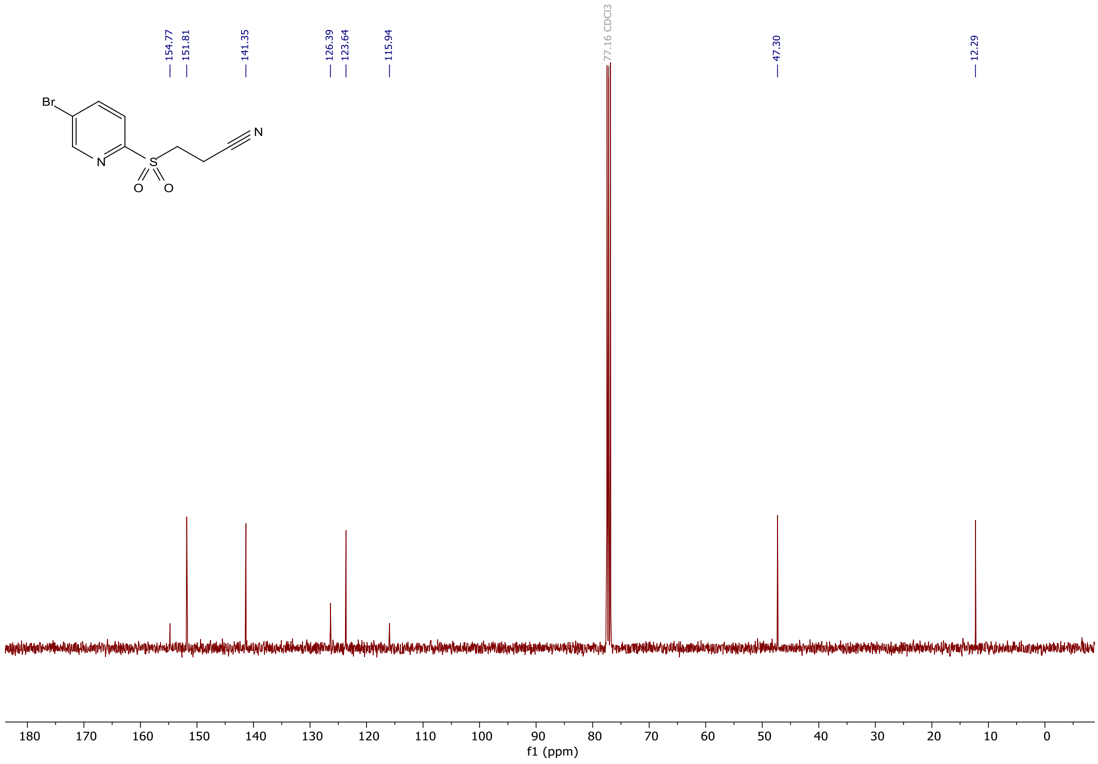

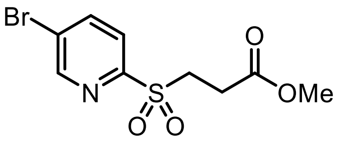

methyl 3-((5-bromopyridin-2-yl)sulfonyl)propanoate (9b)

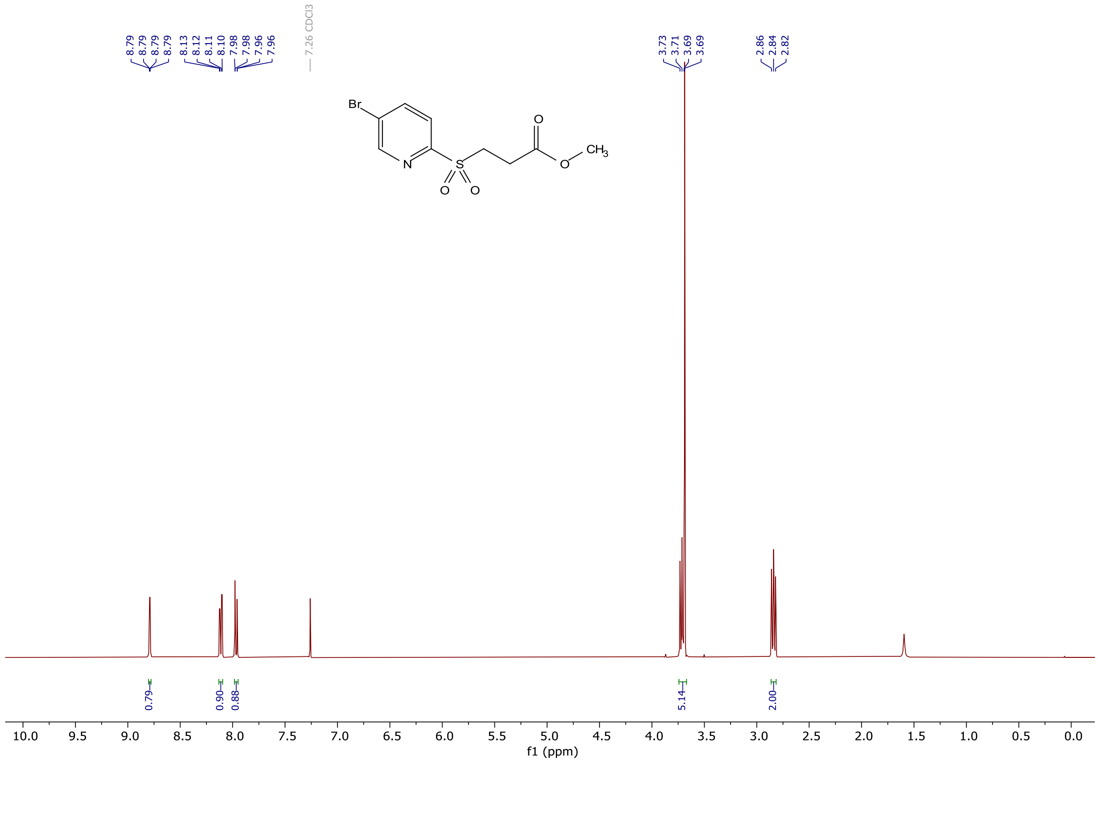

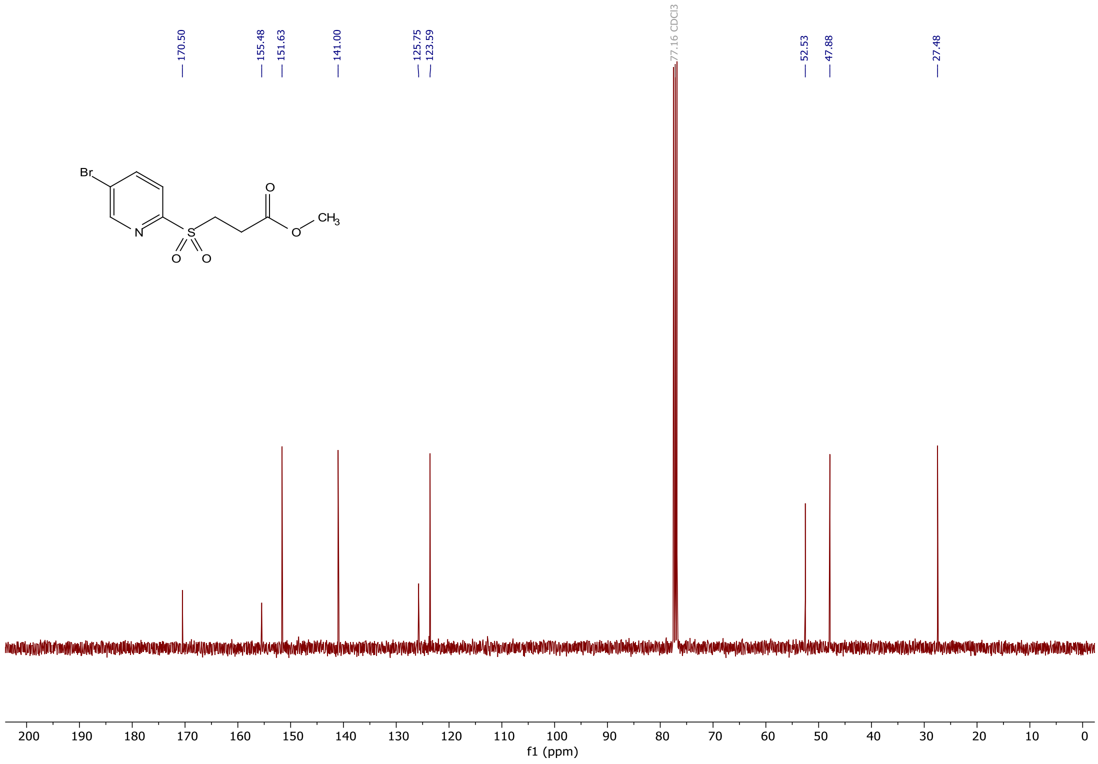

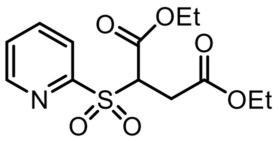

diethyl 2-(pyridin-2-ylsulfonyl)succinate (5a)

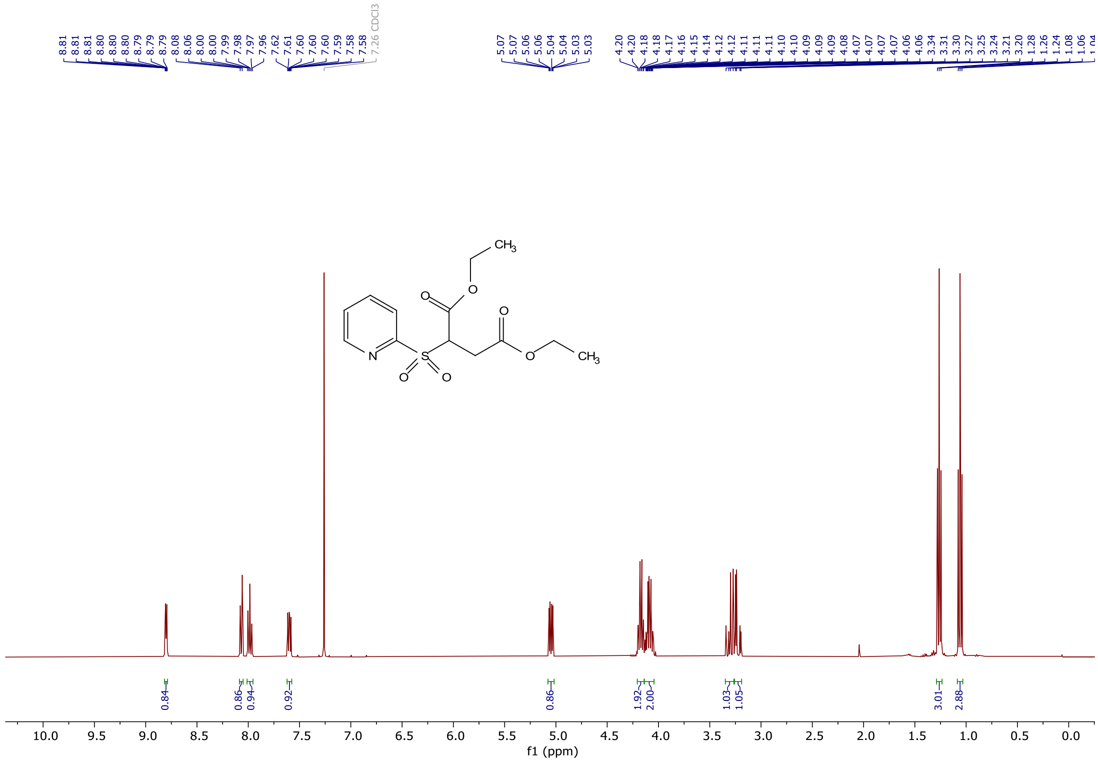

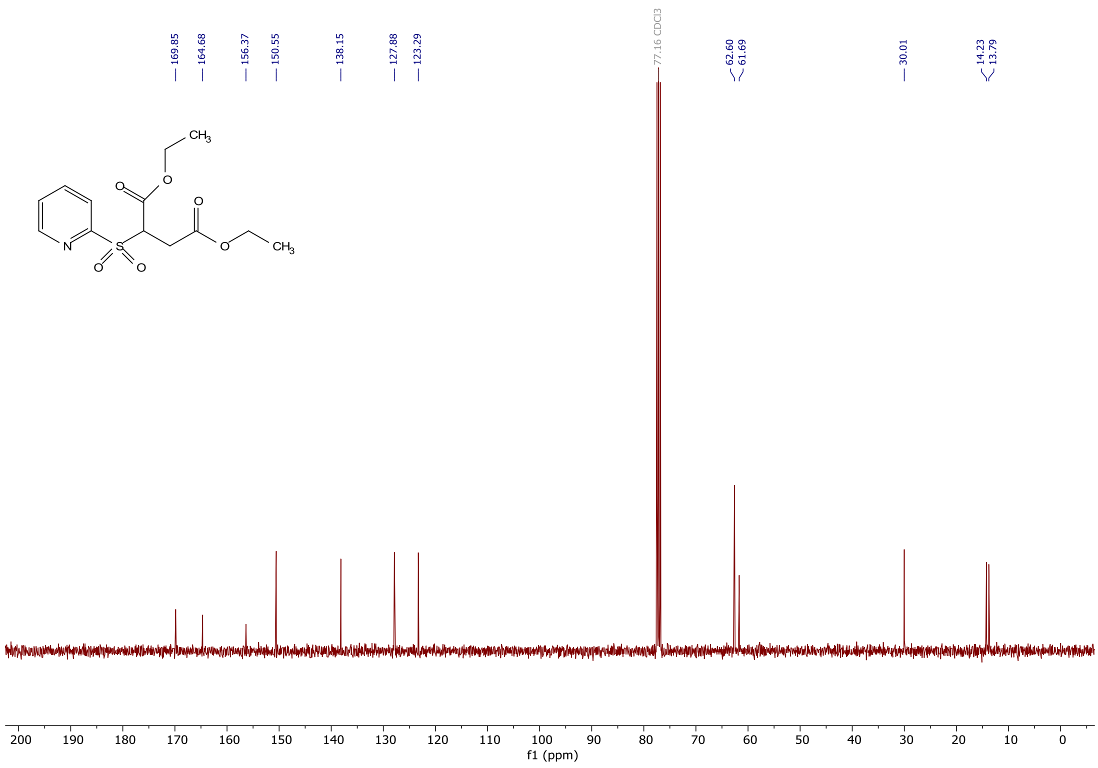

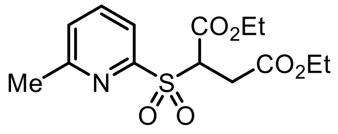

diethyl 2-((6-methylpyridin-2-yl)sulfonyl)succinate (5b)

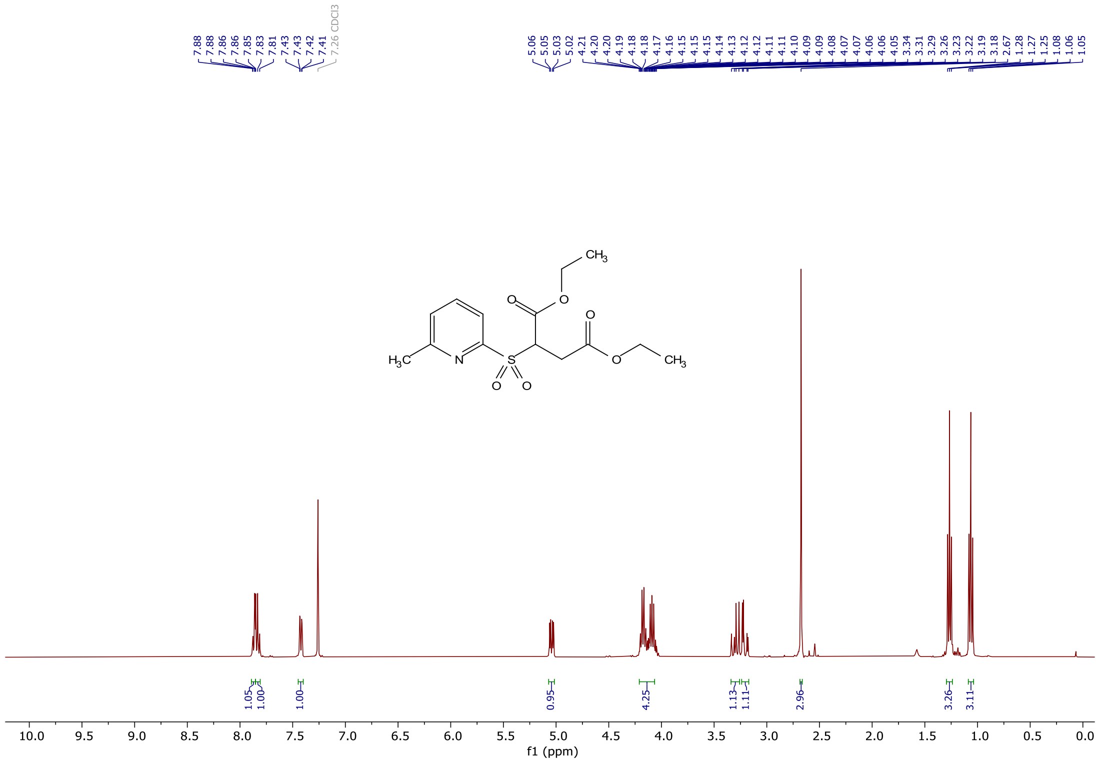

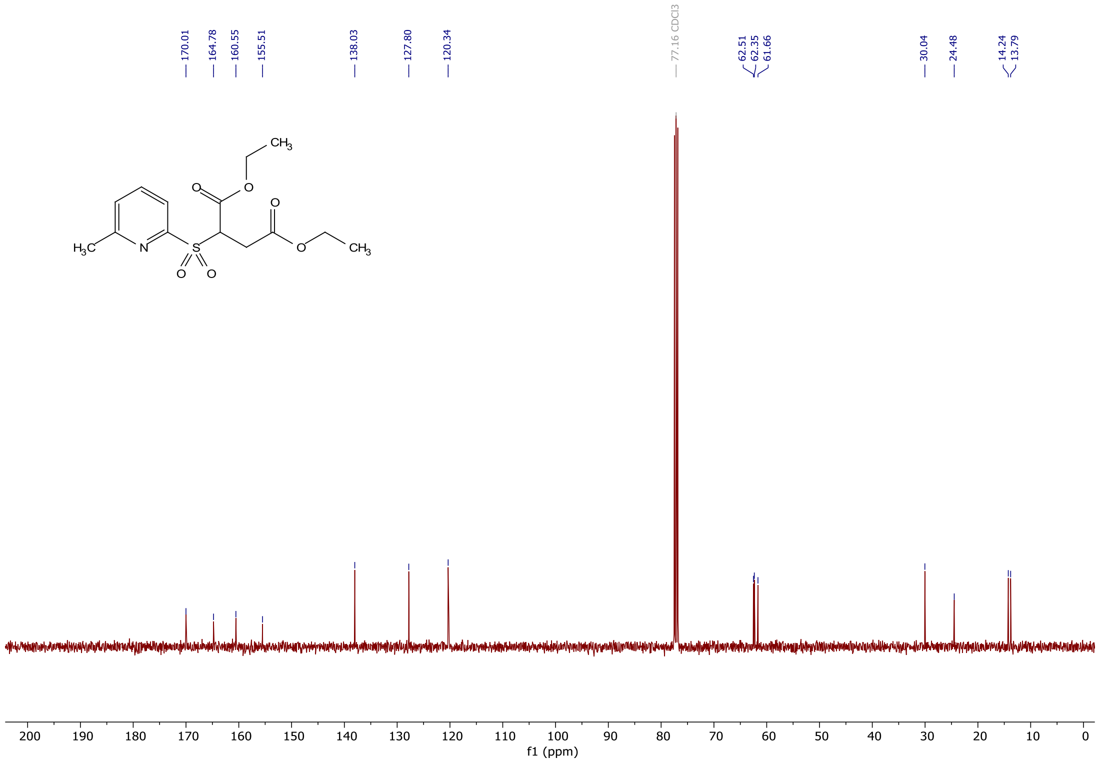

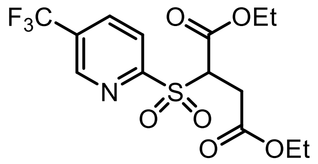

diethyl 2-((5-(trifluoromethyl)pyridin-2-yl)sulfonyl)succinate (5c)

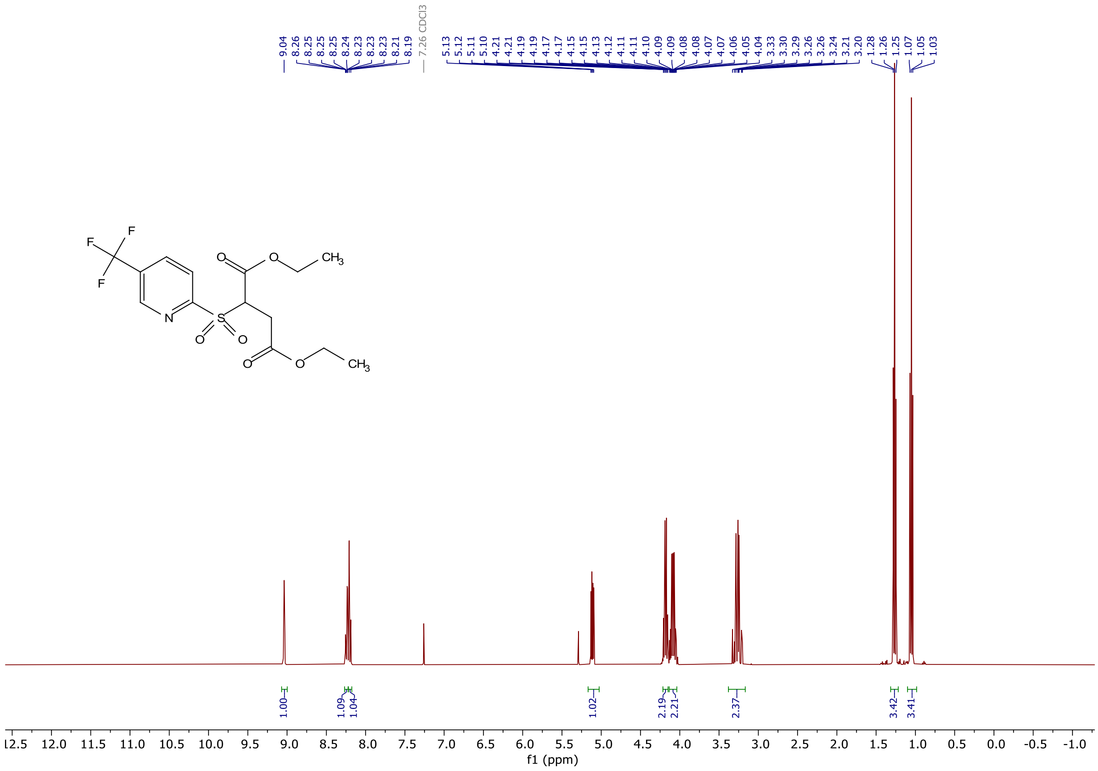

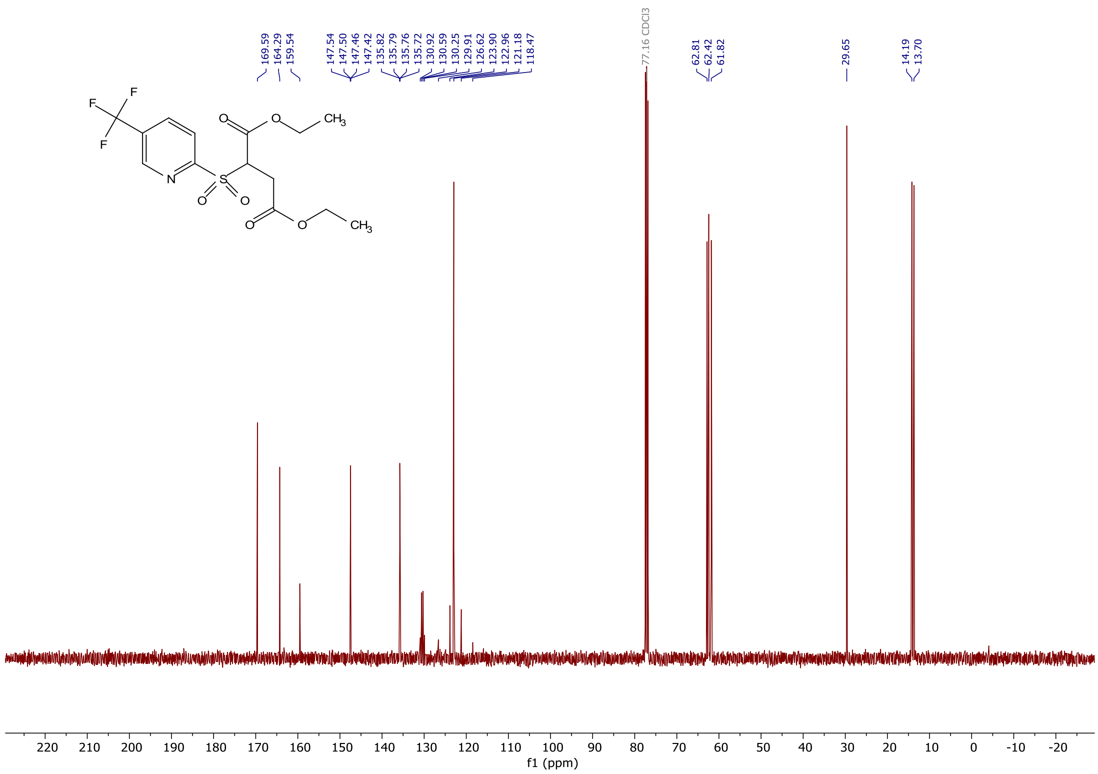
169.59
164.29
159.54
147.54
147.50
147.46
147.42
135.82
135.79
135.76
135.72
130.92
130.59
130.25
129.91
126.62
123.90
122.96
121.18
118.47
77.16 CDCl3
62.81
62.42
61.82
29.65
14.19
13.70
f1 (ppm)

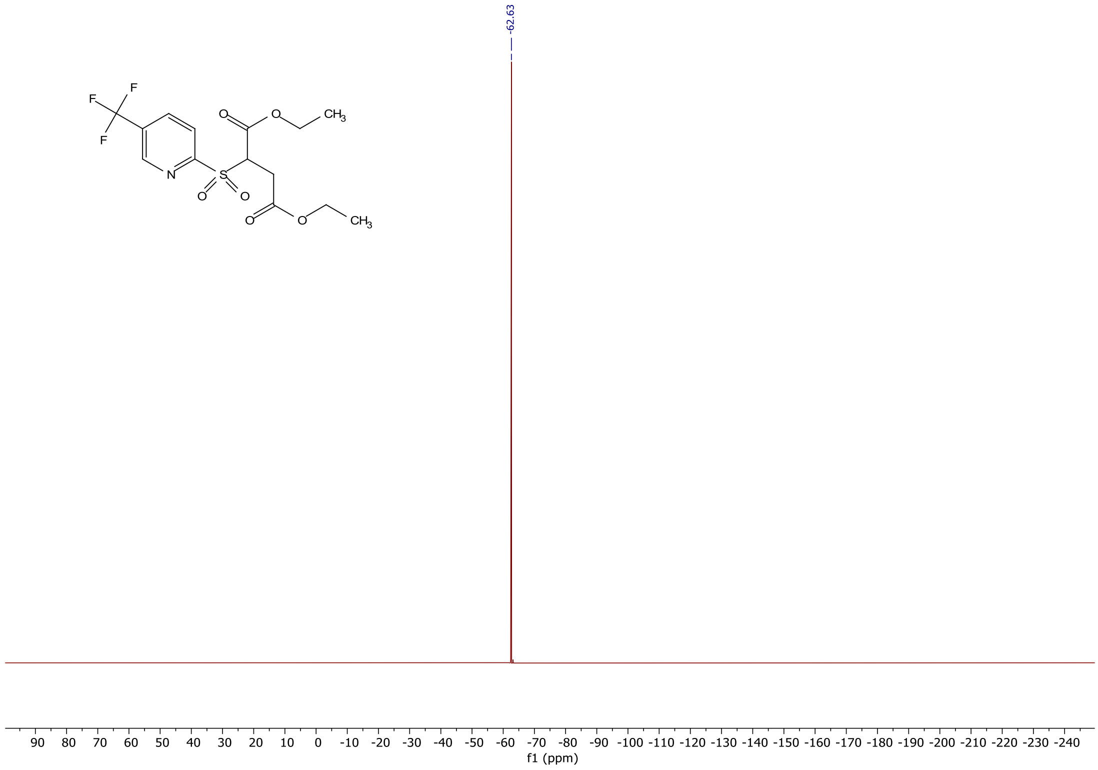
-62.63
f1 (ppm)

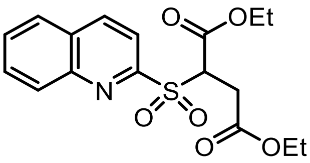

diethyl 2-(quinolin-2-ylsulfonyl)succinate (5d)

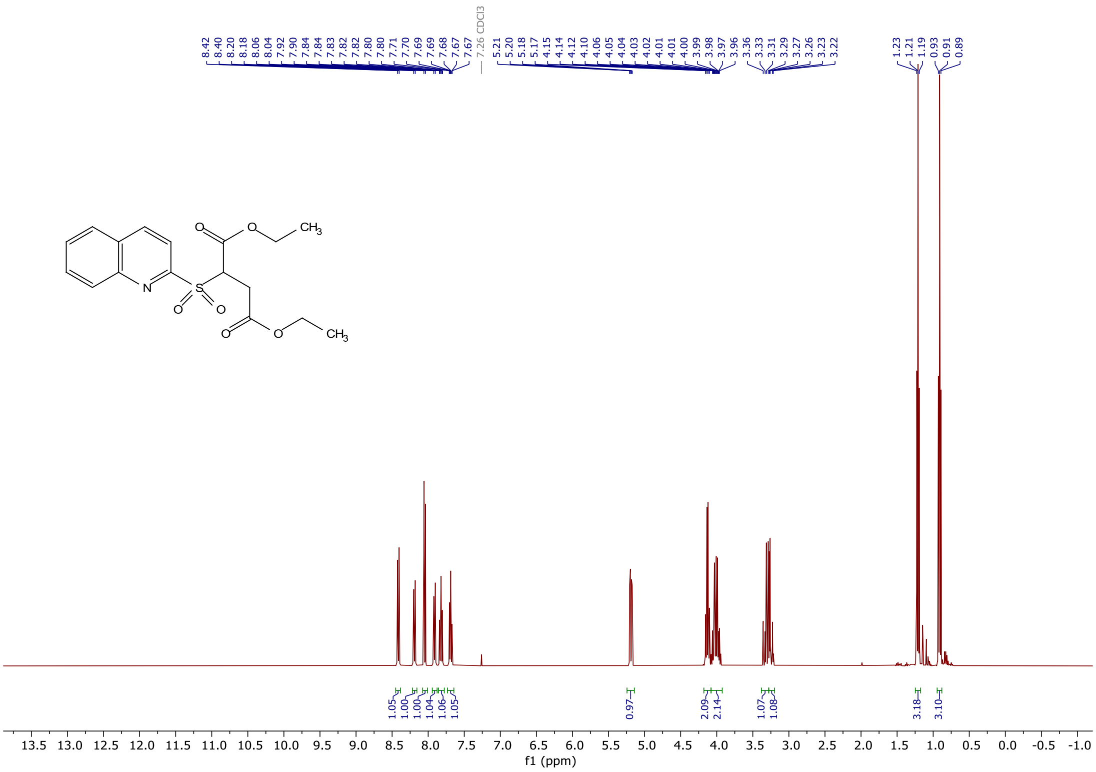

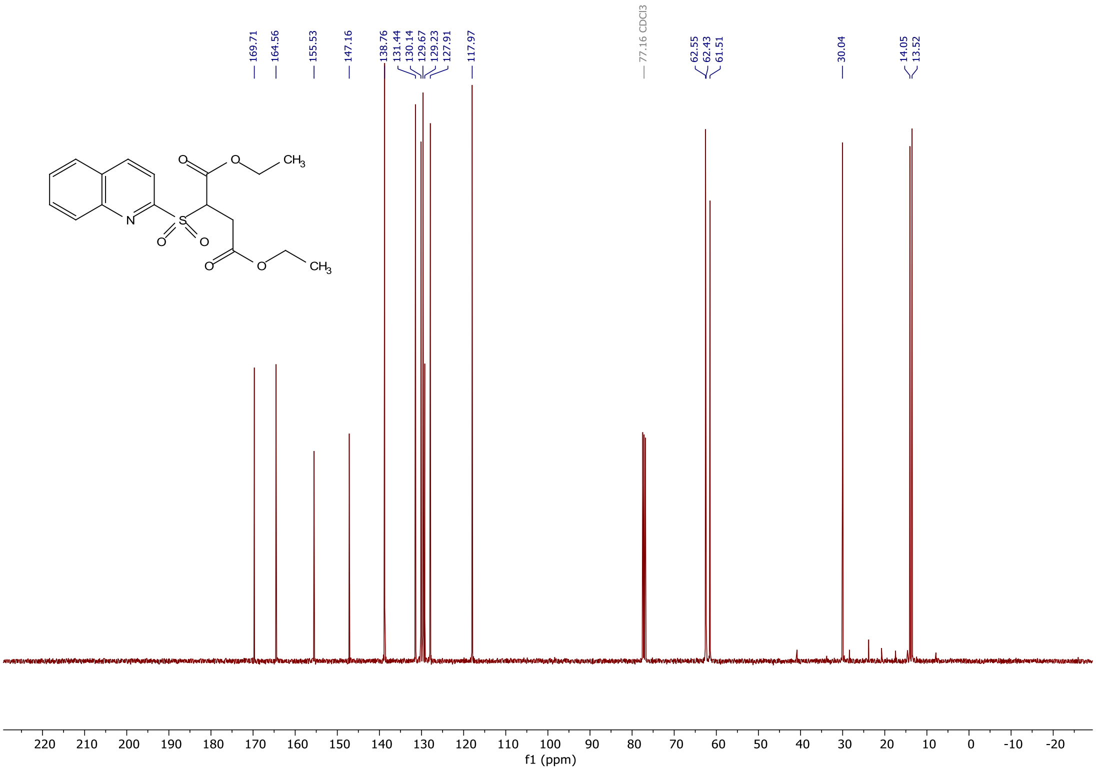

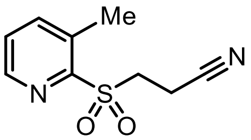

3-((3-methylpyridin-2-yl)sulfonyl)propanenitrile (1g)

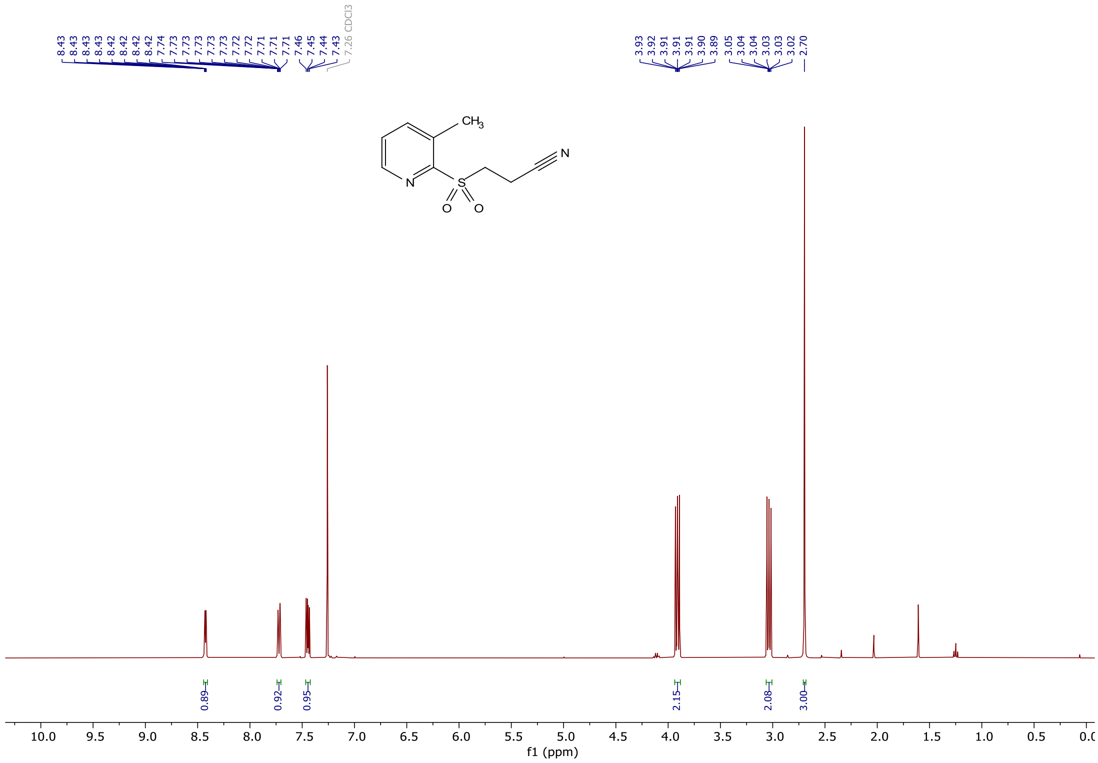

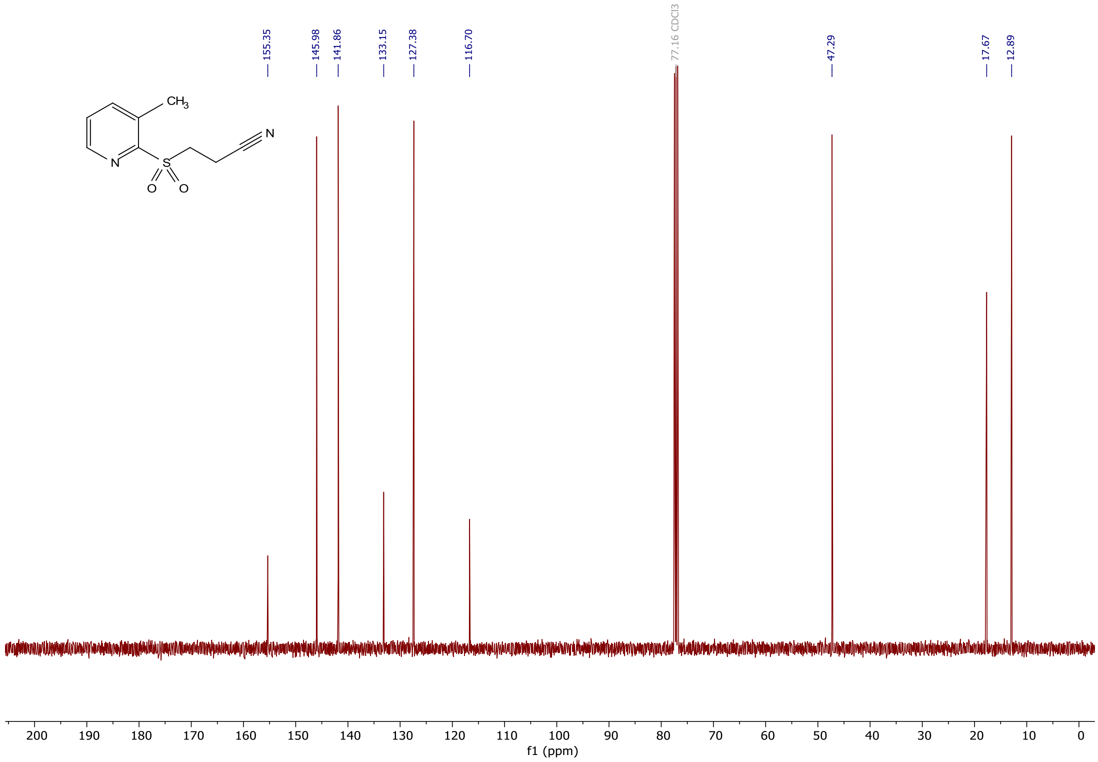

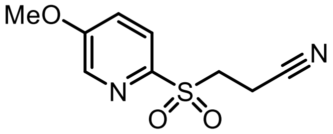

3-((5-methoxypyridin-2-yl)sulfonyl)propanenitrile (1h)

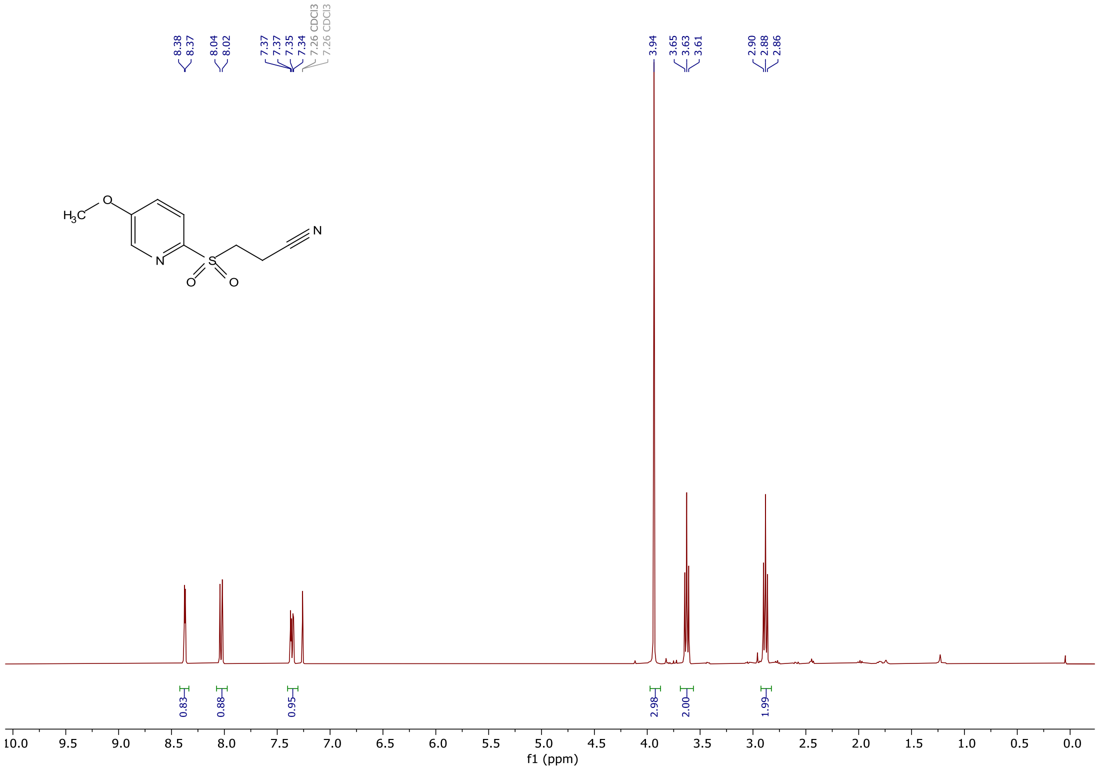

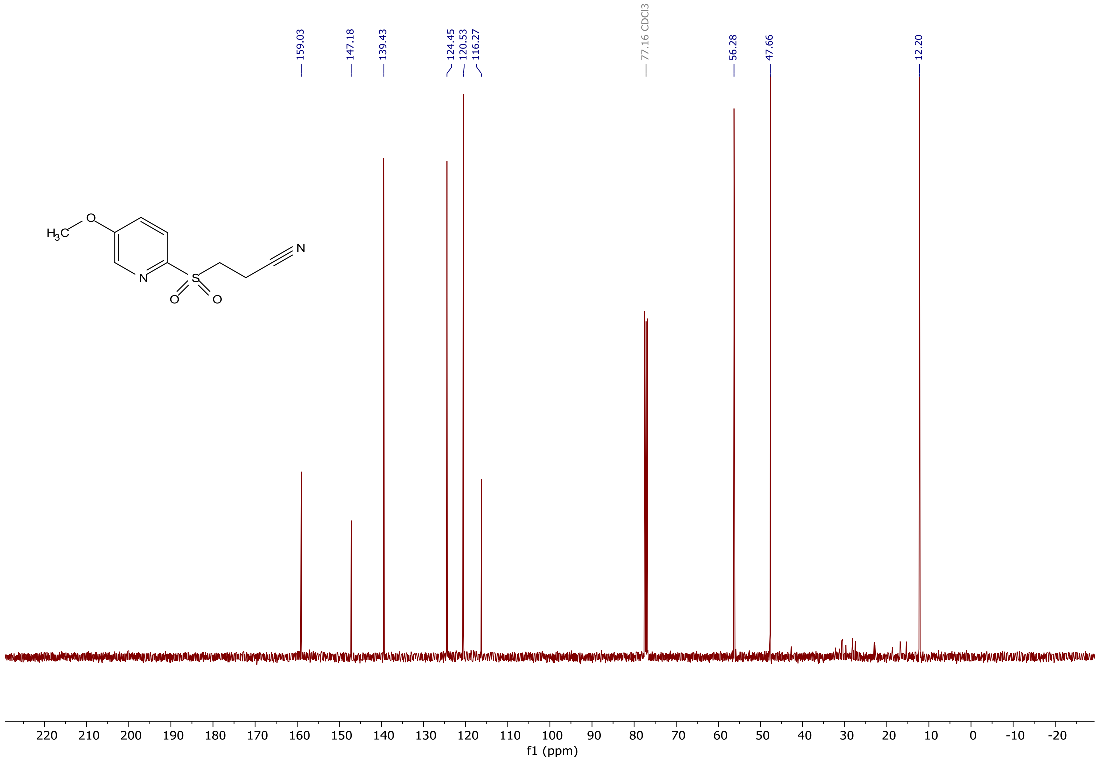

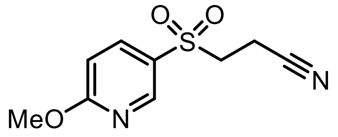

3-((6-methoxypyridin-3-yl)sulfonyl)propanenitrile (1i)

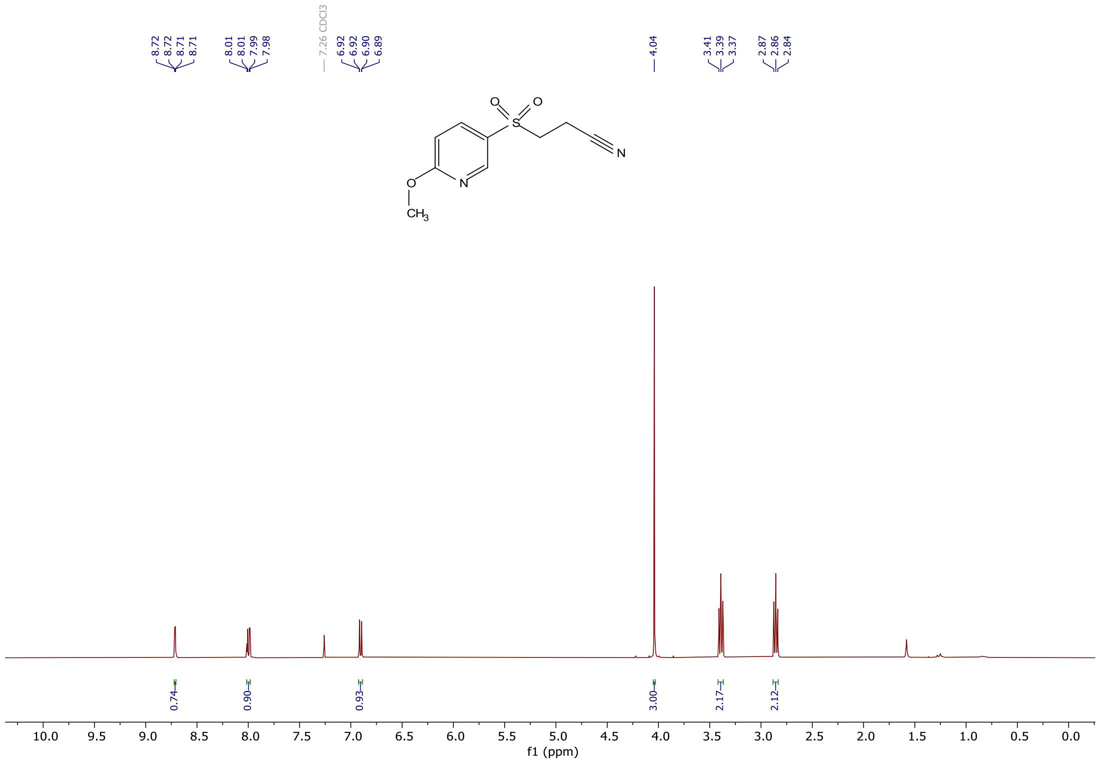

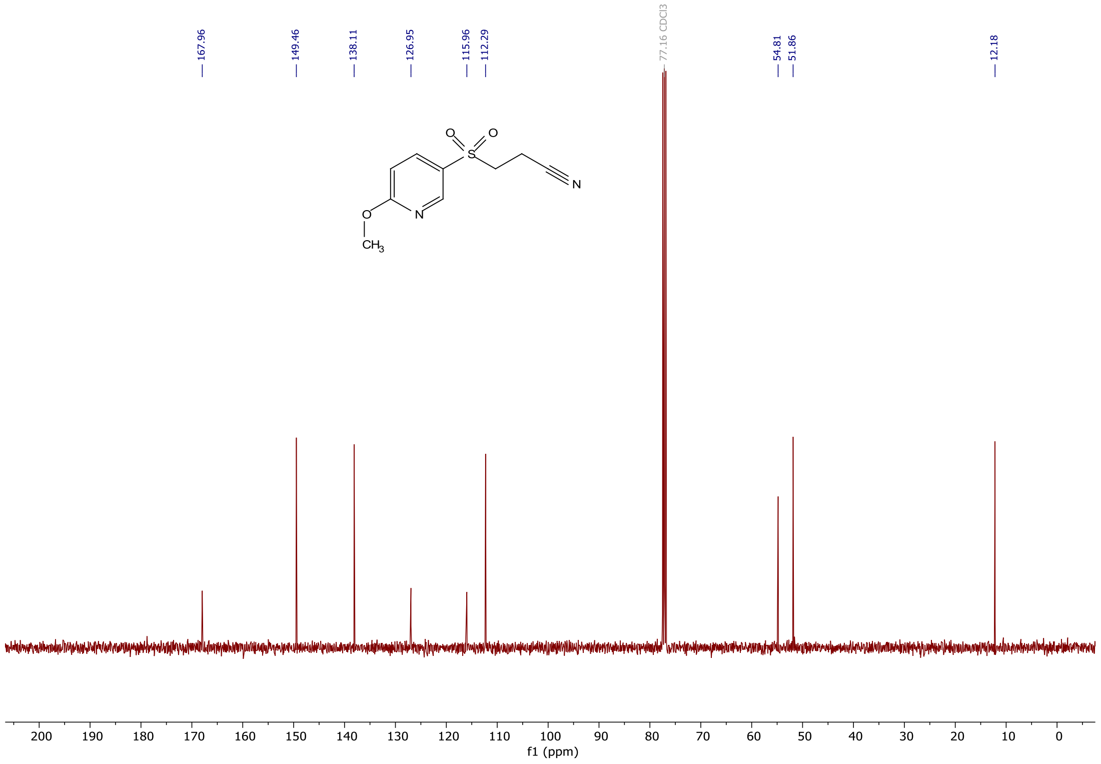

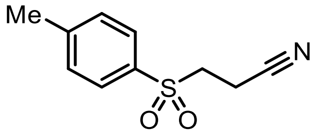

3-tosylpropanenitrile (1j)

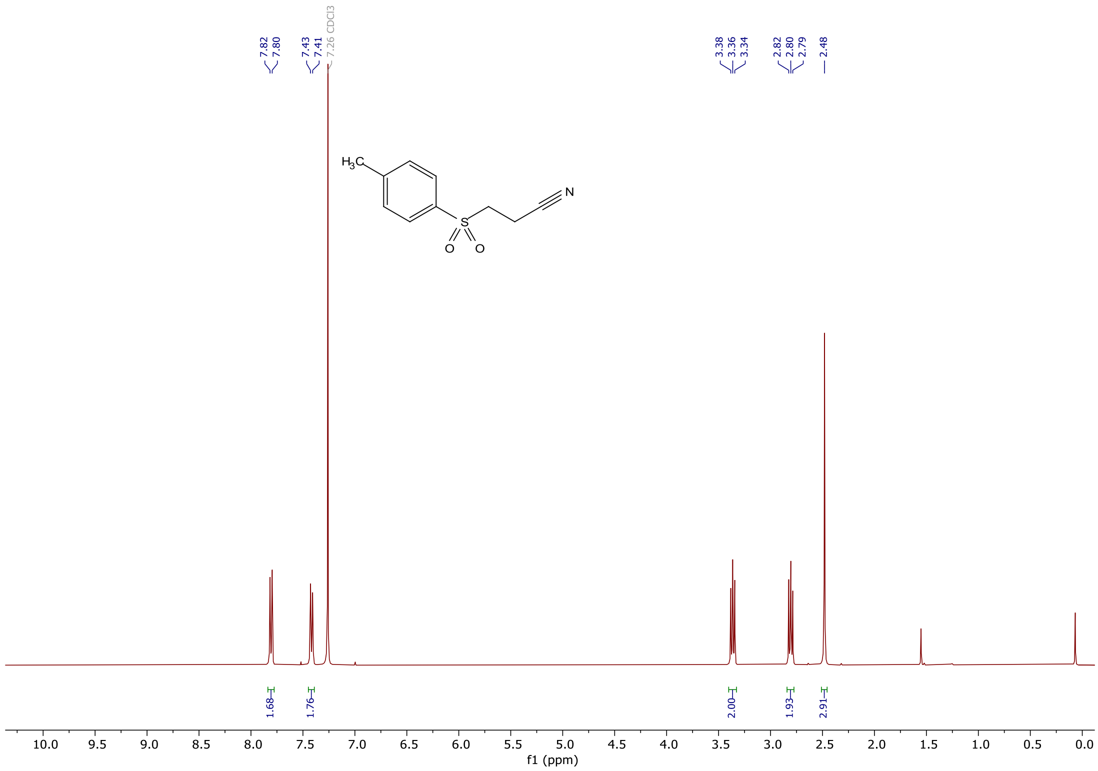

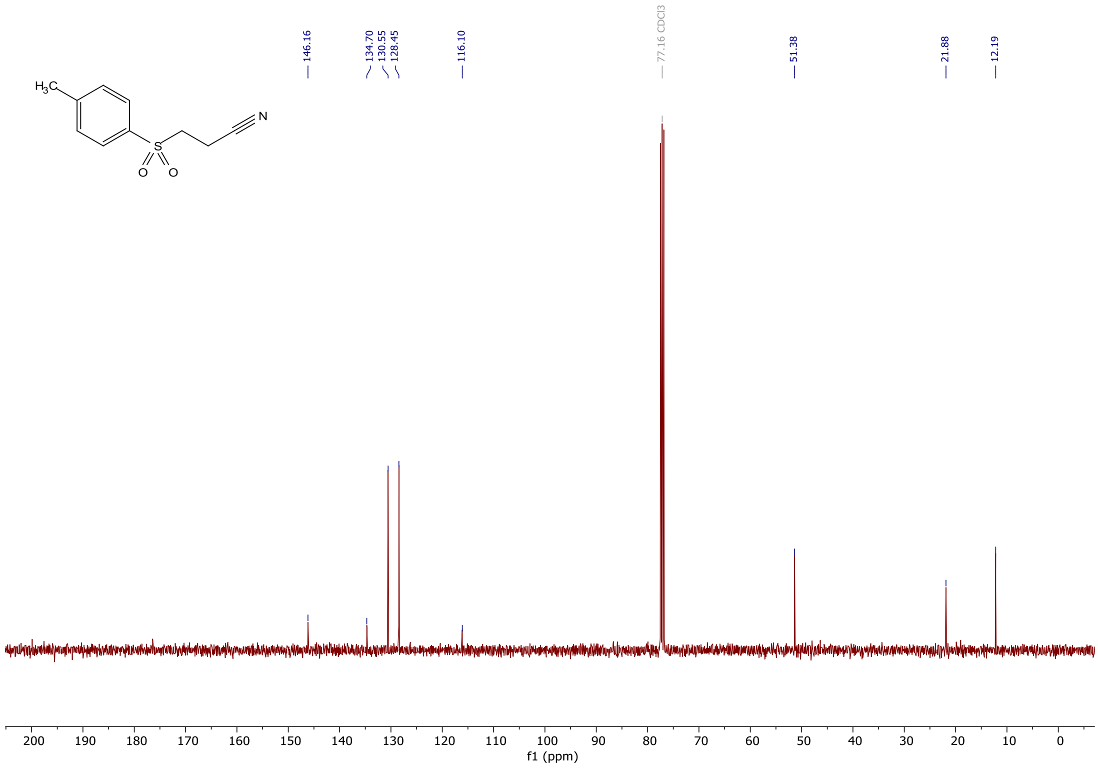

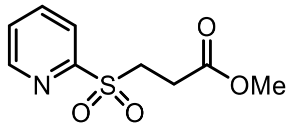

methyl 3-(pyridin-2-ylsulfonyl)propanoate (4l)

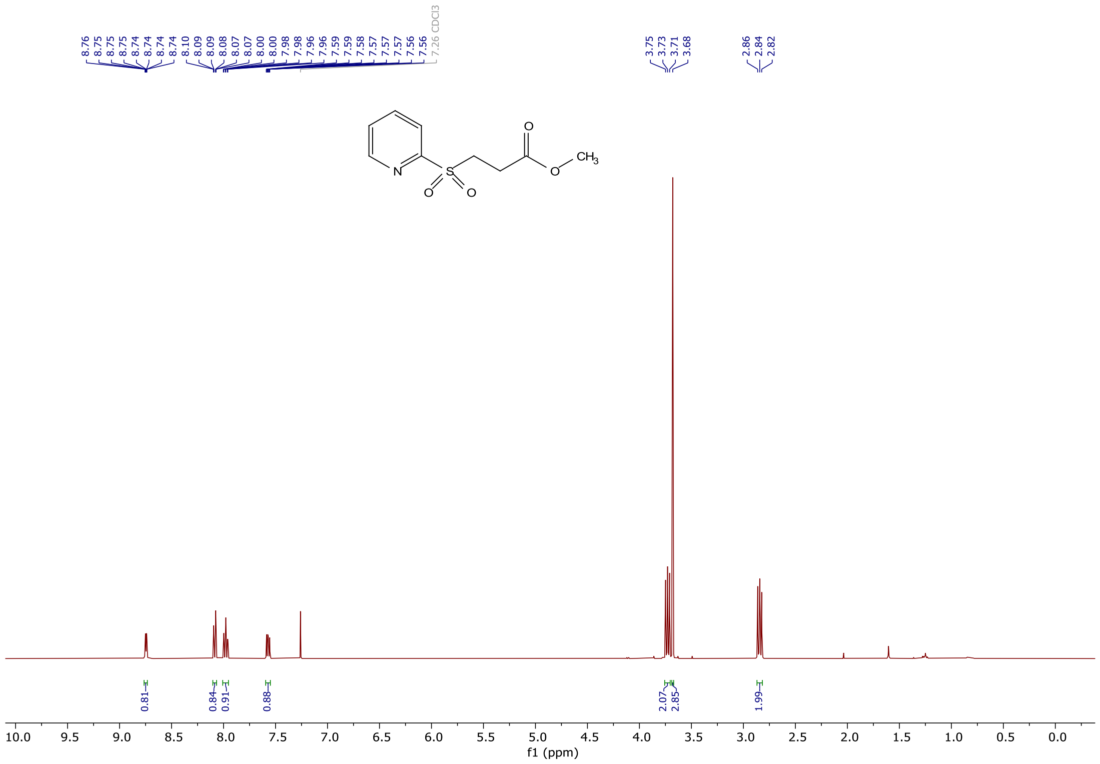

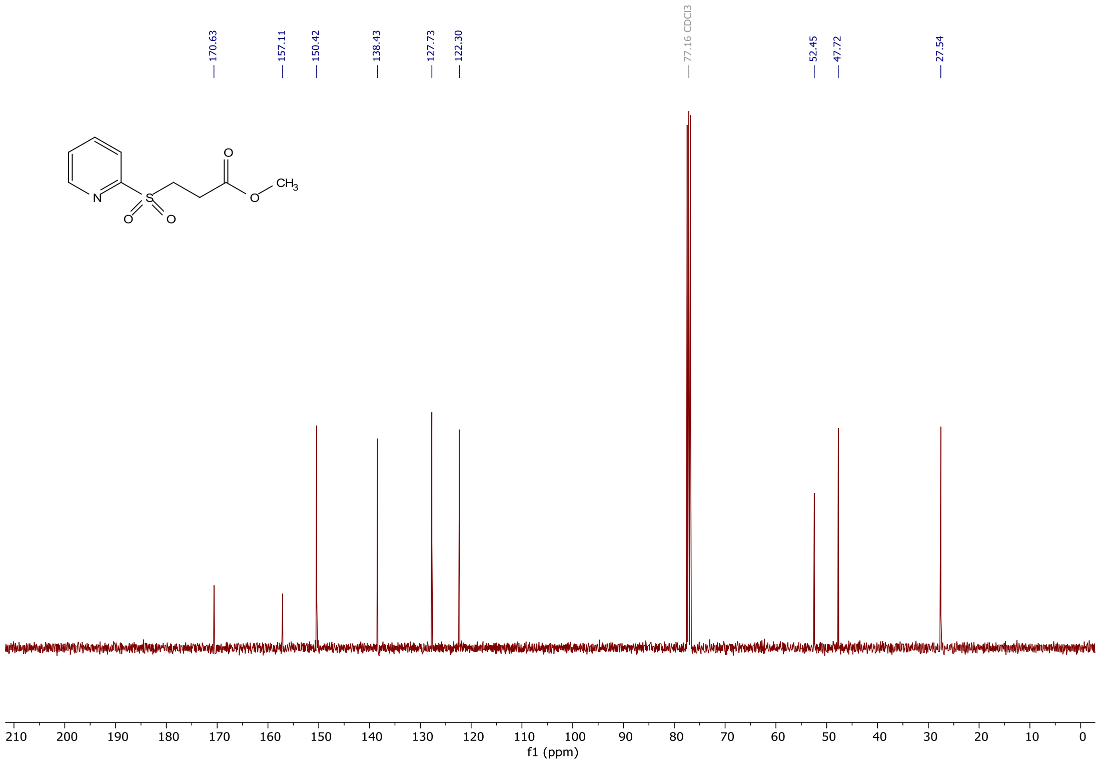

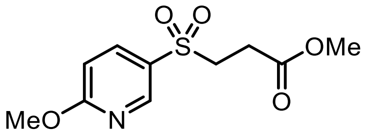

methyl 3-((6-methoxypyridin-3-yl)sulfonyl)propanoate (4m)

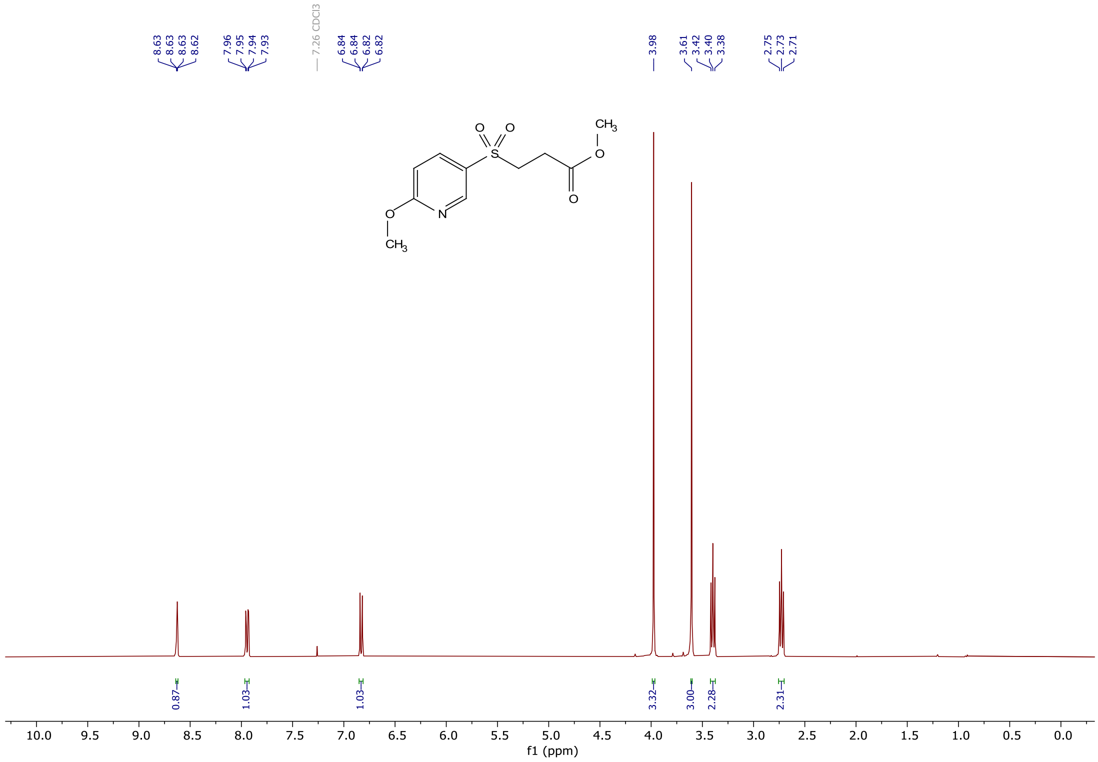

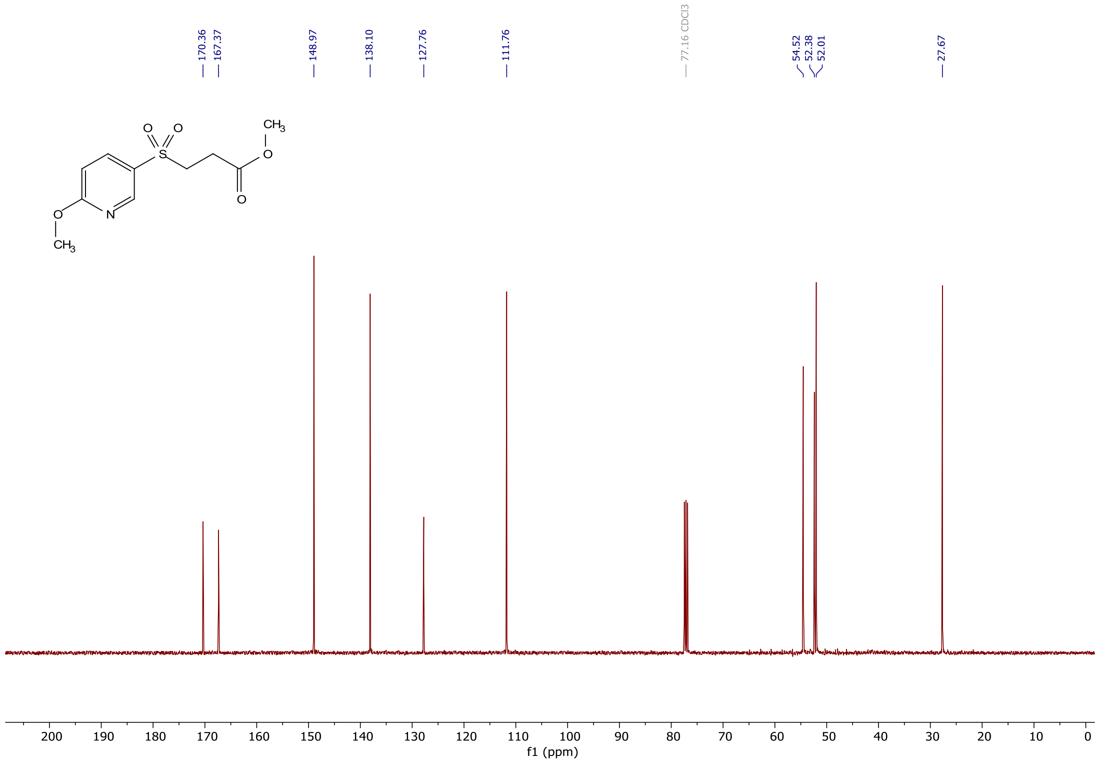

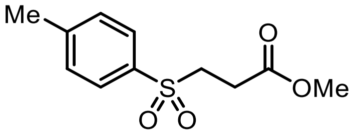

methyl 3-tosylpropanoate (4n)

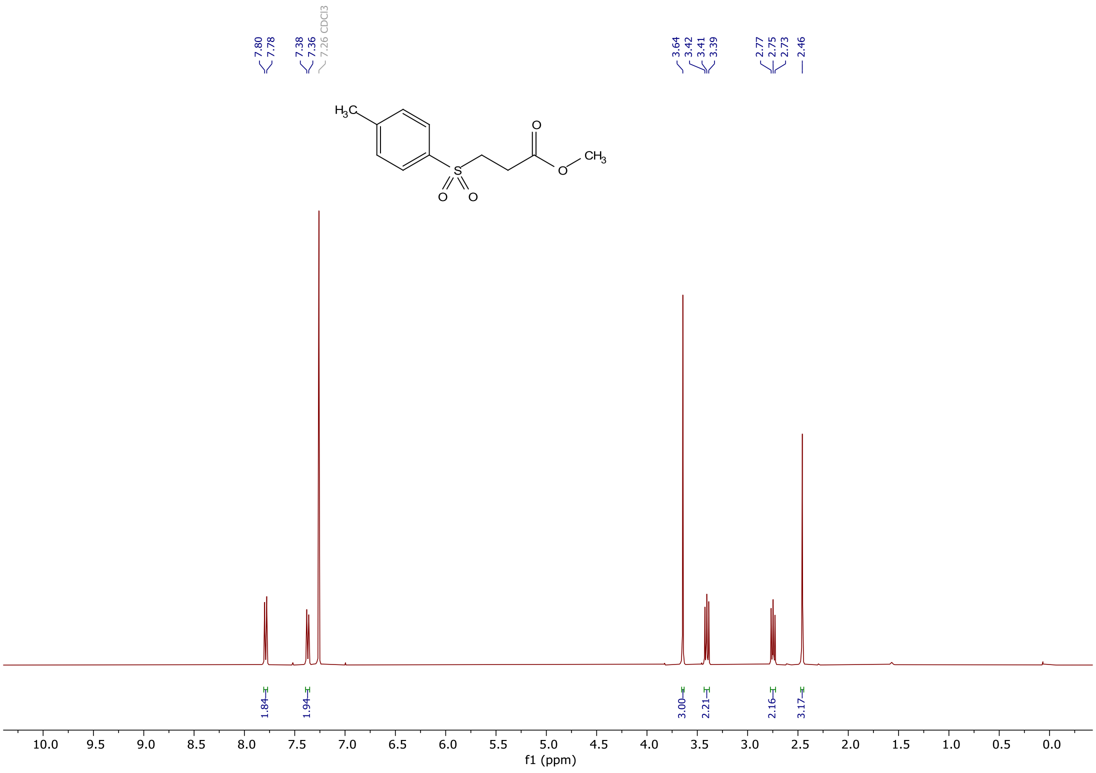

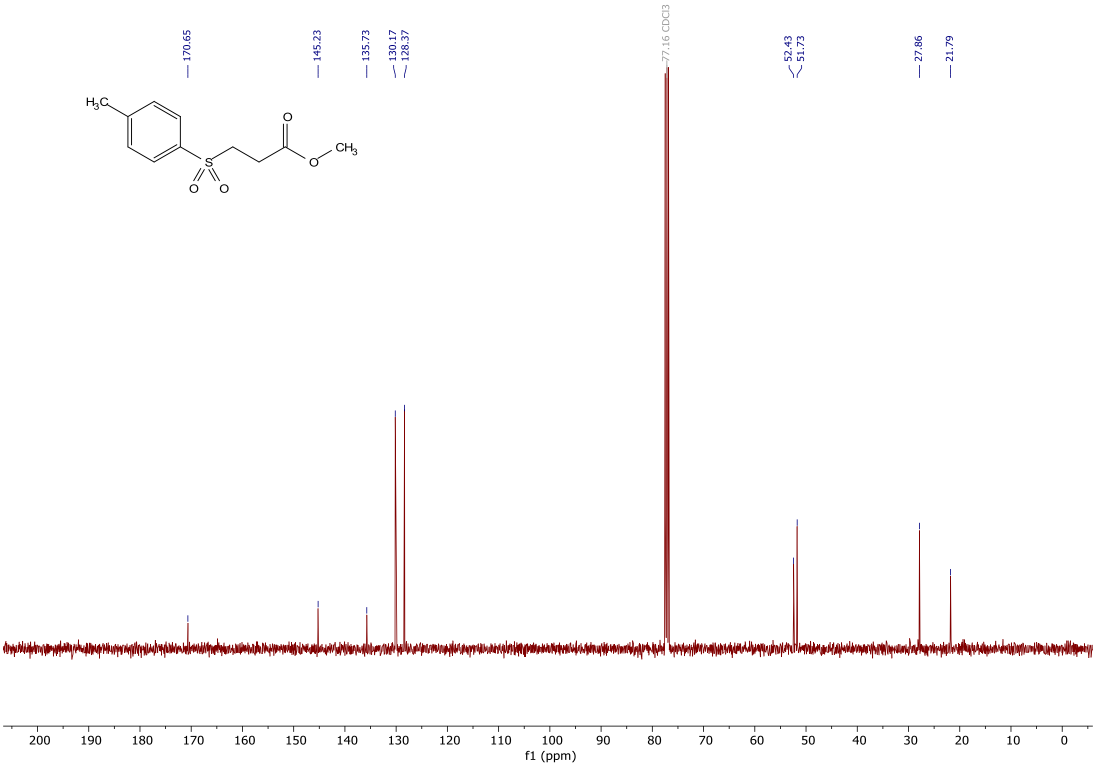

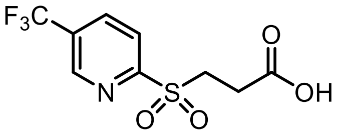

3-((5-(trifluoromethyl)pyridin-2-yl)sulfonyl)propanoic acid (4a-OH)

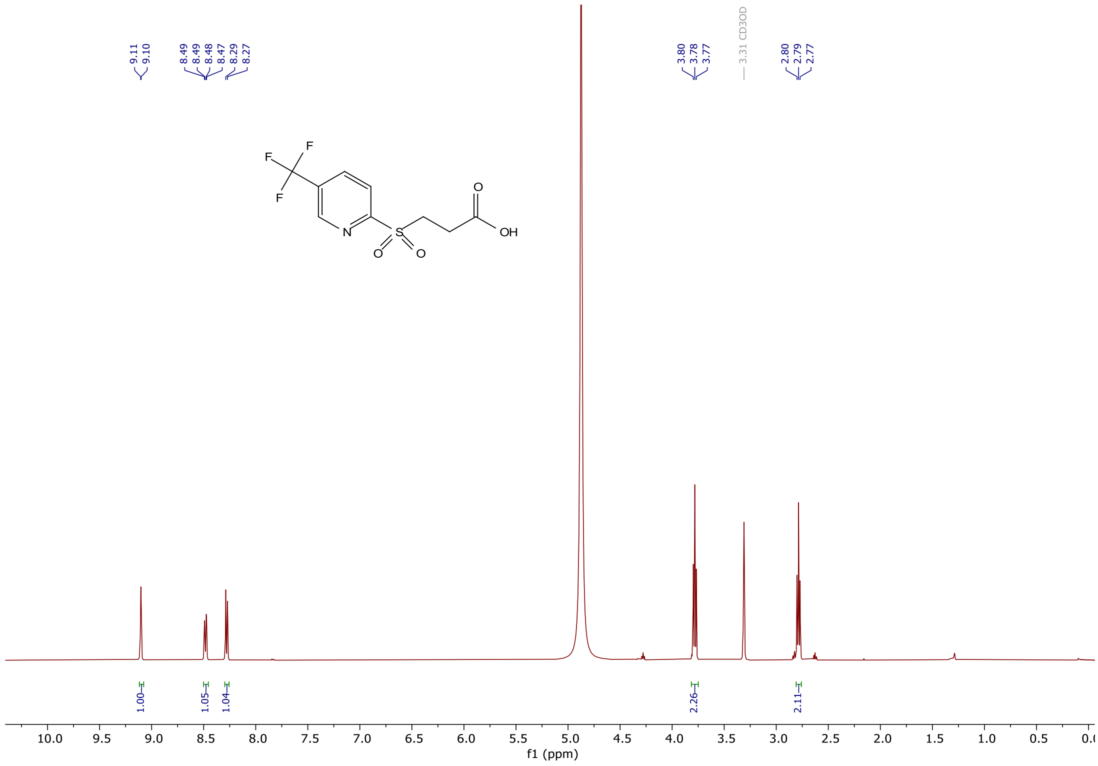

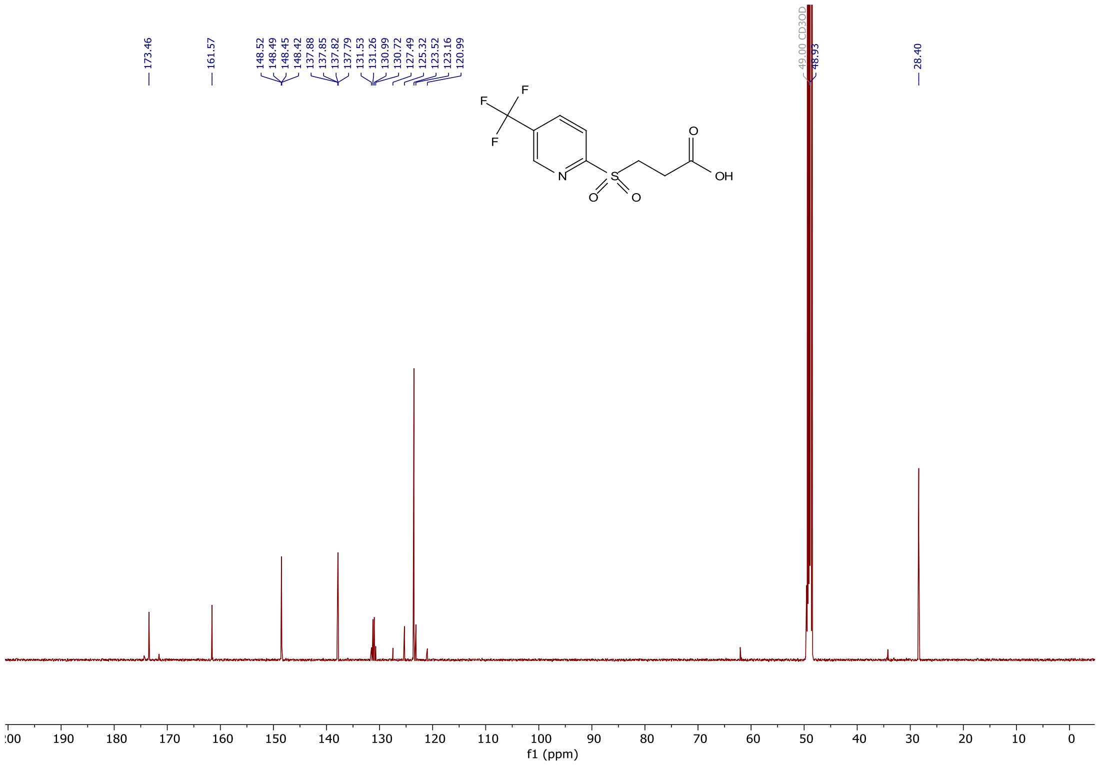

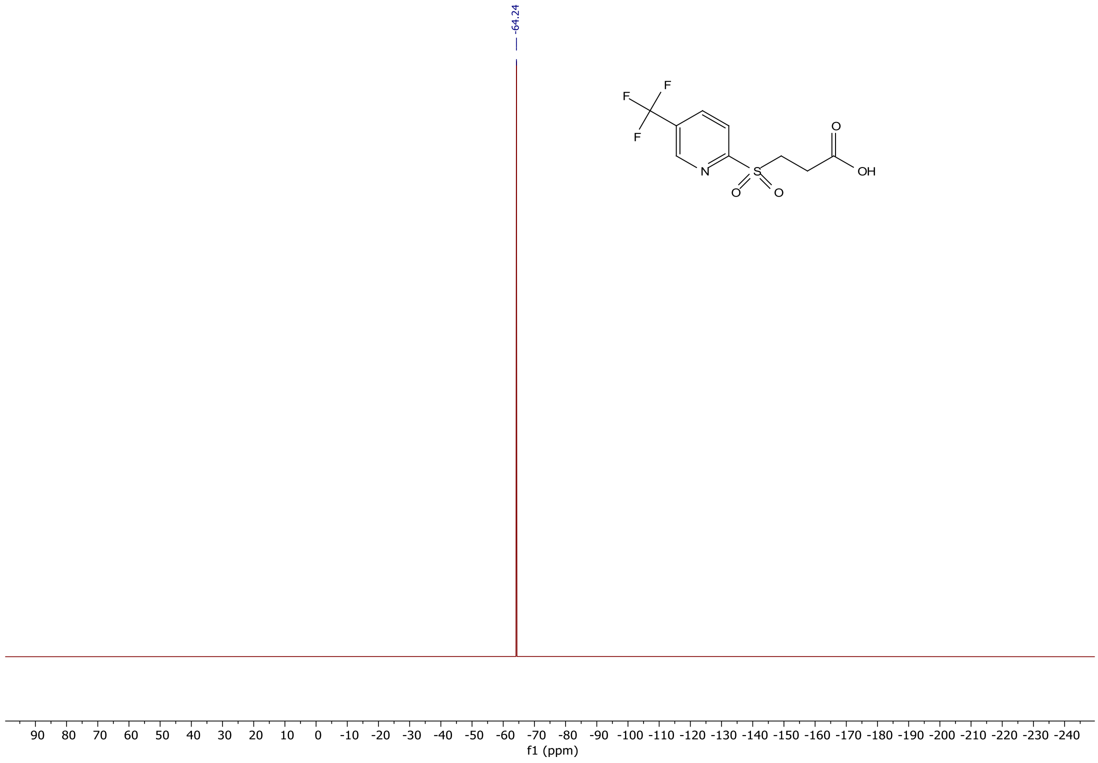

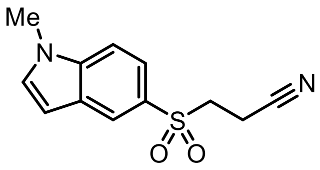

3-((1-methyl-1H-indol-5-yl)sulfonyl)propanenitrile (1k)

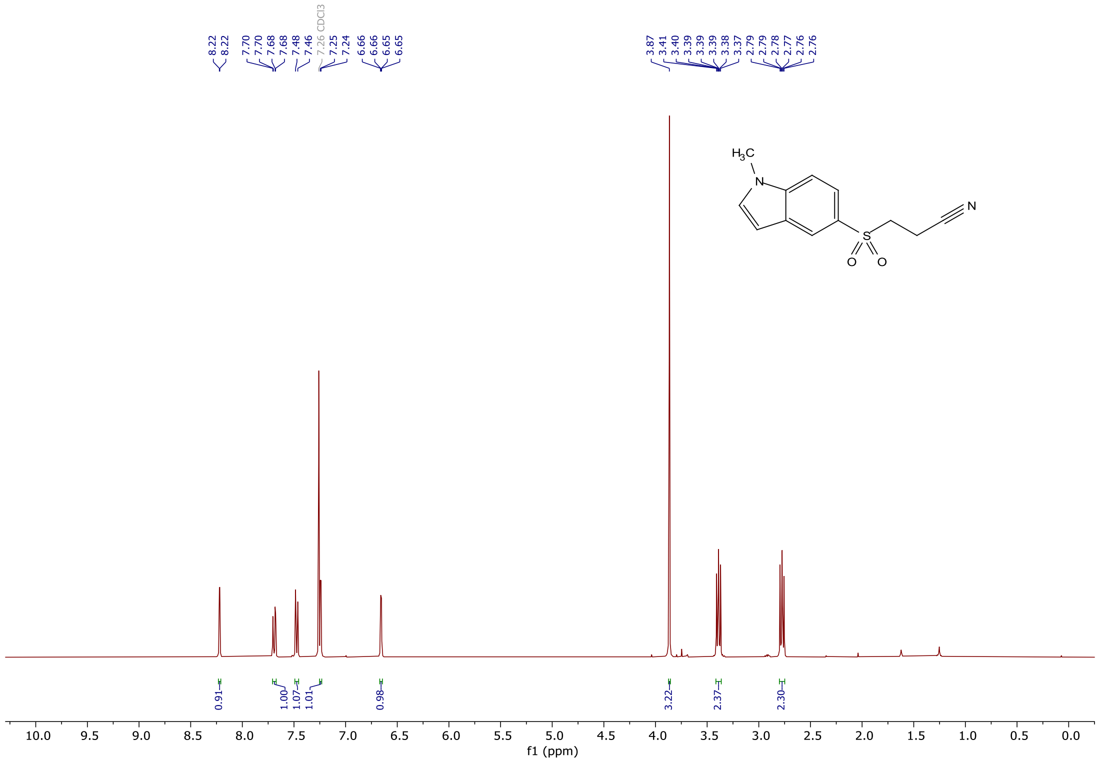

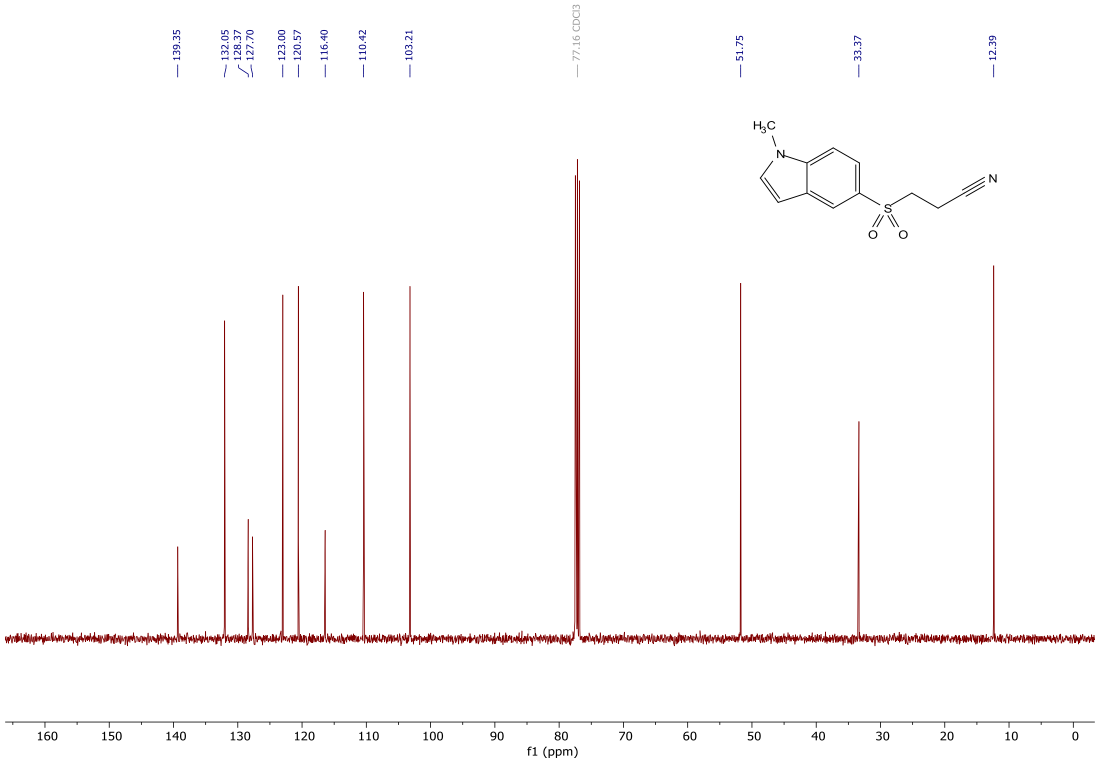

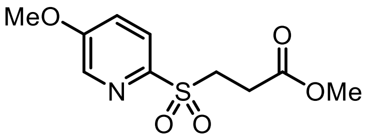

methyl 3-((5-methoxypyridin-2-yl)sulfonyl)propanoate (4o)

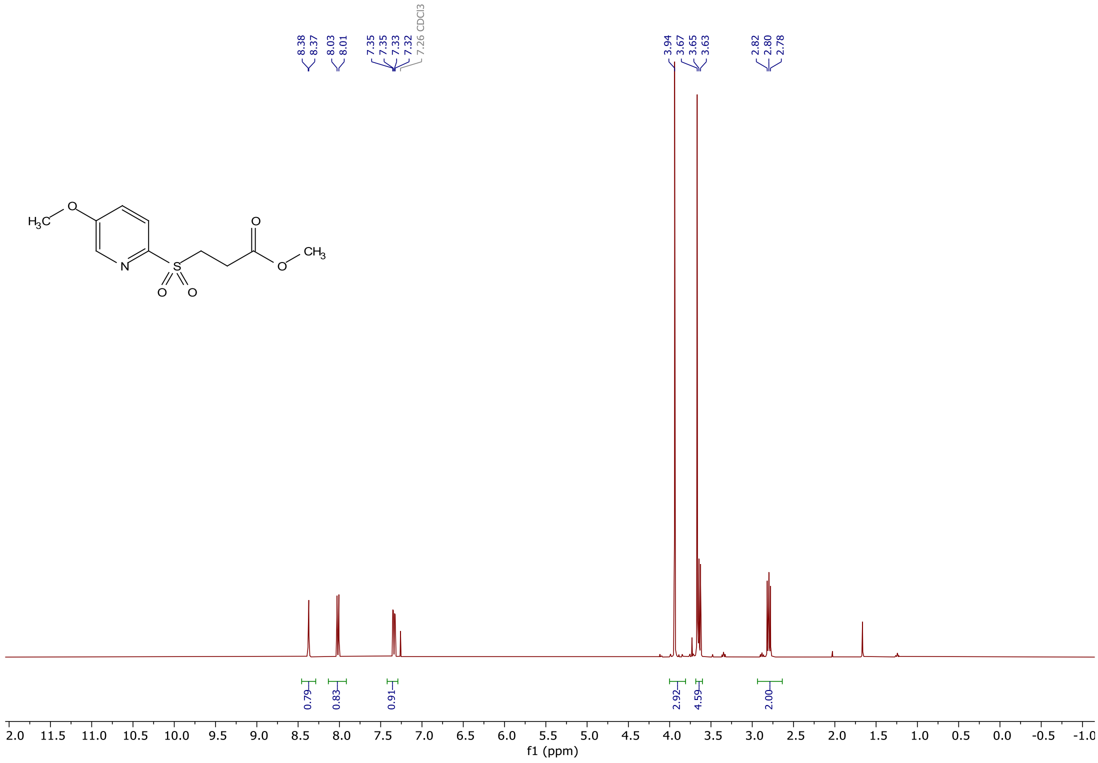

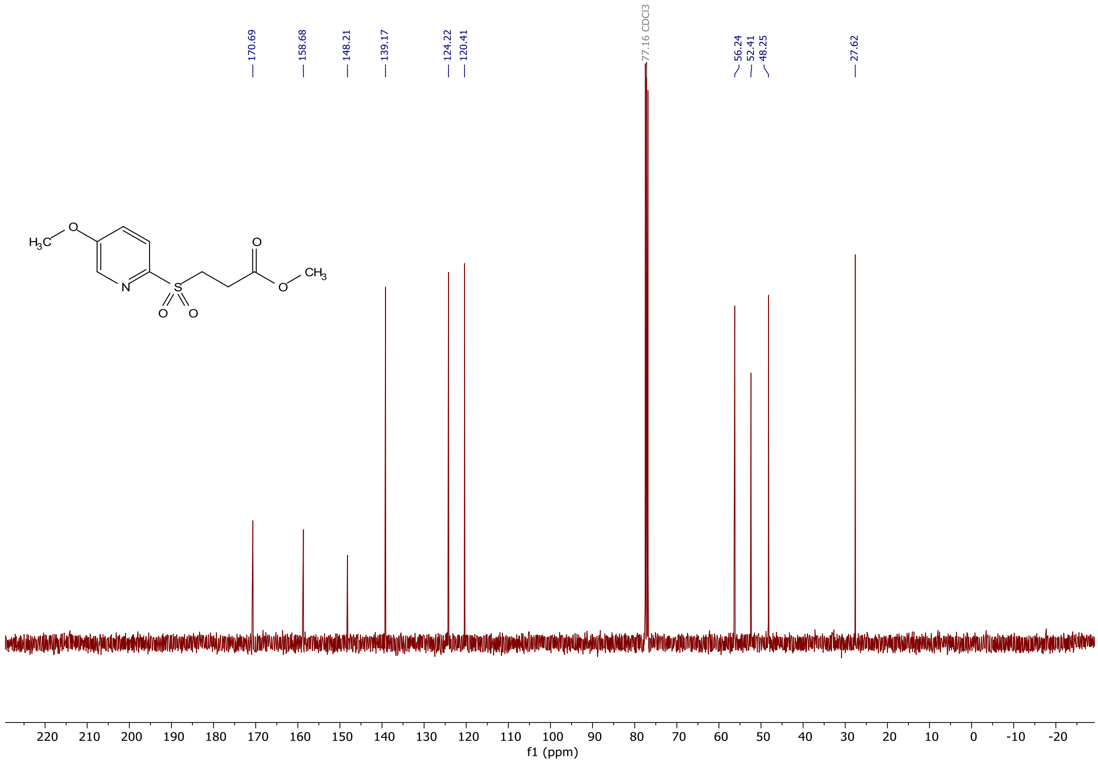

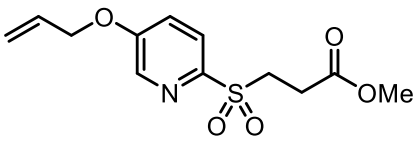

methyl 3-((5-(allyloxy)pyridin-2-yl)sulfonyl)propanoate (6)

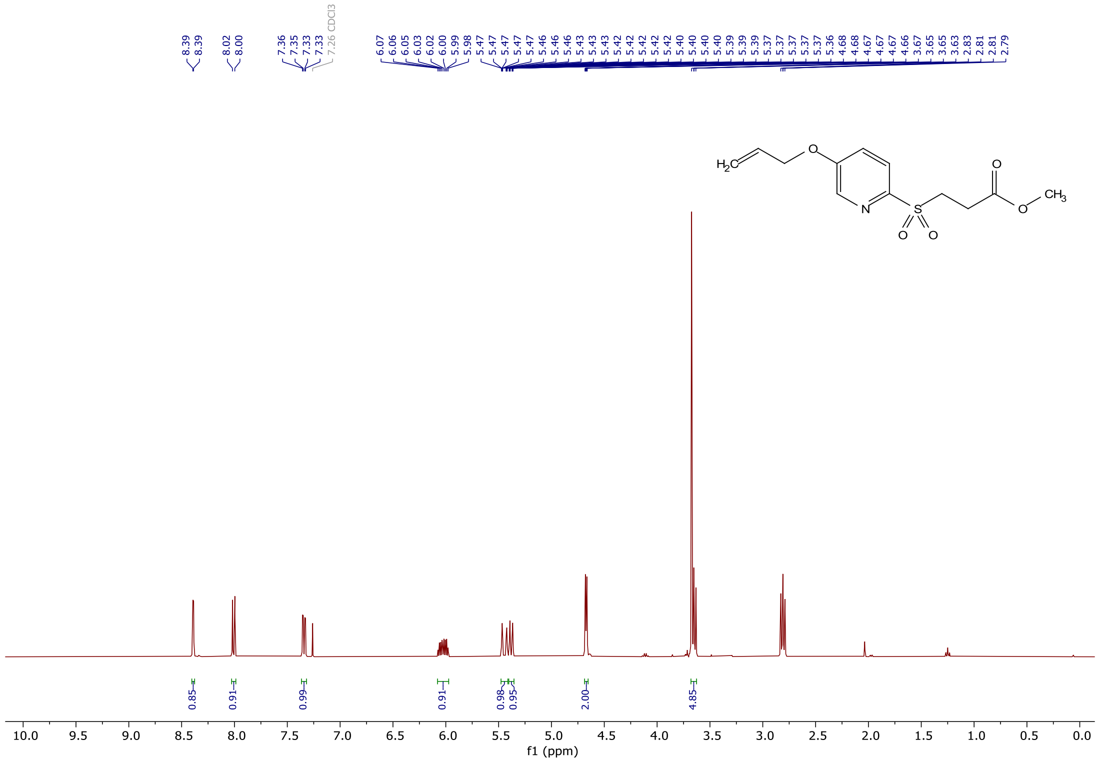

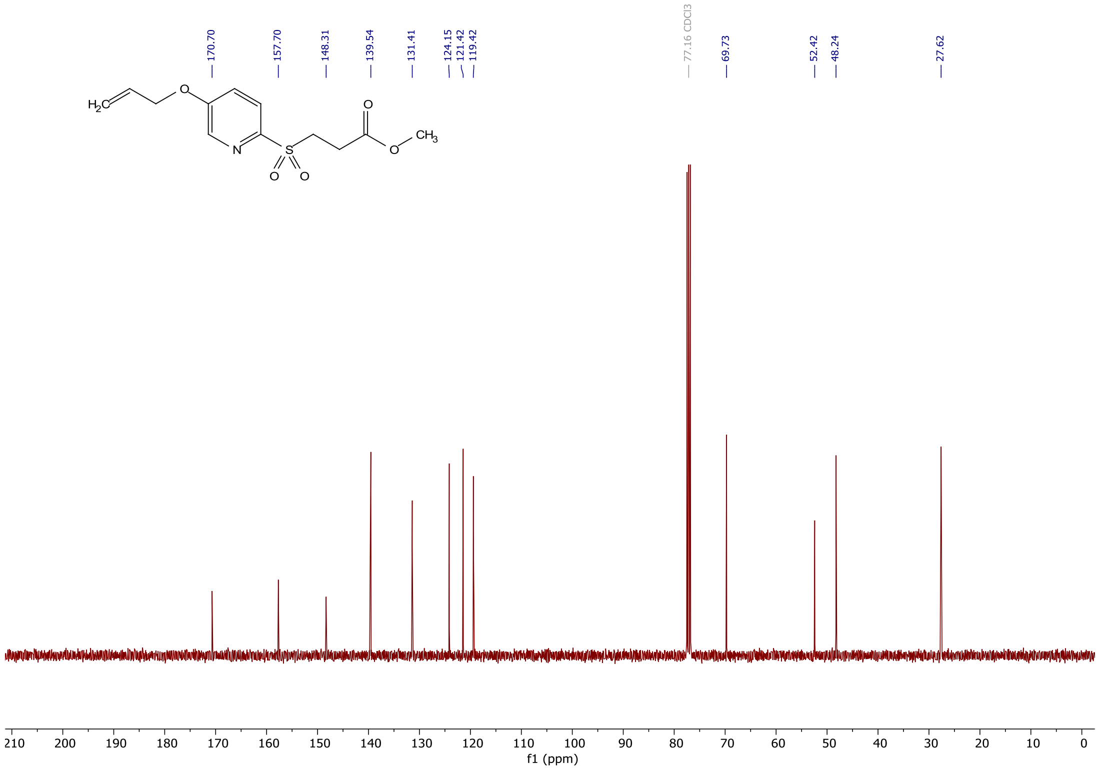

4.3 NMR Spectra of metal sulfinates

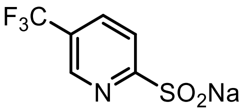

sodium 5-(trifluoromethyl)pyridine-2-sulfinate (15a)

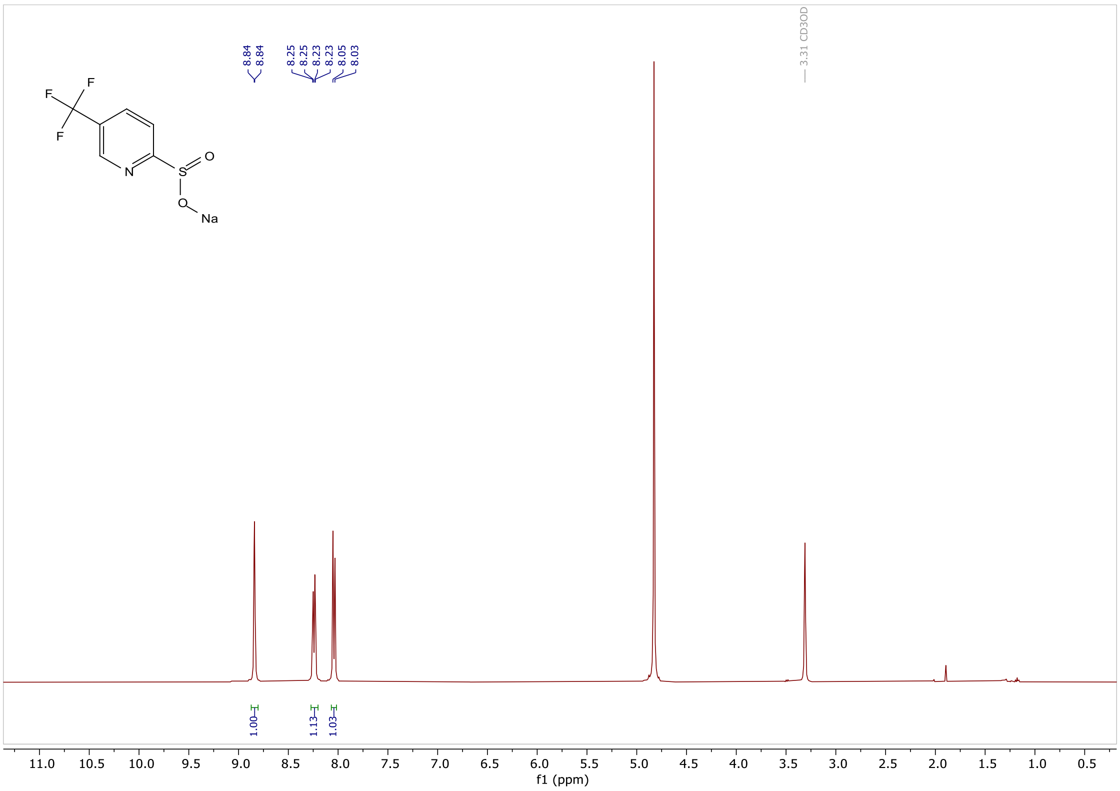

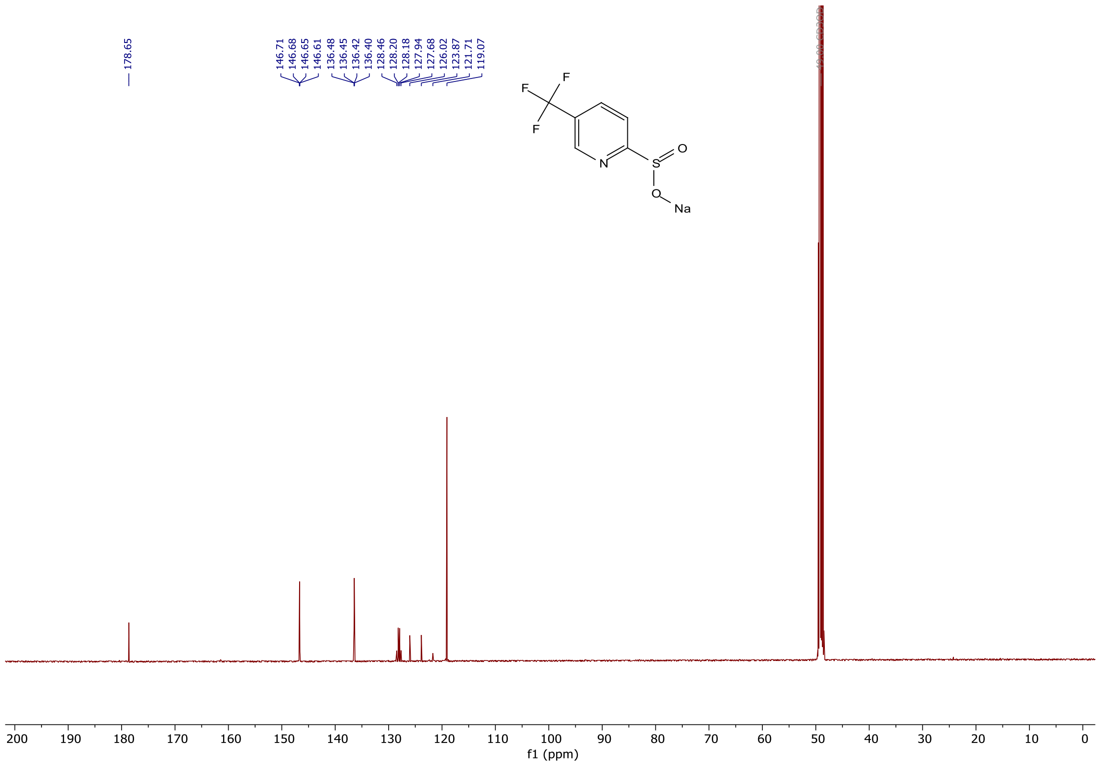
178.65
146.71
146.68
146.65
146.61
136.48
136.45
136.42
136.40
128.46
128.20
128.18
127.94
127.68
126.02
123.87
121.71
119.07
F
F
F
N
S
O
O
Na
200 190 180 170 160 150 140 130 120 110 100 90 80 70 60 50 40 30 20 10 0
f1 (ppm)

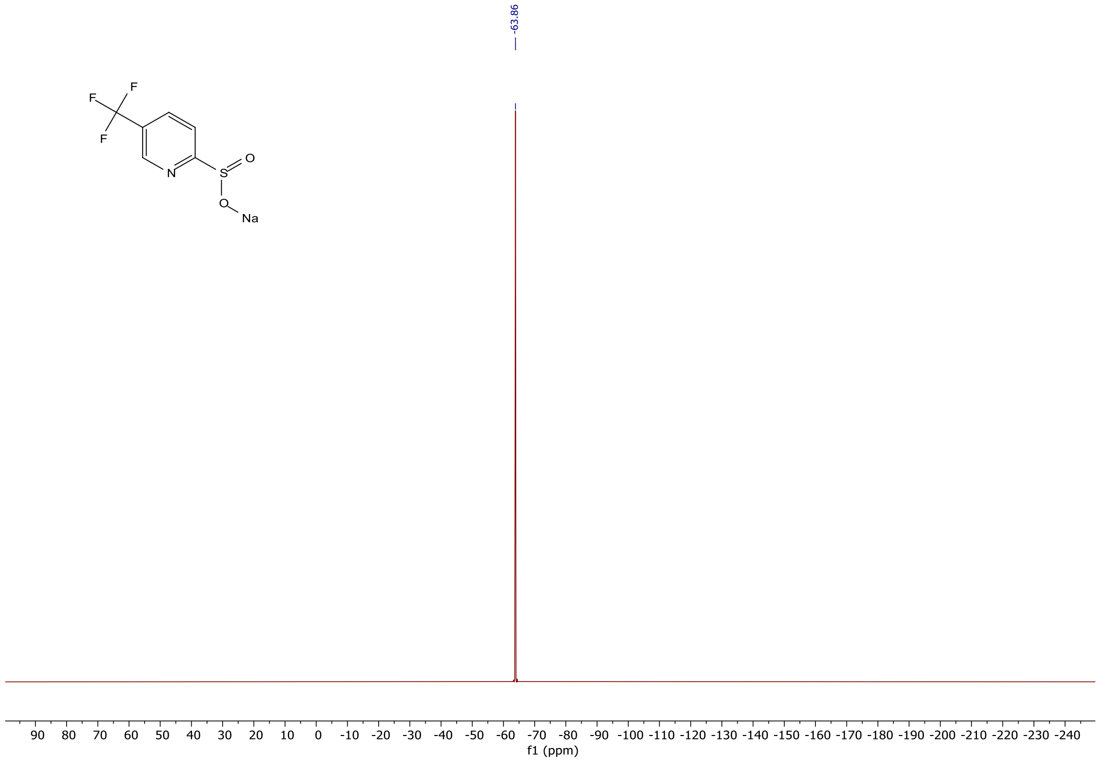
-63.86
F
F
F
N
S
O
O
Na
90 80 70 60 50 40 30 20 10 0 -10 -20 -30 -40 -50 -60 -70 -80 -90 -100 -110 -120 -130 -140 -150 -160 -170 -180 -190 -200 -210 -220 -230 -240
f1 (ppm)

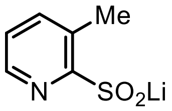

lithium 3-methylpyridine-2-sulfinate (15b)

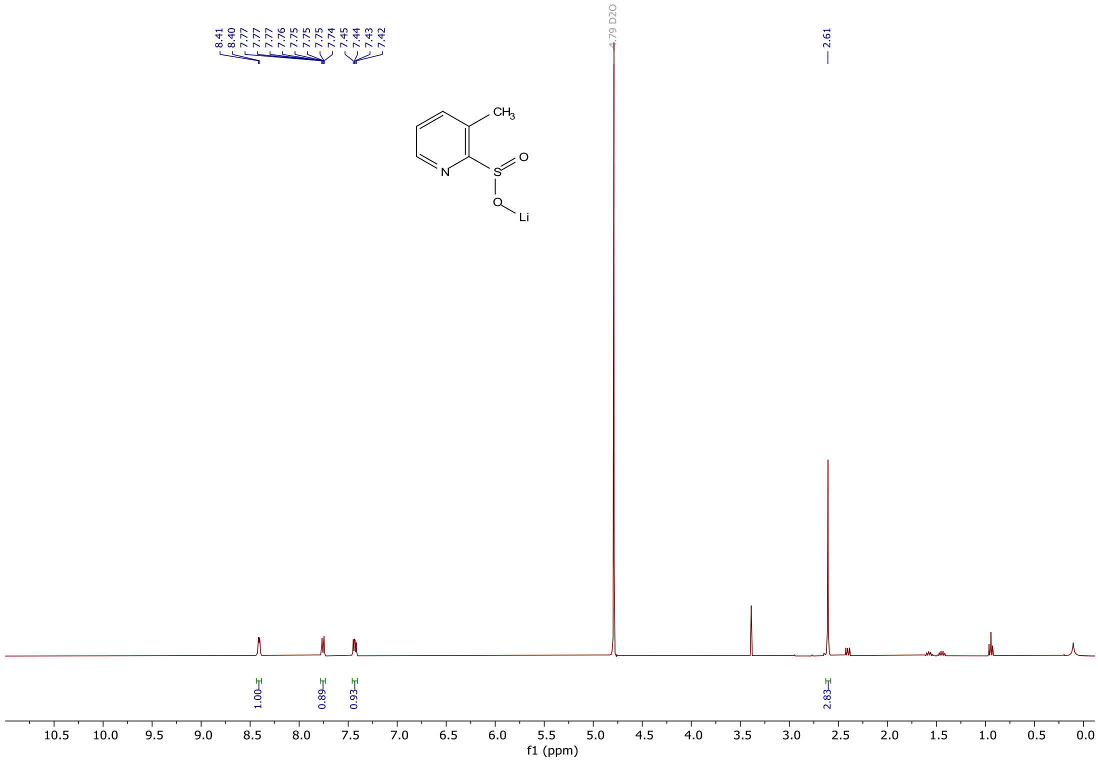

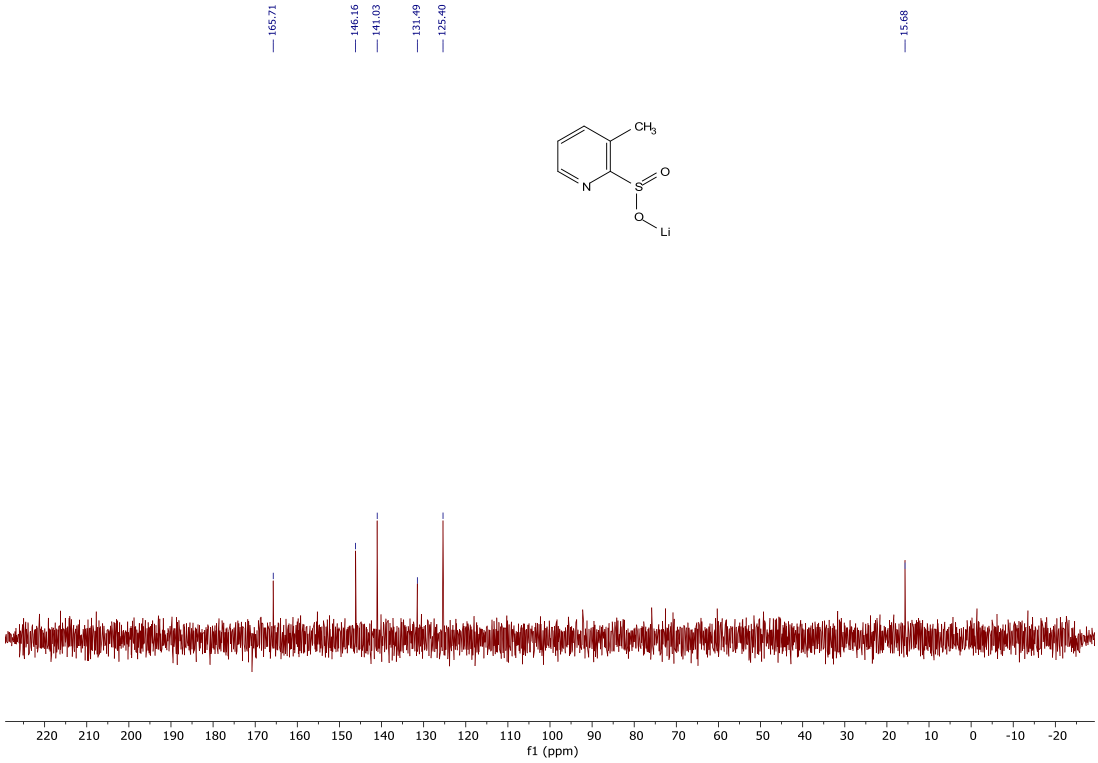

4.4 NMR Spectra of cross-coupled products

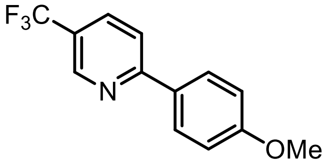

2-(4-methoxyphenyl)-5-(trifluoromethyl)pyridine (3a)

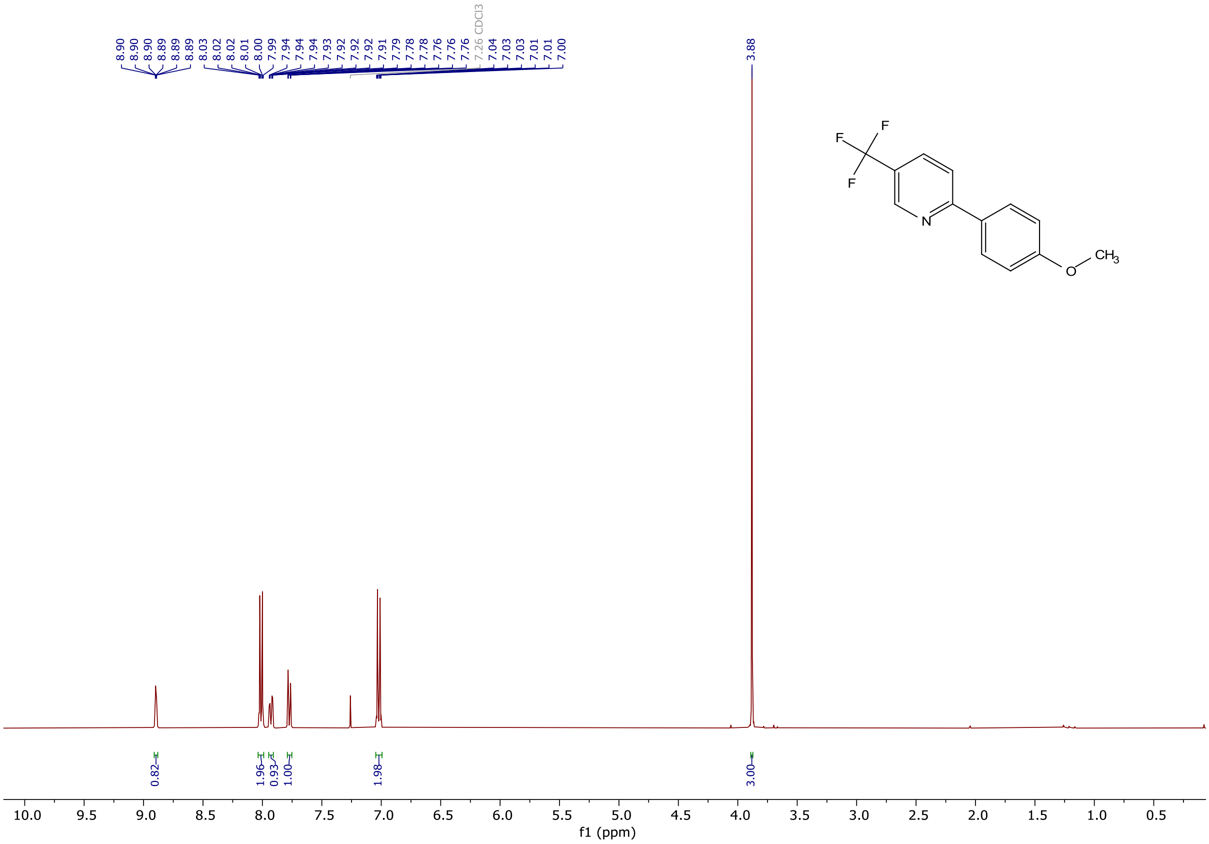

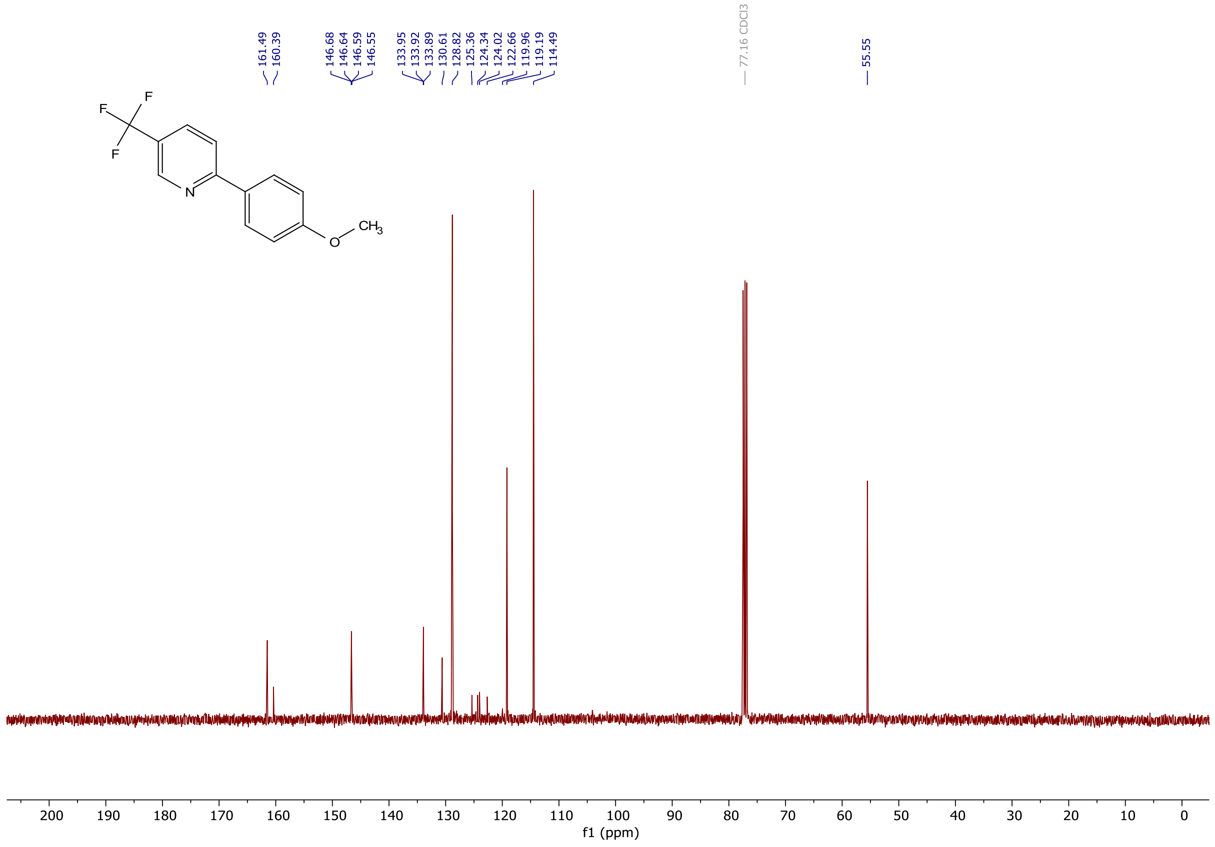
161.49
160.39
146.68
146.64
146.59
146.55
133.95
133.92
133.89
130.61
128.82
125.36
124.34
124.02
122.66
119.96
119.19
114.49
77.16 CDCl3
55.55
F
F
F
N
O
CH3
200 190 180 170 160 150 140 130 120 110 100 90 80 70 60 50 40 30 20 10 0
f1 (ppm)

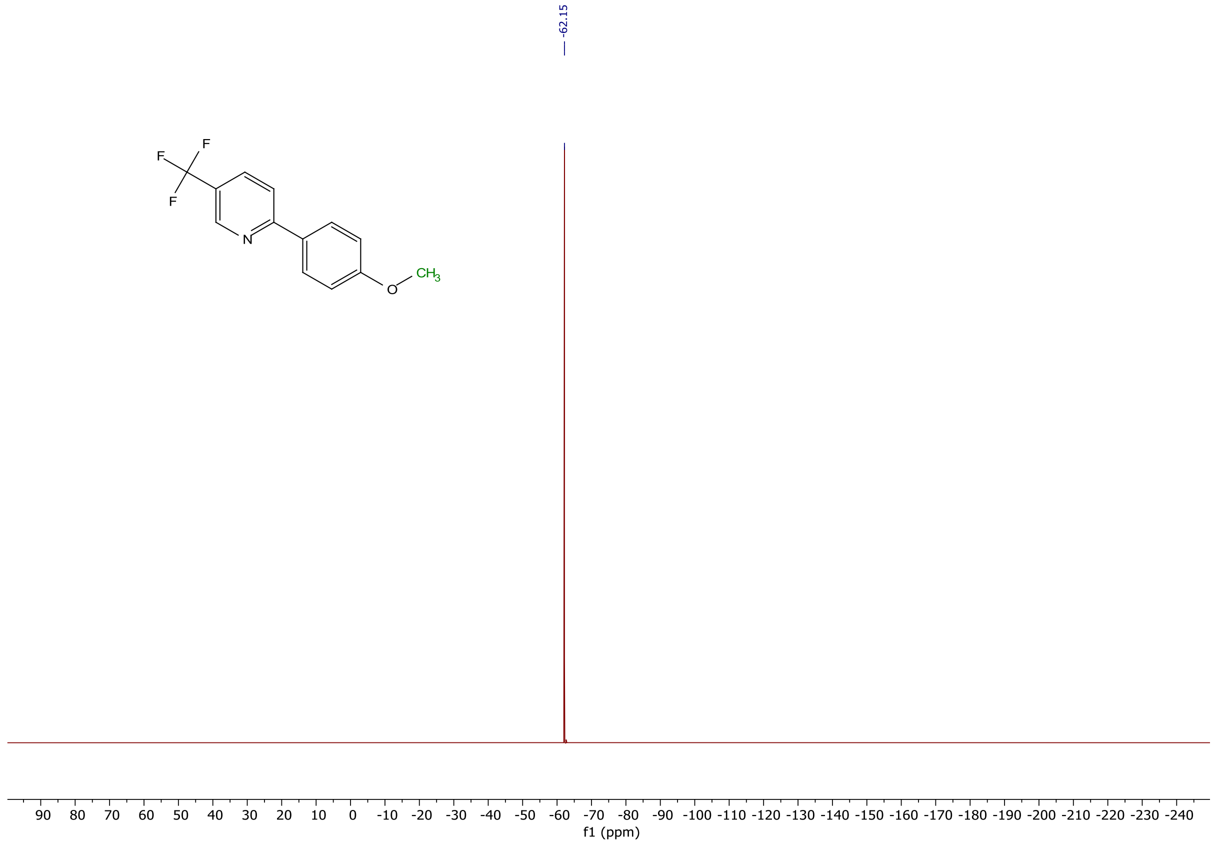
-62.15
F
F
F
N
O
CH3
90 80 70 60 50 40 30 20 10 0 -10 -20 -30 -40 -50 -60 -70 -80 -90 -100 -110 -120 -130 -140 -150 -160 -170 -180 -190 -200 -210 -220 -230 -240
f1 (ppm)

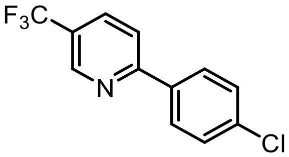

2-(4-chlorophenyl)-5-(trifluoromethyl)pyridine (3b)

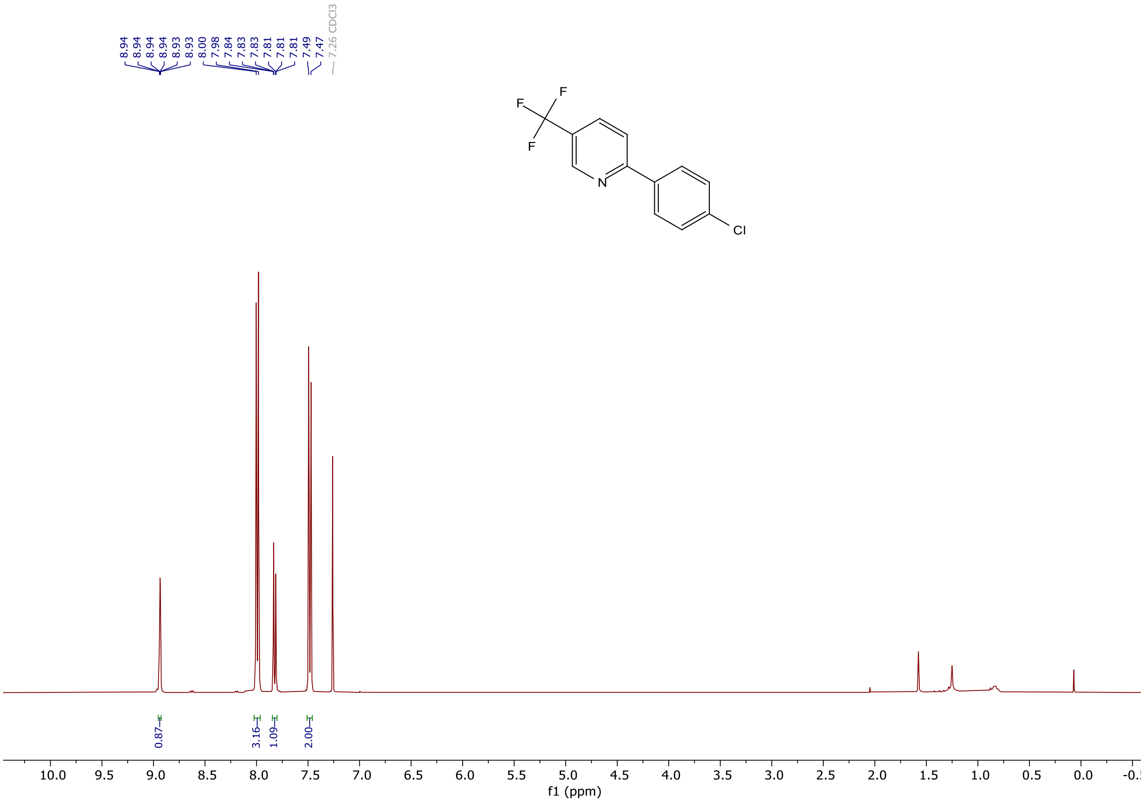

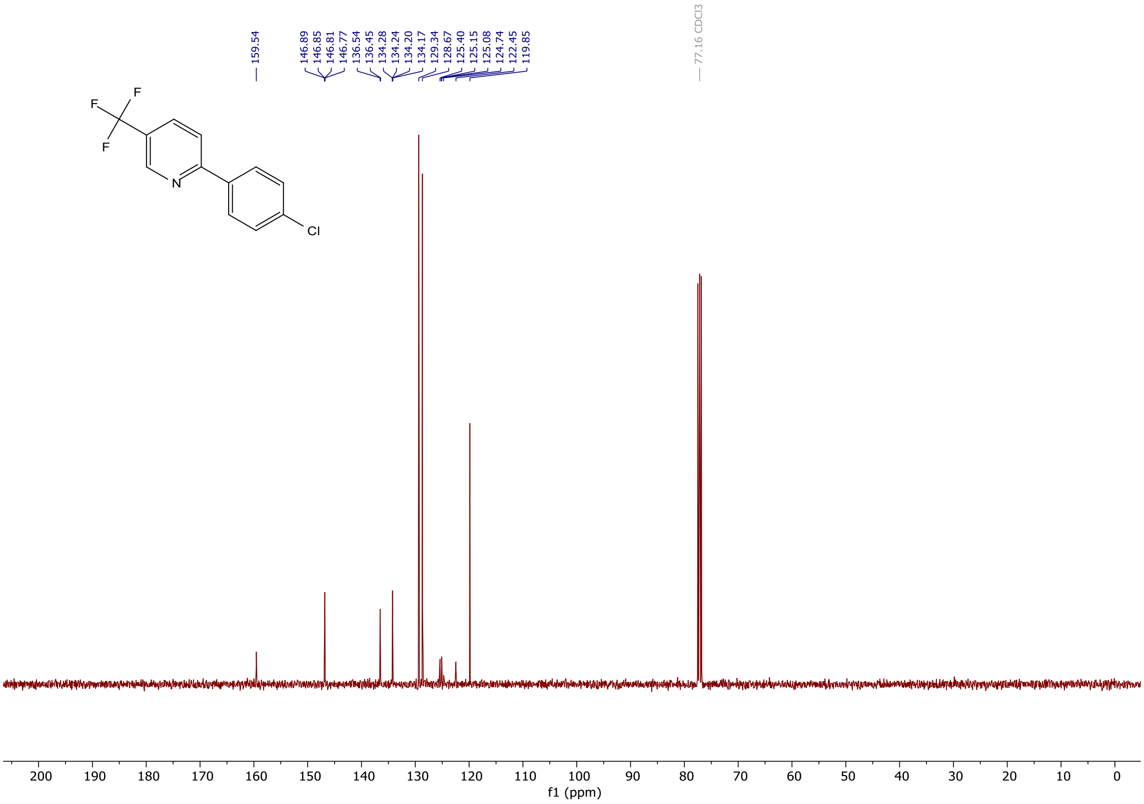

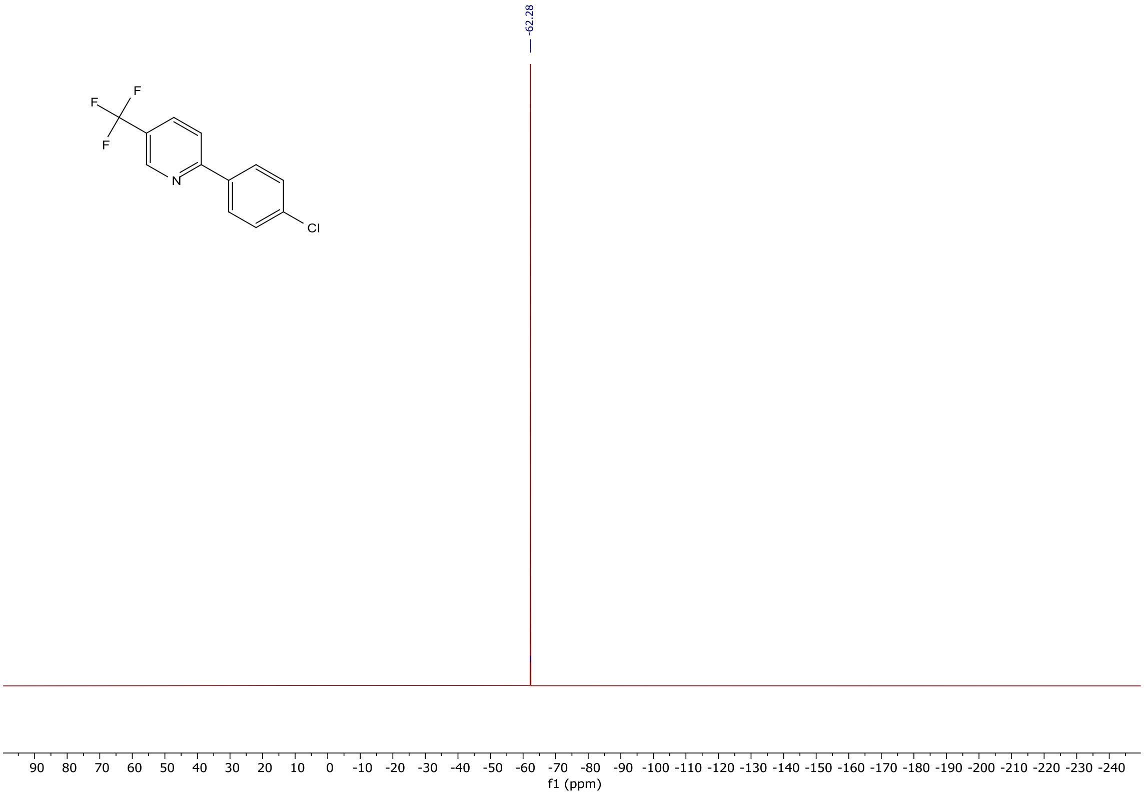

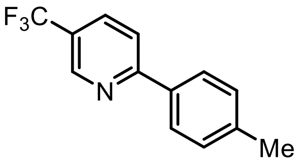

2-(p-tolyl)-5-(trifluoromethyl)pyridine (3c)

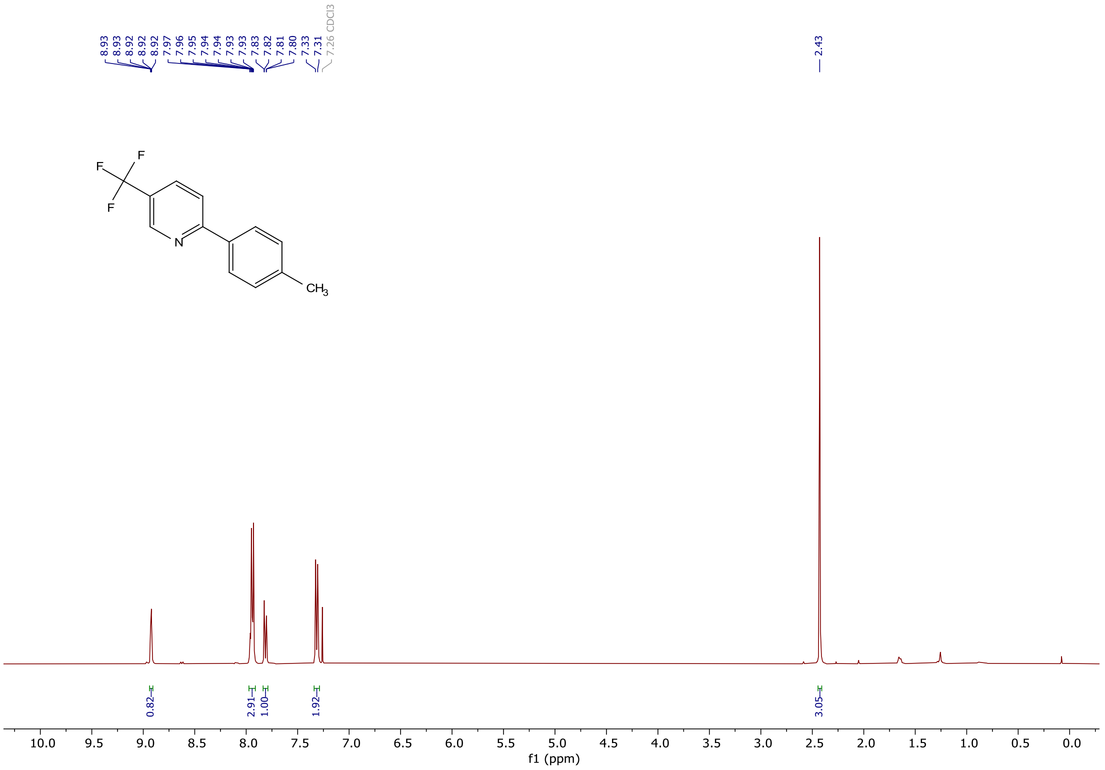

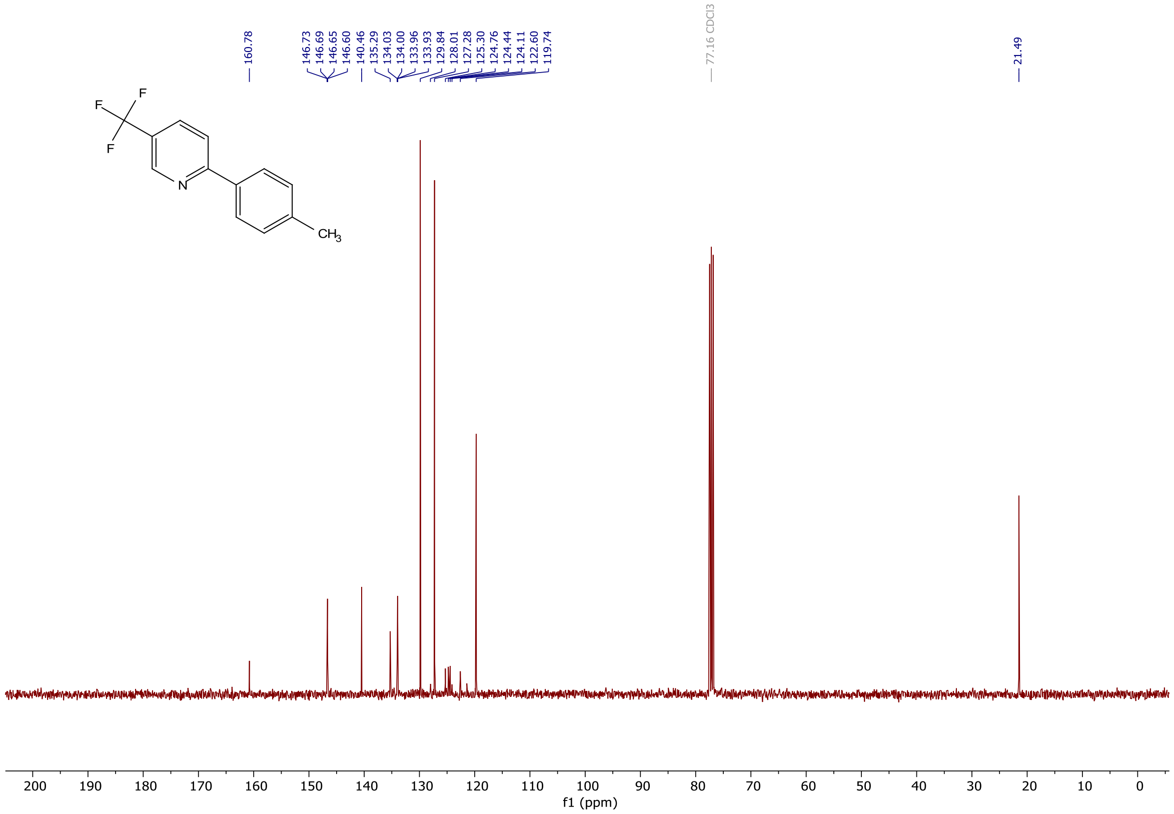
F
F
F
N
CH3
160.78
146.73
146.69
146.65
146.60
140.46
135.29
134.03
134.00
133.96
133.93
129.84
128.01
127.28
125.30
124.76
124.44
124.11
122.60
119.74
77.16 CDCl3
21.49
200 190 180 170 160 150 140 130 120 110 100 90 80 70 60 50 40 30 20 10 0
f1 (ppm)

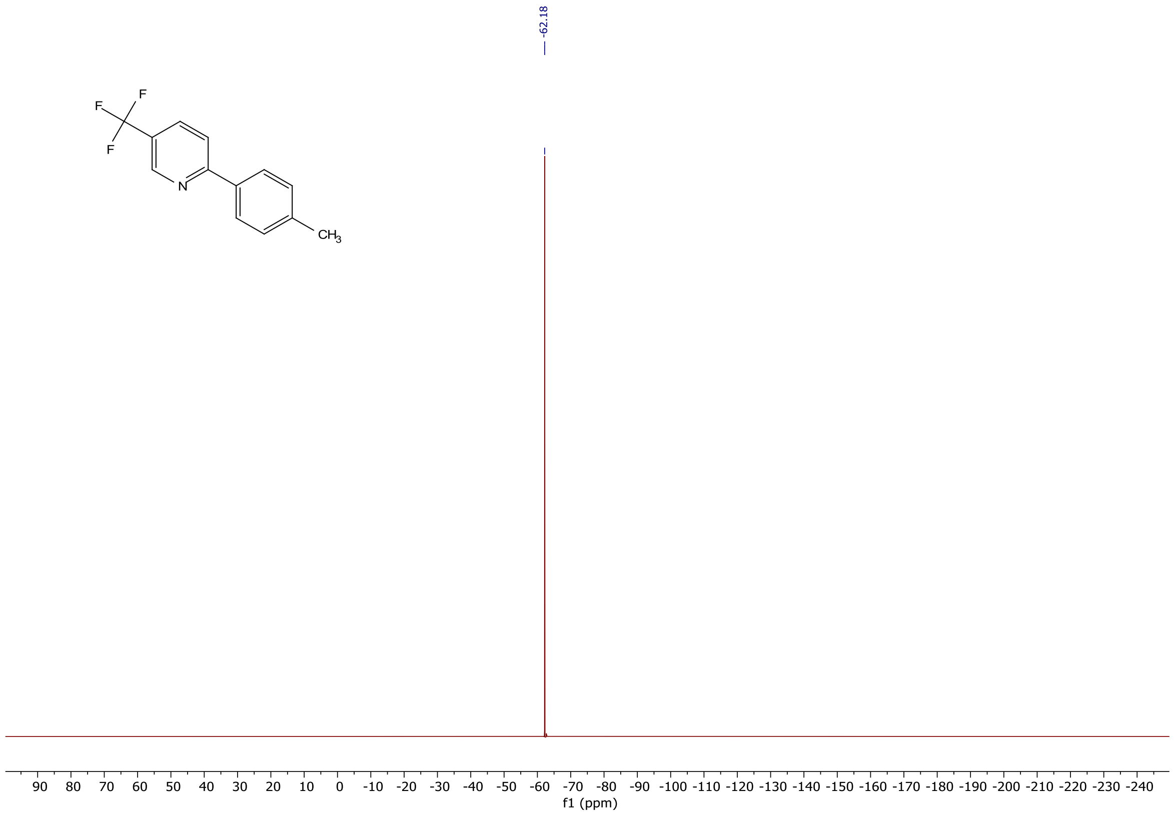
F
F
F
N
CH3
-62.18
90 80 70 60 50 40 30 20 10 0 -10 -20 -30 -40 -50 -60 -70 -80 -90 -100 -110 -120 -130 -140 -150 -160 -170 -180 -190 -200 -210 -220 -230 -240
f1 (ppm)

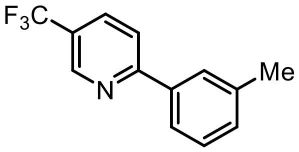

2-(m-tolyl)-5-(trifluoromethyl)pyridine (3d)

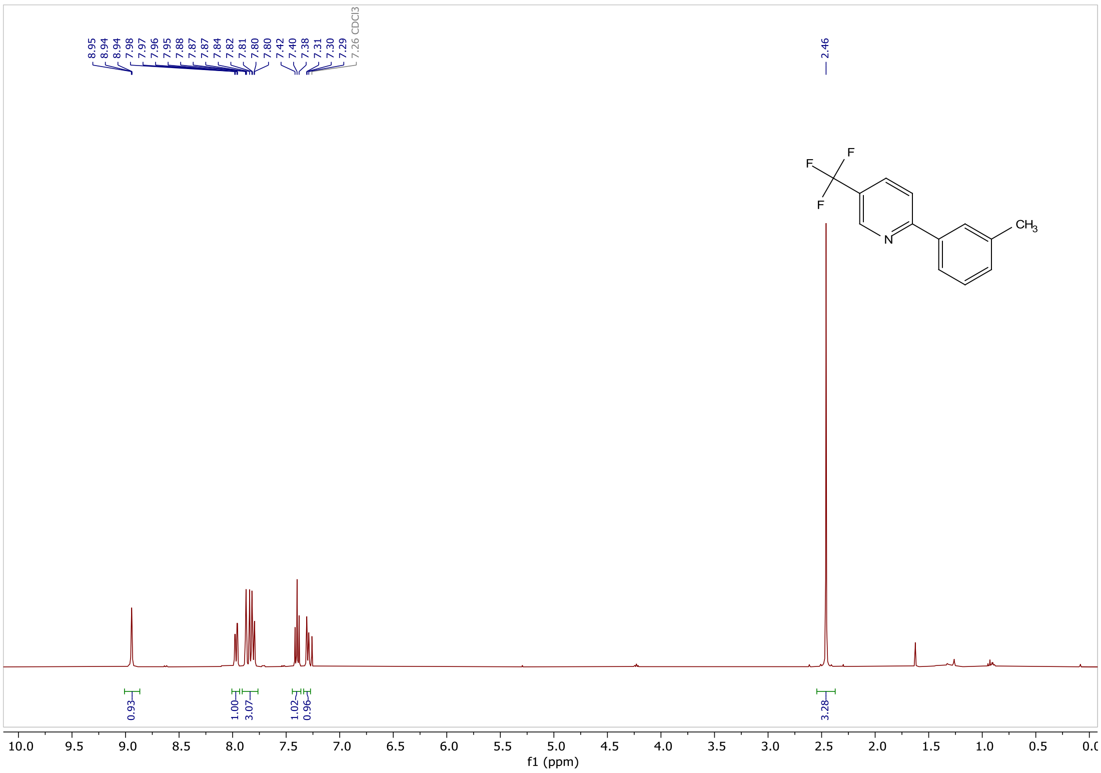

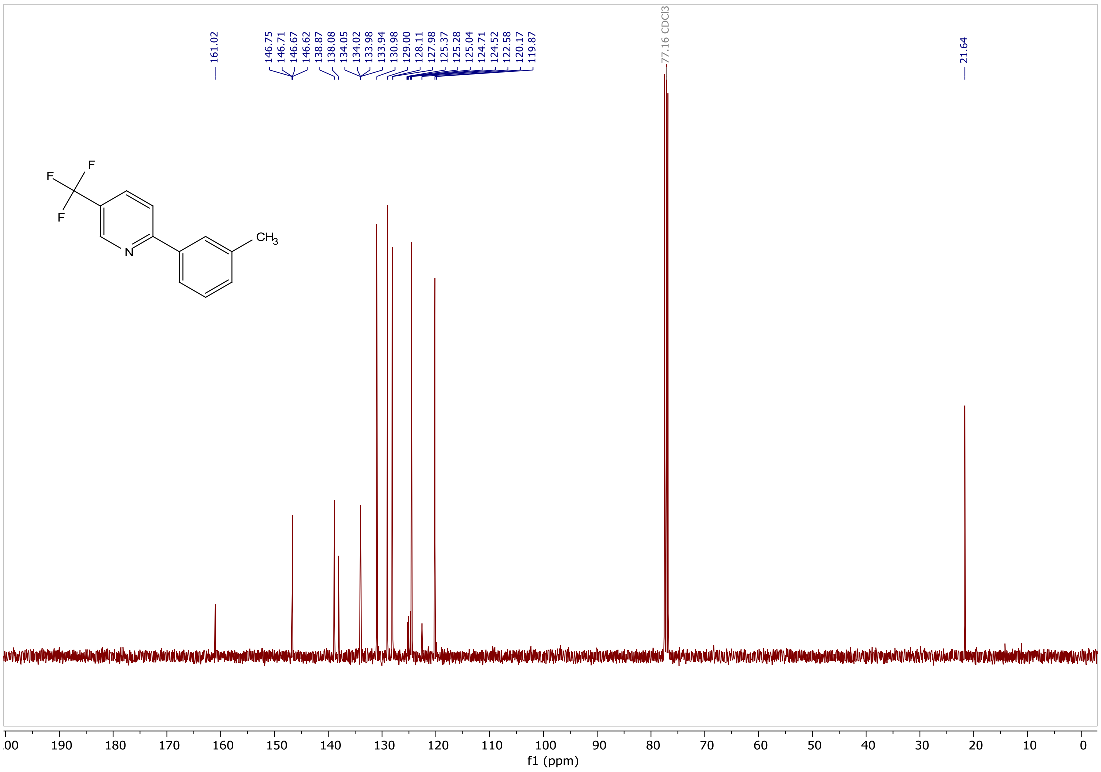

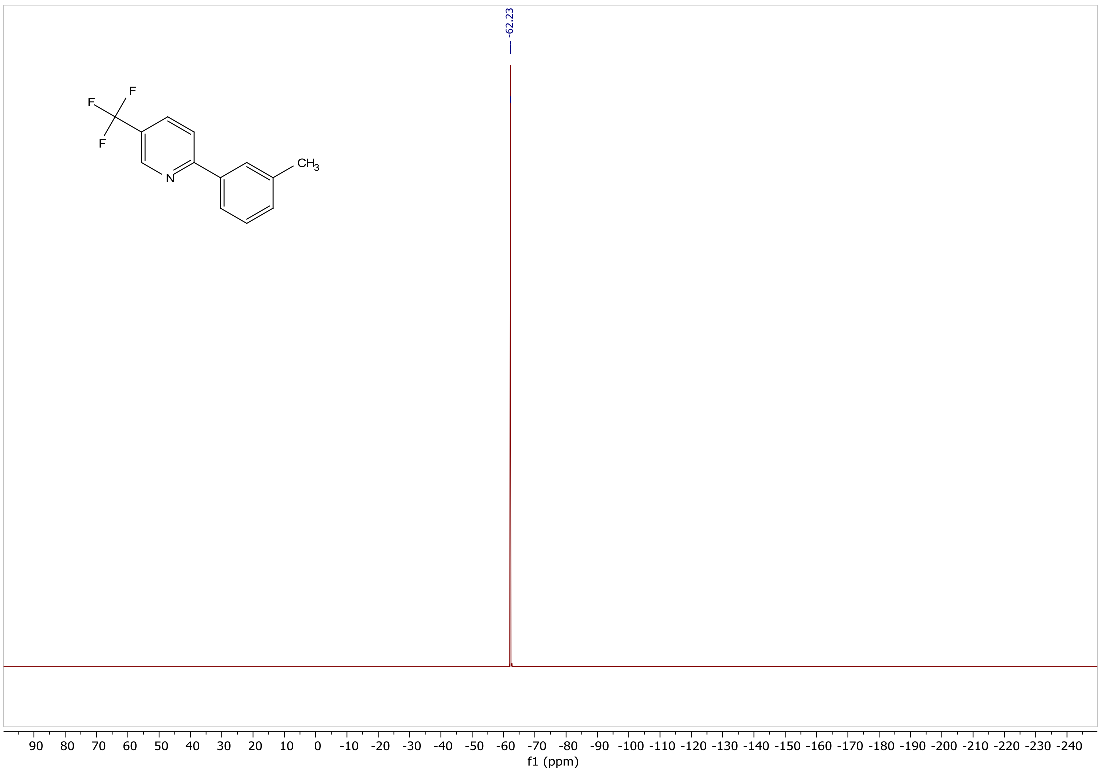
-62.23
F
F
F
N
CH3
90 80 70 60 50 40 30 20 10 0 -10 -20 -30 -40 -50 -60 -70 -80 -90 -100 -110 -120 -130 -140 -150 -160 -170 -180 -190 -200 -210 -220 -230 -240
f1 (ppm)

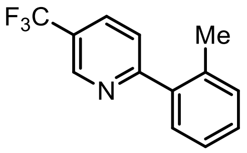

2-(o-tolyl)-5-(trifluoromethyl)pyridine (3e)

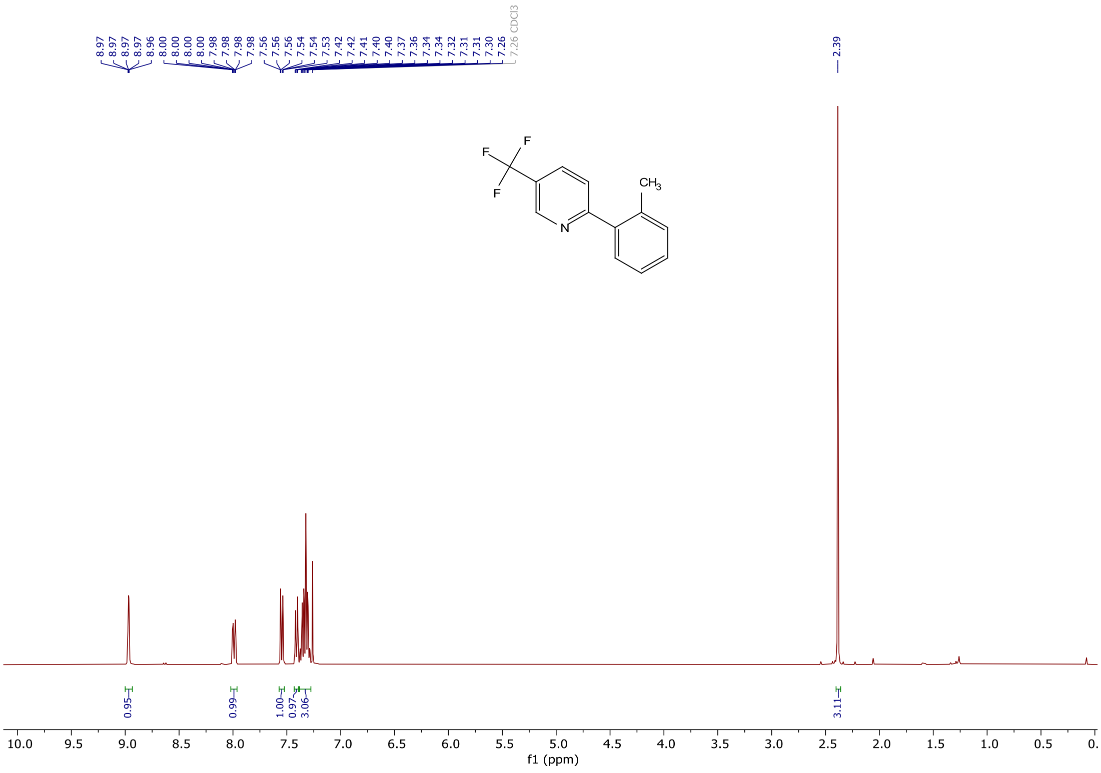

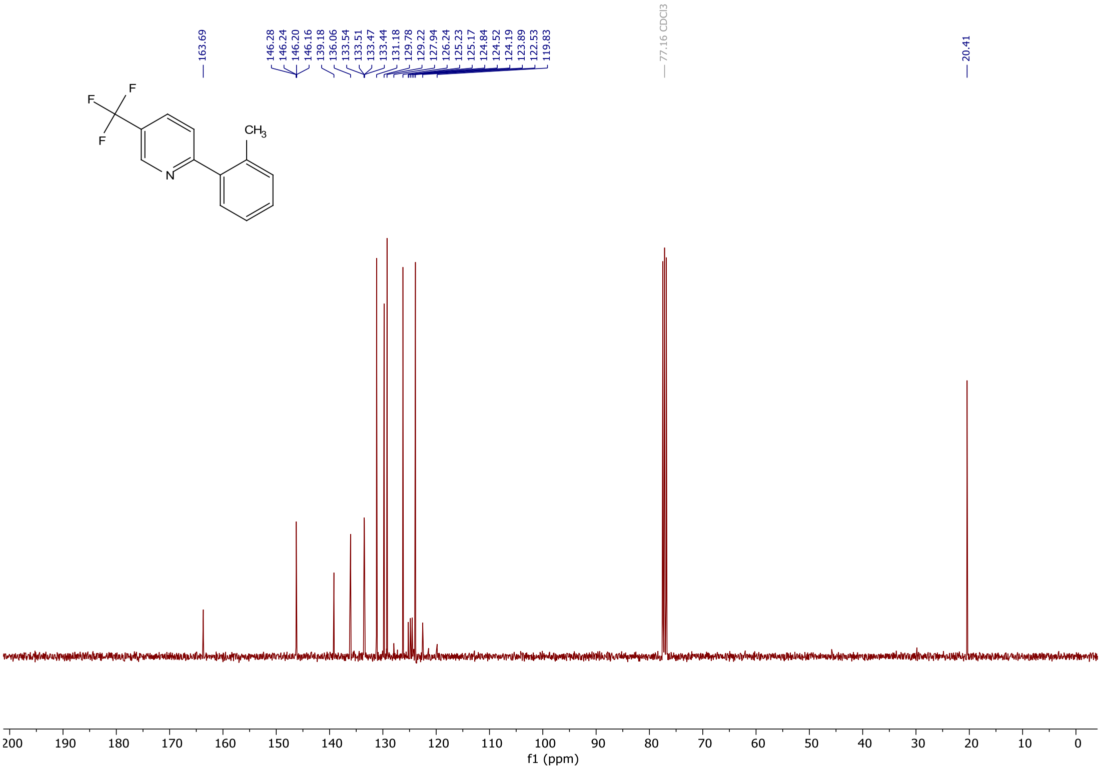

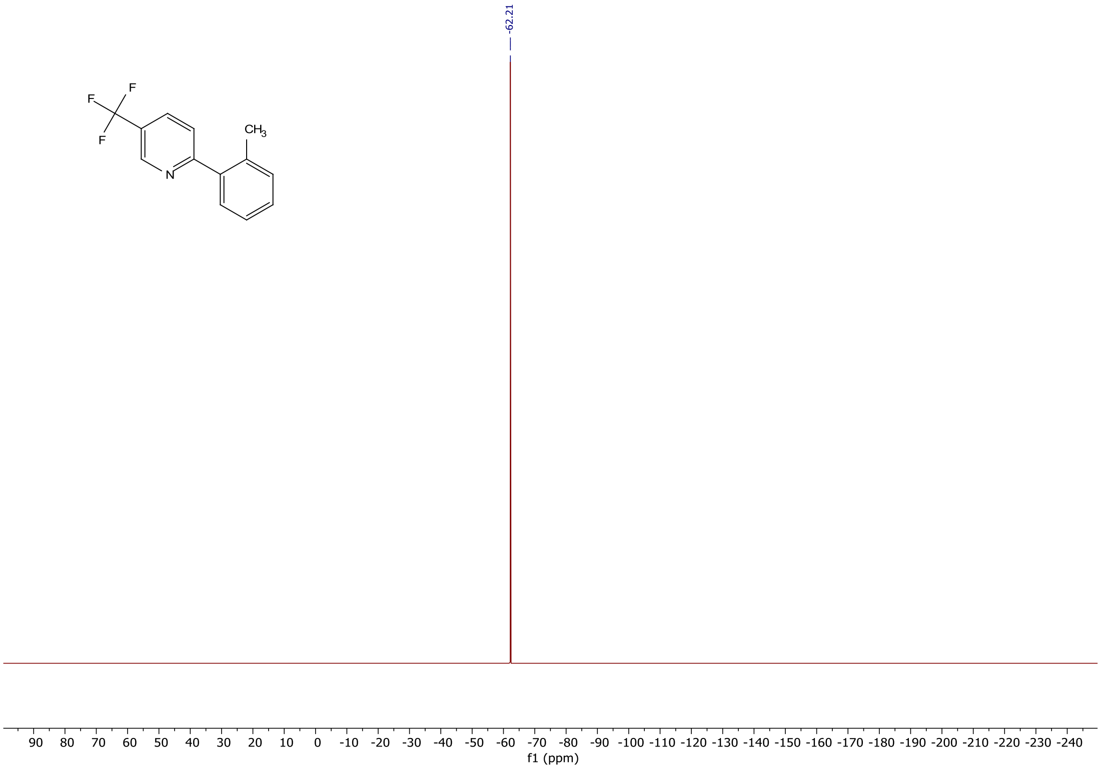

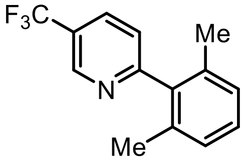

2-(2,6-dimethylphenyl)-5-(trifluoromethyl)pyridine (3f)

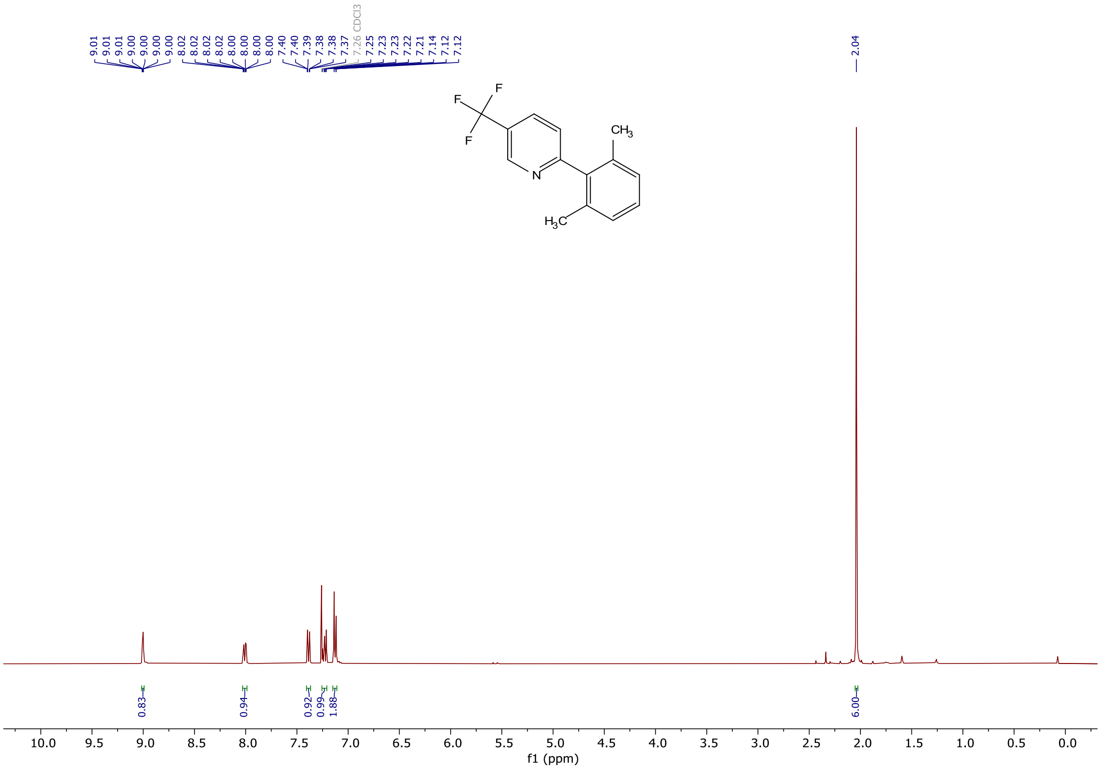

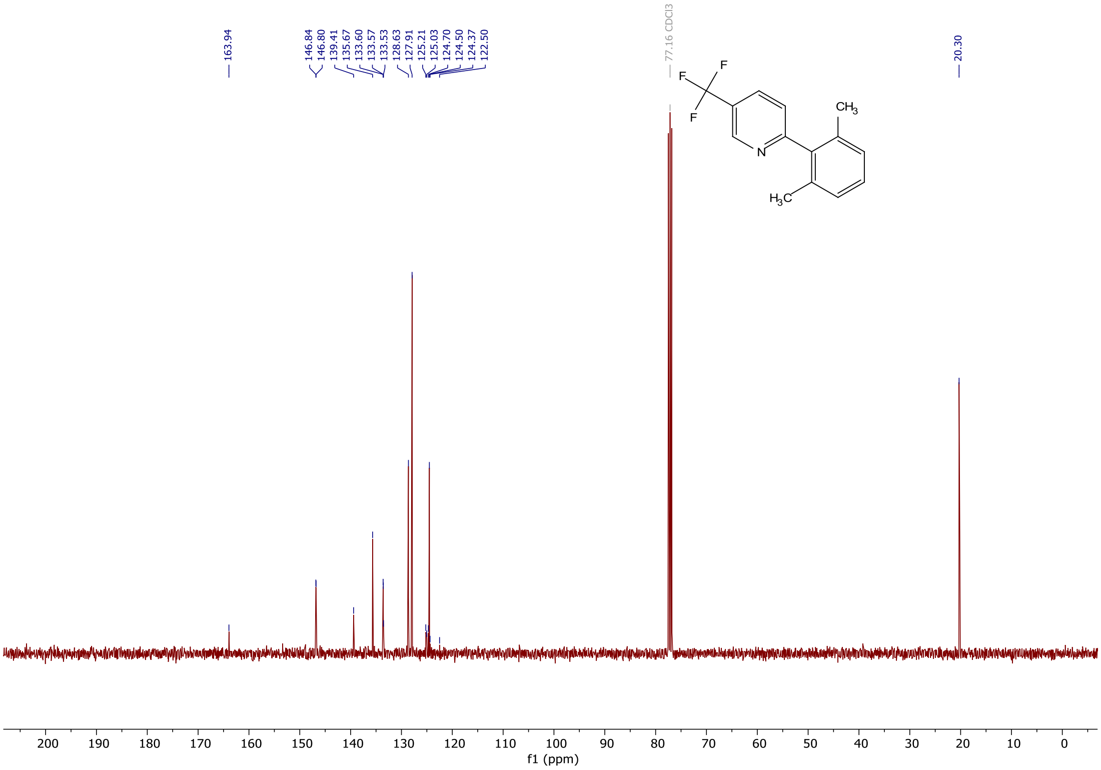

163.94
146.84
146.80
139.41
135.67
133.60
133.57
133.53
128.63
127.91
125.21
125.03
124.70
124.50
124.37
122.50
77.16 CDCl3
20.30
F
F
F
N
CH3
H3C
200 190 180 170 160 150 140 130 120 110 100 90 80 70 60 50 40 30 20 10 0
f1 (ppm)

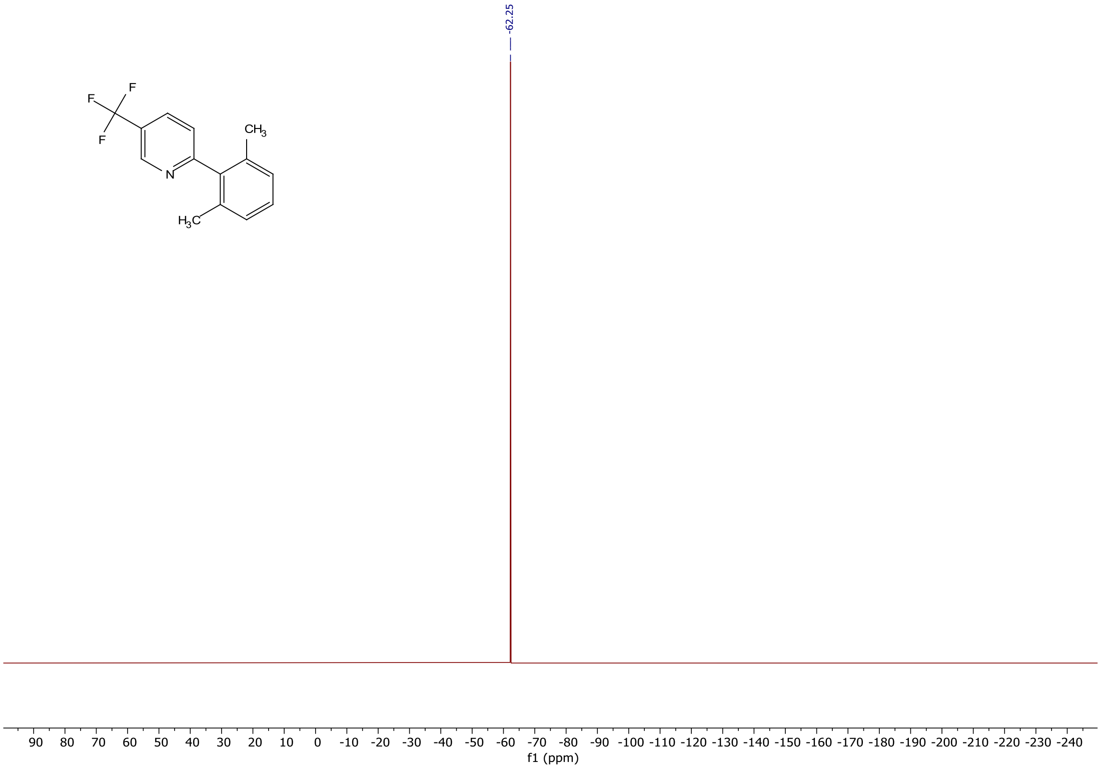

-62.25
F
F
F
N
CH3
H3C
90 80 70 60 50 40 30 20 10 0 -10 -20 -30 -40 -50 -60 -70 -80 -90 -100 -110 -120 -130 -140 -150 -160 -170 -180 -190 -200 -210 -220 -230 -240
f1 (ppm)

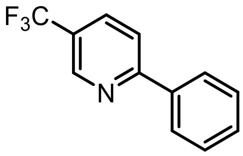

2-phenyl-5-(trifluoromethyl)pyridine (3g)

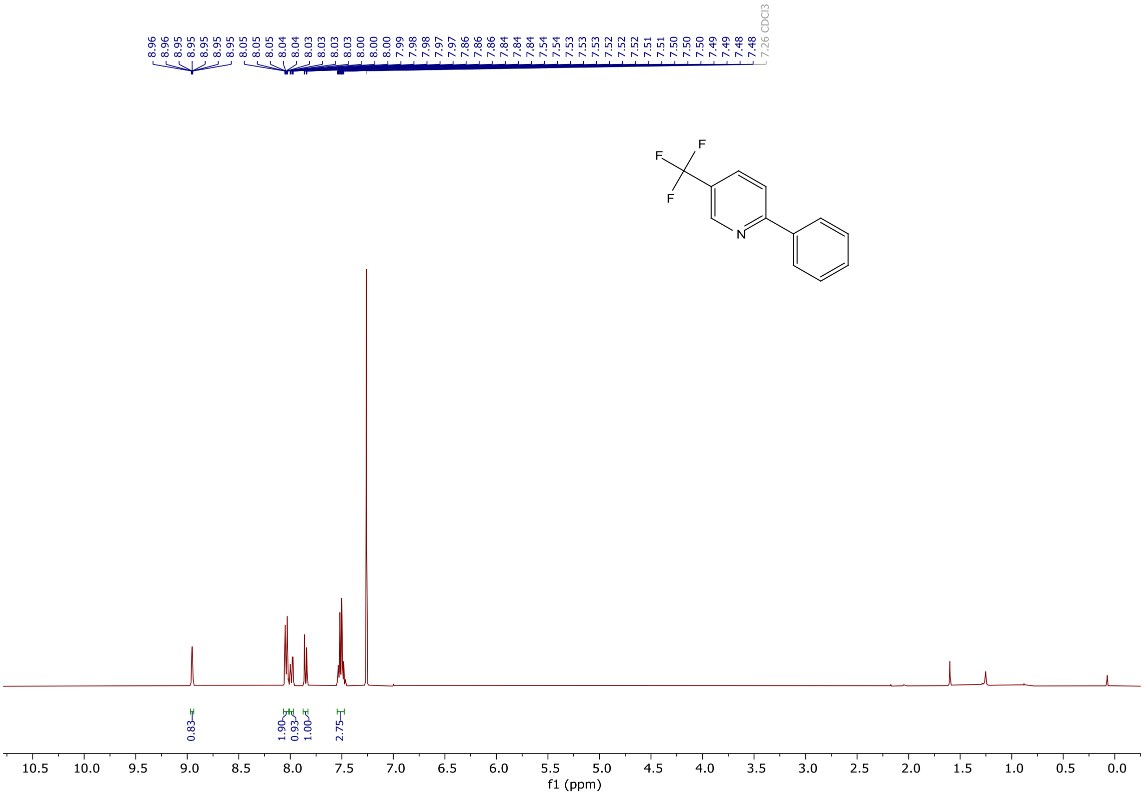

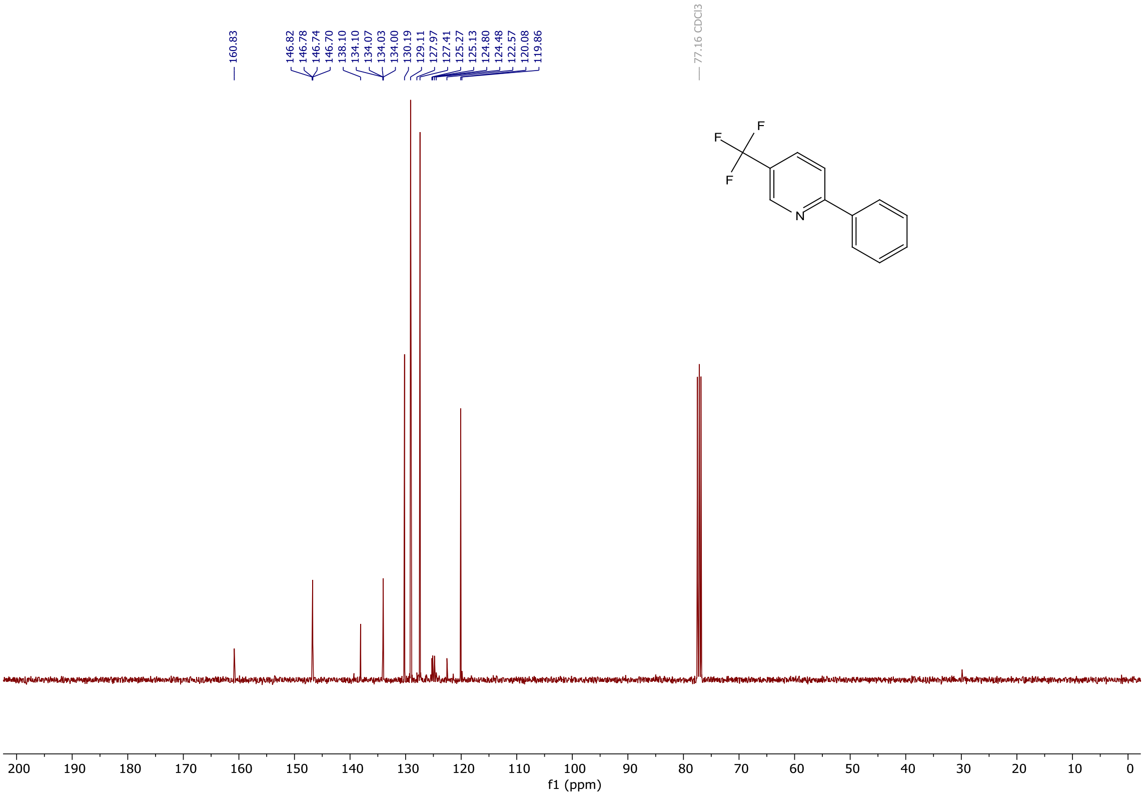

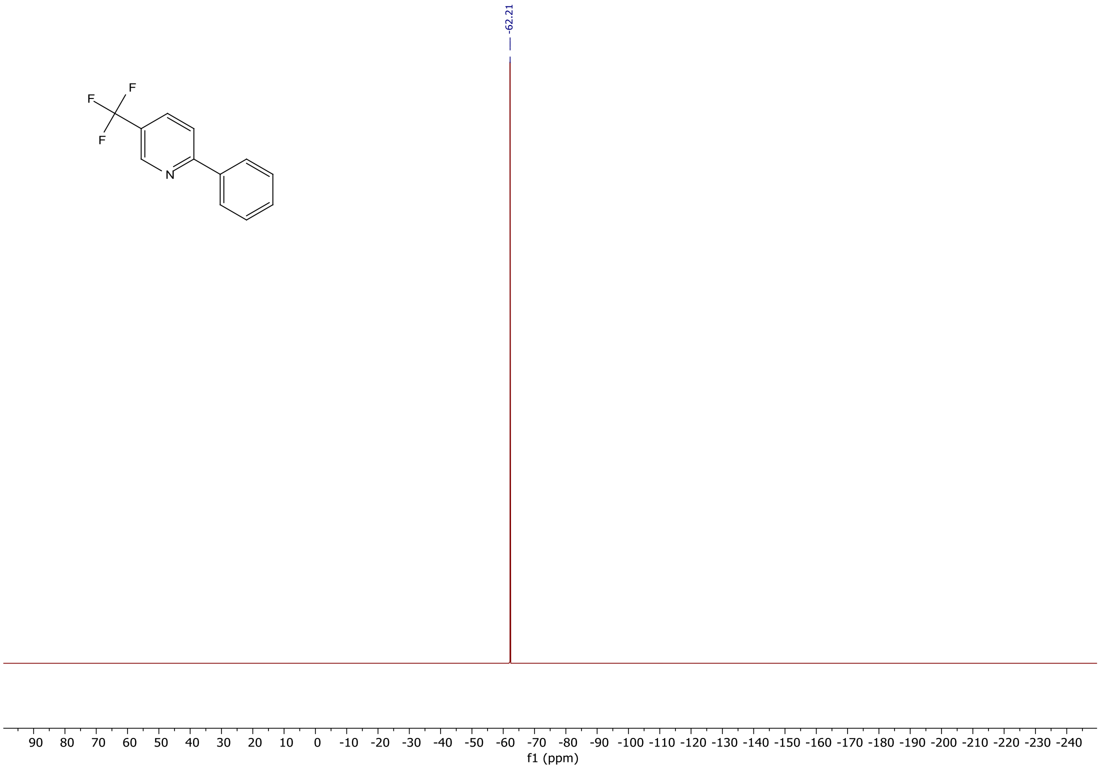

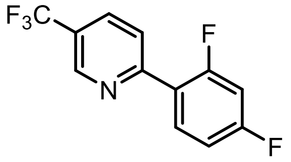

2-(2,4-difluorophenyl)-5-(trifluoromethyl)pyridine (3h)

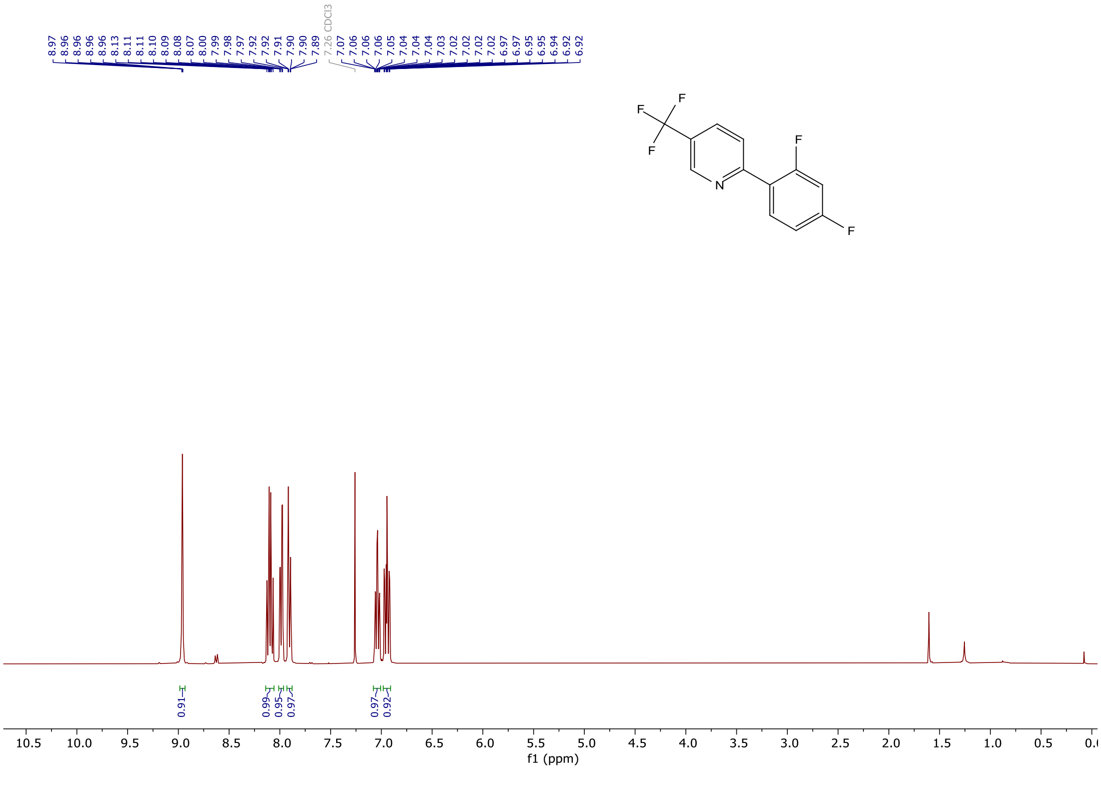

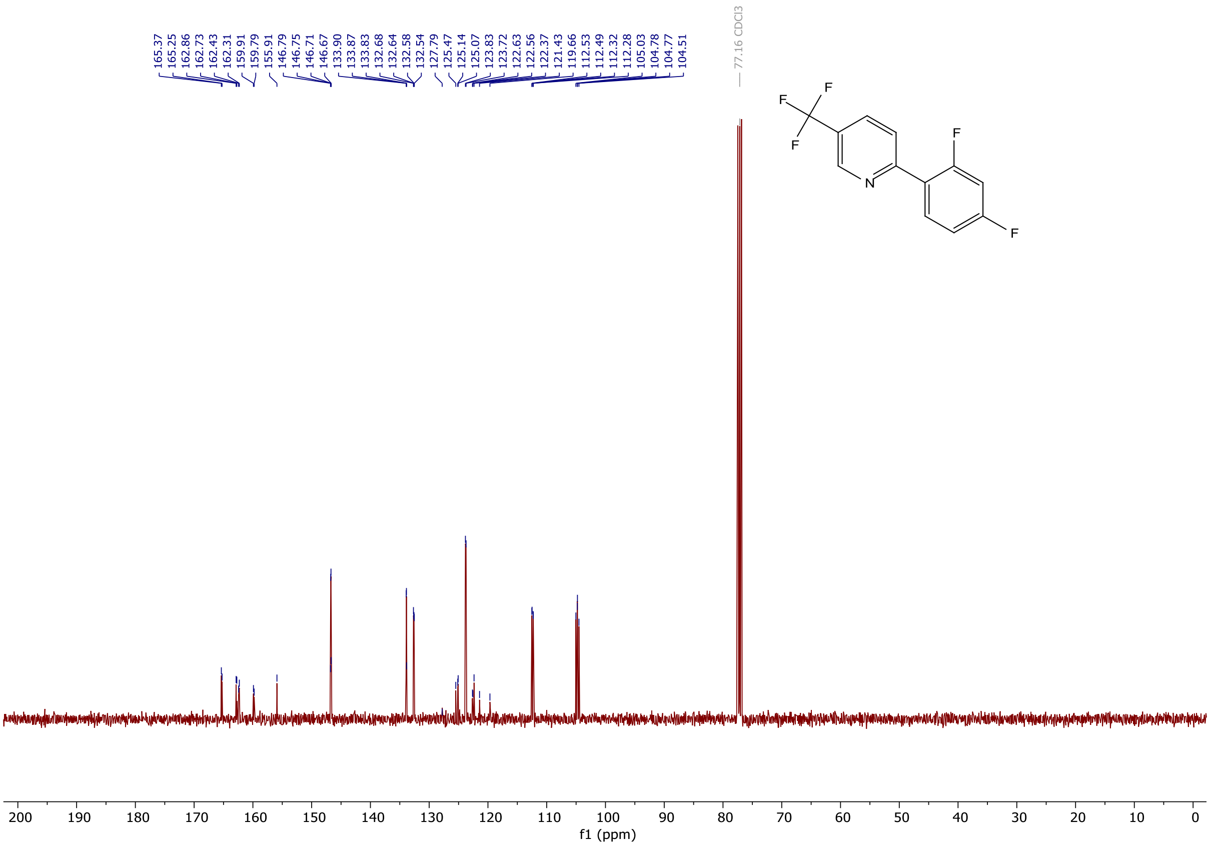
165.37
165.25
162.86
162.73
162.43
162.31
159.91
159.79
155.91
146.79
146.75
146.71
146.67
133.90
133.87
133.83
132.68
132.64
132.58
132.54
127.79
125.47
125.14
125.07
123.83
123.72
122.63
122.56
122.37
121.43
119.66
112.53
112.49
112.32
112.28
105.03
104.78
104.77
104.51
77.16 CDCl3
F
F
F
N
F
F
f1 (ppm)

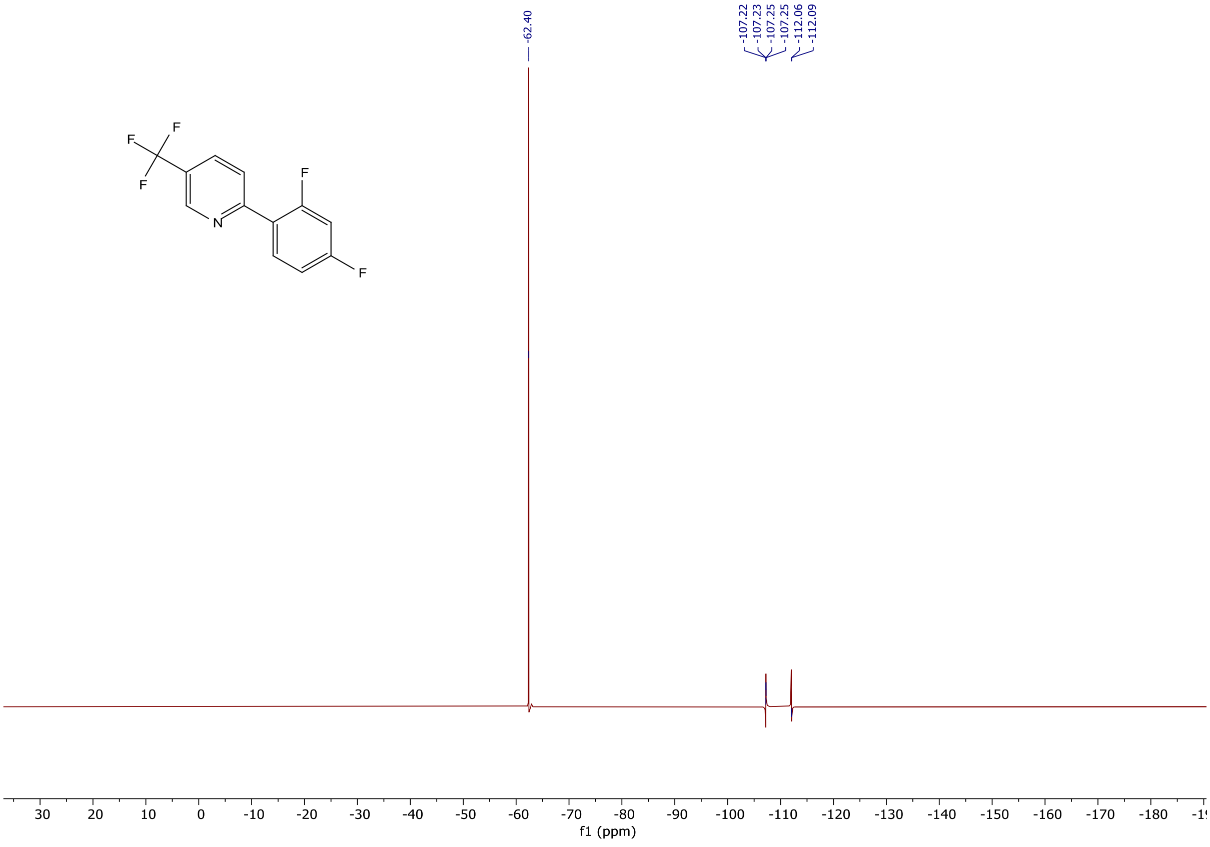
-62.40
-107.22
-107.23
-107.25
-107.25
-112.06
-112.09
F
F
F
N
F
F
f1 (ppm)

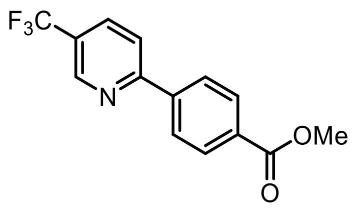

methyl 4-(5-(trifluoromethyl)pyridin-2-yl)benzoate (3i)

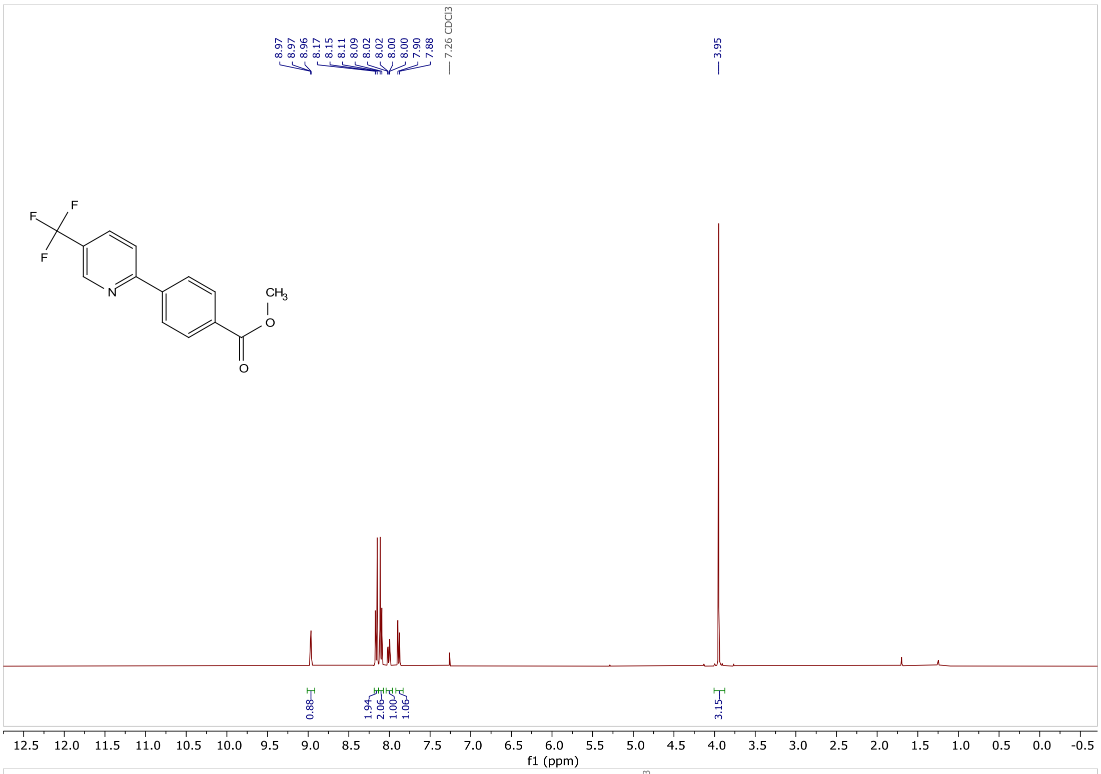

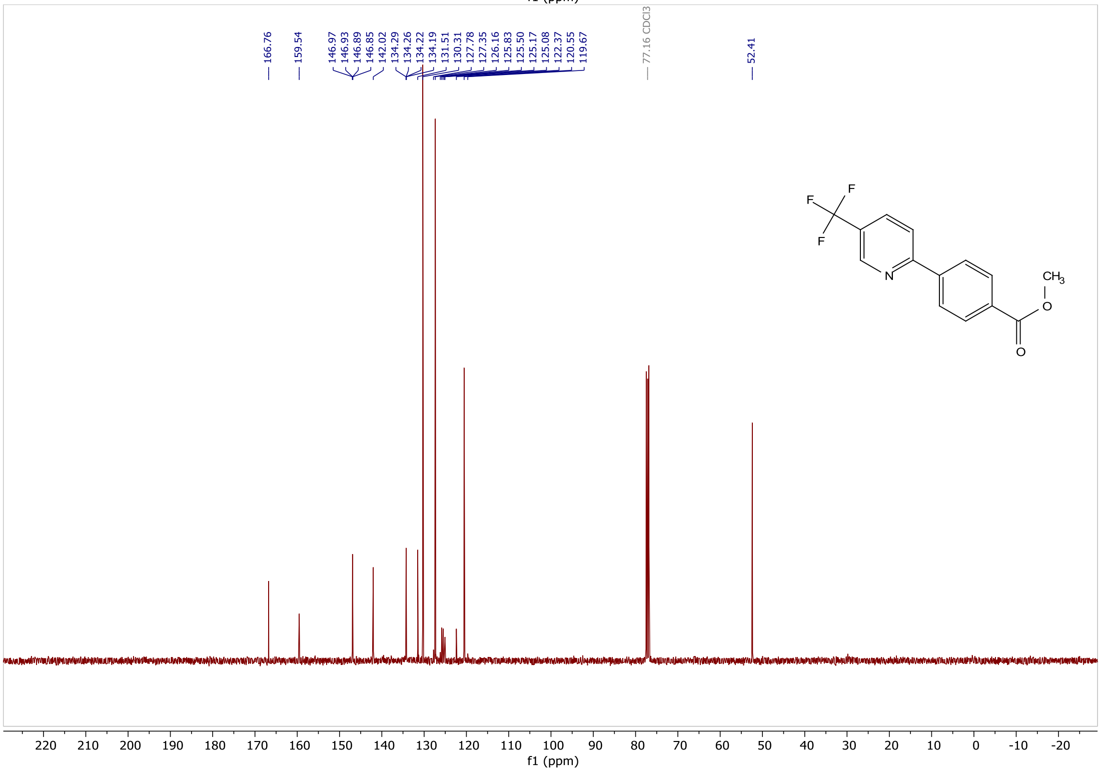

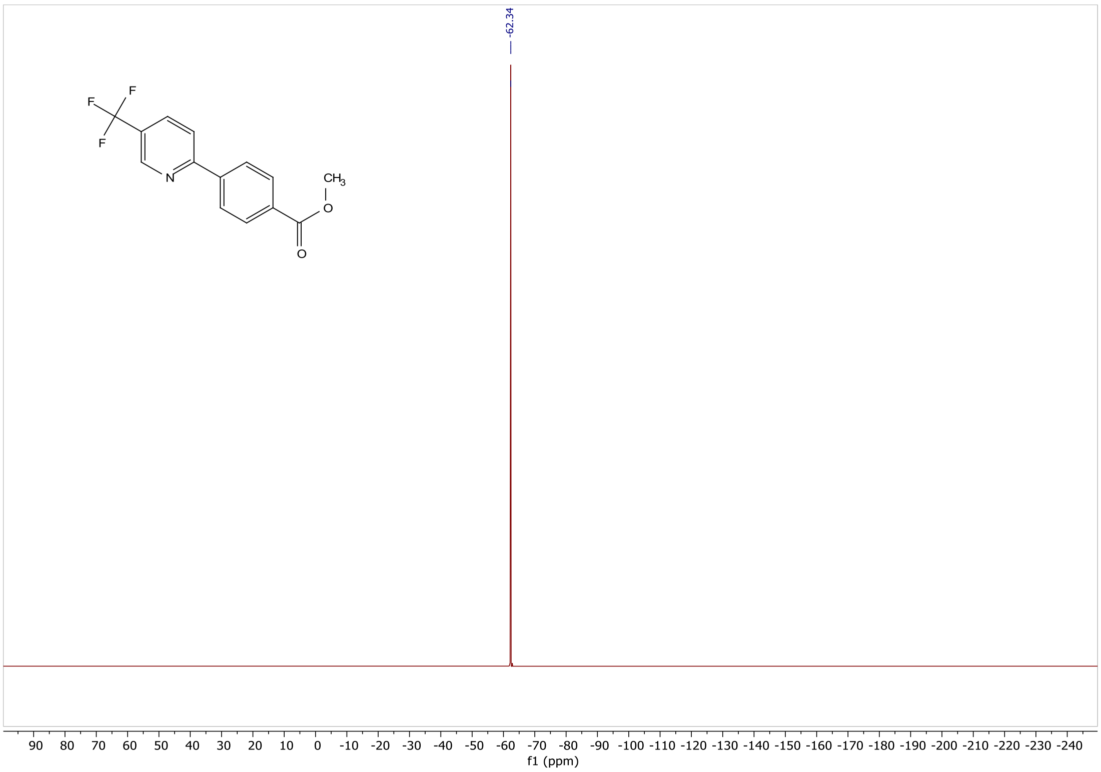

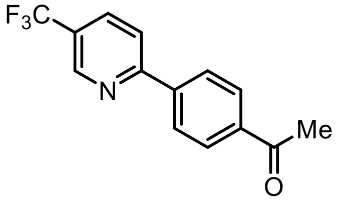

1-(4-(5-(trifluoromethyl)pyridin-2-yl)phenyl)ethan-1-one (3j)

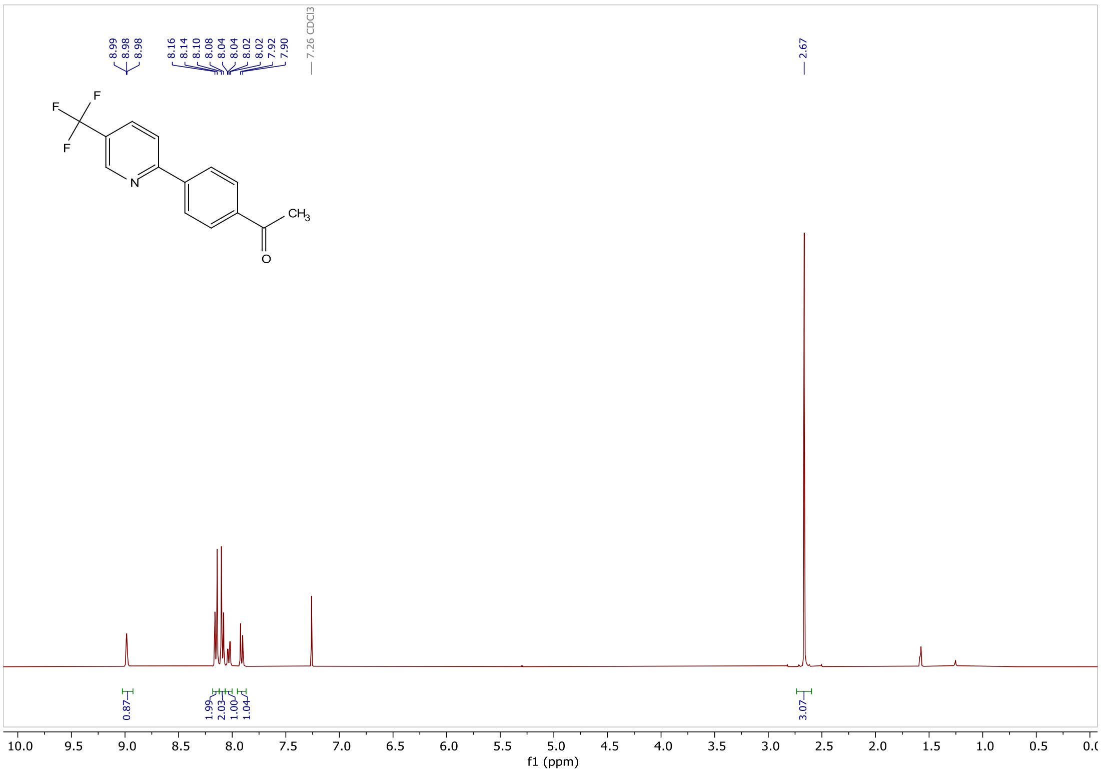

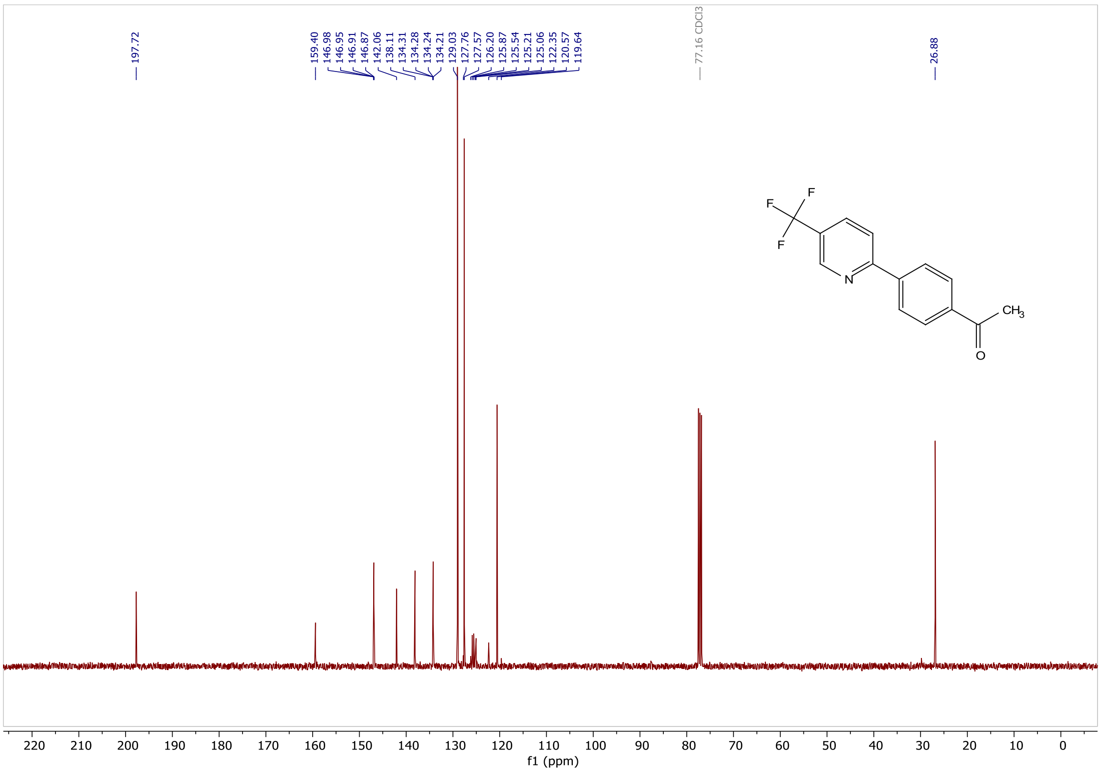
197.72
159.40
146.98
146.95
146.91
146.87
142.06
138.11
134.31
134.28
134.24
134.21
129.03
127.76
127.57
126.20
125.87
125.54
125.21
125.06
122.35
120.57
119.64
77.16 CDCl3
26.88
F
F
F
N
CH3
O
f1 (ppm)

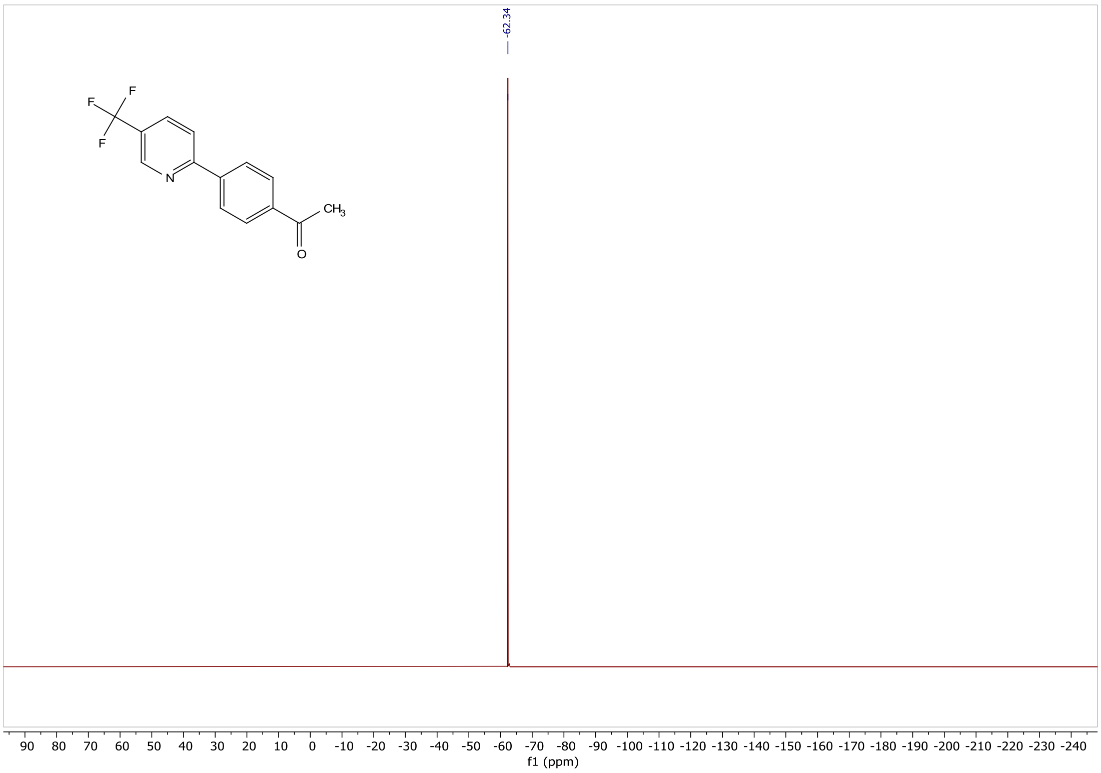
-62.34
F
F
F
N
CH3
O
f1 (ppm)

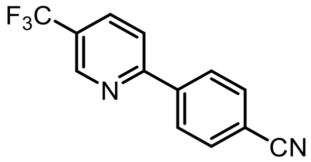

4-(5-(trifluoromethyl)pyridin-2-yl)benzonitrile (3k)

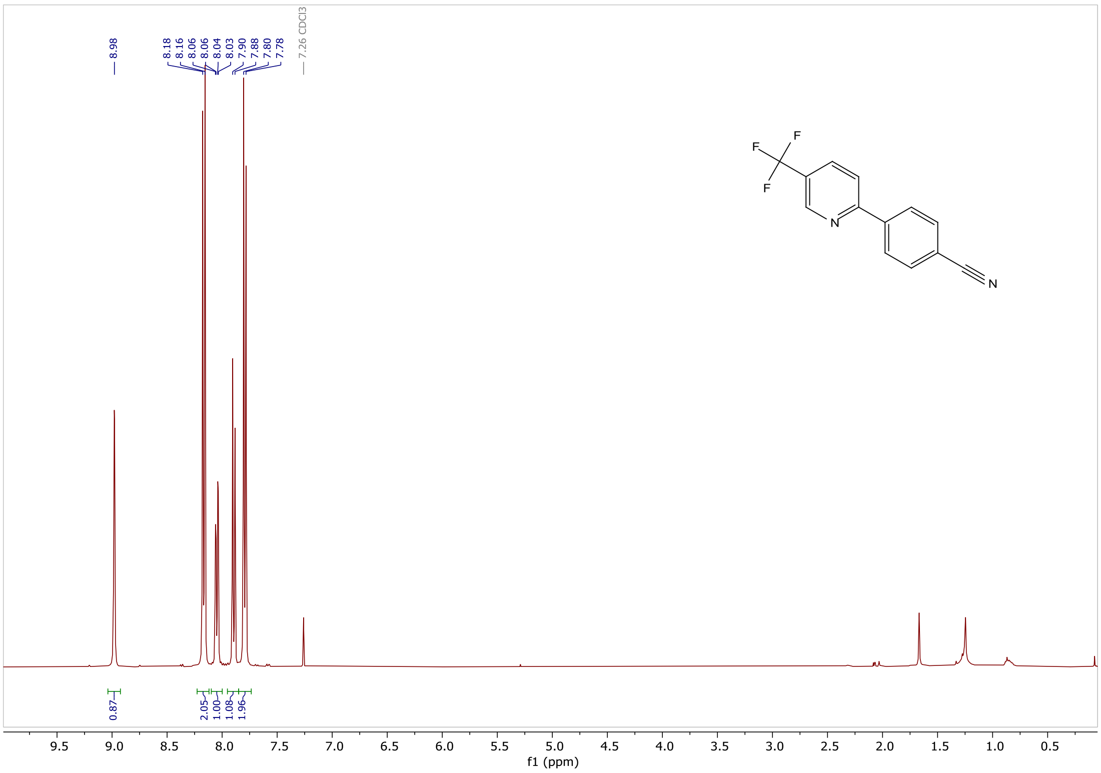

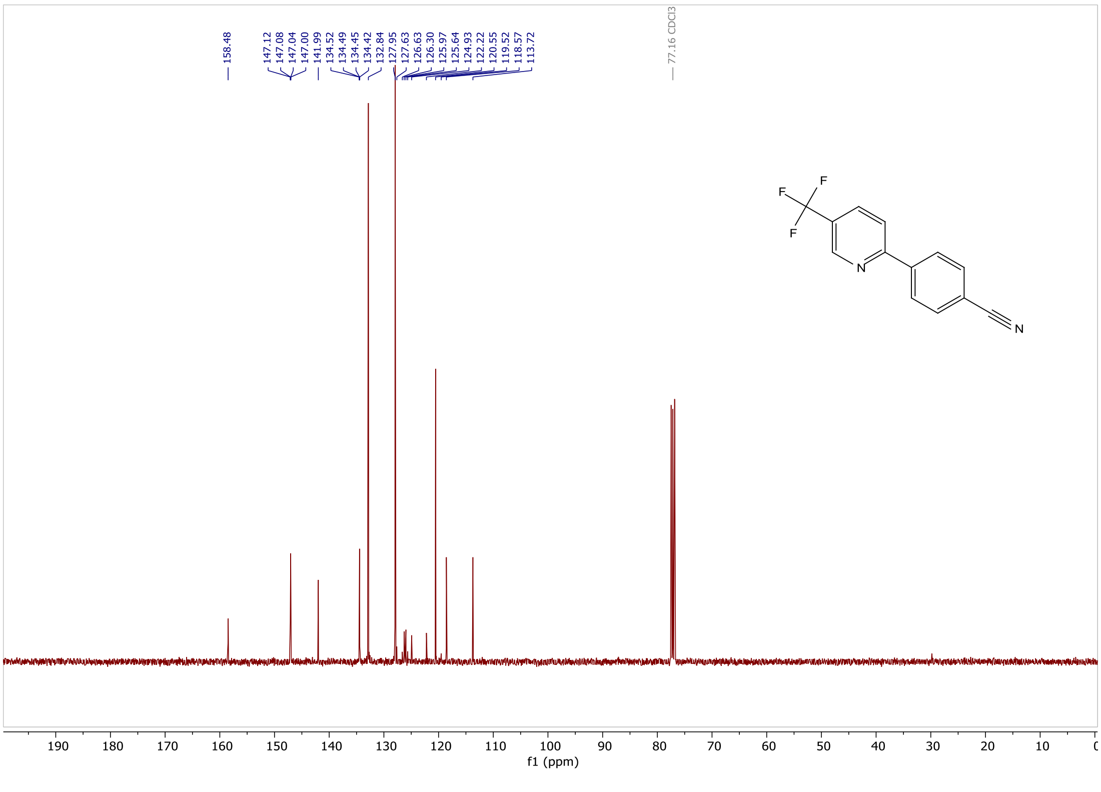

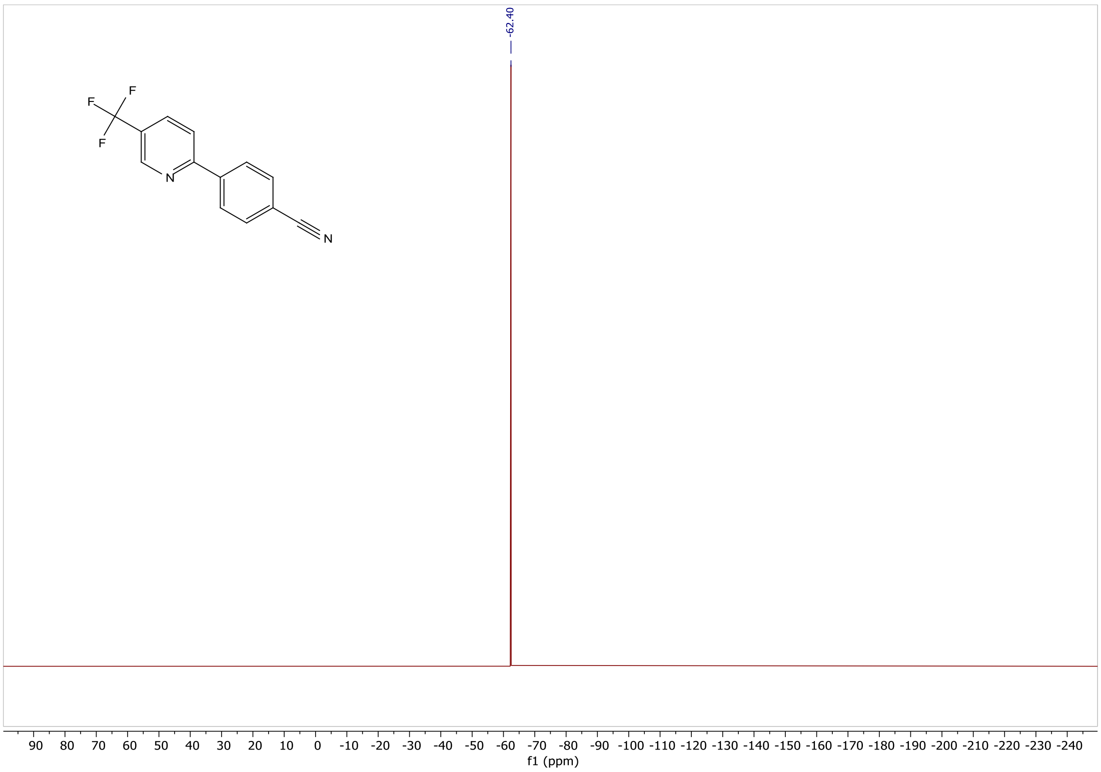

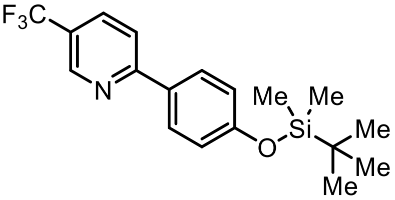

2-(4-((tert-butyldimethylsilyl)oxy)phenyl)-5-(trifluoromethyl)pyridine (3l)

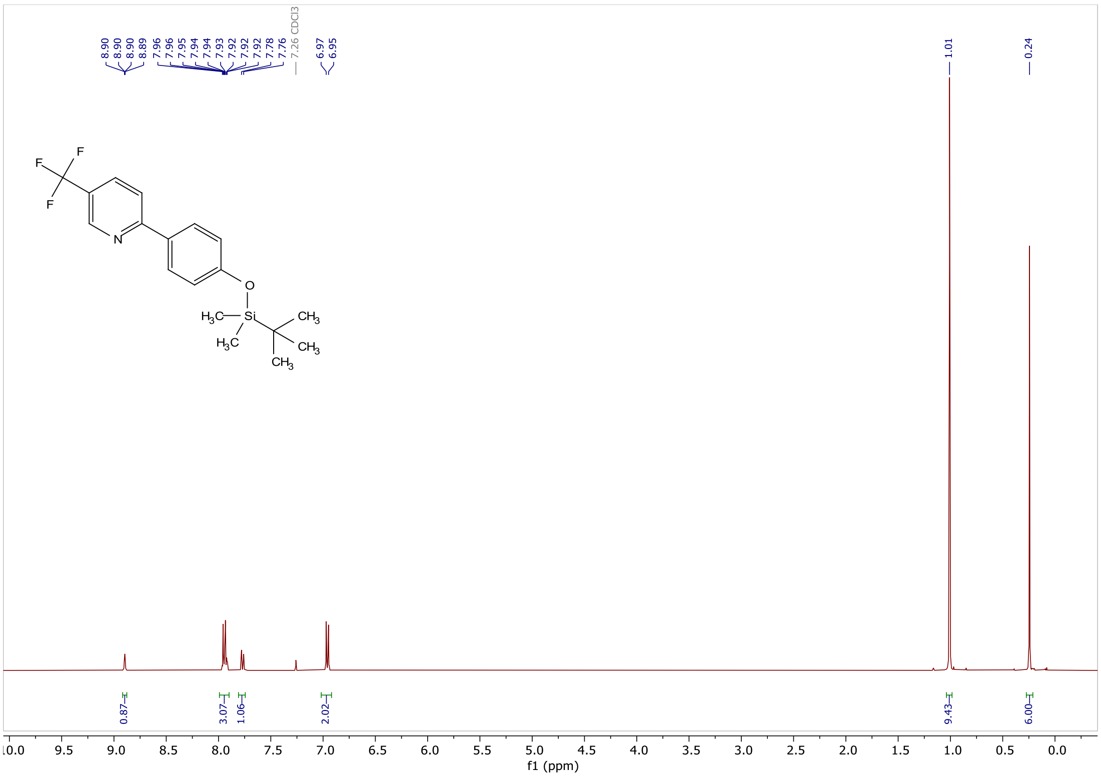

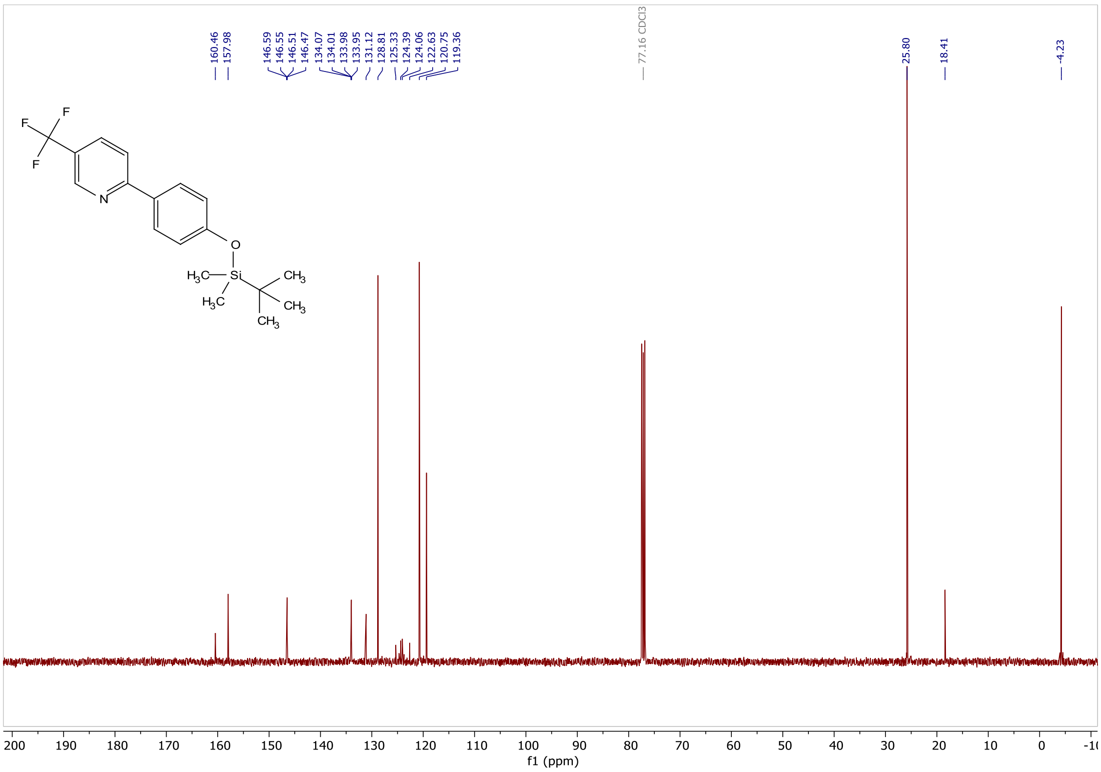

160.46
157.98
146.59
146.55
146.51
146.47
134.07
134.01
133.98
133.95
131.12
128.81
125.33
124.39
124.06
122.63
120.75
119.36
77.16 CDCl3
25.80
18.41
-4.23
200 190 180 170 160 150 140 130 120 110 100 90 80 70 60 50 40 30 20 10 0
f1 (ppm)

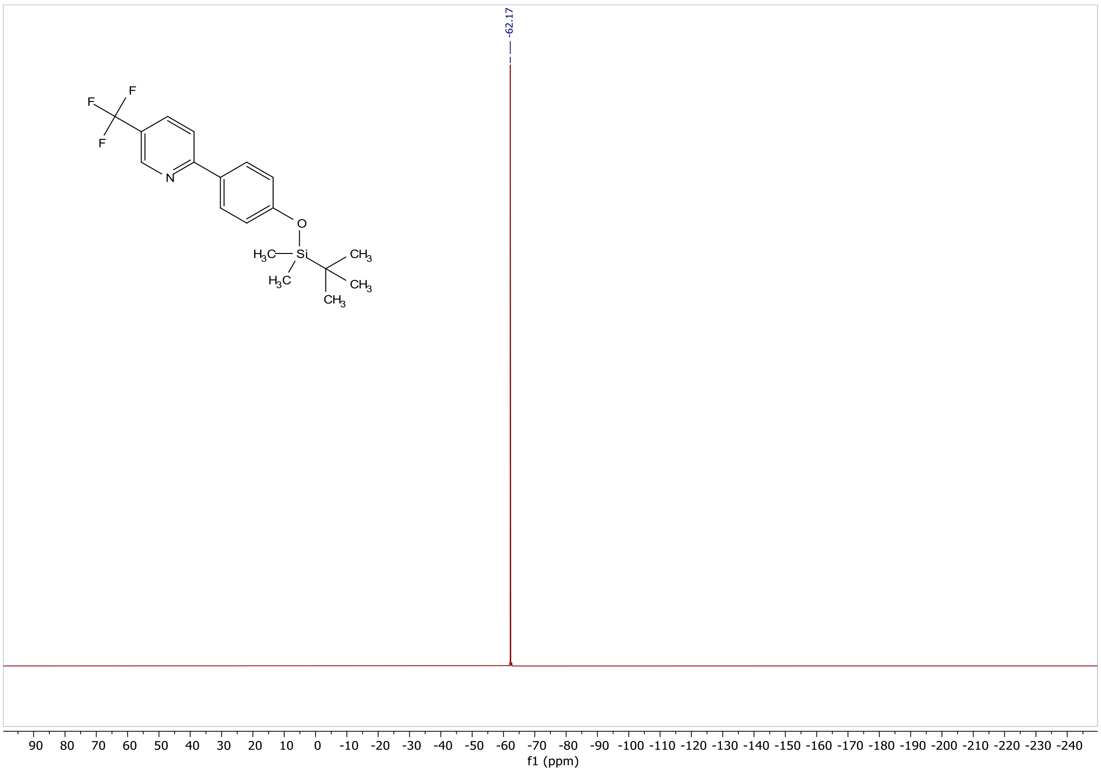

-62.17
90 80 70 60 50 40 30 20 10 0 -10 -20 -30 -40 -50 -60 -70 -80 -90 -100 -110 -120 -130 -140 -150 -160 -170 -180 -190 -200 -210 -220 -230 -240
f1 (ppm)

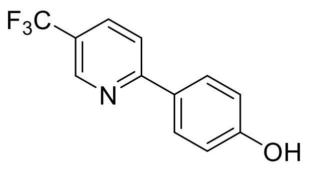

4-(5-(trifluoromethyl)pyridin-2-yl)phenol (3l-OH)

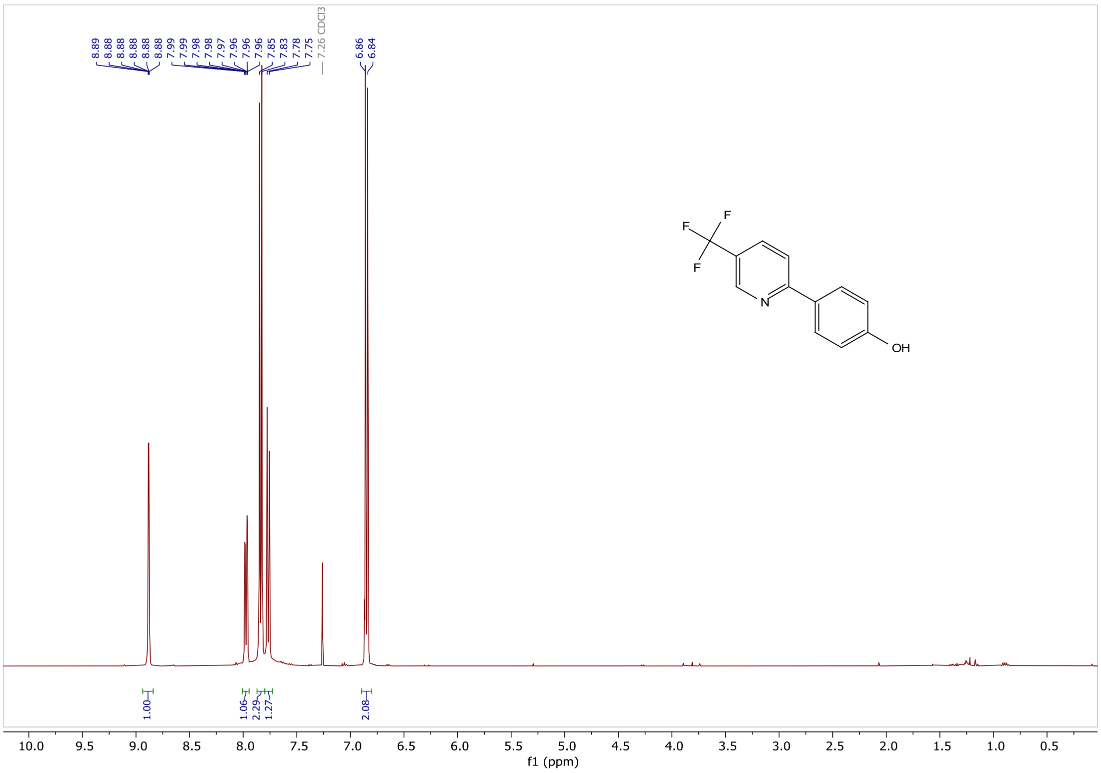

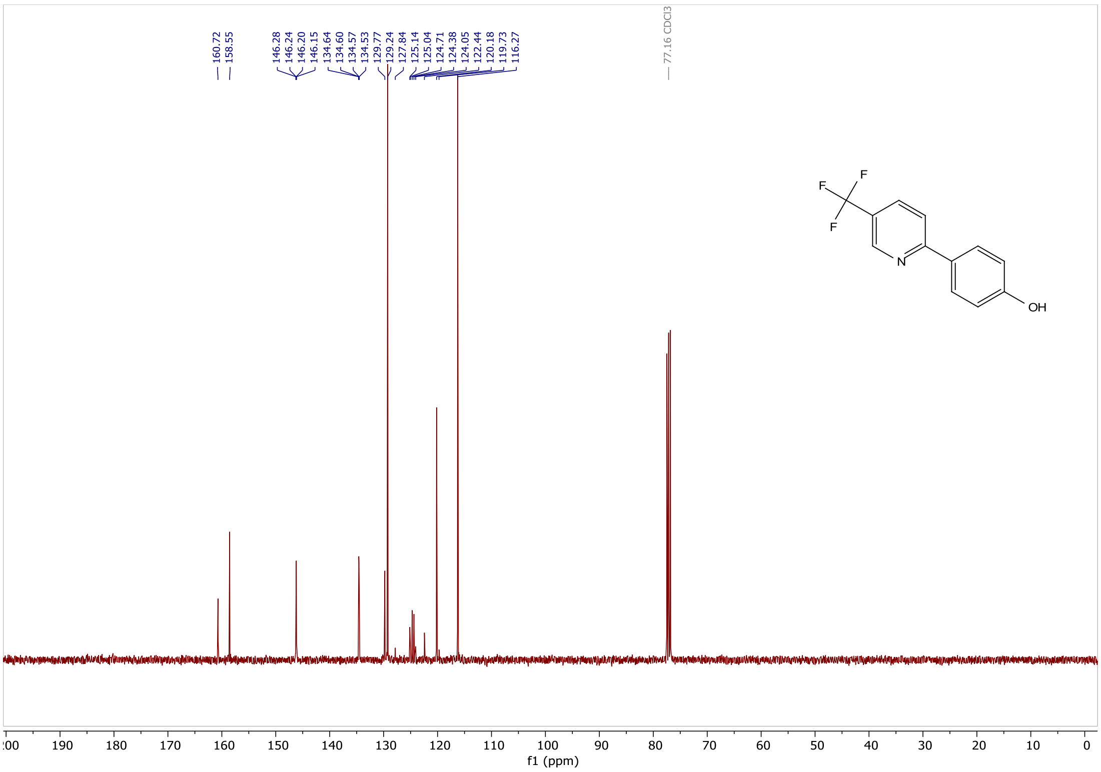

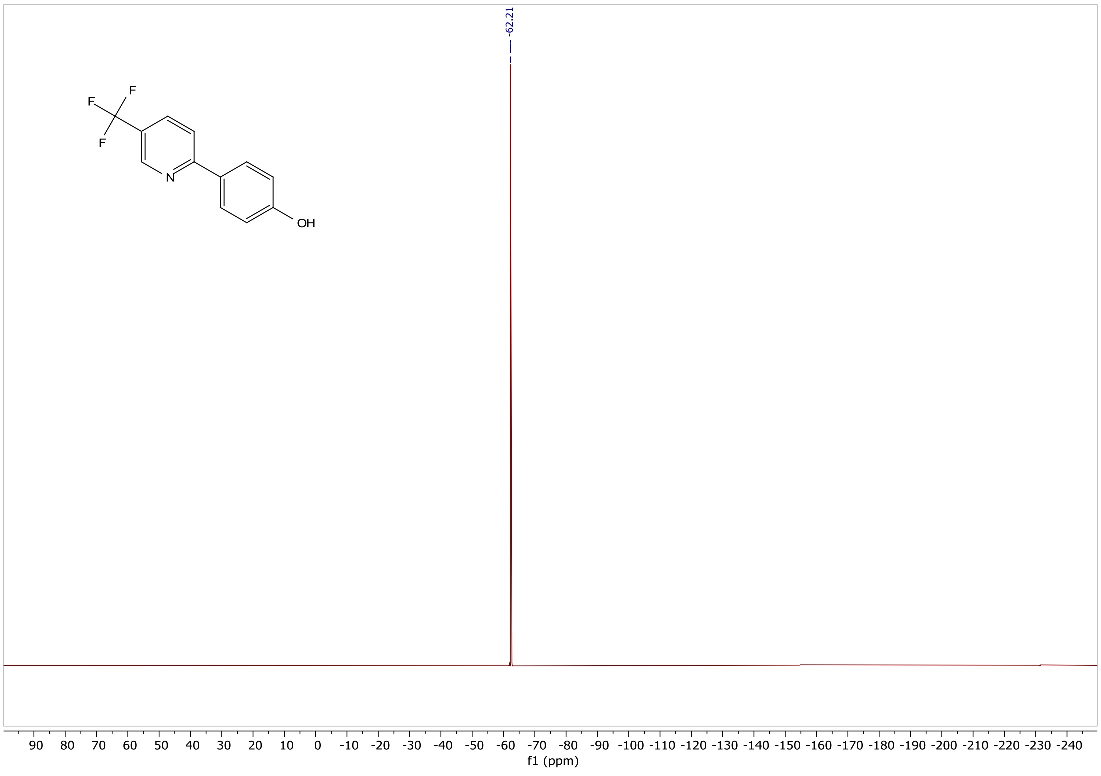

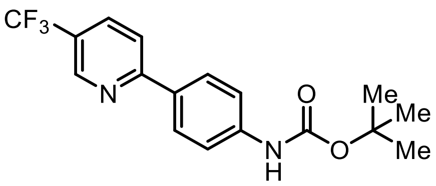

tert-butyl (4-(5-(trifluoromethyl)pyridin-2-yl)phenyl)carbamate (3m)

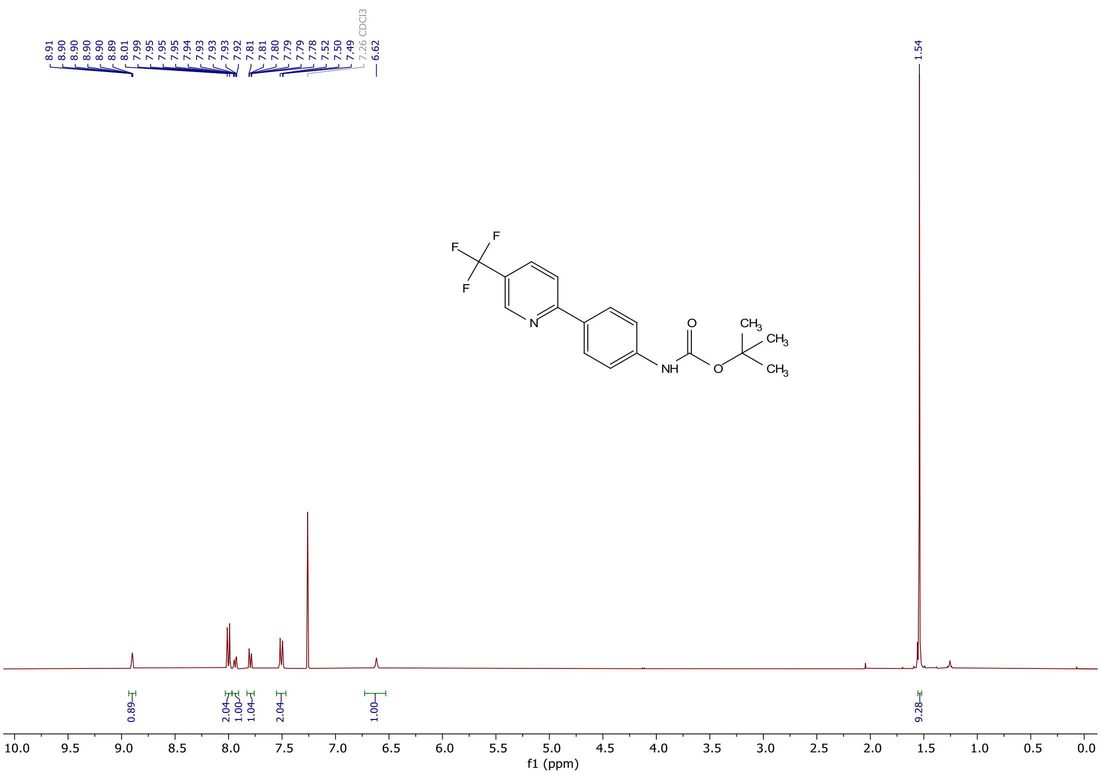

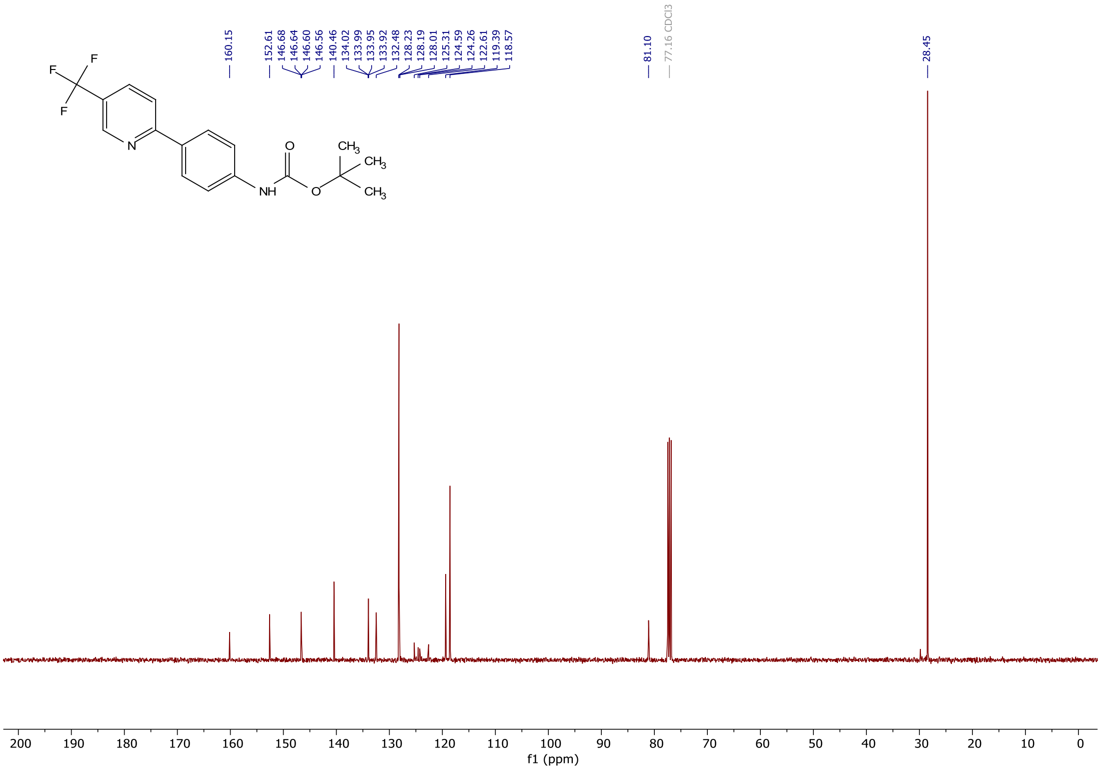
160.15
152.61
146.68
146.64
146.60
146.56
140.46
134.02
133.99
133.95
133.92
132.48
128.23
128.19
128.01
125.31
124.59
124.26
122.61
119.39
118.57
81.10
77.16 CDCl3
28.45
f1 (ppm)

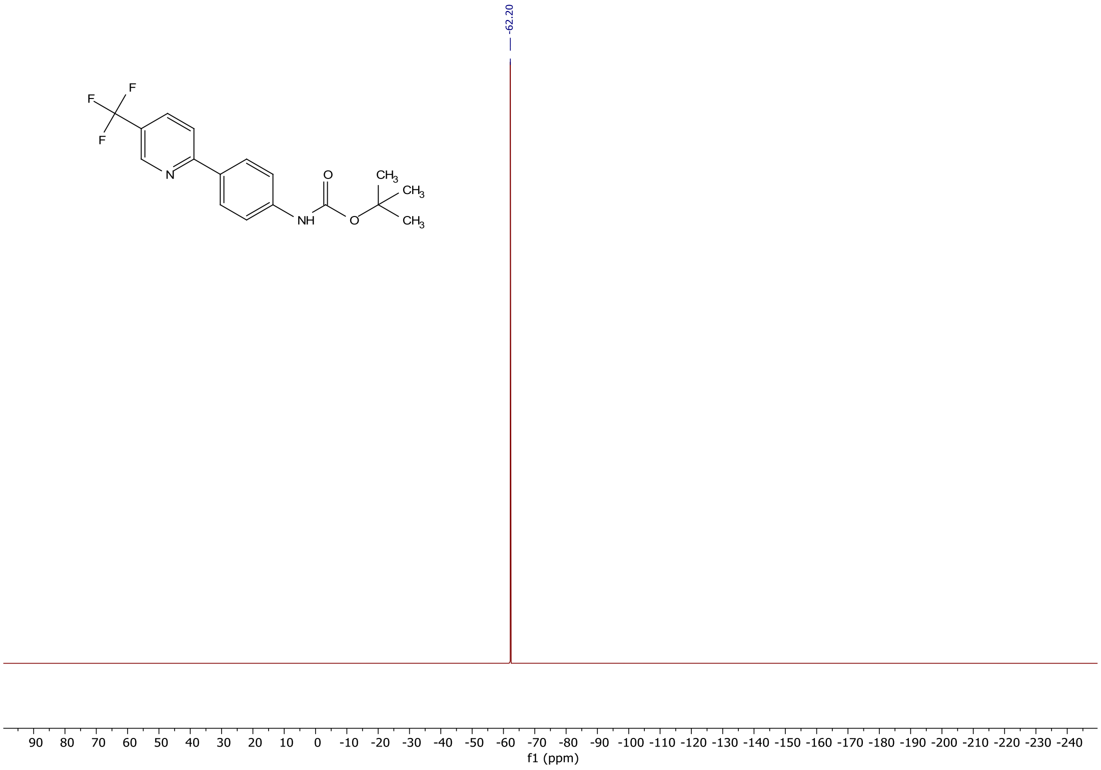
-62.20
f1 (ppm)

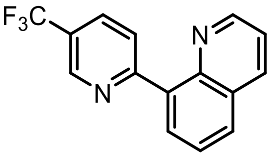

8-(5-(trifluoromethyl)pyridin-2-yl)quinolone (3n)

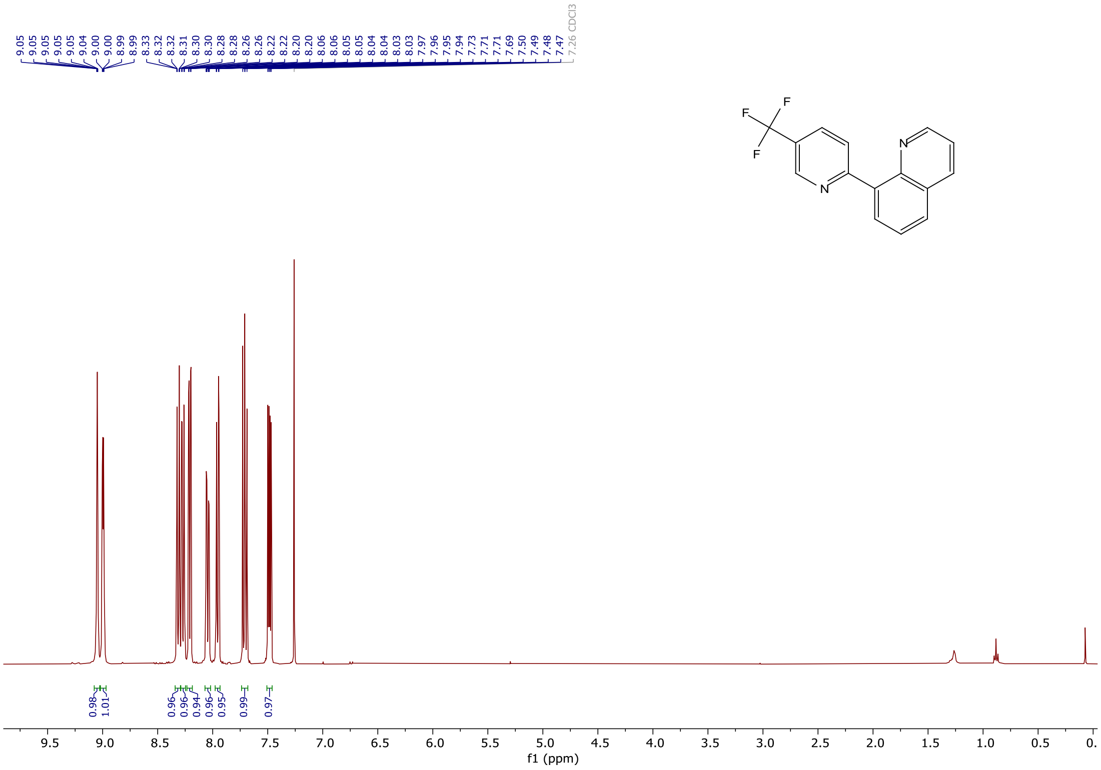

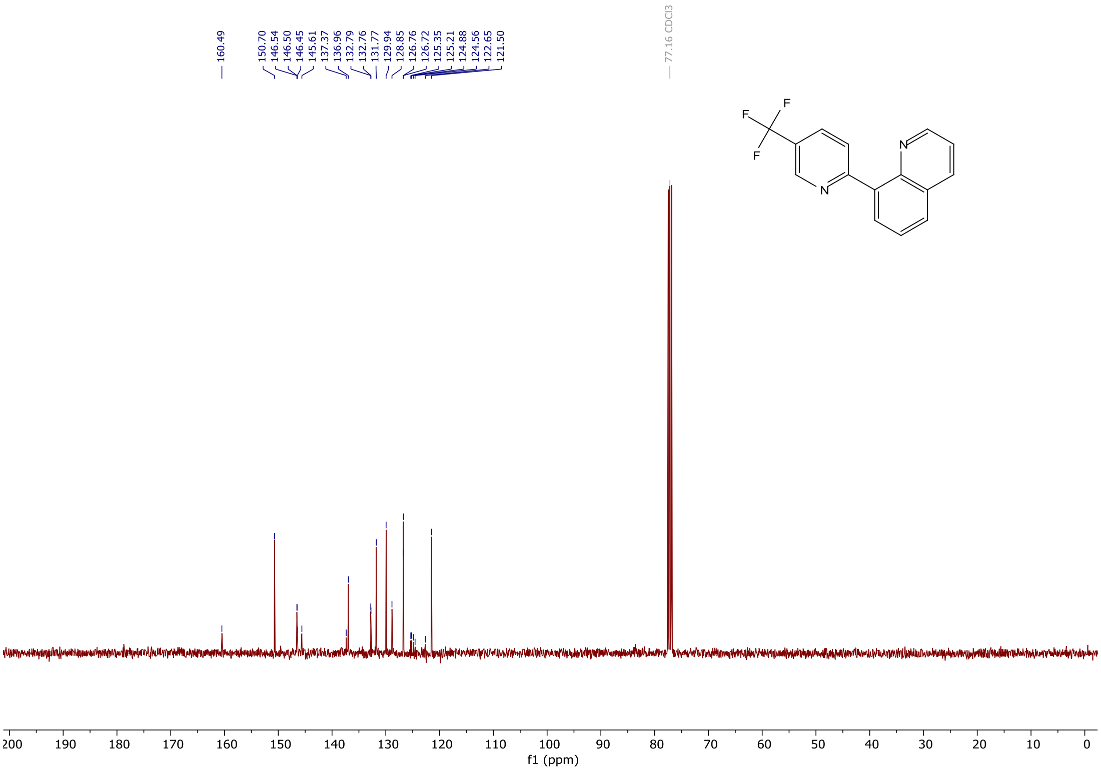

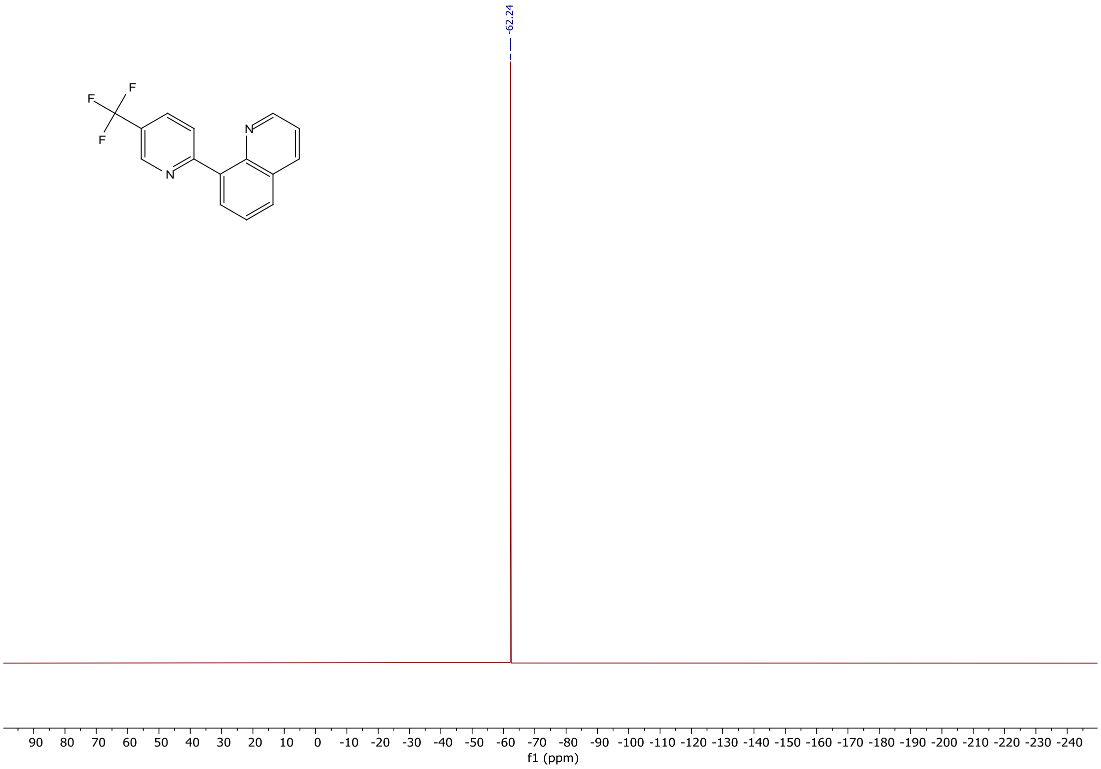
-62.24
90
80
70
60
50
40
30
20
10
0
-10
-20
-30
-40
-50
-60
-70
-80
-90
-100
-110
-120
-130
-140
-150
-160
-170
-180
-190
-200
-210
-220
-230
-240
f1 (ppm)

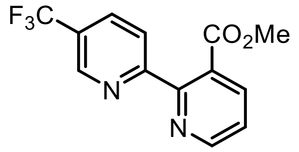

methyl 5'-(trifluoromethyl)-[2,2'-bipyridine]-3-carboxylate (3o)

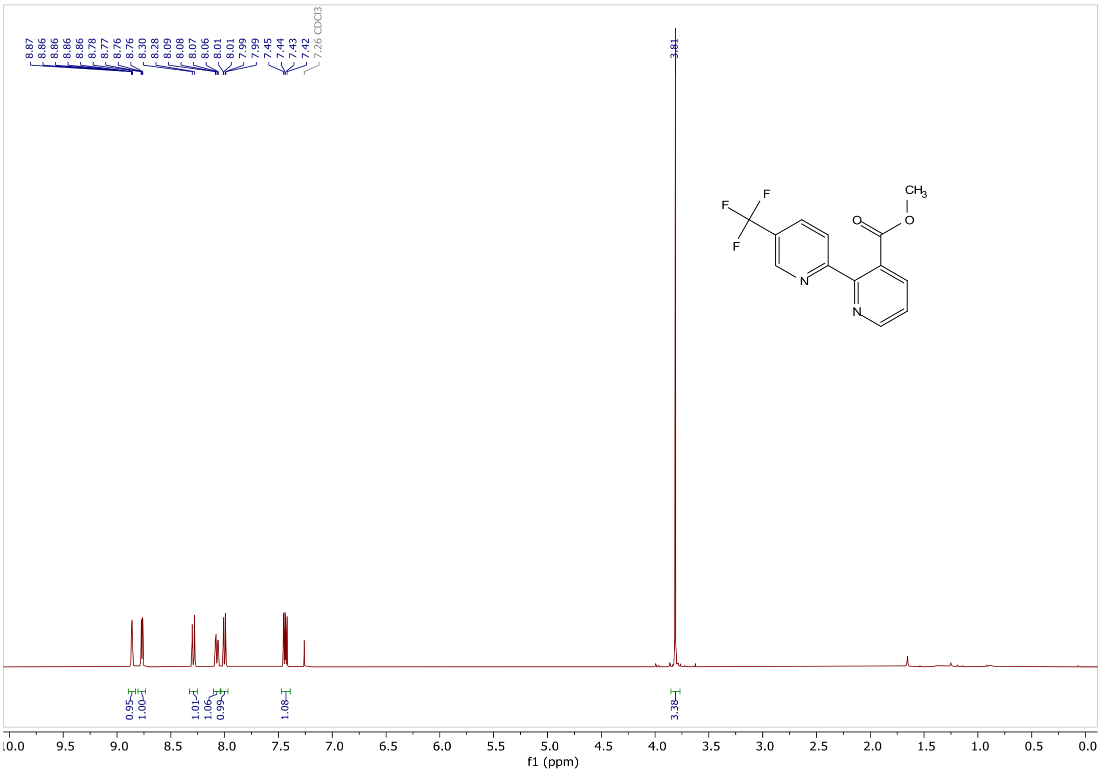

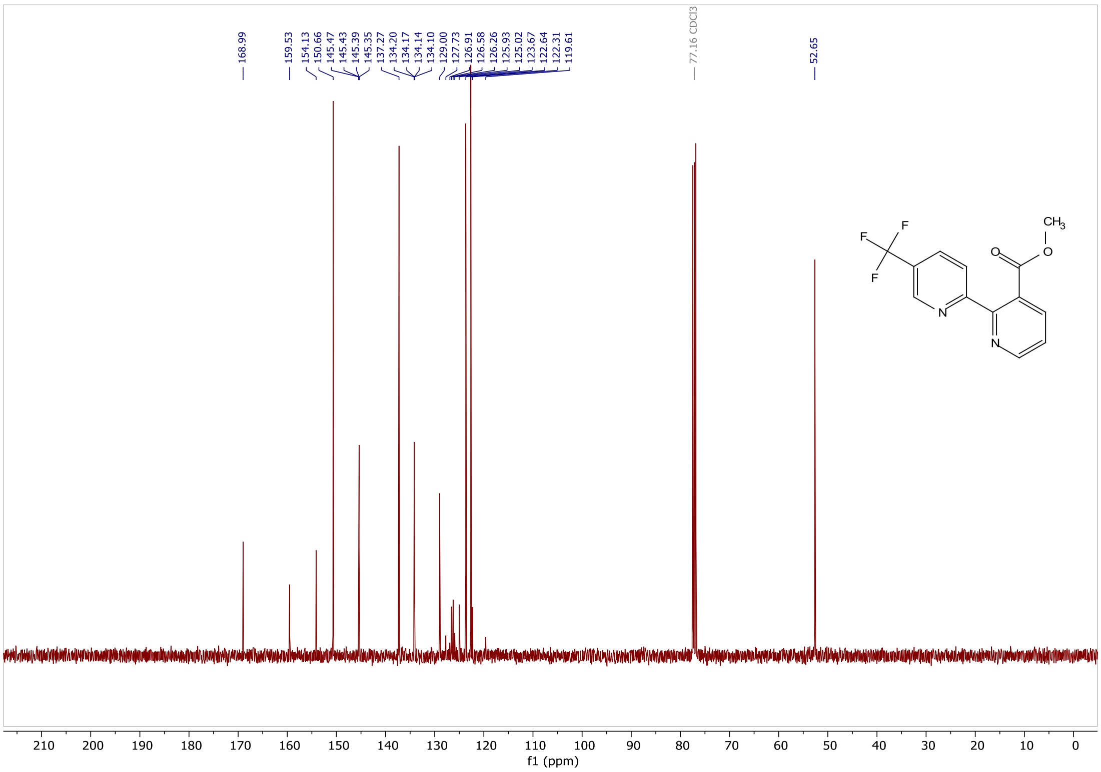

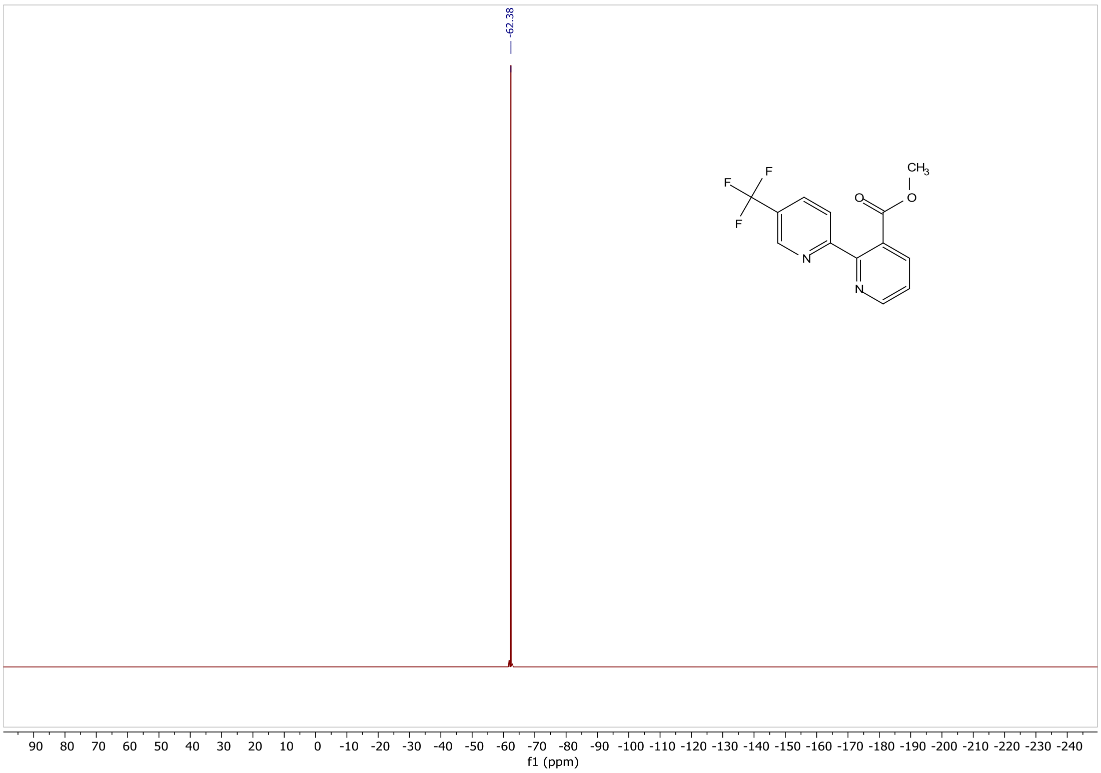

-62.38
90 80 70 60 50 40 30 20 10 0 -10 -20 -30 -40 -50 -60 -70 -80 -90 -100 -110 -120 -130 -140 -150 -160 -170 -180 -190 -200 -210 -220 -230 -240
f1 (ppm)

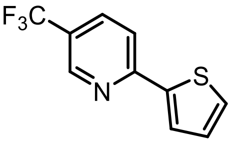

2-(thiophen-2-yl)-5-(trifluoromethyl)pyridine (3p)

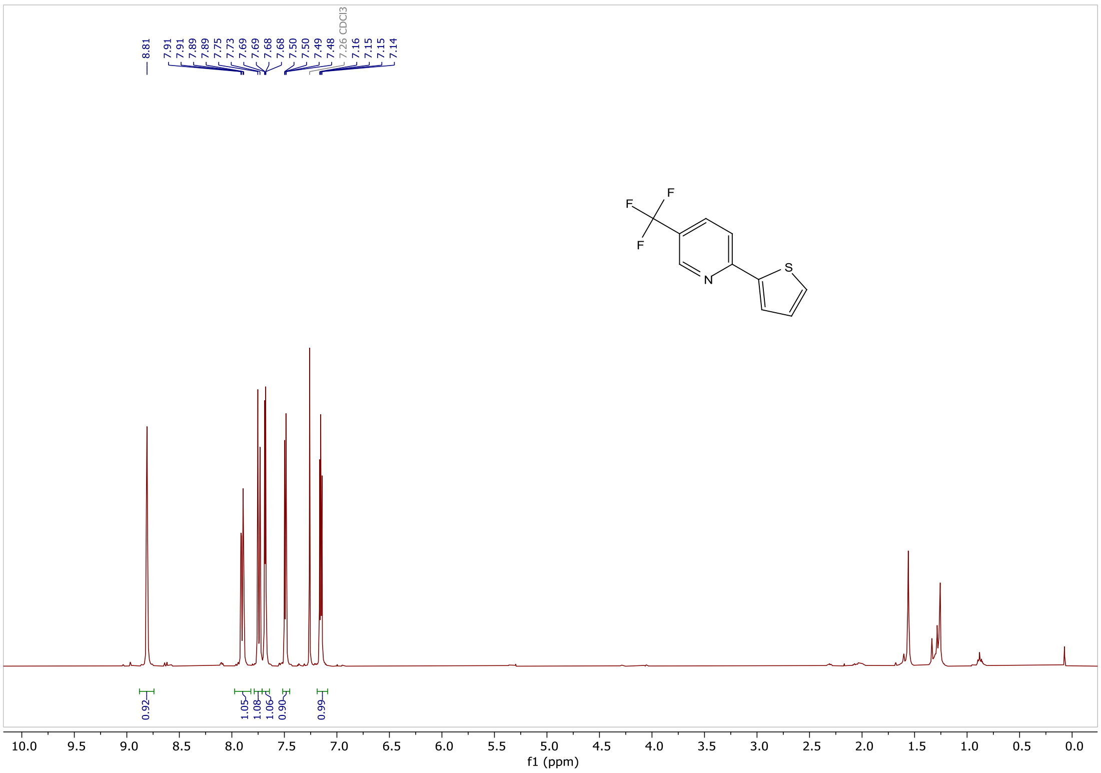

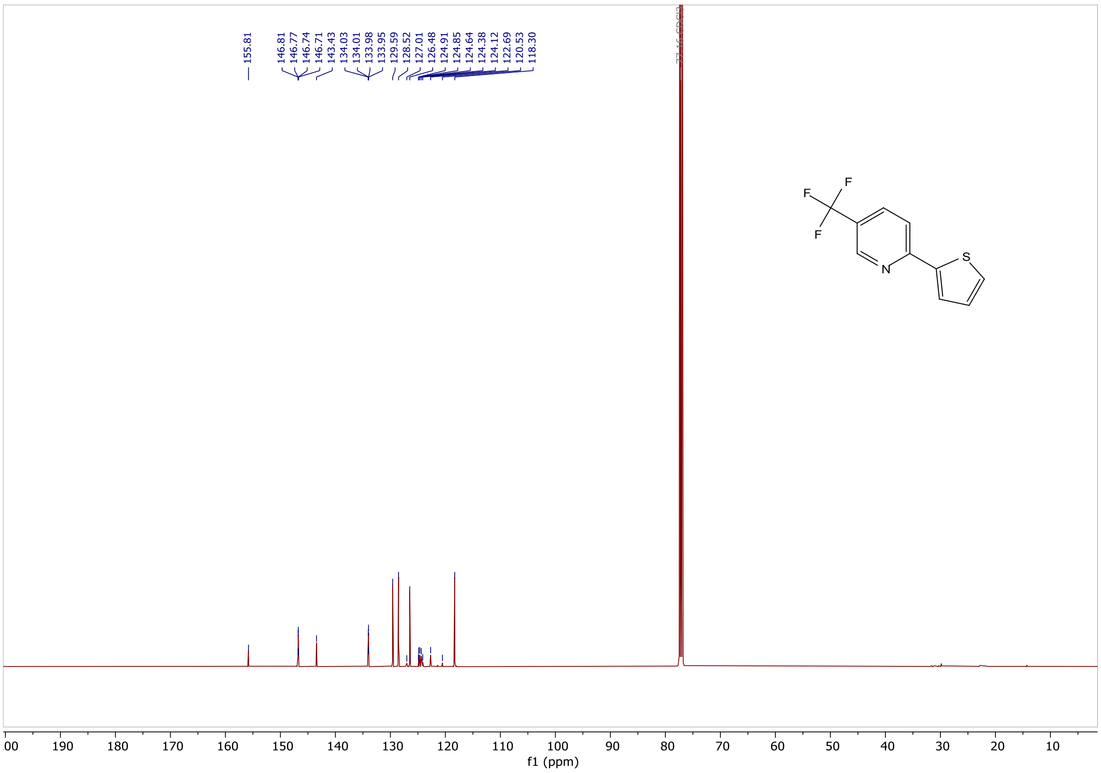

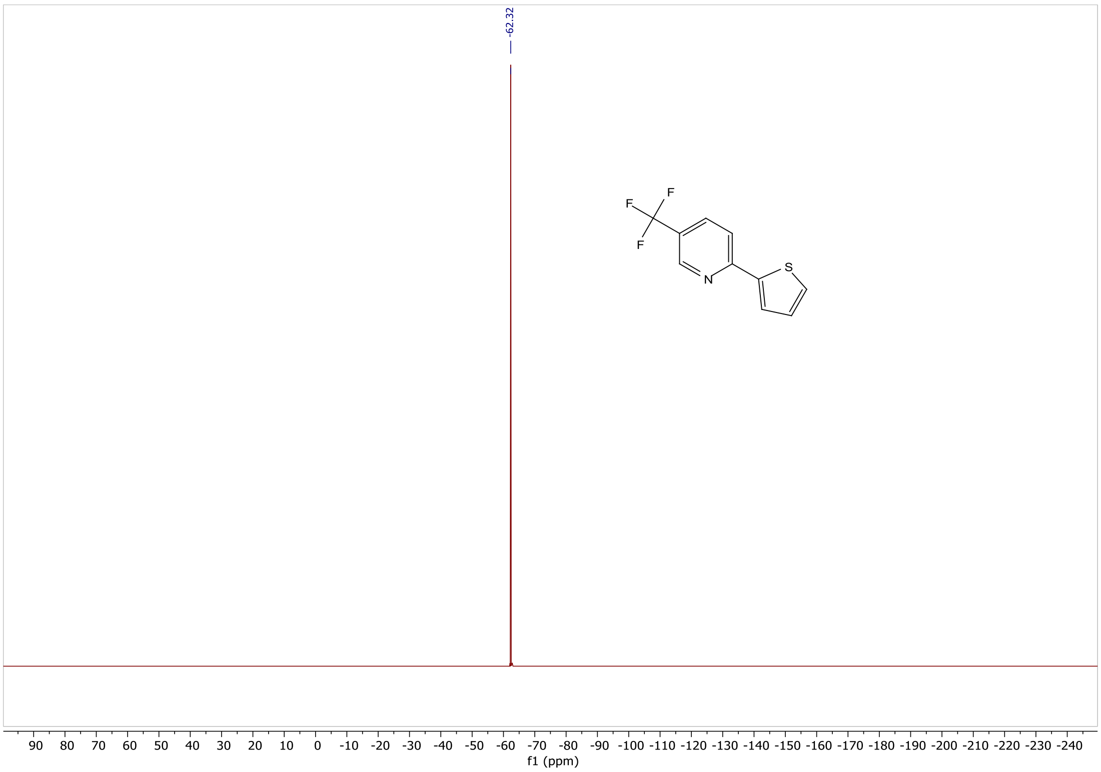

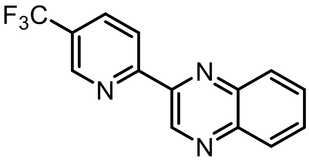

2-(5-(trifluoromethyl)pyridin-2-yl)quinoxaline (3q)

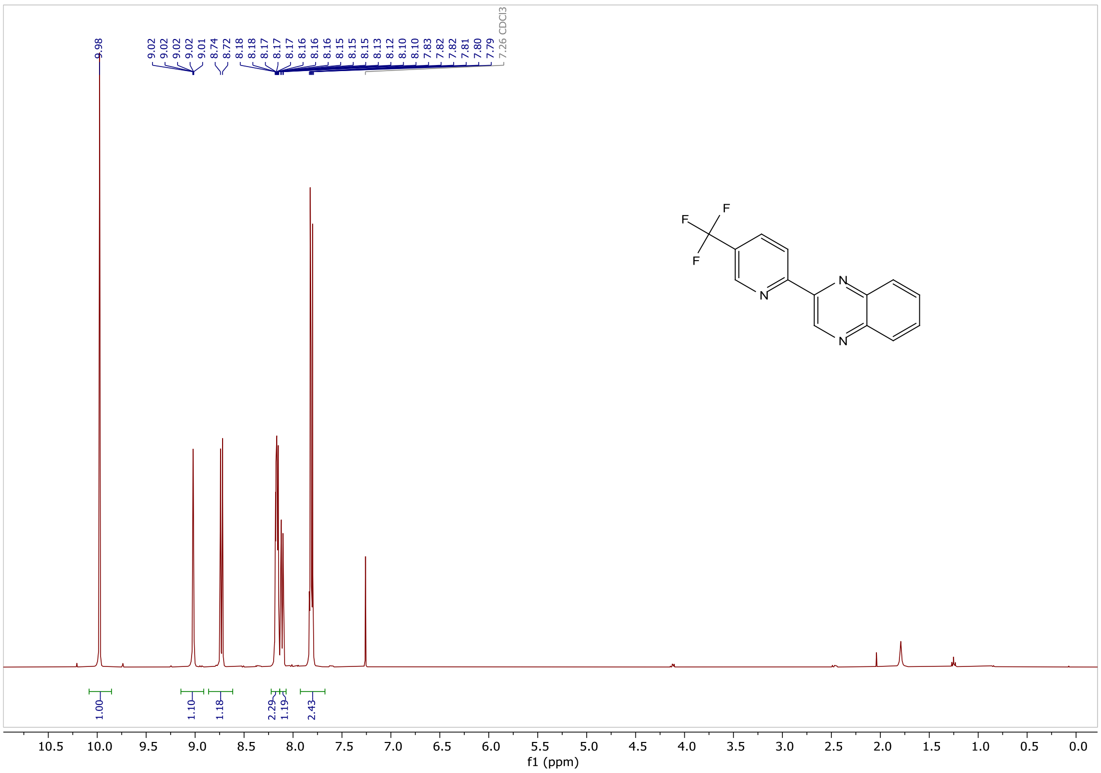

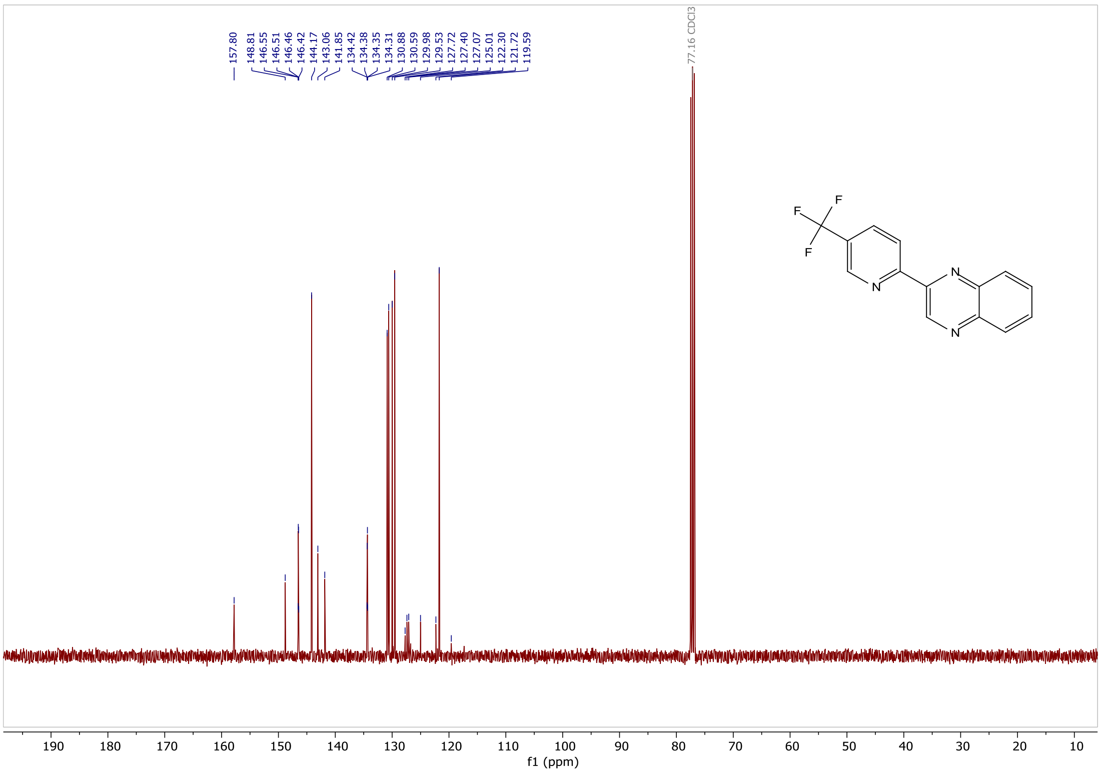

157.80
148.81
146.55
146.51
146.46
146.42
144.17
143.06
141.85
134.42
134.38
134.35
134.31
130.88
130.59
129.98
129.53
127.72
127.40
127.07
125.01
122.30
121.72
119.59
77.16 CDCl3
F
F
F
N
N
N
190
180
170
160
150
140
130
120
110
100
90
80
70
60
50
40
30
20
10
f1 (ppm)

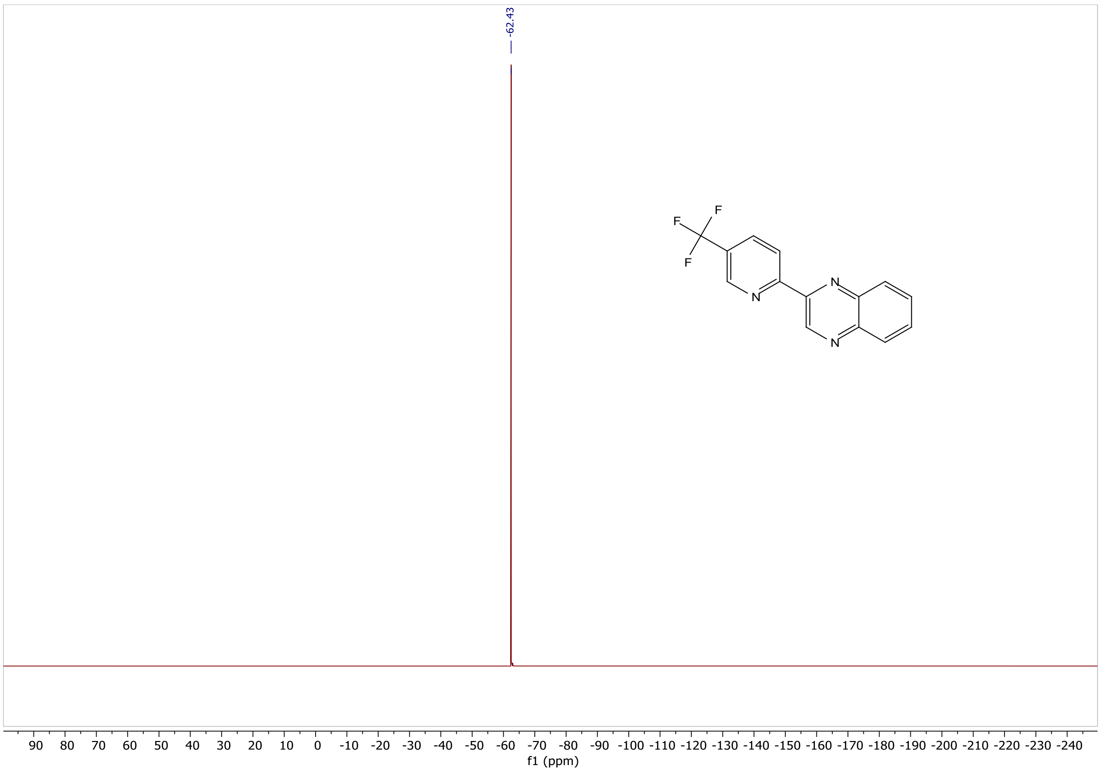

-62.43
F
F
F
N
N
N
90 80 70 60 50 40 30 20 10 0 -10 -20 -30 -40 -50 -60 -70 -80 -90 -100 -110 -120 -130 -140 -150 -160 -170 -180 -190 -200 -210 -220 -230 -240
f1 (ppm)

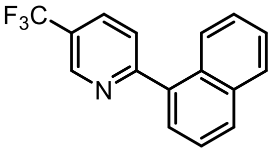

2-(naphthalen-1-yl)-5-(trifluoromethyl)pyridine (3r)

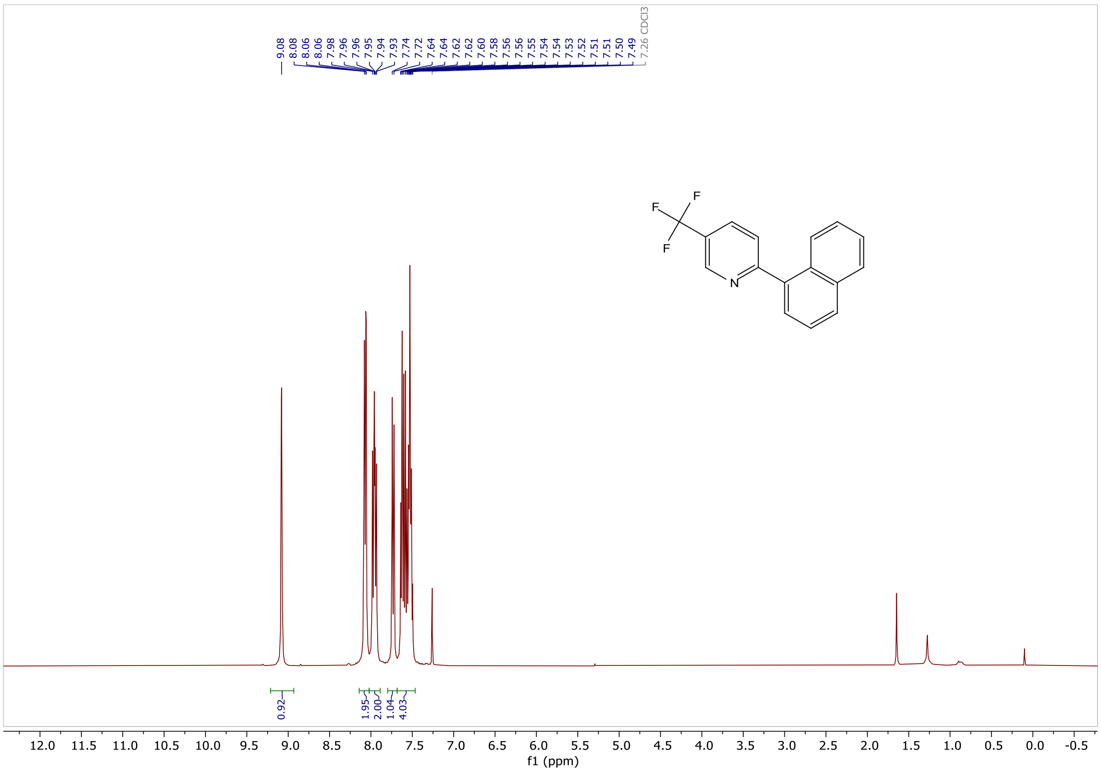

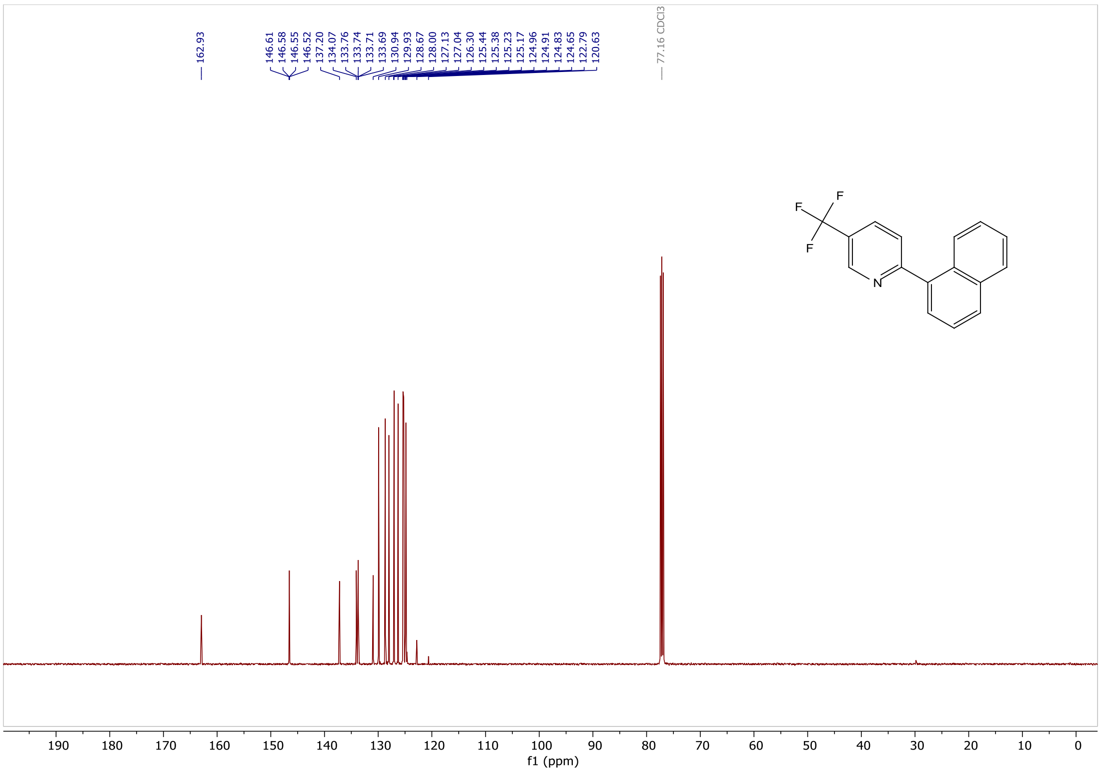

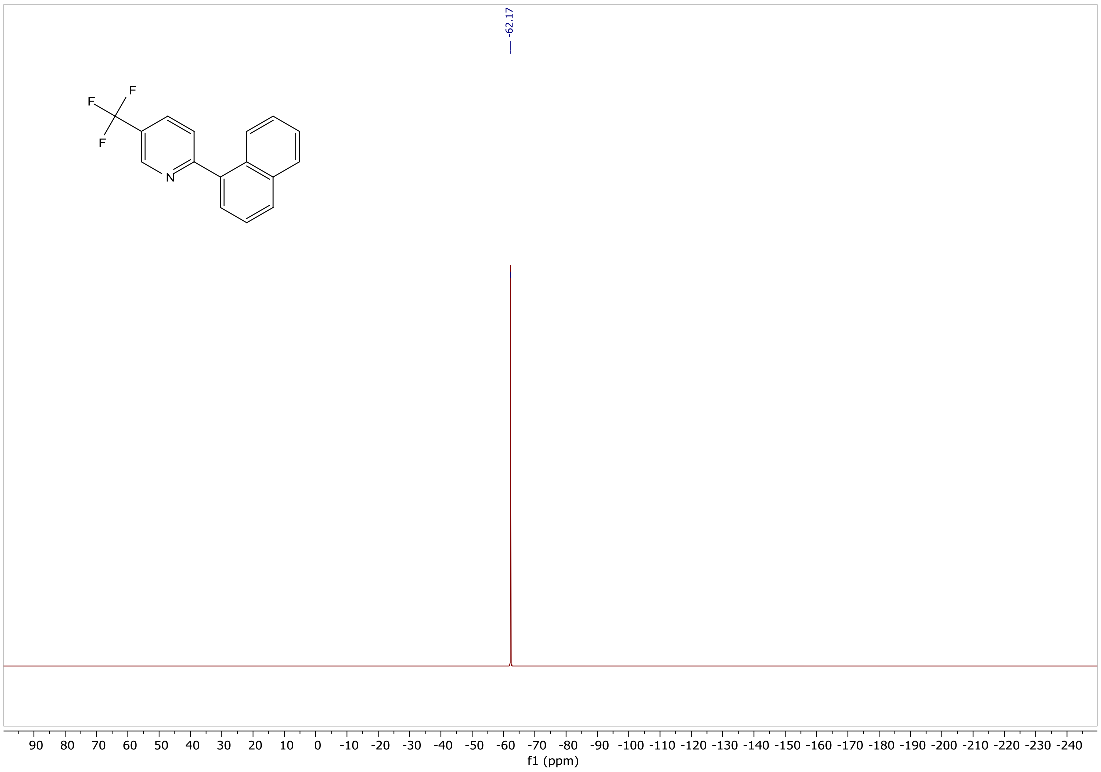

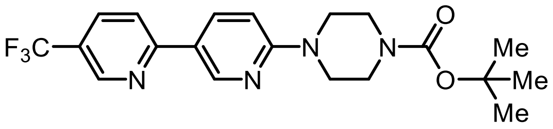

***tert*-butyl 4-(5-(trifluoromethyl)-[2,3'-bipyridin]-6'-yl)piperazine-1-carboxylate (3s)**

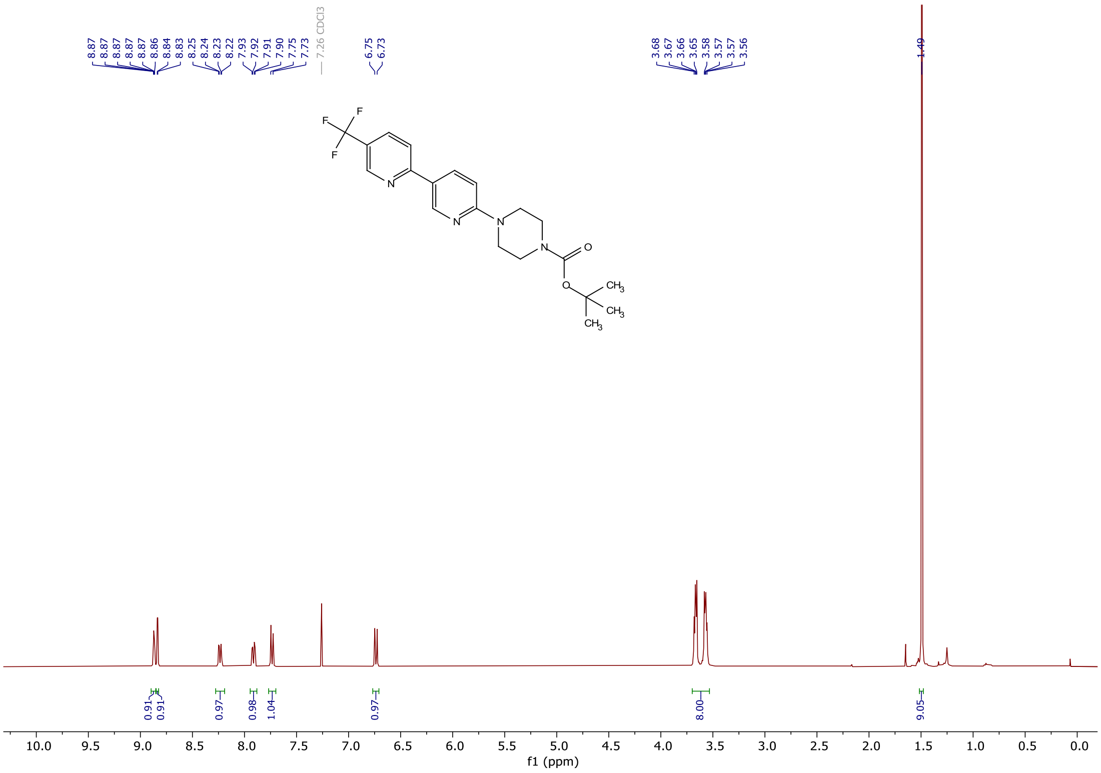

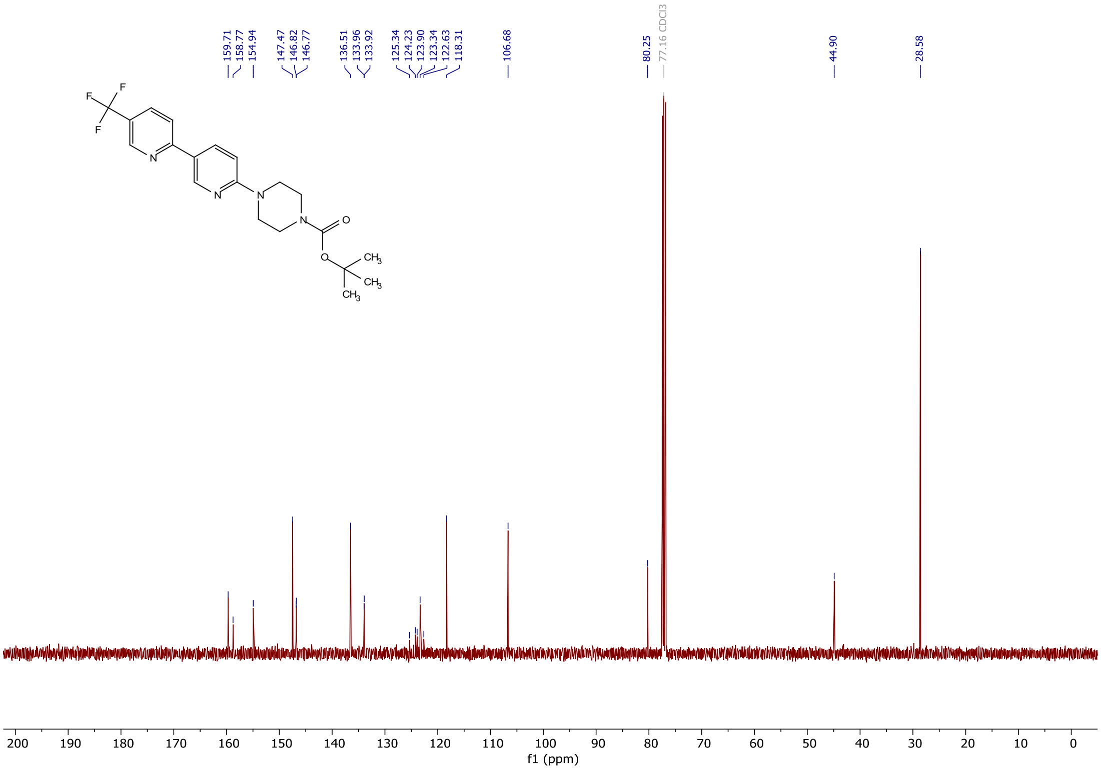
159.71
158.77
154.94
147.47
146.82
146.77
136.51
133.96
133.92
125.34
124.23
123.90
123.34
122.63
118.31
106.68
80.25
77.16 CDCl3
44.90
28.58
f1 (ppm)

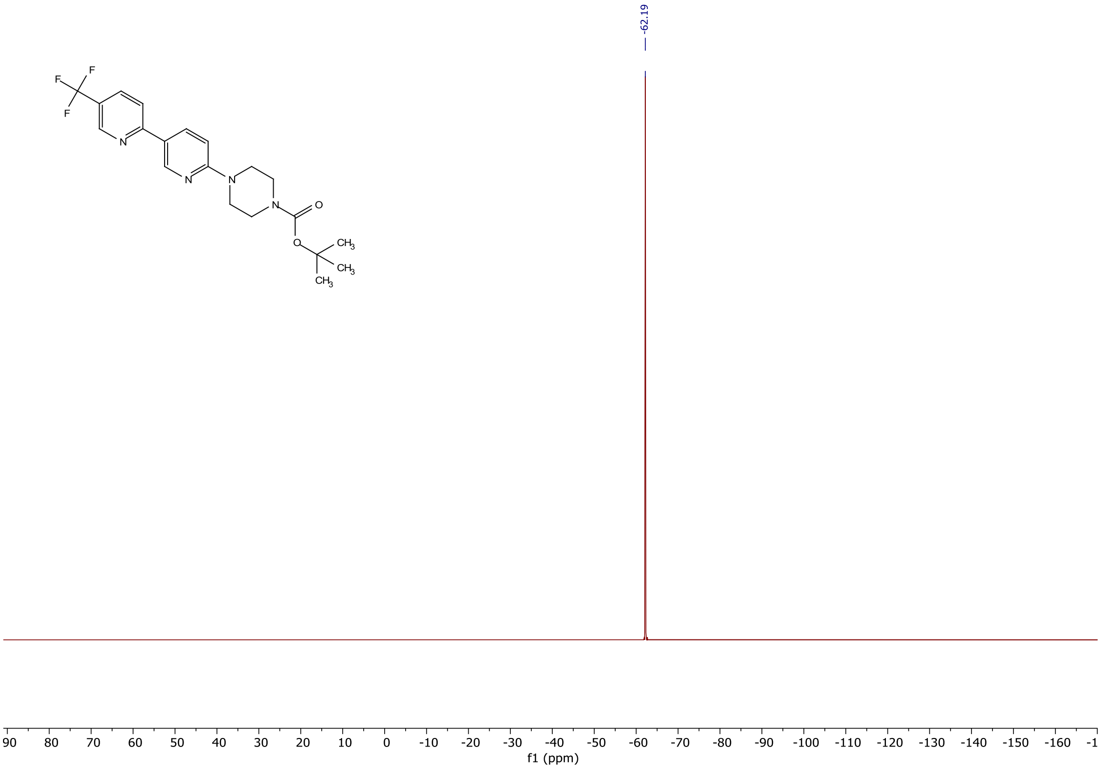
-62.19
f1 (ppm)

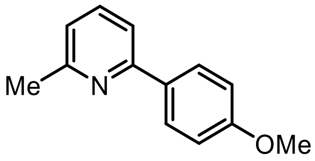

2-(4-methoxyphenyl)-6-methylpyridine (3t)

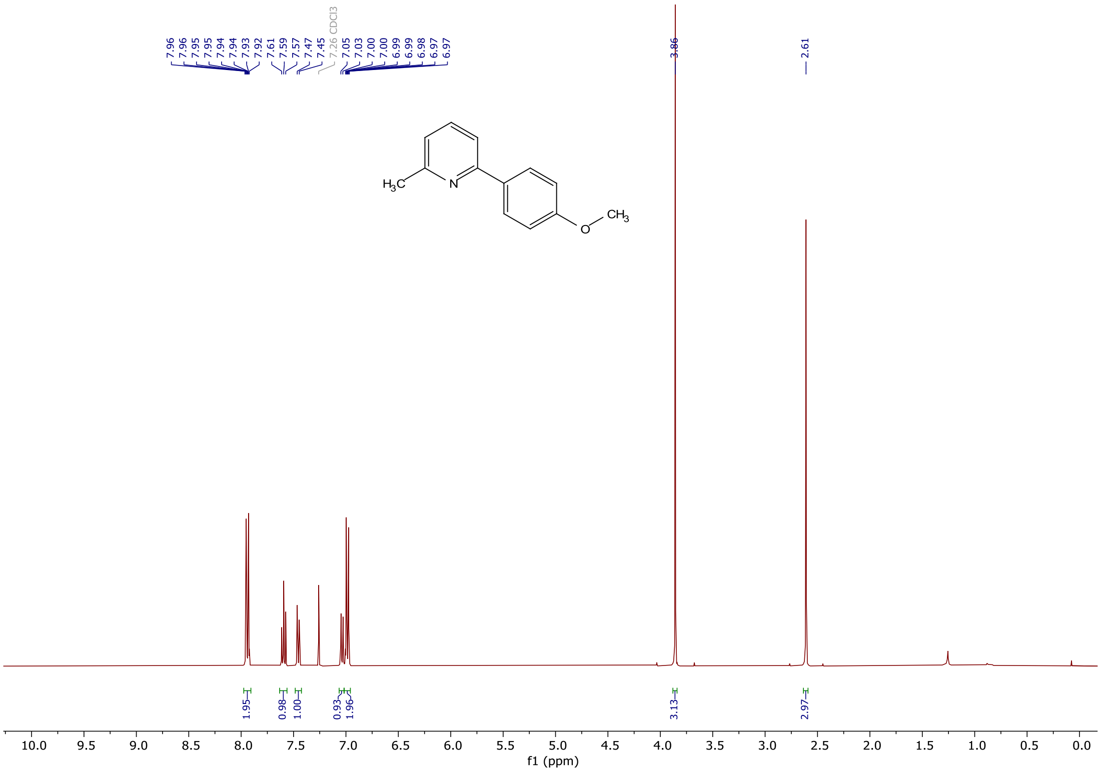

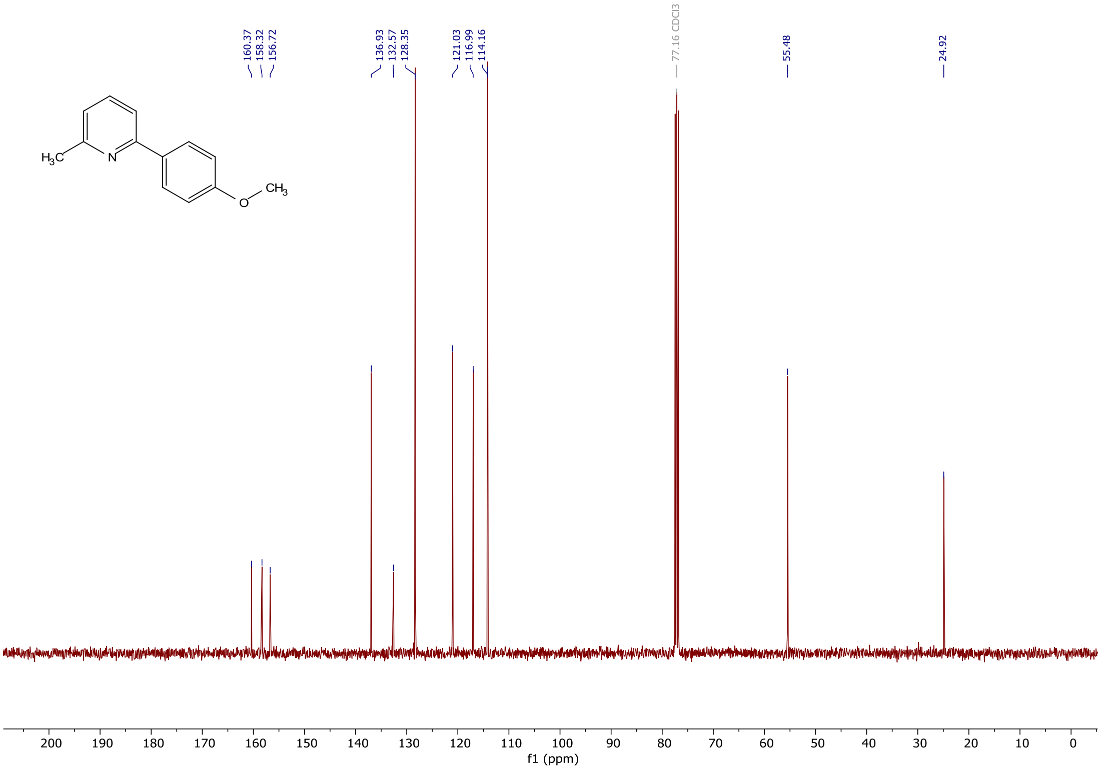

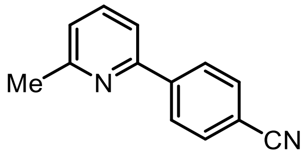

4-(6-methylpyridin-2-yl)benzonitrile (3u)

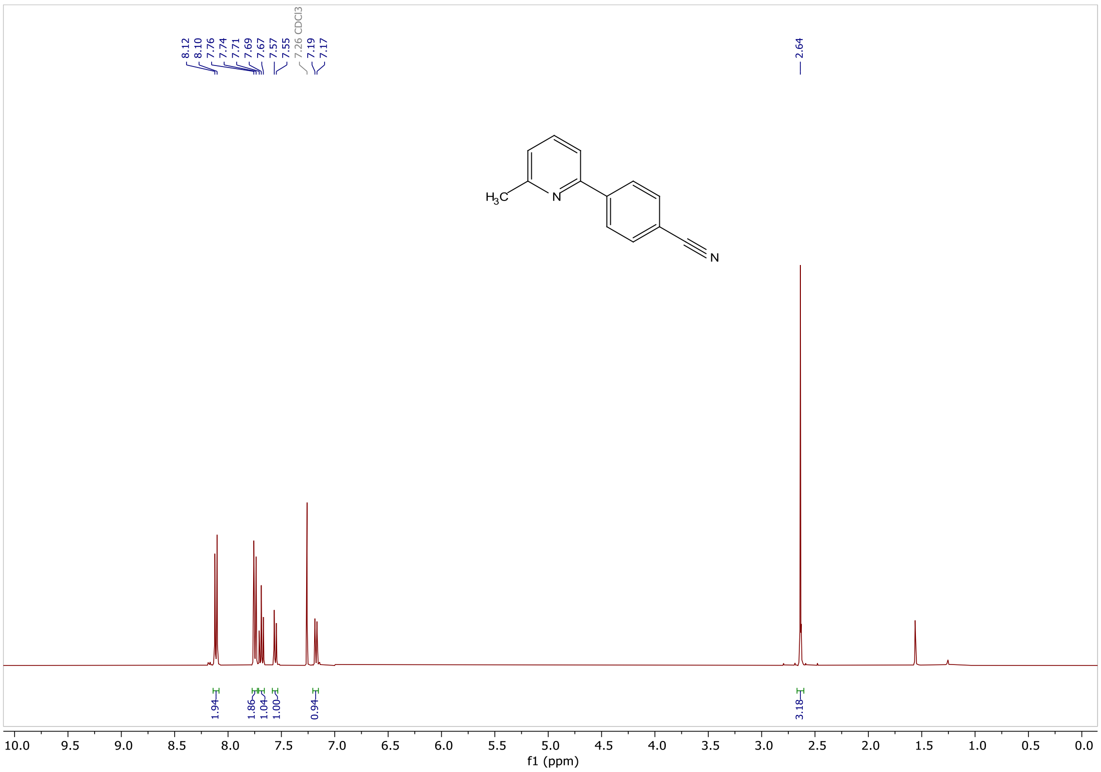

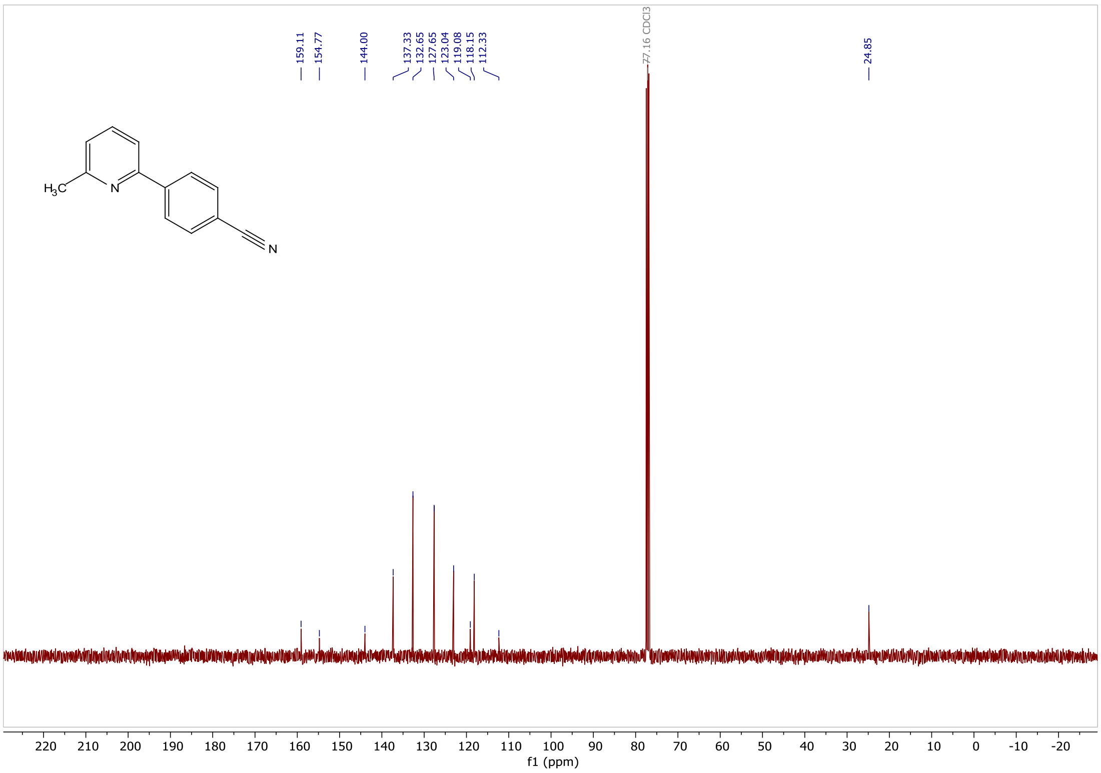

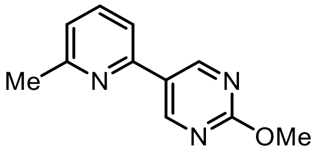

2-methoxy-5-(6-methylpyridin-2-yl)pyrimidine (3v)

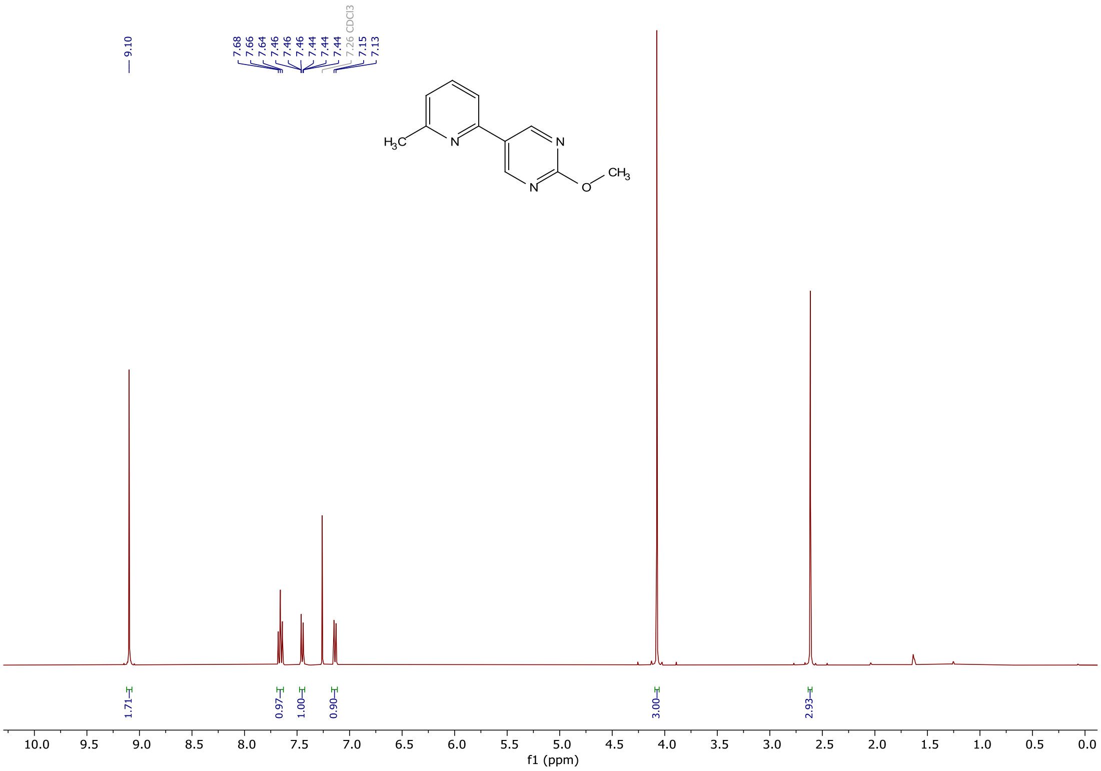

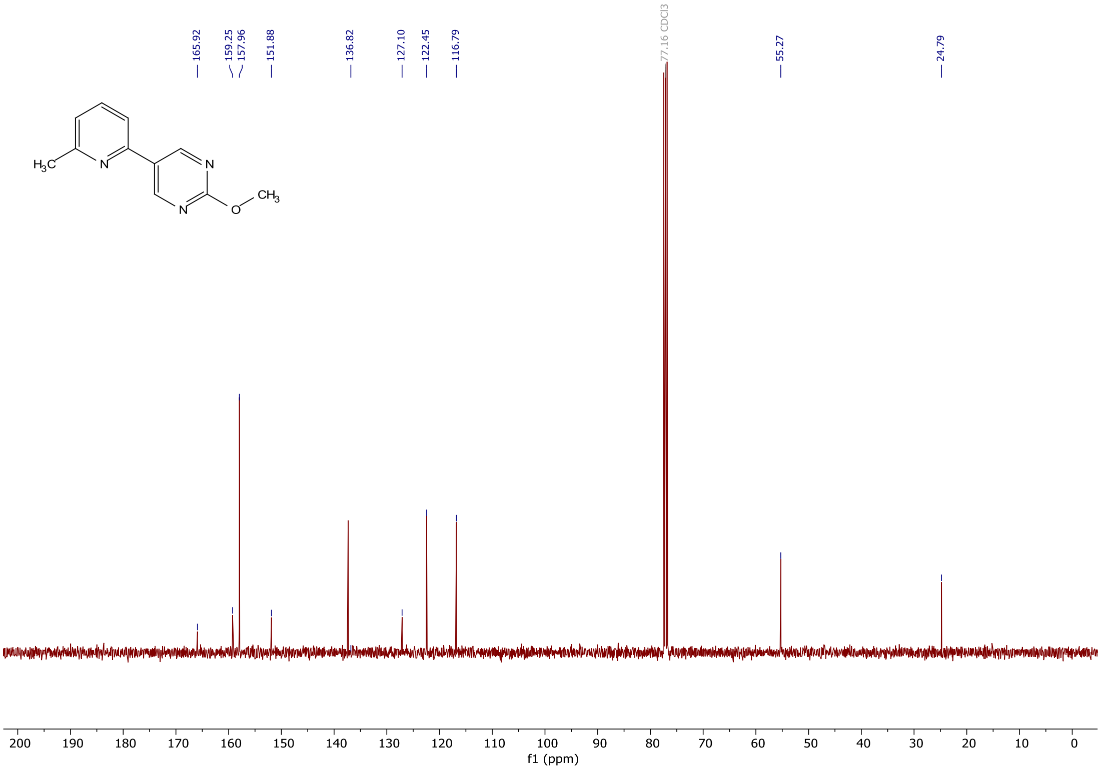

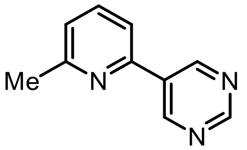

5-(6-methylpyridin-2-yl)pyrimidine (3w)

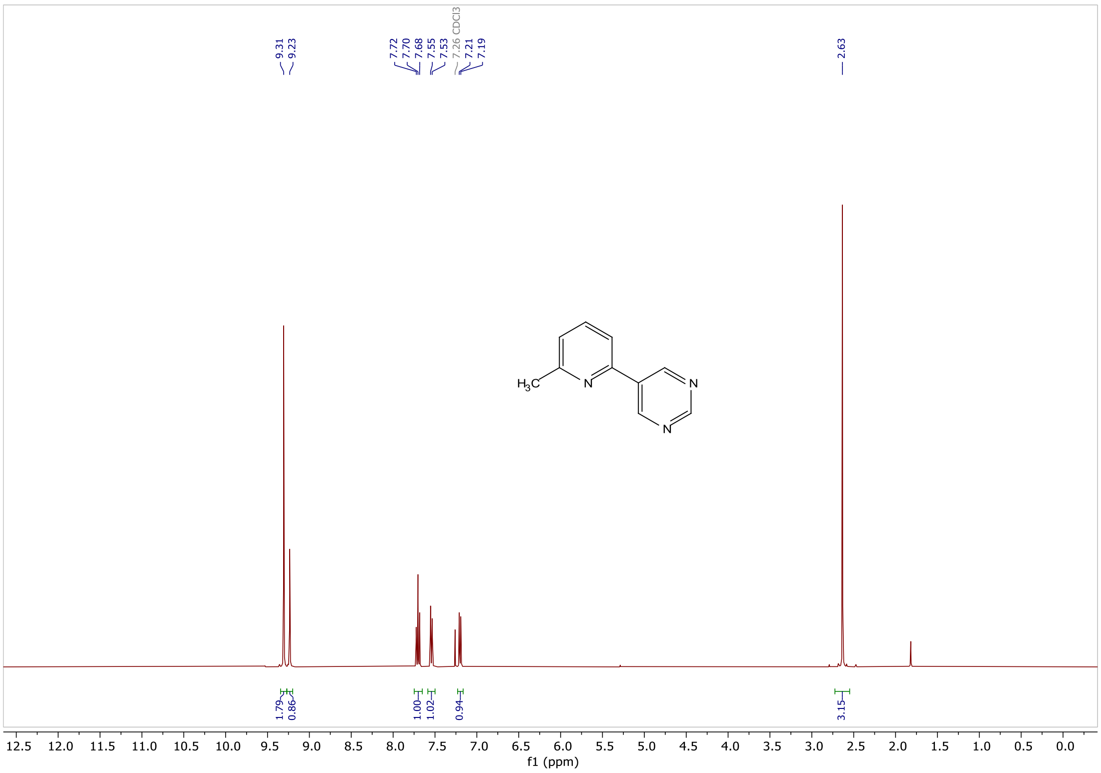

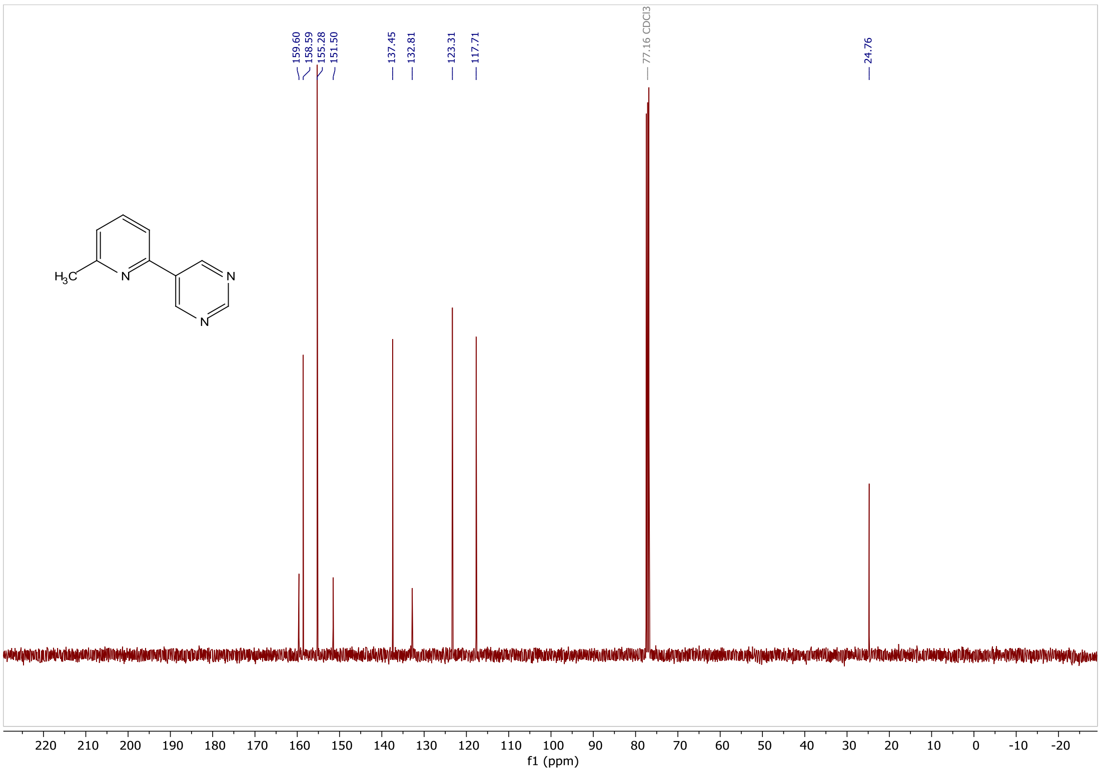

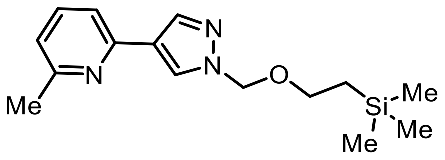

2-methyl-6-(1-((2-(trimethylsilyl)ethoxy)methyl)-1H-pyrazol-4-yl)pyridine (3x)

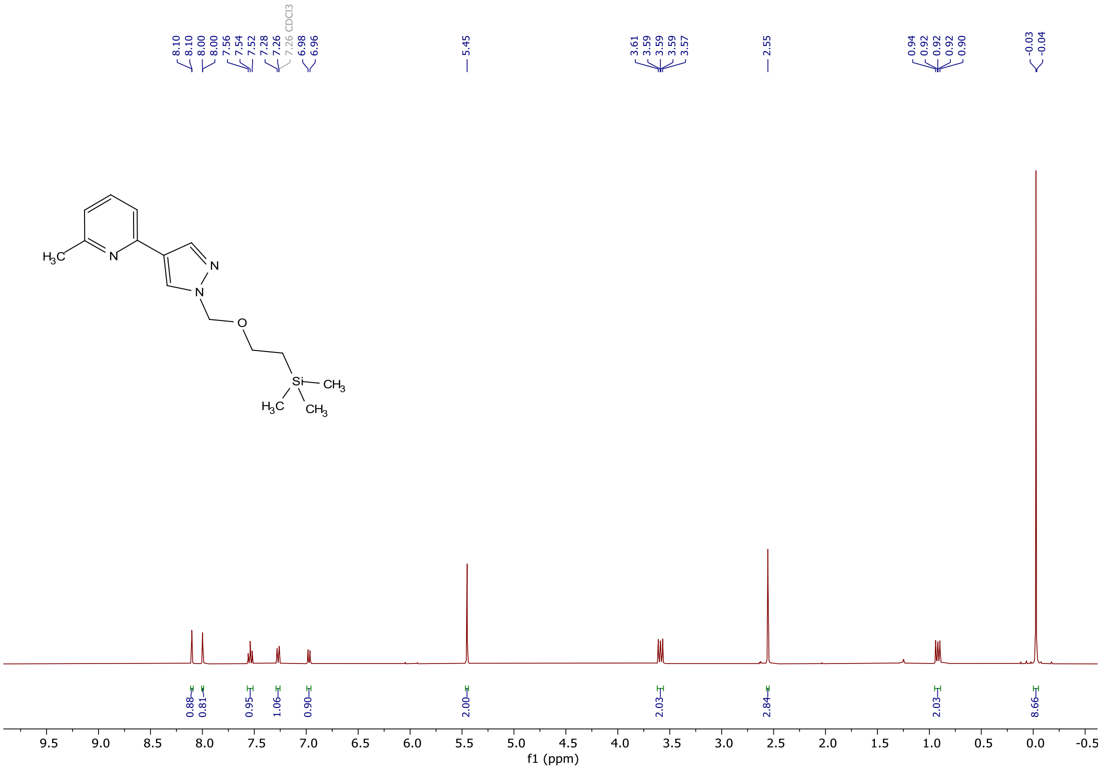

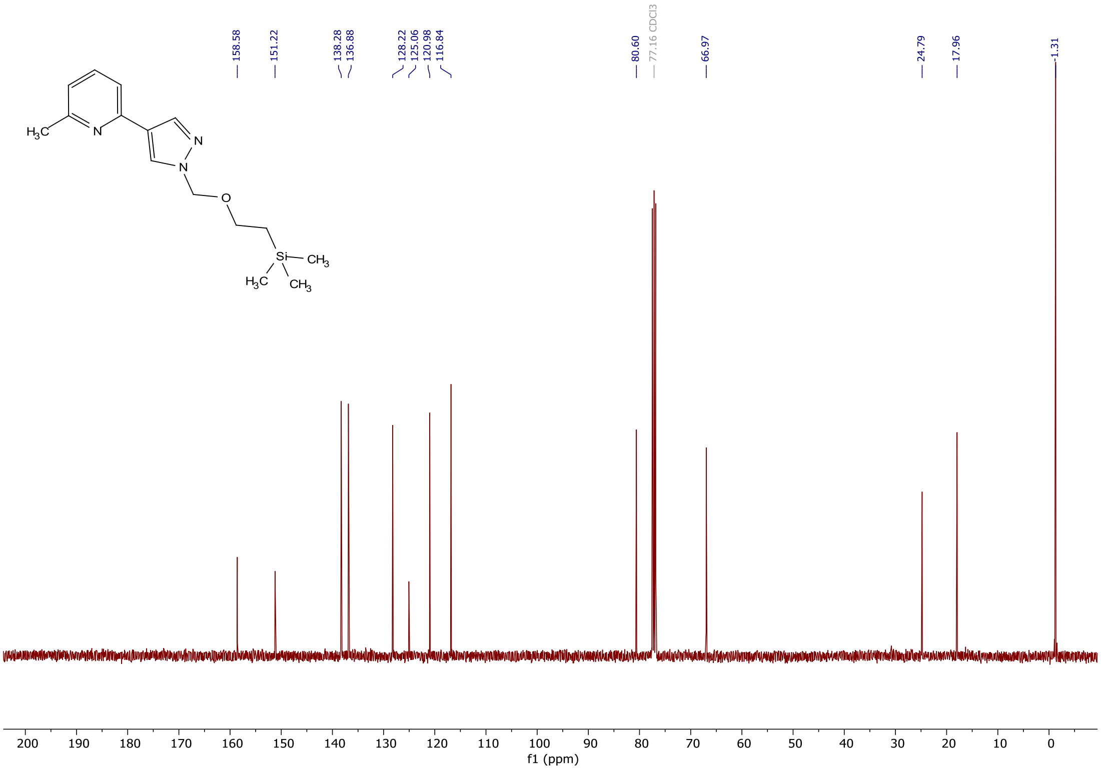

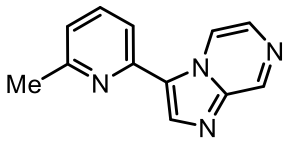

3-(6-methylpyridin-2-yl)imidazo[1,2-a]pyrazine (3y)

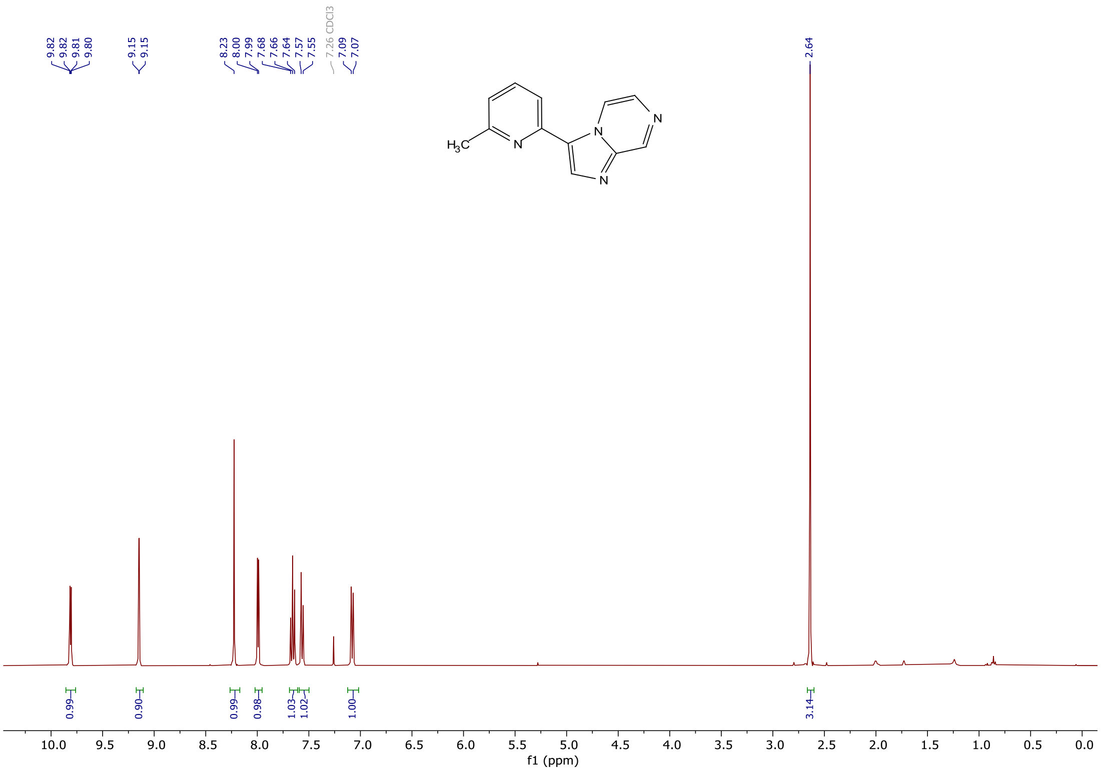

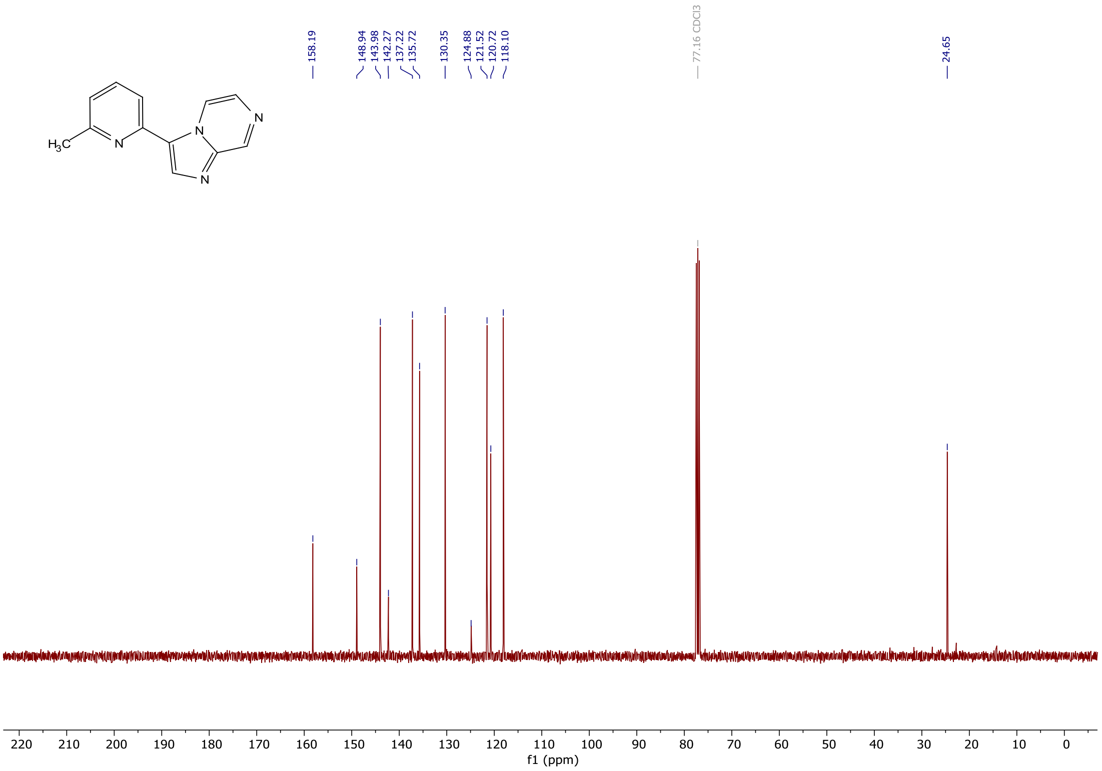

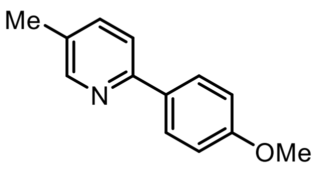

2-(4-methoxyphenyl)-5-methylpyridine (3z)

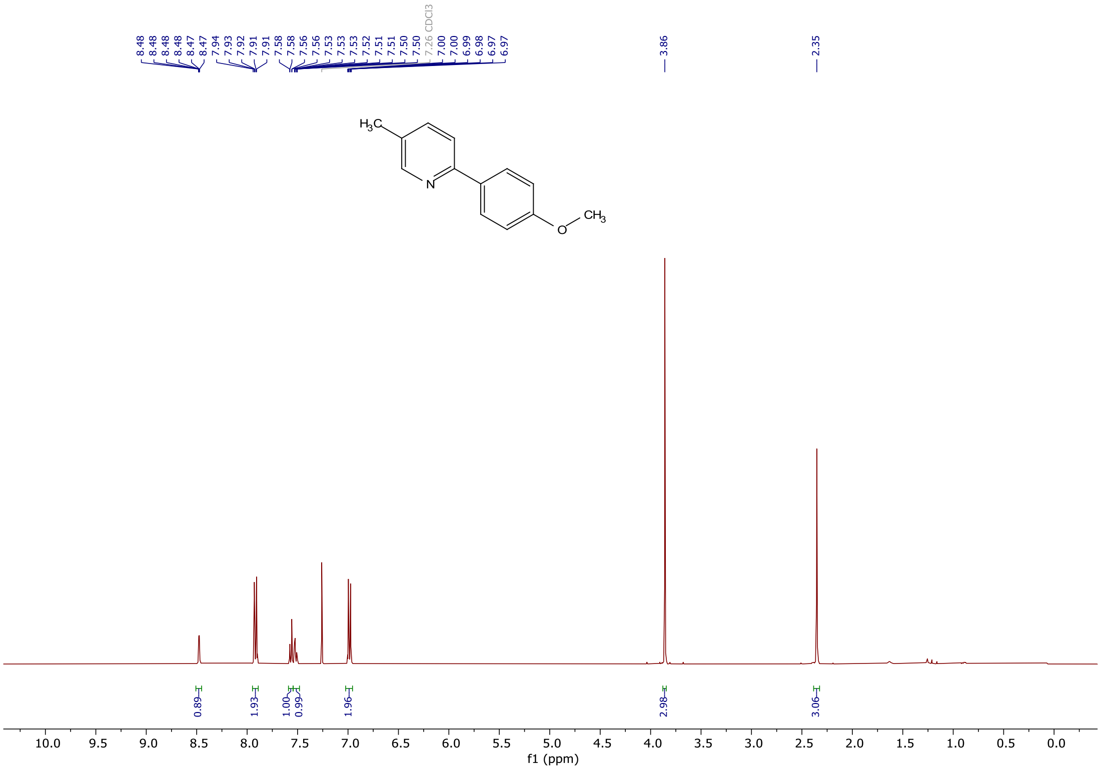

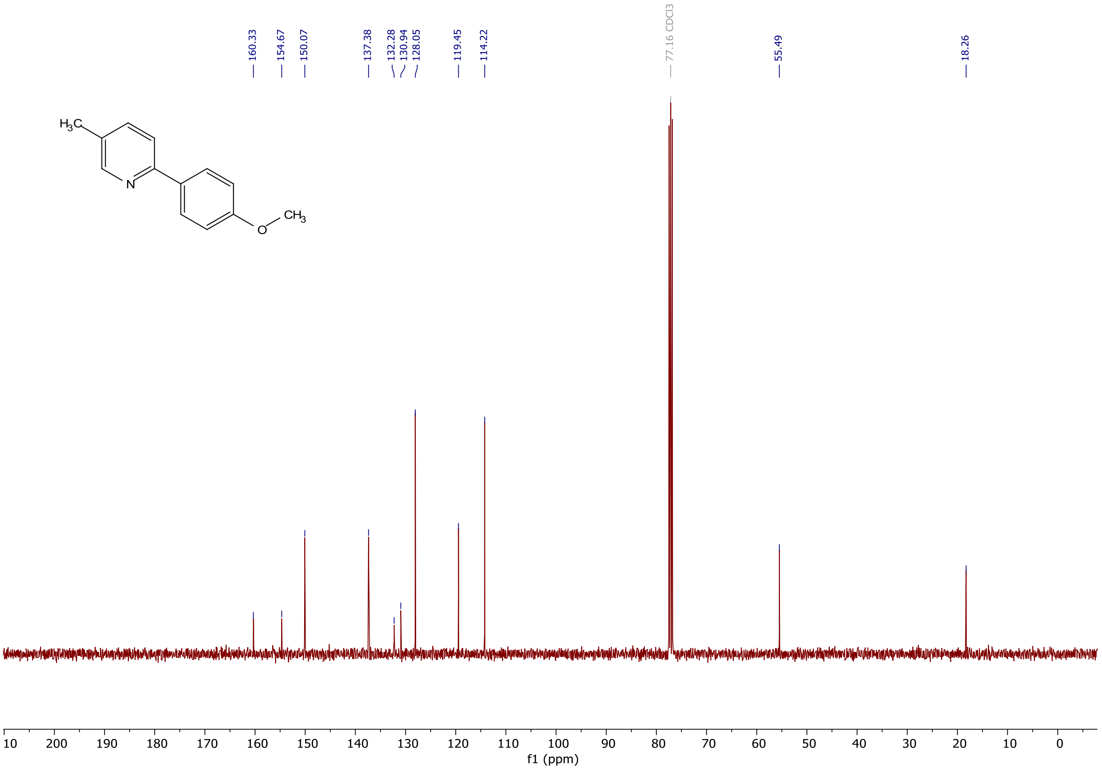

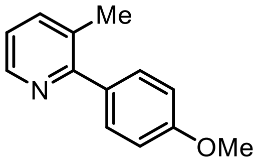

2-(4-methoxyphenyl)-3-methylpyridine (3aa)

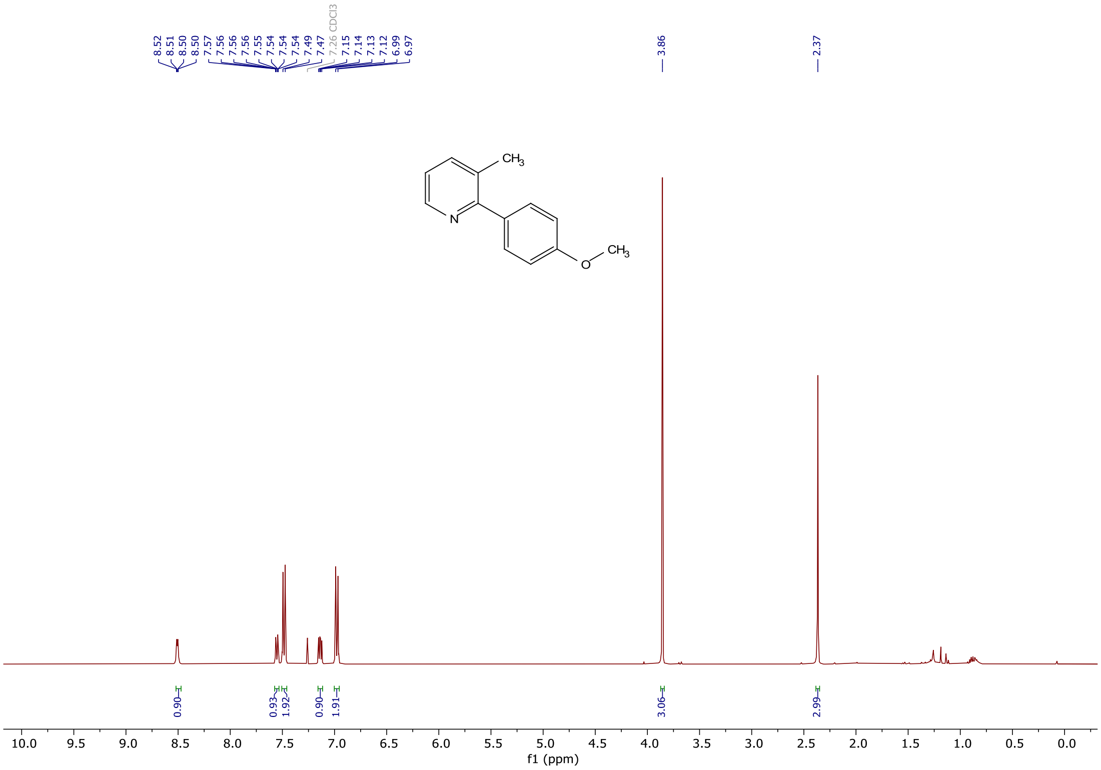

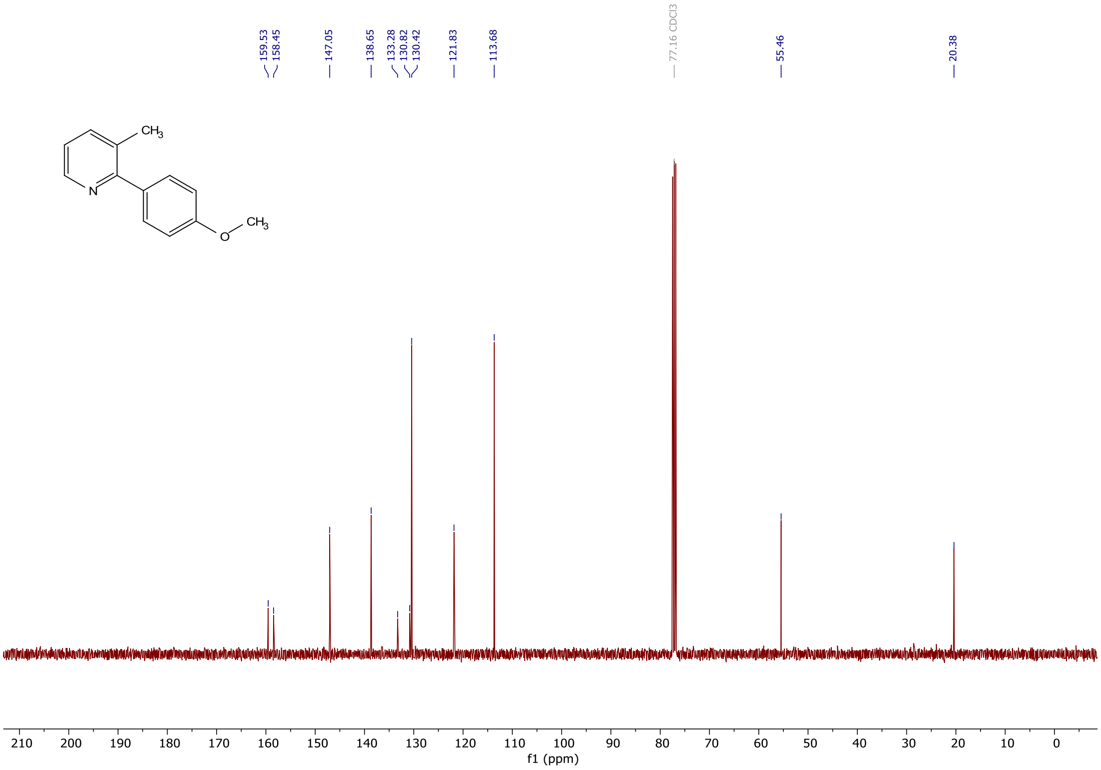

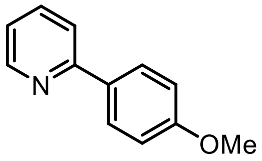

2-(4-methoxyphenyl)pyridine (3ab)

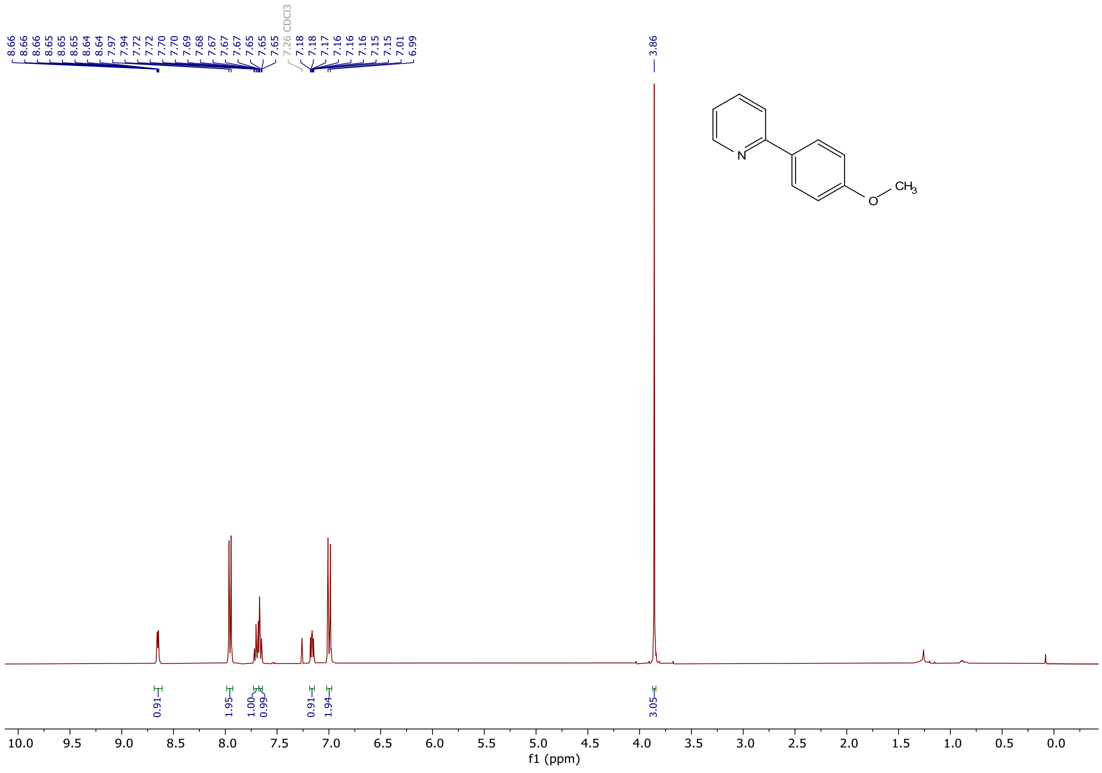

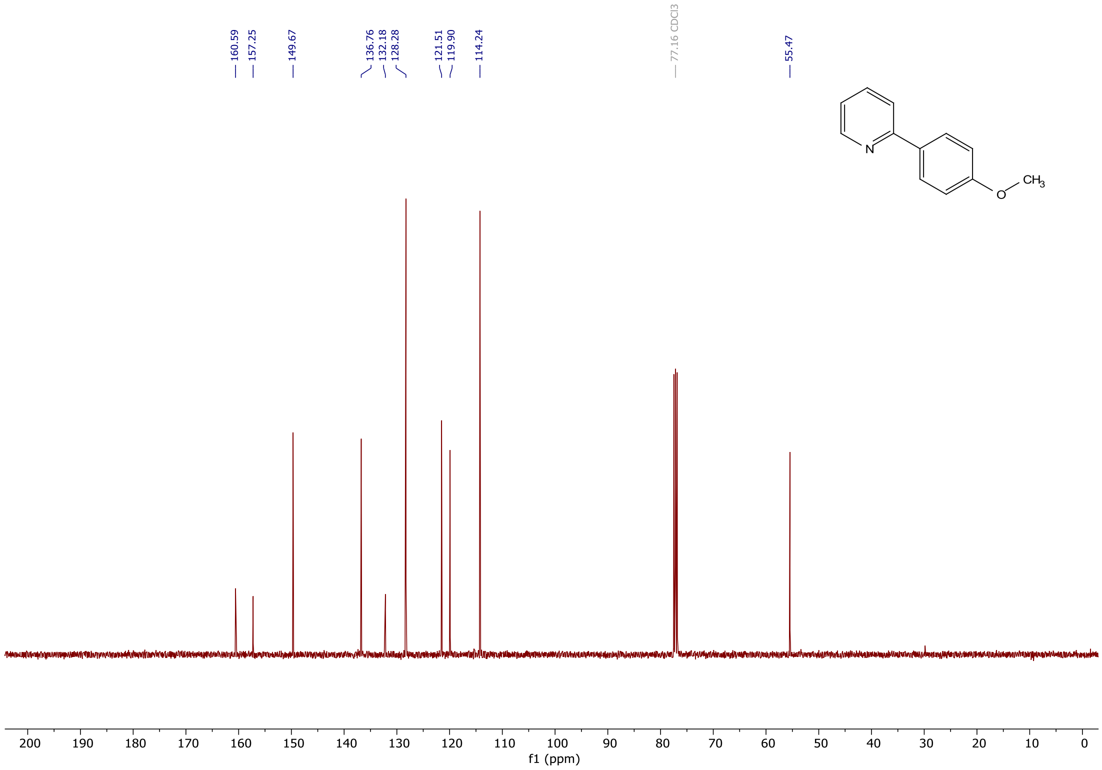

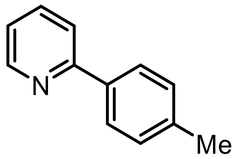

2-(p-tolyl)pyridine (3ac)

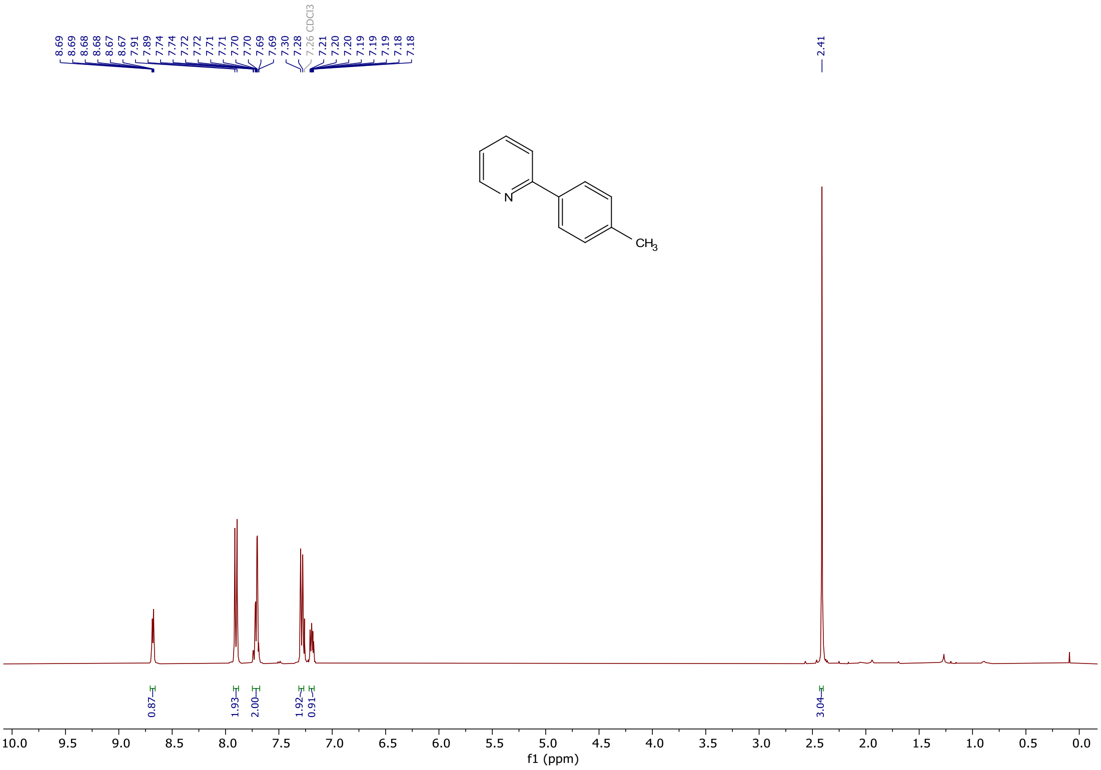

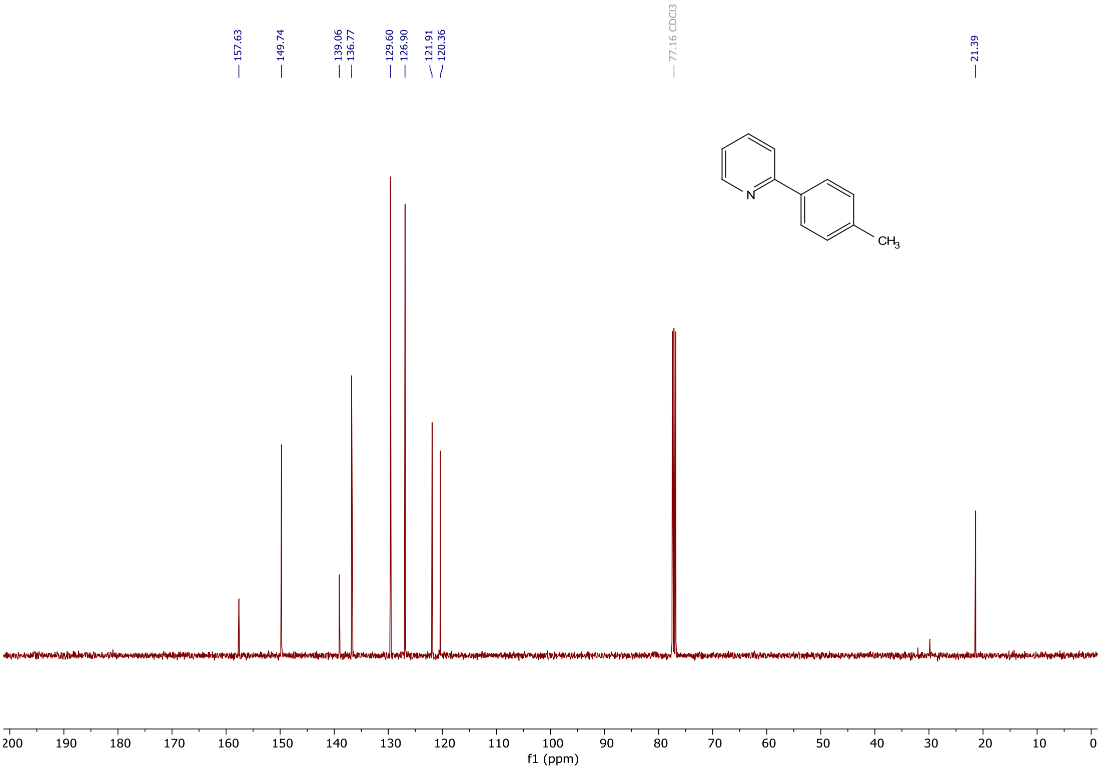

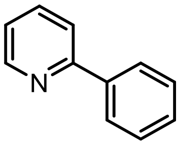

2-phenylpyridine (3ad)

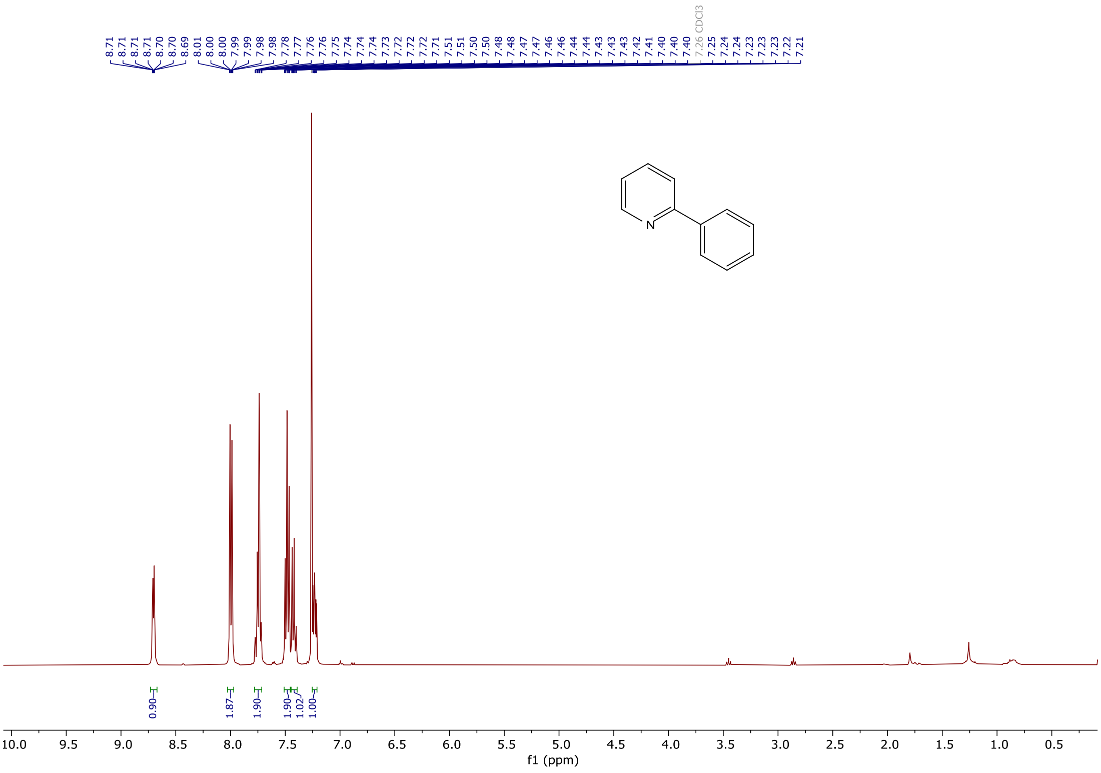

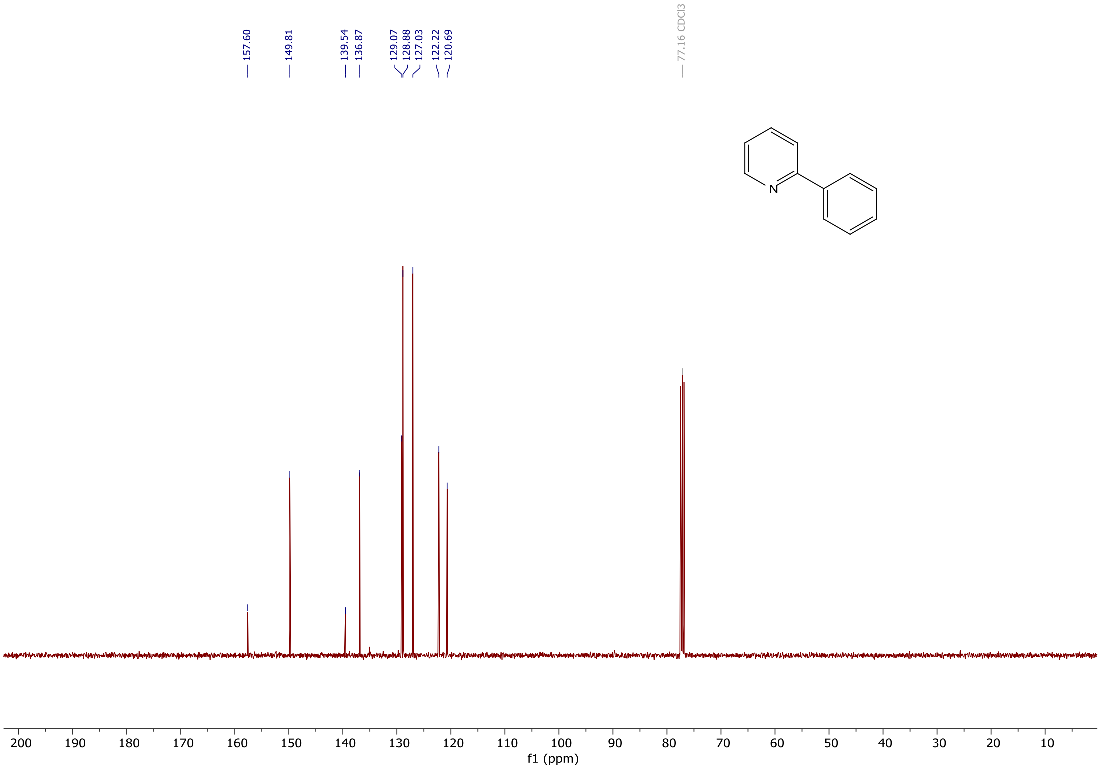

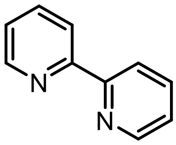

2,2'-bipyridine (3ae)

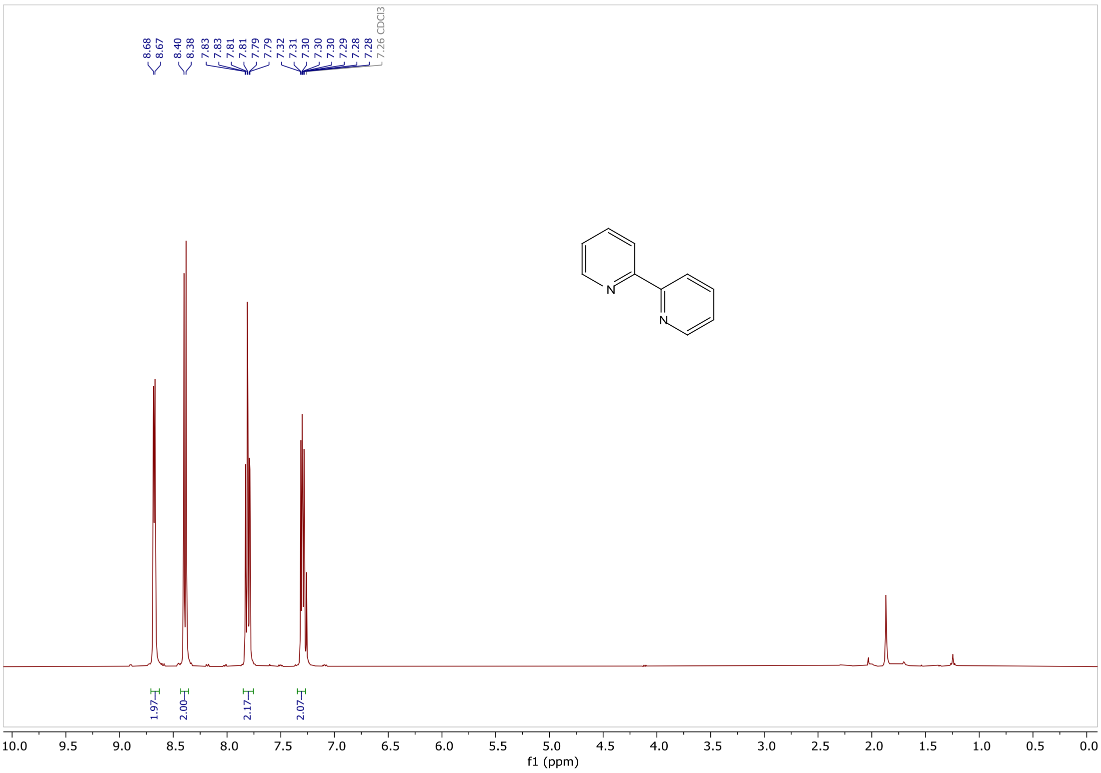

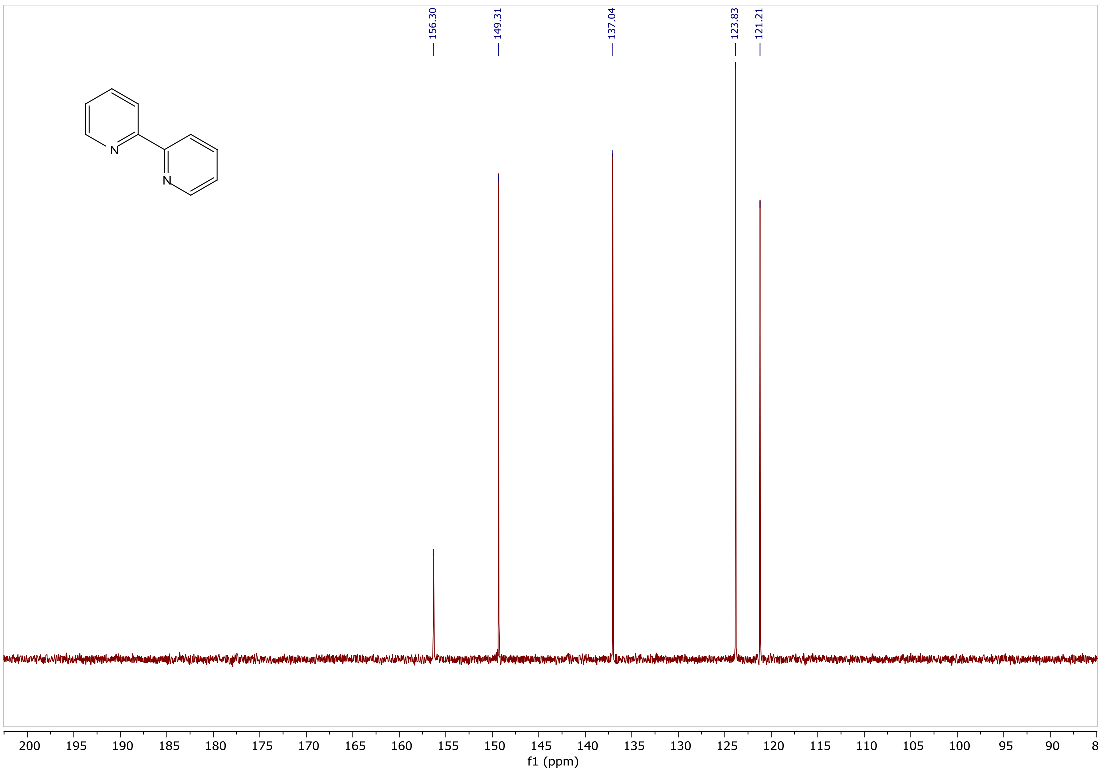

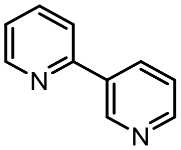

2,3'-bipyridine (3af)

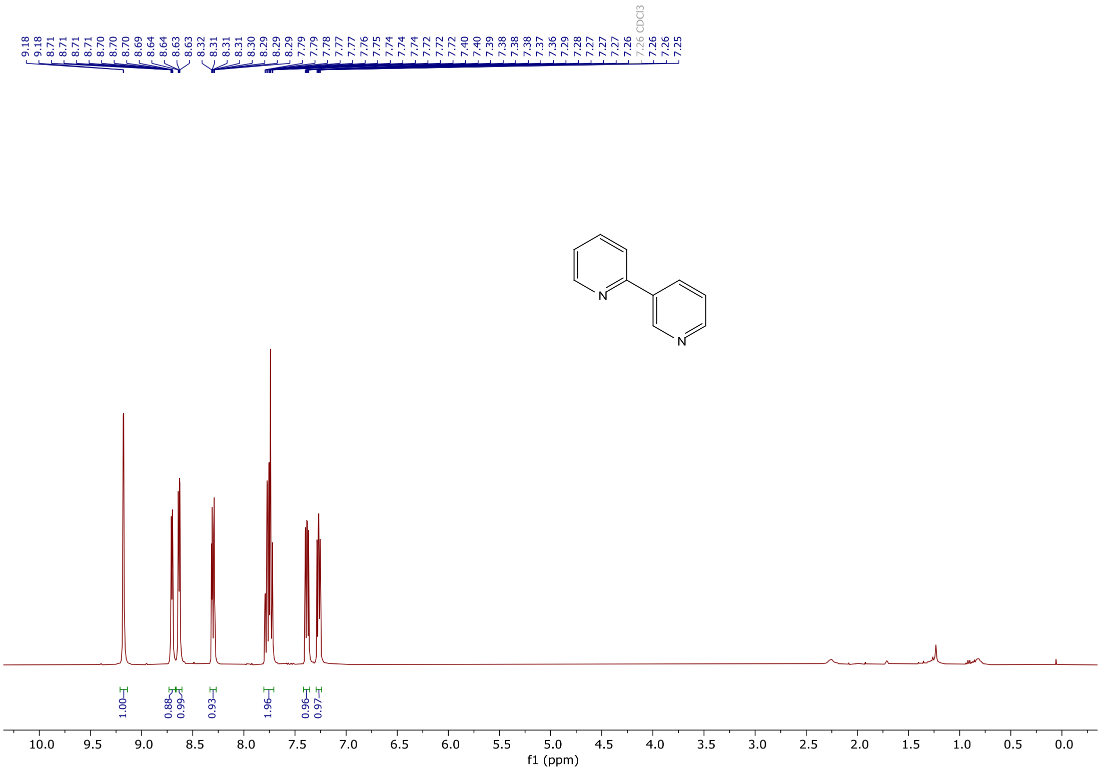

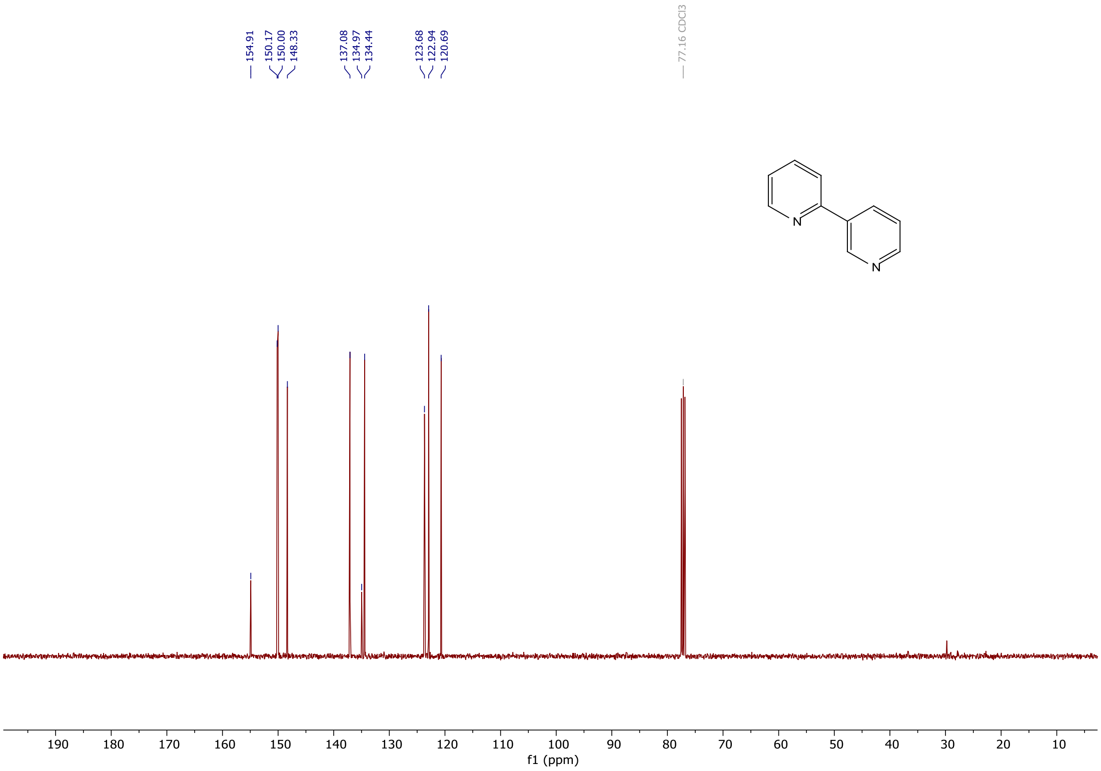

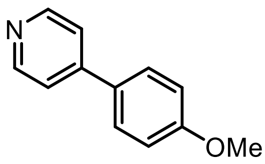

4-(4-methoxyphenyl)pyridine (3ag)

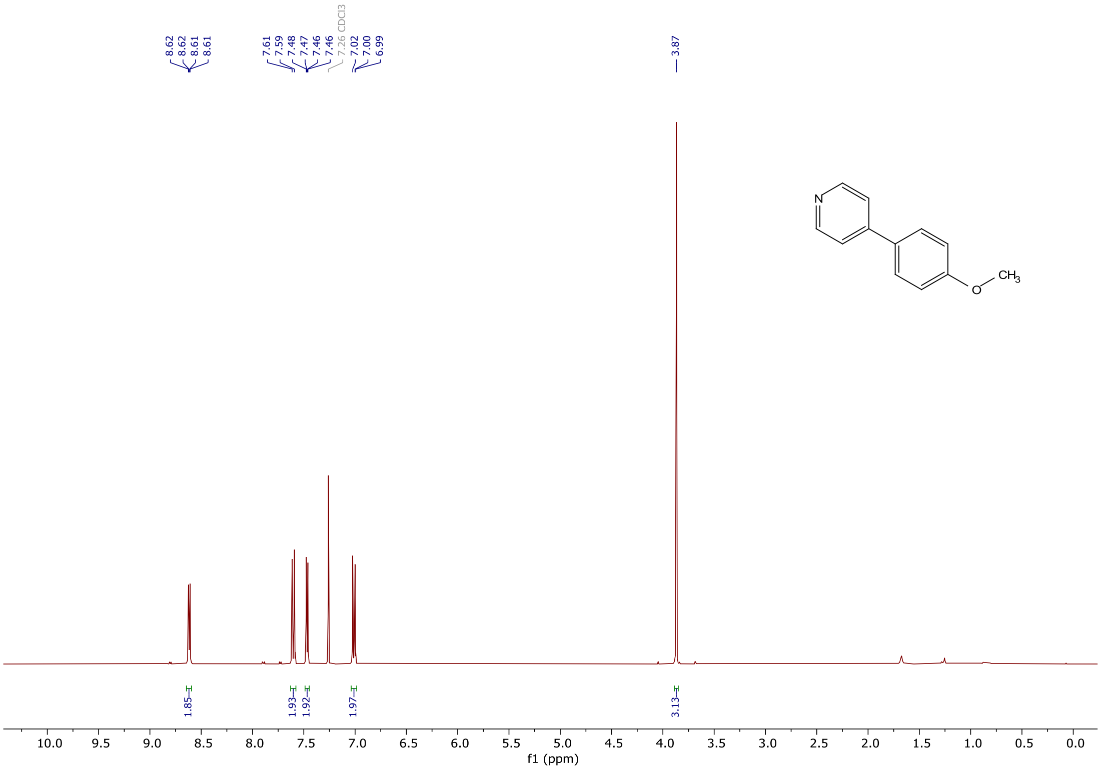

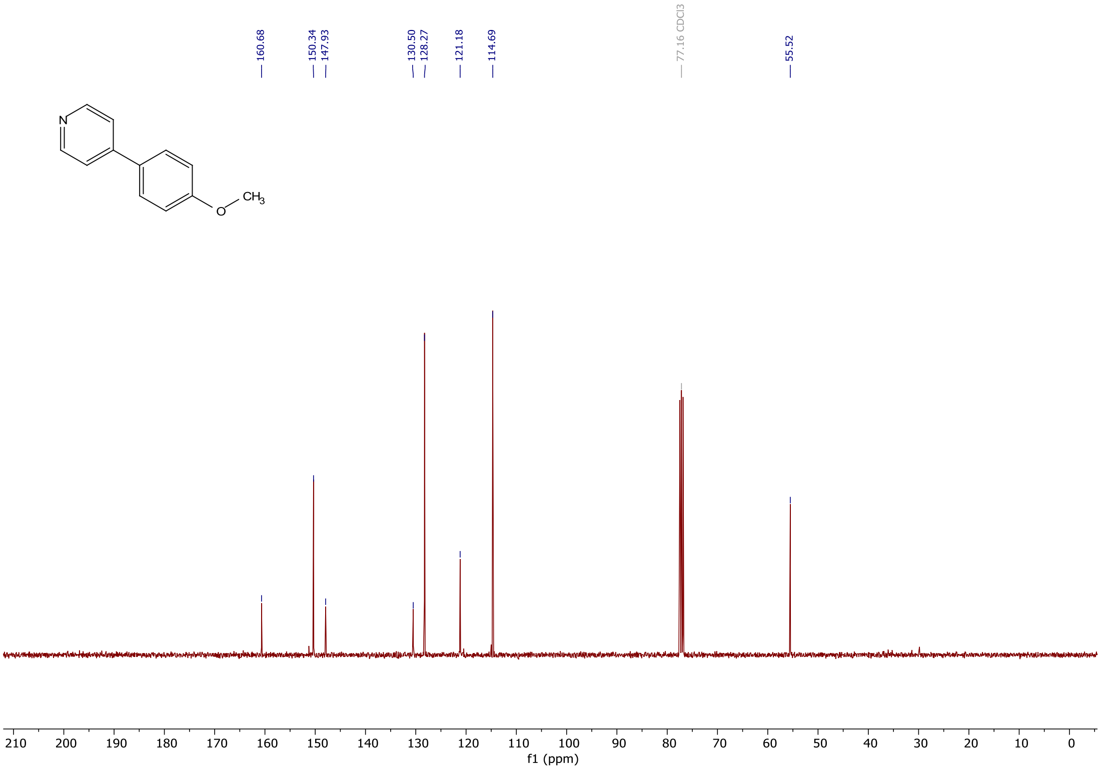

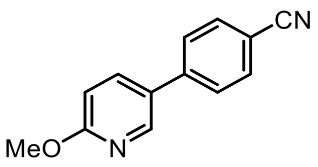

4-(6-methoxypyridin-3-yl)benzonitrile (3ah)

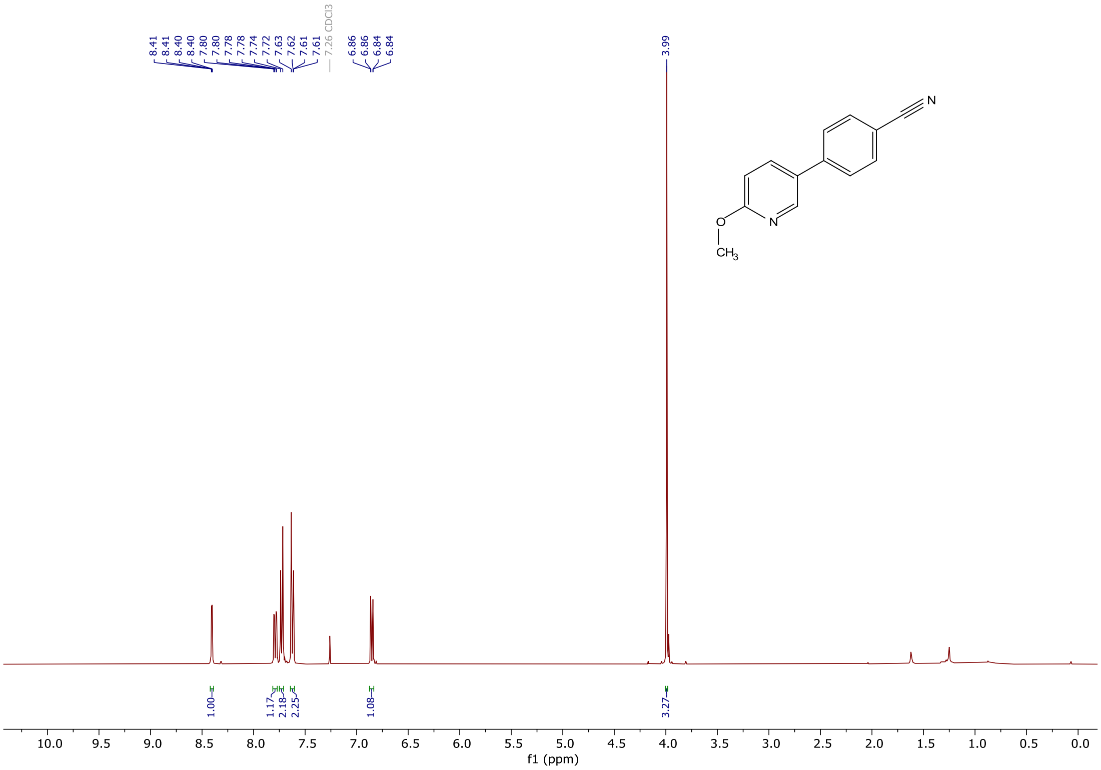

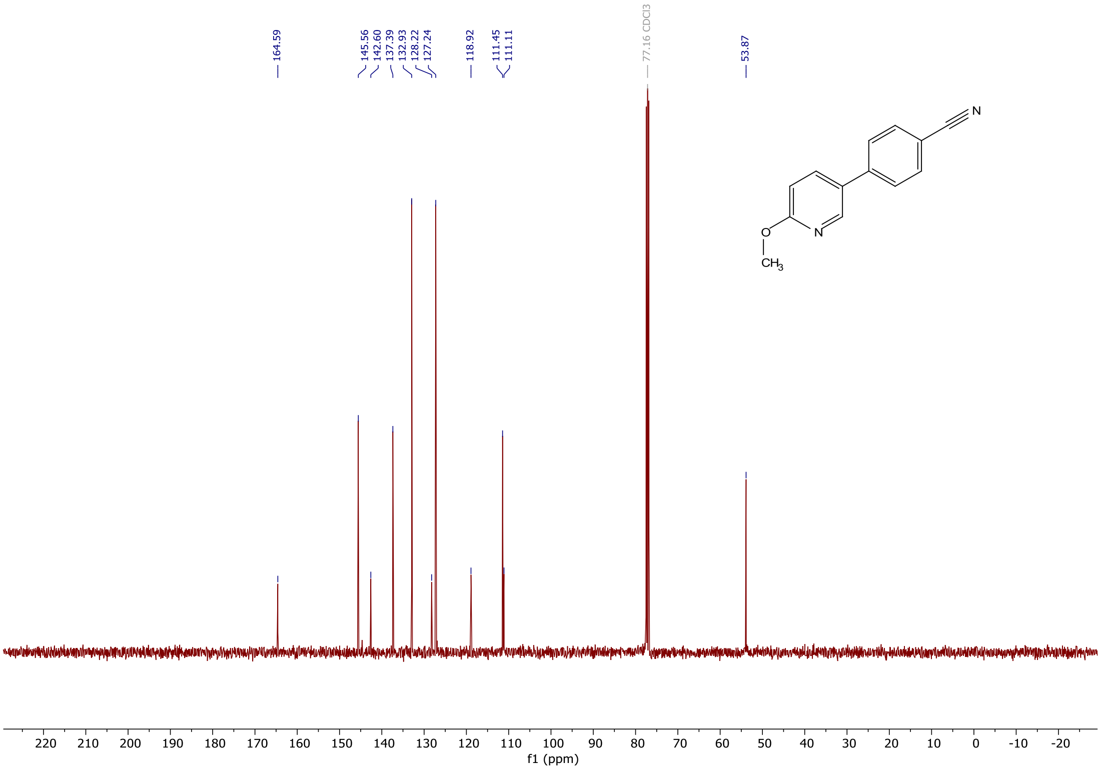

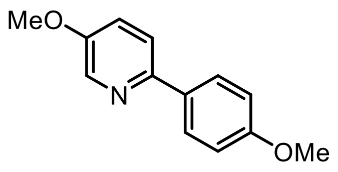

5-methoxy-2-(4-methoxyphenyl)pyridine (3ai)

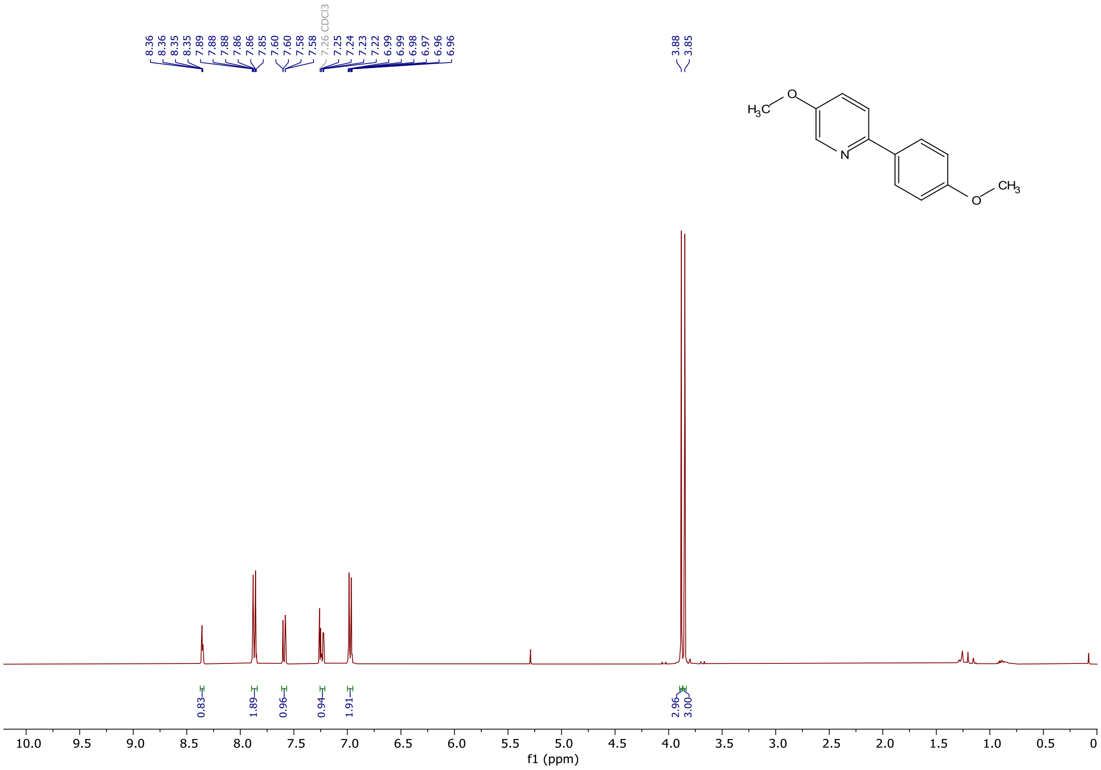

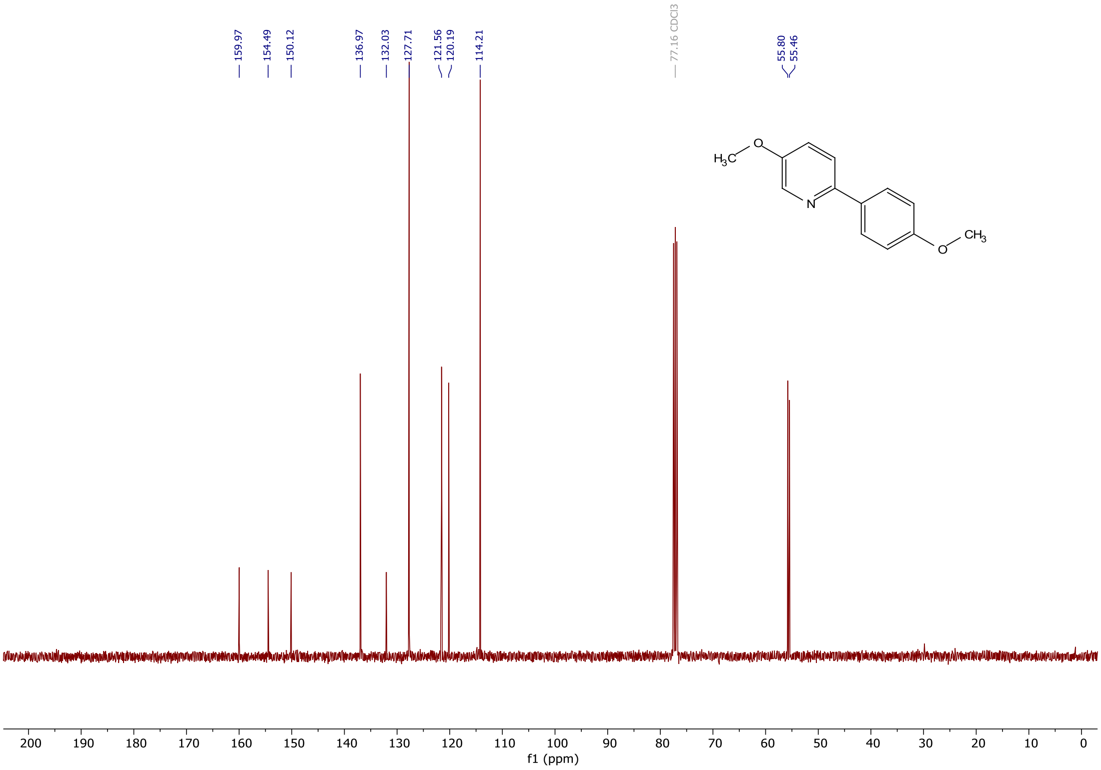

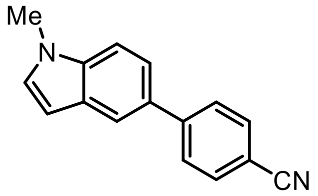

4-(1-methyl-1H-indol-5-yl)benzonitrile (3aj)

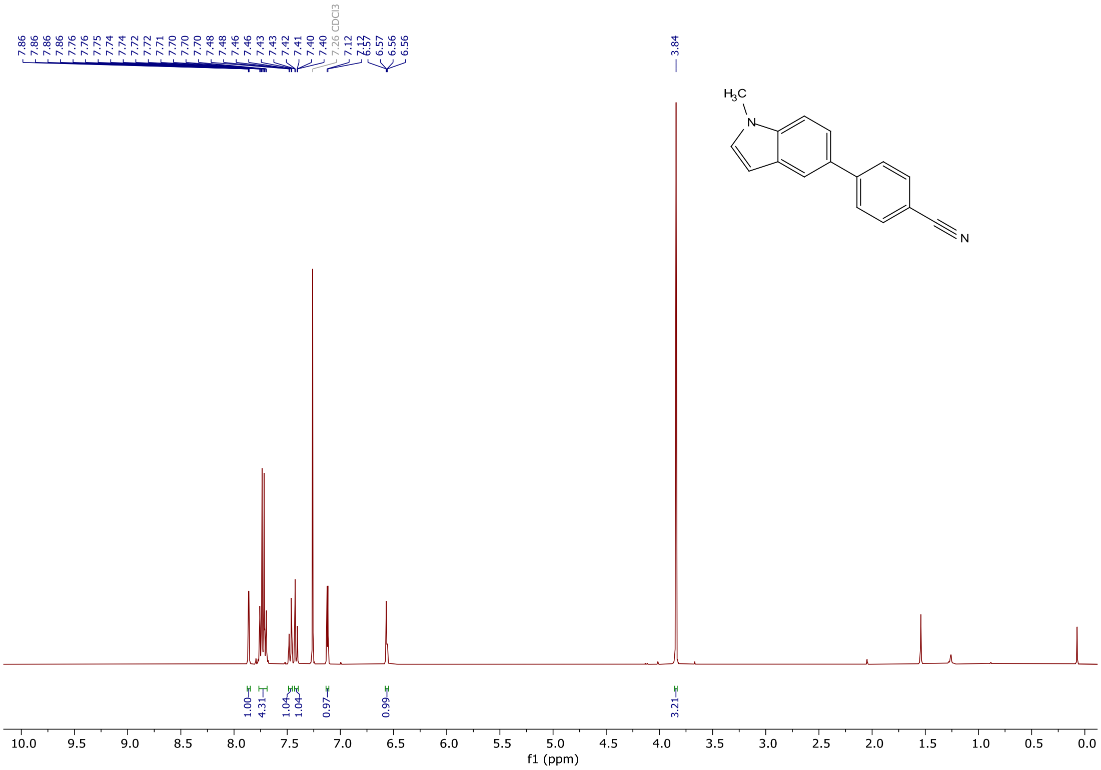

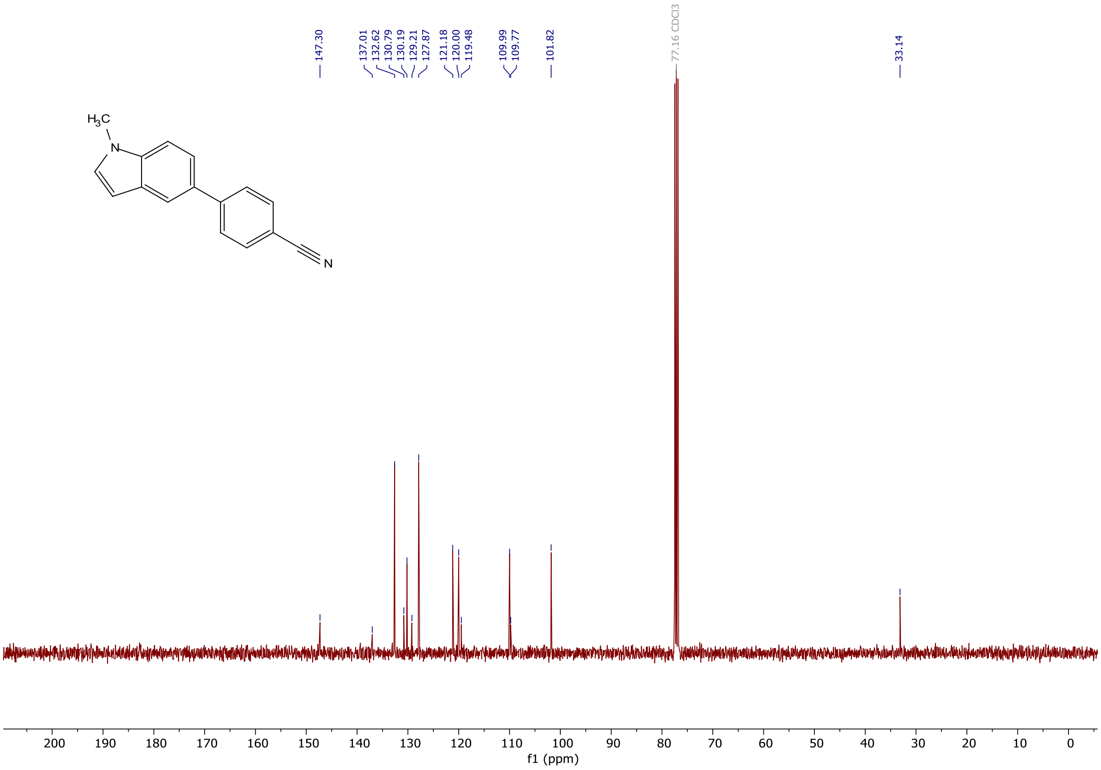

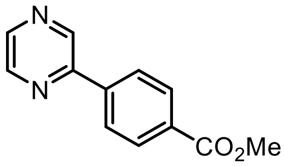

methyl 4-(pyrazin-2-yl)benzoate (3ak)

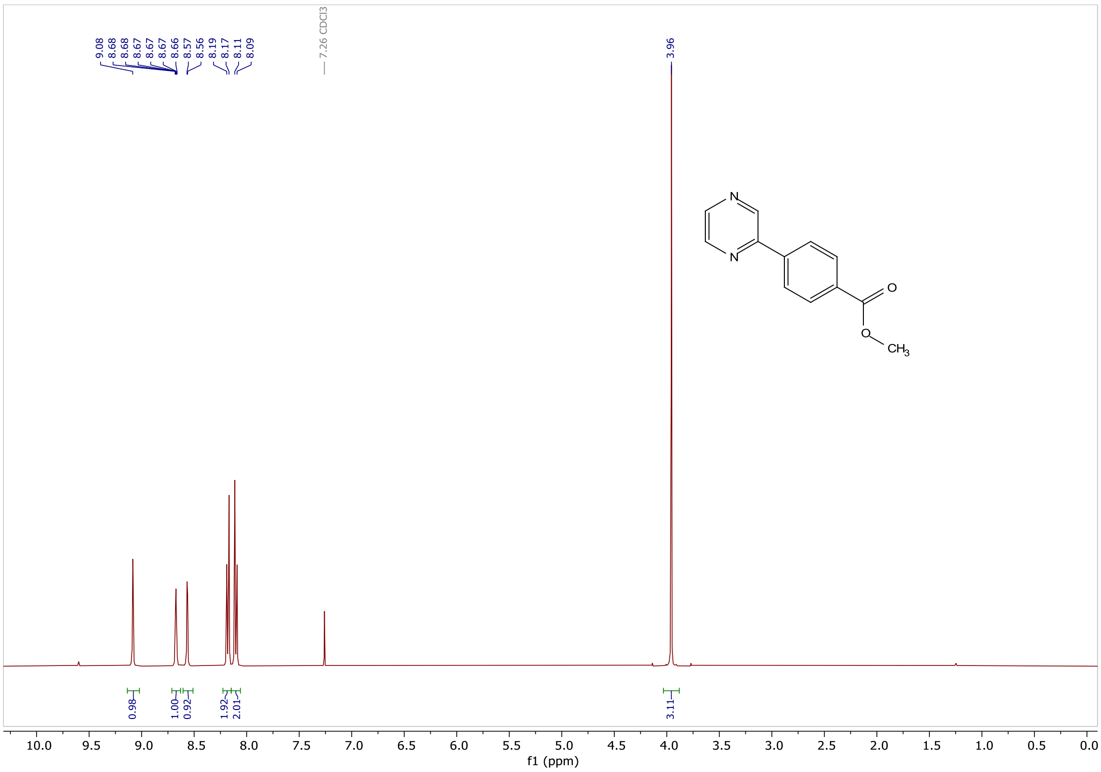

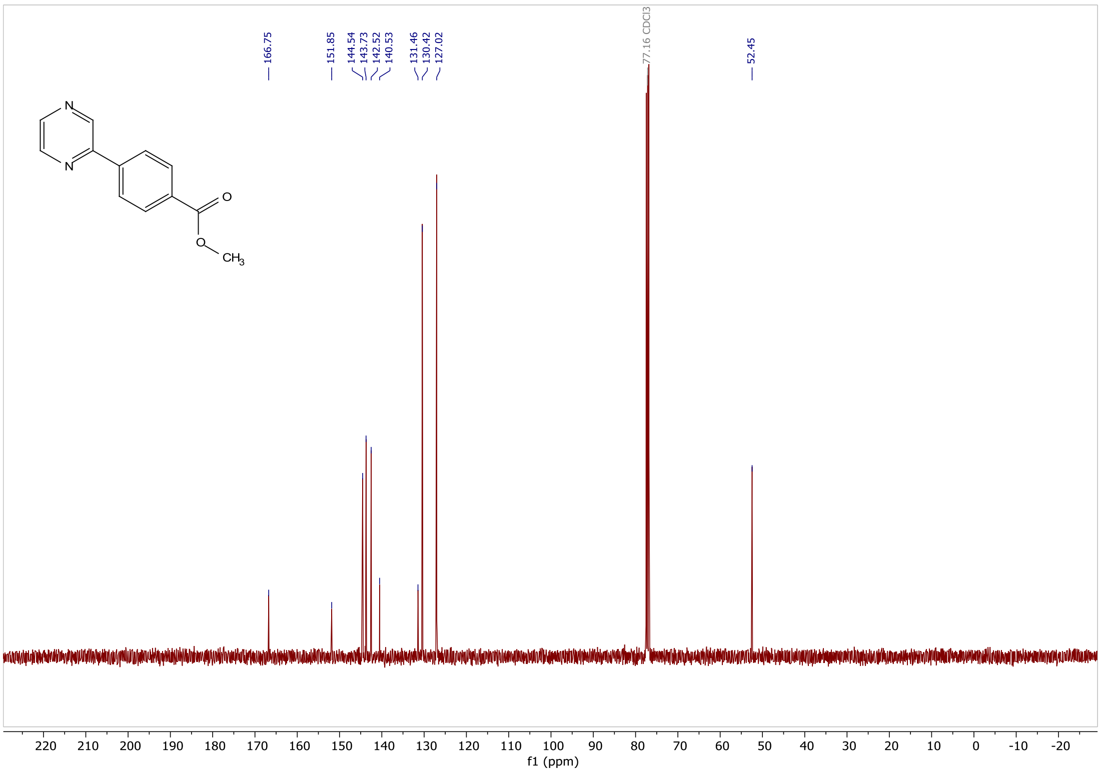

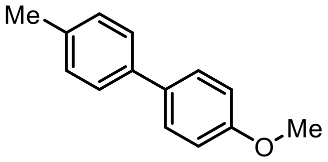

4-methoxy-4'-methyl-1,1'-biphenyl (3al)

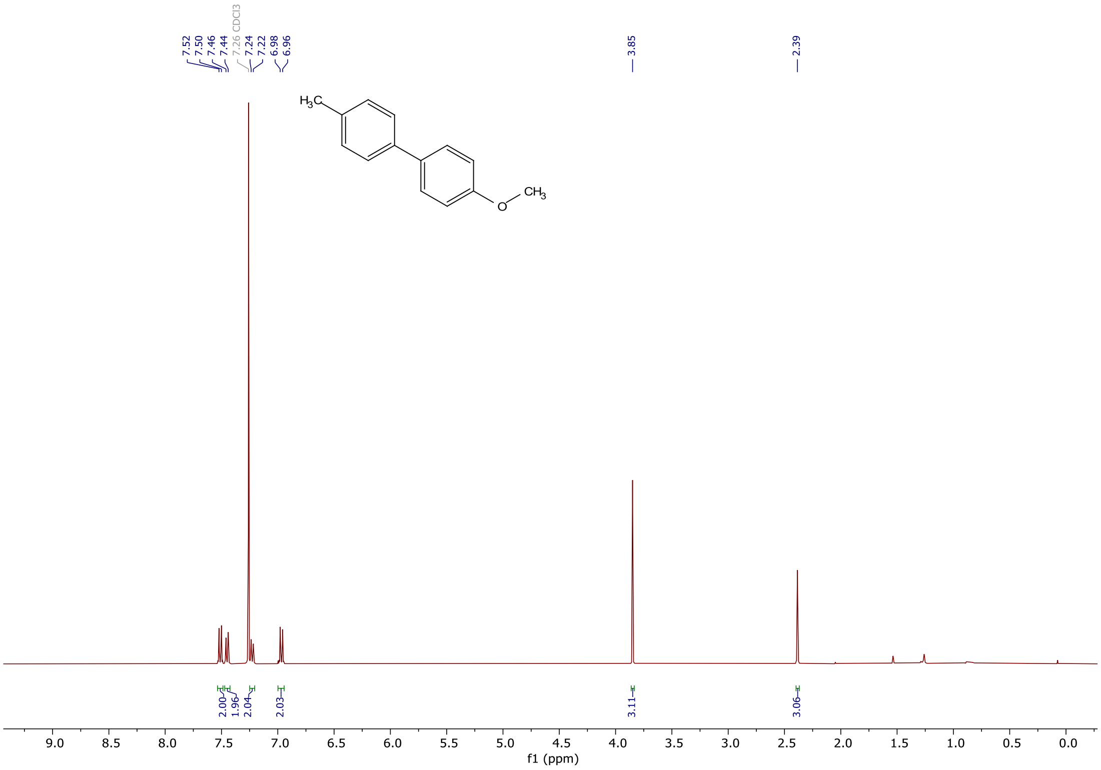

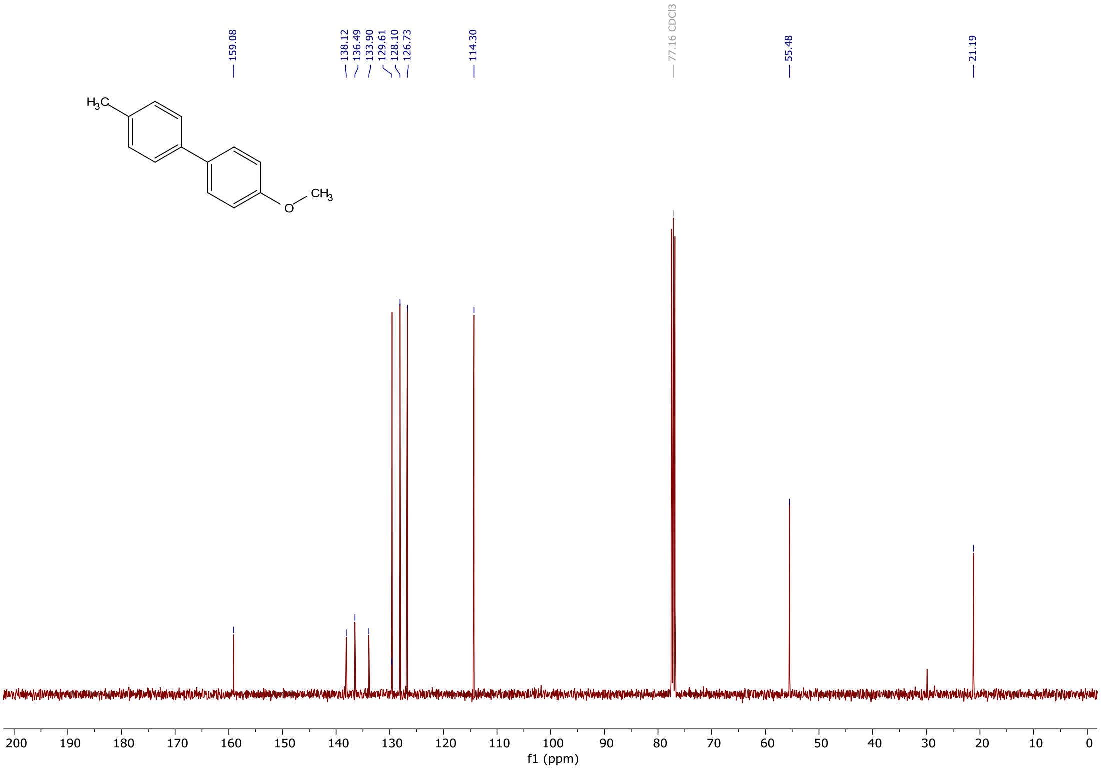

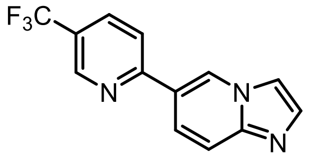

6-(5-(trifluoromethyl)pyridin-2-yl)imidazo[1,2-a]pyridine (3am)

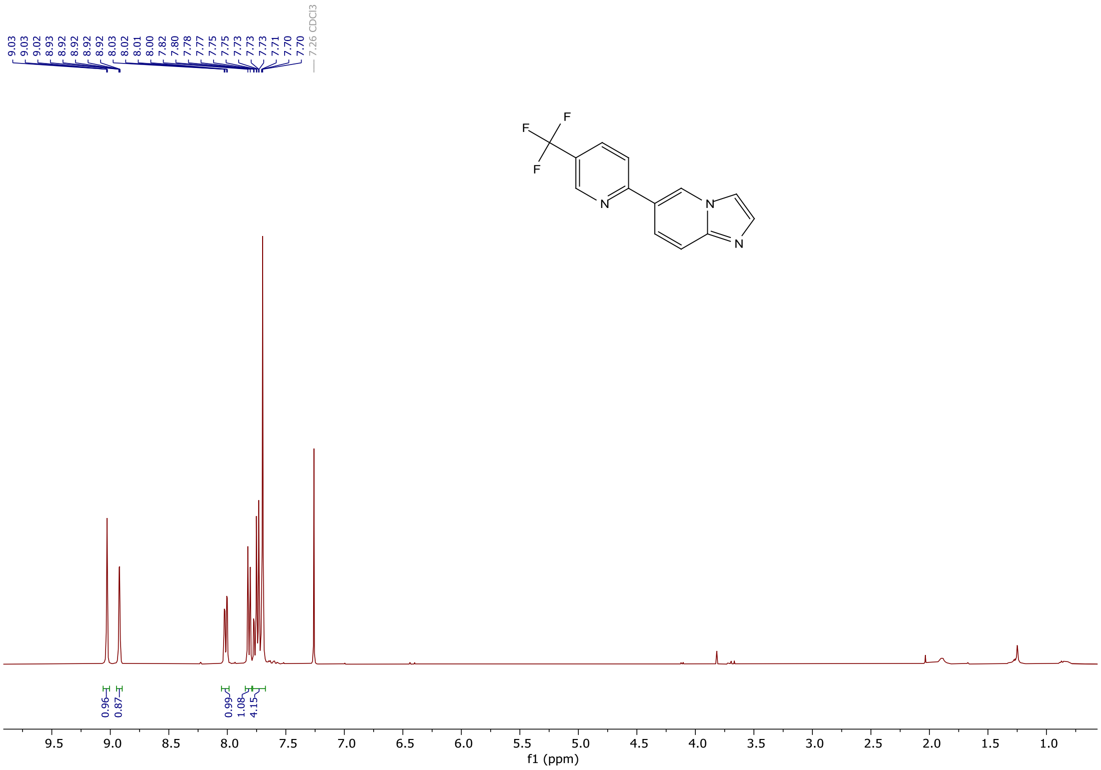

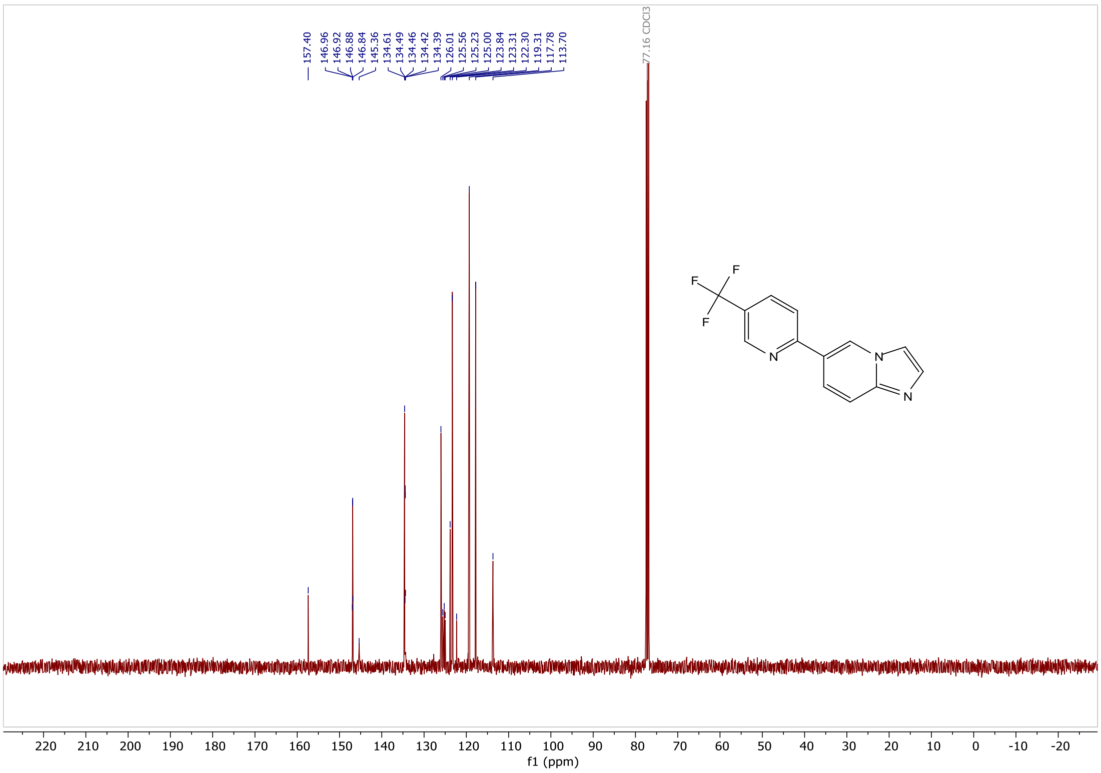

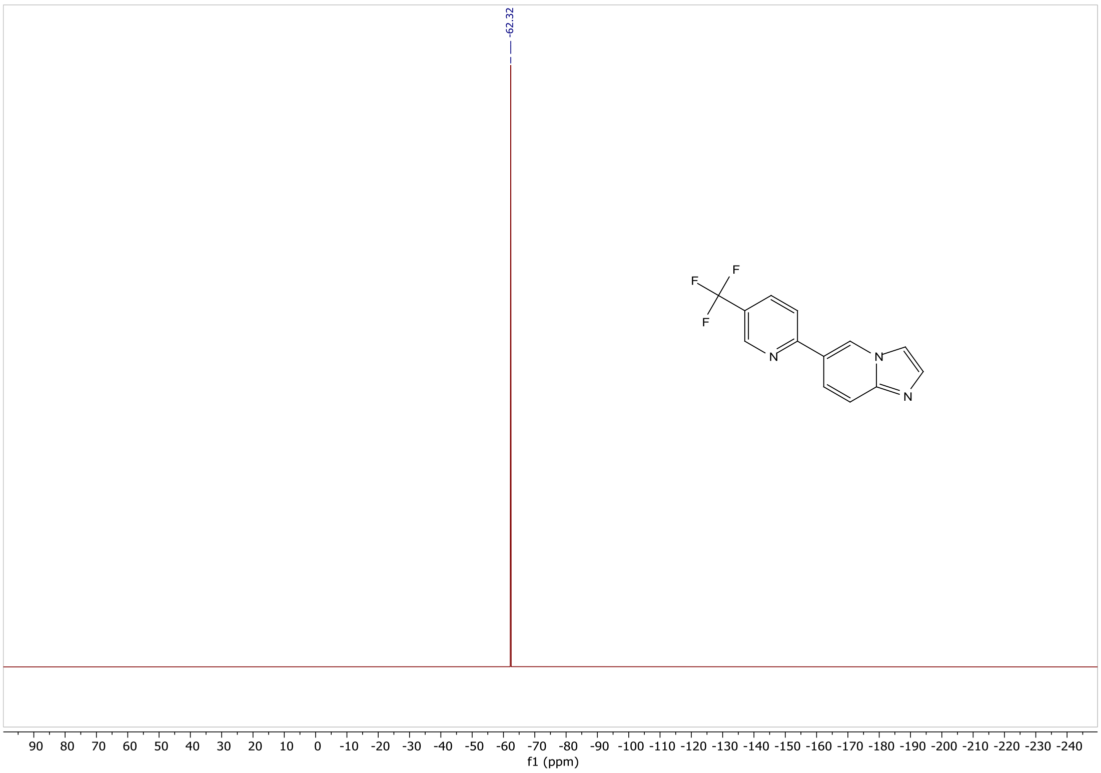
-62.32
90 80 70 60 50 40 30 20 10 0 -10 -20 -30 -40 -50 -60 -70 -80 -90 -100 -110 -120 -130 -140 -150 -160 -170 -180 -190 -200 -210 -220 -230 -240
f1 (ppm)

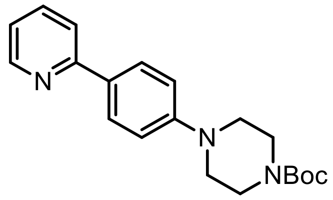

tert-butyl 4-(4-(pyridin-2-yl)phenyl)piperazine-1-carboxylate (3an)

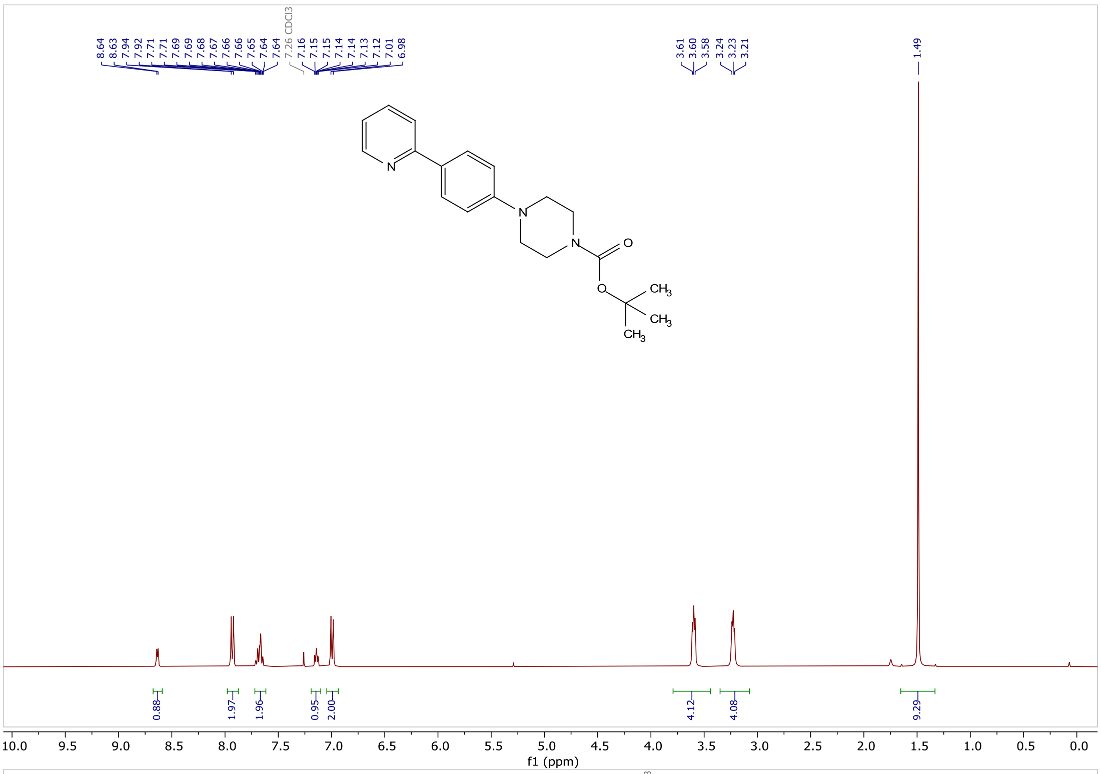

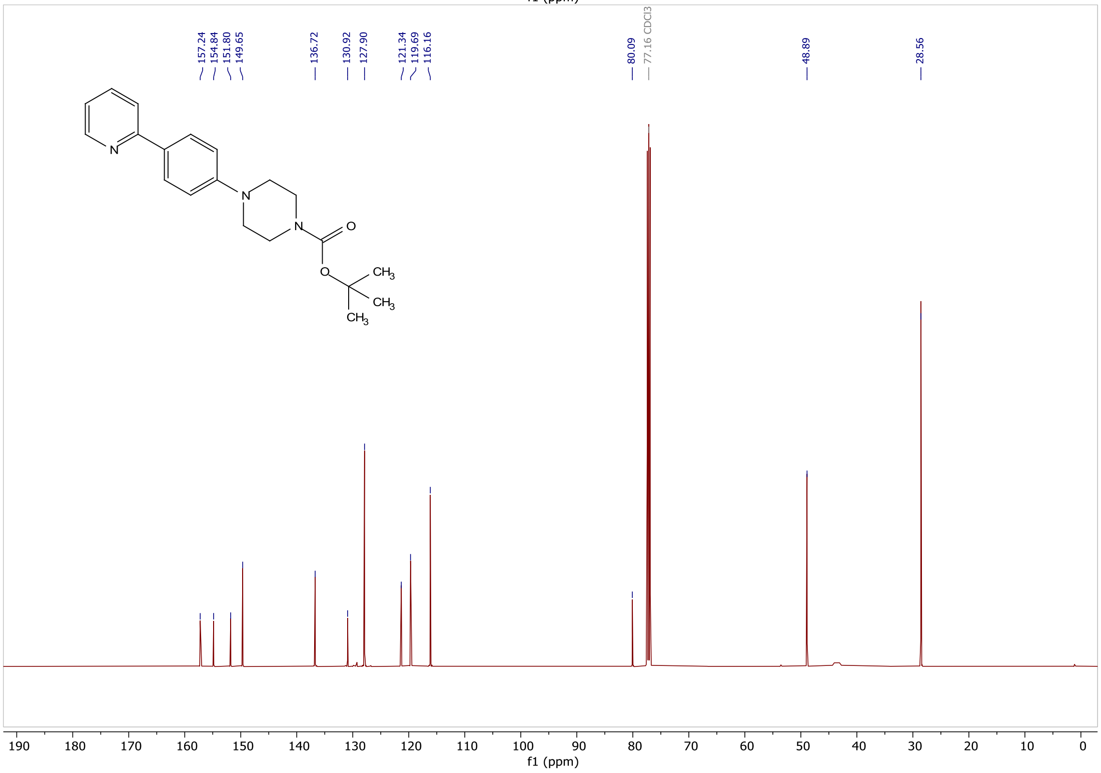

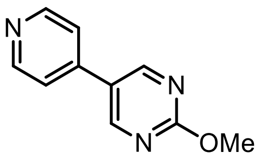

2-methoxy-5-(pyridin-4-yl)pyrimidine (3ao)

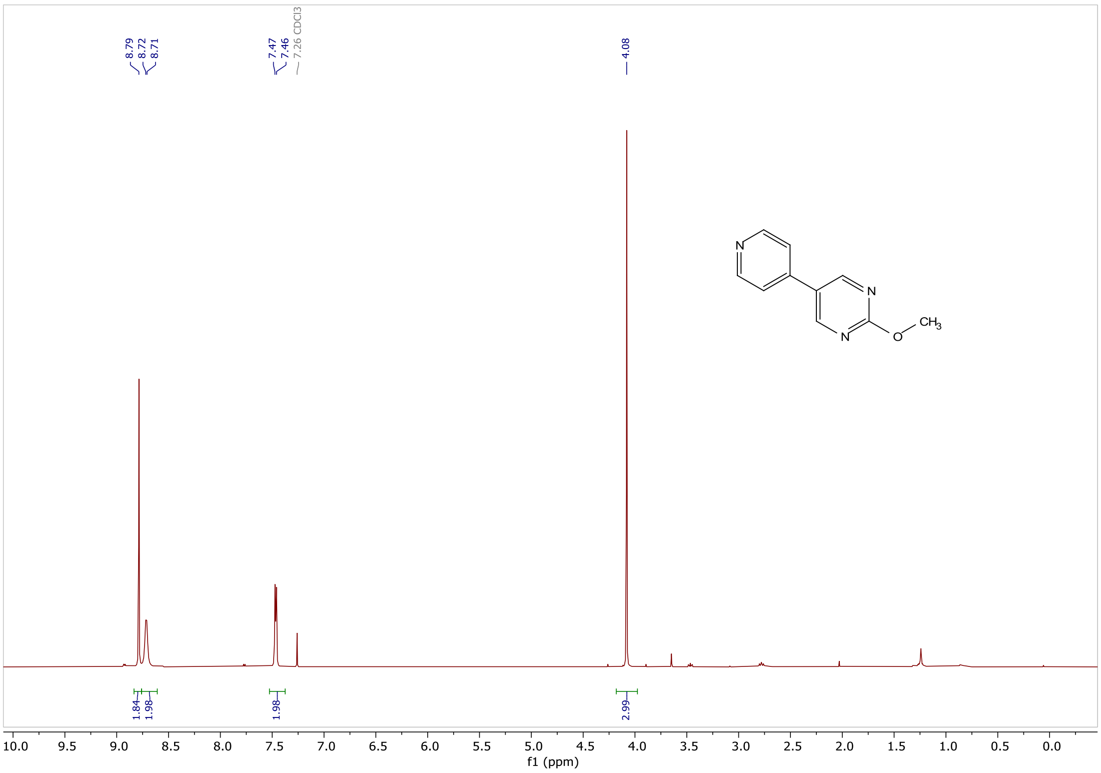

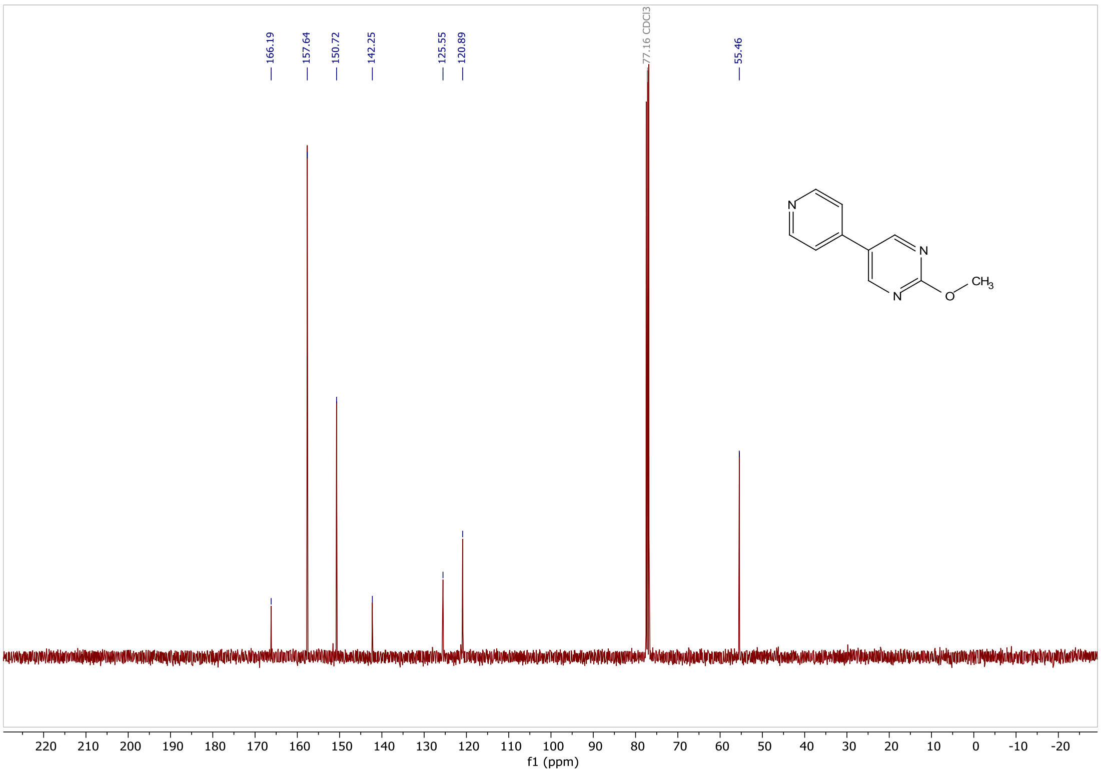

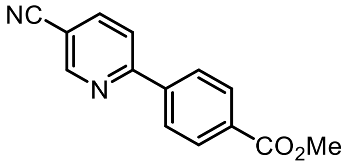

methyl 4-(5-cyanopyridin-2-yl)benzoate (3ap)

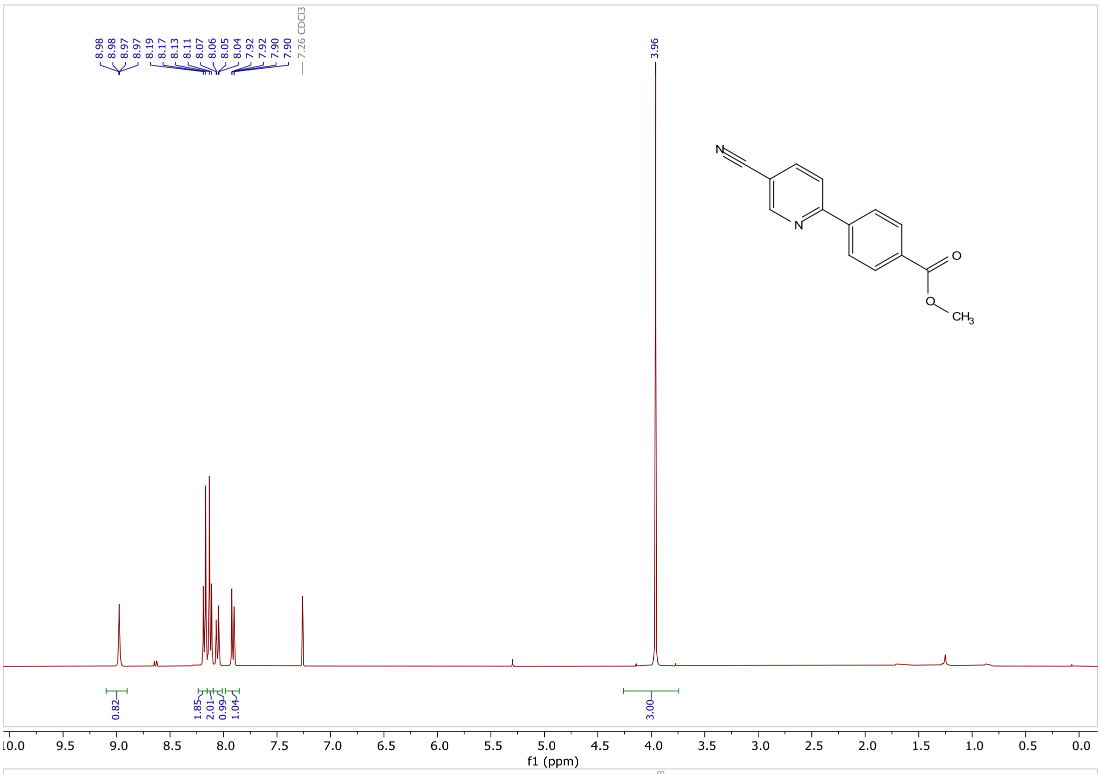

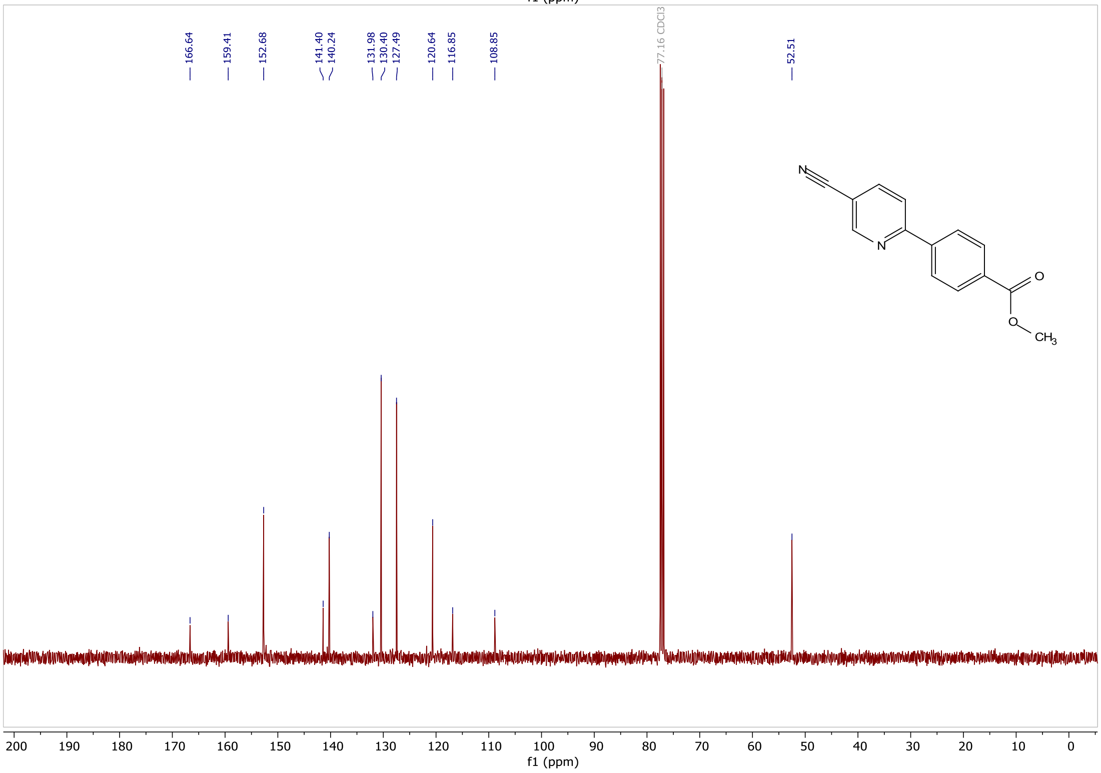

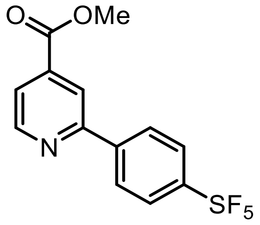

methyl 2-(4-(pentafluoro-l6-sulfaneyl)phenyl)isonicotinate (3aq)

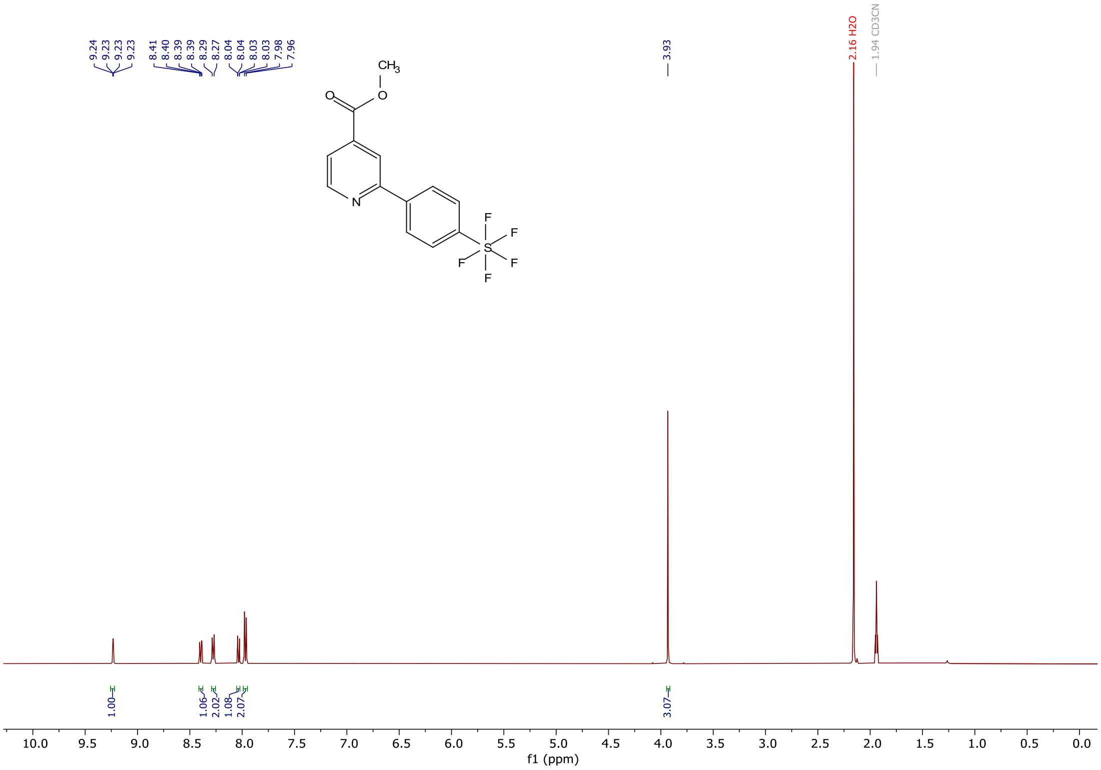

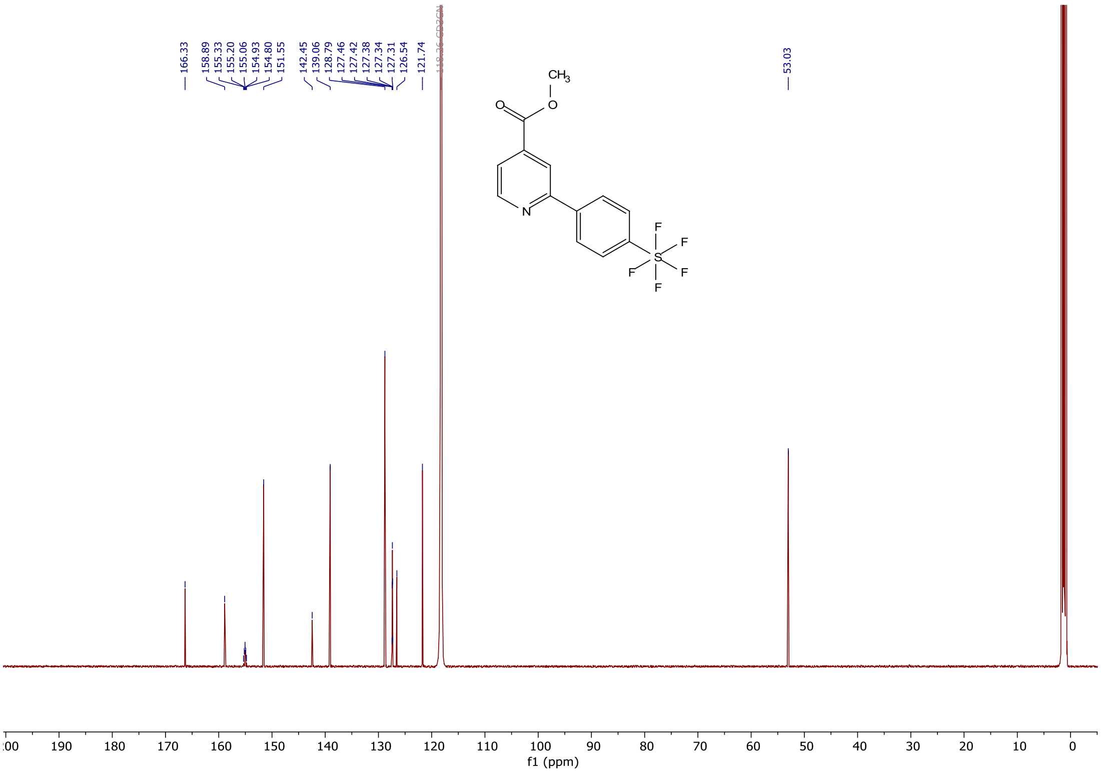

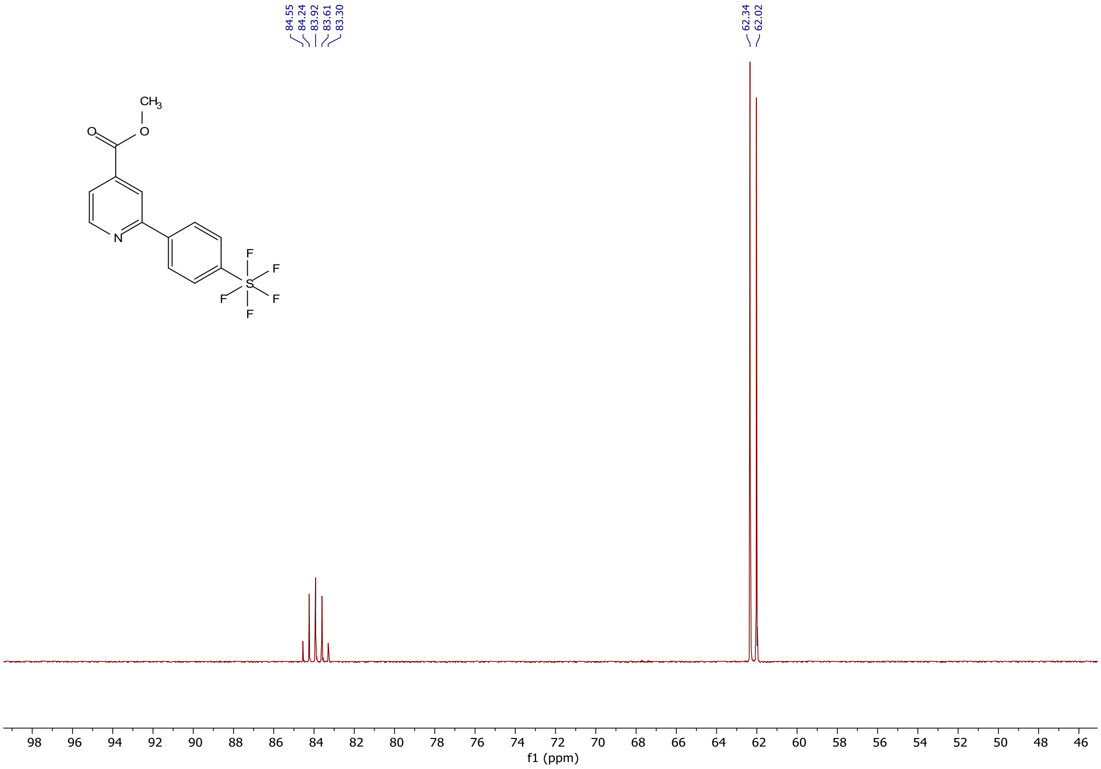
84.55
84.24
83.92
83.61
83.30
62.34
62.02
CH3
O
O
N
F
F
F
F
F
S
98 96 94 92 90 88 86 84 82 80 78 76 74 72 70 68 66 64 62 60 58 56 54 52 50 48 46
f1 (ppm)

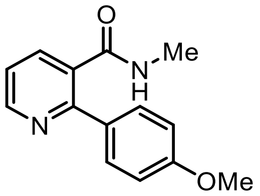

2-(4-methoxyphenyl)-N-methylnicotinamide (3ar)

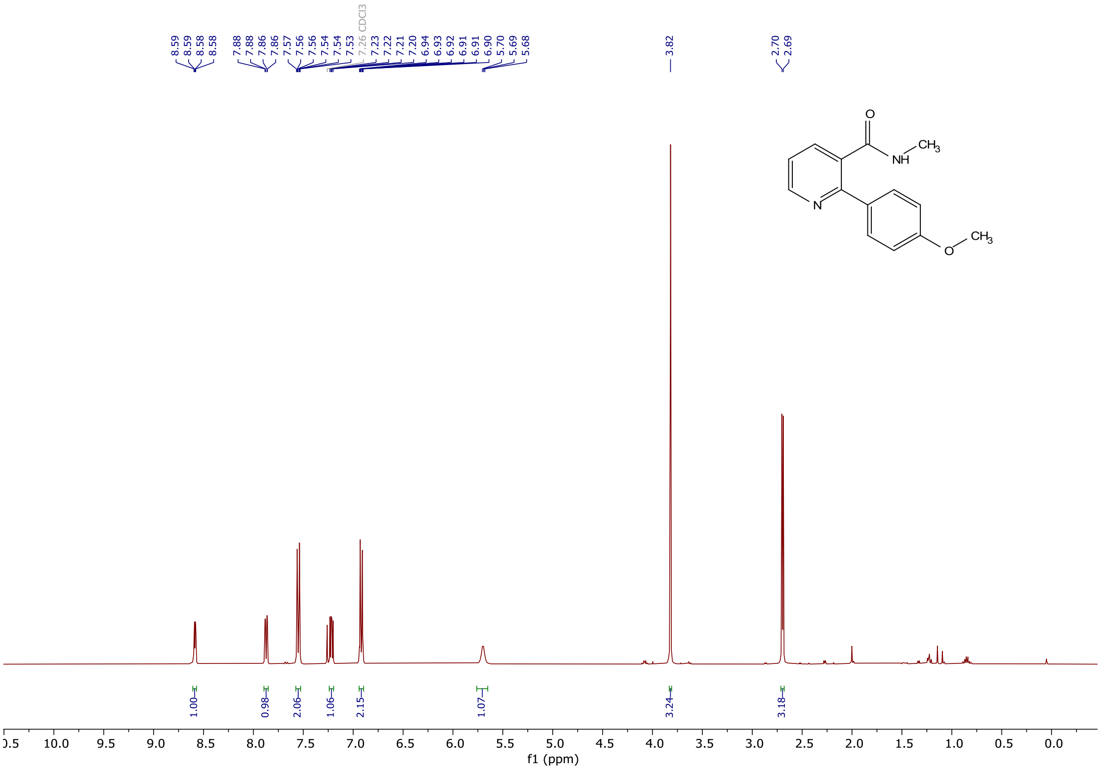

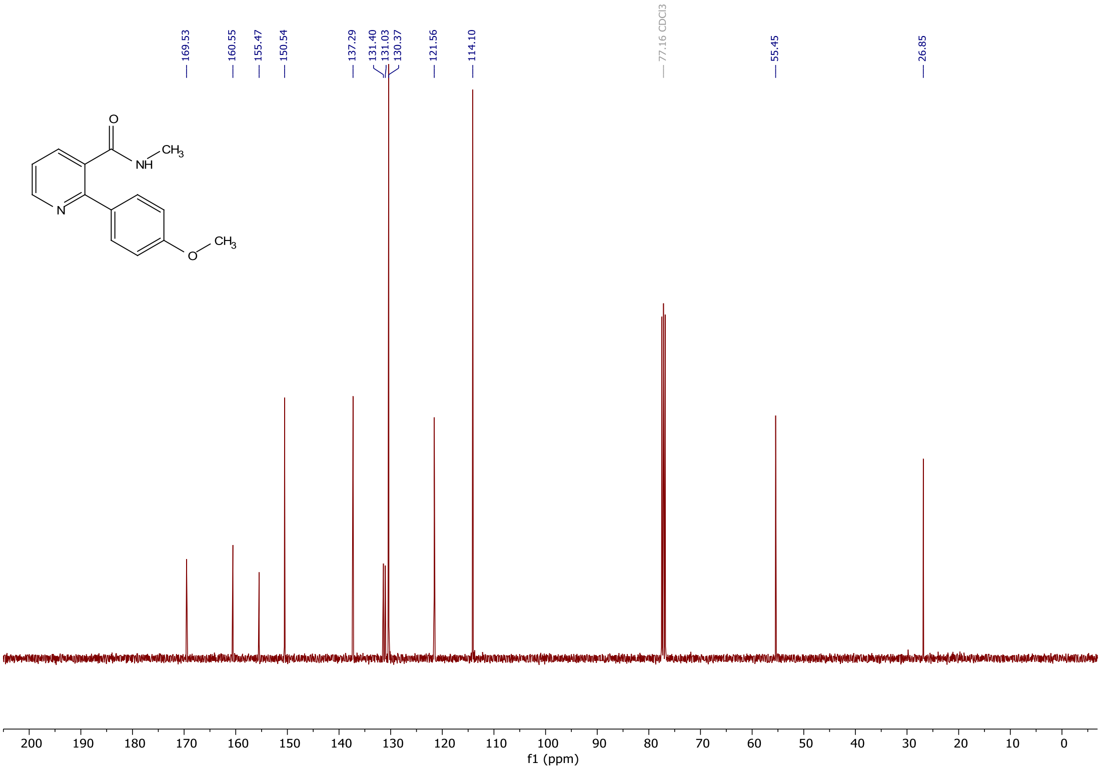

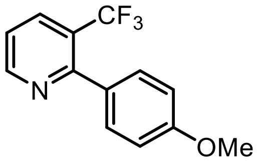

2-(4-methoxyphenyl)-3-(trifluoromethyl)pyridine (3as)

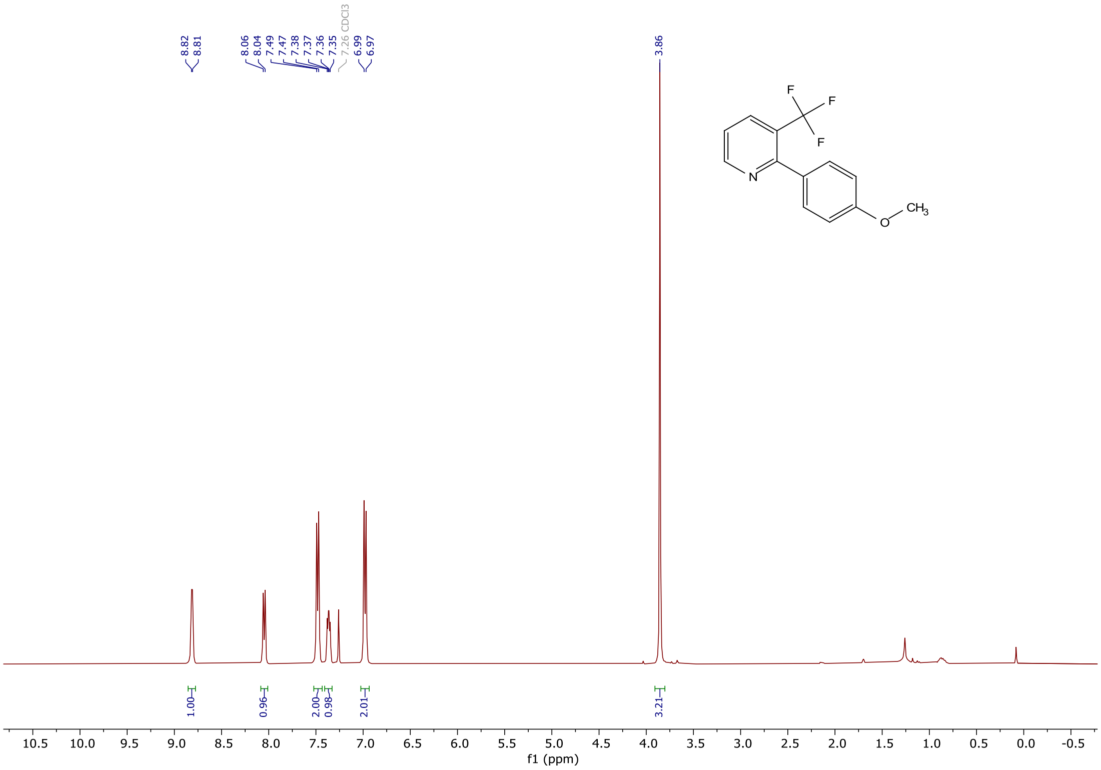

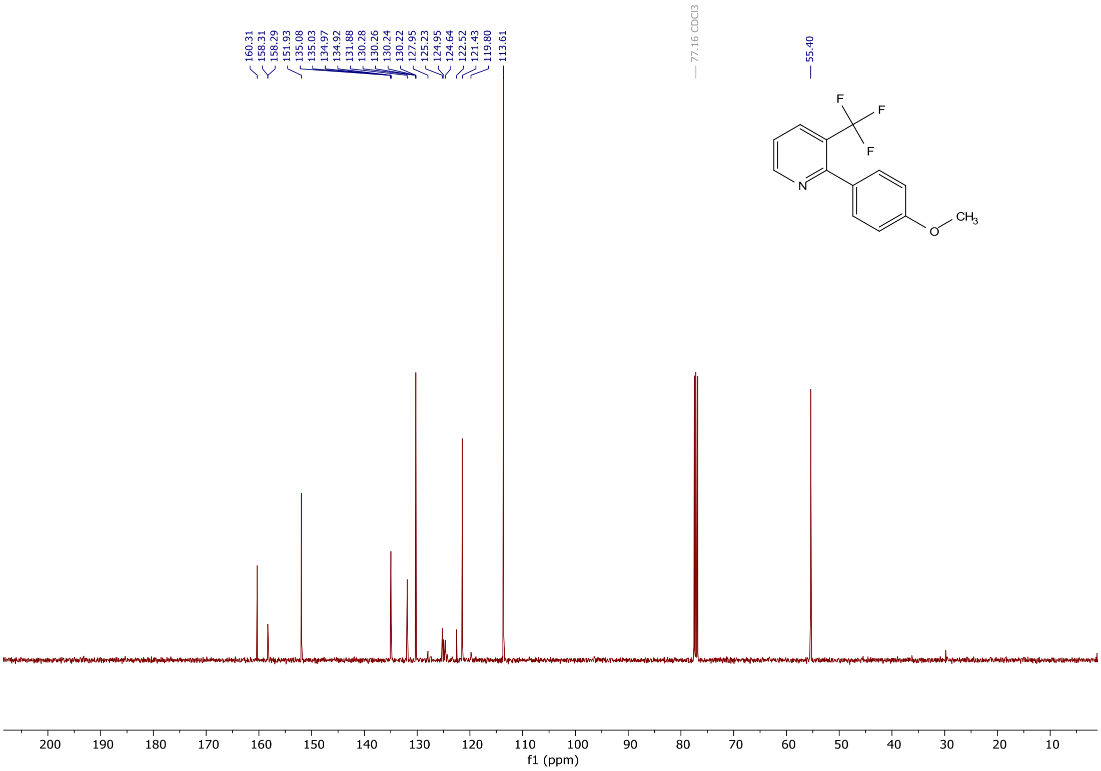

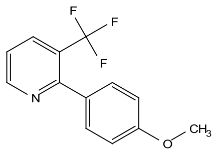

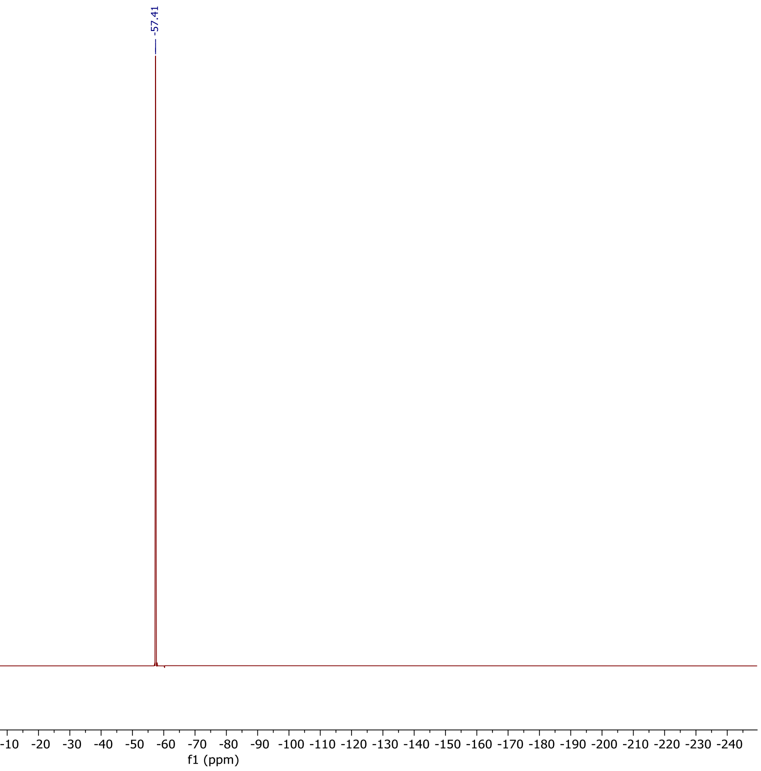

-57.41
90 80 70 60 50 40 30 20 10 0 -10 -20 -30 -40 -50 -60 -70 -80 -90 -100 -110 -120 -130 -140 -150 -160 -170 -180 -190 -200 -210 -220 -230 -240
f1 (ppm)

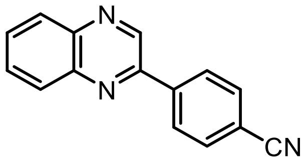

4-(quinoxalin-2-yl)benzonitrile (3at)

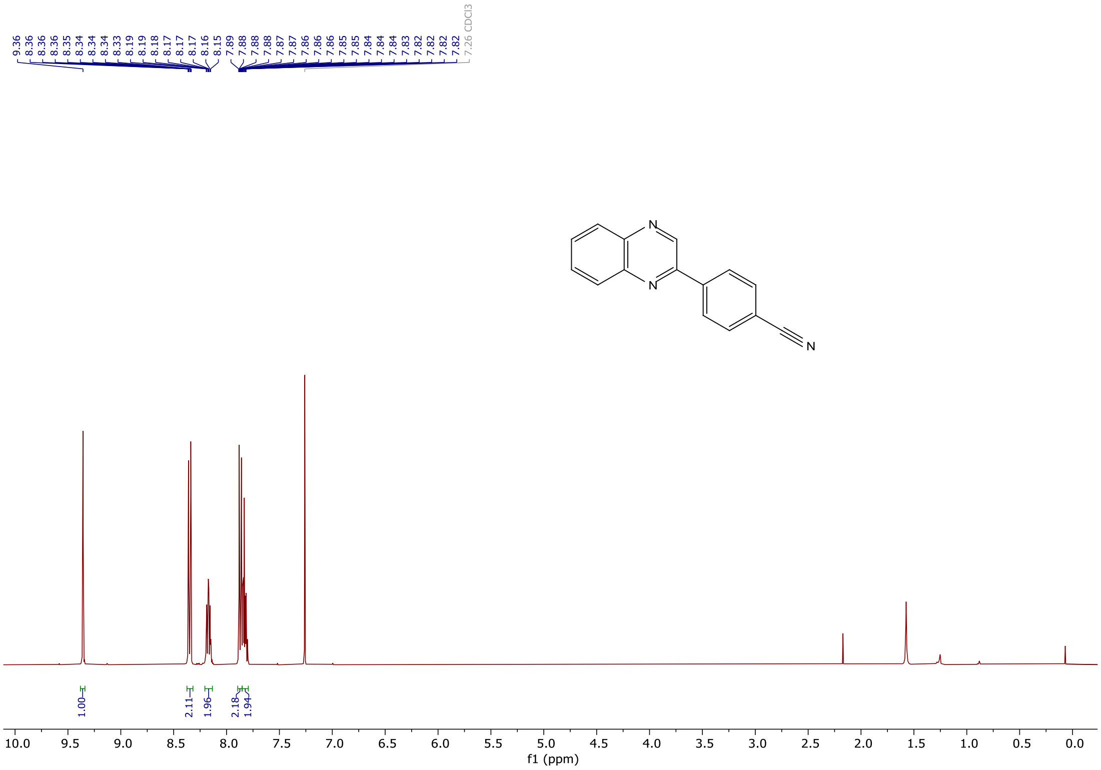

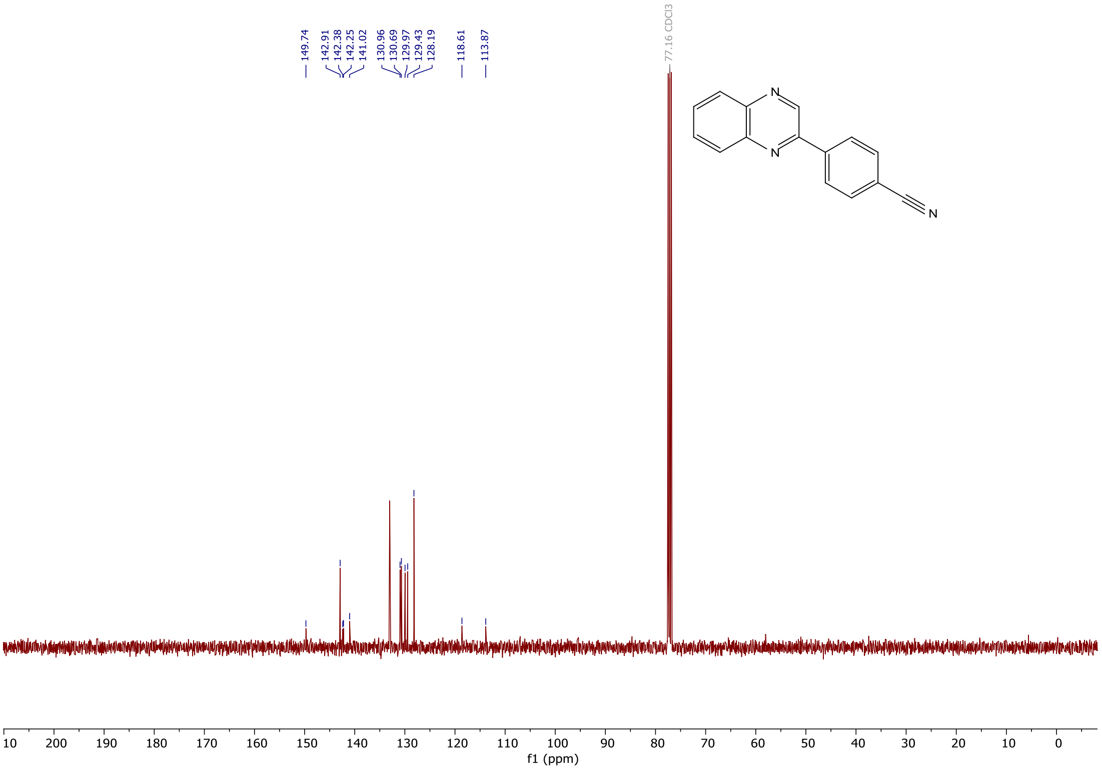

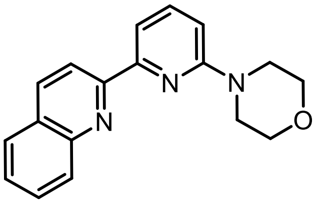

4-(6-(quinolin-2-yl)pyridin-2-yl)morpholine (3au)

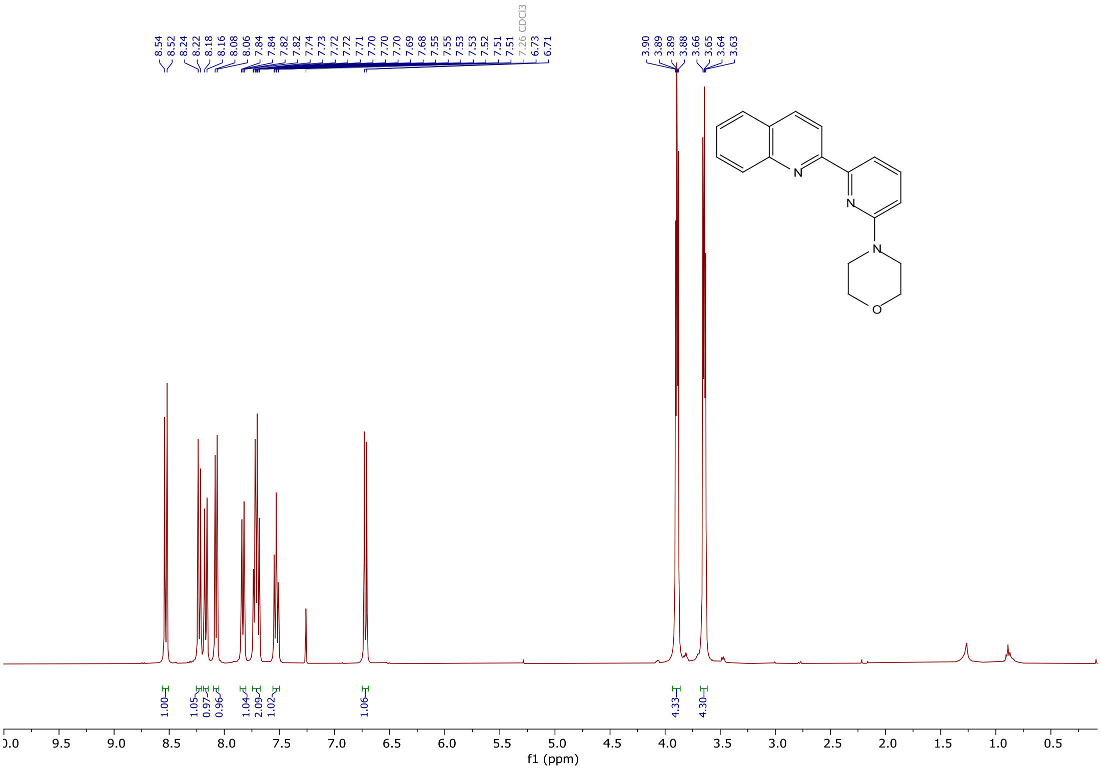

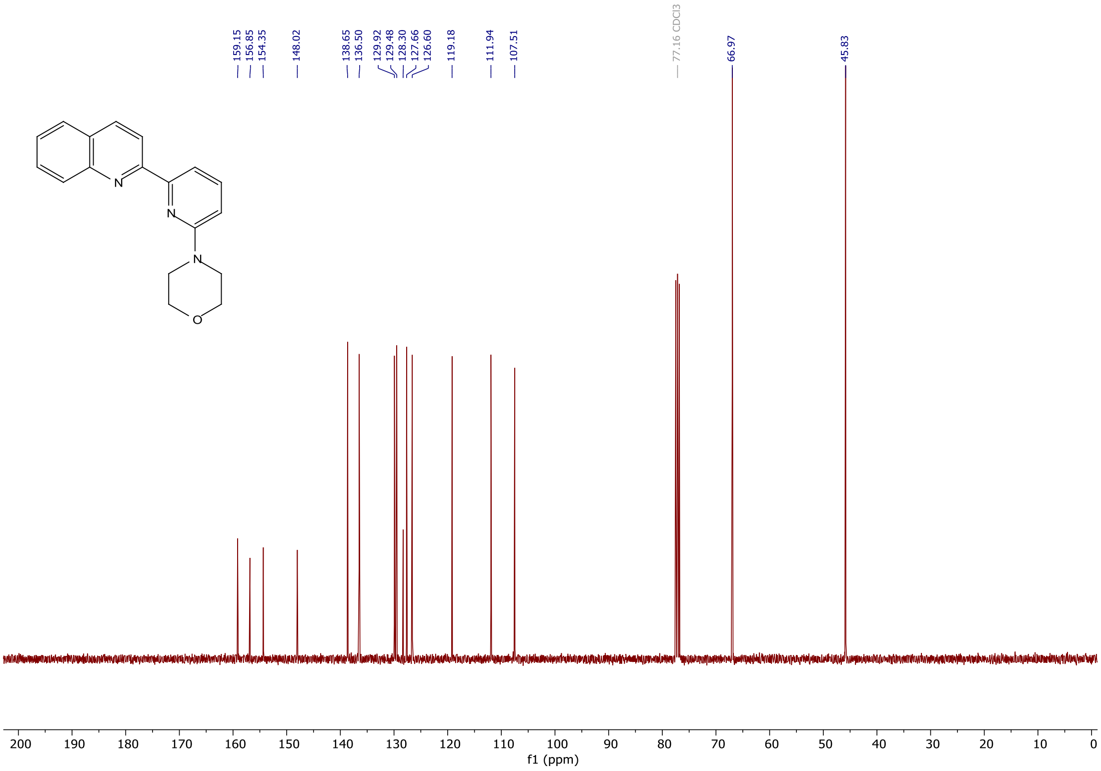

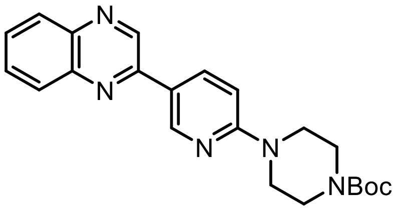

tert-butyl 4-(5-(quinoxalin-2-yl)pyridin-2-yl)piperazine-1-carboxylate (3av)

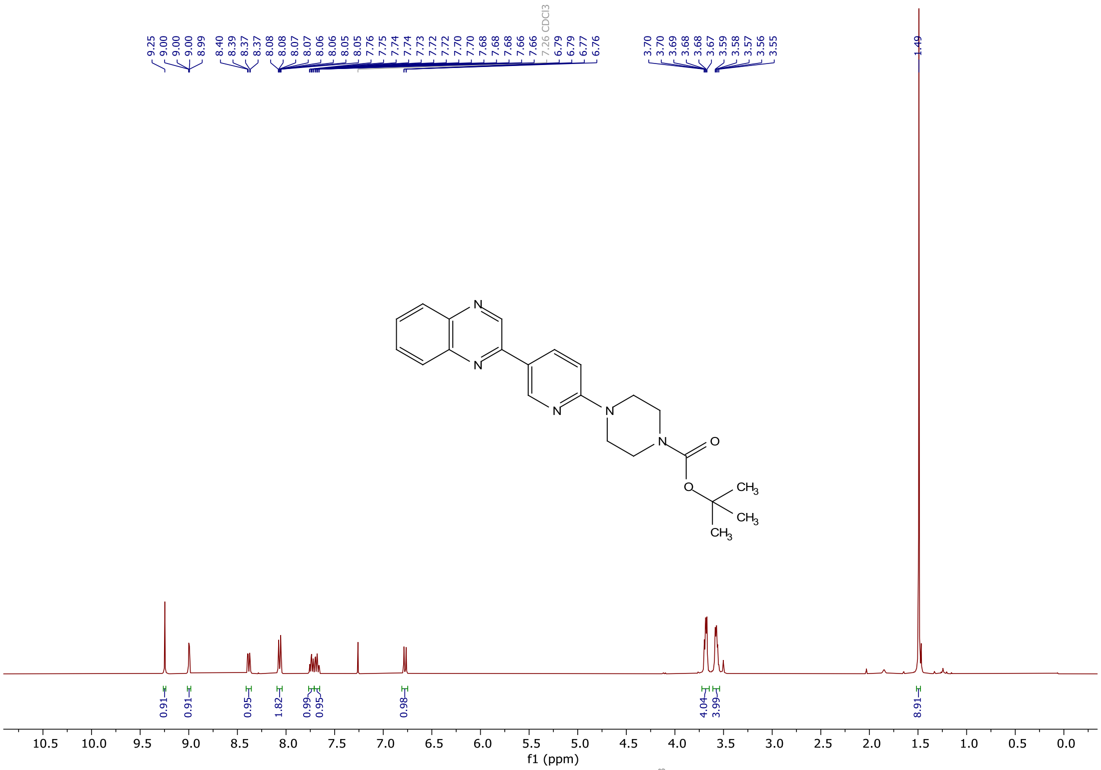

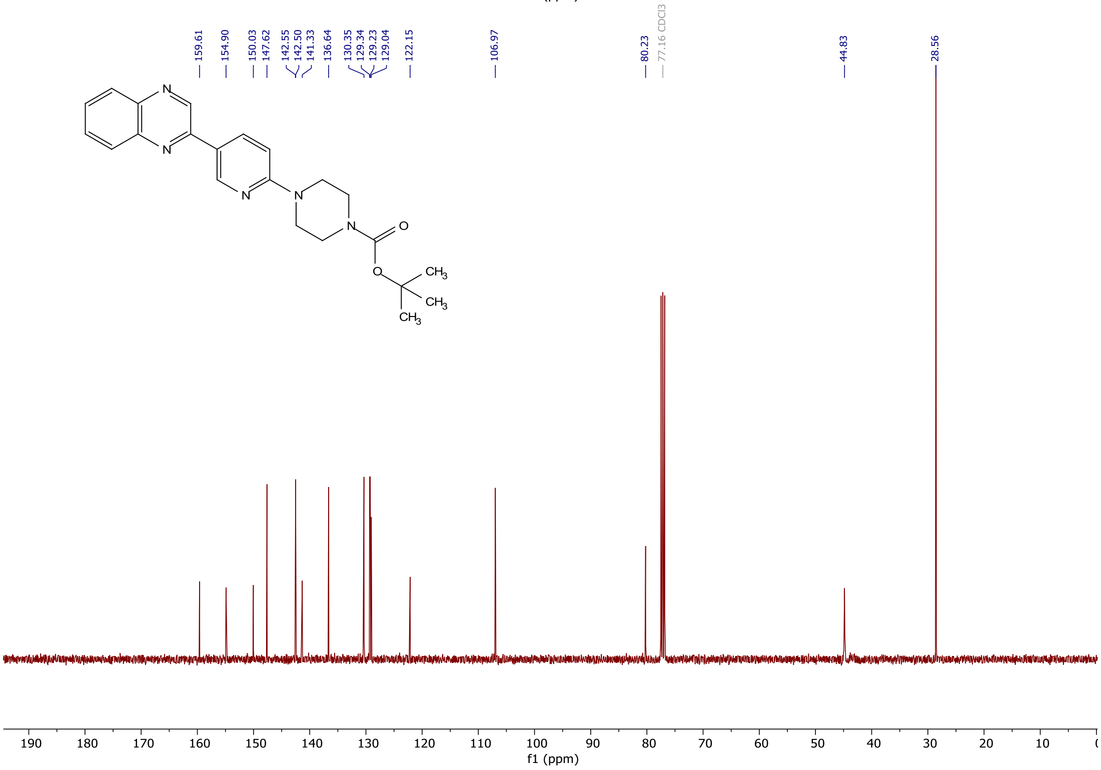

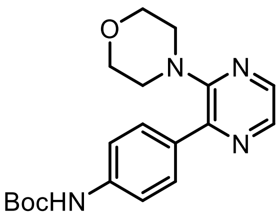

tert-butyl (4-(3-morpholinopyrazin-2-yl)phenyl)carbamate (3aw)

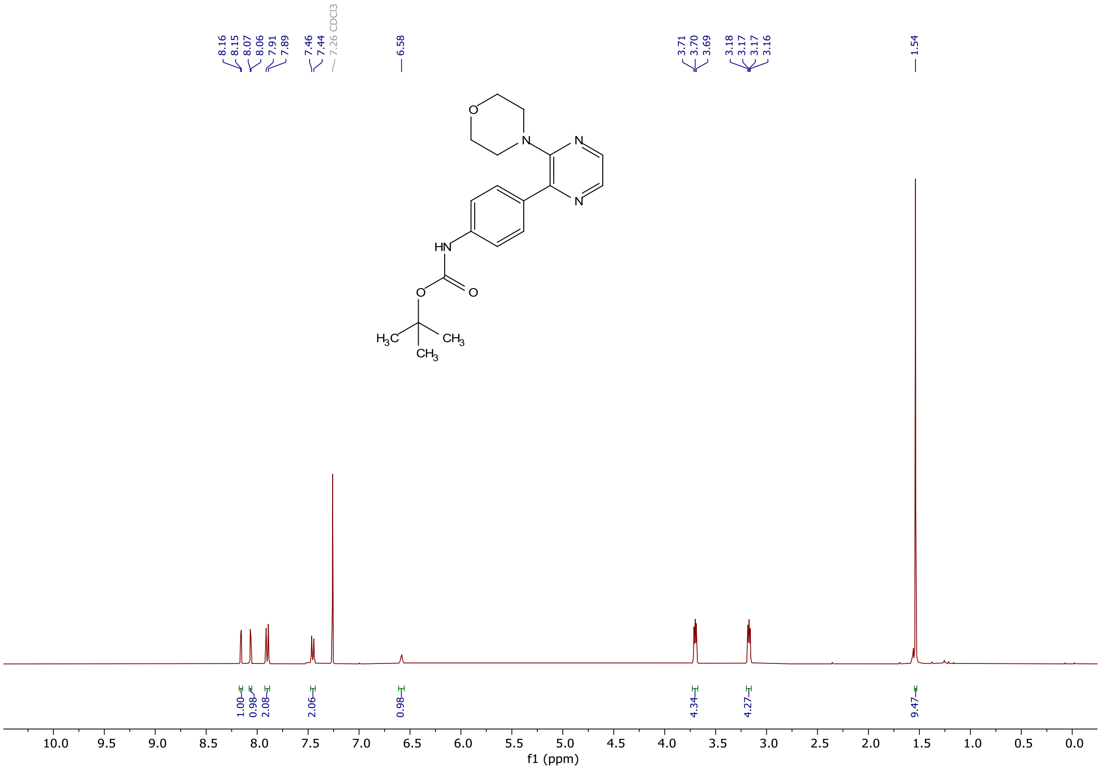

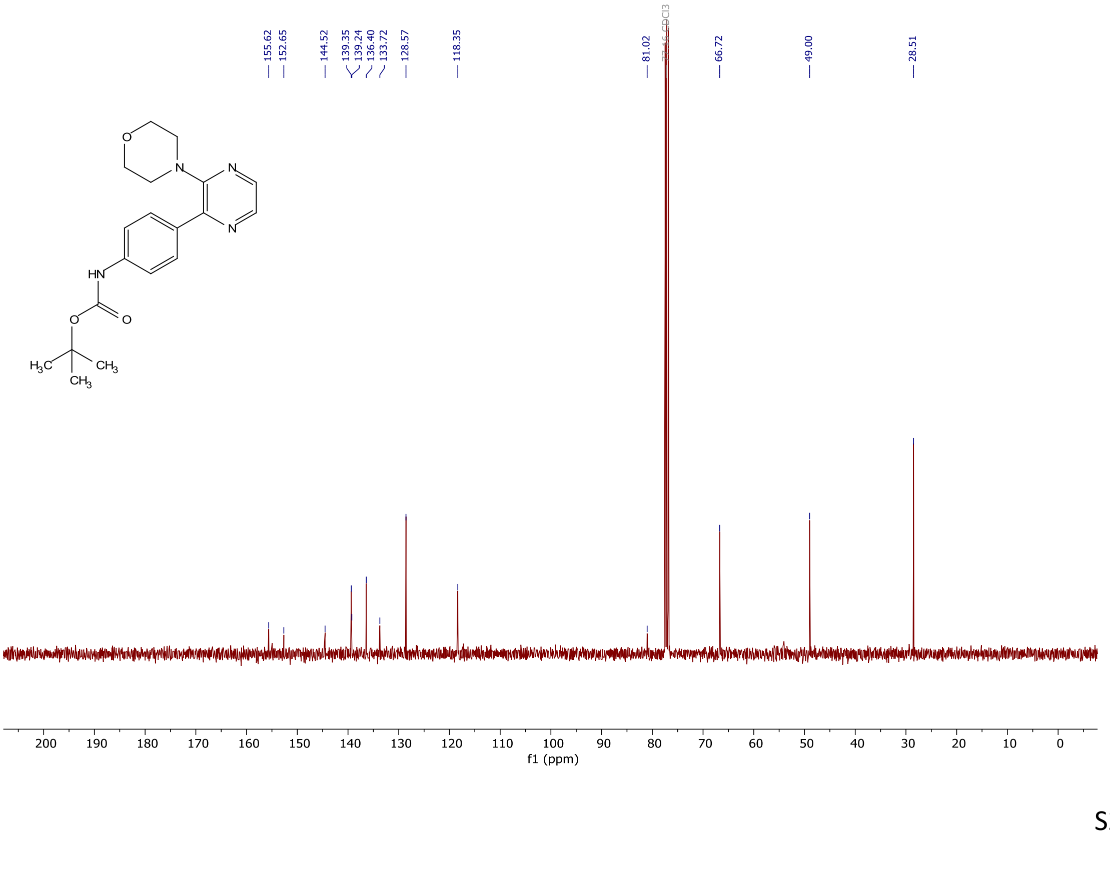

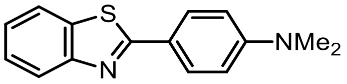

4-(1,3-Benzothiazol-2-yl)-N,N-dimethylaniline (3ax)

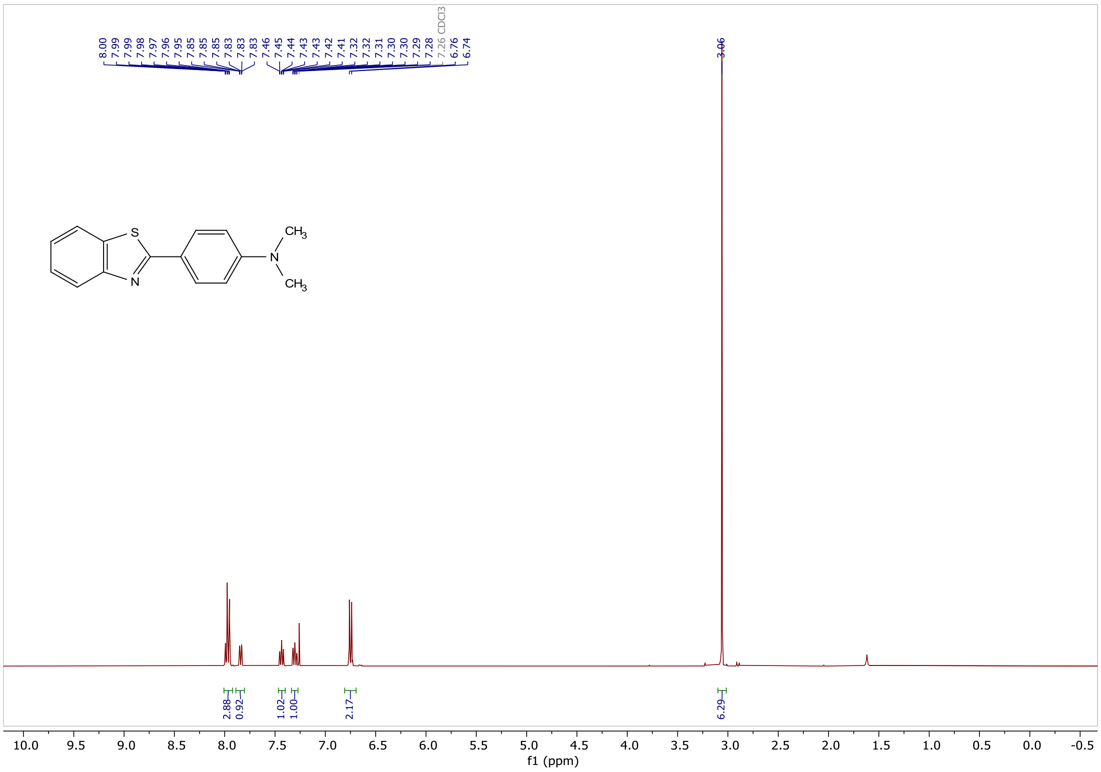

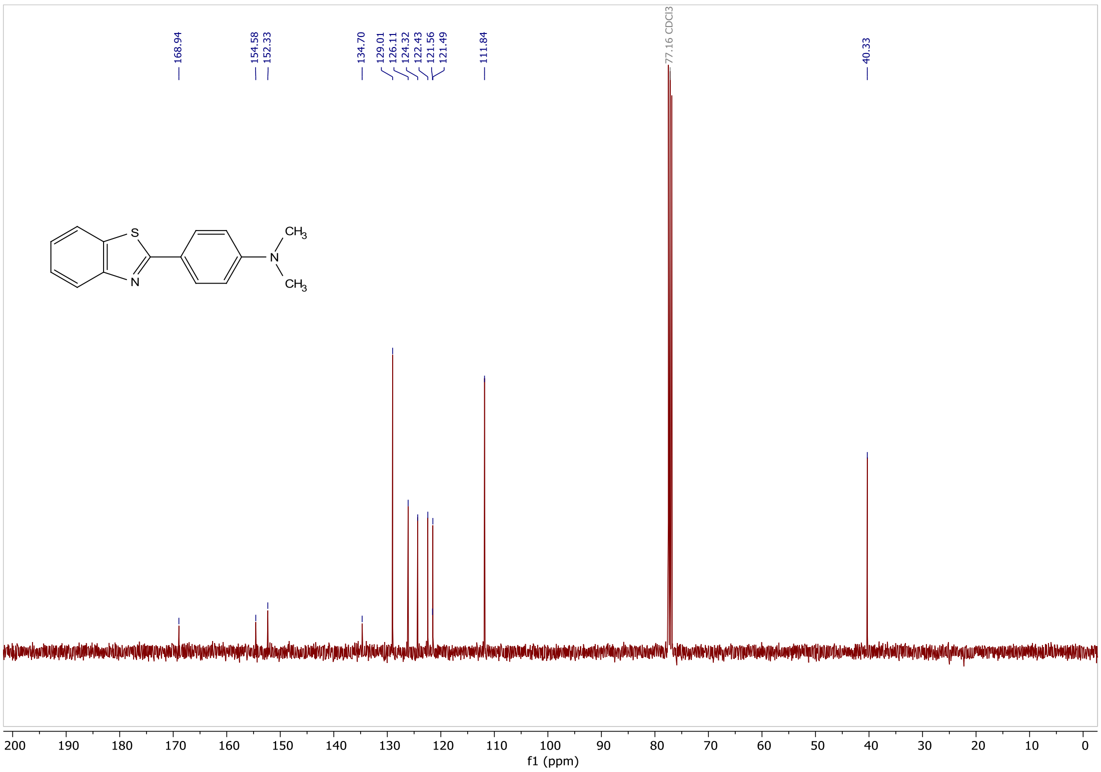

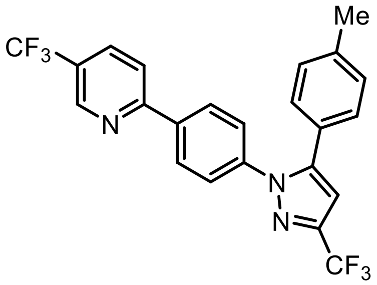

2-(4-(5-(p-tolyl)-3-(trifluoromethyl)-1H-pyrazol-1-yl)phenyl)-5-(trifluoromethyl)pyridine (3ay)

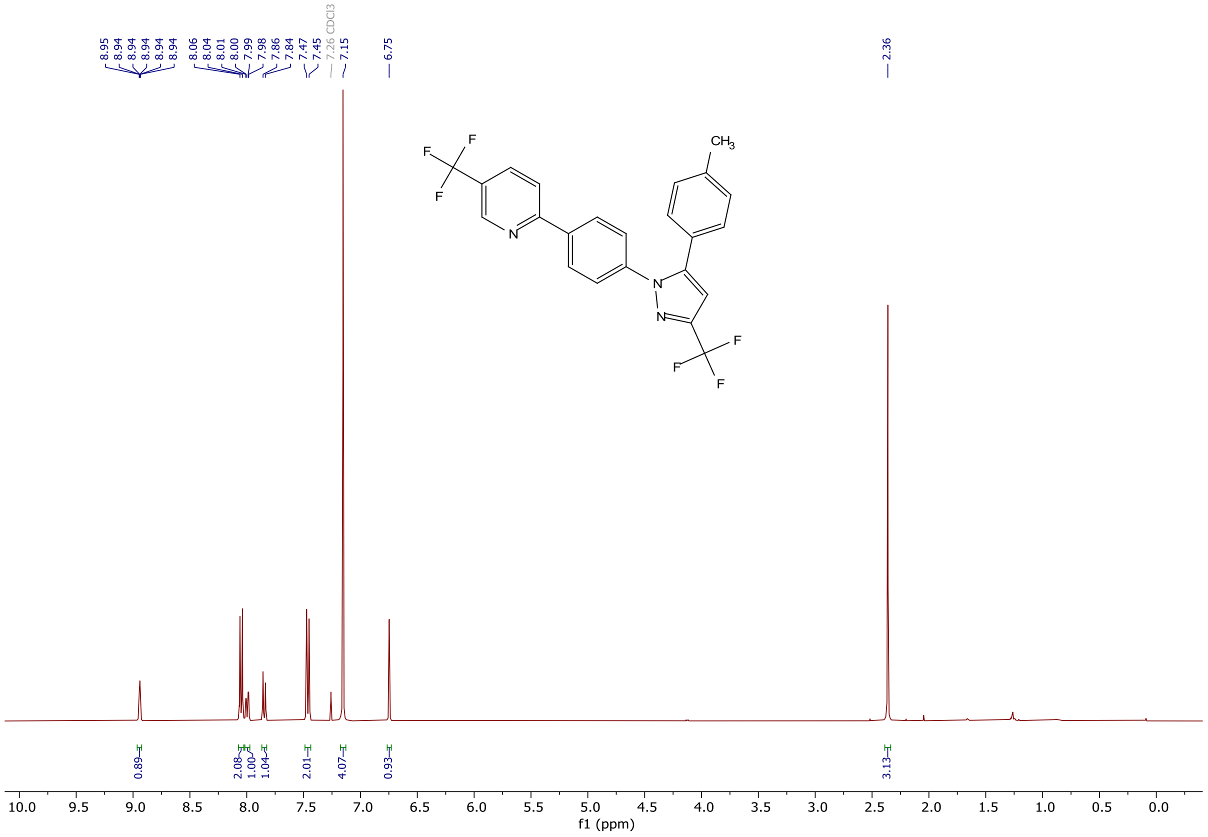

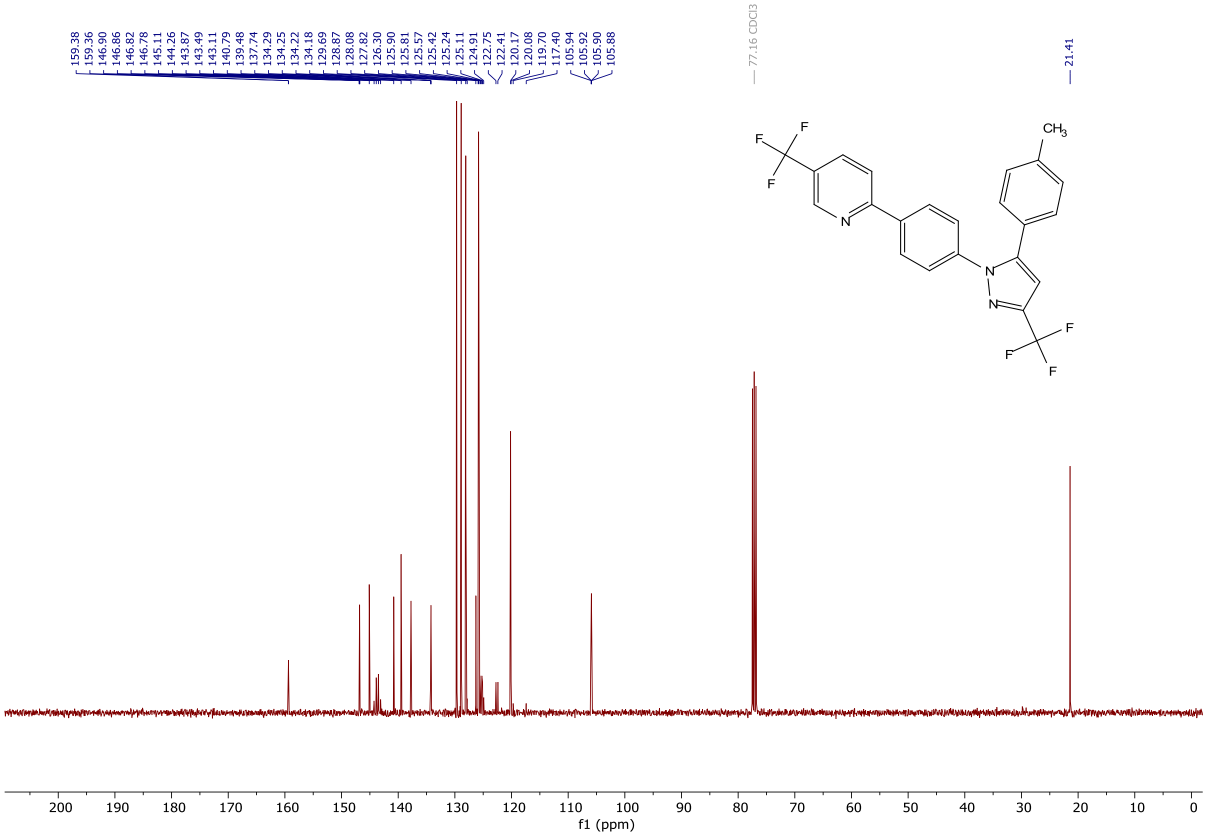
159.38 159.36 146.90 146.86 146.82 146.78 145.11 144.26 143.87 143.49 143.11 140.79 139.48 137.74 134.29 134.25 134.22 134.18 129.69 128.87 128.08 127.82 126.30 125.90 125.81 125.57 125.42 125.24 125.11 124.91 122.75 122.41 120.17 120.08 119.70 117.40 105.94 105.92 105.90 105.88
77.16 CDCl3
21.41
f1 (ppm)

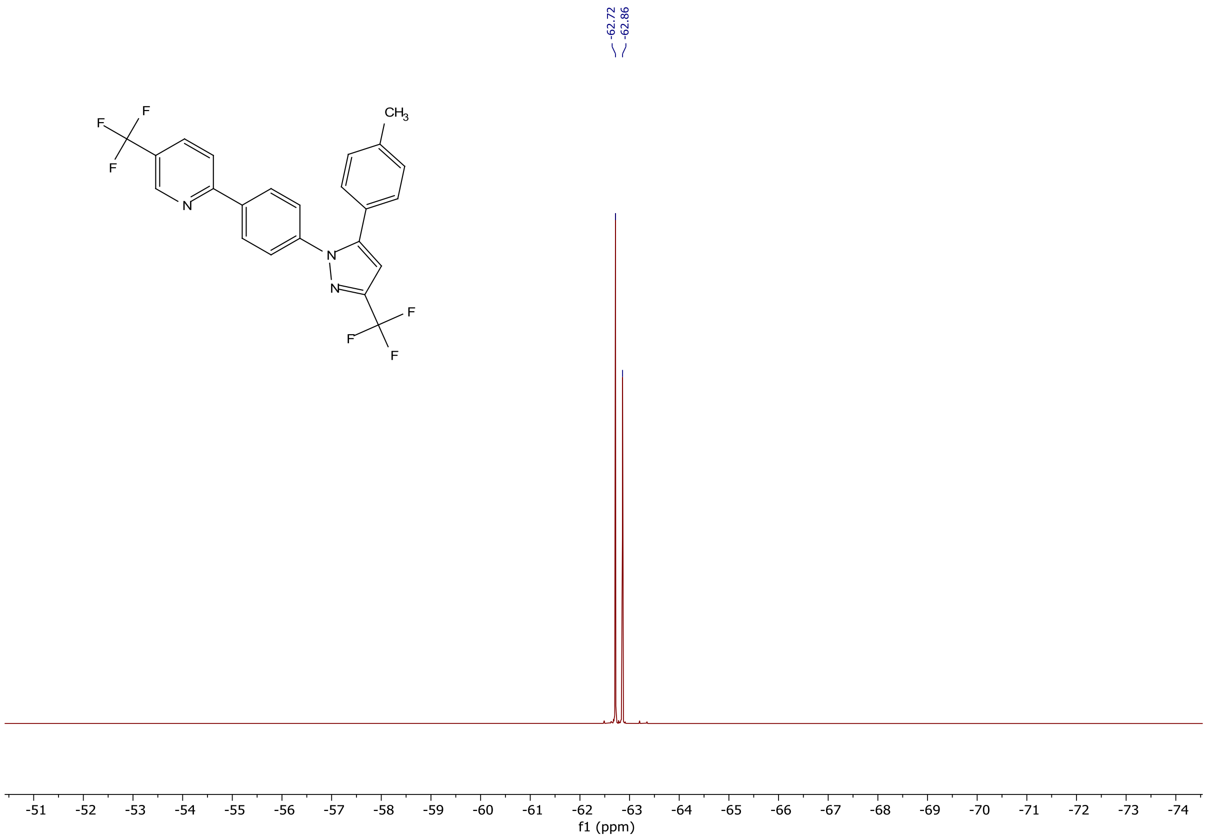
-62.72
-62.86
f1 (ppm)

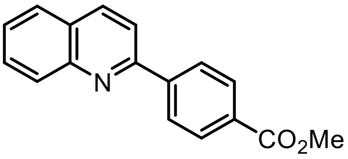

methyl 4-(quinolin-2-yl)benzoate (3az)

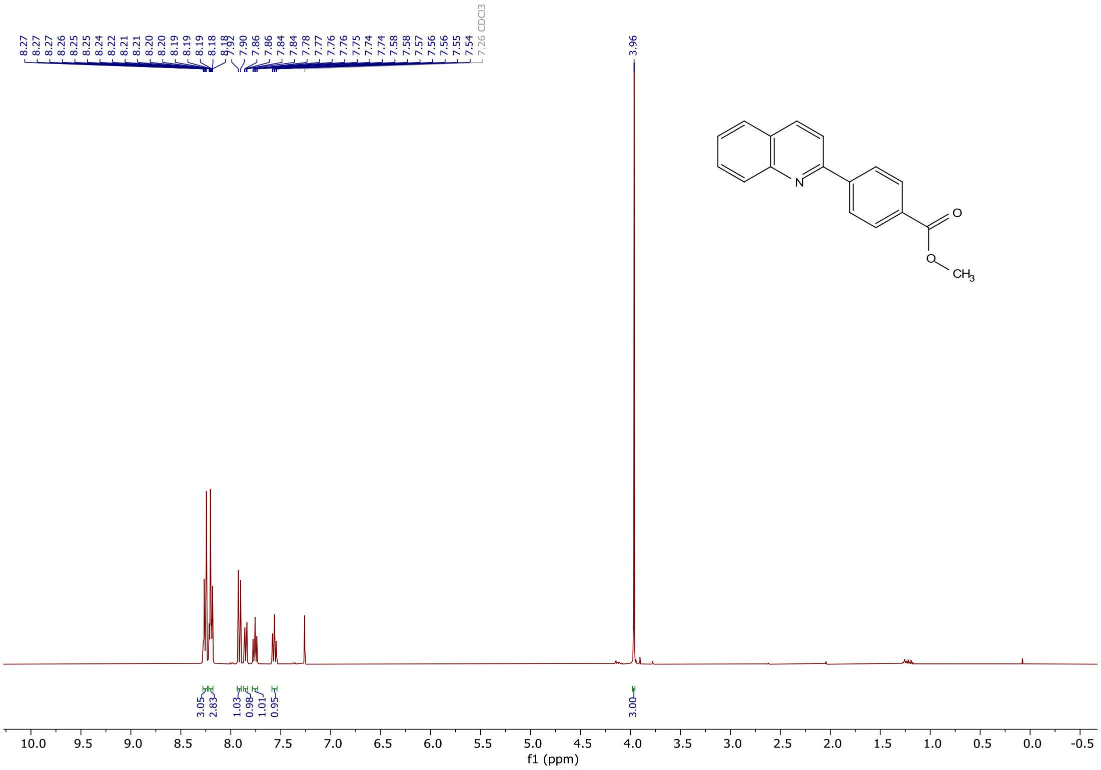

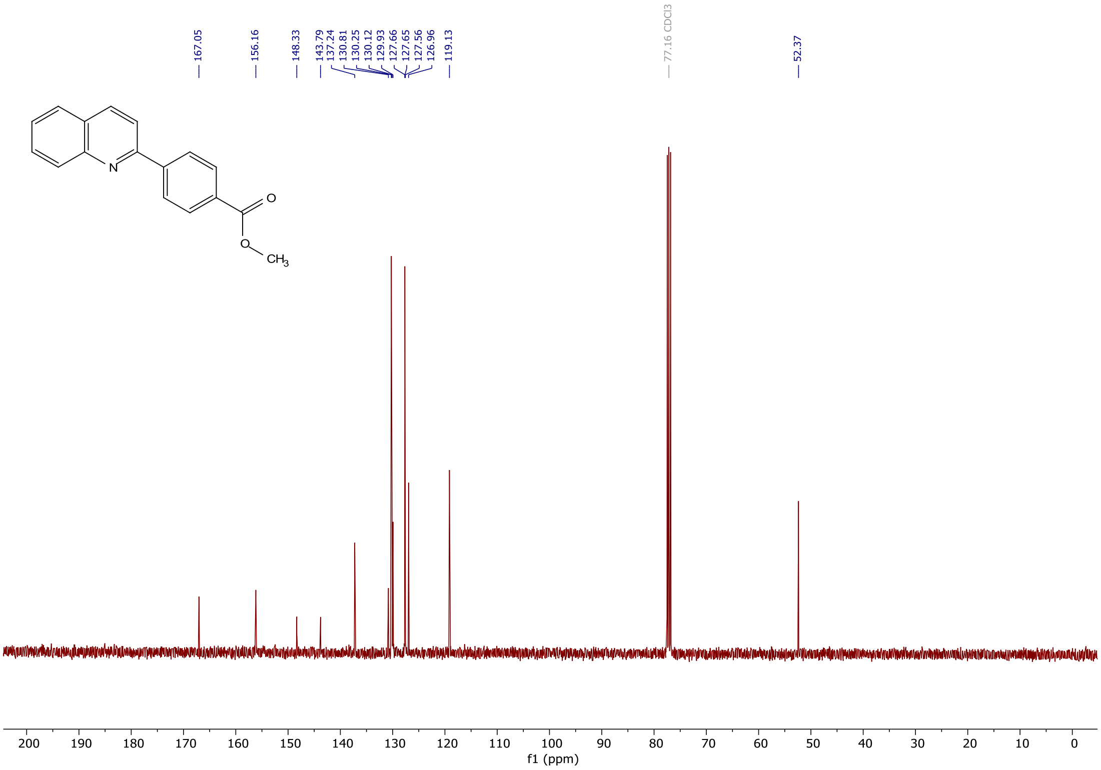

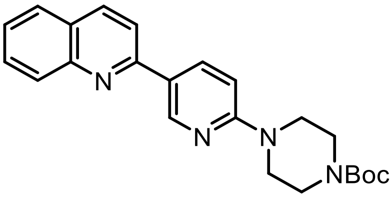

tert-butyl 4-(5-(quinolin-2-yl)pyridin-2-yl)piperazine-1-carboxylate (3ba)

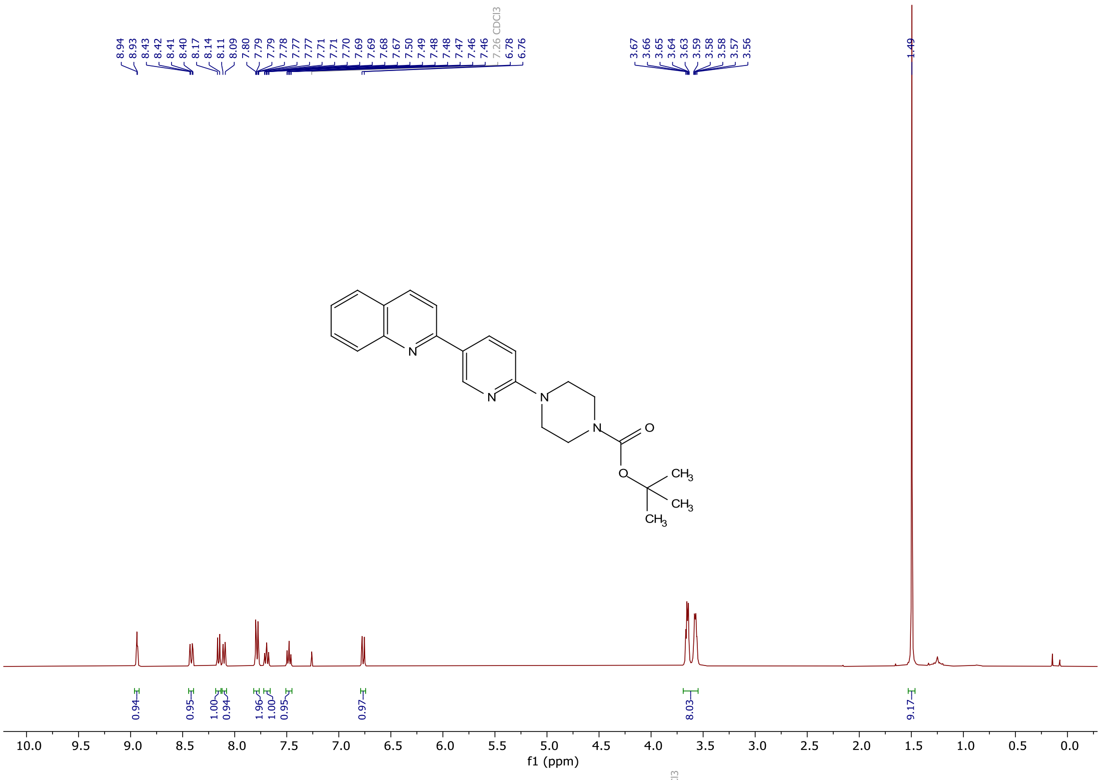

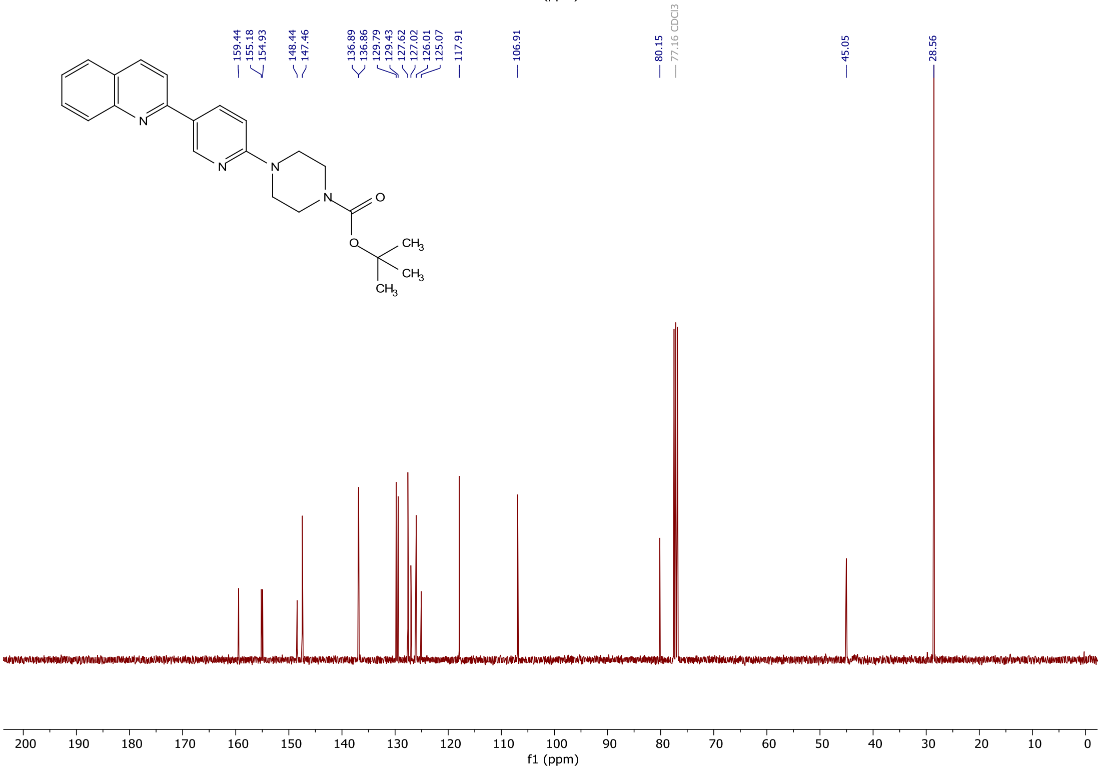

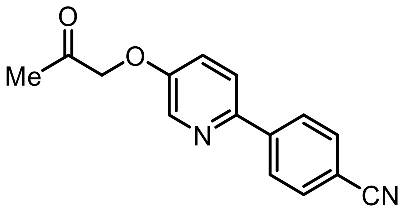

4-(5-(2-oxopropoxy)pyridin-2-yl)benzonitrile (3bb)

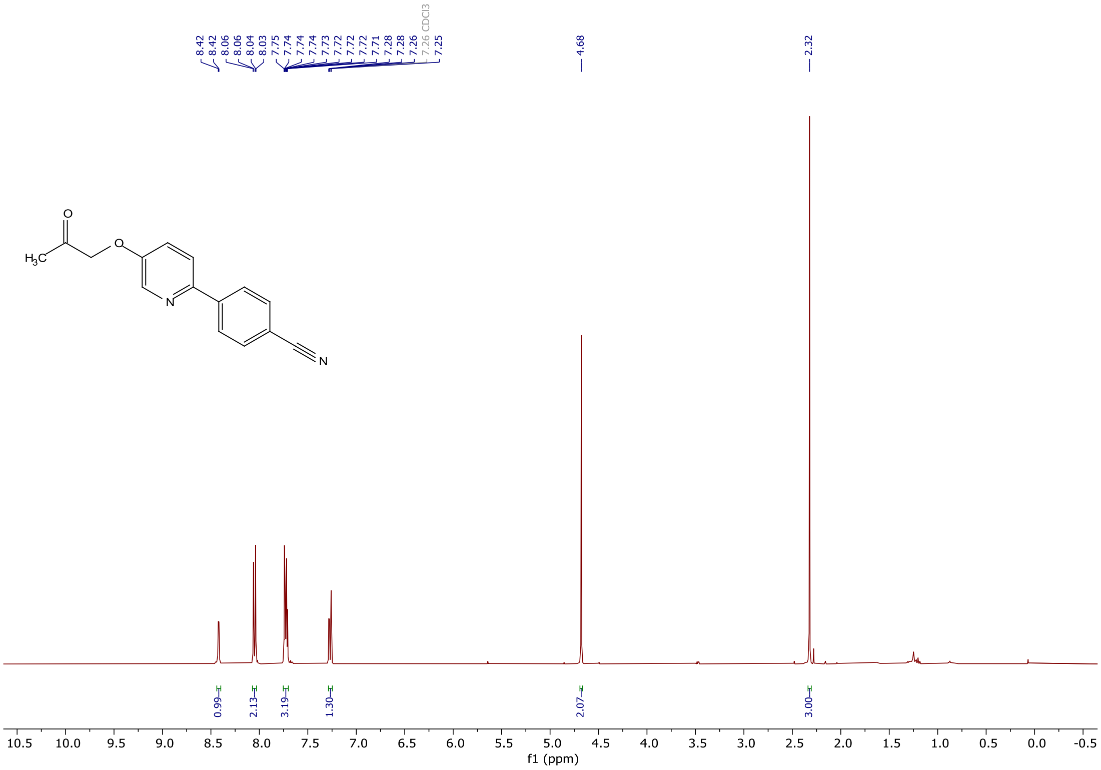
8.42
8.42
8.06
8.06
8.04
8.03
7.75
7.74
7.74
7.73
7.72
7.72
7.72
7.71
7.28
7.28
7.26 CDCl3
7.25
4.68
2.32
H3C
O
O
N
N
0.99
2.13
3.19
1.30
2.07
3.00
10.5 10.0 9.5 9.0 8.5 8.0 7.5 7.0 6.5 6.0 5.5 5.0 4.5 4.0 3.5 3.0 2.5 2.0 1.5 1.0 0.5 0.0 -0.5
f1 (ppm)

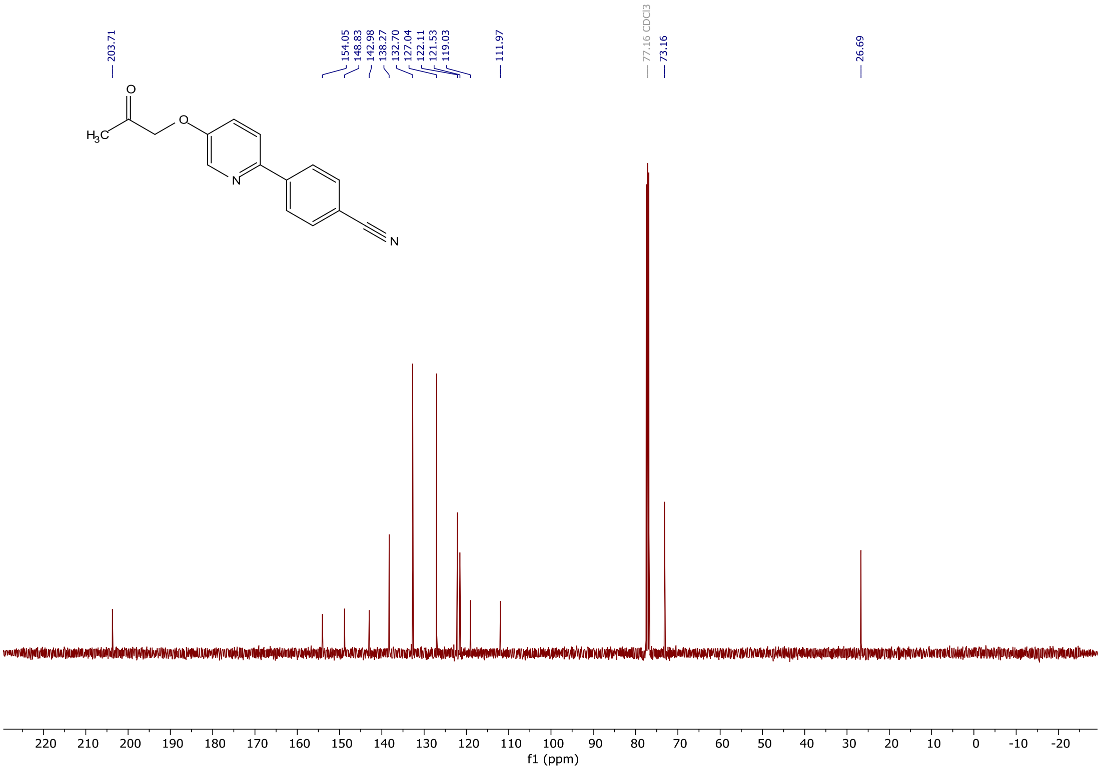
203.71
154.05
148.83
142.98
138.27
132.70
127.04
122.11
121.53
119.03
111.97
77.16 CDCl3
73.16
26.69
H3C
O
O
N
N
220 210 200 190 180 170 160 150 140 130 120 110 100 90 80 70 60 50 40 30 20 10 0 -10 -20
f1 (ppm)

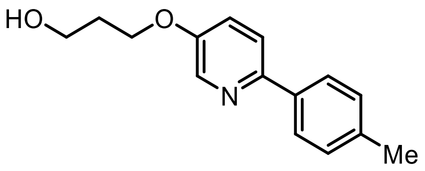
HO
O
N
Me

3-((6-(p-tolyl)pyridin-3-yl)oxy)propan-1-ol (3bc)

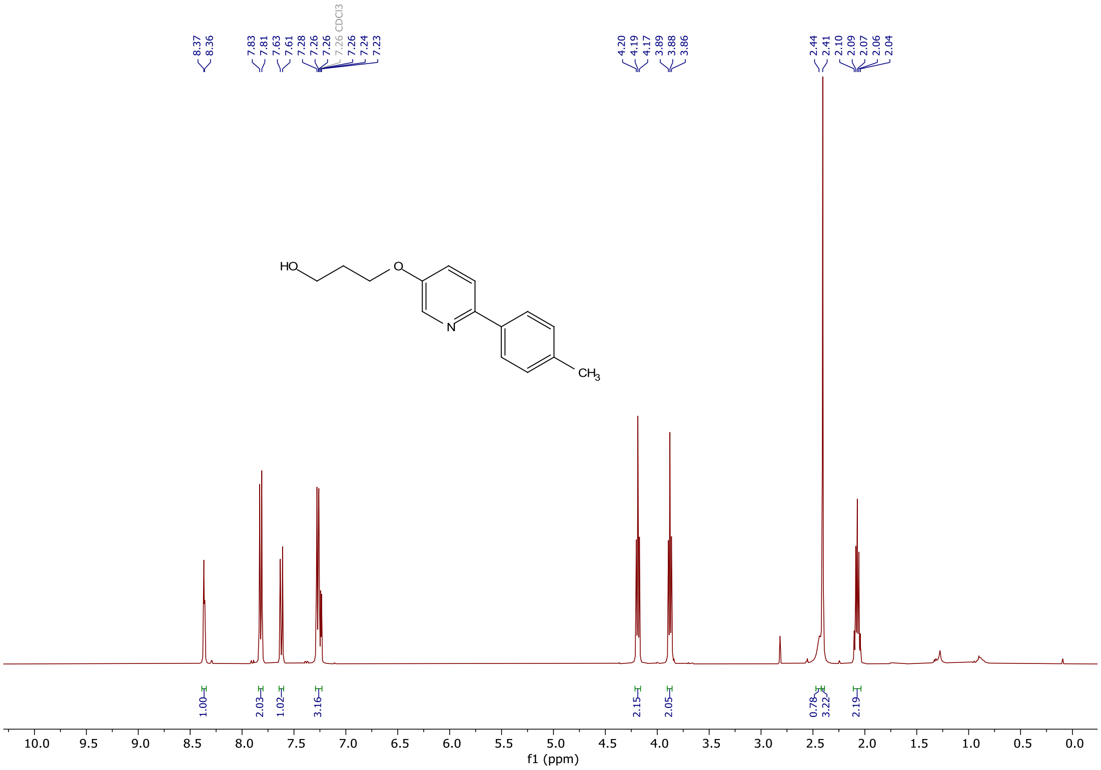

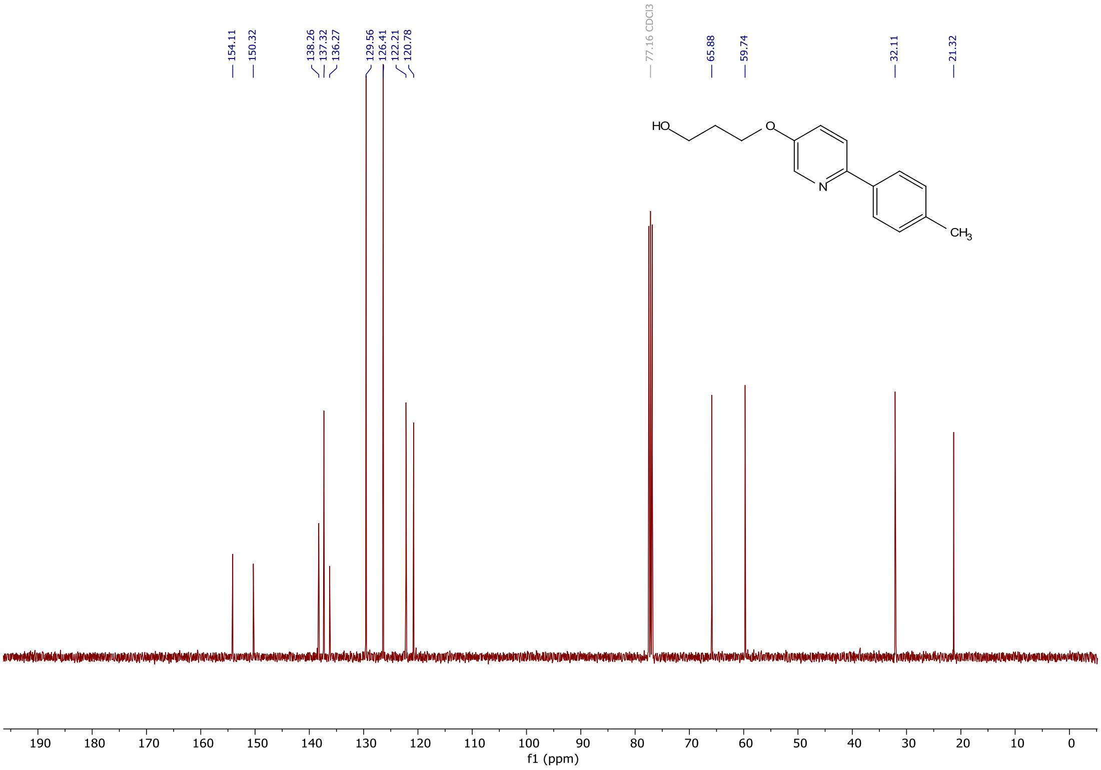

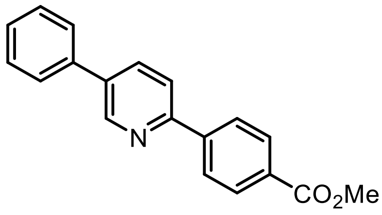

methyl 4-(5-phenylpyridin-2-yl)benzoate (11)

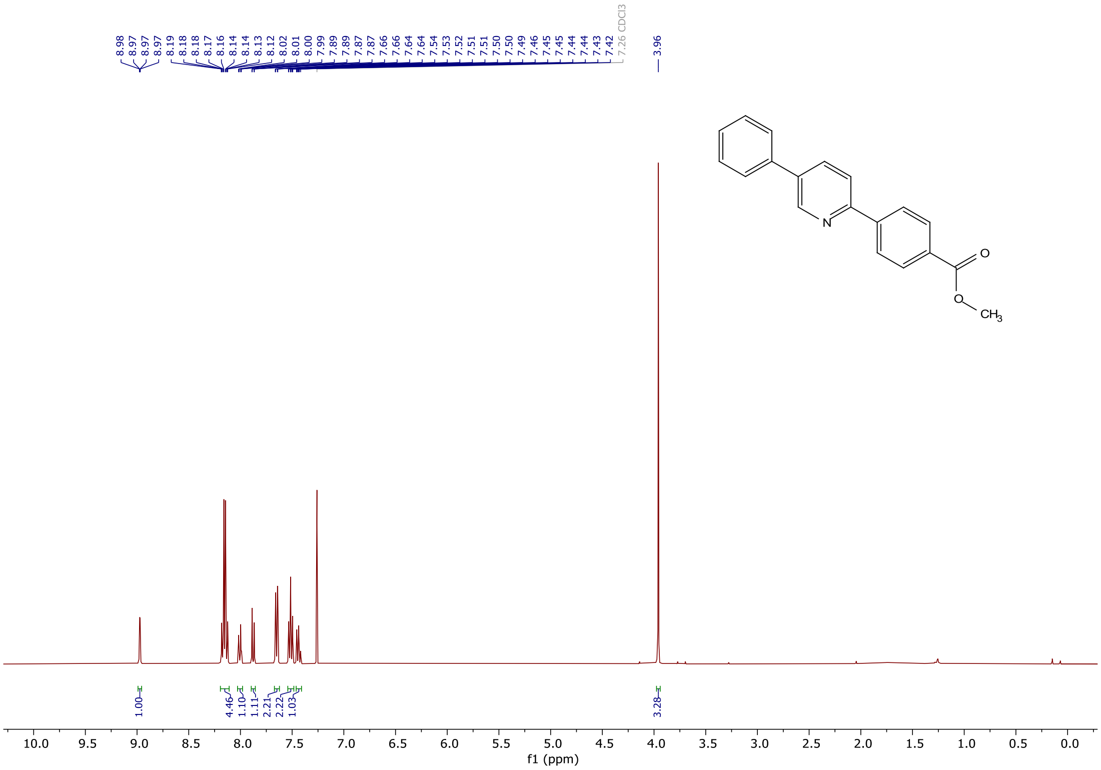

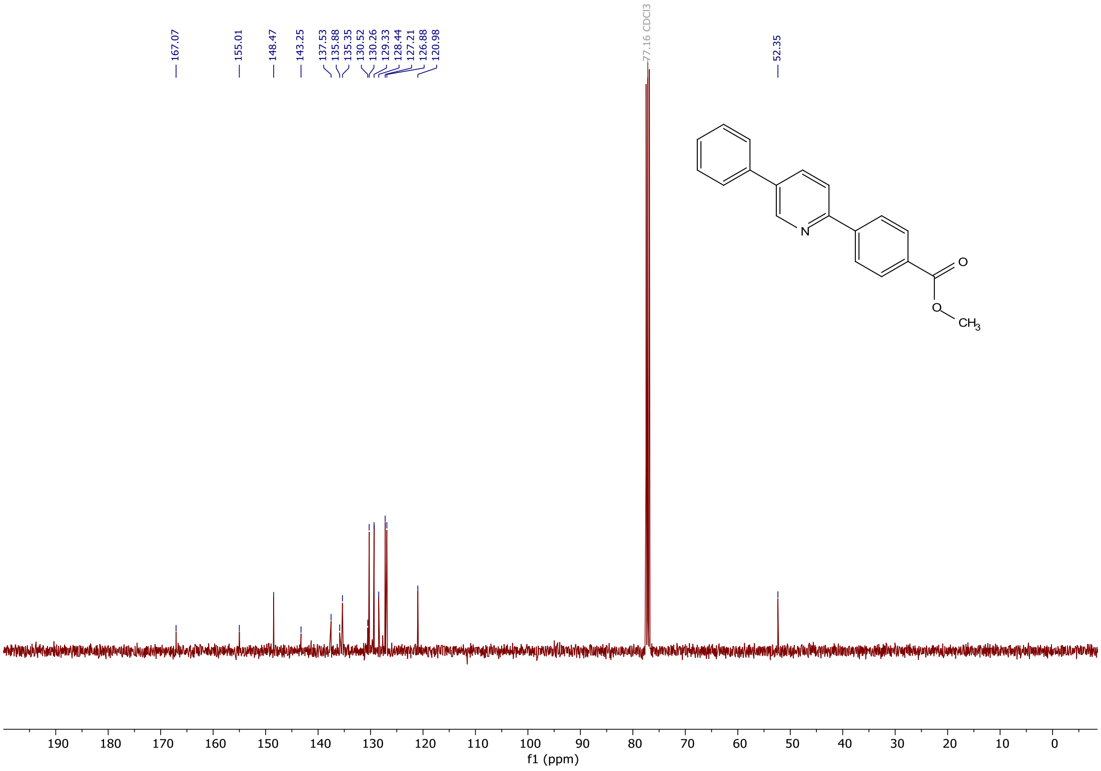

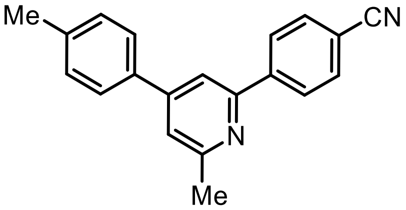

4-(6-methyl-4-(p-tolyl)pyridin-2-yl)benzonitrile (14)

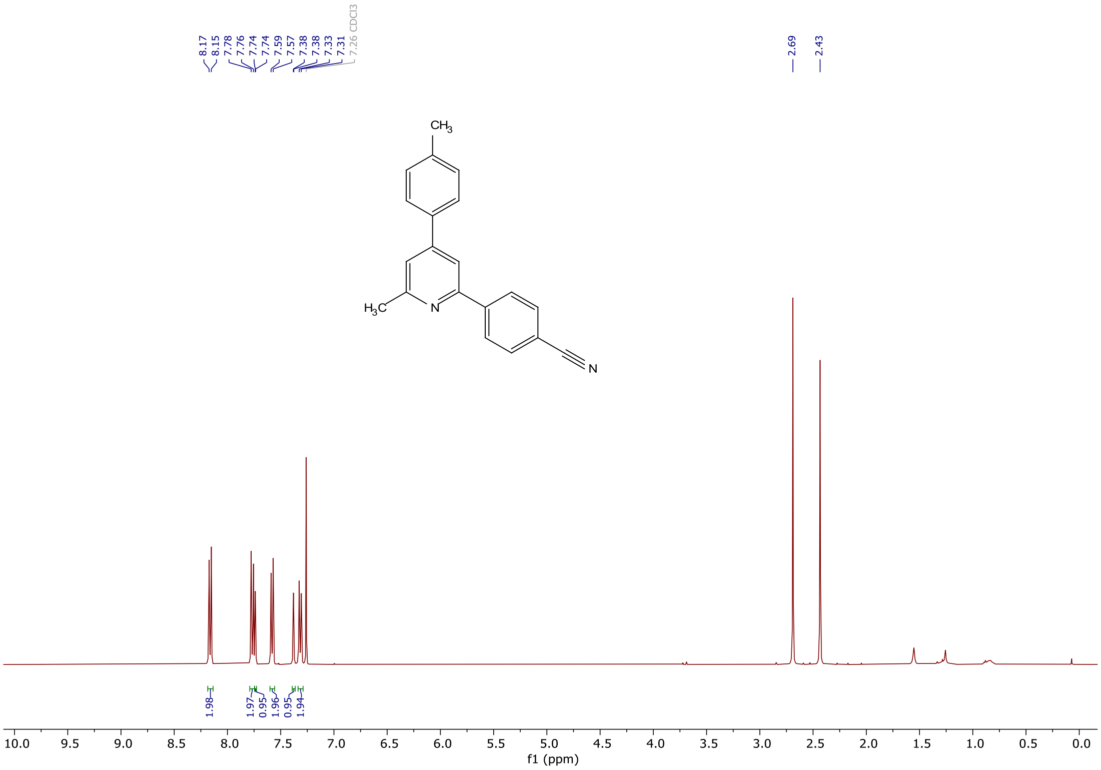

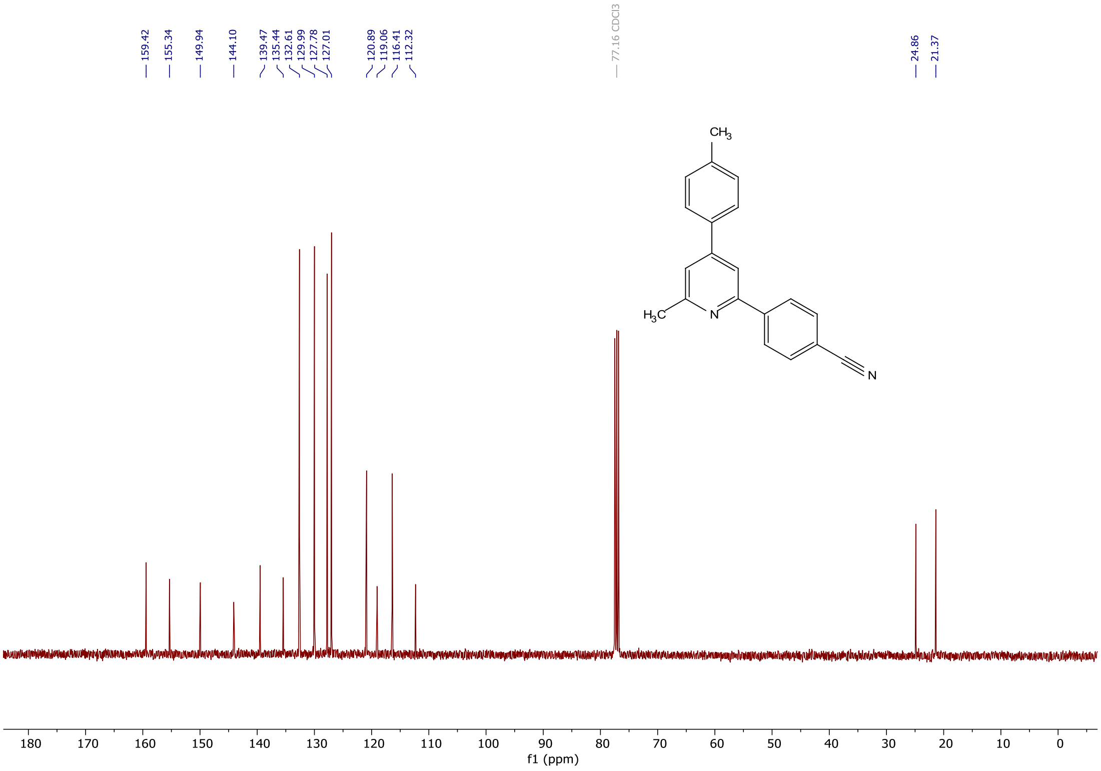

4.5 NMR Spectra of derivatisation compounds

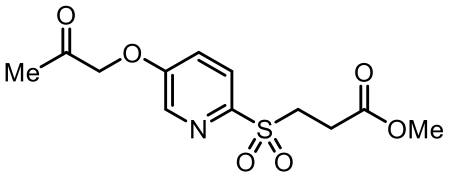

methyl 3-((5-(2-oxopropoxy)pyridin-2-yl)sulfonyl)propanoate (7)

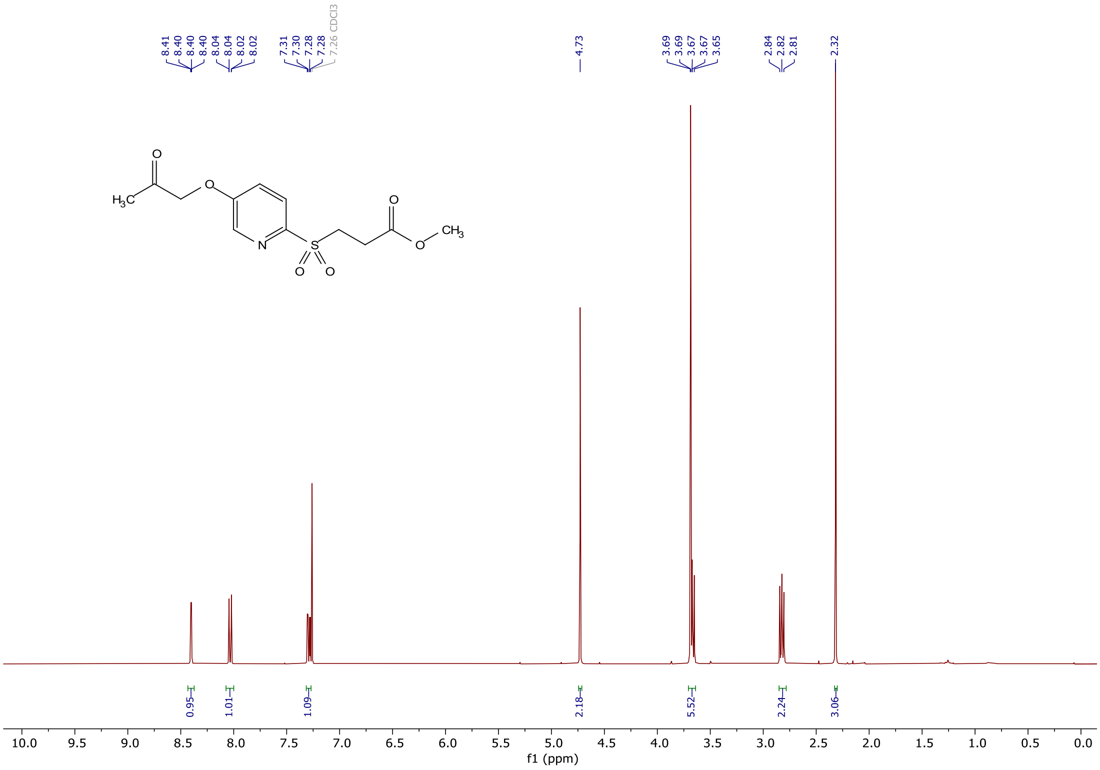

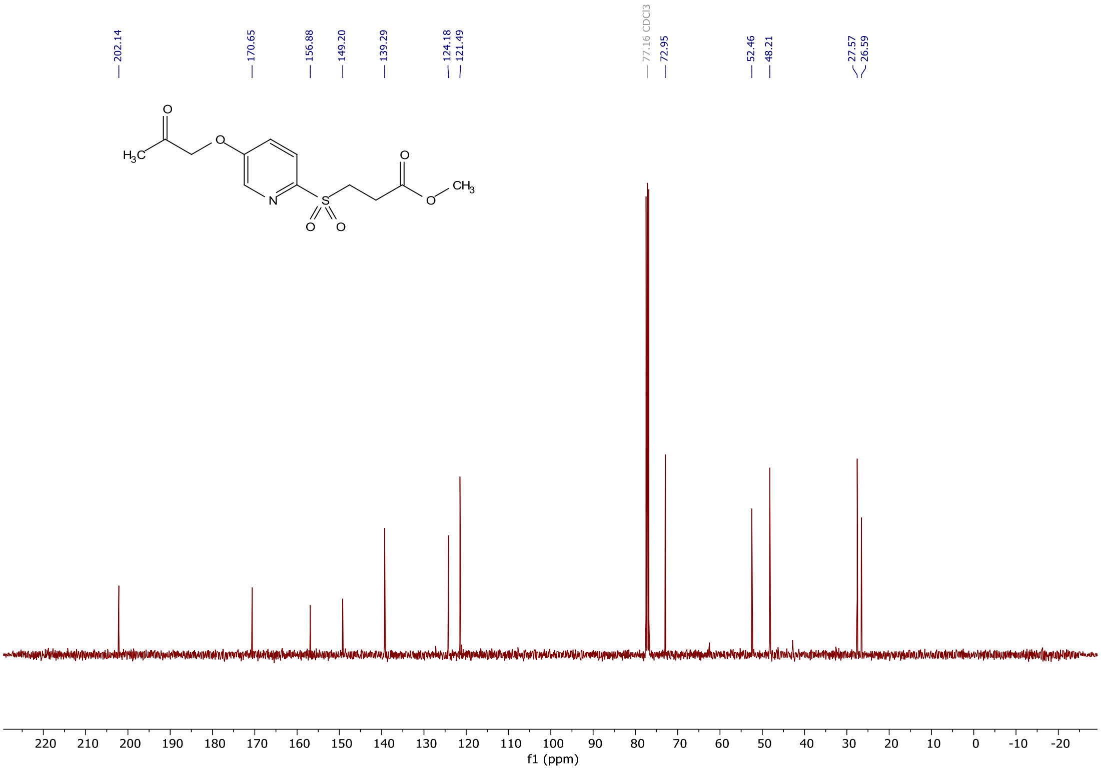

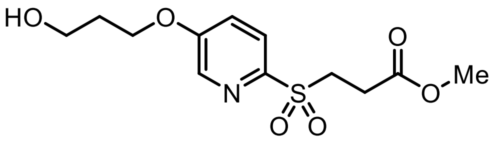

methyl 3-((5-(3-hydroxypropoxy)pyridin-2-yl)sulfonyl)propanoate (8a)

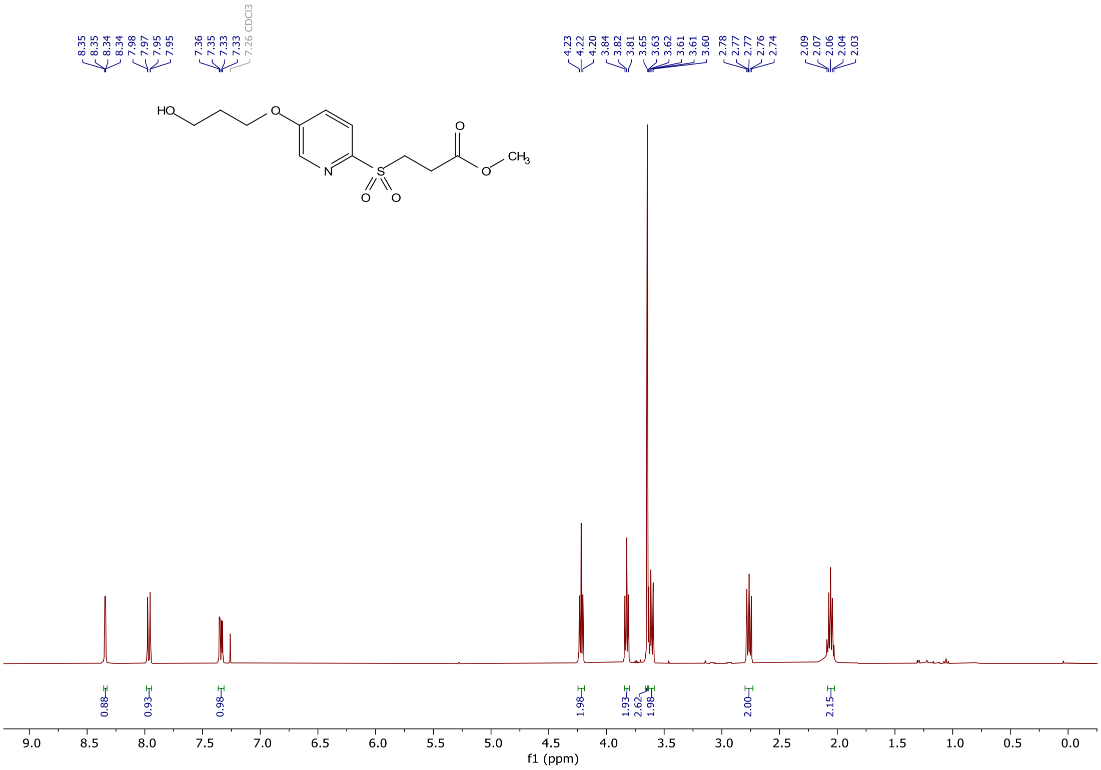

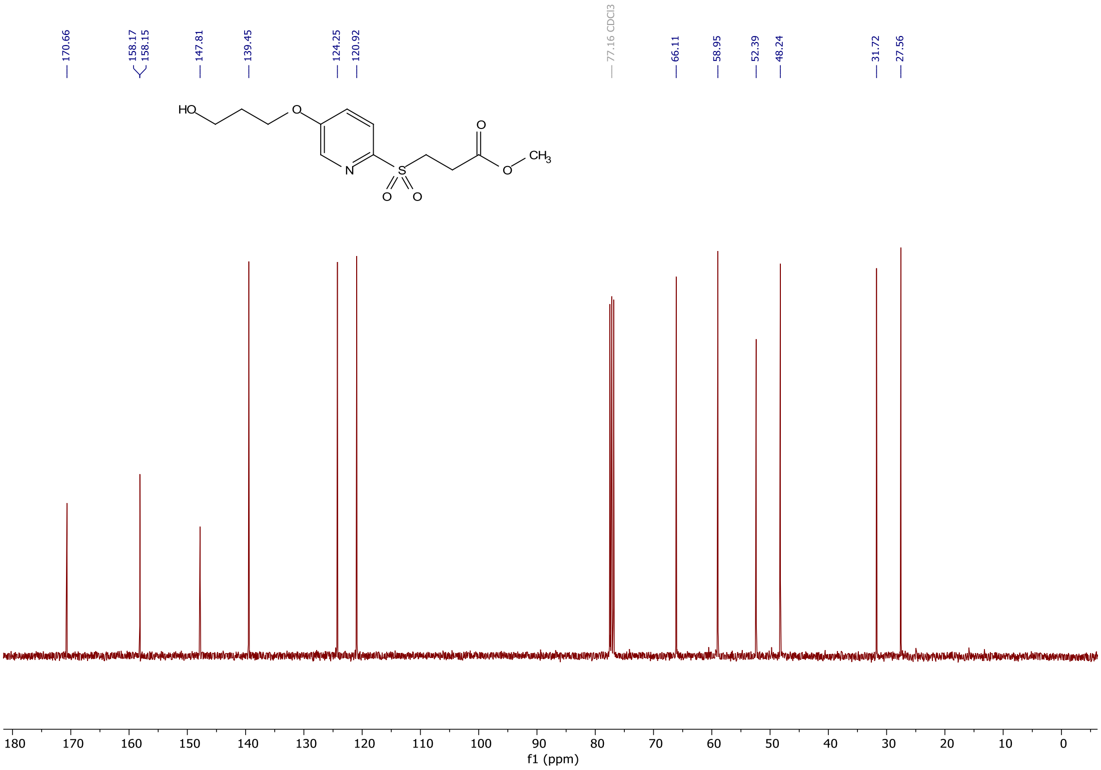

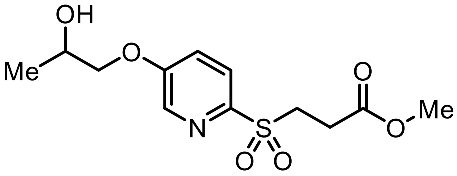

methyl 3-((5-(2-hydroxypropoxy)pyridin-2-yl)sulfonyl)propanoate (8b)

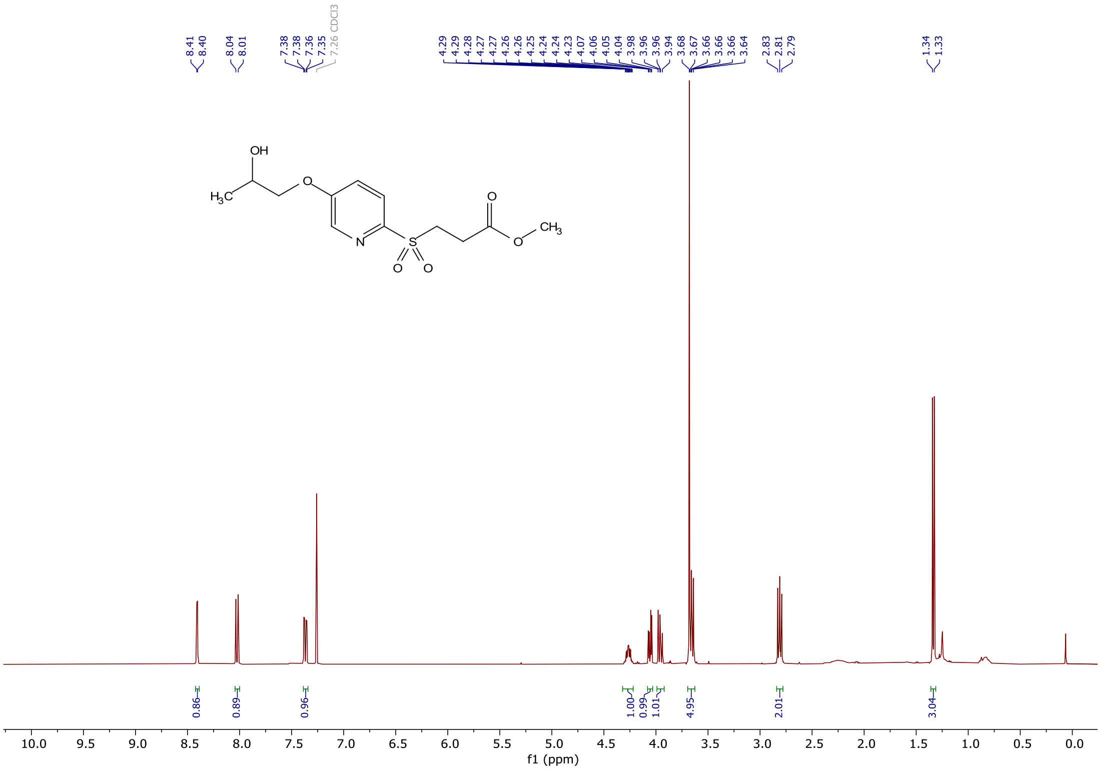

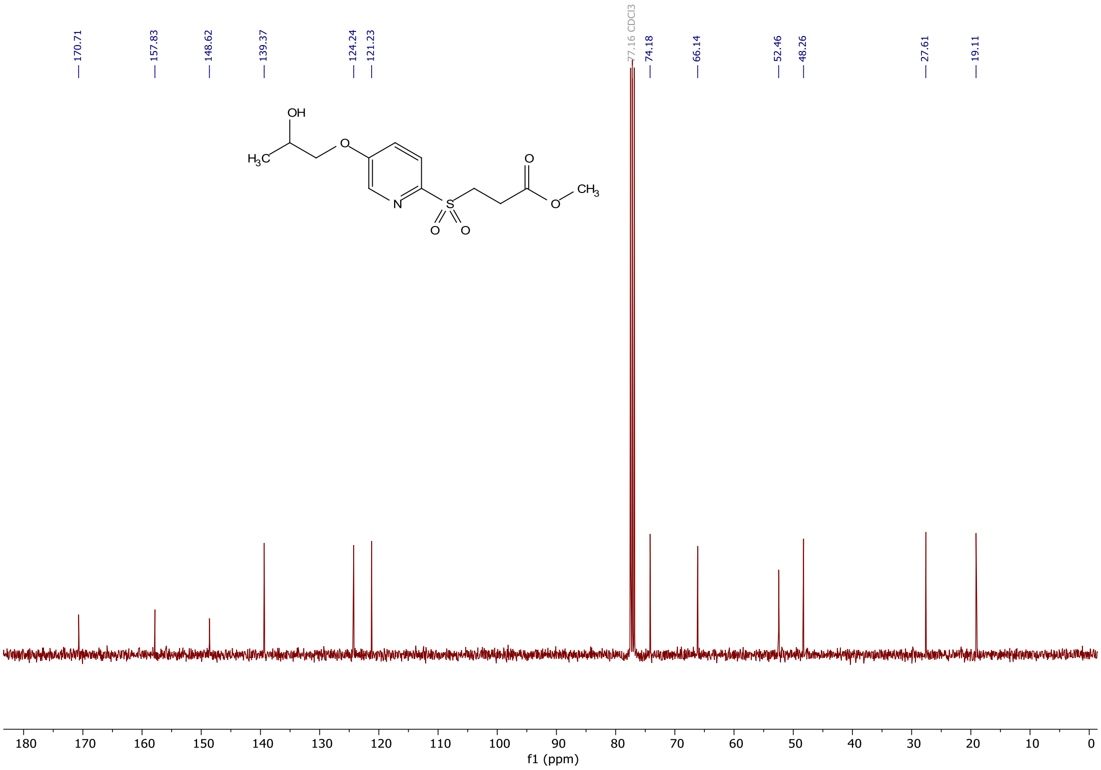

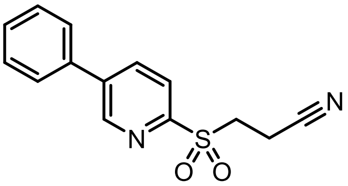

3-((5-phenylpyridin-2-yl)sulfonyl)propanenitrile (10a)

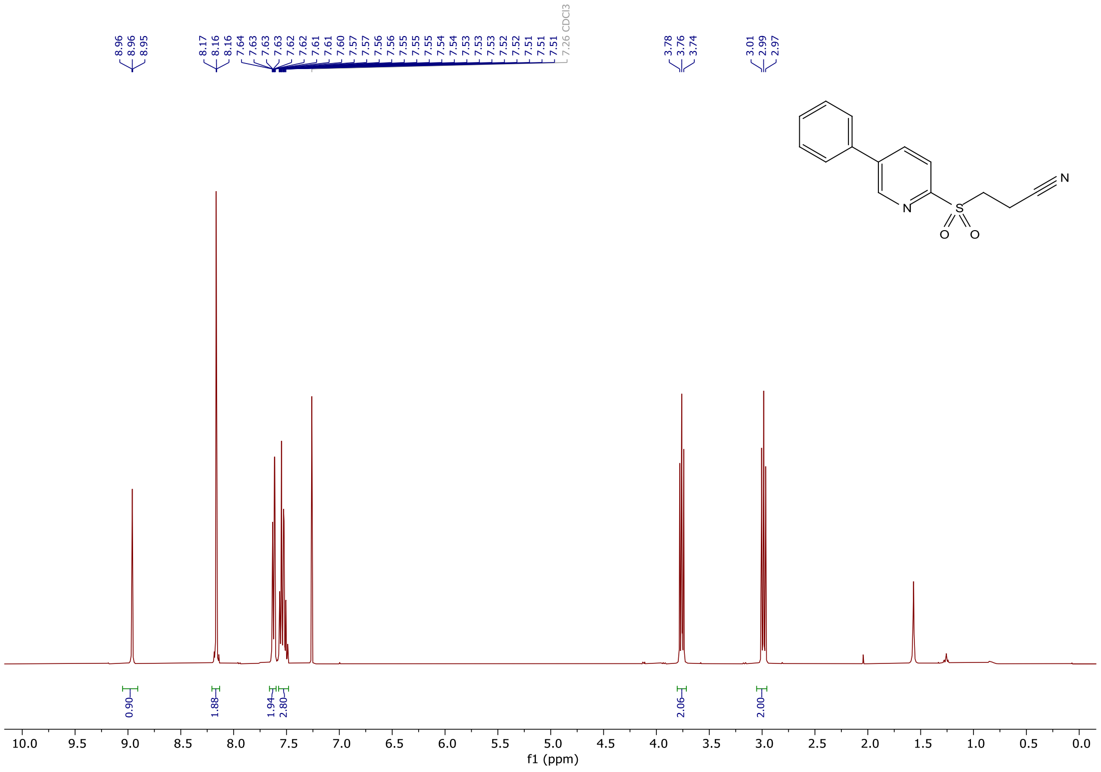

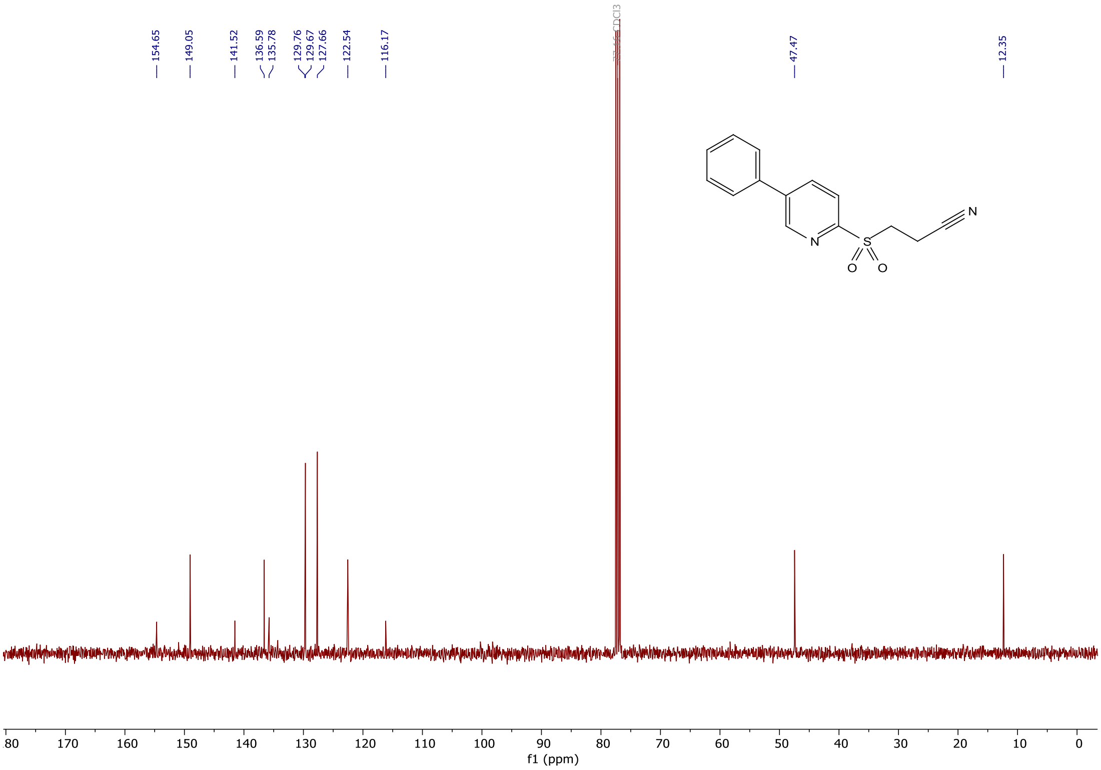

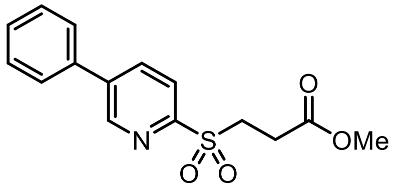

methyl 3-((5-phenylpyridin-2-yl)sulfonyl)propanoate (10b)

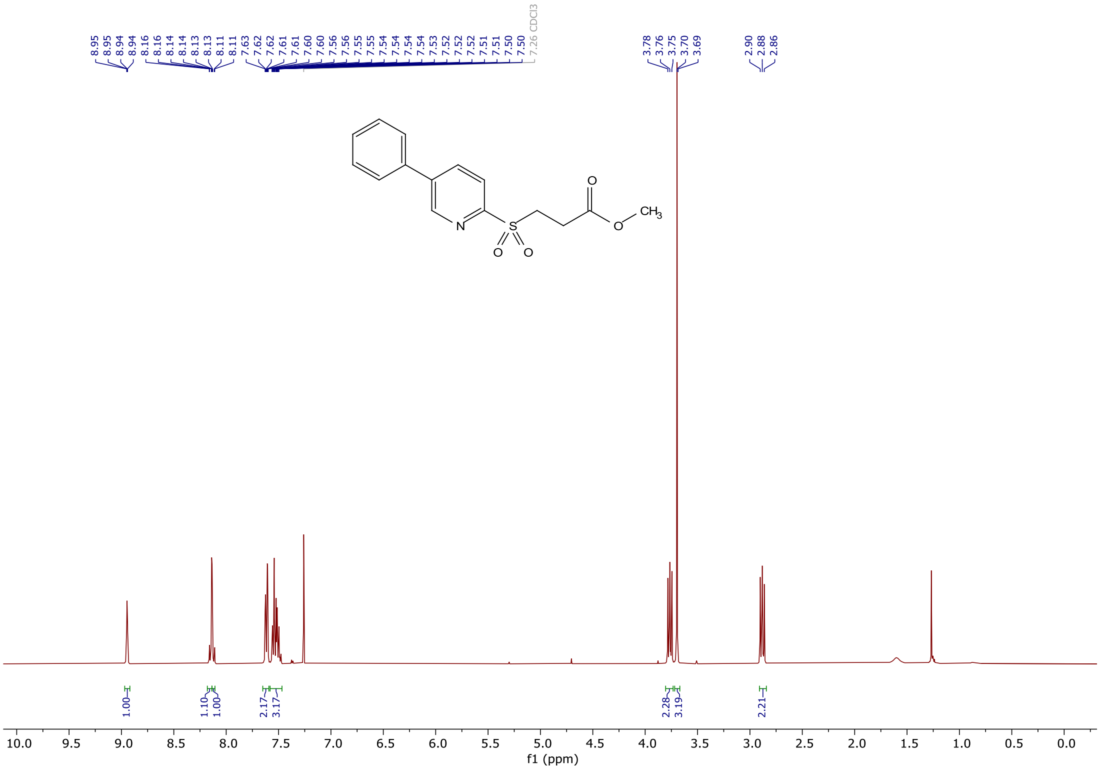

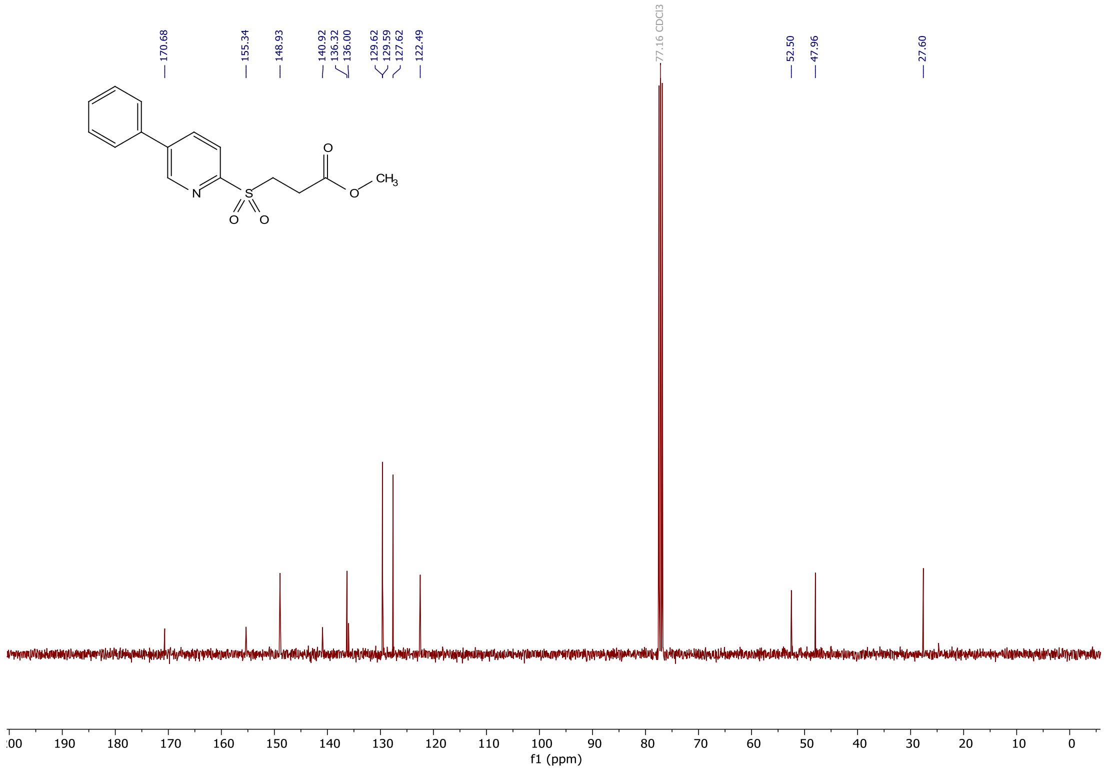

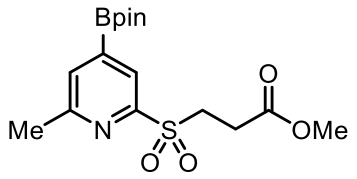

methyl 3-((6-methyl-4-(4,4,5,5-tetramethyl-1,3,2-dioxaborolan-2-yl)pyridin-2-yl)sulfonyl)propanoate (12)

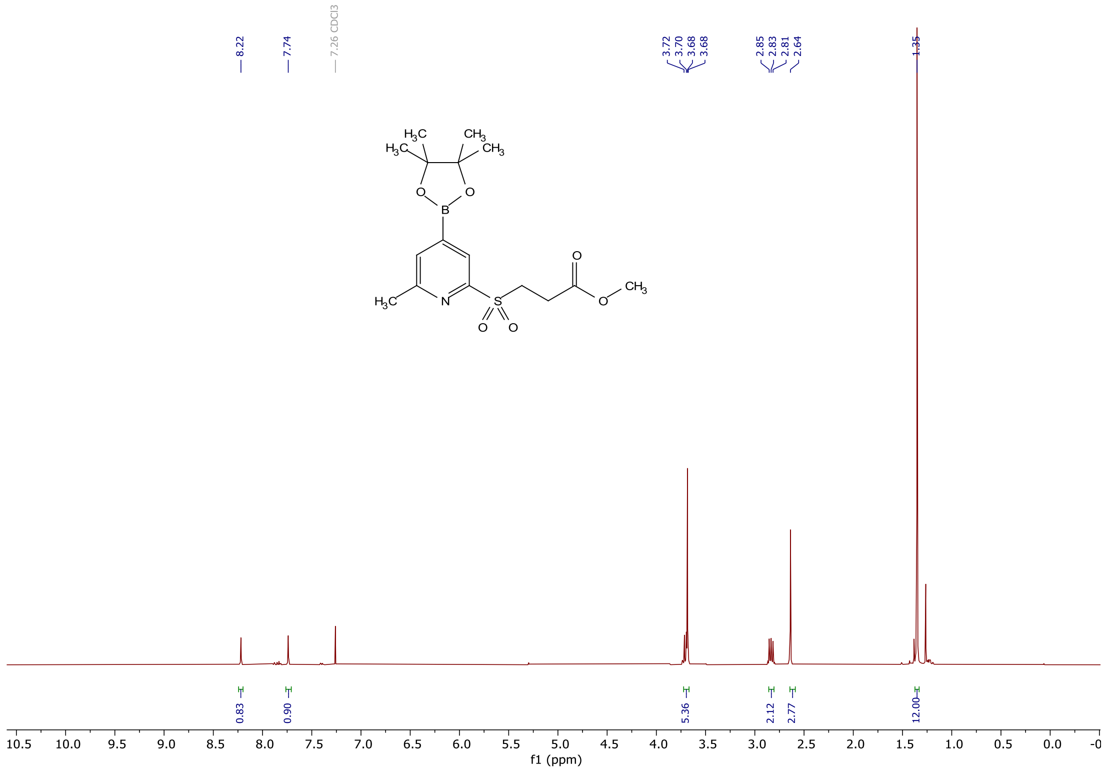

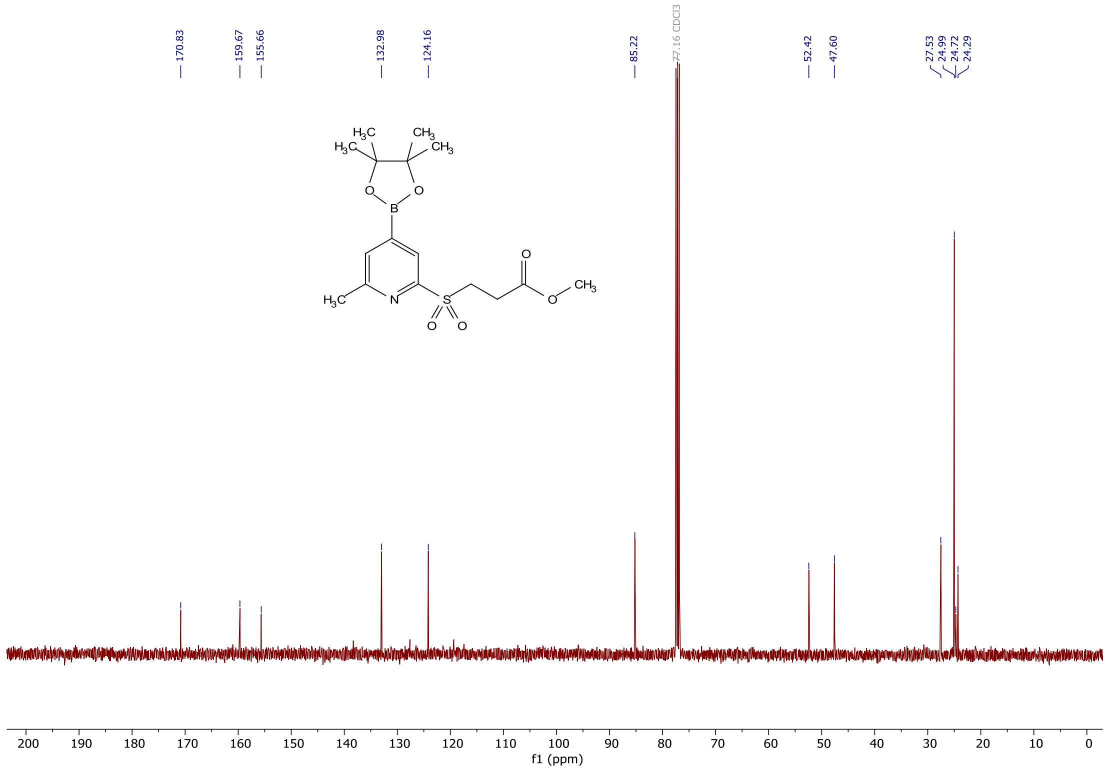

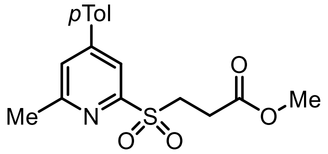

methyl 3-((6-methyl-4-(p-tolyl)pyridin-2-yl)sulfonyl)propanoate (13)

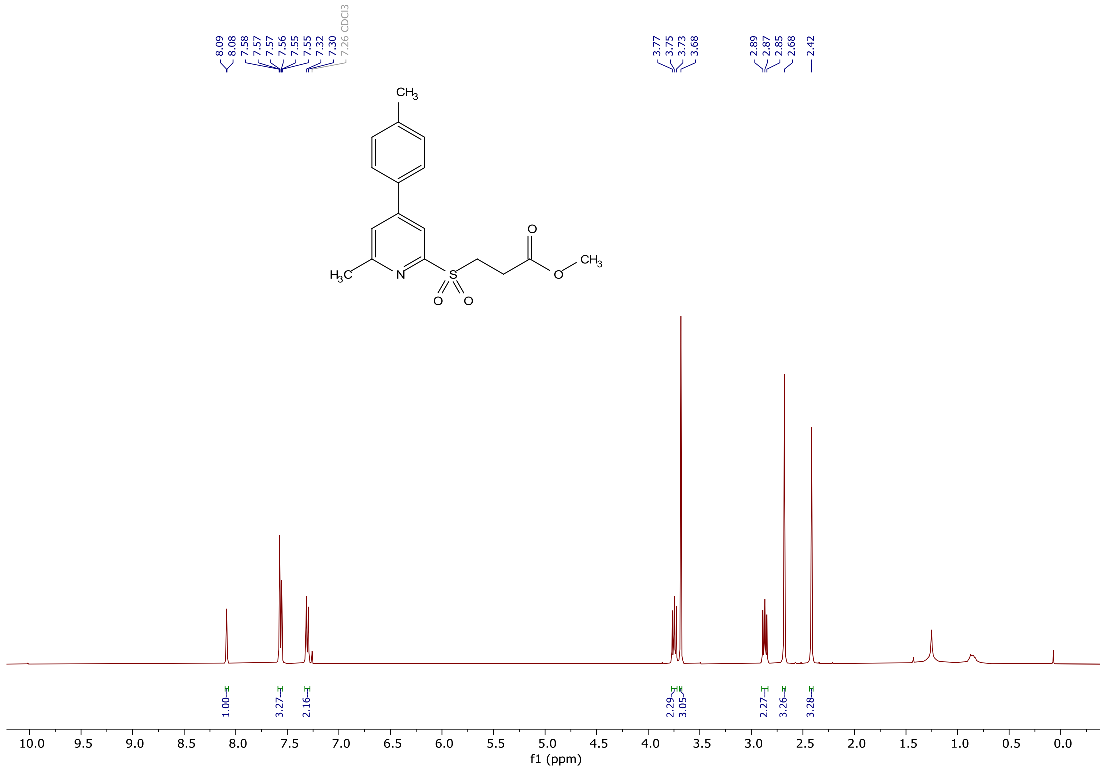

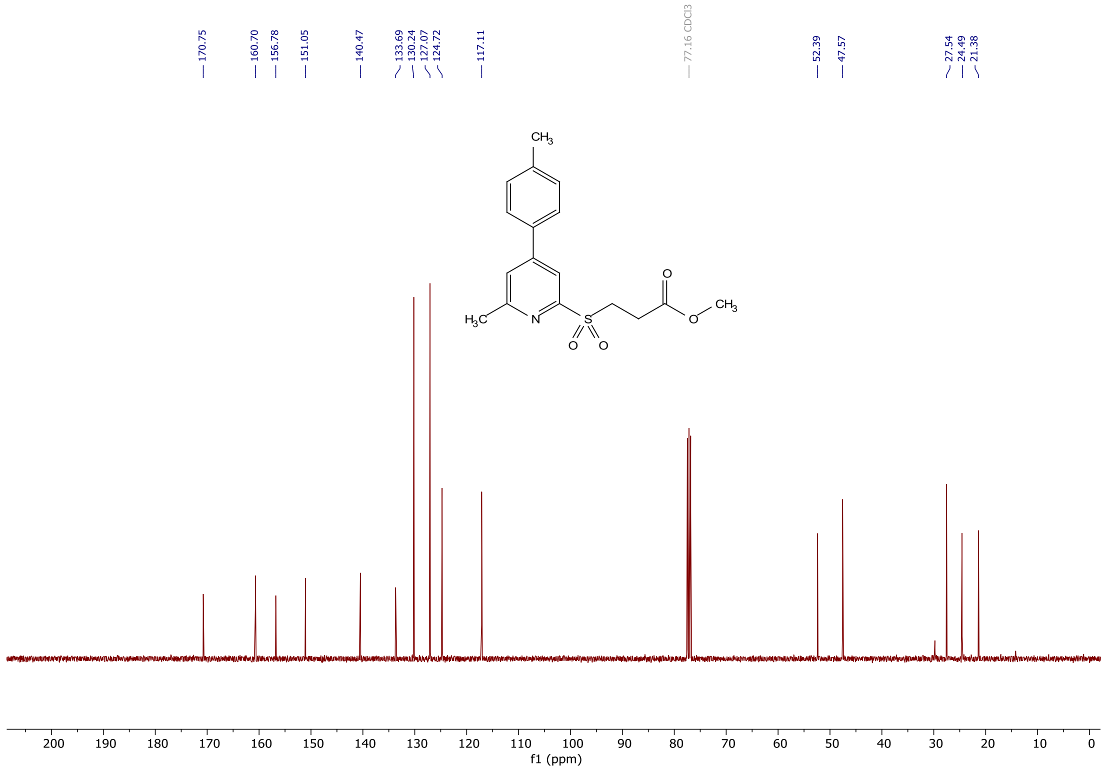

5. References

[1] J. Wei, H. M. Liang, C. F. Ni, R. Sheng, J. B. Hu, *Org. Lett.* **2019**, *21*, 937-940.

[2] M. Gholinejad, H. Firouzabadi, *New J. Chem.* **2015**, *39*, 5953-5959.

[3] L. Shi, X. Liu, H. Zhang, Y. Jiang, D. Ma, *J. Org. Chem.* **2011**, *76*, 4200-4204.

[4] T. Hideo, F. Misa, Y. Masataka, *B. Chem. Soc. Jpn.* **1991**, *64*, 57-67.

[5] J. M. Baskin, Z. Y. Wang, *Tetrahedron Lett.* **2002**, *43*, 8479-8483.

[6] B. Du, P. Qian, Y. Wang, H. Mei, J. Han, Y. Pan, *Org. Lett.* **2016**, *18*, 4144-4147.

[7] W. Li, L. Gao, W. Zhuge, X. Sun, G. Zheng, *Org. Biomol. Chem* **2017**, *15*, 7819-7823.

[8] H. E. Bartrum, D. C. Blakemore, C. J. Moody, C. J. Hayes, *Tetrahedron* **2013**, *69*, 2276-2282.

[9] T. Markovic, B. N. Rocke, D. C. Blakemore, V. Mascitti, M. C. Willis, *Chem. Sci.* **2017**, *8*, 4437-4442.

[10] A. de Gombert, A. I. McKay, C. J. Davis, K. M. Wheelhouse, M. C. Willis, *J. Am. Chem. Soc.* **2020**, *142*, 3564-3576.

[11] K. Sato, M. Hyodo, M. Aoki, X. Q. Zheng, R. Noyori, *Tetrahedron* **2001**, *57*, 2469-2476.

[12] Y. Xie, H. W. Chi, A. Y. Guan, C. L. Liu, H. J. Ma, D. L. Cui, *J. Agr. Food Chem.* **2014**, *62*, 12491-12496.

[13] T. Markovic, B. N. Rocke, D. C. Blakemore, V. Mascitti, M. C. Willis, *Org. Lett.* **2017**, *19*, 6033-6035.

[14] A. Gavryushin, C. Kofink, G. Manolikakes, P. Knochel, *Tetrahedron* **2006**, *62*, 7521-7533.

[15] M. F. Zheng, P. Q. Chen, W. Q. Wu, H. F. Jiang, *Chem. Commun.* **2016**, *52*, 84-87.

[16] K. Cooper, M. J. Fray, M. J. Parry, K. Richardson, J. Steele, *J. Med. Chem.* **1992**, *35*, 3115-3129.

[17] C. Liu, W. B. Yang, *Chem. Commun.* **2009**, 6267-6269.
[18] W. Li, J. Tang, S. Li, X. Zheng, M. Yuan, B. Xu, W. Jiang, F. Haiyan, R. Li, H. Chen, *Organic Letters* **2020**, *22*, 7814-7819.
[19] N. A. Isley, F. Gallou, B. H. Lipshutz, **2013**, *135*, 17707-17710.
[20] C. Fricke, G. J. Sherborne, I. Funes-Ardoiz, E. Senol, S. Guven, F. Schoenebeck, *Angew. Chem. Int. Ed.* **2019**, *58*, 17788-17795.
[21] V. Bonnet, F. Mongin, F. Trécourt, G. Quéguiner, P. Knochel, *Tetrahedron* **2002**, *58*, 4429-4438.
[22] X. Lin, C. Hou, H. Li, Z. Weng, *Chem. Eur. J.* **2016**, *22*, 2075-2084.
[23] A. Y. Dubovtsev, N. V. Shcherbakov, D. V. Dar'In, V. Y. Kukushkin, *J. Org. Chem.* **2020**, *85*, 745-757.
[24] X. Yu, Z. Zhang, R. Song, L. Gou, G. Wang, *Heterocycl. Commun.* **2020**, *26*, 1-5.
[25] S. Y. Lee, C.-H. Cheon, *J. Org. Chem.* **2018**, *83*, 13036-13044.
[26] D. A. Chaudhari, R. A. Fernandes, *J. Org. Chem.* **2016**, *81*, 2113-2121.
[27] D. Hédou, A. S. Voisin-Chiret, *Eur. J. Org. Chem.* **2020**, *2020*, 3640-3649.
[28] E. Demory, K. Devaraj, A. Orthaber, P. J. Gates, L. T. Pilarski, *Angew. Chem. Int. Ed.* **2015**, *54*, 11765-11769.
[29] T. S.-B. Lou, M. C. Willis, *Tetrahedron* **2020**, *76*, 130782.
[30] O. S. Kanishchev, W. R. Dolbier, *Angew. Chem. Int. Ed.* **2015**, *54*, 280-284.
[31] P. Ye, Y. Shao, F. Zhang, J. Zou, X. Ye, J. Chen, *Adv. Synth. Catal.* **2020**, *362*, 851-857.
